# Oxidative Cross-Coupling of α-Amino Ketones with Alcohols Enabled by I_2_-Catalyzed C–H Hydroxylation

**DOI:** 10.1021/acs.joc.3c01469

**Published:** 2023-10-09

**Authors:** Yingwei Wang, Mingrong Yang, Chichou Lao, Hanxuan Wang, Zhihong Jiang

**Affiliations:** † State Key Laboratory of Quality Research in Chinese Medicines, Macau University of Science and Technology, Macau 999078, China; ‡ School of Chemical Engineering, Sichuan University of Science & Engineering, Zigong 643000, China

## Abstract

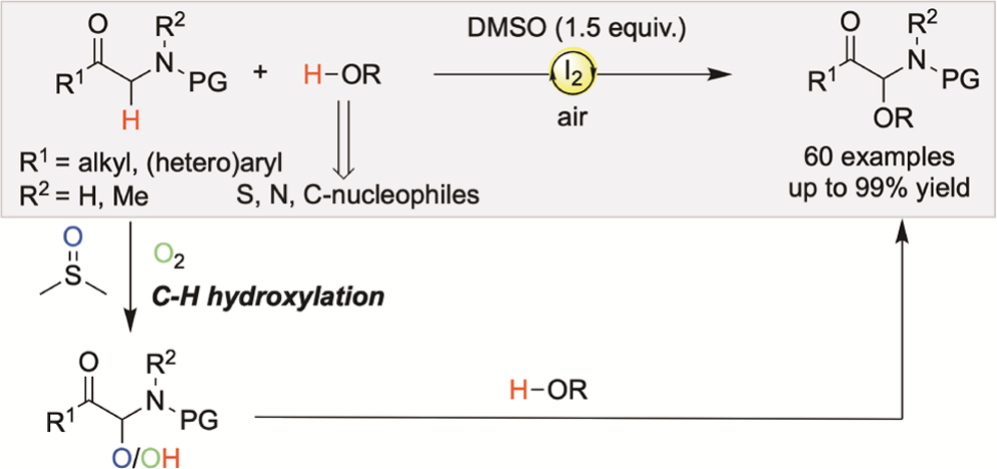

An I_2_-catalyzed oxidative cross-coupling of α-amino ketones with a wide range of alcohols is described. Using a combination of air and dimethyl sulfoxide (DMSO) as oxidants, the protocol allows an efficient synthesis of α-carbonyl *N,O*-acetals with high functional group tolerance and enables the late-stage introduction of α-amino ketones into biorelevant alcohols. Moreover, the present method can be used in the coupling of α-amino ketones with other kinds of nucleophiles, which demonstrates great generality for the functionalization of α-amino ketones. A preliminary mechanistic investigation suggests that C–H hydroxylation of α-amino ketones has been recognized as the key step followed by subsequent dehydration coupling.

## Introduction

In recent years, molecular iodine (I_2_)-catalyzed oxidative cross-coupling reactions in the presence of terminal oxidants, such as dimethyl sulfoxide (DMSO) and O_2_, have attracted great interest owing to the facts that I_2_ is recognized as an inexpensive, insensitive to air and moisture, nontoxic, environmentally begin, and easy-to-handle catalyst under mild reaction conditions.^1^ According to the literature, we found that a few examples of C–O bond formation reactions have been reported;^2^ however, the oxidizing ability of I_2_ limits alcohols as coupling partners applied in oxidative cross-coupling reactions.^3^

**Figure 1:**
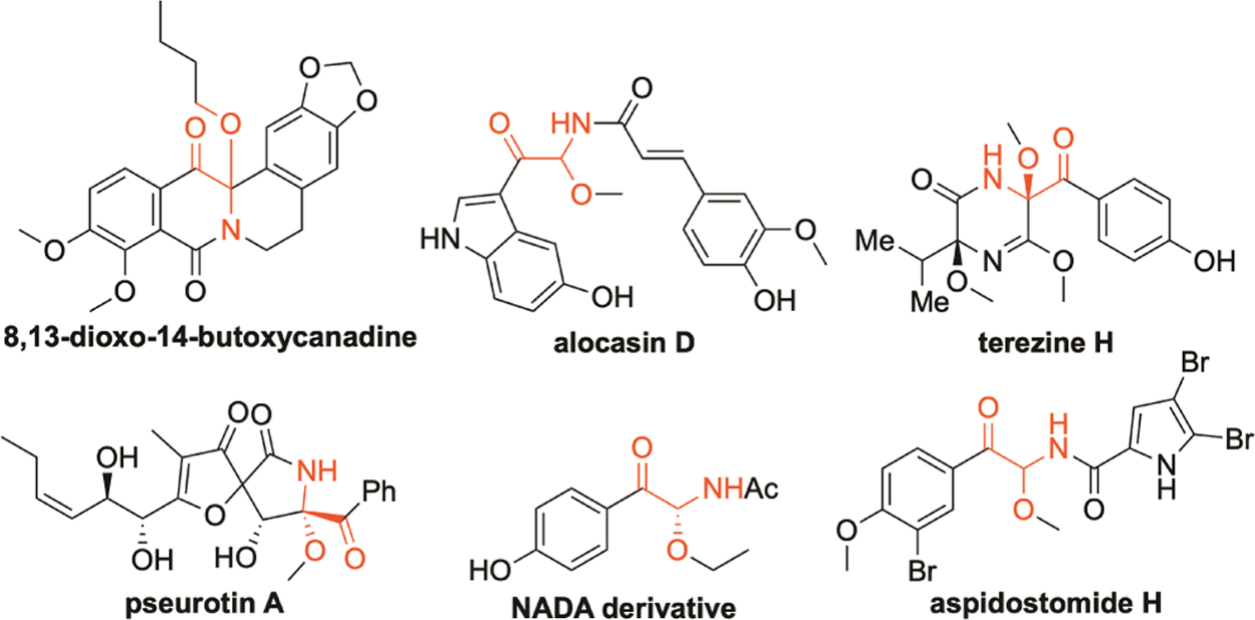
Natural products containing α-carbonyl *N,O*-acetals.

**Scheme 1:**
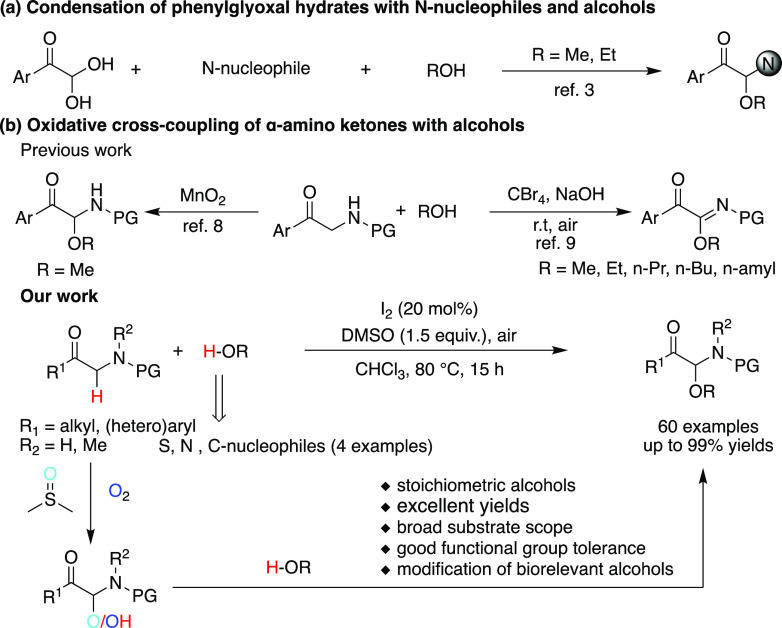
Available Synthetic Routes to α-Carbonyl *N,O*-Acetals with Alcohols

α-Carbonyl *N,O*-acetals are ubiquitous structural units found in indispensable building blocks^4^ as well as many biologically active molecules and natural products,^5^ such as 8,13-dioxo-14-butoxycanadine, alocasin D, terezine H, pseurotin A, NADA derivative, and aspidostomide H (Figure 1). The classical synthetic methodologies generally involve a condensation of phenylglyoxal hydrates with *N*-nucleophiles and alcohols (Scheme 1a), which typically requires the alcohol component as a solvent or cosolvent.^6^ This condition limits the practical scope of these reactions to simple, inexpensive alcohols. Based on the high atom economy and environmentally friendly character, the oxidative cross-coupling of α-amino carbonyl compounds with nucleophiles has lately become a powerful and practical means for the assembly of structurally diversified α-amino acid derivatives.^7,8^ The direct oxidative C–O bond formation of α-amino carbonyl compounds has been well studied by employing *O*-nucleophiles including phenols^9^ and carboxylic acids;^10^ however, methods that enable the direct oxidative C–H alkoxylation of α-amino carbonyl compounds with alcohols have been rarely reported. In 1981, Proctor and co-worker revealed oxidative cross-coupling of *N*-benzoyl-2-aminoacetophenone with methanol in low yields, which required manganese dioxide as oxidant and alcohols as solvent.^11^ Although Huang’s group developed a CBr_4_-mediated oxidative cross-coupling reaction of α-amino ketones with simple primary alcohols, the elimination of N–H bonds could not be suppressed (Scheme 1b).^12^ In addition, the above strategies generally limit the substrates to α-amino arylketones that are more reactive and may shield regioselectivity. Therefore, a more synthetically useful, general, and metal-free method to α-carbonyl *N,O*-acetals requires a new approach that operates at a more reasonable stoichiometry for enabling coupling with more structurally complex alcohols.

## Results and Discussion

As a continuous effort on C–H functionalization of α-amino ketones,^13^ we herein report a direct, general, metal-free, and practical method for the synthesis of α-carbonyl *N,O-*acetals by developing an I_2_-catalyzed oxidative cross-coupling reaction between α-amino ketones and alcohols by using a combination of DMSO and air as oxidants. The protocol features mild reaction conditions, excellent yields, and a broad substrate scope. More Importantly, the compatibility of standard conditions with diverse nucleophiles to expand the landscape of nucleophilic coupling partners makes this reaction particularly attractive (Scheme 1b).

We initiated our investigation on the reactions between α-amino carbonyl compounds **1a**–**d** and benzyl alcohol (**2a**), and the results are summarized in Table 1. When 20 mol % *N*-bromosuccinimide (NBS) was used, both glycine ester **1a** and glycine amide **1b** did not react with **2a** in the presence of DMSO under air (entries 1–2), while **1c** bearing higher reactivity gave a complicated mixture (entry 3). To our delight, substrate **1d** could react with **2a** to provide the desired product **3d** in 72% yield (entry 4). Then, we tried to optimize this transformation by using other halide reagents in CHCl_3_. NIS was more efficient than NBS in this transformation, whereas *N*-chlorosuccinimide (NCS) hardly produced the desired product (entries 5–6). Significantly, 87% yield of **3d** was obtained when I_2_ was used as a catalyst (entry 7). Among various solvents screened (entries 7–13), CHCl_3_ proved to be the most efficient solvent. A higher temperature (80 °C) could improve the yield of oxidative coupling product **3d** to 96% and shorten the reaction time to 15 h (entry 14).

**Table 1:**
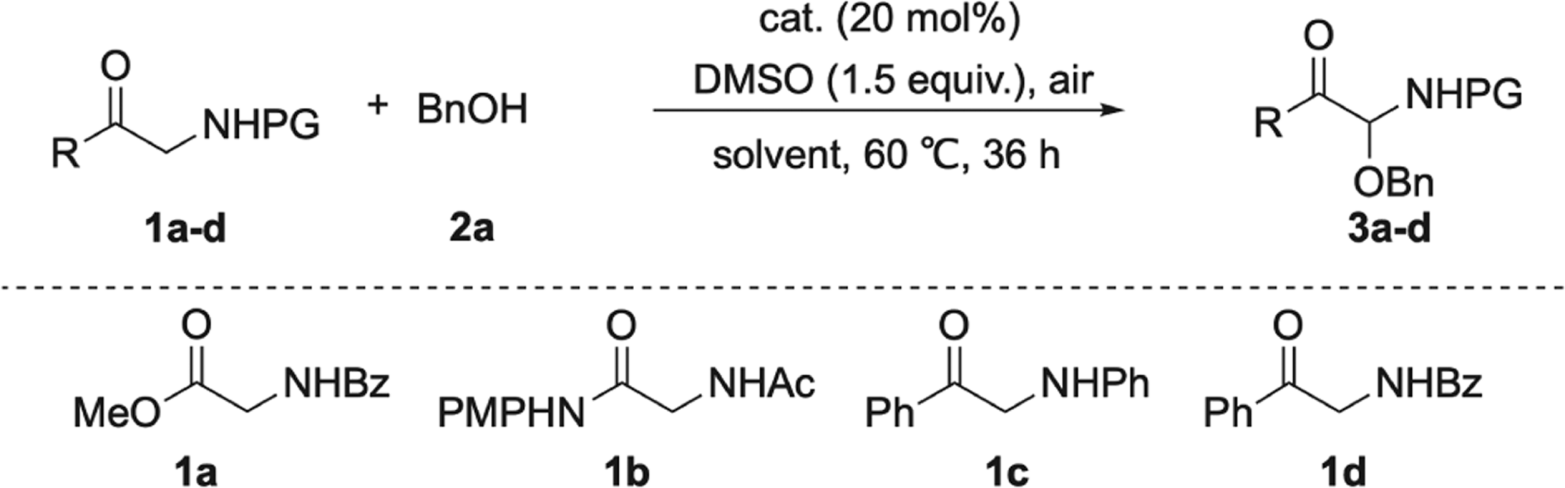
Optimization of Reaction Conditions^a^

entry	**1**	cat.	solvent	yield (%)^b^
1	**1a**	NBS	CHCl_3_	NR^c^
2	**1b**	NBS	CHCl_3_	NR^c^
3	**1c**	NBS	CHCl_3_	0
4	**1d**	NBS	CHCl_3_	72
5	**1d**	NCS	CHCl_3_	6
6	**1d**	NIS	CHCl_3_	83
7	**1d**	I_2_	CHCl_3_	87
8	**1d**	I_2_	PhMe	81
9	**1d**	I_2_	MeCN	45
10	**1d**	I_2_	EtOAc	22
11	**1d**	I_2_	THF	38
12	**1d**	I_2_	DCM	74
13	**1d**	I_2_	DCE	72
14^d^	**1d**	I_2_	CHCl_3_	96

a Reaction conditions: **1** (0.2 mmol), **2a** (0.24 mmol), DMSO (0.3 mmol), cat. (20 mol %), and solvent (1 mL) at 60 °C under air for 36 h.

b Isolated yield.

c No reaction.

d The reaction was run at 80 °C for 15 h.

With the optimal conditions in hand, the scope of alcohols **2** and α-amino ketones **1** was examined (Table 2). Satisfyingly, a variety of alcohols smoothly underwent the oxidative cross-coupling reaction with **1d** to afford the corresponding α-carbonyl *N,O*-acetals **3e**–**z** in moderate to excellent yields. For simple primary alcohols, **3e**–**g** were obtained in 87–93% yields. Isopropanol and cyclohexanol could successfully react with substrate **1d** to furnish **3h** and **3i** efficiently in 84 and 89% yields, respectively. Using *dl*-1-phenethylalcohol, benzhydrol, or *tert*-butanol, the corresponding products **3j**–**l** were isolated in 27–61% yields, and the results indicated the steric effect was obvious. A range of diverse and densely functionalized primary alcohols provided C–O bond coupling products in good yields. Halohydrins (**3m**–**n**), triethylene glycol monomethyl ether (**3o**), 2-(trimethylsilyl)ethanol (**3p**), 2-nitroethanol (**3q**), and alcohols featuring olefin (**3r**), alkyne (**3s**), carboxylic ether (**3t**), sulfonate ether (**3u**), amide (**3v**–**x**), and azide (**3y**) functional groups were compatible with this protocol. The substrate **1d** was selectively coupled to the alcohol (**3w**) bearing a tertiary hydroxyl group in 69% yield. Significantly, late-stage modification of biorelevant alcohols was also performed by this protocol. Steroids, such as pregnenolone, dehydroepiandrosterone, and epiandrosterone, were transferred to their analogues **3aa-ac** in 59–80% yields. Protected carbohydrates were precisely modified to **3ad** in 38% yield. *N*-Fmoc-protected phenylalaninol was suitable to afford the desired product **3ae** in 61% yield. The success of these molecular modifications shows the practical applicability of this oxidative cross-coupling protocol in accessing new biorelevant molecules.

**Table 2:**
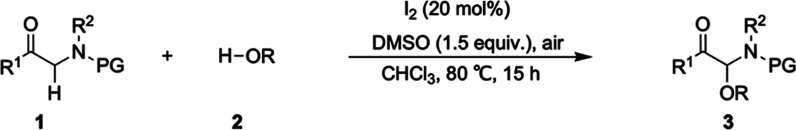

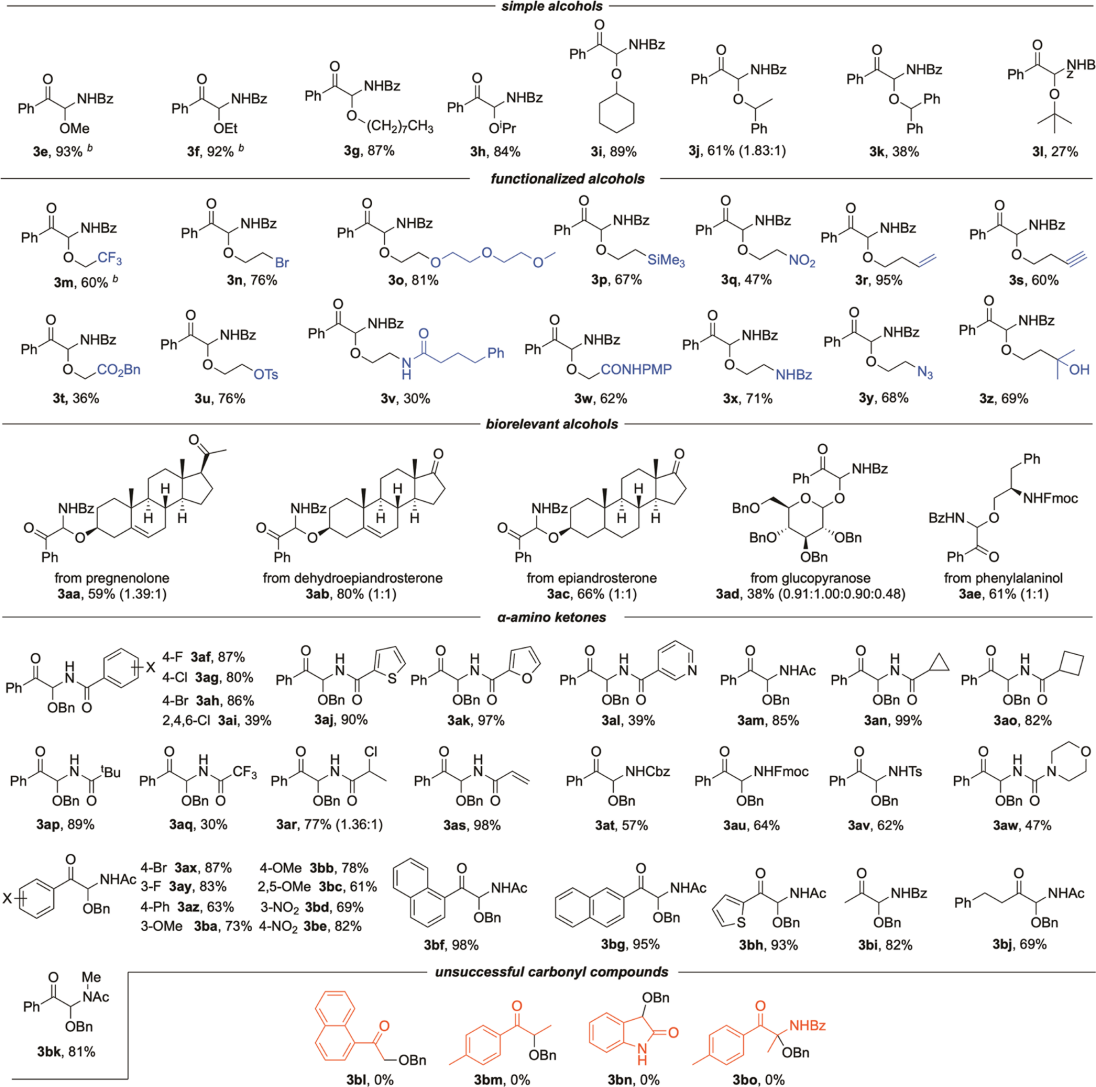
Scope of Substrates^a^

a Reaction conditions: **1** (0.2 mmol), **2** (0.24 mmol), DMSO (0.3 mmol), I_2_ (20 mol %) and CHCl_3_ (1 mL) at 80 °C under air for 15 h. The *dr* value is given in parentheses.

b 10.0 equiv of alcohols was used.

Subsequently, the scope of α-amino ketones was examined. Substrates with different halogens substituted at para position on the benzoamide group of **1d** performed the reaction readily with **2a** to provide the corresponding α-carbonyl *N,O*-acetals **3af**-**ah** in high yields. For 2,4,6-chloro substitution, significant steric hindrance was observed (**3ai**). The heterocyclic substrates also gave the desired products **3aj-al** in 39–90% yields. *N*-Acyl-2-aminoacetophenones bearing fundamental alkyls on the amide carbonyl moiety were found to be suitable coupling partners, giving rise to the corresponding products **3am-ap** in 82–99% yields. In addition, α-fluoro and chloro amides were also able to react well with **2a**, delivering the expected products **3aq-ar** in moderate yields. The introduction of a vinyl group had no effect on product (**3as**) formation. To our delight, when a carbamate such as *N*-Cbz- or *N*-Fmoc-protected substrate was subjected to the optimized reaction conditions, the corresponding products **3at-au** could be furnished in 57 and 64% yields, respectively. Furthermore, we changed the nitrogen-protecting group to *N*-Ts or *N*-carbamoyl and observed similar reactivity to give the title products **3av-aw** without a significant decrease in yields. Then, different substituents on the arene moiety of acetophenone were introduced. For example, the substrates bearing Br, F, Ph, OMe, and NO_2_ groups all survived and gave the target molecules **3ax-3be** in good to high yields. The reaction was readily expanded to 1-naphthoyl or 2-naphthoyl substrates, albeit with high yields (**3bf-bg**). When the substrate with a 2-thiophenecarbonyl moiety was used, the coupling reaction could give the product **3bh** in 93% yield. The reaction was also found to be applicable to *N*-benzoyl-2-aminoalkylketones, affording the desired products **3bi** and **3bj** in 82 and 69% yields, respectively. *N*-Methyl-*N*-aceyl-2-aminoacetophenone was also tested and the product **3bk** was isolated in 81% yield, which indicated that N–H in the substrate was not necessary for the reaction. Furthermore, we tried to extend this protocol toward ketones (**3bl-bm**) and amide (**3bn**), but no desired products were isolated. Finally, α-amino ketone (**3bo**) that bears a tertiary C(sp^3^)-H bond was unfortunately not a suitable reaction partner.

Despite the significant developments of C–S^9^ and C–N^14^ bond formation, the oxidative cross-coupling of α-carbonyl amino compounds with *S*- or *N*-nucleophiles is still desirable. We performed further studies on the coupling reactions of α- amino ketone **1d** with different kinds of nucleophiles under standard conditions (Table 3). 4-Chlorobenzyl mercaptan and 4-(trifluoromethyl)benzenethiolate gave C–S bond coupling products **4a**–**b** in moderated yields (27–37%). 5-Chlorobenzotriazole was used as an *N*-nucleophile, and the corresponding product **4c** was formed in 47% yield. Benzofuran derivative **4d** obtained in satisfactory yield was synthesized through the tandem C–C bond coupling/dehydration condensation reaction between **1d** and 2-naphthol. The above results fully demonstrate the practicability, generality, and diversity of this protocol in direct functionalization of α-amino carbonyl compounds.

**Table 3:**
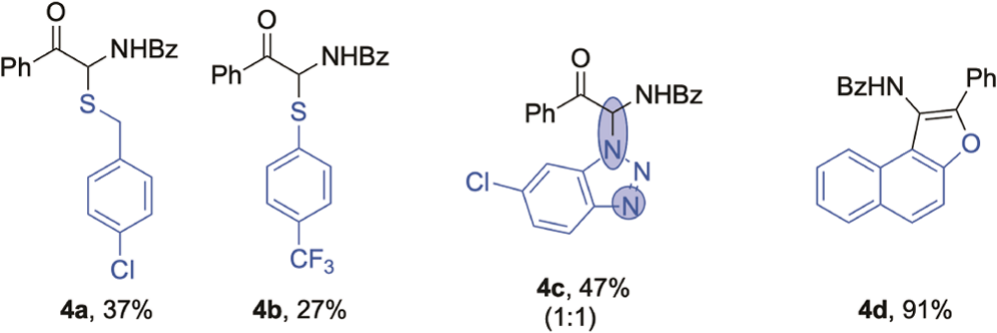
Coupling of **1d** with Other Nucleophiles^a^

a Standard conditions. The *rr* value is given in parentheses.

**Scheme 2:**
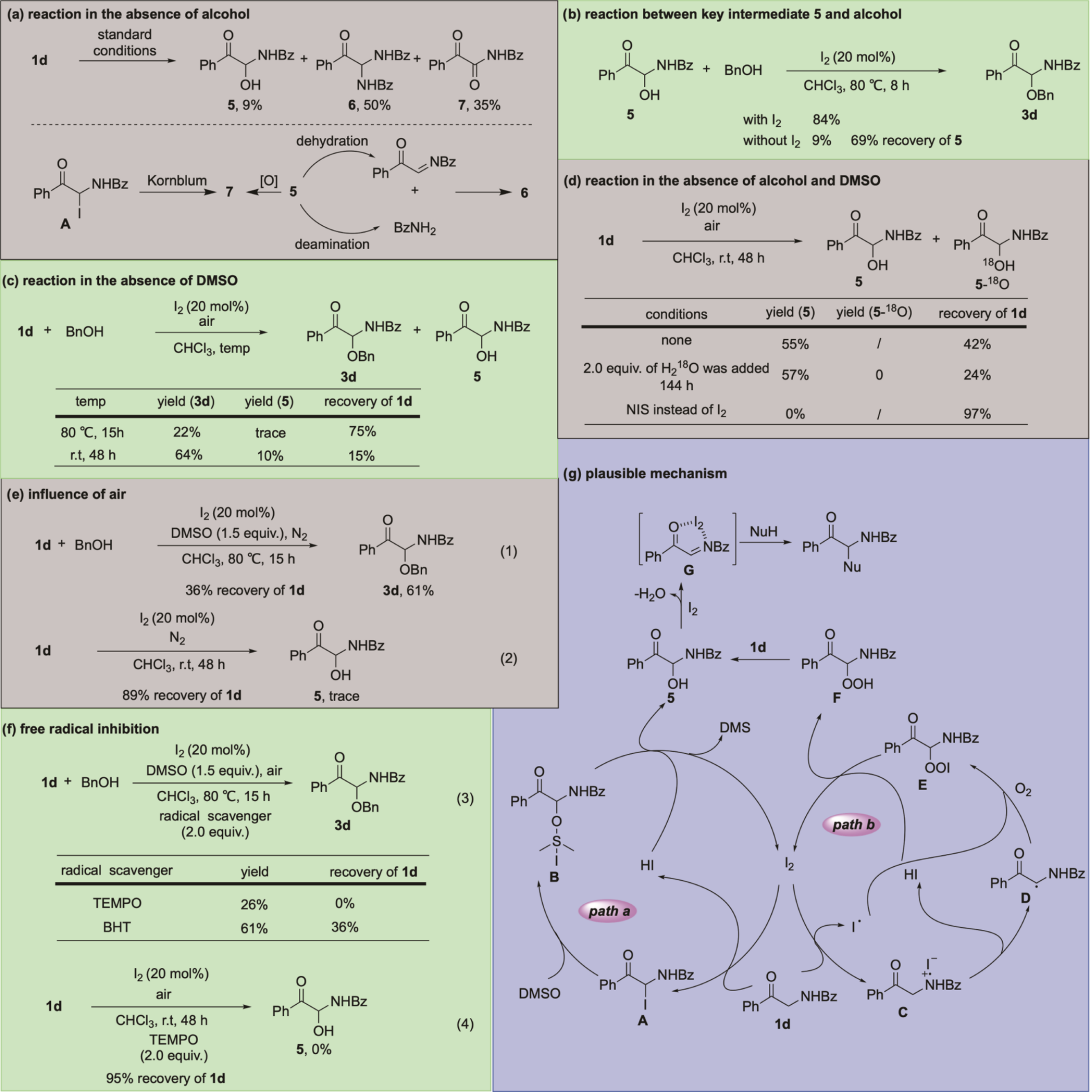
Control Experiments and Plausible Mechanism

To have a better understanding of this transformation, some control experiments were conducted (Scheme 2a–f). In the absence of alcohol, the substrate **1d** was converted to the hydroxylated product **5**, α-carbonyl *N,N*-acetal **6**, and benzoyl formamide **7** (Scheme 2a). The formation of **6** was caused by the reaction between the imine produced by dehydration of **5** and the benzamide formed from deamination of **5**, while the oxidation^15^ of **5** or Kornblum reaction^16^ of iodinated intermediate **A** would lead to **7**. Thus, we infer that **5** acts as the key intermediate for the coupling reaction. Performing the reaction of **5** and **2a** in the presence of I_2_ resulted in coupling product **3ad** in 84% yield, whereas only 9% yield of **3ad** and 69% recovery of **1d** were obtained without I_2_ (Scheme 2b). Interestingly, the reaction could also proceed in the absence of DMSO even at room temperature (Scheme 2c), which suggests that coordination of different reaction paths gives rise to the I_2_-catalyzed oxidative cross-coupling reaction of α-amino ketones with nucleophiles. We further verified whether the key intermediate could be obtained when air was used as the sole oxidant. The I_2_-catalyzed hydroxylation of **1d** was successful under air at room temperature for 48 h to afford **5** in 55% yield. The addition of ^18^O-labeled water to the model reaction did not result in ^18^O-incorporated product. This result demonstrated that O_2_ from air was utilized as the sole oxygen donor in the I_2_-catalyzed hydroxylation of α-amino ketones. No product was observed when NIS instead of I_2_ was used as a typical iodization reagent for carbonyl compounds. Hence, we exclude that the hydroxylated product **5** is not formed from α-iodization of **1d** (Scheme 2d). The coupling reaction of **1d** and **2a** could be investigated under a nitrogen atmosphere, leading to an obviously reduced reaction rate (Scheme 2e, eq 1). Similarly, traces of **5** were obtained when I_2_-catalyzed hydroxylation of **1d** was performed under a nitrogen atmosphere (Scheme 2e, eq 2). Undoubtedly, air plays a vital role in the current reaction to obtain title products efficiently. The reactions of **1d** with **2a** employing 2,2,6,6-tetramethyl-1-piper-idinyloxy (TEMPO) and BHT (2,6-*tert*-butyl-*p*-cresol) as radical scavengers under the standard reaction conditions were investigated, giving the desired products in sharply reduced yields (Scheme 2f, eq 3).

When 2.0 equiv of TEMPO was added to the C–H hydroxylation reaction, the yield of **3d** decreased significantly to 0% (Scheme 2f, eq 4). These results indicated the possibility of a radical pathway.

Definitely, the mechanism is not completely clear yet. On the basis of our preliminary results and previous studies,^17^ a plausible mechanism involving I_2_-catalyzed synergistic C–H hydroxylation promoted by a combination of DMSO and air was proposed in Scheme 2g. Initially, electrophilic iodization of the substrate by I_2_ occurs to afford α-I carbonyl **A** and HI. The subsequent S_N_2 reaction with the nucleophilic oxygen atom of the DMSO generates intermediate **B**, which undergoes protonation with HI to give the hydroxylated product **5** along with the release of dimethyl sulfide and regeneration of I_2_ (path a).^17a^ Meanwhile, the C–H hydroxylation could be realized via a radical pathway. The reaction started with the oxidization of α-amino ketone **1d** by I_2_ to generate radical cation **C** and iodine radical.^18^ Then, the elimination of hydrogen iodide leads to α-amino ketone radical intermediate **D** that was immediately trapped by O_2_ and iodine radical to produce iodine peroxide **E**. Subsequently, proton exchange of E with HI results in the regeneration of I_2_ and the formation of an α-amino ketone hydroperoxide **F**,^19^ which would conduct reduction reaction with **1d** to furnish the expected product **5** (path b).^17b^ Finally, the attack of nucleophiles on the α-carbonyl imine **D** formed by I_2_-mediated dehydration of **5** provides the coupling product.

**Scheme 3:**
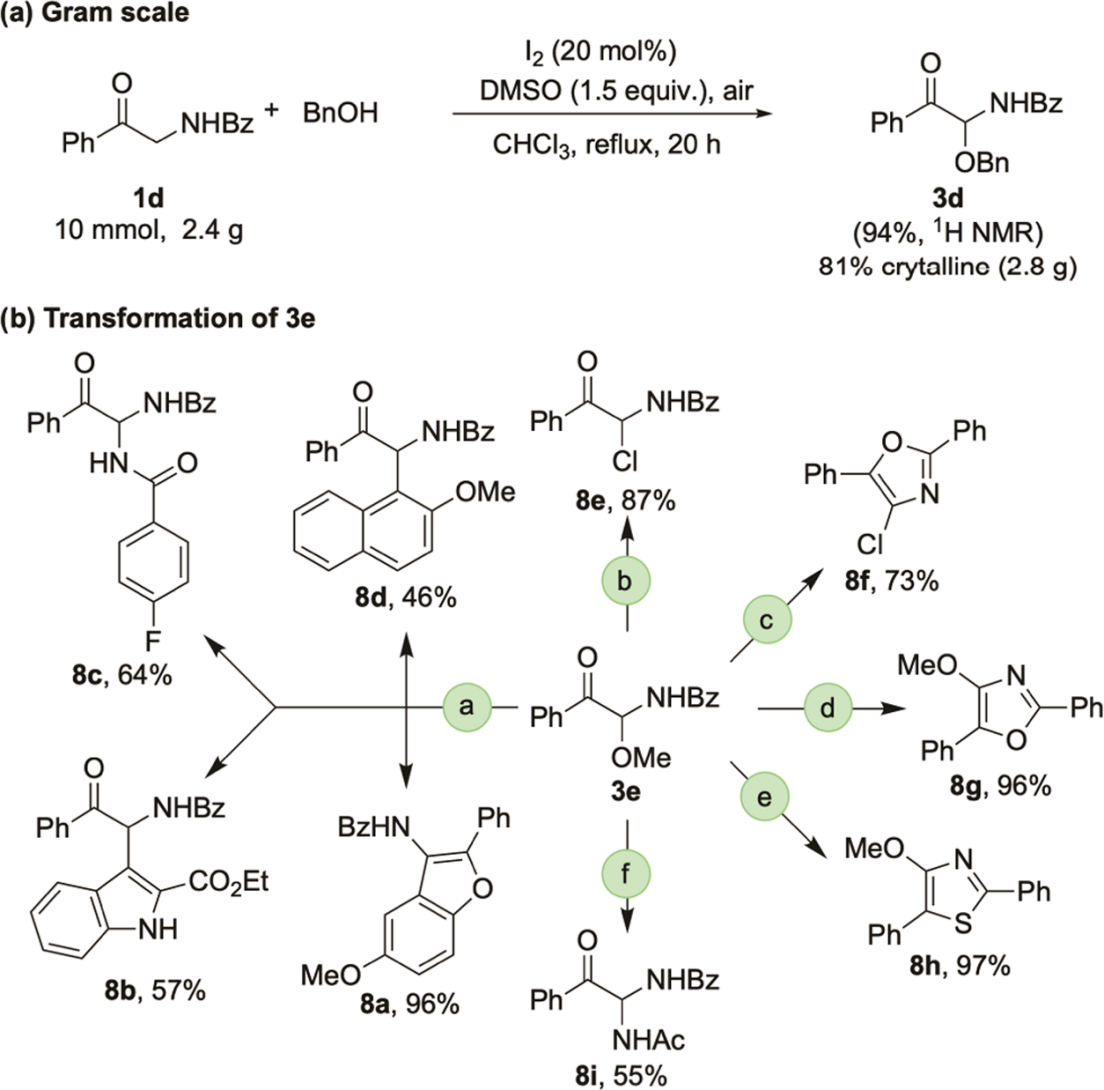
Synthetic Applications Transformation conditions: (a) NuH (1.1 equiv), MsOH (10 mol %), CHCl_3_, 80 °C, 12 h; (b) POCl_3_, r.t, 12 h; (c) POCl_3_, 80 °C, 10 h; (d) PPh_3_ (3.0 equiv), I_2_ (3.0 equiv), Et_3_N (6.0 equiv), CH_2_Cl_2_, r.t, 12 h; (e) Lawesson’s reagent (2.0 equiv), PhMe, 80 °C, 8 h; (f) MsOH (5.0 equiv), MeCN, 50 °C, 2 h.

To evaluate the practicability of this method, the reaction of **1d** (10 mmol) with **2a** was performed in gram scale, and the desired **3d** was obtained in 94% NMR yield and 80% isolated yield (2.8 g) (Scheme 3a). α-Carbonyl *N,O*-acetals constitute a versatile platform for further chemical transformations (Scheme 3b). As the methoxy group was a good leaving group, **3e** could react with various nucleophiles by using 10 mol % methanesulfonic acid (MsOH) as a catalyst to give the corresponding functionalized products **8a**–**d** in 46–96% yields. The reaction of **3e** with POCl_3_ at room temperature gave chlorination product **8e**, which further underwent Robinson–Gabriel reaction^20^ at 80 °C to give trisubstituted oxazole **8f**. When **3e** was treated with PPh_3_ and Et_3_N in dichloromethane at room temperature for 12 h,^21^ the direct Robinson–Gabriel product **8g** was isolated in 96% yield. The cyclization of **3e** with Lawesson’s reagent was readily achieved to afford thiazole **8h** in 97% yield. Finally, **3e** was transformed to α-carbonyl *N,N*-acetal **8i** in 55% yield by Ritter reaction with acetonitrile.^22^

## Conclusions

In conclusion, a simple, efficient, and metal-free version of an I_2_-catalyzed oxidative coupling between α-amino ketones and various nucleophiles has been realized for the direct C–H functionalization. Both stoichiometric DMSO and air serve as the oxidants, making this reaction environmentally friendly and easy to handle. When alcohols were used as the nucleophiles, a broad range of α-carbonyl *N,O*-acetals were prepared with high functional group tolerance in moderate to high yields. The demonstrated utilization of this protocol in the modification of biorelevant alcohols makes it particularly promising as a versatile biomolecular modification strategy. More importantly, we believe that the application of the strategy to other nucleophiles renders it highly attractive for diverse modification of α-amino carbonyl compounds.

## Experimental Section

### General Information

Column chromatography was carried out on silica gel (200–300 mesh) purchased from Shanghai Xian-Ding. Unless noted, all commercially available reagents were used without further purification. Anhydrous chloroform (CHCl_3_) was dried and degassed at reflux over CaH in a 500 mL round-bottom flask for 3 h under argon atmosphere, distilled, then stored under an argon atmosphere, and used directly. Dimethyl sulfoxide (DMSO, with molecular sieve) and I_2_ were purchased from Shanghai Xian-Ding and used as received. Other solvents for screening of conditions were dried following standard methods. ^1^H and ^13^C NMR spectra were recorded at room temperature in CDCl_3_ or *d*^6^-DMSO on a Bruker Ascend 400 or 600 spectrometer. ^1^H NMR spectra were recorded with tetramethylsilane (δ = 0.00 ppm) or solvent residual peak (CDCl_3_: 7.26 ppm; *d*^6^-DMSO: 2.50 ppm) as internal reference; ^13^C NMR spectra were recorded with CDCl_3_ (77.00 ppm) or *d*^6^-DMSO (39.52 ppm) as internal reference. Multiplicities are given as s (singlet), d (doublet), t (triplet), dd (doublet of doublets), q (quartet), or m (multiplet). High-resolution mass spectra were obtained by using the ultrahigh-performance liquid chromatograph coupled with an Agilent 6545 iFunnel accurate mass quadrupole time-of-flight mass spectrometer. The melting points of compounds were measured by an MPA100 Optimelt point apparatus with a USB port and MeltView software.

### General Procedures for Oxidative Cross-Coupling of α-Amino Ketones with Nucleophiles

A screw-capped vial was charged with α-amino ketone (0.2 mmol, 1.0 equiv), nucleophile (unless otherwise noted, 0.24 mol, 1.2 equiv), I_2_ (10.2 mg, 0.04 mmol, 20 mol %), and DMSO (21.3 μL, 0.3 mmol, 1.5 equiv), followed by the addition of anhydrous CHCl_3_ (1 mL). The vial was tightly capped and stirred at 80 °C for 15 h in an oil bath. When the reaction was completed, the crude reaction mixture was allowed to reach room temperature. The solution was then quenched with 10% Na_2_S_2_O_3_ (2 mL) solution (w/w) and extracted with CH_2_Cl_2_ (2 × 4 mL). The combined organic layers were dried over Na_2_SO_4_, filtered, and concentrated in vacuo. The crude product was purified by column chromatography on silica gel to afford the product **3** or **4**.

#### *N*-(1-(Benzyloxy)-2-oxo-2-phenylethyl)benzamide (**3d**)

The general procedure was performed using *N*-(2-oxo-2-phenylethyl)benzamide **1d** (47.8 mg, 0.2 mmol) and benzyl alcohol **2a** (26.0 mg, 24.8 μL, 0.24 mmol). Purification by chromatography (petroleum ether/EtOAc = 4:1) afforded compound **3d** (66.2 mg, 96% yield) as a white solid. *R_f_* = 0.32 (petroleum ether/EtOAc = 4:1). Mp 117.2–118.8 °C. ^1^H NMR (400 MHz, Chloroform-*d*) δ 7.92 (t, *J* = 9.3 Hz, 4H), 7.73 (d, *J* = 8.8 Hz, 1H), 7.63–7.53 (m, 2H), 7.52–7.37 (m, 6H), 7.35–7.28 (m, 3H), 6.73 (d, *J* = 8.8 Hz, 1H), 4.87 (dd, *J* = 24.0, 11.7 Hz, 2H); ^13^C{^1^H} NMR (151 MHz, Chloroform-*d*) δ 192.1, 168.0, 137.1, 134.2, 133.7, 133.6, 132.3, 129.5, 128.7, 128.7, 128.5, 128.4, 128.0, 127.3, 76.7, 70.8. HRMS (ESI) *m*/*z*: [M + Na]^+^ calcd for C_22_H_19_NNaO_3_ 368.1263; found 368.1246.

#### *N*-(1-Methoxy-2-oxo-2-phenylethyl)benzamide (**3e**)

The general procedure was performed using *N*-(2-oxo-2-phenylethyl)benzamide **1d** (47.8 mg, 0.2 mmol) and methanol **2b** (64.1 mg, 81.0 μL, 2.0 mmol). Purification by chromatography (petroleum ether/EtOAc = 4:1) afforded compound **3e** (49.8 mg, 93% yield) as a white solid. *R_f_* = 0.48 (petroleum ether/EtOAc = 3:1). Mp 114.6–116.9 °C. ^1^H NMR (600 MHz, Chloroform-*d*) δ 8.12–8.10 (m, 2H), 7.93–7.91 (m, 2H), 7.66–7.61 (m, 2H), 7.59–7.47 (m, 5H), 6.56 (d, *J* = 8.7 Hz, 1H), 3.57 (s, 3H); ^13^C{^1^H} NMR (151 MHz, Chloroform-*d*) δ 192.3, 167.9, 134.4, 133.6, 133.4, 132.3, 129.4, 128.8, 128.7, 127.3, 78.4, 55.8. HRMS (ESI) *m*/*z*: [M + Na]^+^ calcd for C_16_H_15_NNaO_3_ 292.0950; found 292.0938.

#### *N*-(1-Ethoxy-2-oxo-2-phenylethyl)benzamide (**3f**)

The general procedure was performed using *N*-(2-oxo-2-phenylethyl)benzamide **1d** (47.8 mg, 0.2 mmol) and ethanol **2c** (92.1 mg, 116.8 μL, 2.0 mmol). Purification by chromatography (petroleum ether/EtOAc = 4:1) afforded compound **3f** (52.0 mg, 92% yield) as a white solid. *R_f_* = 0.55 (petroleum ether/EtOAc = 3:1). Mp 123.0–124.4 °C. ^1^H NMR (600 MHz, Chloroform-*d*) δ 8.11 (d, *J* = 7.4 Hz, 2H), 7.91 (d, *J* = 7.3 Hz, 2H), 7.67–7.62 (m, 2H), 7.59–7.45 (m, 5H), 6.63 (d, *J* = 8.7 Hz, 1H), 3.93–3.85 (m, 2H), 1.25 (t, *J* = 7.0 Hz, 3H); ^13^C{^1^H} NMR (151 MHz, Chloroform-*d*) δ 192.4, 167.8, 134.3, 133.7, 133.5, 132.2, 129.5, 128.8, 128.7, 127.3, 77.3, 64.6, 15.2. HRMS (ESI) *m*/*z*: [M + Na]^+^ calcd for C_17_H_17_NNaO_3_ 306.1106; found 306.1108.

#### *N*-(1-(Octyloxy)-2-oxo-2-phenylethyl)benzamide (**3g**)

The general procedure was performed using *N*-(2-oxo-2-phenylethyl)benzamide **1d** (47.8 mg, 0.2 mmol) and 1-octanol **2d** (31.3 mg, 37.7 μL, 0.24 mmol). Purification by chromatography (petroleum ether/EtOAc = 6:1) afforded compound **3g** (63.9 mg, 87% yield) as a white solid. *R_f_* = 0.48 (petroleum ether/EtOAc = 5:1). Mp 73.5–76.5 °C. ^1^H NMR (600 MHz, Chloroform-*d*) δ 8.13–8.08 (m, 2H), 7.94–7.89 (m, 2H), 7.67–7.61 (m, 2H), 7.59–7.46 (m, 5H), 6.61 (d, *J* = 8.7 Hz, 1H), 3.85–3.75 (m, 2H), 1.63–1.54 (m, 2H), 1.33–1.13 (m, 10H), 0.85 (t, *J* = 7.1 Hz, 3H); ^13^C{^1^H} NMR (151 MHz, Chloroform-*d*) δ 192.4, 167.8, 134.2, 133.7, 133.5, 132.2, 129.5, 128.7, 128.7, 127.3, 77.6, 69.0, 31.7, 29.6, 29.2, 29.2, 26.1, 22.6, 14.1. HRMS (ESI) *m*/*z*: [M + Na]^+^ calcd for C_23_H_29_NNaO_3_ 390.2045; found 390.2031.

#### *N*-(1-Isopropoxy-2-oxo-2-phenylethyl)benzamide (**3h**)

The general procedure was performed using *N*-(2-oxo-2-phenylethyl)benzamide **1d** (47.8 mg, 0.2 mmol) and isopropyl alcohol **2e** (16.8 mg, 21.4 μL, 0.24 mmol). Purification by chromatography (petroleum ether/EtOAc = 5:1) afforded compound **3h** (50.1 mg, 84% yield) as a white solid. *R_f_* = 0.63 (petroleum ether/EtOAc = 3:1). Mp 94.9–95.4 °C. ^1^H NMR (600 MHz, Chloroform-*d*) δ 8.11 (dd, *J* = 8.4, 1.4 Hz, 2H), 7.92–7.89 (m, 2H), 7.63 (t, *J* = 7.4 Hz, 1H), 7.60–7.54 (m, 2H), 7.53–7.47 (m, 4H), 6.73 (d, *J* = 9.0 Hz, 1H), 4.22–4.16 (m, 1H), 1.41 (d, *J* = 6.0 Hz, 3H), 1.18 (d, *J* = 6.2 Hz, 3H); ^13^C{^1^H} NMR (151 MHz, Chloroform-*d*) δ 192.6, 167.7, 134.1, 133.8, 133.5, 132.2, 129.5, 128.7, 127.3, 75.3, 70.7, 23.4, 21.7. HRMS (ESI) *m*/*z*: [M + Na]^+^ calcd for C_18_H_19_NNaO_3_ 320.1263; found 320.1260.

#### *N*-(1-(Cyclohexyloxy)-2-oxo-2-phenylethyl)benzamide (**3i**)

The general procedure was performed using *N*-(2-oxo-2-phenylethyl)benzamide **1d** (47.8 mg, 0.2 mmol) and cyclohexanol **2f** (24.0 mg, 25.4 μL, 0.24 mmol). Purification by chromatography (petroleum ether/EtOAc = 6:1) afforded compound **3i** (60.2 mg, 89% yield) as a white solid. *R_f_* = 0.40 (petroleum ether/EtOAc = 4:1). Mp 132.7–134.9 °C. ^1^H NMR (600 MHz, Chloroform-*d*) δ 8.12 (dd, *J* = 8.4, 1.4 Hz, 2H), 7.90 (dd, *J* = 8.3, 1.3 Hz, 2H), 7.63 (t, *J* = 7.4 Hz, 1H), 7.58–7.55 (m, 2H), 7.53–7.48 (m, 4H), 6.77 (d, *J* = 9.0 Hz, 1H), 3.88–3.84 (m, 1H), 2.28 (d, *J* = 8.9 Hz, 1H), 1.83–1.76 (m, 2H), 1.69–1.65 (m, 1H), 1.55–1.51 (m, 1H), 1.48–1.42 (m, 1H), 1.37–1.22 (m, 3H), 1.20–1.13 (m, 1H); ^13^C{^1^H} NMR (151 MHz, Chloroform-*d*) δ 192.7, 167.7, 134.1, 133.9, 133.6, 132.2, 129.5, 128.7, 128.7, 127.3, 76.6, 75.1, 33.6, 31.9, 25.6, 24.3, 24.2. HRMS (ESI) *m*/*z*: [M + Na]^+^ calcd for C_21_H_23_NNaO_3_ 360.1576; found 360.1579.

#### *N*-(2-Oxo-2-phenyl-1-(1-phenylethoxy)ethyl)benzamide (**3j**)

The general procedure was performed using *N*-(2-oxo-2-phenylethyl)benzamide **1d** (47.8 mg, 0.2 mmol) and *dl*-1-phenethylalcohol **2g** (29.3 mg, 29.0 μL, 0.24 mmol) to afford the crude diastereomers at a ratio of 1.83:1 (indicated by ^1^H NMR). Purification by chromatography (petroleum ether/EtOAc = 6:1) afforded major isomer (27.2 mg, 38% yield) as a white solid and minor isomer (16.8 mg, 23% yield) as a white solid. Major isomer: *R_f_* = 0.37 (petroleum ether/EtOAc = 6:1). Mp 110.1–112.3 °C. ^1^H NMR (600 MHz, Chloroform-*d*) δ 7.90 (d, *J* = 7.2 Hz, 2H), 7.64 (d, *J* = 9.1 Hz, 1H), 7.60–7.55 (m, 3H), 7.55–7.49 (m, 5H), 7.44–7.37 (m, 3H), 7.29 (t, *J* = 7.8 Hz, 2H), 6.52 (d, *J* = 9.1 Hz, 1H), 5.00 (q, *J* = 6.6 Hz, 1H), 1.47 (d, *J* = 6.7 Hz, 3H); ^13^C{^1^H} NMR (151 MHz, Chloroform-*d*) δ 192.4, 167.9, 141.6, 134.0, 133.6, 133.3, 132.2, 129.5, 128.7, 128.5, 128.5, 128.4, 127.9, 127.3, 76.2, 74.7, 23.3. HRMS (ESI) *m*/*z*: [M + Na]^+^ calcd for C_23_H_21_NNaO_3_ 382.1419; found 382.1400. Minor isomer: *R_f_* = 0.28 (petroleum ether/EtOAc = 6:1). Mp 93.5–95.7 °C. ^1^H NMR (600 MHz, Chloroform-*d*) δ 8.13 (d, *J* = 7.2 Hz, 2H), 7.64 (t, *J* = 7.4 Hz, 1H), 7.56 (d, *J* = 7.2 Hz, 2H), 7.53 (t, *J* = 7.8 Hz, 2H), 7.49 (t, *J* = 7.4 Hz, 1H), 7.38 (t, *J* = 7.8 Hz, 2H), 7.29–7.25 (m, 3H), 7.20 (t, *J* = 7.6 Hz, 2H), 7.14 (t, *J* = 7.3 Hz, 1H), 6.87 (d, *J* = 9.2 Hz, 1H), 5.04 (q, *J* = 6.5 Hz, 1H), 1.64 (d, *J* = 6.4 Hz, 3H); ^13^C{^1^H} NMR (151 MHz, Chloroform-*d*) δ 192.1, 167.7, 143.9, 134.2, 133.9, 133.6, 132.0, 129.5, 128.7, 128.5, 128.2, 127.3, 127.1, 125.8, 77.0, 76.4. HRMS (ESI) *m*/*z*: [M + Na]^+^ calcd for C_23_H_21_NNaO_3_ 382.1419; found 382.1411.

#### *N*-(1-(Benzhydryloxy)-2-oxo-2-phenylethyl)benzamide (**3k**)

The general procedure was performed using *N*-(2-oxo-2-phenylethyl)benzamide **1d** (47.8 mg, 0.2 mmol) and benzhydrol **2h** (44.2 mg, 0.24 mmol). Purification by chromatography (petroleum ether/EtOAc = 6:1) afforded compound **3k** (31.6 mg, 38% yield) as a white solid. *R_f_* = 0.35 (petroleum ether/EtOAc = 6:1). Mp 87.7–89.5 °C. ^1^H NMR (400 MHz, Chloroform-*d*) δ 7.80 (d, *J* = 7.6 Hz, 2H), 7.68 (d, *J* = 7.4 Hz, 3H), 7.55–7.51 (m, 4H), 7.46–7.37 (m, 5H), 7.37–7.27 (m, 4H), 7.25–7.20 (m, 2H), 7.18–7.12 (m, 1H), 6.78 (d, *J* = 9.3 Hz, 1H), 6.01 (s, 1H); ^13^C{^1^H} NMR (101 MHz, Chloroform-*d*) δ 192.0, 168.1, 141.9, 140.6, 134.1, 133.5, 133.4, 132.2, 129.6, 129.1, 128.6, 128.5, 128.3, 128.1, 127.3, 127.2, 126.5, 81.1, 75.2. HRMS (ESI) *m*/*z*: [M + Na]^+^ calcd for C_28_H_23_NNaO_3_ 444.1576; found 444.1570.

#### *N*-(1-(*tert*-Butoxy)-2-oxo-2-phenylethyl)benzamide (**3l**)

The general procedure was performed using *N*-(2-oxo-2-phenylethyl)benzamide **1d** (47.8 mg, 0.2 mmol) and *tert*-butanol **2i** (17.8 mg, 23.0 μL, 0.24 mmol). Purification by chromatography (petroleum ether/EtOAc = 5:1) afforded compound **3l** (16.8 mg, 27% yield) as a white solid. *R_f_* = 0.46 (petroleum ether/EtOAc = 4:1). Mp 185.5–187.6 °C. ^1^H NMR (600 MHz, Chloroform-*d*) δ 8.09 (dd, *J* = 8.4, 1.4 Hz, 2H), 7.86–7.81 (m, 2H), 7.61 (t, *J* = 7.4 Hz, 1H), 7.57–7.48 (m, 3H), 7.46 (t, *J* = 7.7 Hz, 2H), 6.86 (d, *J* = 9.6 Hz, 1H), 1.37 (s, 9H); ^13^C{^1^H} NMR (151 MHz, Chloroform-*d*) δ 193.8, 166.7, 134.1, 133.8, 132.1, 129.1, 128.7, 128.7, 127.2, 77.1, 72.4, 28.6. HRMS (ESI) *m*/*z*: [M + Na]^+^ calcd for C_19_H_21_NNaO_3_ 334.1419; found 334.1412.

#### *N*-(2-Oxo-2-phenyl-1-(2,2,2-trifluoroethoxy)ethyl)benzamide (**3m**)

The general procedure was performed using *N*-(2-oxo-2-phenylethyl)benzamide **1d** (47.8 mg, 0.2 mmol) and 2-azidoethan-1-ol **2j** (200.1 mg, 141.4 μL, 2.0 mmol, 10 equiv). Purification by chromatography (petroleum ether/EtOAc = 6:1) afforded compound **3m** (40.7 mg, 60% yield) as a white solid. *R_f_* = 0.35 (petroleum ether/EtOAc = 4:1). Mp 197.6–199.7 °C. ^1^H NMR (600 MHz, Chloroform-*d*) δ 8.10 (d, *J* = 7.1 Hz, 2H), 7.92 (d, *J* = 7.1 Hz, 2H), 7.84 (d, *J* = 8.6 Hz, 1H), 7.67 (t, *J* = 7.4 Hz, 1H), 7.59 (t, *J* = 7.4 Hz, 1H), 7.55 (t, *J* = 7.8 Hz, 2H), 7.51 (t, *J* = 7.8 Hz, 2H), 6.71 (d, *J* = 8.6 Hz, 1H), 4.38–4.31 (m, 1H), 4.22–4.15 (m, 1H); ^13^C{^1^H} NMR (151 MHz, Chloroform-*d*) δ 190.5, 168.2, 134.8, 133.1, 132.7, 132.7, 129.6, 128.9, 128.9, 127.4, 123.5 (q, *J*_*C–F*_ = 278.3 Hz), 77.74, 65.91 (q, *J*_*C–F*_ = 35.1 Hz); ^19^F NMR (376 MHz, Chloroform-*d*) δ −73.98. HRMS (ESI) *m*/*z*: [M + Na]^+^ calcd for C_17_H_14_F_3_NNaO_3_ 360.0823; found 360.0816.

#### *N*-(1-(2-Bromoethoxy)-2-oxo-2-phenylethyl)benzamide (**3n**)

The general procedure was performed using *N*-(2-oxo-2-phenylethyl)benzamide **1d** (47.8 mg, 0.2 mmol) and 2-bromoethanol **2k** (30.0 mg, 17.0 μL, 0.24 mmol). Purification by chromatography (petroleum ether/EtOAc = 3:1) afforded compound **3n** (54.8 mg, 76% yield) as a white solid. *R_f_* = 0.39 (petroleum ether/EtOAc = 3:1). Mp 122.3–123.6 °C. ^1^H NMR (600 MHz, Chloroform-*d*) δ 8.15 (dd, *J* = 8.4, 1.3 Hz, 2H), 7.93–7.90 (m, 2H), 7.73 (d, *J* = 8.6 Hz, 1H), 7.68–7.64 (m, 1H), 7.60–7.56 (m, 1H), 7.54 (t, *J* = 7.8 Hz, 2H), 7.50 (t, *J* = 7.7 Hz, 2H), 6.65 (d, *J* = 8.6 Hz, 1H), 4.23–4.12 (m, 2H), 3.59–3.44 (m, 2H); ^13^C{^1^H} NMR (151 MHz, Chloroform-*d*) δ 191.6, 167.9, 134.5, 133.4, 133.2, 132.4, 129.7, 128.8, 128.8, 127.3, 77.3, 68.7, 30.1. HRMS (ESI) *m*/*z*: [M + Na]^+^ calcd for C_17_H_16_BrNNaO_3_ 384.0211; found 384.0208.

#### *N*-(13-Oxo-13-phenyl-2,5,8,11-tetraoxatridecan-12-yl)benzamide (**3o**)

The general procedure was performed using *N*-(2-oxo-2-phenylethyl)benzamide **1d** (47.8 mg, 0.2 mmol) and methoxytriethylene glycol **2l** (39.4 mg, 38.4 μL, 0.24 mmol). Purification by chromatography (petroleum ether/EtOAc = 1:2–0:1) afforded compound **3o** (65.3 mg, 81% yield) as a colorless oil. *R_f_* = 0.46 (EtOAc). ^1^H NMR (400 MHz, Chloroform-*d*) δ 8.16 (d, *J* = 7.8 Hz, 2H), 7.92 (d, *J* = 7.6 Hz, 2H), 7.76 (d, *J* = 8.8 Hz, 1H), 7.57–7.43 (m, 5H), 6.65 (d, *J* = 8.8 Hz, 1H), 4.07–3.92 (m, 2H), 3.76–3.58 (m, 9H), 3.50–3.48 (m, 2H), 3.33 (s, 3H); ^13^C{^1^H} NMR (101 MHz, Chloroform-*d*) δ 192.1, 167.8, 134.2, 133.7, 133.4, 132.2, 129.6, 128.7, 128.7, 127.3, 77.9, 71.8, 70.5, 70.5, 70.5, 70.0, 68.2, 58.9. HRMS (ESI) *m*/*z*: [M + H]^+^ calcd for C_22_H_28_NO_6_ 402.1917; found 402.1932.

#### *N*-(2-Oxo-2-phenyl-1-(2-(trimethylsilyl)ethoxy)ethyl)benzamide (**3p**)

The general procedure was performed using *N*-(2-oxo-2-phenylethyl)benzamide **1d** (47.8 mg, 0.2 mmol) and 2-(trimethylsilyl)ethanol **2m** (28.4 mg, 34.4 μL, 0.24 mmol). Purification by chromatography (petroleum ether/EtOAc = 6:1) afforded compound **3p** (47.4 mg, 67% yield) as a pale yellow solid. *R_f_* = 0.44 (petroleum ether/EtOAc = 4:1). Mp 128.3–129.8 °C. ^1^H NMR (600 MHz, Chloroform-*d*) δ 8.10 (d, *J* = 7.2 Hz, 2H), 7.90 (d, *J* = 7.1 Hz, 2H), 7.63 (t, *J* = 7.4 Hz, 1H), 7.58 (d, *J* = 8.9 Hz, 1H), 7.56 (t, *J* = 7.4 Hz, 1H), 7.52 (t, *J* = 7.8 Hz, 2H), 7.49 (t, *J* = 7.6 Hz, 2H), 6.63 (d, *J* = 8.8 Hz, 1H), 3.91 (t, *J* = 8.2 Hz, 2H), 1.10–1.05 (m, 1H), 0.95–0.85 (m, 1H), −0.03 (s, 9H); ^13^C{^1^H} NMR (151 MHz, Chloroform-*d*) δ 192.5, 167.8, 134.2, 133.8, 133.6, 132.2, 129.4, 128.8, 128.7, 127.3, 76.9, 66.4, 18.3, −1.5. HRMS (ESI) *m*/*z*: [M + Na]^+^ calcd for C_20_H_25_NNaO_3_Si 378.1501; found 378.1511.

#### *N*-(1-(2-Nitroethoxy)-2-oxo-2-phenylethyl)benzamide (**3q**)

The general procedure was performed using *N*-(2-oxo-2-phenylethyl)benzamide **1d** (47.8 mg, 0.2 mmol) and 2-nitroethanol **2n** (21.9 mg, 17.2 μL, 0.24 mmol). Purification by chromatography (petroleum ether/EtOAc = 2:1) afforded compound **3q** (30.6 mg, 47% yield) as a white solid. *R_f_* = 0.28 (petroleum ether/EtOAc = 2:1). Mp 105.3–107.2 °C. ^1^H NMR (600 MHz, Chloroform-*d*) δ 8.05 (d, *J* = 7.0 Hz, 2H), 7.92 (d, *J* = 7.0 Hz, 2H), 7.77 (d, *J* = 8.7 Hz, 1H), 7.65 (t, *J* = 7.4 Hz, 1H), 7.59 (t, *J* = 7.4 Hz, 1H), 7.55–7.49 (m, 4H), 6.65 (d, *J* = 8.6 Hz, 1H), 4.61–4.57 (m, 1H), 4.53–4.47 (m, 2H), 4.35–4.31 (m, 1H); ^13^C{^1^H} NMR (151 MHz, Chloroform-*d*) δ 191.0, 168.2, 134.7, 133.2, 133.0, 132.6, 129.5, 128.9, 128.8, 127.3, 77.3, 74.6, 64.2. HRMS (ESI) *m*/*z*: [M + Na]^+^ calcd for C_17_H_16_N_2_NaO_5_ 351.0957; found 351.0973.

#### *N*-(1-(But-3-en-1-yloxy)-2-oxo-2-phenylethyl)benzamide (**3r**)

The general procedure was performed using *N*-(2-oxo-2-phenylethyl)benzamide **1d** (47.8 mg, 0.2 mmol) and 3-buten-1-ol **2o** (17.3 mg, 20.7 μL, 0.24 mmol). Purification by chromatography (petroleum ether/EtOAc = 4:1) afforded compound **3r** (58.7 mg, 95% yield) as a white solid. *R_f_* = 0.33 (petroleum ether/EtOAc = 4:1). Mp 88.5–91.1 °C. ^1^H NMR (600 MHz, Chloroform-*d*) δ 8.11 (d, *J* = 7.4 Hz, 2H), 7.91 (d, *J* = 7.2 Hz, 2H), 7.65–7.63 (m, 2H), 7.58–7.55 (m, 1H), 7.54–7.48 (m, 4H), 6.62 (d, *J* = 8.6 Hz, 1H), 5.80–5.73 (m, 1H), 5.06 (dd, *J* = 17.2, 1.6 Hz, 1H), 5.00 (d, *J* = 10.2 Hz, 1H), 3.91–3.84 (m, 2H), 2.38–2.35 (m, 2H); ^13^C{^1^H} NMR (151 MHz, Chloroform-*d*) δ 192.2, 167.8, 134.8, 134.3, 133.7, 133.5, 132.3, 129.6, 128.8, 128.7, 127.3, 116.8, 77.5, 68.3, 34.0. HRMS (ESI) *m*/*z*: [M + Na]^+^ calcd for C_19_H_19_NNaO_3_ 332.1263; found 332.1278.

#### *N*-(1-(But-3-yn-1-yloxy)-2-oxo-2-phenylethyl)benzamide (**3s**)

The general procedure was performed using *N*-(2-oxo-2-phenylethyl)benzamide **1d** (47.8 mg, 0.2 mmol) and 3-butyn-1-ol **2p** (16.8 mg, 18.2 μL, 0.24 mmol). Purification by chromatography (petroleum ether/EtOAc = 4:1) afforded compound **3s** (36.7 mg, 60% yield) as a white solid. *R_f_* = 0.42 (petroleum ether/EtOAc = 4:1). Mp 117.5–119.2 °C. ^1^H NMR (400 MHz, Chloroform-*d*) δ 8.15 (d, *J* = 7.8 Hz, 2H), 7.91 (d, *J* = 7.7 Hz, 2H), 7.70–7.63 (m, 2H), 7.59–7.48 (m, 5H), 6.64 (d, *J* = 8.8 Hz, 1H), 4.02–3,91 (m, 2H), 2.52 (d, *J* = 6.6 Hz, 2H), 1.94 (d, *J* = 3.1 Hz, 2H); ^13^C{^1^H} NMR (101 MHz, Chloroform-*d*) δ 191.9, 167.9, 134.4, 133.6, 133.3, 132.3, 129.7, 128.8, 127.3, 81.0, 77.5, 69.6, 67.0, 19.8. HRMS (ESI) *m*/*z*: [M + Na]^+^ calcd for C_19_H_17_NNaO_3_ 330.1106; found 330.1109.

#### Benzyl 2-(1-Benzamido-2-oxo-2-phenylethoxy)acetate (**3t**)

The general procedure was performed using *N*-(2-oxo-2-phenylethyl)benzamide **1d** (47.8 mg, 0.2 mmol) and benzyl 2-hydroxyacetate **2q** (40.0 mg, 0.24 mmol). Purification by chromatography (petroleum ether/EtOAc = 4:1) afforded compound **3t** (29.3 mg, 36% yield) as a white solid. *R_f_* = 0.42 (petroleum ether/EtOAc = 4:1). Mp 149.1–152.0 °C. ^1^H NMR (400 MHz, Chloroform-*d*) δ 8.34 (d, *J* = 7.7 Hz, 2H), 7.90 (d, *J* = 7.5 Hz, 2H), 7.74 (d, *J* = 8.8 Hz, 1H), 7.65–7.55 (m, 2H), 7.49 (t, *J* = 7.6 Hz, 4H), 7.41–7.32 (m, 5H), 6.88 (d, *J* = 8.8 Hz, 1H), 5.32–5.20 (m, 2H), 4.57–4.30 (m, 2H).; ^13^C{^1^H} NMR (151 MHz, Chloroform-*d*) δ 191.5, 169.8, 168.3, 135.3, 134.5, 133.4, 133.0, 132.4, 130.1, 128.77, 128.6, 128.5, 128.4, 127.4, 66.9, 64.7. HRMS (ESI) *m*/*z*: [M + Na]^+^ calcd for C_24_H_21_NNaO_5_ 426.1317; found 426.1311.

#### 2-(1-Benzamido-2-oxo-2-phenylethoxy)ethyl 4-methylbenzenesulfonate (**3u**)

The general procedure was performed using *N*-(2-oxo-2-phenylethyl)benzamide **1d** (47.8 mg, 0.2 mmol) and 2-hydroxyethyl 4-methylbenzenesulfonate **2r** (51.8 mg, 0.24 mmol). Purification by chromatography (petroleum ether/EtOAc = 2:1) afforded compound **3u** (68.8 mg, 76% yield) as a white solid. *R_f_* = 0.30 (petroleum ether/EtOAc = 2:1). Mp 88.6–90.2 °C. ^1^H NMR (600 MHz, Chloroform-*d*) δ 8.09 (d, *J* = 7.8 Hz, 2H), 7.87 (d, *J* = 7.5 Hz, 2H), 7.67–7.64 (m, 4H), 7.58–7.53 (m, 3H), 7.51–7.48 (m, 2H), 7.20 (d, *J* = 7.8 Hz, 2H), 6.55 (d, *J* = 8.6 Hz, 1H), 4.30–4.25 (m, 1H), 4.13–4.09 (m, 2H), 4.02–3.98 (m, 1H), 2.38 (s, 3H); ^13^C{^1^H} NMR (151 MHz, Chloroform-*d*) δ 191.3, 168.0, 144.8, 134.5, 133.3, 133.1, 132.7, 132.5, 129.7, 129.7, 128.9, 128.8, 127.9, 127.3, 77.5, 68.6, 66.6, 21.6. HRMS (ESI) *m*/*z*: [M + Na]^+^ calcd for C_24_H_23_NNaO_6_S 476.1144; found 476.1134.

#### *N*-(2-Oxo-2-phenyl-1-(2-(4-phenylbutanamido)ethoxy)ethyl)benzamide (**3v**)

The general procedure was performed using *N*-(2-oxo-2-phenylethyl)benzamide **1d** (47.8 mg, 0.2 mmol) and 2-hydroxyethyl 4-methylbenzenesulfonate **2s** (49.7 mg, 0.24 mmol). Purification by chromatography (petroleum ether/EtOAc = 1:2) afforded compound **3v** (26.8 mg, 30% yield) as a white solid. *R_f_* = 0.37 (petroleum ether/EtOAc = 1:2). Mp 223.3–226.2 °C. ^1^H NMR (600 MHz, Chloroform-*d*) δ 8.09 (d, *J* = 7.3 Hz, 2H), 7.89 (d, *J* = 7.2 Hz, 2H), 7.75 (d, *J* = 8.1 Hz, 1H), 7.63 (t, *J* = 7.4 Hz, 1H), 7.57 (t, *J* = 7.4 Hz, 1H), 7.51–7.47 (m, 4H), 7.26 (t, *J* = 7.5 Hz, 2H), 7.18 (t, *J* = 7.4 Hz, 1H), 7.13 (d, *J* = 7.3 Hz, 2H), 6.62 (d, *J* = 8.1 Hz, 1H), 5.81 (s, 1H), 3.89–3.85 (m, 1H), 3.81–3.77 (m, 1H), 3.53–3.48 (m, 1H), 3.42–3.37 (m, 1H), 2.57 (t, *J* = 7.5 Hz, 2H), 2.04 (t, *J* = 7.5 Hz, 2H), 1.89–1.84 (m, 2H); ^13^C{^1^H} NMR (151 MHz, Chloroform-*d*) δ 192.0, 172.6, 167.9, 141.5, 134.6, 133.5, 133.0, 132.5, 129.4, 129.0, 128.8, 128.5, 128.4, 127.3, 125.9, 77.6, 66.9, 39.2, 35.8, 35.2, 26.9. HRMS (ESI) *m*/*z*: [M + Na]^+^ calcd for C_27_H_28_N_2_NaO_4_ 467.1947; found 467.1950.

#### *N*-(1-(2-((4-Methoxyphenyl)amino)-2-oxoethoxy)-2-oxo-2-phenylethyl)benzamide (**3w**)

The general procedure was performed using *N*-(2-oxo-2-phenylethyl)benzamide **1d** (47.8 mg, 0.2 mmol) and 2-hydroxy-*N*-(4-methoxyphenyl)acetamide **2t** (68.9 mg, 0.24 mmol). Purification by chromatography (CH_2_Cl_2_/EtOAc = 8:1) afforded compound **3w** (51.5 mg, 62% yield) as a white solid. *R_f_* = 0.35 (CH_2_Cl_2_/EtOAc = 8:1). Mp 161.2–162.7 °C. ^1^H NMR (600 MHz, Chloroform-*d*) δ 8.29 (bs, 1H), 8.19–8.14 (m, 2H), 7.91 (d, *J* = 7.5 Hz, 1H), 7.88–7.82 (m, 2H), 7.70 (t, *J* = 7.4 Hz, 1H), 7.58–7.55 (m, 3H), 7.49–7.45 (m, 2H), 7.40 (d, *J* = 9.0 Hz, 2H), 6.81 (d, *J* = 9.0 Hz, 2H), 6.77 (d, *J* = 7.6 Hz, 1H), 4.25 (dd, 36.9, 15.4 Hz, 2H), 3.77 (s, 3H); ^13^C{^1^H} NMR (151 MHz, Chloroform-*d*) δ 191.6, 168.3, 166.5, 156.5, 135.0, 133.1, 132.7, 132.6, 130.3, 129.6, 129.2, 128.8, 127.4, 121.4, 114.0, 77.7, 65.9, 55.4. HRMS (ESI) *m*/*z*: [M + H]^+^ calcd for C_24_H_23_N_2_O_5_ 419.1607; found 419.1609.

#### *N*-(2-(1-Benzamido-2-oxo-2-phenylethoxy)ethyl)benzamide (**3x**)

The general procedure was performed using *N*-(2-oxo-2-phenylethyl)benzamide **1d** (47.8 mg, 0.2 mmol) and *N*-(2-hydroxyethyl)benzamide **2u** (39.6 mg, 0.24 mmol). Purification by chromatography (petroleum ether/EtOAc = 1:1) afforded compound **3x** (57.3 mg, 71% yield) as a white solid. *R_f_* = 0.26 (petroleum ether/EtOAc = 1:1). Mp 124.5–127.1 °C. ^1^H NMR (600 MHz, Chloroform-*d*) δ 8.09 (d, *J* = 7.5 Hz, 2H), 7.87 (d, *J* = 7.4 Hz, 2H), 7.80 (d, *J* = 7.9 Hz, 1H), 7.64 (d, *J* = 7.3 Hz, 2H), 7.60 (t, *J* = 7.4 Hz, 1H), 7.56 (t, *J* = 7.4 Hz, 1H), 7.49–7.43 (m, 5H), 7.35 (t, *J* = 7.7 Hz, 2H), 6.66 (d, *J* = 8.1 Hz, 1H), 6.60 (bs, 1H), 4.02–3.92 (m, 2H), 3.78–3.73 (m, 1H), 3.61–3.56 (m, 1H); ^13^C{^1^H} NMR (151 MHz, Chloroform-*d*) δ 192.0, 168.0, 167.3, 134.5, 134.2, 133.5, 133.0, 132.4, 131.4, 129.3, 129.0, 128.8, 128.4, 127.3, 126.9, 77.7, 67.0, 39.8. HRMS (ESI) *m*/*z*: [M + Na]^+^ calcd for C_24_H_22_N_2_NaO_4_ 425.1477; found 425.1475.

#### *N*-(1-(2-Azidoethoxy)-2-oxo-2-phenylethyl)benzamide (**3y**)

The general procedure was performed using *N*-(2-oxo-2-phenylethyl)benzamide **1d** (47.8 mg, 0.2 mmol) and 2-azidoethan-1-ol **2v** (20.9 mg, 0.24 mmol). Purification by chromatography (petroleum ether/EtOAc = 4:1) afforded compound **3y** (44.3 mg, 68% yield) as a white solid. *R_f_* = 0.30 (petroleum ether/EtOAc = 4:1). Mp 93.4–94.7 °C. ^1^H NMR (600 MHz, Chloroform-*d*) δ 8.13 (d, *J* = 7.3 Hz, 2H), 7.92 (d, *J* = 7.2 Hz, 2H), 7.75 (d, *J* = 8.6 Hz, 1H), 7.66 (t, *J* = 7.4 Hz, 1H), 7.58 (t, *J* = 7.4 Hz, 1H), 7.55 (t, *J* = 7.8 Hz, 2H), 7.50 (t, *J* = 7.7 Hz, 2H), 6.65 (d, *J* = 8.6 Hz, 1H), 4.06–3.99 (m, 2H), 3.47–3.37 (m, 2H); ^13^C{^1^H} NMR (151 MHz, Chloroform-*d*) δ 191.6, 168.0, 134.5, 133.4, 133.2, 132.4, 129.5, 128.9, 128.8, 127.3, 77.5, 67.7, 50.6. HRMS (ESI) *m*/*z*: [M + Na]^+^ calcd for C_17_H_16_N_4_NaO_3_ 347.1120; found 347.1129.

#### *N*-(1-(3-Hydroxy-3-methylbutoxy)-2-oxo-2-phenylethyl)benzamide (**3z**)

The general procedure was performed using *N*-(2-oxo-2-phenylethyl)benzamide **1d** (47.8 mg, 0.2 mmol) and 3-methylbutane-1,3-diol **2w** (25.0 mg, 26.0 μL, 0.24 mmol). Purification by chromatography (petroleum ether/EtOAc = 1:1) afforded compound **3z** (46.8 mg, 69% yield) as a white solid. *R_f_* = 0.26 (petroleum ether/EtOAc = 1.5:1). Mp 132.2–136.0 °C. ^1^H NMR (600 MHz, Chloroform-*d*) δ 8.09 (d, *J* = 7.2 Hz, 2H), 7.91 (d, *J* = 7.1 Hz, 2H), 7.71 (d, *J* = 8.6 Hz, 1H), 7.65 (t, *J* = 7.4 Hz, 1H), 7.57 (t, *J* = 7.4 Hz, 1H), 7.53 (t, *J* = 7.8 Hz, 2H), 7.49 (t, *J* = 7.6 Hz, 2H), 6.63 (d, *J* = 8.6 Hz, 1H), 4.07–4.00 (m, 2H), 2.39 (bs, 1H), 1.85–1.76 (m, 2H), 1.19 (s, 3H), 1.17 (s, 3H); ^13^C{^1^H} NMR (151 MHz, Chloroform-*d*) δ 192.1, 167.9, 134.5, 133.5, 133.3, 132.4, 129.4, 128.9, 128.8, 127.3, 77.4, 70.1, 65.9, 41.8, 29.4, 29.3. HRMS (ESI) *m*/*z*: [M + Na]^+^ calcd for C_20_H_23_NNaO_4_ 364.1525; found 364.1531.

#### *N*-(1-(((3*S*,8*S*,9*S*,10*R*,13*S*,14*S*,17*S*)-17-Acetyl-10,13-dimethyl-2,3,4,7,8,9,10,11,12,13,14,15,16,17-tetradecahydro-1*H*-cyclopenta[*a*]phenanthren-3-yl)oxy)-2-oxo-2-phenylethyl)benzamide (**3aa**)

The general procedure was performed using *N*-(2-oxo-2-phenylethyl)benzamide **1d** (47.8 mg, 0.2 mmol) and pregnenolone **2x** (76.0 mg, 0.24 mmol). Purification by chromatography (petroleum ether/EtOAc = 3:1) afforded diastereomeric mixture **3aa** (65.5 mg, 59% yield) as a white solid at a ratio of 1.39:1 (determined by crude ^1^H NMR). *R_f_* = 0.28 (petroleum ether/EtOAc = 3:1). Mp 82.5–87.6 °C. ^1^H NMR (600 MHz, Chloroform-*d*) δ 8.12 (d, *J* = 7.8 Hz, 2H), 7.90–7.89 (m, 2H), 7.64 (t, *J* = 7.4 Hz, 1H), 7.59–7.56 (m, 2H), 7.55–7.51 (m, 2H), 7.50–7.47 (m, 2H), 6.79 (d, *J* = 5.1 Hz, 0.6H, d1), 6.77 (d, *J* = 5.0 Hz, 0.4H, d2), 5.47–5.46 (m, 0.4H, d2), 5.27–5.26 (m, 0.6H, d1), 3.82–3.74 (m, 1H), 2.53 (t, *J* = 8.9 Hz, 1H), 2.39–2.15 (m, 3H), 2.12 (s, 1.7H, d1), 2.11 (s, 1.3H, d2), 2.05–1.91 (m, 3H), 1.81–1.42 (m, 10H), 1.30–1.05 (m, 3H), 0.99 (s, 1.7H, d1), 0.98 (s, 1.3H, d2), 0.62 (s, 3H). ^13^C{^1^H} NMR (151 MHz, Chloroform-*d*) δ 209.6, 192.6 (d2), 192.5 (d1), 167.7 (d1), 167.6 (d2), 140.4 (d1), 140.1 (d2), 134.2 (d2), 134.2 (d1), 133.8, 133.6 (d2), 133.6 (d1), 132.2 (d1), 132.2 (d2), 129.5 (d1), 129.5 (d2), 128.8 (d2), 128.8 (d1), 128.7 (d1), 128.7 (d2), 127.3, 122.1 (d2), 121.7 (d1), 77.9 (d2), 77.6 (d1), 75.4 (d2), 74.9 (d1), 63.7, 56.9 (d2), 56.9 (d1), 49.9 (d2), 49.8 (d1), 44.0, 40.0, 38.9 (d1), 38.8 (d2), 37.2 (d2), 37.2 (d1), 36.7 (d1), 36.7 (d2), 31.8 (d2), 31.8 (d1), 31.7 (d2), 31.5 (d1), 29.5 (d2), 28.0 (d1), 24.5 (d2), 24.5 (d1), 22.8 (d2), 22.8 (d1), 21.0 (d1), 21.0 (d2), 19.3 (d1), 19.3 (d2), 13.2. HRMS (ESI) *m*/*z*: [M + Na]^+^ calcd for C_36_H_43_NNaO_4_ 576.3090; found 576.3086.

#### *N*-(1-(((3*S*,8*R*,9*S*,10*R*,13*S*,14*S*)-10,13-Dimethyl-17-oxo-2,3,4,7,8,9,10,11,12,13,14,15,16,17-tetradecahydro-1*H*-cyclopenta[*a*]phenanthren-3-yl)oxy)-2-oxo-2-phenylethyl)benzamide (**3ab**)

The general procedure was performed using *N*-(2-oxo-2-phenylethyl)benzamide **1d** (47.8 mg, 0.2 mmol) and dehydroepiandrosterone **2y** (69.2 mg, 0.24 mmol). Purification by chromatography (petroleum ether/EtOAc = 3:1) afforded diastereomeric mixture **3ab** (83.8 mg, 80% yield) as a white solid at a ratio of 1:1 (determined by crude ^1^H NMR). *R_f_* = 0.23 (petroleum ether/EtOAc = 3:1). Mp 82.0–94.7 °C. ^1^H NMR (600 MHz, Chloroform-*d*) δ 8.12 (d, *J* = 7.7 Hz, 2H), 7.92–7.88 (m, 2H), 7.64 (t, *J* = 7.4 Hz, 1H), 7.61–7.57 (m, 1H), 7.56–7.52 (m, 3H), 7.50–7.47 (m, 2H), 6.79 (d, *J* = 1.4 Hz, 0.5H, d1), 6.78 (d, *J* = 1.4 Hz, 0.5H, d2), 5.49 (d, *J* = 4.9 Hz, 0.5H, d1), 5.29 (d, *J* = 4.9 Hz, 0.5H, d2), 3.81–3.75 (m, 1H), 2.48–2.42 (m, 1H), 2.38–2.24 (m, 2H), 2.14–2.06 (m, 2H), 1.98–1.90 (m, 1H), 1.86–1.79 (m, 2H), 1.70–1.42 (m, 8H), 1.30–1.24 (m, 2H), 1.18–1.13 (m, 0.5H, d1), 1.08–1.05 (m, 0.5H, d2), 1.01 (s, 1.5H, d1), 1.00 (s, 1.5H, d2), 0.87 (s, 3H); ^13^C{^1^H} NMR (151 MHz, Chloroform-*d*) δ 192.5, 192.5, 167.6 (d1), 167.6 (d2), 140.7 (d1), 140.4 (d2), 134.2, 133.8 (d1), 133.8 (d2), 133.6 (d1), 133.5 (d2), 132.2, 129.5 (d1), 129.5 (d2), 128.8 (d1), 128.7 (d2), 127.3, 121.6, 121.2, 77.7 (d1), 77.5 (d2), 75.3 (d1), 75.0 (d2), 51.7 (d1), 51.7 (d2), 50.1 (d1), 50.1 (d2), 47.5, 40.0 (d1), 38.9 (d2), 37.2 (d1), 37.1 (d2), 36.8 (d1), 36.8 (d2), 35.8 (d1), 35.8 (d2), 31.5 (d1), 31.4 (d2), 31.4, 30.8 (d1), 30.7 (d2), 29.5 (d1), 28.0 (d2), 21.9 (d1), 21.8 (d2), 20.3 (d1), 20.3 (d2), 19.4 (d1), 19.3 (d2), 13.5. HRMS (ESI) *m*/*z*: [M + Na]^+^ calcd for C_34_H_39_NNaO_4_ 548.2777; found 548.2776.

#### *N*-(1-(((3*S*,8*R*,9*S*,10*S*,13*S*,14*S*)-10,13-Dimethyl-17-oxohexadecahydro-1*H*-cyclopenta[*a*]phenanthren-3-yl)oxy)-2-oxo-2-phenylethyl)benzamide (**3ac**)

The general procedure was performed using *N*-(2-oxo-2-phenylethyl)benzamide **1d** (47.8 mg, 0.2 mmol) and epiandrosterone **2z** (69.6 mg, 0.24 mmol). Purification by chromatography (petroleum ether/EtOAc = 3:1) afforded diastereomeric mixture **3ac** (70.0 mg, 66% yield) as a white solid at a ratio of 1:1 (determined by crude ^1^H NMR). *R_f_* = 0.38 (petroleum ether/EtOAc = 3:1). Mp 88.3–100.1 °C. ^1^H NMR (600 MHz, Chloroform-*d*) δ 8.12 (d, *J* = 8.4 Hz, 2H), 7.90 (d, *J* = 8.4 Hz, 2H), 7.65–7.62 (m, 1H), 7.61–7.59 (m, 1H), 7.56 (t, *J* = 7.4 Hz, 1H), 7.54–7.51 (m, 2H), 7.49 (t, *J* = 7.6 Hz, 2H), 6.78 (d, *J* = 2.8 Hz, 0.5H, d1), 6.77 (d, *J* = 2.8 Hz, 0.5H, d2), 3.88–3.82 (m, 1H), 2.45–2.39 (m, 1H), 2.09–2.02 (m, 1H), 1.95–1.88 (m, 1H), 1.81–1.72 (m, 3H), 1.69–1.59 (m, 3H), 1.54–1.18 (m, 11H), 1.17–0.91 (m, 2H), 0.84 (s, 3H), 0.80 (s, 3H); ^13^C{^1^H} NMR (151 MHz, Chloroform-*d*) δ 192.59, 167.72 (d1), 167.63 (d2), 134.1 (d1), 134.1 (d2), 133.8 (d1), 133.8 (d2), 133.6, 132.2 (d1), 132.2 (d2), 129.5, 128.7 (d1), 128.7 (d2), 128.7, 127.3, 77.5, 75.2 (d1), 75.1 (d2), 54.3 (d1), 54.3(d2), 51.4 (d1), 51.4 (d2), 47.8, 44.9 (d1), 44.7 (d2), 37.0 (d1), 36.9 (d2), 35.9 (d1), 35.8 (d2), 35.8 (d1), 35.8 (d2), 35.0 (d1), 35.0 (d2), 34.4, 31.5 (d1), 31.5 (d2), 30.8 (d1), 30.8 (d2), 29.3 (d1), 28.5 (d2), 28.3 (d1), 27.9 (d2), 21.8 (d1), 21.7 (d2), 20.4 (d1), 20.4 (d2), 13.8, 12.2 (d1), 12.2 (d2). HRMS (ESI) *m*/*z*: [M + Na]^+^ calcd for C_34_H_41_NNaO_4_ 550.2933; found 550.2924.

#### *N*-(2-Oxo-2-phenyl-1-(((3*R*,4*S*,5*R*,6*R*)-3,4,5-tris(benzyloxy)-6-((benzyloxy)methyl)tetrahydro-2*H*-pyran-2-yl)oxy)ethyl)benzamide (**3ad**)

The general procedure was performed using *N*-(2-oxo-2-phenylethyl)benzamide **1d** (47.8 mg, 0.2 mmol) and 2,3,4,6-tetra-*O*-benzyl-d-glucopyranose **2aa** (129.5 mg, 0.24 mmol). Purification by chromatography (petroleum ether/EtOAc = 1:1) afforded a single diastereomer **3ad** (20.2 mg, 13% yield) as a white solid and a diastereomeric mixture of three other diastereomers (38.5 mg, 25% yield) as a white solid at a ratio of 1.00:0.90:0.48 (determined by ^1^H NMR of isolated product). single diastereomer: *R_f_* = 0.35 (petroleum ether/EtOAc = 1:1). Mp 89.1–92.8 °C. ^1^H NMR (600 MHz, Chloroform-*d*) δ 8.34 (d, *J* = 7.3 Hz, 2H), 7.77 (d, *J* = 7.2 Hz, 2H), 7.62 (d, *J* = 9.1 Hz, 1H), 7.57 (t, *J* = 7.4 Hz, 1H), 7.54 (t, *J* = 7.4 Hz, 1H), 7.45–7.40 (m, 6H), 7.34 (t, *J* = 7.4 Hz, 2H), 7.30–7.25 (m, 8H), 7.23–7.19 (m, 8H), 7.10 (d, *J* = 9.1 Hz, 1H), 4.99 (d, *J* = 8.0 Hz, 1H), 4.87 (d, *J* = 11.1 Hz, 1H), 4.83 (d, *J* = 11.0 Hz, 1H), 4.78–4.74 (m, 3H), 4.65–4.61 (m, 3H), 3.94–3.88 (m, 2H), 3.78 (t, *J* = 9.4 Hz, 1H), 3.69–3.64 (m, 2H), 3.46 (dd, *J* = 9.1, 8.0 Hz, 1H); ^13^C{^1^H} NMR (151 MHz, Chloroform-*d*) δ 191.4, 168.0, 138.6, 138.5, 138.3, 138.3, 134.3, 133.4, 133.3, 132.2, 130.1, 128.7, 128.7, 128.4, 128.3, 128.3, 128.2, 127.9, 127.8, 127.8, 127.6, 127.6, 127.6, 127.5, 127.5, 127.4, 100.8, 84.7, 82.0, 77.5, 75.6, 75.1, 74.8, 74.6, 74.4, 73.6, 68.9. HRMS (ESI) *m*/*z*: [M + Na]^+^ calcd for C_49_H_47_NNaO_8_ 800.3199; found 800.3203. diastereomeric mixture: *R_f_* = 0.30 (petroleum ether/EtOAc = 1:1). The NMR spectrum is complicated and not be analyzed. HRMS (ESI) *m*/*z*: [M + Na]^+^ calcd for C_49_H_47_NNaO_8_ 800.3199; found 800.3205.

#### (9*H*-Fluoren-9-yl)methyl ((2*R*)-1-(1-benzamido-2-oxo-2-phenylethoxy)-3-phenylpropan-2-yl)carbamate (**3ae**)

The general procedure was performed using *N*-(2-oxo-2-phenylethyl)benzamide **1d** (47.8 mg, 0.2 mmol) and (9*H*-fluoren-9-yl)methyl (*R*)-(1-hydroxy-3-phenylpropan-2-yl)carbamate **2ab** (89.5 mg, 0.24 mmol). Purification by chromatography (petroleum ether/EtOAc = 1:1) afforded diastereomeric mixture **3ae** (74.1 mg, 61% yield) as a white solid at a ratio of 1:1 (determined by crude ^1^H NMR). *R_f_* = 0.44 (petroleum ether/EtOAc = 1:1). Mp 140.3–147.1. ^1^H NMR (600 MHz, Chloroform-*d*) δ 8.12 (d, *J* = 7.7 Hz, 2H), 7.88–7.85 (m, 2H), 7.76–7.70 (m, 3H), 7.64 (t, *J* = 7.0 Hz, 1H), 7.57–7.45 (m, 7H), 7.40–7.37 (m, 2H), 7.37–7.26 (m, 2H), 7.19–7.16 (m, 2H), 7.17–7.06 (m, 3H), 6.66 (d, *J* = 8.6 Hz, 0.5H, d1), 6.63 (d, *J* = 8.5 Hz, 0.5H, d2), 5.14 (d, *J* = 8.8 Hz, 0.5H, d1), 5.07 (d, *J* = 9.0 Hz, 0.5H, d2), 4.34–4.30 (m, 1H), 4.23–4.21 (m, 0.5H, d1), 4.18–4.15 (m, 0.5H, d2), 4.12–4.09 (m, 1H), 4.04 (bs, 1H), 3.81–3.64 (m, 2H), 2.85–2.75 (m, 2H); ^13^C{^1^H} NMR (151 MHz, Chloroform-*d*) δ 192.1 (d1), 192.0 (d2), 167.9 (d1), 167.8 (d2), 155.7 (d2), 155.7 (d1), 143.9 (d1), 143.9 (d2), 141.3, 137.5 (d2), 137.4 (d1), 134.7 (d2), 134.6 (d1), 133.5 (d1), 133.5 (d2), 133.2 (d1), 133.2 (d2), 132.4 (d2), 132.4 (d1), 129.5 (d1), 129.5 (d2), 129.3 (d1), 129.2 (d2), 129.0 (d2), 128.9 (d1), 128.7 (d1), 128.7 (d2), 128.5 (d1), 128.4 (d2), 127.6, 127.3 (d1), 127.3 (d2), 127.0 (d2), 127.0 (d1), 126.5 (d1), 126.4 (d2), 125.1 (d2), 125.0 (d1), 119.9 (d2), 119.9 (d1), 77.4, 67.6 (d1), 67.5 (d2), 66.7 (d2), 66.6 (d1), 52.0 (d1), 52.0 (d2), 47.2 (d1), 47.1 (d2), 37.6 (d1), 37.5 (d2). HRMS (ESI) *m*/*z*: [M + Na]^+^ calcd for C_39_H_34_N_2_NaO_5_ 633.2365; found 633.2357.

#### *N*-(1-(Benzyloxy)-2-oxo-2-phenylethyl)-4-fluorobenzamide (**3af**)

The general procedure was performed using 4-fluoro-*N*-(2-oxo-2-phenylethyl)benzamide **1af** (51.4 mg, 0.2 mmol) and benzyl alcohol **2a** (26.0 mg, 24.8 μL, 0.24 mmol). Purification by chromatography (petroleum ether/EtOAc = 4:1) afforded compound **3af** (62.8 mg, 87% yield) as a white solid. *R_f_* = 0.39 (petroleum ether/EtOAc = 4:1). Mp 120.2–124.5 °C. ^1^H NMR (600 MHz, Chloroform-*d*) δ 7.94–7.91 (m, 4H), 7.66 (d, *J* = 9.0 Hz, 1H), 7.60 (t, *J* = 7.4 Hz, 1H), 7.44 (t, *J* = 7.8 Hz, 2H), 7.39 (d, *J* = 6.9 Hz, 2H), 7.35–7.28 (m, 3H), 7.16 (t, *J* = 8.6 Hz, 2H), 6.71 (d, *J* = 8.7 Hz, 1H), 4.87 (dd, 32.0, 11.7 Hz, 2H); ^13^C{^1^H} NMR (151 MHz, Chloroform-*d*) δ 192.0, 166.9, 165.2 (d, *J*_*CF*_ = 253.3 Hz), 136.9, 134.3, 133.5, 129.7, 129.7, 129.6 (d, *J*_*CF*_ = 3.2 Hz), 129.5, 128.7, 128.5, 128.1, 115.8 (d, *J*_*CF*_ = 22.0 Hz), 76.7, 70.8; ^19^F NMR (376 MHz, Chloroform-*d*) δ −106.76. HRMS (ESI) *m*/*z*: [M + Na]^+^ calcd for C_22_H_18_FNNaO_3_ 386.1168; found 386.1161.

#### *N*-(1-(Benzyloxy)-2-oxo-2-phenylethyl)-4-chlorobenzamide (**3ag**)

The general procedure was performed using 4-chloro-*N*-(2-oxo-2-phenylethyl)benzamide **1ag** (54.6 mg, 0.2 mmol) and benzyl alcohol **2a** (26.0 mg, 24.8 μL, 0.24 mmol). Purification by chromatography (petroleum ether/EtOAc = 5:1) afforded compound **3ag** (60.5 mg, 80% yield) as a white solid. *R_f_* = 0.43 (petroleum ether/EtOAc = 4:1). Mp 162.4–164.7 °C. ^1^H NMR (600 MHz, Chloroform-*d*) δ 7.92 (d, *J* = 7.4 Hz, 2H), 7.83 (d, *J* = 8.5 Hz, 2H), 7.70 (d, *J* = 8.8 Hz, 1H), 7.60 (t, *J* = 7.4 Hz, 1H), 7.47–7.42 (m, 4H), 7.38 (d, *J* = 6.8 Hz, 2H), 7.35–7.28 (m, 3H), 6.71 (d, *J* = 8.7 Hz, 1H), 4.86 (dd, *J* = 30.7, 11.7 Hz, 2H); ^13^C{^1^H} NMR (151 MHz, Chloroform-*d*) δ 191.9, 166.9, 138.6, 136.9, 134.3, 133.4, 131.8, 129.5, 129.0, 128.8, 128.7, 128.5, 128.4, 128.1, 76.7, 70.9. HRMS (ESI) *m*/*z*: [M + Na]^+^ calcd for C_22_H_18_ClNNaO_3_ 402.0873; found 402.0866.

#### *N*-(1-(Benzyloxy)-2-oxo-2-phenylethyl)-4-bromobenzamide (**3ah**)

The general procedure was performed using 4-bromo-*N*-(2-oxo-2-phenylethyl)benzamide **1ah** (63.4 mg, 0.2 mmol) and benzyl alcohol **2a** (26.0 mg, 24.8 μL, 0.24 mmol). Purification by chromatography (petroleum ether/EtOAc = 5:1) afforded compound **3ah** (72.7 mg, 86% yield) as a white solid. *R_f_* = 0.49 (petroleum ether/EtOAc = 4:1). Mp 158.7–162.1 °C. ^1^H NMR (600 MHz, Chloroform-*d*) δ 7.93 (d, *J* = 7.2 Hz, 2H), 7.76 (d, *J* = 8.5 Hz, 2H), 7.67 (d, *J* = 8.8 Hz, 1H), 7.64–7.59 (m, 3H), 7.44 (t, *J* = 7.8 Hz, 2H), 7.40–7.37 (m, 2H), 7.36–7.28 (m, 3H), 6.70 (d, *J* = 8.7 Hz, 1H), 4.86 (dd, *J* = 39.0, 11.7 Hz, 2H); ^13^C{^1^H} NMR (151 MHz, Chloroform-*d*) δ 191.9, 167.0, 136.9, 134.3, 133.4, 132.3, 132.0, 129.5, 128.9, 128.7, 128.5, 128.1, 127.1, 76.7, 70.9. HRMS (ESI) *m*/*z*: [M + Na]^+^ calcd for C_22_H_18_BrNNaO_3_ 446.0368; found 446.0360.

#### *N*-(1-(Benzyloxy)-2-oxo-2-phenylethyl)-2,4,6-trichlorobenzamide (**3ai**)

The general procedure was performed using 2,4,6-trichloro-*N*-(2-oxo-2-phenylethyl)benzamide **1ai** (68.0 mg, 0.2 mmol) and benzyl alcohol **2a** (26.0 mg, 24.8 μL, 0.24 mmol). Purification by chromatography (petroleum ether/EtOAc = 5:1) afforded compound **3ai** (35.3 mg, 39% yield) as a white solid. *R_f_* = 0.46 (petroleum ether/EtOAc = 4:1). Mp 141.1–142.8 °C. ^1^H NMR (600 MHz, Chloroform-*d*) δ 7.90 (d, *J* = 7.2 Hz, 2H), 7.60 (t, *J* = 7.4 Hz, 1H), 7.46–7.41 (m, 4H), 7.40 (s, 2H), 7.38–7.32 (m, 4H), 6.68 (d, *J* = 8.9 Hz, 1H), 4.95 (s, 2H); ^13^C{^1^H} NMR (151 MHz, Chloroform-*d*) δ 190.9, 164.6, 136.5, 136.3, 134.4, 133.9, 133.2, 132.8, 129.6, 128.8, 128.73, 128.5, 128.3, 128.3, 76.1, 71.0. HRMS (ESI) *m*/*z*: [M + Na]^+^ calcd for C_22_H_16_Cl_3_NaNO_3_ 470.0093; found 470.0081.

#### *N*-(2-Oxo-2-phenylethyl)thiophene-2-carboxamide (**3aj**)

The general procedure was performed using 4-fluoro-*N*-(2-oxo-2-phenylethyl)benzamide **1aj** (49.0 mg, 0.2 mmol) and benzyl alcohol **2a** (26.0 mg, 24.8 μL, 0.24 mmol). Purification by chromatography (petroleum ether/EtOAc = 3:1) afforded compound **3aj** (63.4 mg, 90% yield) as a white solid. *R_f_* = 0.40 (petroleum ether/EtOAc = 3:1). Mp 126.4–130.4 °C. ^1^H NMR (600 MHz, Chloroform-*d*) δ 7.91 (d, *J* = 7.3 Hz, 2H), 7.68 (dd, *J* = 3.8, 1.1 Hz, 1H), 7.62–7.54 (m, 3H), 7.45–7.38 (m, 4H), 7.36–7.29 (m, 3H), 7.14 (dd, *J* = 5.0, 3.7 Hz, 1H), 6.67 (d, *J* = 8.8 Hz, 1H), 4.87 (dd, *J* = 27.3, 11.7 Hz, 2H); ^13^C{^1^H} NMR (151 MHz, Chloroform-*d*) δ 191.8, 162.4, 138.0, 136.9, 134.3, 133.5, 131.4, 129.5, 129.1, 128.7, 128.6, 128.5, 128.1, 127.9, 76.4, 70.8. HRMS (ESI) *m*/*z*: [M + Na]^+^ calcd for C_20_H_17_NNaO_3_S 374.0827; found 374.0832.

#### *N*-(1-(Benzyloxy)-2-oxo-2-phenylethyl)furan-2-carboxamide (**3ak**)

The general procedure was performed using *N*-(2-oxo-2-phenylethyl)furan-2-carboxamide **1ak** (45.8 mg, 0.2 mmol) and benzyl alcohol **2a** (26.0 mg, 24.8 μL, 0.24 mmol). Purification by chromatography (petroleum ether/EtOAc = 3:1) afforded compound **3ak** (64.7 mg, 97% yield) as a white solid. *R_f_* = 0.27 (petroleum ether/EtOAc = 4:1). Mp 106.3–107.6 °C. ^1^H NMR (600 MHz, Chloroform-*d*) δ 7.91 (dd, *J* = 8.3, 1.1 Hz, 2H), 7.83 (d, *J* = 9.1 Hz, 1H), 7.59 (t, *J* = 7.4 Hz, 1H), 7.55 (dd, *J* = 1.6, 0.7 Hz, 1H), 7.43 (t, *J* = 7.9 Hz, 2H), 7.41–7.38 (m, 2H), 7.35–7.29 (m, 3H), 7.25 (dd, *J* = 3.5, 0.8 Hz, 1H), 6.66 (d, *J* = 9.1 Hz, 1H), 6.56 (dd, *J* = 3.5, 1.7 Hz, 1H), 4.84 (dd, *J* = 25.2, 11.5 Hz, 2H); ^13^C{^1^H} NMR (151 MHz, Chloroform-*d*) δ 191.6, 158.8, 147.1, 144.8, 136.8, 134.2, 133.5, 129.5, 128.7, 128.6, 128.4, 128.1, 115.7, 112.38, 75.9, 70.6. HRMS (ESI) *m*/*z*: [M + Na]^+^ calcd for C_20_H_17_NNaO_4_ 358.1055; found 358.1045.

#### *N*-(1-(Benzyloxy)-2-oxo-2-phenylethyl)nicotinamide (**3al**)

The general procedure was performed using *N*-(2-oxo-2-phenylethyl)nicotinamide **1al** (48.0 mg, 0.2 mmol) and benzyl alcohol **2a** (26.0 mg, 24.8 μL, 0.24 mmol). Purification by chromatography (petroleum ether/EtOAc = 1:1) afforded compound **3al** (27.1 mg, 39% yield) as a pale yellow solid. *R_f_* = 0.23 (petroleum ether/EtOAc = 1:1). Mp 137.4–139.2 °C. ^1^H NMR (600 MHz, Chloroform-*d*) δ 9.12 (d, *J* = 1.8 Hz, 1H), 8.80 (dd, *J* = 4.9, 1.7 Hz, 1H), 8.19–8.17 (m, 1H), 7.96–7.92 (m, 2H), 7.73 (d, *J* = 8.7 Hz, 1H), 7.62 (t, *J* = 7.4 Hz, 1H), 7.48–7.43 (m, 3H), 7.41–7.38 (m, 2H), 7.36–7.29 (m, 3H), 6.72 (d, *J* = 8.7 Hz, 1H), 4.88 (dd, 31.0, *J* = 11.8 Hz, 2H); ^13^C{^1^H} NMR (151 MHz, Chloroform-*d*) δ 191.7, 166.2, 153.0, 148.5, 136.8, 135.1, 134.4, 133.4, 129.5, 129.2, 128.8, 128.5, 128.5, 128.2, 123.5, 76.6, 71.0. HRMS (ESI) *m*/*z*: [M + Na]^+^ calcd for C_21_H_18_N_2_NaO_3_ 369.1215; found 369.1225.

#### *N*-(1-(Benzyloxy)-2-oxo-2-phenylethyl)acetamide (**3am**)

The general procedure was performed using *N*-(2-oxo-2-phenylethyl)acetamide **1am** (35.4 mg, 0.2 mmol) and benzyl alcohol **2a** (26.0 mg, 24.8 μL, 0.24 mmol). Purification by chromatography (petroleum ether/EtOAc = 1.5:1) afforded compound **3am** (47.9 mg, 85% yield) as a white solid. *R_f_* = 0.44 (petroleum ether/EtOAc = 1:1). Mp 87.3–88.4 °C. ^1^H NMR (600 MHz, Chloroform-*d*) δ 7.89 (d, *J* = 7.9 Hz, 2H), 7.58 (t, *J* = 7.1 Hz, 1H), 7.42 (t, *J* = 7.8 Hz, 2H), 7.38–7.28 (m, 5H), 7.01 (d, *J* = 9.2 Hz, 1H), 6.52 (d, *J* = 9.1 Hz, 1H), 4.78 (dd, *J* = 22.8, 11.7 Hz, 2H), 2.13 (s, 3H); ^13^C{^1^H} NMR (151 MHz, Chloroform-*d*) δ 192.0, 171.1, 137.0, 134.2, 133.5, 129.4, 128.7, 128.4, 128.3, 128.0, 76.2, 70.6, 23.5. HRMS (ESI) *m*/*z*: [M + Na]^+^ calcd for C_17_H_17_NNaO_3_ 306.1106; found 306.1117.

#### *N*-(1-(Benzyloxy)-2-oxo-2-phenylethyl)cyclopropanecarboxamide (**3an**)

The general procedure was performed using *N*-(2-oxo-2-phenylethyl)benzamide **1an** (40.6 mg, 0.2 mmol) and benzyl alcohol **2a** (26.0 mg, 24.8 μL, 0.24 mmol). Purification by chromatography (petroleum ether/EtOAc = 3:1) afforded compound **3an** (60.9 mg, 99% yield) as a white solid. *R_f_* = 0.40 (petroleum ether/EtOAc = 3:1). Mp 118.0–121.0 °C. ^1^H NMR (600 MHz, Chloroform-*d*) δ 7.87 (dd, *J* = 8.4, 1.4 Hz, 2H), 7.57 (t, *J* = 7.4 Hz, 1H), 7.41 (t, *J* = 7.8 Hz, 2H), 7.38–7.36 (m, 2H), 7.35–7.28 (m, 3H), 7.18 (d, *J* = 9.4 Hz, 1H), 6.52 (dd, *J* = 9.1, 0.9 Hz, 1H), 4.78 (dd, *J* = 17.5, 11.7 Hz, 2H), 1.57–1.53 (m, 1H), 1.12–1.08 (m, 1H), 1.05–1.01 (m, 1H), 0.91–0.82 (m, 2H); ^13^C{^1^H} NMR (151 MHz, Chloroform-*d*) δ 192.1, 174.8, 137.0, 134.1, 133.6, 129.4, 128.6, 128.5, 128.4, 128.0, 76.0, 70.5, 15.0, 8.2, 8.0. HRMS (ESI) *m*/*z*: [M + Na]^+^ calcd for C_19_H_19_NNaO_3_ 332.1263; found 332.1266.

#### *N*-(1-(Benzyloxy)-2-oxo-2-phenylethyl)cyclobutanecarboxamide (**3ao**)

The general procedure was performed using *N*-(2-(4-bromophenyl)-2-oxoethyl)acetamide **1ao** (43.4 mg, 0.2 mmol) and benzyl alcohol **2a** (26.0 mg, 24.8 μL, 0.24 mmol). Purification by chromatography (petroleum ether/EtOAc = 4:1) afforded compound **3ao** (52.9 mg, 82% yield) as a white solid. *R_f_* = 0.34 (petroleum ether/EtOAc = 4:1). Mp 105.6–108.5 °C. ^1^H NMR (600 MHz, Chloroform-*d*) δ 7.89–7.85 (m, 2H), 7.58 (t, *J* = 7.4 Hz, 1H), 7.41 (t, *J* = 7.8 Hz, 2H), 7.39–7.36 (m, 2H), 7.35–7.29 (m, 3H), 6.84 (d, *J* = 9.1 Hz, 1H), 6.51 (d, *J* = 9.1 Hz, 1H), 4.84–4.78 (dd, *J* = 26.1, 11.8 Hz, 2H), 3.19–3.13 (m, 1H), 2.38–2.28 (m, 2H), 2.28–2.18 (m, 2H), 2.05–1.97 (m, 1H), 1.95–1.87 (m, 1H); ^13^C{^1^H} NMR (151 MHz, Chloroform-*d*) δ 192.0, 176.0, 137.1, 134.1, 133.6, 129.4, 128.6, 128.4, 128.4, 128.0, 76.0, 70.6, 39.9, 25.3, 25.2, 18.1. HRMS (ESI) *m*/*z*: [M + Na]^+^ calcd for C_20_H_21_NNaO_3_ 346.1419; found 346.1418.

#### *N*-(1-(Benzyloxy)-2-oxo-2-phenylethyl)pivalamide (**3ap**)

The general procedure was performed using *N*-(2-oxo-2-phenylethyl)pivalamide **1ap** (43.8 mg, 0.2 mmol) and benzyl alcohol **2a** (26.0 mg, 24.8 μL, 0.24 mmol). Purification by chromatography (petroleum ether/EtOAc = 5:1) afforded compound **3ap** (58.4 mg, 89% yield) as a white solid. *R_f_* = 0.45 (petroleum ether/EtOAc = 4:1). Mp 114.9–116.8 °C. ^1^H NMR (600 MHz, Chloroform-*d*) δ 7.88 (dd, *J* = 8.4, 1.4 Hz, 2H), 7.58 (t, *J* = 7.4 Hz, 1H), 7.42 (t, *J* = 7.9 Hz, 2H), 7.39–7.37 (m, 2H), 7.35–7.28 (m, 3H), 7.18 (d, *J* = 8.9 Hz, 1H), 6.50 (d, *J* = 8.8 Hz, 1H), 4.77 (dd, *J* = 26.6, 11.8 Hz, 2H), 1.28 (s, 9H); ^13^C{^1^H} NMR (151 MHz, Chloroform-*d*) δ 192.2, 179.5, 137.1, 134.1, 133.6, 129.4, 128.7, 128.4, 128.0, 76.2, 70.6, 39.2, 27.4. HRMS (ESI) *m*/*z*: [M + Na]^+^ calcd for C_20_H_23_NNaO_3_ 348.1576; found 348.1563.

#### *N*-(1-(Benzyloxy)-2-oxo-2-phenylethyl)-2,2,2-trifluoroacetamide (**3aq**)

The general procedure was performed using 2,2,2-trifluoro-*N*-(2-oxo-2-phenylethyl)acetamide **1aq** (46.2 mg, 0.2 mmol) and benzyl alcohol **2a** (26.0 mg, 24.8 μL, 0.24 mmol). Purification by chromatography (petroleum ether/EtOAc = 4:1) afforded compound **3aq** (20.5 mg, 30% yield) as a white solid. *R_f_* = 0.47 (petroleum ether/EtOAc = 4:1). Mp 80.4–82.8 °C. ^1^H NMR (600 MHz, Chloroform-*d*) δ 7.87–7.81 (m, 3H), 7.62 (t, *J* = 7.4 Hz, 1H), 7.44 (t, *J* = 7.9 Hz, 2H), 7.37–7.34 (d, *J* = 3.1 Hz, 5H), 6.47 (d, *J* = 8.6 Hz, 1H), 4.79 (s, 2H); ^13^C{^1^H} NMR (151 MHz, Chloroform-*d*) δ 190.2, 158.1 (q, *J*_*CF*_ = 38.3 Hz), 135.9, 134.7, 132.8, 129.5, 128.9, 128.6, 128.6, 128.5, 115.6 (q, *J*_*CF*_ = 288.0 Hz), 76.5, 71.5; ^19^F NMR (376 MHz, Chloroform-*d*) δ −75.91. HRMS (ESI) *m*/*z*: [M + Na]^+^ calcd for C_17_H_14_F_3_NNaO_3_ 360.0823; found 360.0834.

#### *N*-(1-(Benzyloxy)-2-oxo-2-phenylethyl)-2-chloropropanamide (**3ar**)

The general procedure was performed using dl-2-chloro-*N*-(2-oxo-2-phenylethyl)propanamide **1ar** (45.0 mg, 0.2 mmol) and benzyl alcohol **2a** (26.0 mg, 24.8 μL, 0.24 mmol). Purification by chromatography (petroleum ether/EtOAc = 4:1) afforded diastereomeric mixture **3ar** (50.7 mg, 77% yield) as a white solid at a ratio of 1.36:1 (determined by crude ^1^H NMR). *R_f_* = 0.35 (petroleum ether/EtOAc = 4:1). Mp 79.7–81.0 °C. ^1^H NMR (600 MHz, Chloroform-*d*) δ 7.95 (bs, 1H), 7.89 (t, *J* = 8.3 Hz, 2H), 7.59 (t, *J* = 7.4 Hz, 1H), 7.49–7.41 (m, 2H), 7.39–7.30 (m, 5H), 6.48–6.45 (m, 1H), 4.77 (dd, *J* = 18.6, 11.7 Hz, 2H), 4.52–4.46 (m, 1H), 1.78–1.75 (m, 3H); ^13^C{^1^H} NMR (151 MHz, Chloroform-*d*) δ 191.3 (d1), 191.3 (d2), 170.8 (d1), 170.7 (d2), 136.7 (d1), 136.7 (d2), 134.3 (d1), 134.3 (d2), 133.4, 129.4 (d1), 129.4 (d2), 128.7 (d1), 128.7 (d2), 128.5 (d2), 128.5 (d1), 128.4, 128.2 (d2), 128.2 (d1), 76.7, 70.8 (d2), 70.7 (d1), 55.4 (d1), 55.4 (d2), 22.4 (d1), 22.3 (d2). HRMS (ESI) *m*/*z*: [M + Na]^+^ calcd for C_18_H_18_ClNNaO_3_ 354.0873; found 354.0864.

#### *N*-(1-(Benzyloxy)-2-oxo-2-phenylethyl)acrylamide (**3as**)

The general procedure was performed using *N*-(2-oxo-2-phenylethyl)acrylamide **1as** (37.8 mg, 0.2 mmol) and benzyl alcohol **2a** (26.0 mg, 24.8 μL, 0.24 mmol). Purification by chromatography (petroleum ether/EtOAc = 3:1) afforded compound **3as** (57.8 mg, 98% yield) as a white solid. *R_f_* = 0.33 (petroleum ether/EtOAc = 3:1). Mp 111.7–113.2 °C. ^1^H NMR (600 MHz, Chloroform-*d*) δ 7.89 (d, *J* = 7.3 Hz, 2H), 7.58 (t, *J* = 7.4 Hz, 1H), 7.42 (t, *J* = 7.6 Hz, 2H), 7.37 (d, *J* = 7.0 Hz, 2H), 7.35–7.29 (m, 3H), 7.12 (bs, 1H), 6.60 (d, *J* = 9.0 Hz, 1H), 6.43 (d, *J* = 17.0 Hz, 1H), 6.30–6.25 (m, 1H), 5.80 (d, *J* = 10.3 Hz, 1H), 4.81 (dd, *J* = 25.8, 11.7 Hz, 2H); ^13^C{^1^H} NMR (151 MHz, Chloroform-*d*) δ 191.8, 166.1, 136.9, 134.2, 133.5, 130.4, 129.5, 128.7, 128.5, 128.4, 128.3, 128.1, 76.2, 70.7. HRMS (ESI) *m*/*z*: [M + H]^+^ calcd for C_18_H_17_NNaO_3_ 318.1106; found 318.1112.

#### Benzyl (1-(Benzyloxy)-2-oxo-2-phenylethyl)carbamate (**3at**)

The general procedure was performed using 4-chloro-*N*-(2-oxo-2-phenylethyl)benzamide **1at** (53.8 mg, 0.2 mmol) and benzyl alcohol **2a** (26.0 mg, 24.8 μL, 0.24 mmol). Purification by chromatography (petroleum ether/EtOAc = 5:1) afforded compound **3at** (42.6 mg, 57% yield) as a pale yellow solid. *R_f_* = 0.43 (petroleum ether/EtOAc = 4:1). Mp 77.2–79.0 °C. ^1^H NMR (600 MHz, Chloroform-*d*) δ 7.89 (d, *J* = 6.6 Hz, 2H), 7.58 (t, *J* = 7.4 Hz, 1H), 7.46–7.31 (m, 12H), 6.40 (bs, 1H), 6.33 (s, 1H), 5.20 (s, 2H), 4.78 (s, 2H); ^13^C{^1^H} NMR (151 MHz, Chloroform-*d*) δ 191.45, 156.27, 136.89, 135.9, 134.2, 133.5, 129.4, 128.7, 128.6, 128.4, 128.4, 128.3, 128.1, 128.0, 78.8, 70.0, 67.4. HRMS (ESI) *m*/*z*: [M + Na]^+^ calcd for C_23_H_21_NNaO_4_ 398.1368; found 398.1354.

#### (9*H*-Fluoren-9-yl)methyl (1-(Benzyloxy)-2-oxo-2-phenylethyl)carbamate (**3au**)

The general procedure was performed using (9*H*-fluoren-9-yl)methyl (2-oxo-2-phenylethyl)carbamate **1au** (71.4 mg, 0.2 mmol) and benzyl alcohol **2a** (26.0 mg, 24.8 μL, 0.24 mmol). Purification by chromatography (petroleum ether/EtOAc = 4:1) afforded compound **3au** (59.3 mg, 64% yield) as a white solid. *R_f_* = 0.37 (petroleum ether/EtOAc = 4:1). Mp 116.1–119.5 °C. ^1^H NMR (600 MHz, Chloroform-*d*) δ 7.89 (d, *J* = 7.6 Hz, 2H), 7.78 (t, *J* = 5.9 Hz, 2H), 7.67–7.62 (m, 2H), 7.59 (t, *J* = 7.2 Hz, 1H), 7.48–7.29 (m, 11H), 6.40 (s, 1H), 6.32 (s, 1H), 4.75 (s, 2H), 4.50 (d, *J* = 45.2 Hz, 2H), 4.27 (t, *J* = 6.8 Hz, 1H); ^13^C{^1^H} NMR (151 MHz, Chloroform-*d*) δ 191.5, 156.3, 143.7, 143.5, 141.4, 136.9, 134.2, 133.5, 129.4, 128.7, 128.5, 128.4, 128.1, 127.8, 127.1, 127.1, 125.1, 125.1, 120.1, 120.0, 78.7, 70.1, 67.3, 47.1. HRMS (ESI) *m*/*z*: [M + Na]^+^ calcd for C_30_H_25_NNaO_4_ 486.1681; found 486.1669.

#### *N*-(1-(Benzyloxy)-2-oxo-2-phenylethyl)-4-methylbenzenesulfonamide (**3av**)

The general procedure was performed using 4-methyl-*N*-(2-oxo-2-phenylethyl)benzenesulfonamide **1av** (57.8 mg, 0.2 mmol) and benzyl alcohol **2a** (26.0 mg, 24.8 μL, 0.24 mmol). Purification by chromatography (petroleum ether/EtOAc = 2:1) afforded compound **3av** (49.0 mg, 62% yield) as a white solid. *R_f_* = 0.43 (petroleum ether/EtOAc = 2:1). Mp 138.6–141.4 °C. ^1^H NMR (600 MHz, Chloroform-*d*) δ 7.82 (d, *J* = 7.3 Hz, 2H), 7.75 (d, *J* = 8.3 Hz, 2H), 7.58 (t, *J* = 7.4 Hz, 1H), 7.40 (t, *J* = 7.8 Hz, 2H), 7.29–7.26 (m, 3H), 7.20 (d, *J* = 8.1 Hz, 2H), 7.15–7.12 (m, 2H), 6.36 (d, *J* = 8.4 Hz, 1H), 5.96 (d, *J* = 8.3 Hz, 1H), 4.55 (d, *J* = 20.4, 11.2 Hz, 2H), 2.35 (s, 3H); ^13^C{^1^H} NMR (151 MHz, Chloroform-*d*) δ 191.1, 143.7, 137.8, 136.1, 134.5, 132.9, 129.7, 129.4, 128.7, 128.6, 128.3, 128.1, 126.8, 80.9, 68.4, 21.5. HRMS (ESI) *m*/*z*: [M + Na]^+^ calcd for C_22_H_21_NNaO_4_S 418.1089; found 418.1070.

#### *N*-(1-(Benzyloxy)-2-oxo-2-phenylethyl)morpholine-4-carboxamide (**3aw**)

The general procedure was performed using *N*-(2-oxo-2-phenylethyl)morpholine-4-carboxamide **1aw** (49.6 mg, 0.2 mmol) and benzyl alcohol **2a** (26.0 mg, 24.8 μL, 0.24 mmol). Purification by chromatography (petroleum ether/EtOAc = 1:1) afforded compound **3aw** (33.6 mg, 47% yield) as a white solid. *R_f_* = 0.40 (petroleum ether/EtOAc = 1:1). Mp 116.5–120.1 °C. ^1^H NMR (600 MHz, Chloroform-*d*) δ 7.91 (d, *J* = 7.3 Hz, 2H), 7.58 (t, *J* = 7.4 Hz, 1H), 7.43 (t, *J* = 7.8 Hz, 2H), 7.37 (d, *J* = 6.9 Hz, 2H), 7.35–7.27 (m, 3H), 6.51 (d, *J* = 8.3 Hz, 1H), 6.20 (d, *J* = 8.3 Hz, 1H), 4.83 (dd, *J* = 28.8, 11.8 Hz, 2H), 3.78–3.67 (m, 4H), 3.50–3.42 (m, 4H); ^13^C{^1^H} NMR (151 MHz, Chloroform-*d*) δ 192.6, 156.6, 137.5, 134.2, 133.6, 129.5, 128.7, 128.4, 128.3, 127.9, 78.4, 70.2, 66.4, 44.0. HRMS (ESI) *m*/*z*: [M + Na]^+^ calcd for C_20_H_22_N_2_NaO_4_ 377.1477; found 377.1484.

#### *N*-(1-(Benzyloxy)-2-(4-bromophenyl)-2-oxoethyl)acetamide (**3ax**)

The general procedure was performed using *N*-(2-(4-bromophenyl)-2-oxoethyl)acetamide **1ax** (51.0 mg, 0.2 mmol) and benzyl alcohol **2a** (26.0 mg, 24.8 μL, 0.24 mmol). Purification by chromatography (petroleum ether/EtOAc = 2:1) afforded compound **3ax** (62.8 mg, 87% yield) as a white solid. *R_f_* = 0.33 (petroleum ether/EtOAc = 2:1). Mp 147.9–149.2 °C. ^1^H NMR (600 MHz, Chloroform-*d*) δ 7.70 (d, *J* = 8.6 Hz, 2H), 7.54 (d, *J* = 8.6 Hz, 2H), 7.39–7.31 (m, 5H), 6.91 (d, *J* = 9.1 Hz, 1H), 6.44 (d, *J* = 9.1 Hz, 1H), 4.76 (dd, *J* = 19.0, 11.7 Hz, 2H), 2.14 (s, 3H); ^13^C{^1^H} NMR (151 MHz, Chloroform-*d*) δ 191.1, 171.1, 136.7, 132.3, 132.0, 130.9, 129.6, 128.5, 128.5, 128.2, 76.0, 70.6, 23.5. HRMS (ESI) *m*/*z*: [M + Na]^+^ calcd for C_17_H_16_BrNNaO_3_ 384.0211; found 384.0212.

#### *N*-(1-(Benzyloxy)-2-(3-fluorophenyl)-2-oxoethyl)acetamide (**3ay**)

The general procedure was performed using *N*-(2-(3-fluorophenyl)-2-oxoethyl)acetamide **1ay** (39.0 mg, 0.2 mmol) and benzyl alcohol **2a** (26.0 mg, 24.8 μL, 0.24 mmol). Purification by chromatography (petroleum ether/EtOAc = 1.5:1) afforded compound **3ay** (49.7 mg, 83% yield) as a white solid. *R_f_* = 0.47 (petroleum ether/EtOAc = 1:1). Mp 103.2–105.8 °C. ^1^H NMR (600 MHz, Chloroform-*d*) δ 7.63 (d, *J* = 7.8 Hz, 1H), 7.57–7.55 (m, 1H), 7.43–7.27 (m, 7H), 6.88 (d, *J* = 9.1 Hz, 1H), 6.45 (d, *J* = 9.1 Hz, 1H), 4.78 (dd, *J* = 18.1, 11.7 Hz, 2H), 2.14 (s, 3H); ^13^C{^1^H} NMR (151 MHz, Chloroform-*d*) δ 190.9 (d, *J*_*CF*_ = 2.3 Hz), 171.0, 162.7 (d, *J*_*CF*_ = 248.3 Hz), 136.7, 135.6 (d, *J*_*CF*_ = 6.6 Hz), 130.3 (d, *J*_*CF*_ = 7.6 Hz), 128.5, 128.2, 125.2 (d, *J*_*CF*_ = 3.1 Hz), 121.3 (d, *J*_*CF*_ = 21.4 Hz), 116.0 (d, *J*_*CF*_ = 22.6 Hz), 76.2, 70.7, 23.5; ^19^F NMR (376 MHz, Chloroform-*d*) δ −111.31. HRMS (ESI) *m*/*z*: [M + Na]^+^ calcd for C_17_H_16_FNNaO_3_ 324.1012; found 324.1018.

#### *N*-(2-([1,1′-Biphenyl]-4-yl)-1-(benzyloxy)-2-oxoethyl)acetamide (**3az**)

The general procedure was performed using *N*-(2-([1,1′-biphenyl]-4-yl)-2-oxoethyl)acetamide **1az** (50.6 mg, 0.2 mmol) and benzyl alcohol **2a** (26.0 mg, 24.8 μL, 0.24 mmol). Purification by chromatography (petroleum ether/EtOAc = 1.5:1) afforded compound **3az** (45.3 mg, 63% yield) as a white solid. *R_f_* = 0.50 (petroleum ether/EtOAc = 1:1). Mp 157.3–159.0 °C. ^1^H NMR (600 MHz, Chloroform-*d*) δ 7.96 (d, *J* = 8.4 Hz, 2H), 7.66–7.60 (m, 4H), 7.47 (t, *J* = 7.7 Hz, 2H), 7.43–7.37 (m, 3H), 7.36–7.29 (m, 3H), 7.00 (d, *J* = 9.0 Hz, 1H), 6.54 (d, *J* = 9.0 Hz, 1H), 4.80 (dd, *J* = 25.9, 11.7 Hz, 2H), 2.15 (s, 3H); ^13^C{^1^H} NMR (151 MHz, Chloroform-*d*) δ 191.5, 171.1, 146.9, 139.6, 137.0, 132.2, 130.0, 129.0, 128.5, 128.4, 128.41, 128.0, 127.3, 127.3, 76.2, 70.6, 23.5. HRMS (ESI) *m*/*z*: [M + Na]^+^ calcd for C_23_H_21_NNaO_3_ 382.1419; found 382.1404.

#### *N*-(1-(Benzyloxy)-2-(3-methoxyphenyl)-2-oxoethyl)acetamide (**3ba**)

The general procedure was performed using *N*-(2-(3-methoxyphenyl)-2-oxoethyl)acetamide **1ba** (41.4 mg, 0.2 mmol) and benzyl alcohol **2a** (26.0 mg, 24.8 μL, 0.24 mmol). Purification by chromatography (petroleum ether/EtOAc = 1.5:1) afforded compound **3ba** (45.7 mg, 73% yield) as a white solid. *R_f_* = 0.43 (petroleum ether/EtOAc = 1:1). Mp 90.3–91.2 °C. ^1^H NMR (600 MHz, Chloroform-*d*) δ 7.49–7.44 (m, 2H), 7.37–7.27 (m, 6H), 7.13 (dd, *J* = 8.2, 2.0 Hz, 1H), 6.97 (d, *J* = 8.1 Hz, 1H), 6.50 (d, *J* = 9.0 Hz, 1H), 4.78 (dd, *J* = 20.7, 11.7 Hz, 2H), 3.80 (s, 3H), 2.13 (s, 3H); ^13^C{^1^H} NMR (151 MHz, Chloroform-*d*) δ 191.8, 171.01, 159.8, 137.0, 134.9, 129.7, 128.4, 128.3, 128.0, 122.1, 121.1, 113.1, 76.4, 70.6, 55.4, 23.5. HRMS (ESI) *m*/*z*: [M + Na]^+^ calcd for C_18_H_19_NNaO_4_ 336.1206; found 336.1202.

#### *N*-(1-(Benzyloxy)-2-(4-methoxyphenyl)-2-oxoethyl)acetamide (**3bb**)

The general procedure was performed using *N*-(2-(4-methoxyphenyl)-2-oxoethyl)acetamide **1bb** (41.4 mg, 0.2 mmol) and benzyl alcohol **2a** (26.0 mg, 24.8 μL, 0.24 mmol). Purification by chromatography (petroleum ether/EtOAc = 1:1) afforded compound **3bb** (49.0 mg, 78% yield) as a white solid. *R_f_* = 0.37 (petroleum ether/EtOAc = 1:1). Mp 111.1–112.7 °C. ^1^H NMR (600 MHz, Chloroform-*d*) δ 7.88 (d, *J* = 8.9 Hz, 2H), 7.39–7.28 (m, 5H), 7.00 (d, *J* = 9.0 Hz, 1H), 6.89 (d, *J* = 8.9 Hz, 2H), 6.46 (d, *J* = 8.9 Hz, 1H), 4.76 (dd, 27.4, 11.8 Hz, 2H), 3.87 (s, 3H), 2.13 (s, 3H); ^13^C{^1^H} NMR (151 MHz, Chloroform-*d*) δ 190.3, 171.1, 164.4, 137.2, 131.9, 128.4, 128.3, 127.9, 126.5, 113.9, 76.0, 70.3, 55.5, 23.6. HRMS (ESI) *m*/*z*: [M + Na]^+^ calcd for C_18_H_19_NNaO_4_ 336.1206; found 336.1194.

#### *N*-(1-(Benzyloxy)-2-(2,5-dimethoxyphenyl)-2-oxoethyl)acetamide (**3bc**)

The general procedure was performed using *N*-(2-(2,5-dimethoxyphenyl)-2-oxoethyl)acetamide **1bc** (47.4 mg, 0.2 mmol) and benzyl alcohol **2a** (26.0 mg, 24.8 μL, 0.24 mmol). Purification by chromatography (petroleum ether/EtOAc = 1:1.5) afforded compound **3bc** (41.7 mg, 61% yield) as a white solid. *R_f_* = 0.33 (petroleum ether/EtOAc = 1:1.5). Mp 116.0–116.7 °C. ^1^H NMR (600 MHz, Chloroform-*d*) δ 7.29 (d, *J* = 3.3 Hz, 1H), 7.25–7.22 (m, 3H), 7.21–7.19 (m, 2H), 7.09 (dd, *J* = 9.0, 3.2 Hz, 1H), 6.89–6.85 (dd, *J* = 12.0, 8.9 Hz, 2H), 6.61 (d, *J* = 9.2 Hz, 1H), 4.87–4.78 (dd, *J* = 26.7, 11.7 Hz, 2H), 3.79 (s, 3H), 3.77 (s, 3H), 2.11 (s, 3H); ^13^C{^1^H} NMR (151 MHz, Chloroform-*d*) δ 193.7, 170.9, 153.7, 153.5, 137.9, 128.2, 127.7, 127.6, 124.8, 121.7, 114.8, 112.9, 80.1, 72.1, 56.0, 55.8, 23.6. HRMS (ESI) *m*/*z*: [M + Na]^+^ calcd for C_19_H_21_NNaO_5_ 366.1317; found 366.1322.

#### *N*-(1-(Benzyloxy)-2-(3-nitrophenyl)-2-oxoethyl)acetamide (**3bd**)

The general procedure was performed using *N*-(2-(3-nitrophenyl)-2-oxoethyl)acetamide **1bd** (44.4 mg, 0.2 mmol) and benzyl alcohol **2a** (26.0 mg, 24.8 μL, 0.24 mmol). Purification by chromatography (petroleum ether/EtOAc = 1:1) afforded compound **3bd** (45.3 mg, 69% yield) as a white solid. *R_f_* = 0.33 (petroleum ether/EtOAc = 1:1). Mp 147.8–148.2 °C. ^1^H NMR (600 MHz, Chloroform-*d*) δ 8.78 (s, 1H), 8.44–8.41 (m, 1H), 8.10 (d, *J* = 7.8 Hz, 1H), 7.60 (t, *J* = 8.0 Hz, 1H), 7.41–7.31 (m, 5H), 6.87 (d, *J* = 9.3 Hz, 1H), 6.52 (d, *J* = 9.1 Hz, 1H), 4.80 (dd, *J* = 23.3, 11.6 Hz, 2H), 2.17 (s, 3H); ^13^C{^1^H} NMR (151 MHz, Chloroform-*d*) δ 190.3, 171.1, 148.4, 136.4, 134.9, 134.8, 129.9, 128.7, 128.6, 128.4, 128.2, 124.3, 76.5, 71.0, 23.5. HRMS (ESI) *m*/*z*: [M + Na]^+^ calcd for C_17_H_16_N_2_NaO_5_ 351.0957; found 351.0954.

#### *N*-(1-(Benzyloxy)-2-(4-nitrophenyl)-2-oxoethyl)acetamide (**3be**)

The general procedure was performed using *N*-(2-(4-nitrophenyl)-2-oxoethyl)acetamide **1be** (44.4 mg, 0.2 mmol) and benzyl alcohol **2a** (26.0 mg, 24.8 μL, 0.24 mmol). Purification by chromatography (petroleum ether/EtOAc = 1:1.5) afforded compound **3be** (53.8 mg, 82% yield) as a white solid. *R_f_* = 0.43 (petroleum ether/EtOAc = 1:1.5). Mp 169.8–171.5 °C. ^1^H NMR (600 MHz, Chloroform-*d*) δ 8.21 (d, *J* = 8.9 Hz, 2H), 7.94 (d, *J* = 8.8 Hz, 2H), 7.41–7.36 (m, 5H), 6.85 (bs, 1H), 6.50 (d, *J* = 9.1 Hz, 1H), 4.78 (dd, *J* = 15.0, 11.7 Hz, 2H), 2.17 (s, 3H); ^13^C{^1^H} NMR (151 MHz, Chloroform-*d*) δ 190.8, 171.1, 150.7, 138.1, 136.3, 130.4, 128.8, 128.6, 128.5, 123.7, 76.2, 70.8, 23.5. HRMS (ESI) *m*/*z*: [M + Na]^+^ calcd for C_17_H_16_N_2_NaO_5_ 351.0957; found 351.0949.

#### *N*-(1-(Benzyloxy)-2-(naphthalen-1-yl)-2-oxoethyl)acetamide (**3bf**)

The general procedure was performed using *N*-(2-(naphthalen-1-yl)-2-oxoethyl)acetamide **1bf** (45.4 mg, 0.2 mmol) and benzyl alcohol **2a** (26.0 mg, 24.8 μL, 0.24 mmol). Purification by chromatography (petroleum ether/EtOAc = 1:1) afforded compound **3bf** (65.1 mg, 98% yield) as a white solid. *R_f_* = 0.37 (petroleum ether/EtOAc = 1:1). Mp 113.7–115.9 °C. ^1^H NMR (600 MHz, Chloroform-*d*) δ 8.75 (d, *J* = 8.6 Hz, 1H), 8.03 (d, *J* = 8.2 Hz, 1H), 7.89 (d, *J* = 8.1 Hz, 1H), 7.86 (d, *J* = 7.2 Hz, 1H), 7.64–7.54 (m, 1H), 7.56 (t, *J* = 7.5 Hz, 1H), 7.41 (t, *J* = 7.9 Hz, 1H), 7.28–7.24 (m, 5H), 7.01 (d, *J* = 9.0 Hz, 1H), 6.57 (d, *J* = 9.0 Hz, 1H), 4.76 (dd, *J* = 16.9, 11.7 Hz, 2H), 2.16 (s, 3H); ^13^C{^1^H} NMR (151 MHz, Chloroform-*d*) δ 194.8, 171.0, 136.9, 134.4, 133.9, 131.0, 131.0, 130.3, 128.6, 128.6, 128.4, 128.3, 127.9, 126.6, 125.6, 124.3, 77.4, 71.0, 23.6. HRMS (ESI) *m*/*z*: [M + Na]^+^ calcd for C_21_H_19_NNaO_3_ 356.1263; found 356.1255.

#### *N*-(1-(Benzyloxy)-2-(naphthalen-2-yl)-2-oxoethyl)acetamide (**3bg**)

The general procedure was performed using *N*-(2-(naphthalen-2-yl)-2-oxoethyl)acetamide **1bg** (45.4 mg, 0.2 mmol) and benzyl alcohol **2a** (26.0 mg, 24.8 μL, 0.24 mmol). Purification by chromatography (petroleum ether/EtOAc = 1:1) afforded compound **3bg** (63.2 mg, 95% yield) as a white solid. *R_f_* = 0.42 (petroleum ether/EtOAc = 1:1). Mp 116.8–117.7 °C. ^1^H NMR (600 MHz, Chloroform-*d*) δ 8.31 (s, 1H), 7.97 (dd, *J* = 8.7, 1.8 Hz, 1H), 7.86 (d, *J* = 8.5 Hz, 2H), 7.78 (d, *J* = 8.2 Hz, 1H), 7.63–7.60 (m, 1H), 7.55–7.54 (m, 1H), 7.44–7.42 (m, 2H), 7.38–7.34 (m, 3H), 7.04 (d, *J* = 9.1 Hz, 1H), 6.67 (d, *J* = 9.0 Hz, 1H), 4.82 (dd, *J* = 36.4, 11.7 Hz, 2H), 2.17 (s, 3H); ^13^C{^1^H} NMR (151 MHz, Chloroform-*d*) δ 191.9, 171.2, 137.0, 136.1, 132.3, 132.1, 130.8, 129.9, 129.1, 128.8, 128.6, 128.6, 128.2, 127.8, 126.9, 124.3, 75.8, 70.5, 23.6. HRMS (ESI) *m*/*z*: [M + Na]^+^ calcd for C_21_H_19_NNaO_3_ 356.1263; found 356.1258.

#### *N*-(1-(Benzyloxy)-2-oxo-2-(thiophen-2-yl)ethyl)acetamide (**3bh**)

The general procedure was performed using *N*-(2-oxo-2-(thiophen-2-yl)ethyl)acetamide **1bh** (36.6 mg, 0.2 mmol) and benzyl alcohol **2a** (26.0 mg, 24.8 μL, 0.24 mmol). Purification by chromatography (petroleum ether/EtOAc = 1.5:1) afforded compound **3bh** (53.6 mg, 93% yield) as a white solid. *R_f_* = 0.43 (petroleum ether/EtOAc = 1:1). Mp 100.1–104.7 °C. ^1^H NMR (600 MHz, Chloroform-*d*) δ 7.71 (d, *J* = 4.9 Hz, 1H), 7.67 (d, *J* = 2.9 Hz, 1H), 7.39 (d, *J* = 7.0 Hz, 2H), 7.36–7.28 (m, 3H), 7.10 (t, *J* = 4.4 Hz, 1H), 6.92 (d, *J* = 8.6 Hz, 1H), 6.36 (d, *J* = 9.1 Hz, 1H), 4.78 (dd, *J* = 16.1, 10.2 Hz, 2H), 2.12 (s, 3H); ^13^C{^1^H} NMR (151 MHz, Chloroform-*d*) δ 185.2, 171.0, 140.2, 137.0, 135.6, 134.7, 128.4, 128.4, 128.3, 128.0, 77.5, 70.7, 23.5. HRMS (ESI) *m*/*z*: [M + Na]^+^ calcd for C_15_H_15_NNaO_3_S 312.0670; found 312.0678.

#### *N*-(1-(Benzyloxy)-2-oxopropyl)benzamide (**3bi**)

The general procedure was performed using *N*-(2-oxopropyl)benzamide **1bi** (35.4 mg, 0.2 mmol) and benzyl alcohol **2a** (26.0 mg, 24.8 μL, 0.24 mmol). Purification by chromatography (petroleum ether/EtOAc = 2.5:1) afforded compound **3bi** (46.6 mg, 82% yield) as a colorless oil. *R_f_* = 0.44 (petroleum ether/EtOAc = 2:1). ^1^H NMR (600 MHz, Chloroform-*d*) δ 7.86–7.84 (m, 2H), 7.55 (t, *J* = 7.4 Hz, 1H), 7.46 (t, *J* = 7.7 Hz, 2H), 7.43 (d, *J* = 7.3 Hz, 2H), 7.39–7.34 (m, 2H), 7.34–7.30 (m, 2H), 5.83 (d, *J* = 8.5 Hz, 1H), 4.83 (dd, *J* = 23.4, 11.7 Hz, 2H), 2.30 (s, 3H); ^13^C{^1^H} NMR (151 MHz, Chloroform-*d*) δ 201.7, 168.1, 137.1, 133.3, 132.2, 128.7, 128.5, 128.3, 128.1, 127.2, 80.5, 71.7, 26.6. HRMS (ESI) *m*/*z*: [M + Na]^+^ calcd for C_17_H_17_NNaO_3_ 306.1106; found 306.1120.

#### *N*-(1-(Benzyloxy)-2-oxo-4-phenylbutyl)acetamide (**3bj**)

The general procedure was performed using *N*-(2-oxo-4-phenylbutyl)acetamide **1bj** (41.0 mg, 0.2 mmol) and benzyl alcohol **2a** (26.0 mg, 24.8 μL, 0.24 mmol). Purification by chromatography (petroleum ether/EtOAc = 1:1) afforded compound **3bj** (43.1 mg, 69% yield) as a white solid. *R_f_* = 0.33 (petroleum ether/EtOAc = 1:1). Mp 86.0–88.1 °C. ^1^H NMR (600 MHz, Chloroform-*d*) δ 7.37–7.34 (m, 4H), 7.32–7.29 (m, 1H), 7.27 (t, *J* = 7.3 Hz, 2H), 7.20 (t, *J* = 7.4 Hz, 1H), 7.13 (d, *J* = 7.3 Hz, 2H), 6.50 (d, *J* = 8.9 Hz, 1H), 5.60 (d, *J* = 8.8 Hz, 1H), 4.72 (dd, *J* = 32.5, 11.7 Hz, 2H), 3.12–3.06 (m, 1H), 2.88 (t, *J* = 7.5 Hz, 2H), 2.77–2.72 (m, 1H), 2.07 (s, 3H); ^13^C{^1^H} NMR (151 MHz, Chloroform-*d*) δ 203.0, 171.1, 140.4, 137.1, 128.5, 128.5, 128.3, 128.2, 128.1, 126.3, 79.8, 71.6, 40.6, 29.2, 23.4. HRMS (ESI) *m*/*z*: [M + Na]^+^ calcd for C_19_H_21_NNaO_3_ 334.1419; found 334.1413.

#### *N*-(1-(Benzyloxy)-2-oxo-2-phenylethyl)-*N*-methylacetamide (**3bk**)

The general procedure was performed using *N*-methyl-*N*-(2-oxo-2-phenylethyl)acetamide **1bk** (38.2 mg, 0.2 mmol) and benzyl alcohol **2a** (26.0 mg, 24.8 μL, 0.24 mmol). Purification by chromatography (petroleum ether/EtOAc = 2:1) afforded compound **3bk** (48.4 mg, 81% yield) as a pale yellow oil. *R_f_* = 0.29 (petroleum ether/EtOAc = 2:1). ^1^H NMR (600 MHz, Chloroform-*d*) δ 7.95 (d, *J* = 7.3 Hz, 2H), 7.56 (t, *J* = 7.4 Hz, 1H), 7.44–7.40 (m, 4H), 7.36 (t, *J* = 7.4 Hz, 2H), 7.33–7.29 (m, 1H), 7.06 (s, 1H), 4.66 (dd, *J* = 30.4, 12.0 Hz, 2H), 2.81 (s, 3H), 2.07 (s, 3H); ^13^C{^1^H} NMR (151 MHz, Chloroform-*d*) δ 193.1, 172.2, 136.8, 134.2, 133.8, 128.8, 128.6, 128.4, 128.2, 128.1, 81.1, 70.9, 30.4, 22.0. HRMS (ESI) *m*/*z*: [M + Na]^+^ calcd for C_18_H_19_NNaO_3_ 320.1263; found 320.1268.

#### *N*-(1-((4-Chlorobenzyl)thio)-2-oxo-2-phenylethyl)benzamide (**4a**)

The general procedure was performed using *N*-(2-oxo-2-phenylethyl)acetamide **1d** (47.8 mg, 0.2 mmol) and (4-chlorophenyl)methanethiol (38.1 mg, 31.7 μL, 0.24 mmol). Purification by chromatography (petroleum ether/EtOAc = 6:1) afforded compound **4a** (29.3 mg, 37% yield) as a white solid. *R_f_* = 0.33 (petroleum ether/EtOAc = 6:1). Mp 123.6–125.7 °C. ^1^H NMR (600 MHz, Chloroform-*d*) δ 7.84 (d, *J* = 7.0 Hz, 2H), 7.67 (d, *J* = 7.0 Hz, 2H), 7.60 (t, *J* = 7.4 Hz, 1H), 7.54 (t, *J* = 7.4 Hz, 1H), 7.47–7.41 (m, 4H), 7.38 (s, 1H), 7.36 (d, *J* = 8.3 Hz, 2H), 7.25 (d, *J* = 8.0 Hz, 2H), 6.70 (d, *J* = 8.2 Hz, 1H), 3.88 (dd, *J* = 131.0, 13.9 Hz, 2H); ^13^C{^1^H} NMR (151 MHz, Chloroform-*d*) δ 190.9, 166.3, 136.4, 134.2, 133.2, 133.1, 132.1, 130.7, 128.9, 128.9, 128.8, 128.6, 127.0, 55.6, 34.5. HRMS (ESI) *m*/*z*: [M + Na]^+^ calcd for C_22_H_18_ClNNaO_2_S 418.0644; found 418.0642.

#### *N*-(2-Oxo-2-phenyl-1-((4-(trifluoromethyl)phenyl)thio)ethyl)benzamide (**4b**)

The general procedure was performed using *N*-(2-oxo-2-phenylethyl)acetamide **1d** (47.8 mg, 0.2 mmol) and 4-mercaptobenzotrifluoride (42.8 mg, 32.9 μL, 0.24 mmol). Purification by chromatography (petroleum ether/EtOAc = 4:1) afforded compound **4b** (22.5 mg, 27% yield) as a white solid. *R_f_* = 0.35 (petroleum ether/EtOAc = 4:1). Mp 108.4–112.9 °C. ^1^H NMR (600 MHz, Chloroform-*d*) δ 7.99 (d, *J* = 6.9 Hz, 2H), 7.81 (d, *J* = 7.0 Hz, 2H), 7.66 (t, *J* = 7.4 Hz, 1H), 7.59–7.47 (m, 10H), 6.86 (d, *J* = 7.9 Hz, 1H); ^13^C{^1^H} NMR (151 MHz, Chloroform-*d*) δ 189.9, 165.9, 135.7, 134.6, 134.4, 133.2, 133.1, 132.4, 131.5 (q, *J*_*C–F*_ = 32.9 Hz), 129.0, 128.9, 128.8, 127.1, 125.9 (d, *J*_*C–F*_ = 3.6 Hz), 125.9 (d, *J*_*C–F*_ = 3.8 Hz), 58.4; ^19^F NMR (376 MHz, Chloroform-*d*) δ −62.82. HRMS (ESI) *m*/*z*: [M + Na]^+^ calcd for C_22_H_16_F_3_NNaO_2_S 438.0752; found 438.0749.

#### *N*-(1-(6-Chloro-1*H*-benzo[*d*][1,2,3]triazol-1-yl)-2-oxo-2-phenylethyl)benzamide (**4c**)

The general procedure was performed using *N*-(2-oxo-2-phenylethyl)acetamide **1d** (47.8 mg, 0.2 mmol) and 6-chloro-1*H*-benzo[*d*][1,2,3]triazole **2a** (36.7 mg, 0.24 mmol, 1.2 equiv). Purification by chromatography (petroleum ether/EtOAc = 3:1) afforded positional isomers **4c** (36.9 mg, 47% yield) as a white solid at a ratio of 1:1 (determined by crude ^1^H NMR). *R_f_* = 0.37 (petroleum ether/EtOAc = 3:1). Mp 141.7–143.4 °C. ^1^H NMR (600 MHz, Chloroform-*d*) δ 8.36–8.34 (m, 1H), 8.17–8.12 (m, 2H), 8.00–7.86 (m, 5H), 7,59–7.53 (m, 3H), 7.49–7.45 (m, 2H), 7.40–7.36 (m, 2H); ^13^C{^1^H} NMR (151 MHz, Chloroform-*d*) δ 188.0 (r1), 188.0 (r2), 167.3 (r1), 167.3 (r2), 146.5 (r1), 144.5 (r2), 135.2 (r1), 134.9, 132.9 (r2), 132.8 (r1), 132.7 (r2), 132.6 (r1), 132.6 (r2), 132.3 (r1), 132.2 (r2), 131.0 (r1), 130.6 (r2), 129.6, 129.2 (r1), 129.2 (r2), 128.8 (r1), 128.8 (r2), 127.4 (r1), 127.4 (r2), 126.0, 121.1 (r1), 119.5 (r2), 111.5 (r1), 110.3 (r2), 62.9. HRMS (ESI) *m*/*z*: [M + Na]^+^ calcd for C_21_H_15_ClN_4_NaO_2_ 413.0781; found 413.0775.

#### *N*-(2-Phenylnaphtho[2,1-*b*]furan-1-yl)benzamide (**4d**)

The general procedure was performed using *N*-(2-oxo-2-phenylethyl)acetamide **1d** (47.8 mg, 0.2 mmol) and 2-naphthol (34.6 mg, 0.24 mmol, 1.2 equiv). Purification by chromatography (petroleum ether/EtOAc = 4:1) afforded compound **4d** (66.0 mg, 91% yield) as a white solid. *R_f_* = 0.33 (petroleum ether/EtOAc = 4:1). ^1^H NMR (600 MHz, DMSO-*d*_6_) δ 10.75 (s, 1H), 8.24 (d, *J* = 7.9 Hz, 1H), 8.19 (d, *J* = 7.1 Hz, 2H), 8.08 (d, *J* = 7.1 Hz, 1H), 7.96 (d, *J* = 8.0 Hz, 2H), 7.94–7.89 (m, 2H), 7.71 (t, *J* = 7.4 Hz, 1H), 7.65 (t, *J* = 7.4 Hz, 2H), 7.56–7.51 (m, 4H), 7.41 (t, *J* = 7.4 Hz, 1H); ^13^C{^1^H} NMR (151 MHz, DMSO-*d*_6_) δ 167.2, 150.8, 149.1, 134.1, 132.7, 130.9, 129.8, 129.5, 129.4, 129.4, 129.2, 128.2, 127.7, 127.2, 126.8, 125.8, 125.4, 122.7, 121.4, 117.3, 113.0. The spectral characteristics are in agreement with spectral data previously reported.^23^ HRMS (ESI) *m*/*z* calcd for C_25_H_18_NO_2_ (M + H)^+^: 364.1338; found: 364.1349.

### Gram Scale

A 100 mL round-bottom flask was charged with *N*-(2-oxo-2-phenylethyl)benzamide **1d** (10 mmol, 2.4 g), benzyl alcohol **2a** (12 mmol, 1.3 g, 1.2 equiv), I_2_ (508 mg, 2 mmol, 20 mol %), and DMSO (15 mmol, 1.2 g, 1.5 equiv), followed by the addition of anhydrous CHCl_3_ (50 mL). The reaction mixture was heated to reflux for 20 h under air in an oil bath. When the reaction was completed, the crude reaction mixture was allowed to reach room temperature. 94% NMR yield was determined with dibromomethane as internal standard. The solution was then quenched with 10% Na_2_S_2_O_3_ (30 mL) solution (w/w) and extracted with CH_2_Cl_2_ (3 × 30 mL). The combined organic layers were dried over Na_2_SO_4_, filtered, and concentrated in vacuo. The crude product was purified by crystallization with ethyl acetate and petroleum ether to afford the product **3d** in 81% isolated yield (2.8 g).

#### Transformation of Product **3e**

##### General Procedure A

A screw-capped vial was charged with **3e** (53.8 mg, 0.2 mmol), nucleophile (0.22 mol, 1.1 equiv), and methanesulfonic acid (1.3 μL, 0.02 mmol, 0.1 equiv), followed by the addition of anhydrous CHCl_3_ (1 mL). The vial was tightly capped and stirred at 80 °C for 12 h in an oil bath. The crude mixture was directly purified by silica gel chromatography to give the title product.

##### *N*-(5-Methoxy-2-phenylbenzofuran-3-yl)benzamide (**8a**)

The general procedure A was performed using 4-methoxyphenol (27.3 mg, 0.22 mol, 1.1 equiv). Purification by chromatography (CH_2_Cl_2_/EtOAc = 8:1) afforded compound **8a** (65.7 mg, 96%) as a white solid. *R_f_* = 0.23 (CH_2_Cl_2_/EtOAc = 8:1). Mp 217.8–219.5 °C. ^1^H NMR (600 MHz, Chloroform-*d*) δ 7.99 (d, *J* = 7.6 Hz, 2H), 7.90–7.79 (m, 2H), 7.70 (s, 1H), 7.62 (t, *J* = 7.4 Hz, 1H), 7.54 (t, *J* = 7.6 Hz, 2H), 7.44 (t, *J* = 7.8 Hz, 2H), 7.39 (d, *J* = 8.9 Hz, 1H), 7.35 (t, *J* = 7.4 Hz, 1H), 6.97 (d, *J* = 2.5 Hz, 1H), 6.93 (dd, *J* = 8.9, 2.6 Hz, 1H), 3.82 (s, 3H); ^13^C{^1^H} NMR (151 MHz, Chloroform-*d*) δ 166.2, 156.2, 148.8, 148.1, 133.7, 132.3, 129.8, 128.9, 129.90, 128.7, 127.5, 127.1, 126.1, 114.5, 114.4, 112.1, 102.2, 55.9. HRMS (ESI) *m*/*z* calcd for C_22_H_18_NO_3_ (M + H)^+^: 344.1287; found: 344.1300.

##### Ethyl 3-(1-Benzamido-2-oxo-2-phenylethyl)-1*H*-indole-2-carboxylate (**8b**)

The general procedure A was performed using ethyl 1*H*-indole-2-carboxylate (41.6 mg, 0.22 mol, 1.1 equiv). Purification by chromatography (petroleum ether/EtOAc = 3:2) afforded compound **8b** (48.7 mg, 57%) as a white solid. *R_f_* = 0.30 (petroleum ether/EtOAc = 3:2). Mp 232.0–235.1 °C. ^1^H NMR (600 MHz, Chloroform-*d*) δ 9.28 (s, 1H), 8.17–8.15 (m, 3H), 7.86–7.84 (m, 3H), 7.76 (d, *J* = 6.8 Hz, 1H), 7.49–7.39 (m, 5H), 7.32 (t, *J* = 7.8 Hz, 2H), 7.29 (t, *J* = 7.6 Hz, 1H), 7.16 (t, *J* = 7.6 Hz, 1H), 4.31 (dq, *J* = 10.9, 7.1 Hz, 1H), 4.14 (dq, *J* = 10.8, 7.2 Hz, 1H), 1.29 (t, *J* = 7.1 Hz, 3H); ^13^C{^1^H} NMR (151 MHz, Chloroform-*d*) δ 195.8, 166.4, 161.2, 135.8, 134.7, 134.2, 133.5, 131.6, 128.9, 128.5, 128.5, 127.2, 126.0, 125.6, 124.7, 121.3, 120.9, 117.4, 112.4, 61.3, 52.0, 14.3. HRMS (ESI) *m*/*z* calcd for C_26_H_23_N_2_O_4_ (M + H)^+^: 427.1658; found: 427.1654.

##### *N*-(1-Benzamido-2-oxo-2-phenylethyl)-4-fluorobenzamide (**8c**)

The general procedure A was performed using 4-fluorobenzamide (30.6 mg, 0.22 mol, 1.1 equiv). Purification by chromatography (CH_2_Cl_2_/EtOAc = 8:1) afforded compound **8c** (47.8 mg, 64%) as a white solid. *R_f_* = 0.48 (CH_2_Cl_2_/EtOAc = 8:1). Mp 213.4–216.7 °C. ^1^H NMR (600 MHz, DMSO-*d*_6_) δ 9.31–9.23 (m, 2H), 8.01–7.96 (m, 4H), 7.89–7.88 (m, 2H), 7.63 (t, *J* = 7.4 Hz, 1H), 7.57–7.47 (m, 3H), 7.48 (t, *J* = 7.7 Hz, 3H), 7.32 (t, *J* = 8.8 Hz, 2H), 7.01 (t, *J* = 7.4 Hz, 1H); ^13^C{^1^H} NMR (151 MHz, DMSO-*d*_6_) δ 192.9, 166.5, 165.5 (d, *J* = 3.1 Hz), 163.8, 134.8, 134.0, 133.7, 132.3, 130.8 (d, *J* = 9.0 Hz), 130.2 (d, *J* = 2.8 Hz), 129.2, 128.9, 128.6, 128.0, 115.8 (d, *J* = 21.6 Hz), 60.21; ^19^F NMR (376 MHz, DMSO-*d*_6_) δ −108.43. HRMS (ESI) *m*/*z* calcd for C_22_H_18_FN_2_O_3_ (M + H)^+^: 377.1301; found: 377.1311.

##### *N*-(1-(2-Methoxynaphthalen-1-yl)-2-oxo-2-phenylethyl)benzamide (**8d**)

The general procedure A was performed using 2-methoxynaphthalene (34.8 mg, 0.22 mol, 1.1 equiv). Purification by chromatography (petroleum ether/EtOAc = 3:1) afforded compound **8d** (36.1 mg, 46%) as a white solid. *R_f_* = 0.28 (petroleum ether/EtOAc = 3:1). Mp 71.0–74.2 °C. ^1^H NMR (400 MHz, Chloroform-*d*) δ 8.57 (d, *J* = 8.7 Hz, 1H), 7.90–7.75 (m, 6H), 7.66 (t, *J* = 7.9 Hz, 1H), 7.58 (d, *J* = 8.0 Hz, 1H), 7.51–7.31 (m, 6H), 7.23–7.10 (m, 3H), 3.83 (s, 3H); ^13^C{^1^H} NMR (101 MHz, Chloroform-*d*) δ 197.1, 167.0, 155.2, 135.0, 134.3, 132.8, 132.2, 131.5, 131.0, 129.6, 128.8, 128.4, 128.2, 128.1, 128.0, 127.3, 124.0, 123.0, 119.5, 113.3, 56.3, 53.6. HRMS (ESI) *m*/*z* calcd for C_26_H_22_NO_3_ (M + H)^+^: 396.1600; found 396.1610.

##### *N*-(1-Chloro-2-oxo-2-phenylethyl)benzamide (**8e**)

**3d** (53.8 mg, 0.2 mmol) and POCl_3_ (0.5 mL) were charged into a test tube, stirred for 12 h at room temperature, and then cooled to 0 °C. The resulting mixture was diluted with sat. aq NaHCO_3_ (10 mL) and extracted with CH_2_Cl_2_ (2 × 10 mL). The combined organic layers were washed with sat. aq NaHCO_3_ (10 mL) and sat. aq NaCl (10 mL) and dried over Na_2_SO_4_. Filtration and removal of the solvent under reduced pressure produced the crude product. Purification by silica gel chromatography (petroleum ether/ethyl acetate = 1.5:1) provided the title compound **8e** as a white solid (47.3 mg, 87%). *R_f_* = 0.43 (petroleum ether/EtOAc = 1:1). Mp 143.3–146.4 °C. ^1^H NMR (600 MHz, Chloroform-*d*) δ 8.19–8.17 (m, 2H), 7.84–7.82 (m, 2H), 7.65–7.61 (m, 2H), 7.56–7.52 (m, 1H), 7.52–7.50 (m, 2H), 7.47–7.44 (m, 2H), 6.73 (d, *J* = 7.8 Hz, 1H); ^13^C{^1^H} NMR (151 MHz, Chloroform-*d*) δ 194.0, 167.8, 134.6, 133.1, 132.7, 132.4, 129.6, 128.9, 128.7, 127.3, 73.1. HRMS (ESI) *m*/*z*: [M – Cl + OH + Na]^+^ calcd for C_15_H_13_NNaO_3_ 278.0793; found 278.0800.

##### 4-Chloro-2,5-diphenyloxazole (**8f**)

A screw-capped vial was charged with **3d** (53.8 mg, 0.2 mmol) and POCl_3_ (0.5 mL), The vial was tightly capped and stirred at 80 °C for 10 h in an oil bath. The resulting mixture was cooled to 0 °C, diluted with sat. aq NaHCO_3_ (10 mL), and extracted with CH_2_Cl_2_ (2 × 10 mL). The combined organic layers were washed with sat. aq NaHCO_3_ (10 mL) and sat. aq NaCl (10 mL) and dried over Na_2_SO_4_. Filtration and removal of the solvent under reduced pressure produced the crude product. Purification by silica gel chromatography (petroleum ether/ethyl acetate = 20:1) provided the title compound **8f** as a pale yellow solid (37.6 mg, 73%). *R_f_* = 0.46 (petroleum ether/EtOAc = 20:1). Mp 149.0–152.3 °C. ^1^H NMR (600 MHz, Chloroform-*d*) δ 8.10–8.08 (m, 2H), 7.95 (d, *J* = 8.3 Hz, 2H), 7.51–7.47 (m, 5H), 7.38 (t, *J* = 7.4 Hz, 1H); ^13^C{^1^H} NMR (151 MHz, Chloroform-*d*) δ 159.0, 143.9, 1301.0, 129.0, 128.8, 128.7, 126.9, 126.4, 126.4, 126.3, 125.0. HRMS (ESI) *m*/*z* calcd for C_15_H_11_ClNO (M + H)^+^: 256.0529; found: 256.0546.

##### 4-Methoxy-2,5-diphenyloxazole (**8g**)

A screw-capped vial was charged with **1d** (53.8 mg, 0.2 mmol), triphenylphosphine (157.4 mg, 0.6 mmol, 3.0 equiv), I_2_ (152.3 mg, 0.6 mmol, 3.0 equiv), and triethylamine (121.4 mg, 167 μL, 1.2 mmol, 6.0 equiv), followed by the addition of anhydrous CH_2_Cl_2_ (1 mL). The vial was tightly capped and stirred at room temperature for 12 h. When the reaction was completed, the solution was quenched with 10% Na_2_S_2_O_3_ (10 mL) solution (w/w) and extracted with CH_2_Cl_2_ (2 × 10 mL). The combined organic layers were dried over Na_2_SO_4_, filtered, and concentrated in vacuo. Purification by silica gel chromatography (petroleum ether/ethyl acetate = 100:1) provided the title compound **8g** as a pale yellow solid (48.2 mg, 96%). *R_f_* = 0.45 (petroleum ether/EtOAc = 100:1). Mp 65.3–65.7 °C. ^1^H NMR (600 MHz, Chloroform-*d*) δ 8.06 (d, *J* = 6.9 Hz, 2H), 7.78 (d, *J* = 7.9 Hz, 2H), 7.48–7.43 (m, 3H), 7.41 (t, *J* = 7.7 Hz, 2H), 7.21 (t, *J* = 7.4 Hz, 1H), 4.13 (s, 3H); ^13^C{^1^H} NMR (151 MHz, Chloroform-*d*) δ 155.9, 151.9, 130.2, 130.1, 128.8, 128.7, 128.6, 127.5, 126.2, 126.0, 123.0, 57.2. HRMS (ESI) *m*/*z* calcd for C_16_H_14_NO_2_ (M + H)^+^: 252.1025; found: 252.1036.

##### 4-Methoxy-2-phenyl-5-phenylthiazole (**8h**)

Lawesson’s reagent (161.8 mg, 0.4 mmol, 2.0 equiv) was added to a solution of **1d** (53.8 mg, 0.2 mmol) in toluene (2 mL). The mixture was heated to 80 °C under N_2_ for 8 h in an oil bath. The toluene was removed under reduced pressure, and the residue was purified by silica gel chromatography (petroleum ether/ethyl acetate = 30:1) to give a light yellow solid **8h** (39.7 mg, 97%). *R_f_* = 0.48 (petroleum ether/EtOAc = 30:1). Mp 43.5–46.9 °C. ^1^H NMR (600 MHz, Chloroform-*d*) δ 7.93 (d, *J* = 6.7 Hz, 2H), 7.73 (d, *J* = 7.8 Hz, 2H), 7.44–7.39 (m, 3H), 7.37 (t, *J* = 7.8 Hz, 2H), 7.22 (t, *J* = 7.4 Hz, 1H), 4.18 (s, 3H); ^13^C{^1^H} NMR (151 MHz, Chloroform-*d*) δ 160.2, 159.4, 133.7, 131.7, 129.8, 128.9, 128.6, 126.7, 126.4, 125.5, 111.2, 57.7. HRMS (ESI) *m*/*z*: [M + H]^+^ calcd for C_16_H_14_NOS 268.0796; found 268.0799.

##### *N*-(1-Acetamido-2-oxo-2-phenylethyl)benzamide (**8i**)

A screw-capped vial was charged with **1d** (53.8 mg, 0.2 mmol) and MeCN (1 mL), followed by the addition of methanesulfonic acid (65 μL, 1 mmol, 5 equiv). The vial was tightly capped and stirred at 50 °C for 2 h in an oil bath. The resulting mixture was diluted with sat. aq NaHCO_3_ (10 mL) and extracted with CH_2_Cl_2_ (2 × 10 mL). The combined organic layers were washed with sat. aq NaCl (10 mL) and dried over Na_2_SO_4_. Filtration and removal of the solvent under reduced pressure produced the crude product. Purification by silica gel chromatography (CH_2_Cl_2_/EtOAc = 1:1) provided the title compound **8i** as a white solid (32.6 mg, 55%). *R_f_* = 0.38 (CH_2_Cl_2_/EtOAc = 1:1). Mp 171.2–173.4 °C. ^1^H NMR (600 MHz, DMSO-*d*_6_) δ 9.40 (d, *J* = 7.1 Hz, 1H), 8.74 (d, *J* = 8.0 Hz, 1H), 7.94 (d, *J* = 7.0 Hz, 2H), 7.82 (d, *J* = 7.0 Hz, 2H), 7.62 (t, *J* = 7.4 Hz, 1H), 7.55–7.50 (m, 3H), 7.45 (t, *J* = 7.7 Hz, 2H), 6.71 (t, *J* = 7.5 Hz, 1H), 1.92 (s, 3H); ^13^C{^1^H} NMR (151 MHz, DMSO-*d*_6_) δ 193.5, 169.9, 166.4, 134.9, 133.8, 133.6, 132.3, 129.1, 128.8, 128.6, 127.9, 59.4, 22.9. HRMS (ESI) *m*/*z* calcd for C_17_H_16_N_2_NaO_3_ (M + H)^+^: 319.1059; found: 319.1063.

## Data Availability

The data underlying this study are available in the published article and it is Supporting Information.

## Supporting Information Available

Experimental procedures, characterization data, and copies of NMR spectra (PDF)
