# Supplementary material for: Oxidative Cross-Coupling of α-Amino Ketones with Alcohols Enabled by I2-Catalyzed C–H Hydroxylation
Source: J Org Chem. 2023 Oct 9;88(20):14470–86. doi: 10.1021/acs.joc.3c01469 (PMC10594655; doi:10.1021/acs.joc.3c01469)

Supporting Information

Oxidative Cross-Coupling of α-Amino Ketones with Alcohols Enabled by I_2-Catalyzed C-H Hydroxylation

Yingwei Wang,[a,b] Mingrong Yang, [a] Chichou Lao, [a] Hanxuan Wang, [a] and Zhihong Jiang*[a]

[a]State Key Laboratory of Quality Research in Chinese Medicines, Macau University of Science and Technology, Macau 999078, China

[b]School of Chemical Engineering, Sichuan University of Science & Engineering, Zigong 643000 China

Corresponding Author
*E-mail: zhjiang@must.edu.mo

Table of Contents

1. Preparation of Starting Materials S2

1.1 Preparation of Alcohols S2

1.2 Preparation of α-Amino Ketones S3

2. Unsuccessful Carbonyl Compounds for Oxidative Cross-Coupling with Benzyl Alcohol S9

3. Mechanistic Studies S11

4. References S14

5. Spectra S15

1. Preparation of Starting Materials

1.1 Preparation of Alcohols

Unless otherwise noted, alcohols were purchased from Shanghai Xian-Ding and used as received.

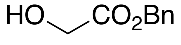

benzyl 2-hydroxyacetate (**2q**): is known compound and was synthesized according to the related literature.[1]

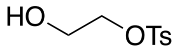

2-hydroxyethyl 4-methylbenzenesulfonate (**2r**): is known compound and was synthesized according to the related literature.[2]

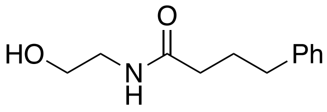

N-(2-hydroxyethyl)-4-phenylbutanamide (**2s**): A solution of 4-phenylbutanoyl chloride (2.0 mmol, 364 mg) in anhydrous CH_2Cl_2 (5 mL) was added dropwise to a mixture ethanolamine (2.0 mmol, 122 mg, 1.0 equiv) and triethylamine (4.0 mmol, 405 mg, 2.0 equiv.) at 0 °C. Stirring was continued while warming to room temperature over a period of 2 h. The solvent was evaporated in vacuo and the crude residue was purified by column chromatography (EtOAc) to afford the product **2s** (229.4 mg, 55%) as a white solid. ^{1}H NMR (600 MHz, Chloroform-*d*) δ 7.28 – 7.26 (m, 2H), 7.19 – 7.15 (m, 3H), 6.21 (s, 1H), 3.66 (t, *J* = 4.9 Hz, 2H), 3.45 – 3.35 (m, 3H), 2.64 (t, *J* = 7.6 Hz, 2H), 2.18 (t, *J* = 7.6 Hz, 2H), 1.95 (p, *J* = 7.6 Hz, 3H); ^{13}C{^{1}H} NMR (151 MHz, Chloroform-*d*) δ 174.0, 141.3, 128.4, 128.3, 125.9, 62.0, 42.3, 35.7, 35.1, 27.0. The spectral characteristics are in agreement with spectral data previously reported.[3]

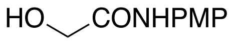

2-hydroxy-*N*-(4-methoxyphenyl)acetamide (**2t**): To a flame-dried round-bottomed flask were added *p*-anisidine (246.3 mg, 2 mmol), glycolic acid (182.5 mg, 2.4 mol, 1.2 equiv.), 3-nitrophenylboronic acid (66.8 mg, 0.4 mol, 20 mol%) and $CHCl_3$ (5 mL). The mixture was refluxed for 10 h, cooled to room temperature and directly subjected to column chromatography to afford the compound **2t** (165.5 mg, 46%) as a dark purple solid. ^{1}H NMR (600 MHz, Chloroform-*d*) δ 8.39 (s, 1H), 7.41 (d, *J* = 9.0 Hz, 2H), 6.85 (d, *J* = 9.0 Hz, 2H), 4.15 (s, 2H), 3.77 (s, 3H); ^{13}C{^{1}H} NMR (151 MHz, Chloroform-*d*) δ 170.0, 156.7, 129.9, 121.8, 114.2, 62.3, 55.4. The spectral characteristics are in agreement with spectral data previously reported.[4]

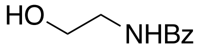

N-(2-hydroxyethyl)benzamide (**2u**): is known compound and was synthesized according to the related literature.[5]

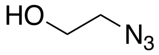

2-azidoethan-1-ol (**2v**): is known compound and was synthesized according to the related literature.[6]

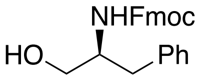

(9*H*-fluoren-9-yl)methyl (*S*)-(1-hydroxy-3-phenylpropan-2-yl)carbamate (**2ab**): is known compound and was synthesized according to the related literature.[7]

1.2 Preparation of α-Amino Ketones

Unless otherwise specified, α-amino ketones are known compounds and were synthesized according to the related literature.[8]

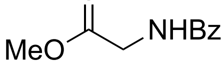

methyl benzoylglycinate (**1a**): is known compound and was synthesized according to the related literature.[9]

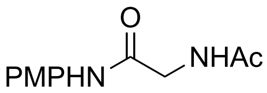

2-acetamido-*N*-(4-methoxyphenyl)acetamide (**1b**): EDCI.HCl (1.15 g, 6 mmol, 2.0 equiv) was added to a mixture of *N*-acetylglycine (351 mg, 3 mmol), *p*-anisidine (369 mg, 3 mmol, 1.0 equiv.) and HOBt (811 mg, 6 mmol, 2.0 equiv.) in 10 mL of THF. The reaction mixture was stirred for 12 h. After this time, the mixture was diluted with EtOAc (30 mL), The organic layer was washed with 2N HCl (2*20 mL) and with sat. $NaHCO_3$ (20 mL), dried over Na_2SO_4. Filtration and removal of the solvent under reduced pressure produced the crude product that could be purified by silica gel chromatography to (CH_2Cl_2: methanol = 30:1) afford the product as a light purple solid (580 mg, 87%).

Rf = 0.38 (CH_2Cl_2: methanol = 8:1)

m.p. 192.1 - 195.2 °C

^{1}H NMR (600 MHz, DMSO-d_6) δ 9.80 (s, 1H), 8.17 (t, J = 5.8 Hz, 1H), 7.48 (d, J = 9.0 Hz, 2H), 6.87 (d, J = 9.0 Hz, 2H), 3.83 (d, J = 5.8 Hz, 2H), 3.71 (s, 3H), 1.88 (s, 3H); ^{13}C{^{1}H} NMR (151 MHz, DMSO-d_6) δ 170.2, 167.9, 155.7, 132.5, 121.2, 114.4, 55.6, 43.1, 23.0.

HRMS (ESI) m/z: $[M+H]^+$ calcd for $C_{11}H_{14}N_2NaO_3$ 245.0902; found 245.0903.

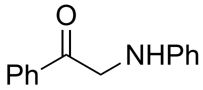

1-phenyl-2-(phenylamino)ethan-1-one (**1c**): s known compound and was synthesized according to the related literature.[10]

General procedure: CH_2Cl_2 (3 ml) and sat. $NaHCO_3$ (6 mL) were stirred vigorously and chilled in an ice bath. Acyl chloride or sulfonyl chloride (2.6 mmol, 1.3 equiv.) was added, followed immediately by 2-aminoketone hydrochloride (2 mmol). Stirring was continued while warming to room temperature over a period of 2 h. The mixture was extracted with CH_2Cl_2 (3*5 mL), the combined organic layers washed with sat. NaCl (10 mL) and dried over Na_2SO_4. Filtration and removal of the solvent under reduced

pressure produced the crude product that could be purified by silica gel chromatography or crystallization.

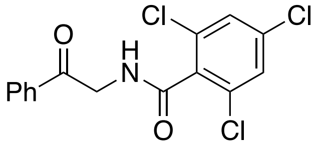

2,4,6-trichloro-*N*-(2-oxo-2-phenylethyl)benzamide (**1ai**): Following the general procedure , purification by crystallization with ethyl acetate and petroleum ether afforded the product **1ai** (321.2 mg, 47%) as a white solid.

Rf = 0.59 (petroleum ether: EtOAc = 2:1)

m.p. 160.0 - 162.1 °C

^{1}H NMR (600 MHz, Chloroform-*d*) δ 8.02 (d, *J* = 7.2 Hz, 2H), 7.66 (t, *J* = 7.4 Hz, 1H), 7.54 (t, *J* = 7.8 Hz, 2H), 7.38 (s, 2H), 6.96 (s, 1H), 5.00 (d, *J* = 4.2 Hz, 2H); ^{13}C NMR (151 MHz, Chloroform-*d*) δ 193.39, 169.79, 134.28, 134.21, 128.98, 127.92, 55.50, 46.62, 22.64; ^{13}C{^{1}H} NMR (151 MHz, Chloroform-*d*) δ 193.1, 163.7, 136.1, 134.5, 134.2, 134.1, 133.1, 129.0, 128.2, 128.0, 46.7.

HRMS (ESI) m/z: $[M+H]^+$ calcd for $C_{15}H_{11}Cl_3NO_2$ 341.9855; found 341.9847.

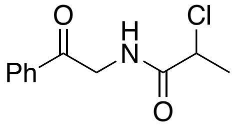

2-chloro-*N*-(2-oxo-2-phenylethyl)propenamide (**1ar**): Following the general procedure , purification by silica gel chromatography with petroleum ether and EtOAc (2:1) as eluent afforded the product **1ar** (383.0 mg, 85%) as a white solid.

Rf = 0.41 (petroleum ether EtOAc = 2:1)

m.p. 88.7 - 89.1 °C

^{1}H NMR (600 MHz, Chloroform-*d*) δ 7.99 (d, *J* = 7.4 Hz, 2H), 7.66 – 7.61 (m, 2H), 7.52 (t, *J* = 7.8 Hz, 2H), 4.78 (d, *J* = 4.4 Hz, 2H), 4.50 (q, *J* = 7.0 Hz, 1H), 1.79 (d, *J* = 7.0 Hz, 3H); ^{13}C NMR (151 MHz, Chloroform-*d*) δ 193.4, 169.8, 134.3, 134.2, 129.0, 127.9, 55.5, 46.6, 22.6.

HRMS (ESI) m/z: $[M+H]^+$ calcd for $C_{11}H_{13}ClNO_2$ 226.0635; found 226.0639.

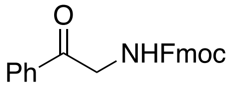

(9*H*-fluoren-9-yl)methyl (2-oxo-2-phenylethyl)carbamate (**1au**): Following the general procedure , purification by crystallization with petroleum ether and EtOAc afforded the product **1au** (442.8 mg, 62%) as a white solid.

Rf = 0.50 (petroleum ether: EtOAc = 2:1)

m.p. 142.7 - 143.6 °C

^{1}H NMR (400 MHz, Chloroform-d) δ 7.98 (d, J = 7.7 Hz, 2H), 7.77 (d, J = 7.6 Hz, 2H), 7.63 (t, J = 7.6 Hz, 3H), 7.51 (t, J = 7.6 Hz, 2H), 7.41 (t, J = 7.5 Hz, 2H), 7.33 (t, J = 7.4 Hz, 2H), 5.86 (s, 1H), 4.74 (d, J = 4.5 Hz, 2H), 4.43 (d, J = 7.2 Hz, 2H), 4.26 (t, J = 7.1 Hz, 1H); ^{13}C{^{1}H} NMR (151 MHz, Chloroform-*d*) δ 194.0, 156.2, 143.8, 141.3, 134.3, 134.1, 128.9, 127.9, 127.1, 125.1, 120.0, 67.2, 47.8, 47.1.

HRMS (ESI) m/z: [M+Na]$^+$ calcd for $C_{23}H_{19}NNaO_3$ 380.1263; found 380.1230.

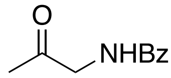

N-(2-oxopropyl)benzamide (**1bi**): Following the general procedure , purification by silica gel chromatography with petroleum ether and EtOAc (2:1) as eluent afforded the product **1bi** (205.6 mg, 58%) as a white solid.

^{1}H NMR (600 MHz, Chloroform-*d*) δ 7.82 (dd, *J* = 8.3, 1.3 Hz, 2H), 7.52 (t, *J* = 7.4 Hz, 1H), 7.45 (t, *J* = 7.6 Hz, 2H), 6.94 (bs, 1H), 4.37 (d, *J* = 4.4 Hz, 2H), 2.28 (s, 3H); ^{13}C{^{1}H} NMR (151 MHz, Chloroform-*d*) δ 202.9, 167.2, 133.7, 131.8, 128.6, 127.0, 50.3, 27.4. The spectral characteristics are in agreement with spectral data previously reported.[11]

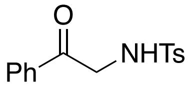

4-methyl-*N*-(2-oxo-2-phenylethyl)benzenesulfonamide (**1av**): Following the general procedure , purification by crystallization with ethyl acetate and petroleum ether afforded the product **1av** (318.6 mg, 55%) as a white solid.

^1H NMR (600 MHz, Chloroform-*d*) δ 7.85 (d, J = 7.7 Hz, 2H), 7.78 (d, J = 8.3 Hz, 2H), 7.61 (t, J = 7.4 Hz, 1H), 7.47 (t, J = 7.2 Hz, 2H), 7.29 (d, J = 8.0 Hz, 2H), 5.65 (bs, 1H), 4.46 (d, J = 4.5 Hz, 2H), 2.39 (s, 3H); $^{13}C\{^1H\}$ NMR (151 MHz, Chloroform-*d*) δ 192.5 (d, J = 3.4 Hz), 143.8, 136.1, 134.4, 133.8, 129.8, 129.0, 127.9, 127.2, 48.6, 21.5. The spectral characteristics are in agreement with spectral data previously reported.[12]

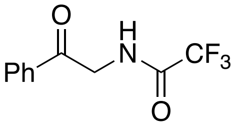

2,2,2-trifluoro-*N*-(2-oxo-2-phenylethyl)acetamide (**1aq**): Et_3N (445.0 mg, 4.4 mmol, 2.2 equiv.) was added to a suspension of 2-aminoacetophenone hydrochloride (342 mg, 2 mmol) in CH_2Cl_2 (5 mL). Trifluoroacetic anhydride (546.0 mg, 2.6 mmol, 1.3 equiv.) was added to this suspension at 0 °C and the suspension was allowed to warm to room temperature for 2 h. Water (5 mL) was added, the mixture extracted with CH_2Cl_2 (3*5 mL), the combined organic layer washed with sat. $NaHCO_3$ (10 mL) and brine (10 mL). The solution was dried over Na_2SO_4, filtered and concentrated under reduced pressure. Purification by silica gel chromatography with ethyl acetate and petroleum ether (1:2) as eluent afforded the product **1aq** (126.2 mg, 27%) as a white solid. ^1H NMR (600 MHz, Chloroform-*d*) δ 7.99 (d, J = 8.3 Hz, 2H), 7.69 – 7.66 (m, 1H), 7.58 – 7.49 (m, 3H), 4.83 (d, J = 4.3 Hz, 2H); $^{13}C\{^1H\}$ NMR (151 MHz, Chloroform-*d*) δ 192.0, 157.2 (q, J_{CF} = 37.7 Hz), 134.8, 133.6, 129.2, 128.0, 115.7 (q, J_{CF} = 287.4 Hz), 46.2. The spectral characteristics are in agreement with spectral data previously reported.[13]

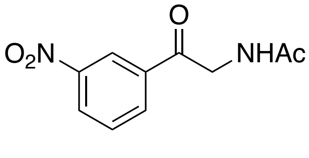

N-(2-(3-nitrophenyl)-2-oxoethyl)acetamide (**1bd**): 2-amino-1-(3-nitrophenyl)ethan-1-one hydrochloride (432.0 mg, 2 mmol) was suspended in Ac_2O (0.5 mL) and anhydrous NaOAc (328.0 mg, 4 mmol, 2.0 equiv.) was gradually added. After 30 min, water (2 mL) was added and the mixture was stirred vigorously for 1 h. The resulting solution was diluted with sat. aq $NaHCO_3$ (6 mL) and extracted with EtOAc (2*10 mL). The

combined organic layers were washed with sat. aq $NaHCO_3$ (6 mL), sat. aq NaCl (6 mL) and dried over Na_2SO_4. Filtration and removal of the solvent under reduced pressure produced the crude product that could be purified by crystallization to afford the product **1bd** (277.4 mg, 62%) as a white solid.

Rf = 0.21 (EtOAc)

m.p. 95.2 - 98.7 ℃

^{1}H NMR (600 MHz, Chloroform-*d*) δ 8.82 (t, *J* = 2.0 Hz, 1H), 8.50 – 8.48 (m, 1H), 8.31 (d, *J* = 7.9 Hz, 1H), 7.75 (t, *J* = 8.0 Hz, 1H), 6.53 (bs, 1H), 4.84 (d, *J* = 4.5 Hz, 2H), 2.14 (s, 3H); ^{13}C{^{1}H} NMR (151 MHz, Chloroform-*d*) δ 192.5, 170.3, 148.6, 135.6, 133.4, 130.3, 128.3, 122.9, 46.8, 23.0.

HRMS (ESI) m/z: $[M+H]^+$ calcd for $C_{10}H_{11}N_2O_4$ 223.0719; found 223.0720.

2. Unsuccessful Carbonyl Compounds for Oxidative Cross-Coupling with Benzyl Alcohol

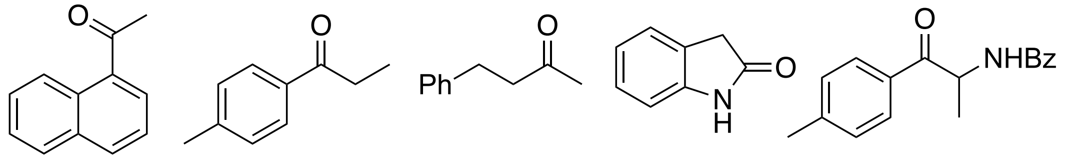

Unless otherwise noted, carbonyl compounds were purchased from Shanghai Xian-Ding and used as received.

Preparation of *N*-(1-oxo-1-(*p*-tolyl)propan-2-yl)benzamide

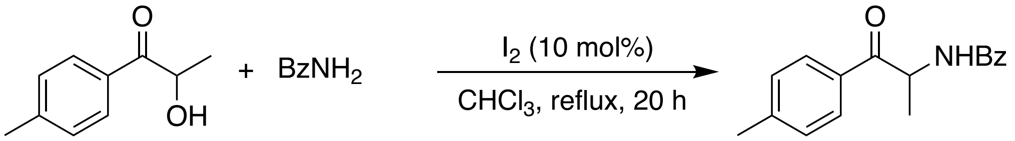

2-Hydroxy-1-(*p*-tolyl)propan-1-one is known compound and was synthesized according to the related literature.[14]

To a flame-dried round-bottomed flask were added 2-hydroxy-1-(*p*-tolyl)propan-1-one (820 mg, 5 mmol), benzamide (605 mg, 5 mol, 1.0 equiv.), iodine (127 mg, 0.5 mol, 10 mol%) and $CHCl_3$ (20 mL). The mixture was refluxed for 20 h, cooled to room temperature and then quenched by 10% $Na_2S_2O_3$ (30 mL) solution (w/w) and extracted with CH_2Cl_2 (3 * 15 mL). The combined organic layers were washed with brine (30 mL), dried over Na_2SO_4, filtered, and concentrated in vacuo. The crude product was purified by column chromatography on silica gel (eluent: petroleum ether: EtOAc = 4:1) to afford the title product (552 mg, 41% yield) as a yellow solid.

Rf = 0.37 (petroleum ether: EtOAc = 4:1)

m. p. 107.3 - 111.0 °C

^{1}H NMR (600 MHz, Chloroform-*d*) δ 7.95 (d, *J* = 8.3 Hz, 2H), 7.86 (d, *J* = 7.1 Hz, 2H), 7.53 – 7.50 (m, 1H), 7.47 – 7.44 (m, 2H), 7.40 (d, *J* = 7.0 Hz, 1H), 7.32 (d, *J* = 8.0 Hz, 2H), 5.74 (p, *J* = 7.0 Hz, 1H), 2.44 (s, 3H), 1.54 (d, *J* = 7.1 Hz, 3H); ^{13}C{^{1}H} NMR (151 MHz, Chloroform-*d*) δ 198.7, 166.6, 145.1, 134.2, 131.6, 131.2, 129.6, 128.9, 128.6, 127.0, 50.4, 21.7, 20.1.

HRMS (ESI) m/z: $[M+H]^+$ calcd for $C_{17}H_{18}N_2$ 268.1338; found 268.1343.

3. Mechanistic Studies

(a) Reaction in the absence of alcohol

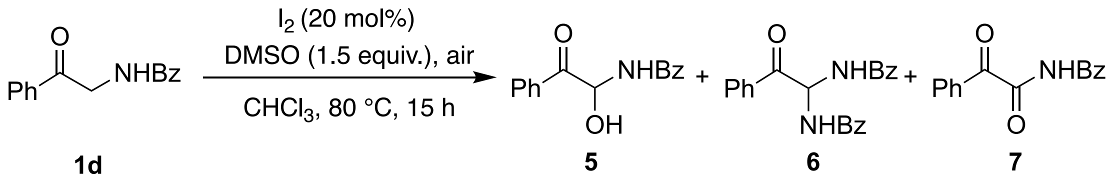

To a screw capped vial was charged with *N*-(1-hydroxy-2-oxo-2-phenylethyl)benzamide **1d** (47.8 mg, 0.2 mmol, 1.0 equiv.), I_2 (10.2 mg, 0.04 mmol, 20 mol%) and DMSO (21.3 μL, 0.3 mmol, 1.5 equiv.), followed by addition of anhydrous $CHCl_3$ (1 mL). The vial was tightly capped and stirred at 80 °C for 15 h. When the reaction was completed, the crude reaction mixture was allowed to reach room temperature. The solution was then quenched by 10% $Na_2S_2O_3$ (2 mL) solution (w/w) and extracted with CH_2Cl_2 (3 * 5 mL). The combined organic layers were dried over Na_2SO_4, filtered, and concentrated in vacuo. The crude product was purified by column chromatography on silica gel (For **5** and **6**: petroleum ether: EtOAc = 1: 1; for **7**: petroleum ether: EtOAc = 1.5:1) to afford the product **5** (4.6 mg, 9%), **6** (19.8 mg, 55%) and **7** (17.5 mg, 35%).

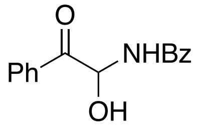

N-(1-hydroxy-2-oxo-2-phenylethyl)benzamide (**5**)

white solid

R_f = 0.30 (petroleum ether: EtOAc = 1: 1)

m. p. 139.8 - 142.3 °C

^1H NMR (600 MHz, DMSO-d_6) δ 9.36 (d, J = 6.9 Hz, 1H), 7.98 (d, J = 7.2 Hz, 2H), 7.86 (d, J = 7.2 Hz, 2H), 7.62 (t, J = 7.4 Hz, 1H), 7.55 – 7.49 (m, 3H), 7.45 (t, J = 7.7

Hz, 2H), 6.52 – 6.48 (m, 2H); $^{13}C\{^{1}H\}$ NMR (151 MHz, DMSO-d_6) δ 195.6, 166.5, 134.7, 134.0, 133.9, 132.2, 129.1, 129.0, 128.9, 128.0, 74.1.

HRMS (ESI) m/z: $[M+Na]^+$ calcd for $C_{15}H_{13}NNaO_3$ 278.0793; found 278.0791.

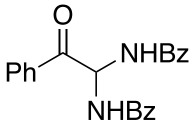

N,*N'*-(2-oxo-2-phenylethane-1,1-diyl)dibenzamide (**6**)

white solid

R_f = 0.28 (petroleum ether: EtOAc = 1: 1)

^{1}H NMR (600 MHz, DMSO-d_6) δ 9.24 (d, *J* = 7.4 Hz, 2H), 8.00 (d, *J* = 7.3 Hz, 2H), 7.89 (d, *J* = 7.0 Hz, 4H), 7.65 – 7.62 (m, 1H), 7.58 – 7.52 (m, 4H), 7.48 (t, *J* = 7.6 Hz, 4H), 7.02 (t, *J* = 7.4 Hz, 1H); $^{13}C\{^{1}H\}$ NMR (151 MHz, DMSO-d_6) δ 193.0, 166.5, 134.8, 134.0, 133.7, 132.3, 129.2, 128.9, 128.6, 128.0, 60.2. The spectral characteristics are in agreement with spectral data previously reported.[15]

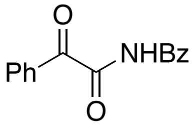

N-(2-oxo-2-phenylacetyl)benzamide (**7**)

light yellow solid

R_f = 0.48 (petroleum ether: EtOAc = 1.5: 1)

^{1}H NMR (600 MHz, Chloroform-*d*) δ 9.93 (bs, 1H), 8.13 (d, *J* = 7.6 Hz, 2H), 7.93 – 7.92 (m, 2H), 7.68 – 7.62 (m, 2H), 7.54 – 7.50 (m, 4H); $^{13}C\{^{1}H\}$ NMR (151 MHz, Chloroform-*d*) δ 186.6, 165.3, 165.3, 134.7, 134.0, 132.3, 130.1, 129.1, 128.9, 128.1, 128.1. The spectral characteristics are in agreement with spectral data previously reported.[16]

(b) Reaction between key intermediate **5** and alcohol

Preparation of substrate **5**

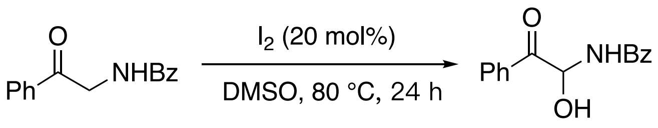

To a screw capped vial was charged with *N*-(2-oxo-2-phenylethyl)benzamide 1d (239.1 mg, 1.0 mmol, 1.0 equiv) and I_2 (50.8 mg, 0.2 mmol, 20 mol%), followed by addition of DMSO (2 mL). The vial was tightly capped and stirred at 80 °C for 24 h. When the reaction was completed, the crude reaction mixture was allowed to reach room temperature. The solution was then quenched by 10% $Na_2S_2O_3$ (15 mL) solution (w/w) and extracted with CH_2Cl_2 (3 * 15 mL). The combined organic layers were washed with brine (30 mL), dried over Na_2SO_4, filtered, and concentrated in vacuo. The crude product was purified by column chromatography on silica gel (eluent: CH_2Cl_2: EtOAc = 6:1) to afford the product **5** (168.2 mg, 66% yield) as a white solid.

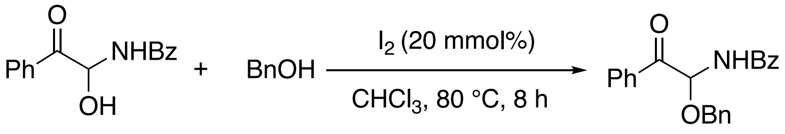

To a screw capped vial was charged with *N*-(1-hydroxy-2-oxo-2-phenylethyl)benzamide **5** (51.0 mg, 0.2 mmol, 1.0 equiv), benzyl alcohol **2a** (0.24 mol, 1.2 equiv) and I_2 (10.2 mg, 0.04 mmol, 20 mol%), followed by addition of anhydrous $CHCl_3$ (1 mL). The vial was tightly capped and stirred at 80 °C for 8 h. When the reaction was completed, the crude reaction mixture was allowed to reach room temperature. The solution was then quenched by 10% $Na_2S_2O_3$ (2 mL) solution (w/w) and extracted with CH_2Cl_2 (2 * 4 mL). The combined organic layers were dried over Na_2SO_4, filtered, and concentrated in vacuo. The crude product was purified by column chromatography on silica gel (eluent: petroleum ether/ EtOAc) to afford the product **3d** (57.8 mg, 84%).

4. References

1 Zall A, Kieser D, Höttecke N, Naumann EC, Thomaszewski B, Schneider K, Steinbacher DT, Schubenel R, Masur S, Baumann K, Schmidt B. *Bioorg Med Chem,* 2011, 19: 4903-4909

2 Ming XX, Wu S, Tian ZY, Song JW, Zhang CP. *Org Lett,* 2021, 23: 6795-6800

3 Günther R, Bordusa F. *Chem – Eur J,* 2000, 6: 463-467

4 Hung JM, Arabshahi HJ, Leung E, Reynisson J, Barker D. *Eur J Med Chem,* 2014, 86: 420-437

5 Winkel A, Wilhelm R. *Tetrahedron: Asymmetry,* 2009, 20: 2344-2350

6 Assali M, Abualhasan M, Sawaftah H, Hawash M, Mousa A. *J Chem,* 2020, 2020: 6393428

7 Wenschuh H, Beyermann M, Haber H, Seydel JK, Krause E, Bienert M, Carpino LA, El-Faham A, Albericio F. *J Org Chem,* 1995, 60: 405-410

8 Wang Y, Yang M, Lao C, Jiang Z. *Org Lett,* 2022, 24: 2625-2629

9 Prein M, Manley PJ, Padwa A. *Tetrahedron,* 1997, 53: 7777-7794

10 Zhu M, Chen D, Zeng S, Xing C, Deng W, Xiang J, Wang R-J. *Tetrahedron Lett,* 2018, 59: 3214-3219

11 Sakurai M, Kihara N, Watanabe N, Ikari Y, Takata T. *Chem Lett,* 2018, 47: 144-147

12 Zhang X, Li SS, Wang L, Xu L, Xiao J, Liu ZJ. *Tetrahedron,* 2016, 72: 8073-8077

13 De A, Santra S, Zyryanov GV, Majee A. *Org Lett,* 2020, 22: 3926-3930

14 Liang YF, Wu K, Song S, Li X, Huang X, Jiao N. *Org Lett,* 2015, 17: 876-879

15 Tajbakhsh M, Hosseinzadeh R, Alinezhad H, Rezaee P. *Synth Commun,* 2013, 43: 2370-2379

16 Wu X, Gao Q, Liu S, Wu A. *Org Lett,* 2014, 16: 2888-2891

5. Spectra

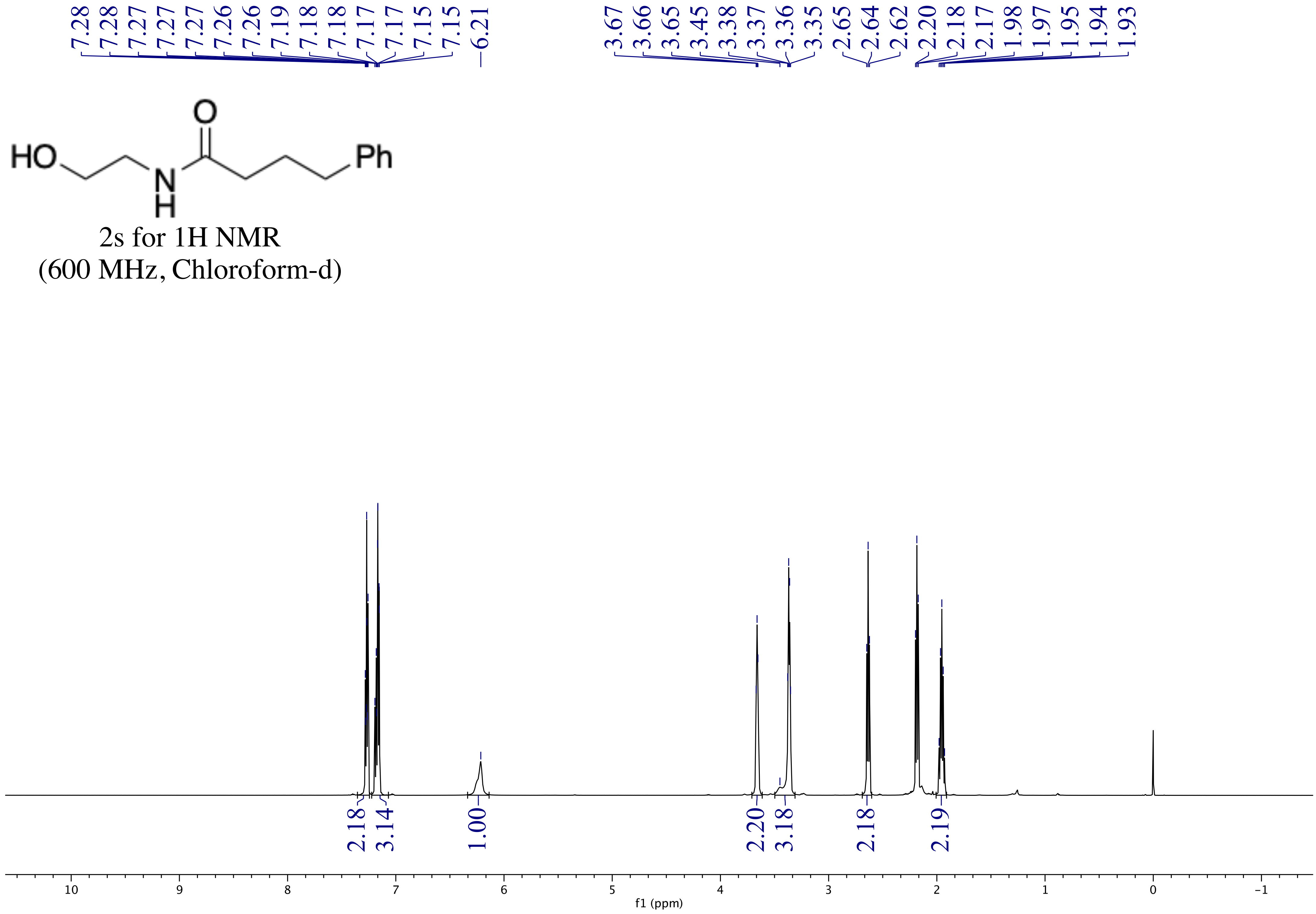
7.28 7.28 7.27 7.27 7.27 7.26 7.26 7.19 7.18 7.18 7.17 7.17 7.15 7.15 6.21
3.67 3.66 3.65 3.45 3.38 3.37 3.36 3.35 2.65 2.64 2.62 2.20 2.18 2.17 1.98 1.97 1.95 1.94 1.93
HO
N
H
O
Ph
2s for 1H NMR
(600 MHz, Chloroform-d)
2.18 3.14 1.00 2.20 3.18 2.18 2.19
10 9 8 7 6 5 4 3 2 1 0 -1
f1 (ppm)

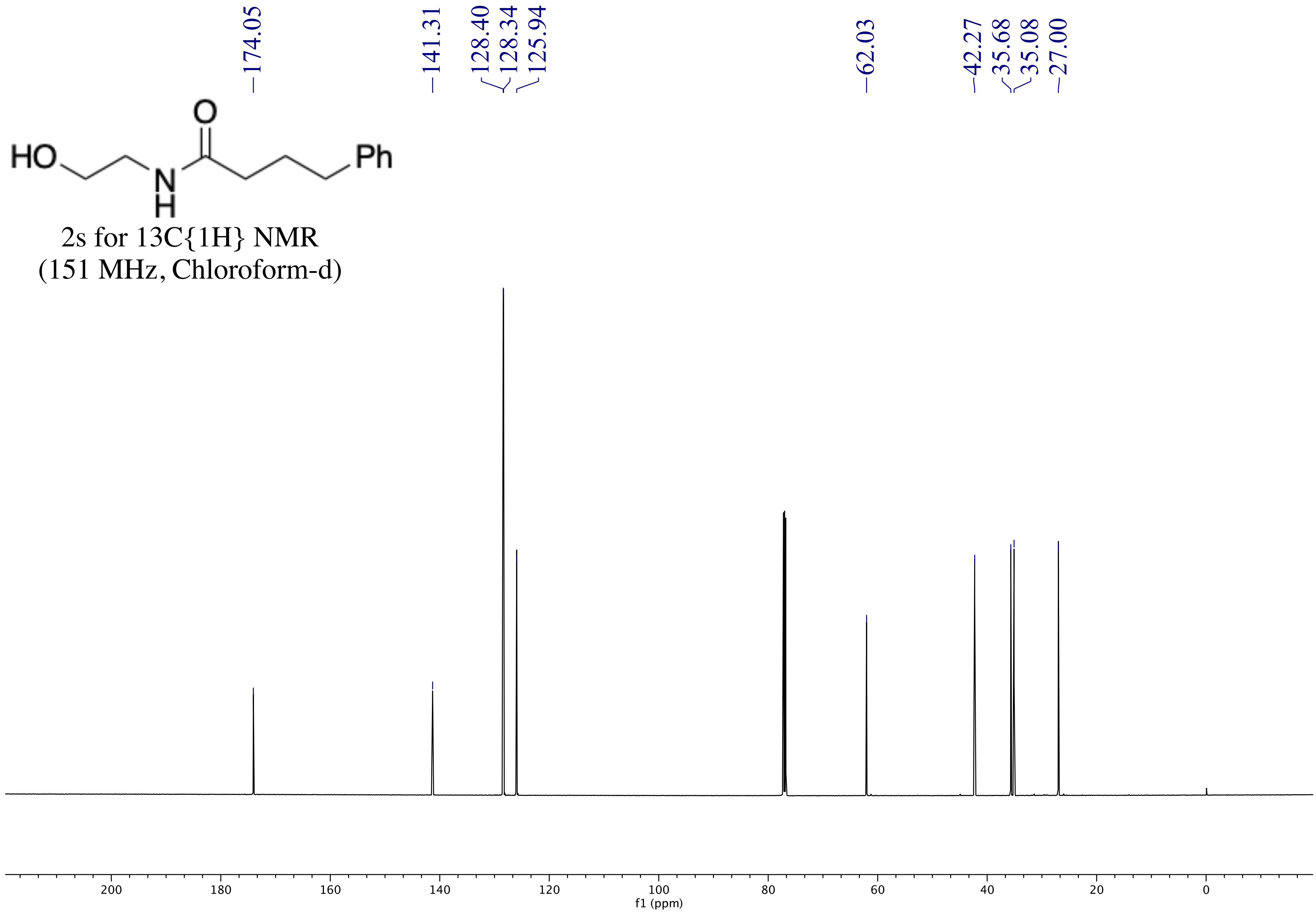

HO
N
H
O
Ph
2s for 13C{1H} NMR
(151 MHz, Chloroform-d)
174.05
141.31
128.40
128.34
125.94
62.03
42.27
35.68
35.08
27.00
200
180
160
140
120
100
80
60
40
20
0
f1 (ppm)

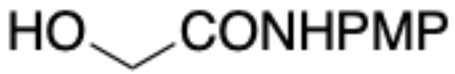

2t for 1H NMR
(600 MHz, Chloroform-d)

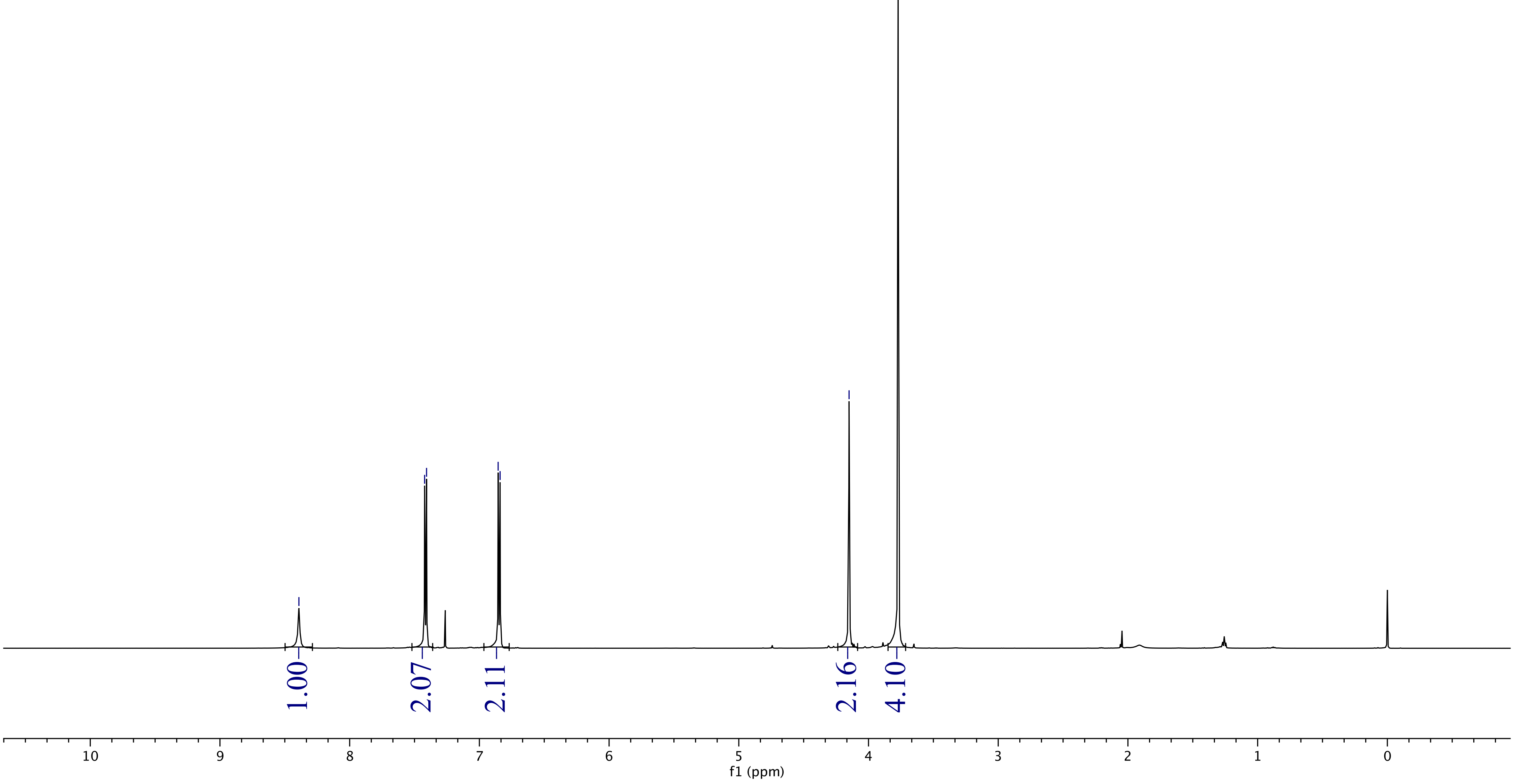

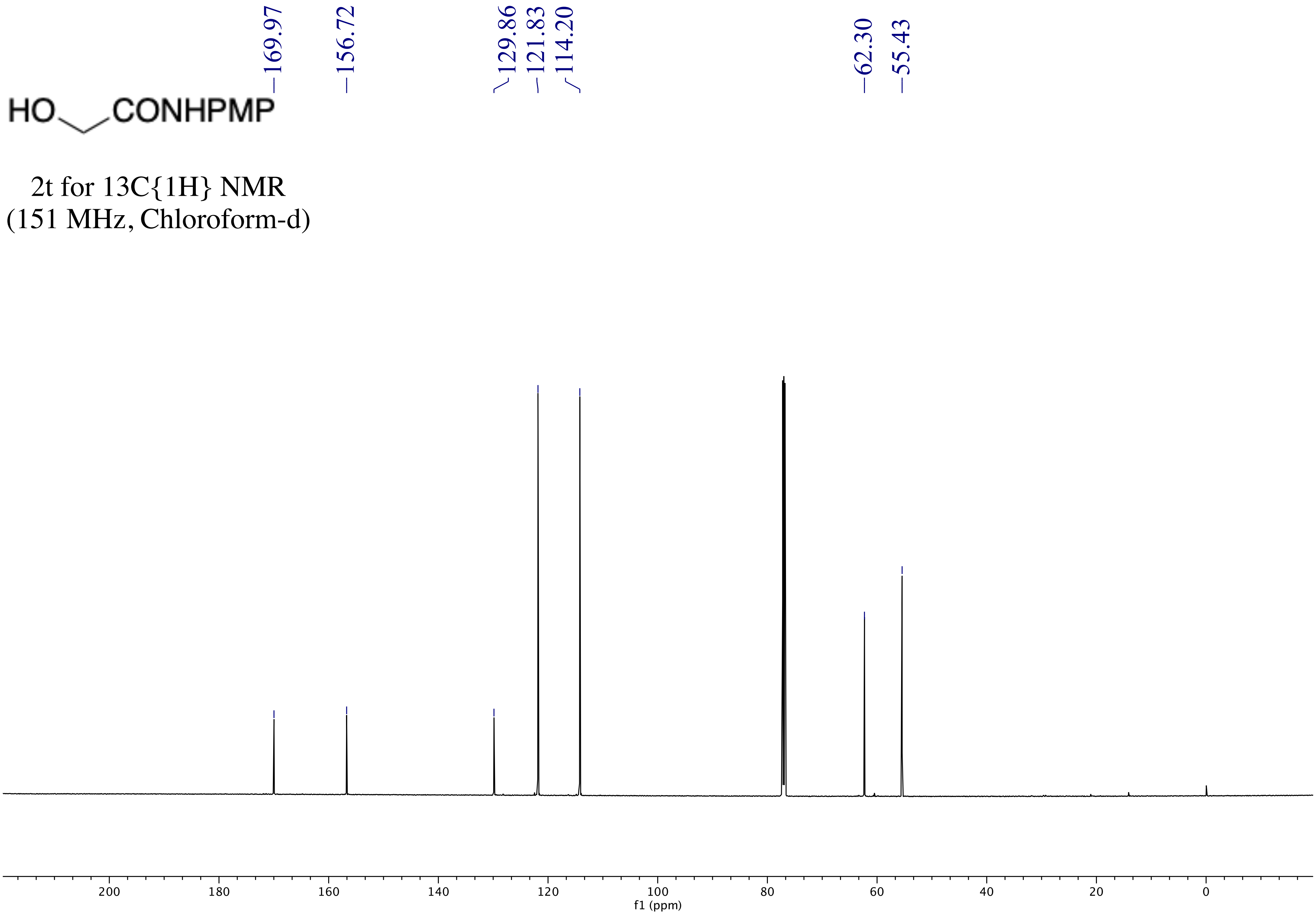
169.97
156.72
129.86
121.83
114.20
62.30
55.43
HO CONHPMP
2t for 13C{1H} NMR
(151 MHz, Chloroform-d)
200
180
160
140
120
100
80
60
40
20
0
f1 (ppm)

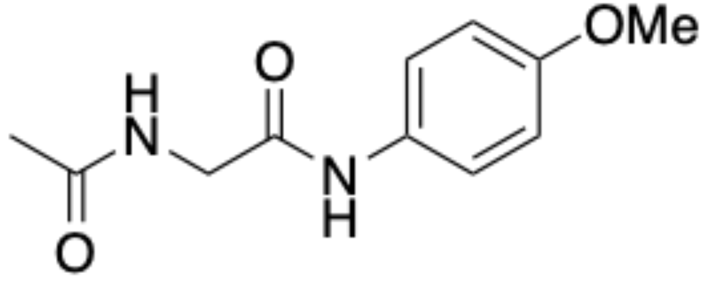

1b for 1H NMR
(600 MHz, DMSO-d)

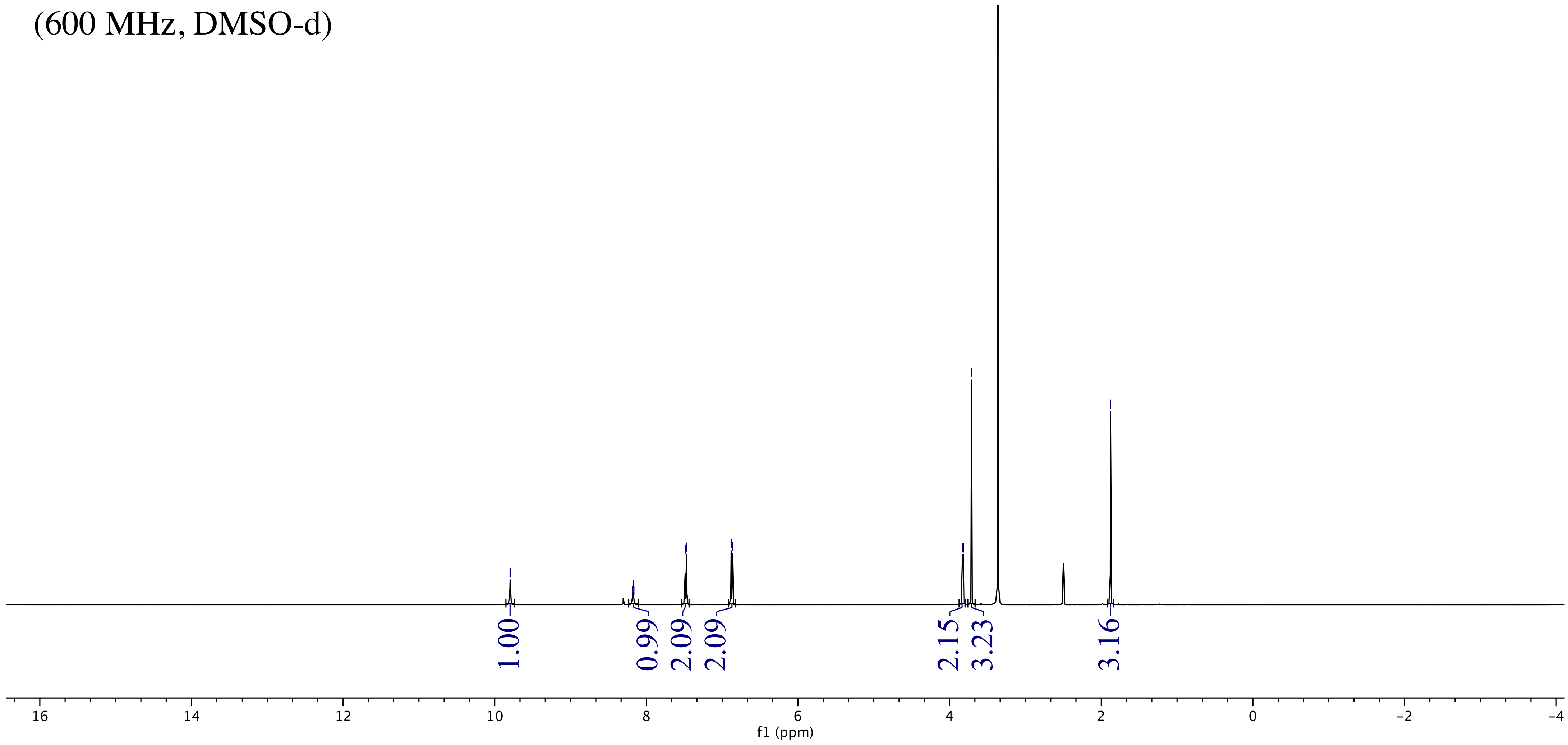

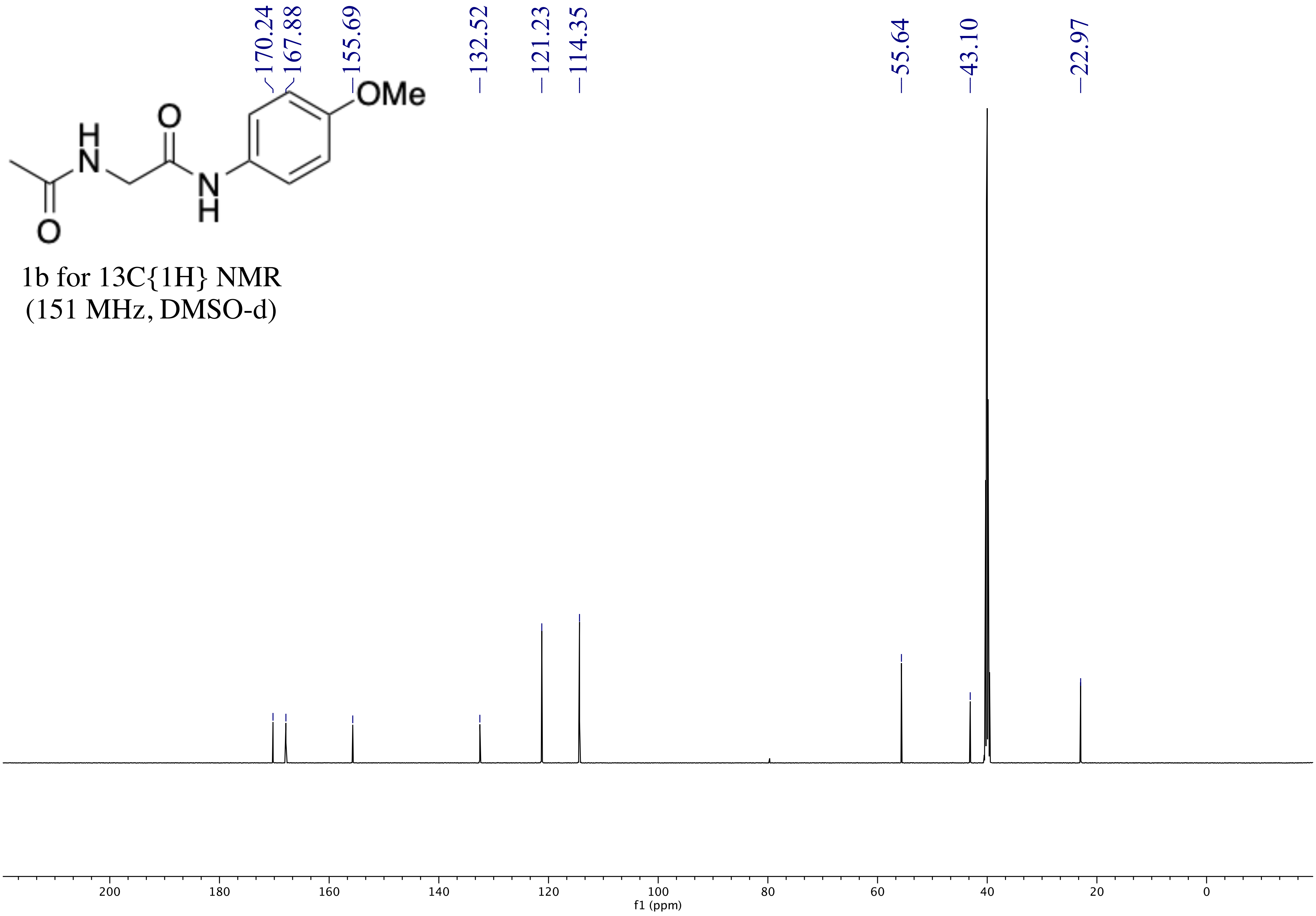

1b for 13C{1H} NMR
(151 MHz, DMSO-d)

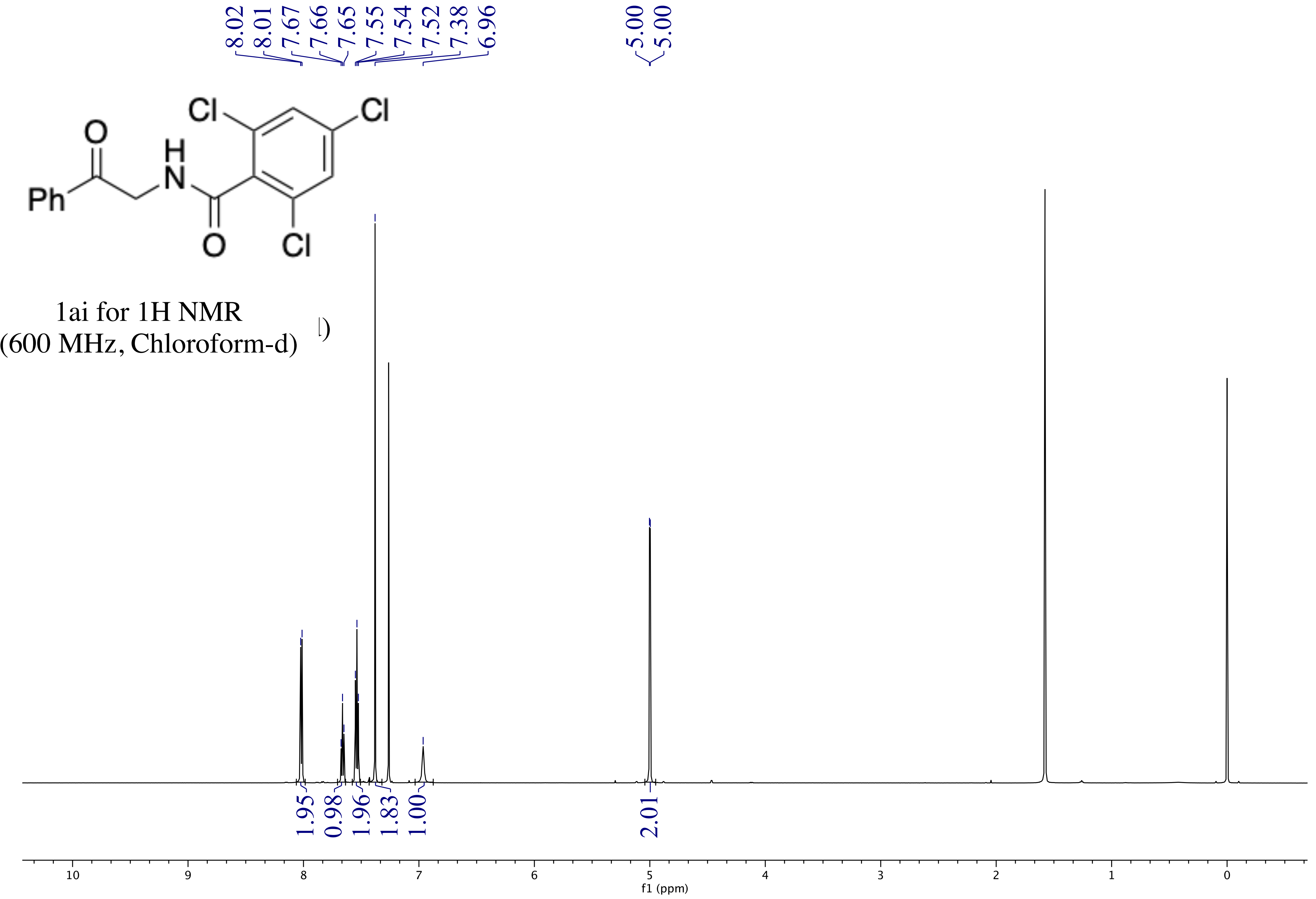
8.02
8.01
7.67
7.66
7.65
7.55
7.54
7.52
7.38
6.96
5.00
5.00
Cl
Cl
Cl
O
H
N
Ph
O
1ai for 1H NMR
(600 MHz, Chloroform-d)
1.95
0.98
1.96
1.83
1.00
2.01
10
9
8
7
6
5
4
3
2
1
0
f1 (ppm)

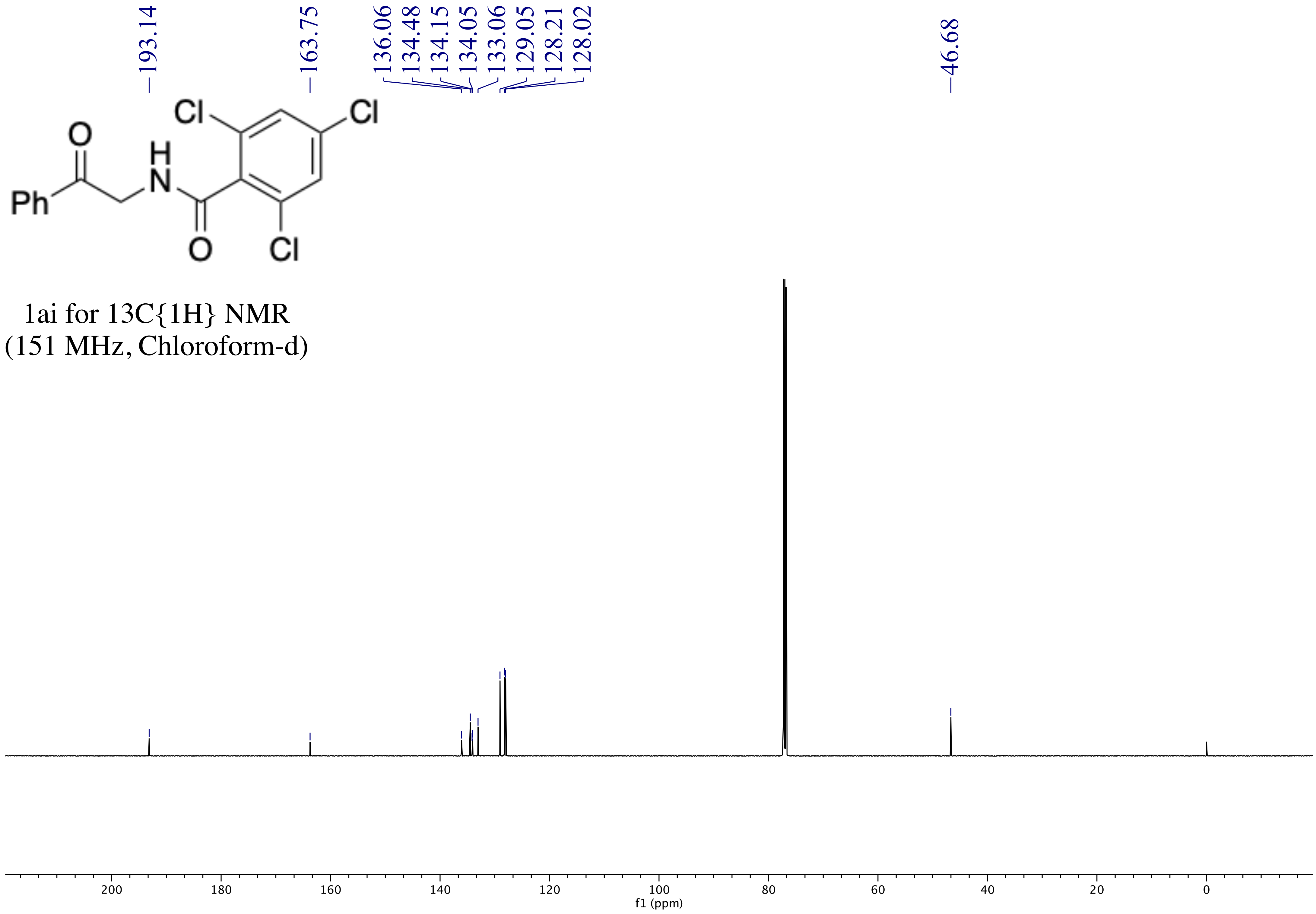
193.14
163.75
136.06
134.48
134.15
134.05
133.06
129.05
128.21
128.02
46.68
Ph
O
H
N
O
Cl
Cl
Cl
1ai for 13C{1H} NMR
(151 MHz, Chloroform-d)
200
180
160
140
120
100
80
60
40
20
0
f1 (ppm)

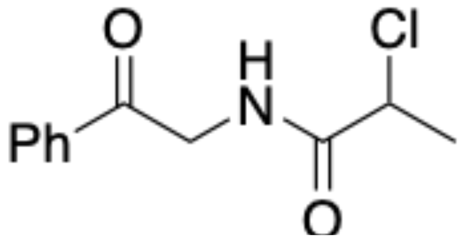

1ar for 1H NMR
(600 MHz, Chloroform-d)

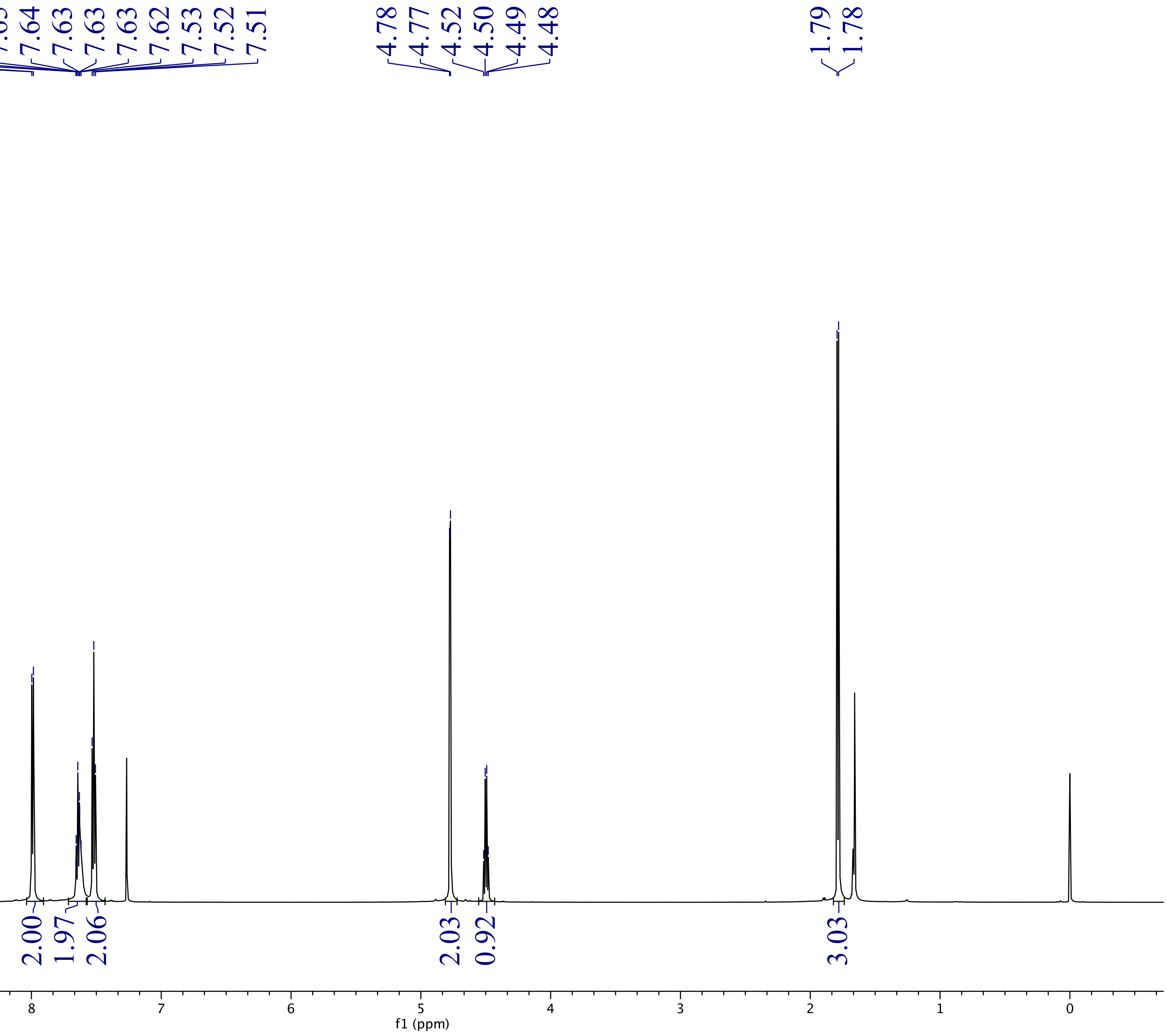

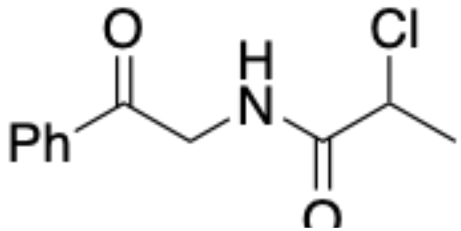

1ar for 13C{1H} NMR
(151 MHz, Chloroform-d)

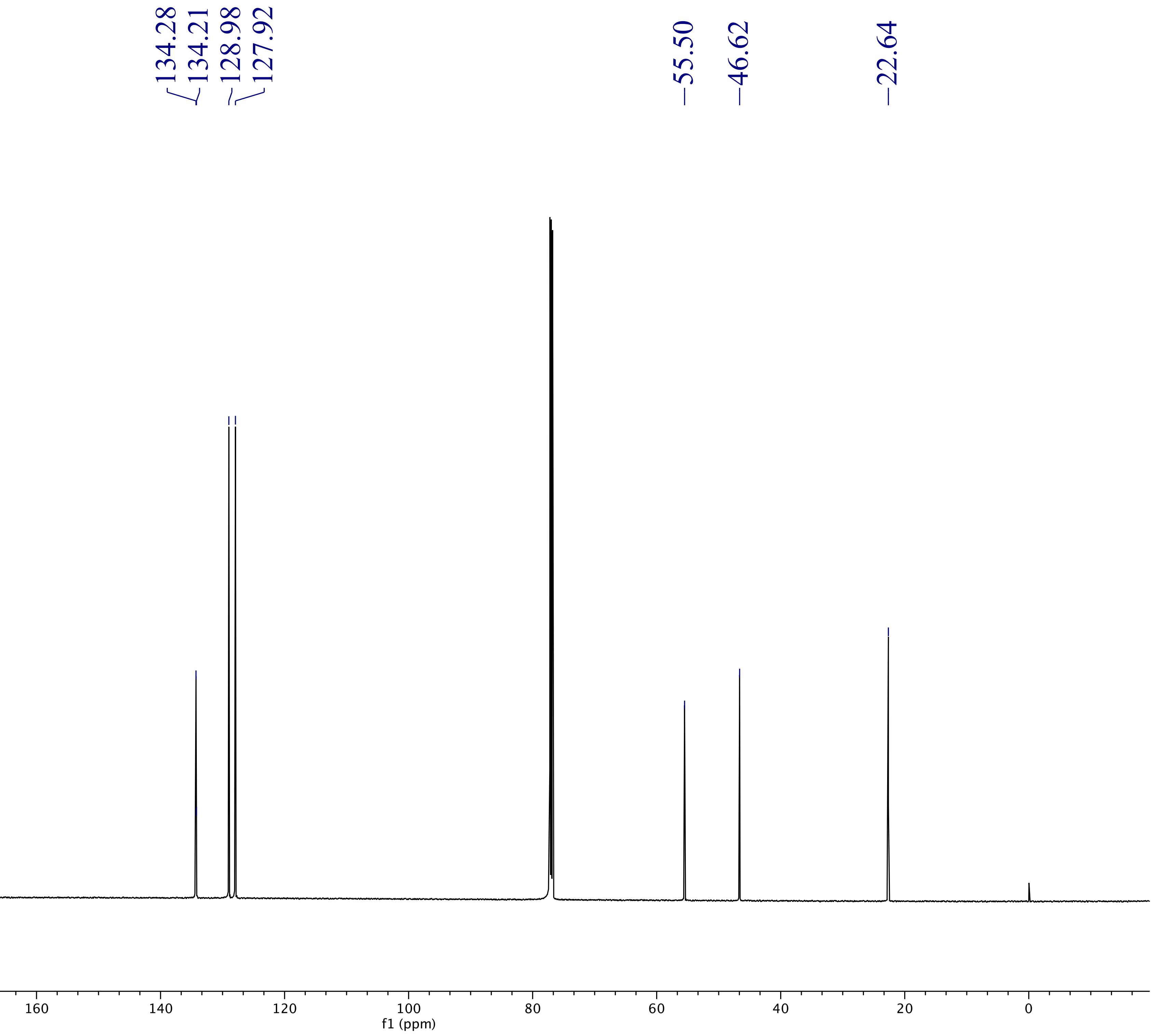

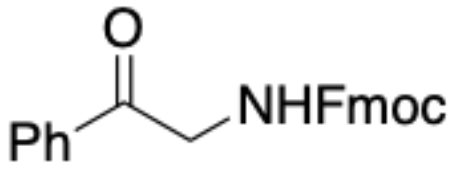

1au for 1H NMR
(600 MHz, Chloroform-d)

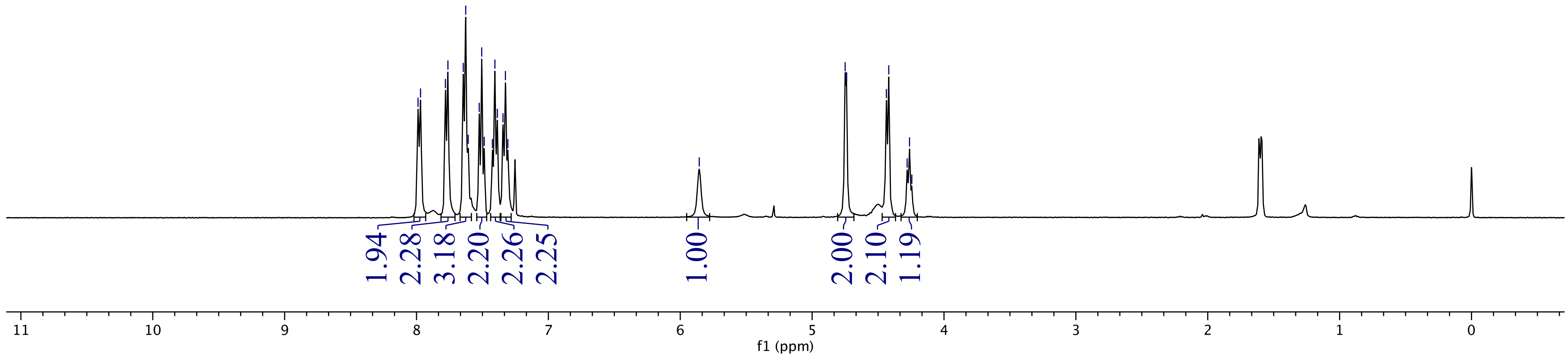

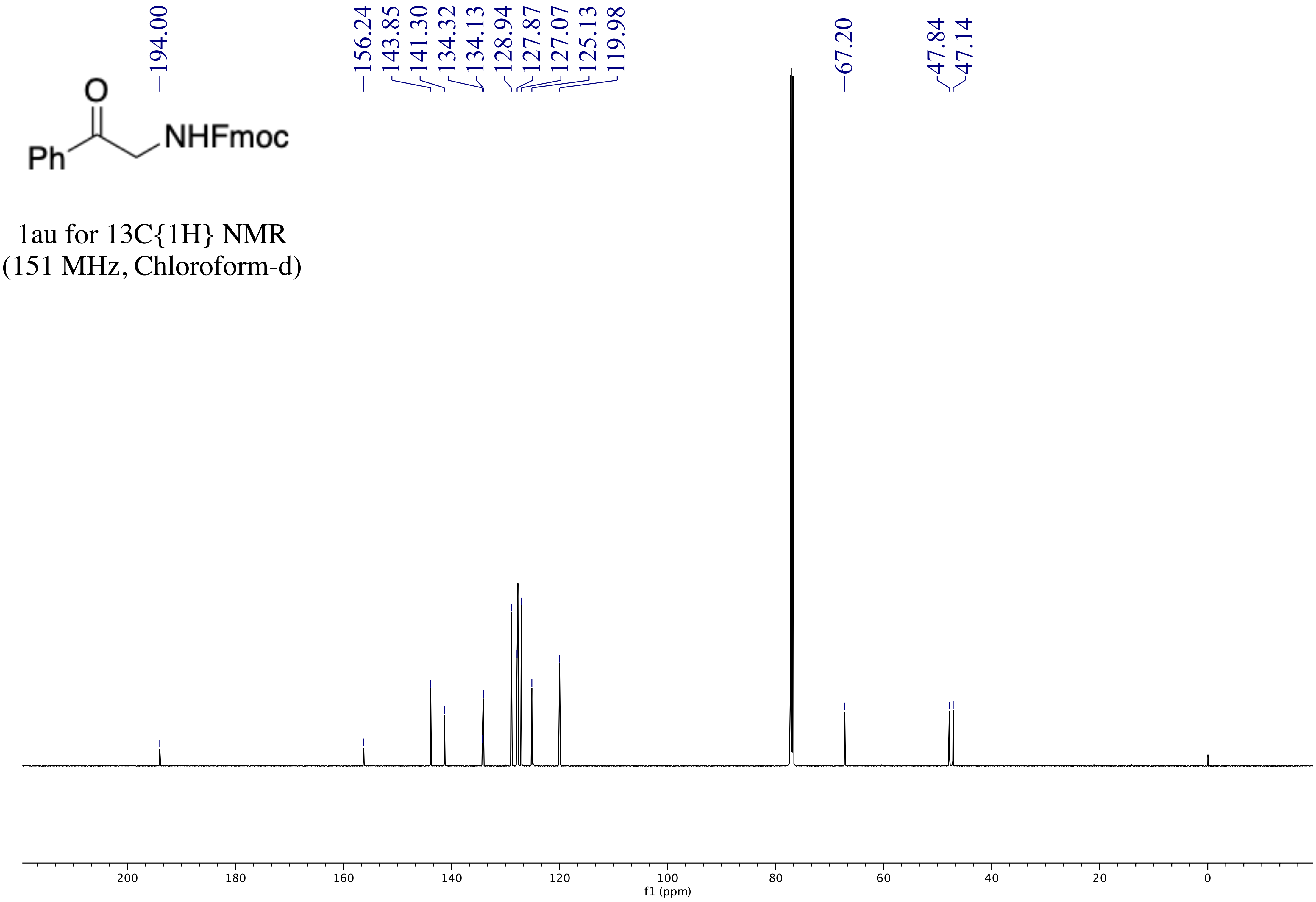

194.00
156.24
143.85
141.30
134.32
134.13
128.94
127.87
127.07
125.13
119.98
67.20
47.84
47.14
Ph
O
NHFmoc
1au for 13C{1H} NMR
(151 MHz, Chloroform-d)
200
180
160
140
120
100
80
60
40
20
0
f1 (ppm)

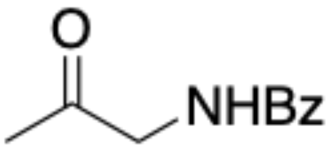

1bi for 1H NMR
(600 MHz, Chloroform-d)

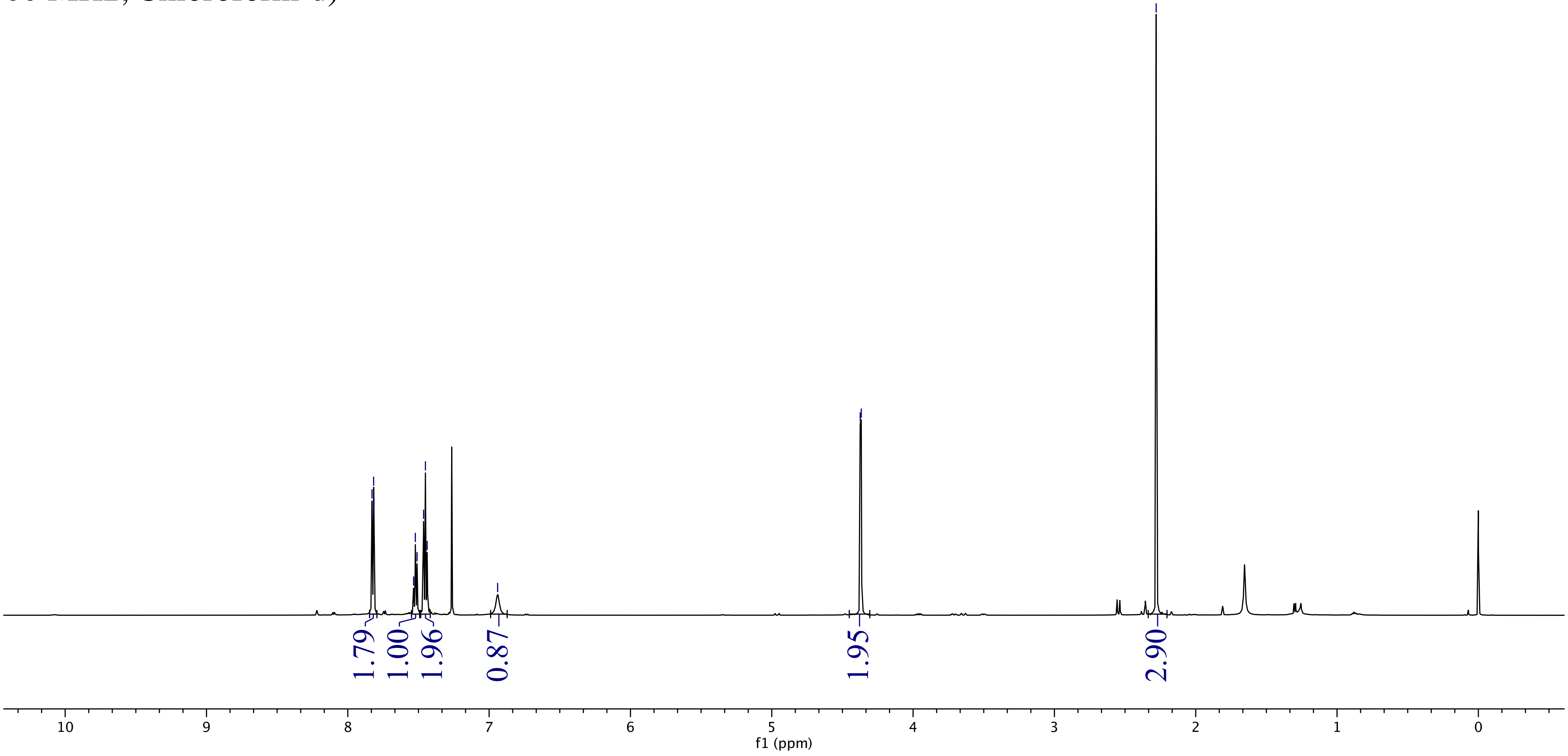

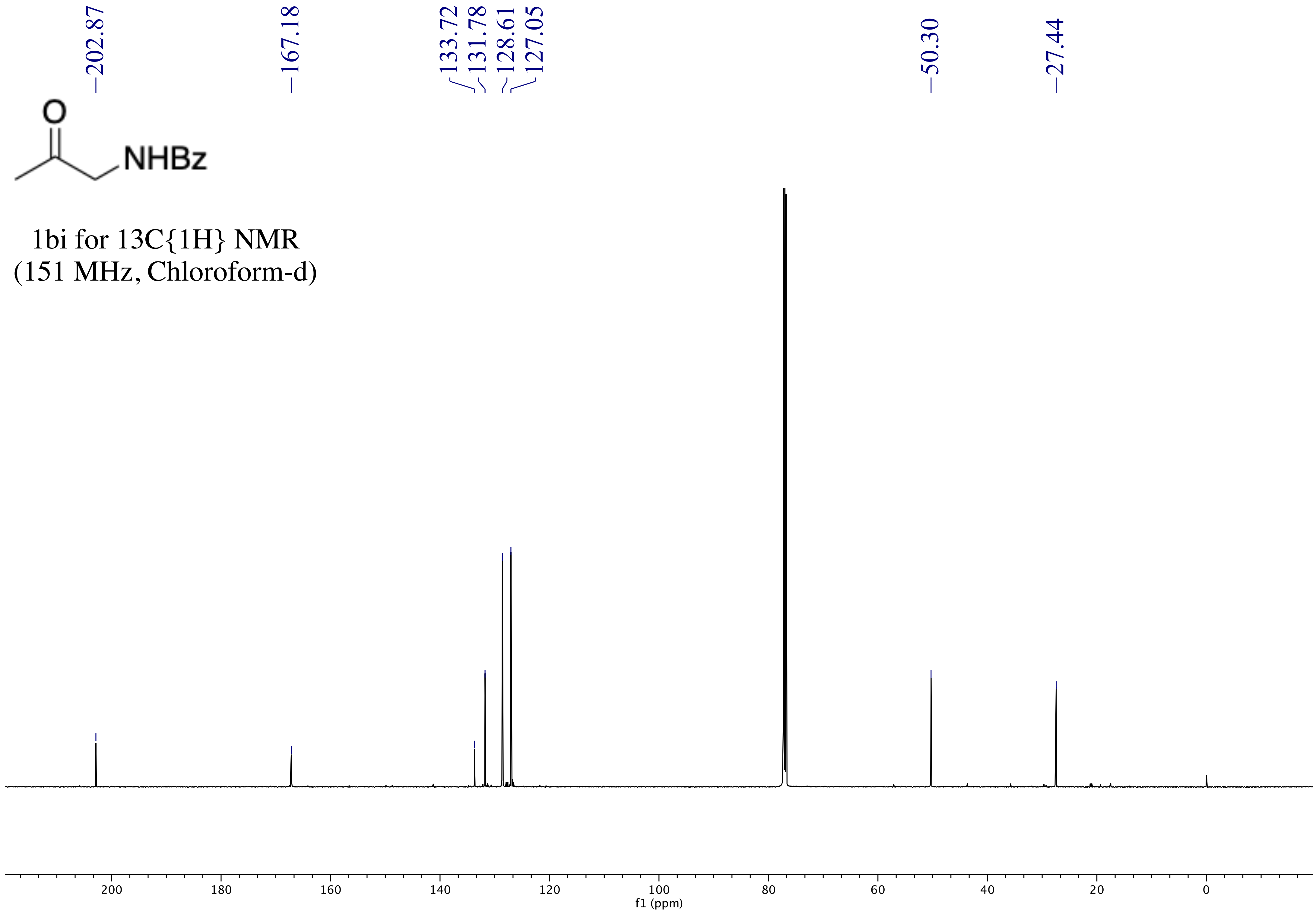

202.87
167.18
133.72
131.78
128.61
127.05
50.30
27.44
NHBz
1bi for 13C{1H} NMR
(151 MHz, Chloroform-d)
200
180
160
140
120
100
80
60
40
20
0
f1 (ppm)

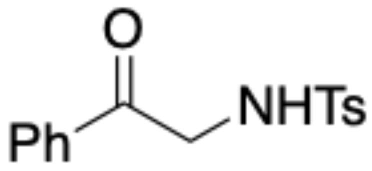

1av for 1H NMR
(600 MHz, Chloroform-d)

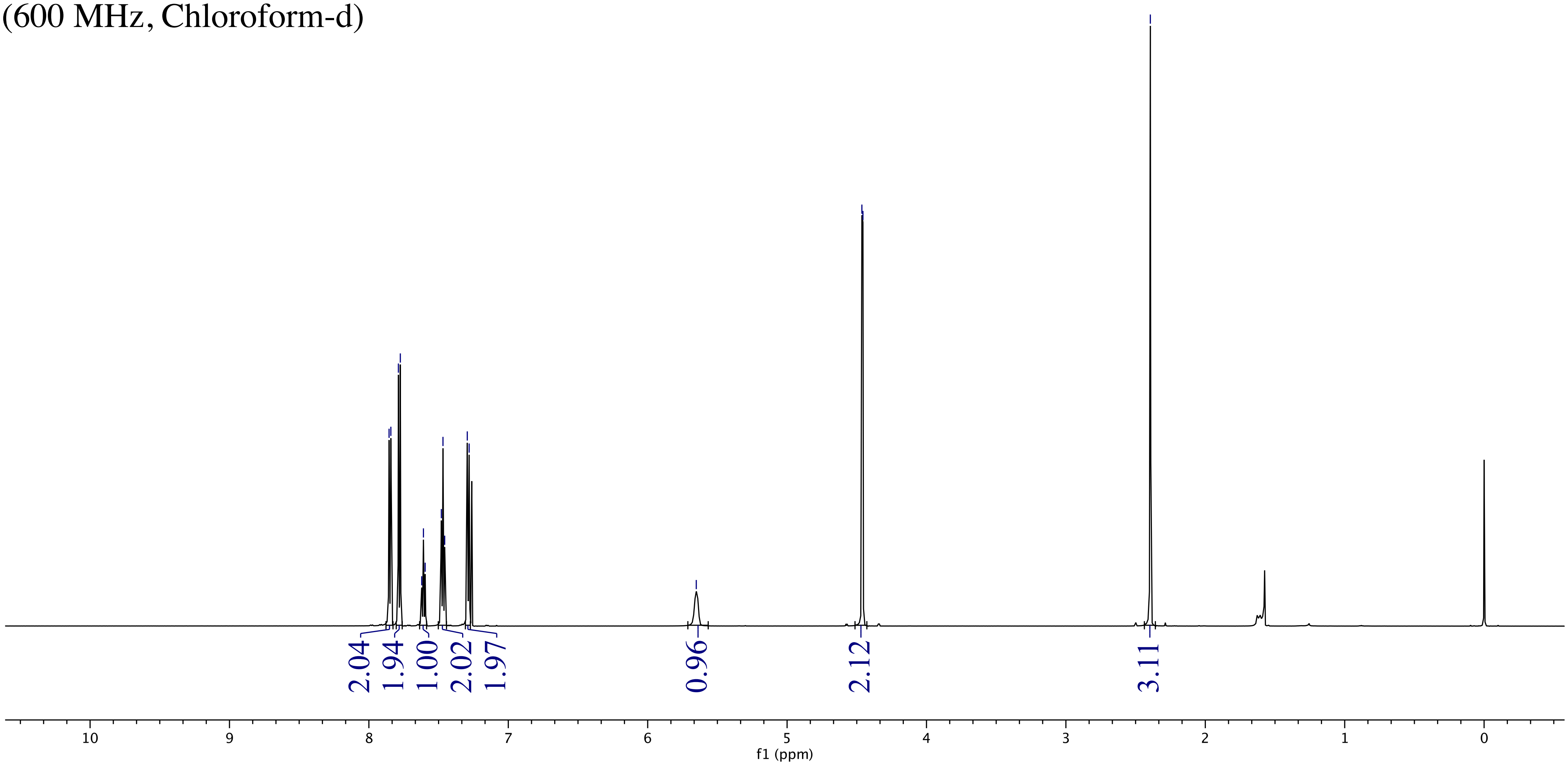

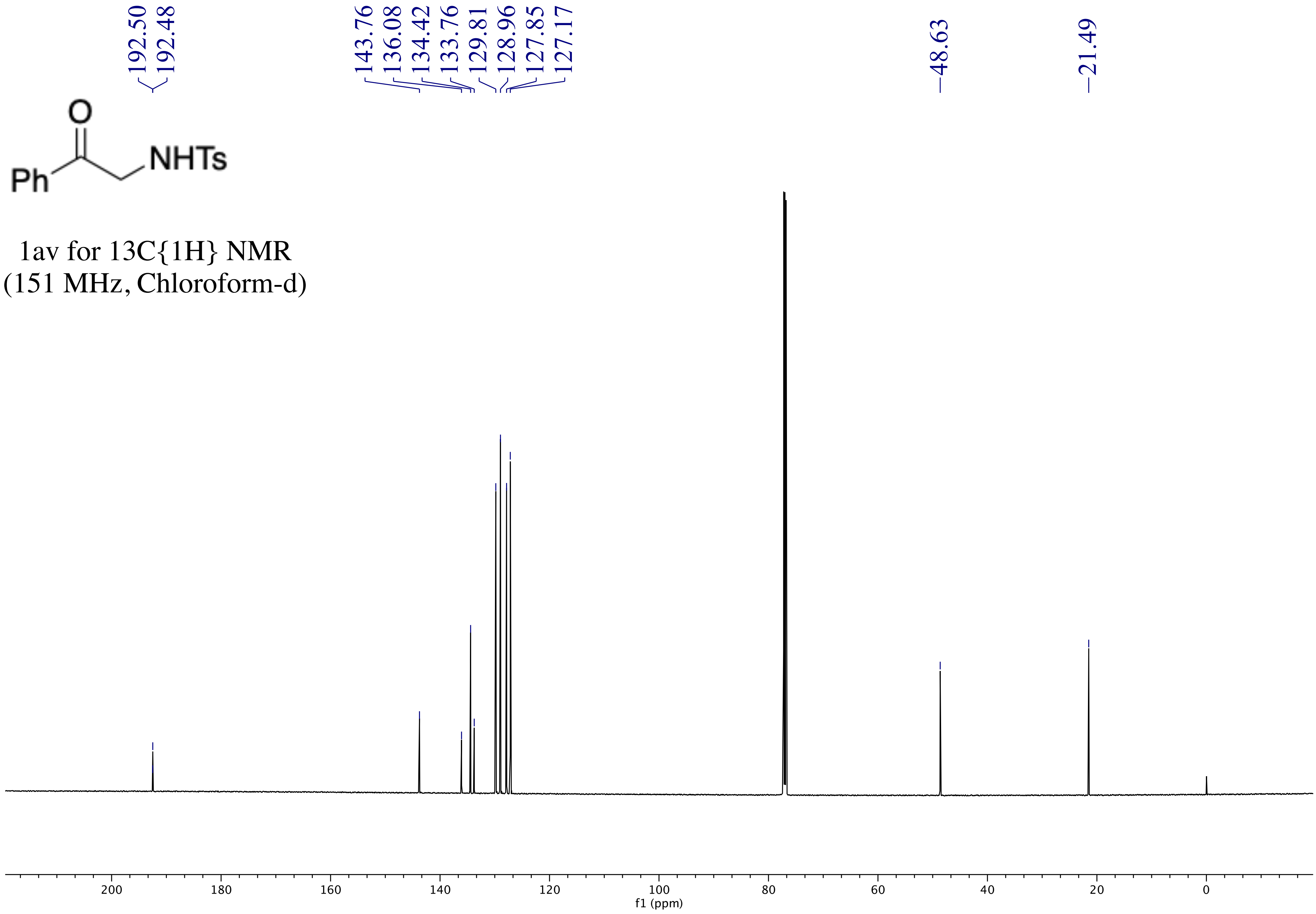
192.50
192.48
143.76
136.08
134.42
133.76
129.81
128.96
127.85
127.17
48.63
21.49
NHTs
Ph
O
1av for 13C{1H} NMR
(151 MHz, Chloroform-d)
200
180
160
140
120
100
80
60
40
20
0
f1 (ppm)

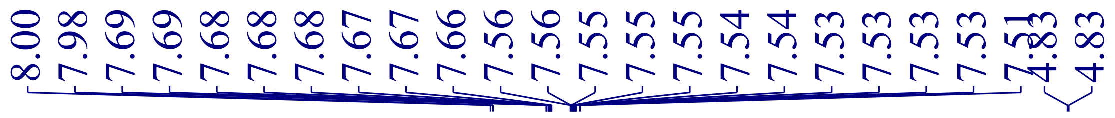

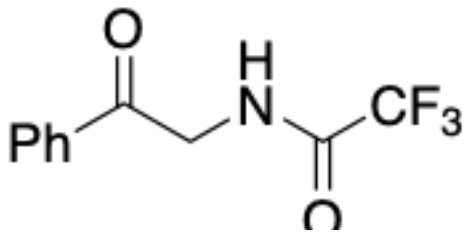

1aq for 1H NMR
(600 MHz, Chloroform-d)

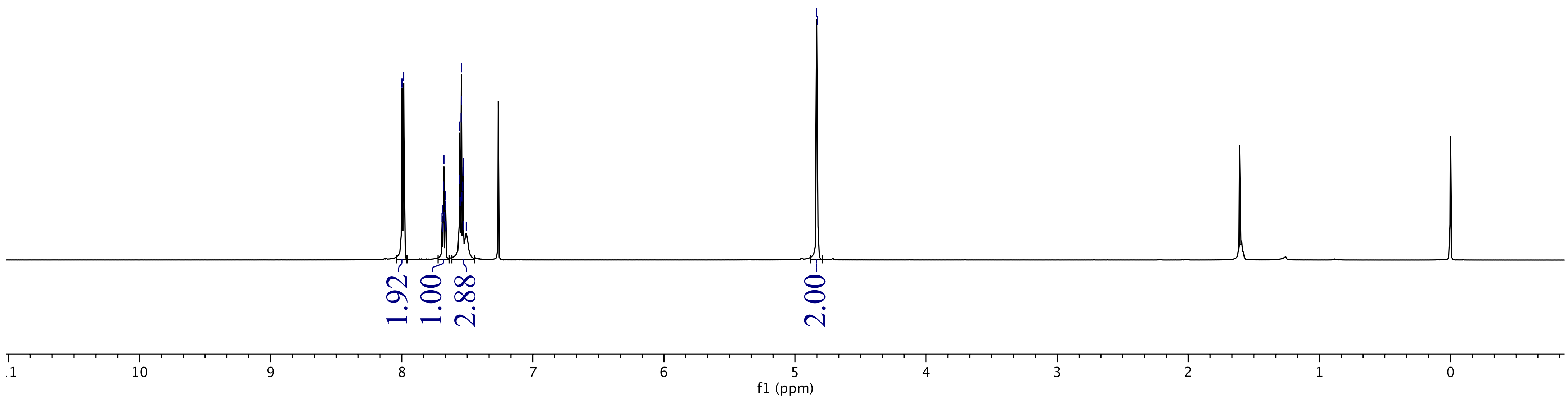

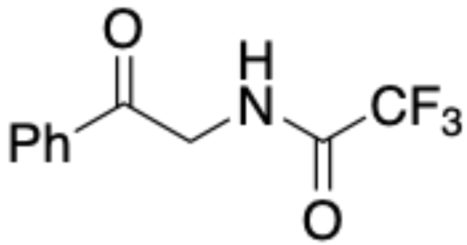

1aq for 13C{1H} NMR
(151 MHz, Chloroform-d)

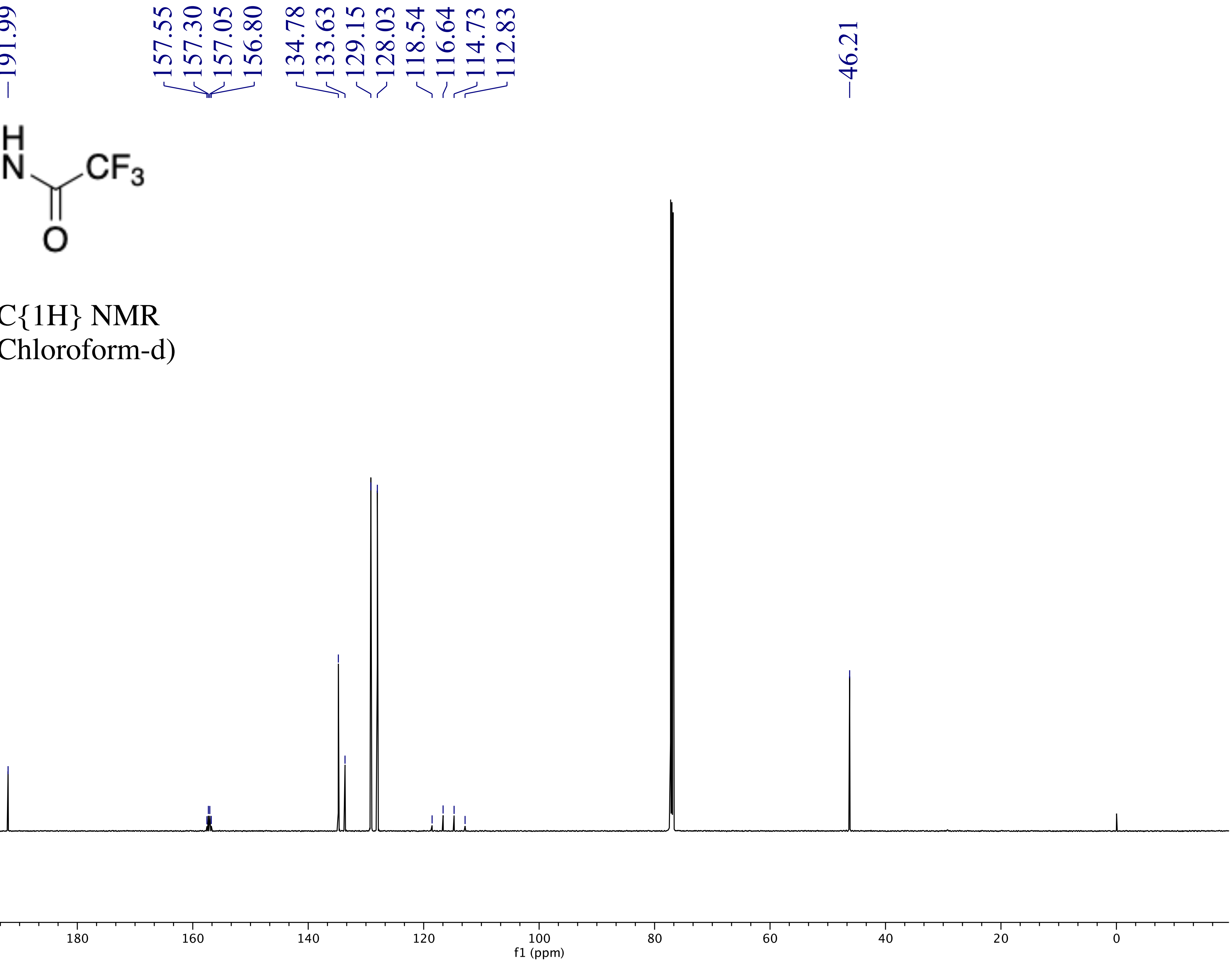

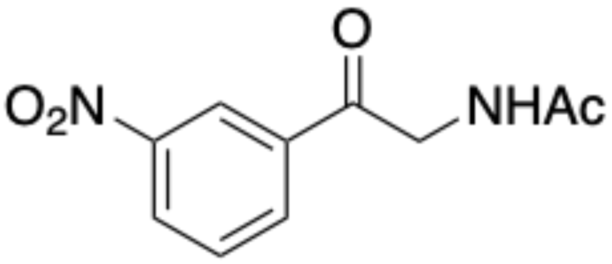

1bd for 1H NMR
(600 MHz, Chloroform-d)

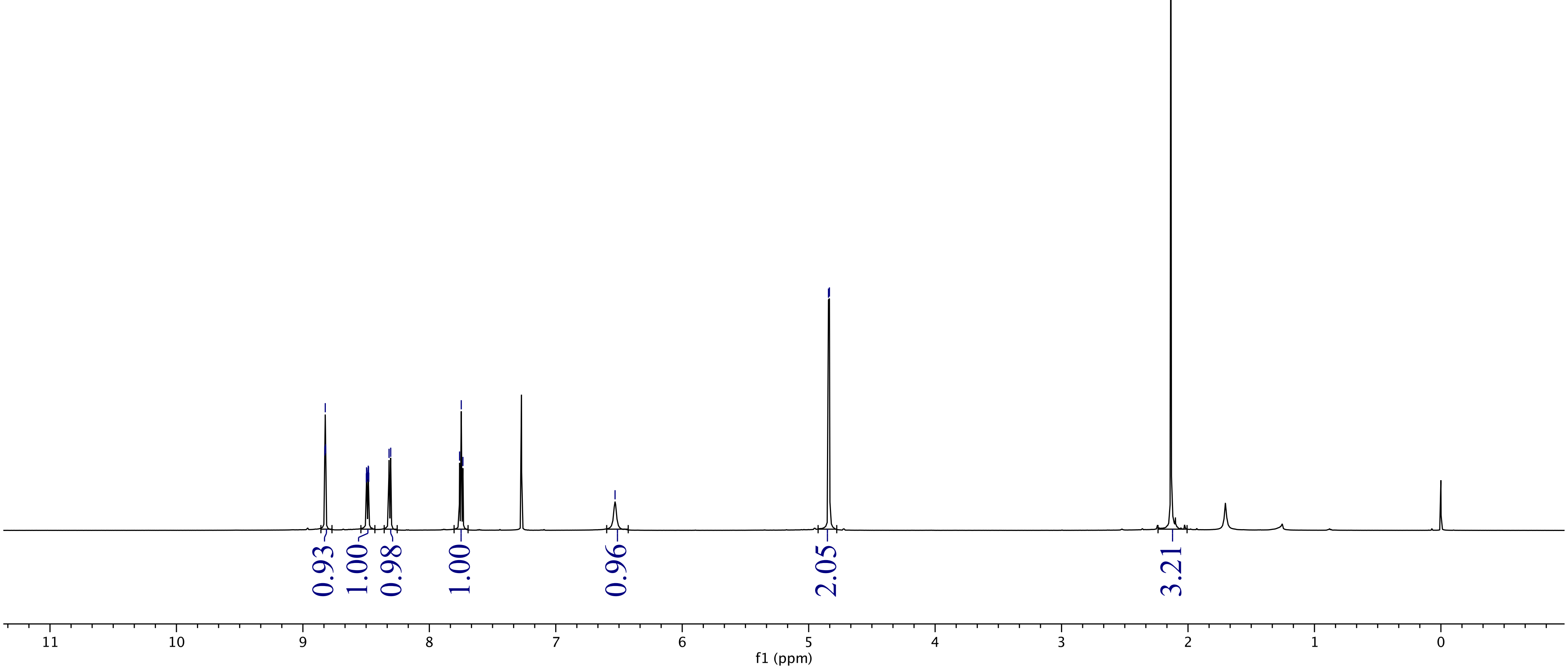

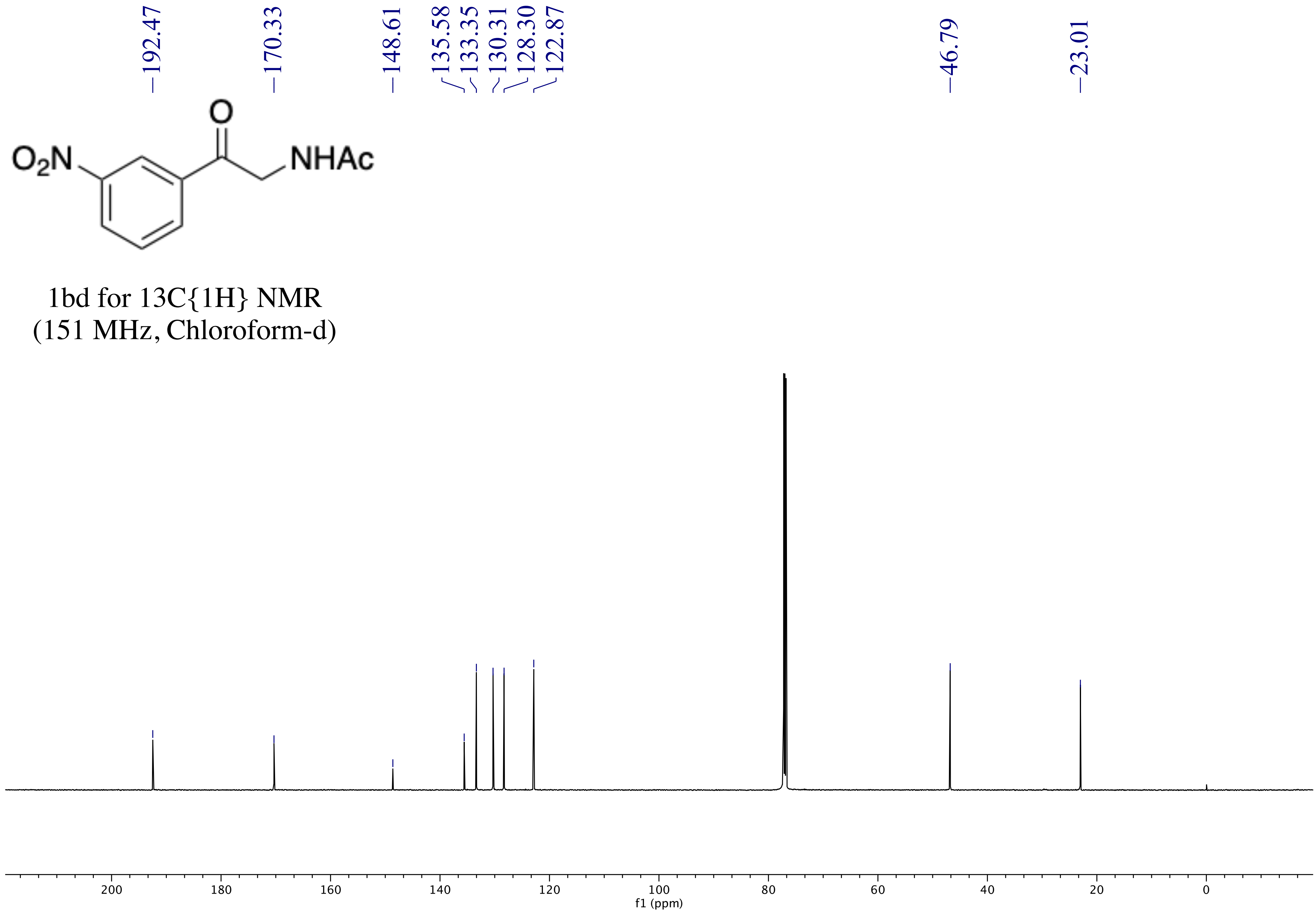

192.47
170.33
148.61
135.58
133.35
130.31
128.30
122.87
46.79
23.01
O2N
O
NHAc
1bd for 13C{1H} NMR
(151 MHz, Chloroform-d)
200
180
160
140
120
100
80
60
40
20
0
f1 (ppm)

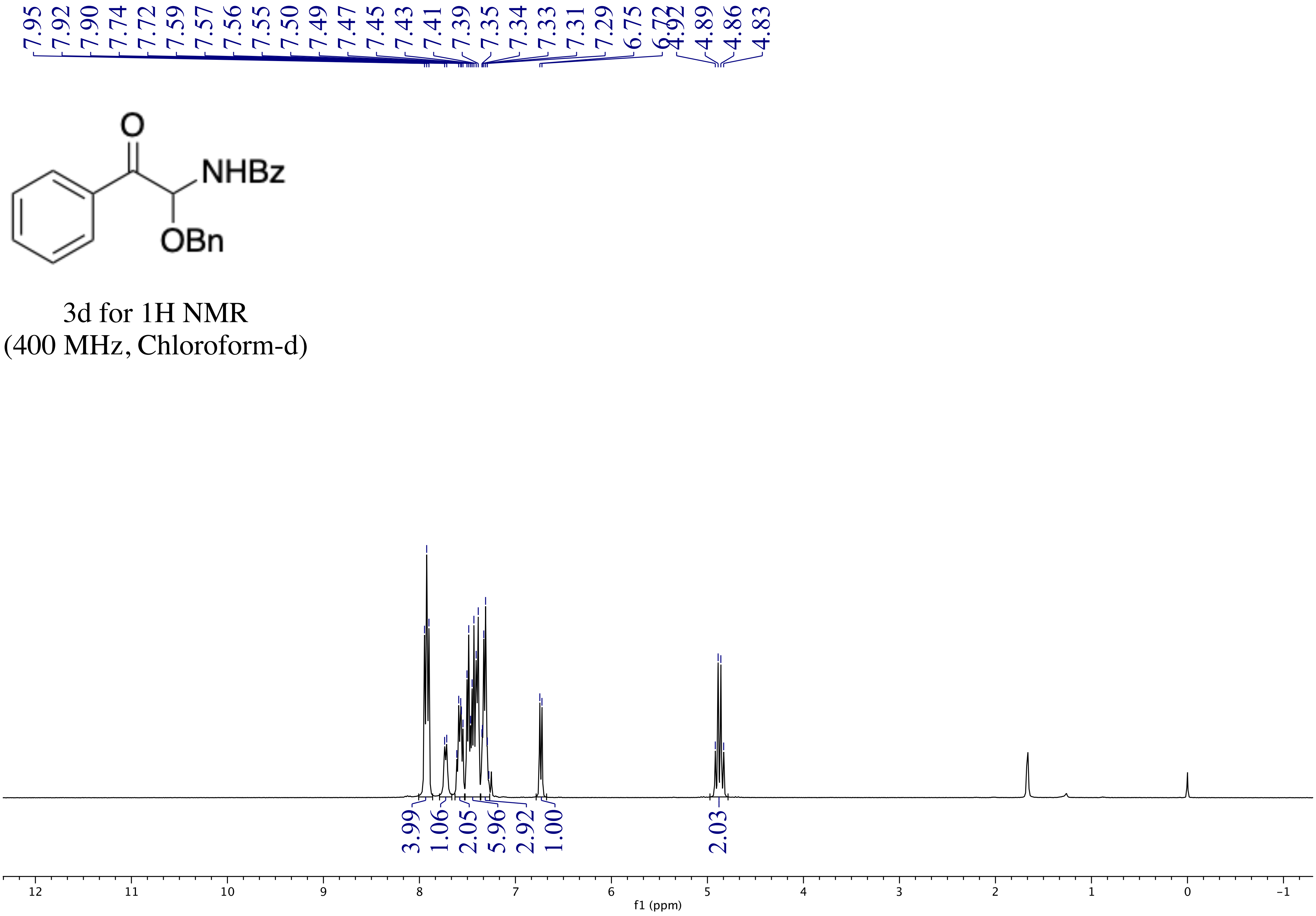
7.95 7.92 7.90 7.74 7.72 7.59 7.57 7.56 7.55 7.50 7.49 7.47 7.45 7.43 7.41 7.39 7.35 7.34 7.33 7.31 7.29 6.75 6.72 4.92 4.89 4.86 4.83
O
NHBz
OBn
3d for 1H NMR
(400 MHz, Chloroform-d)
3.99 1.06 2.05 5.96 2.92 1.00 2.03
12 11 10 9 8 7 6 5 4 3 2 1 0 -1
f1 (ppm)

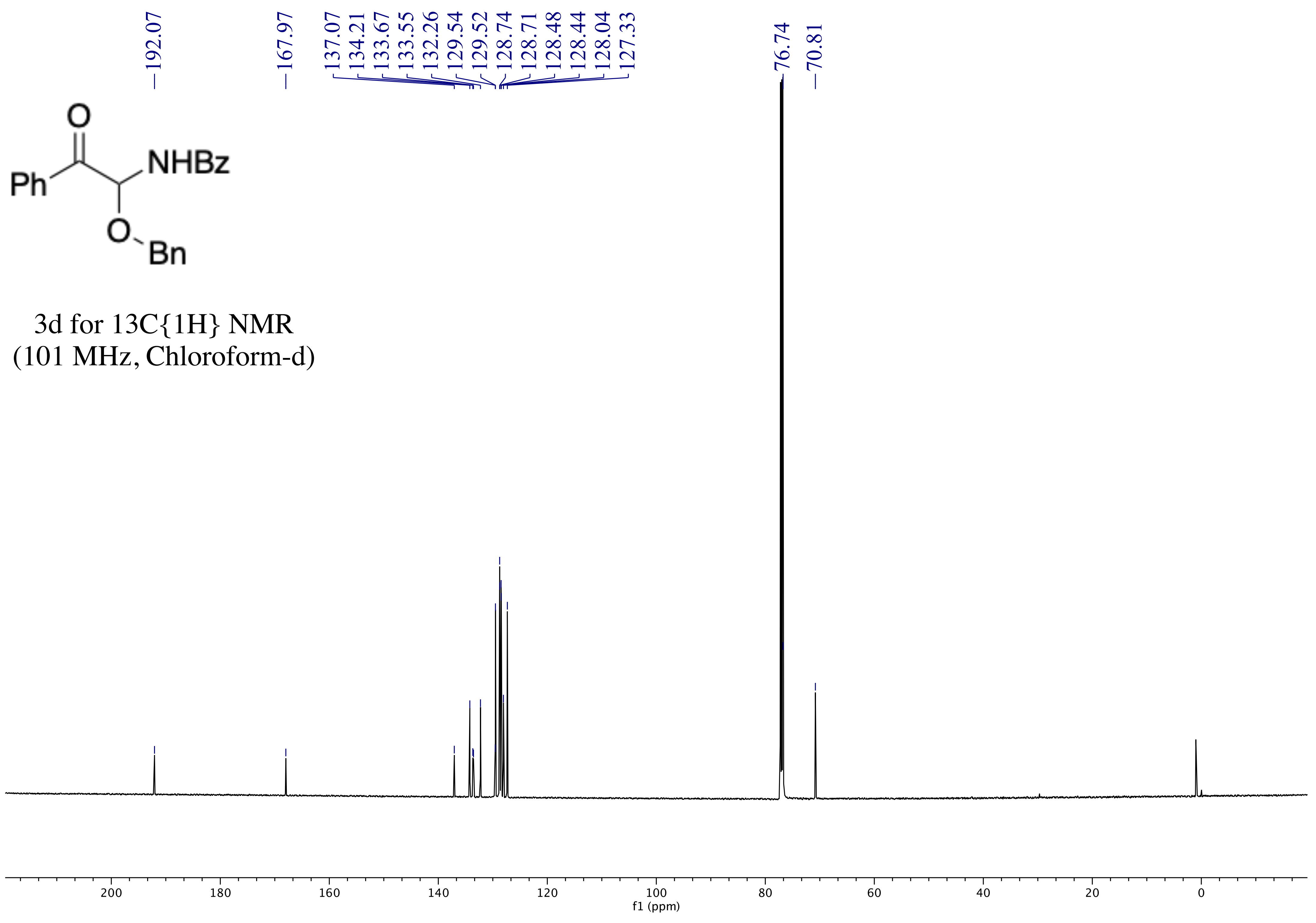

192.07
167.97
137.07
134.21
133.67
133.55
132.26
129.54
129.52
128.74
128.71
128.48
128.44
128.04
127.33
76.74
70.81
Ph
O
NHBz
O
Bn
3d for 13C{1H} NMR
(101 MHz, Chloroform-d)
200
180
160
140
120
100
80
60
40
20
0
f1 (ppm)

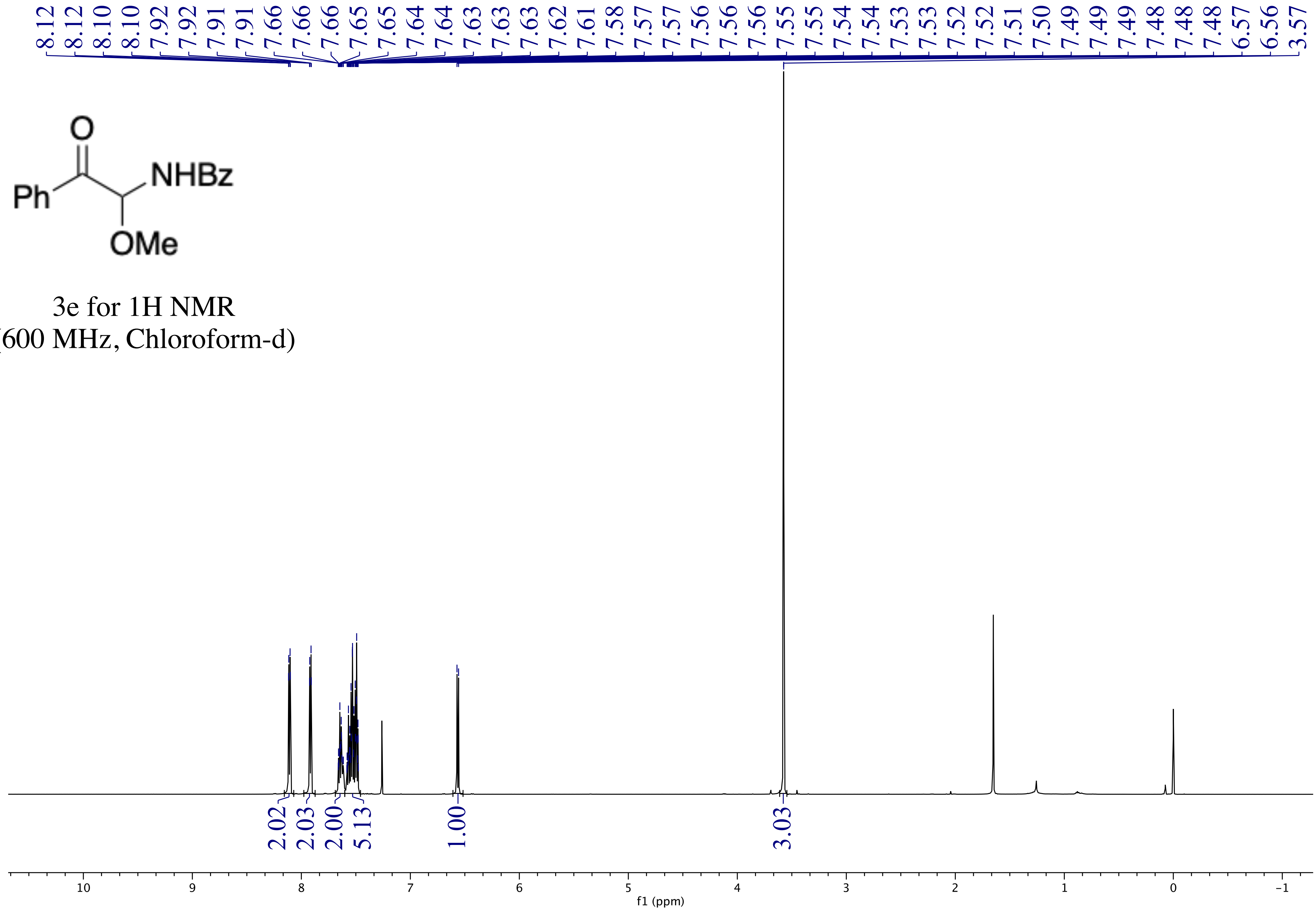

3e for 1H NMR
(600 MHz, Chloroform-d)

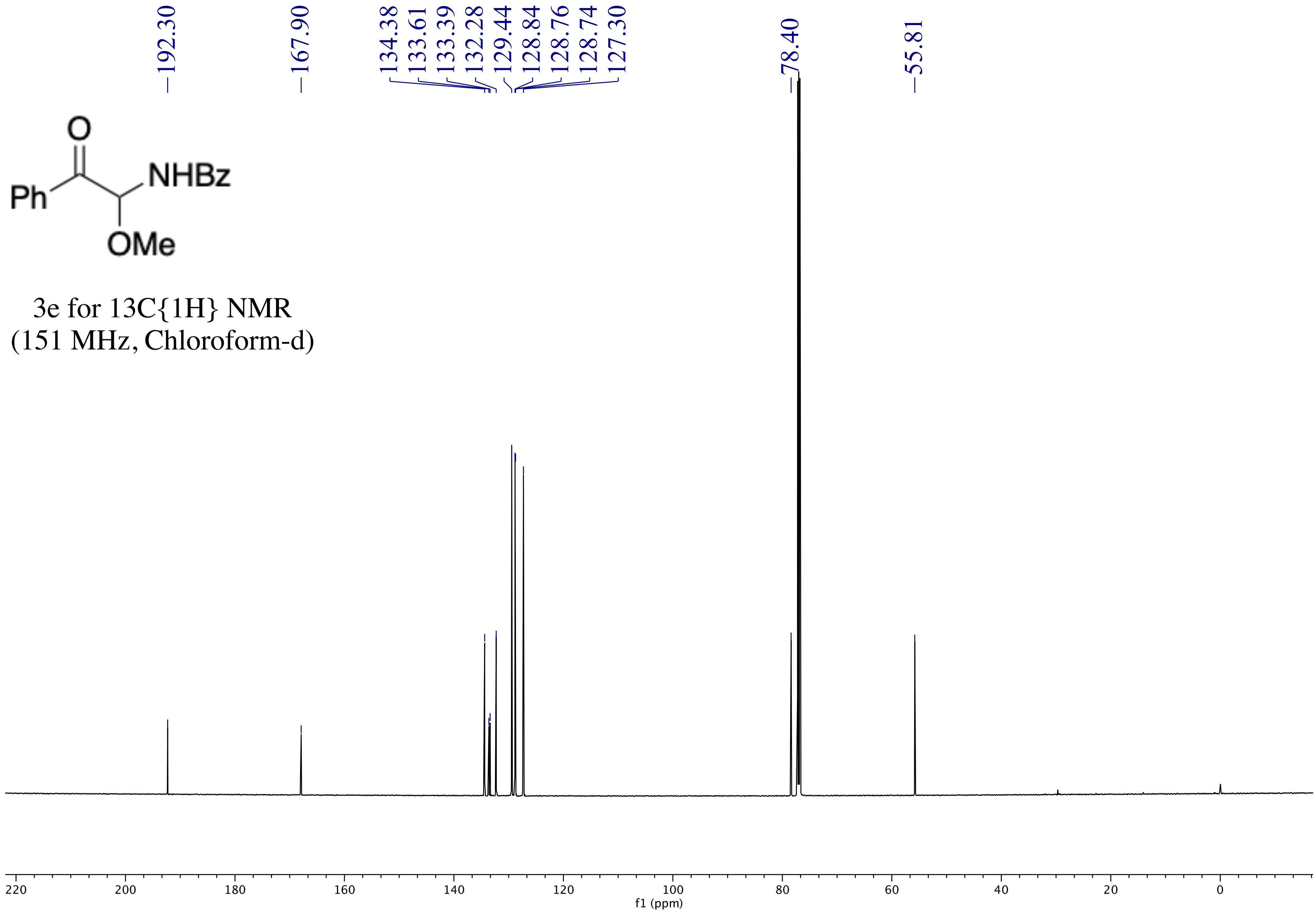
192.30
167.90
134.38
133.61
133.39
132.28
129.44
128.84
128.76
128.74
127.30
78.40
55.81
O
Ph
NHBz
OMe
3e for 13C{1H} NMR
(151 MHz, Chloroform-d)
220
200
180
160
140
120
100
80
60
40
20
0
f1 (ppm)

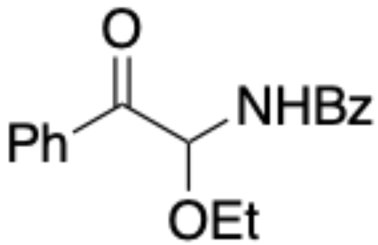

3f for 1H NMR
(600 MHz, Chloroform-d)

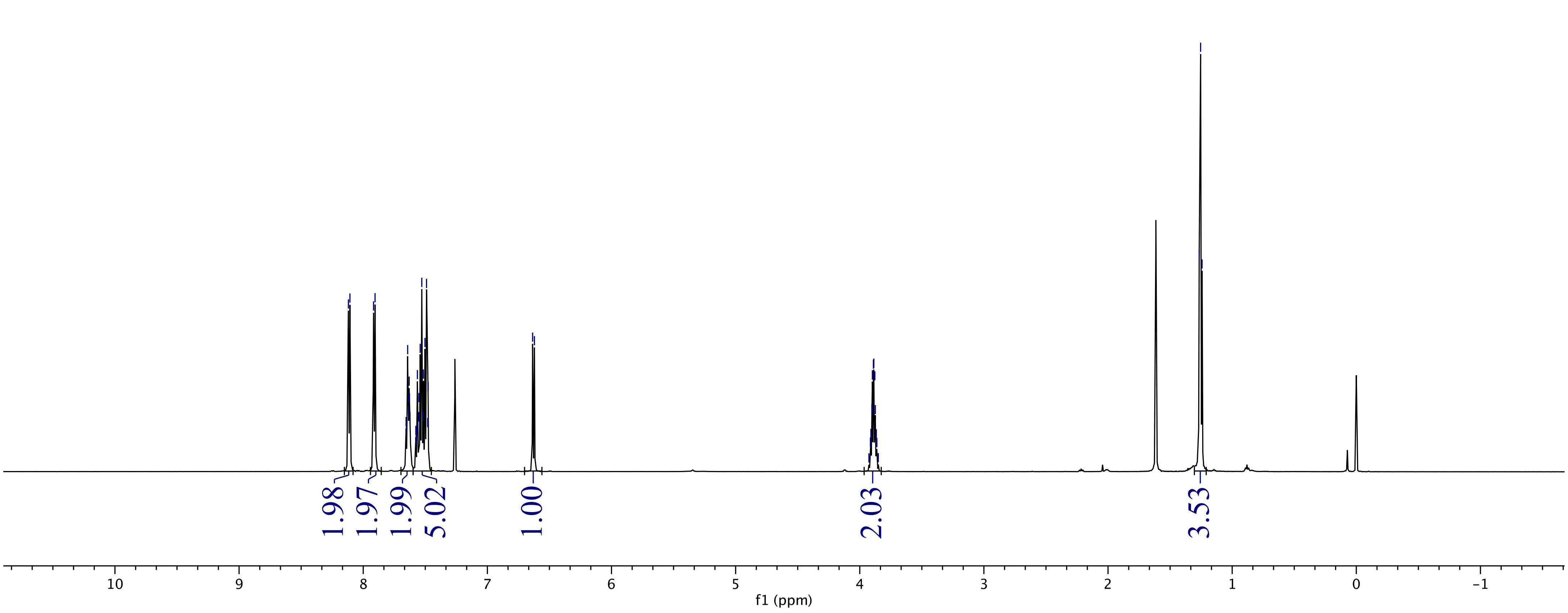

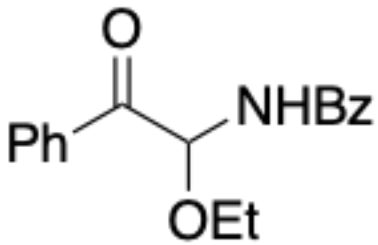

3f for 13C{1H} NMR
(151 MHz, Chloroform-d)

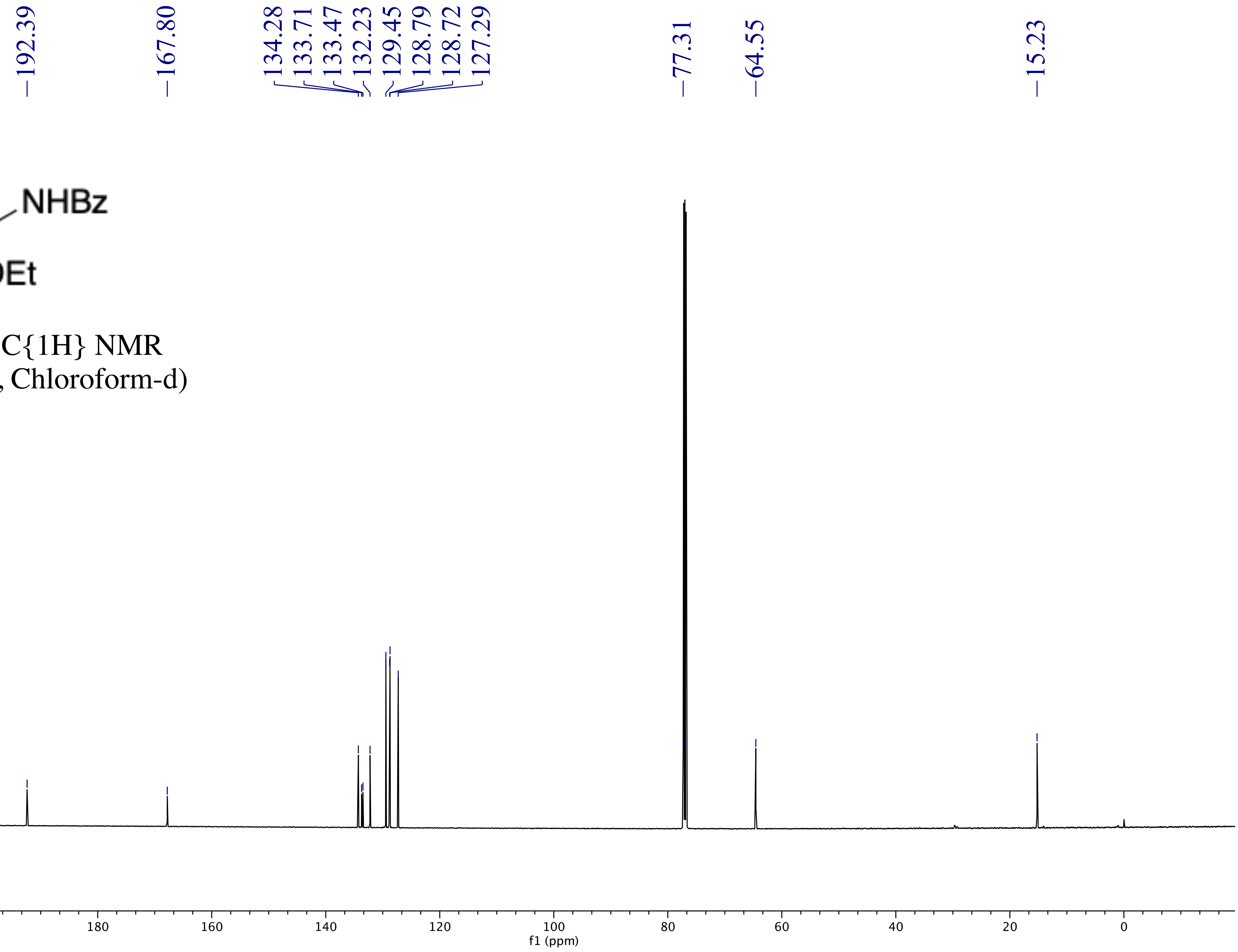

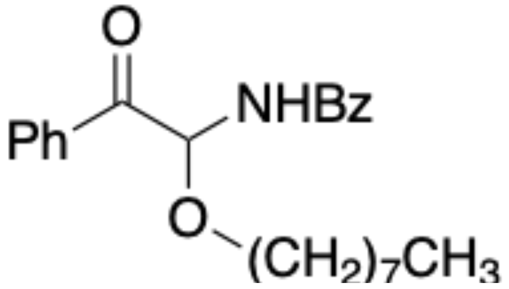

3g for 1H NMR
(600 MHz, Chloroform-d)

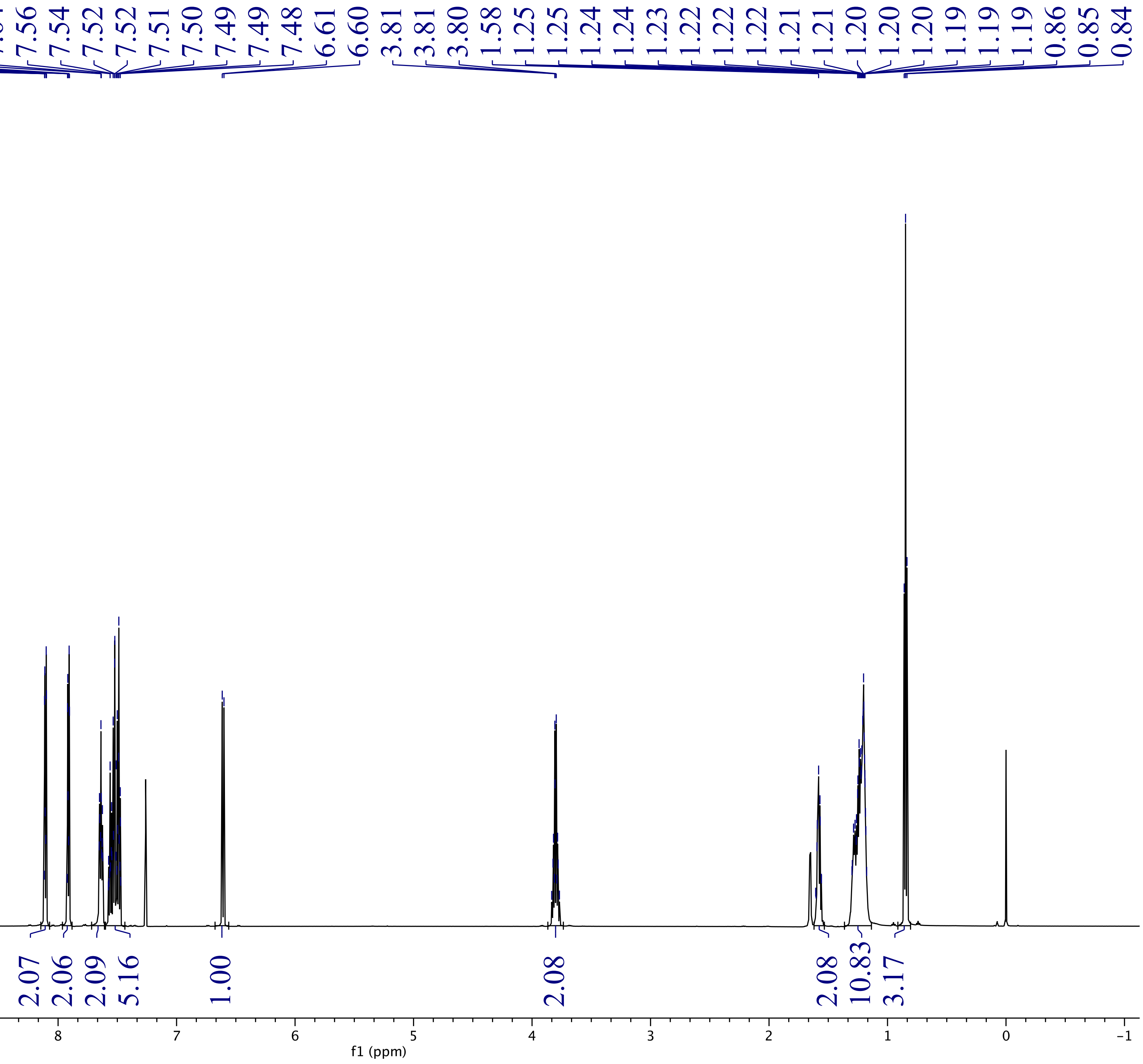

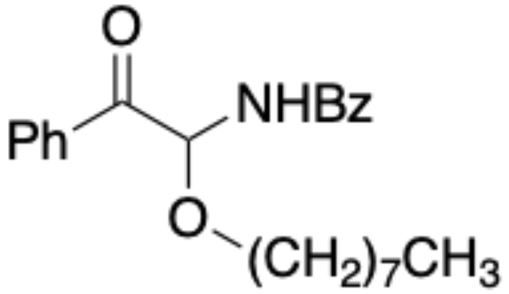

3g for 13C{1H} NMR
(151 MHz, Chloroform-d)

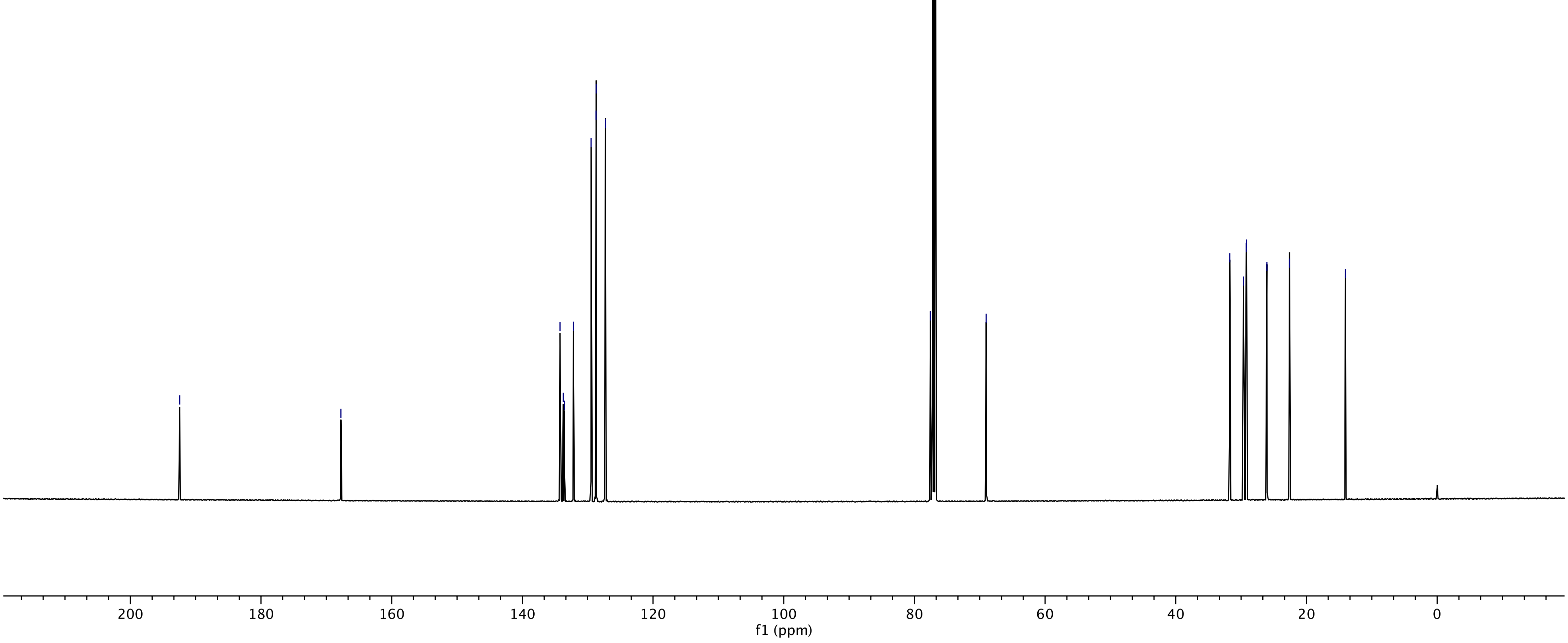

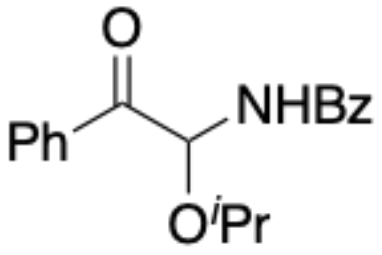

3h for 1H NMR
(600 MHz, Chloroform-d)

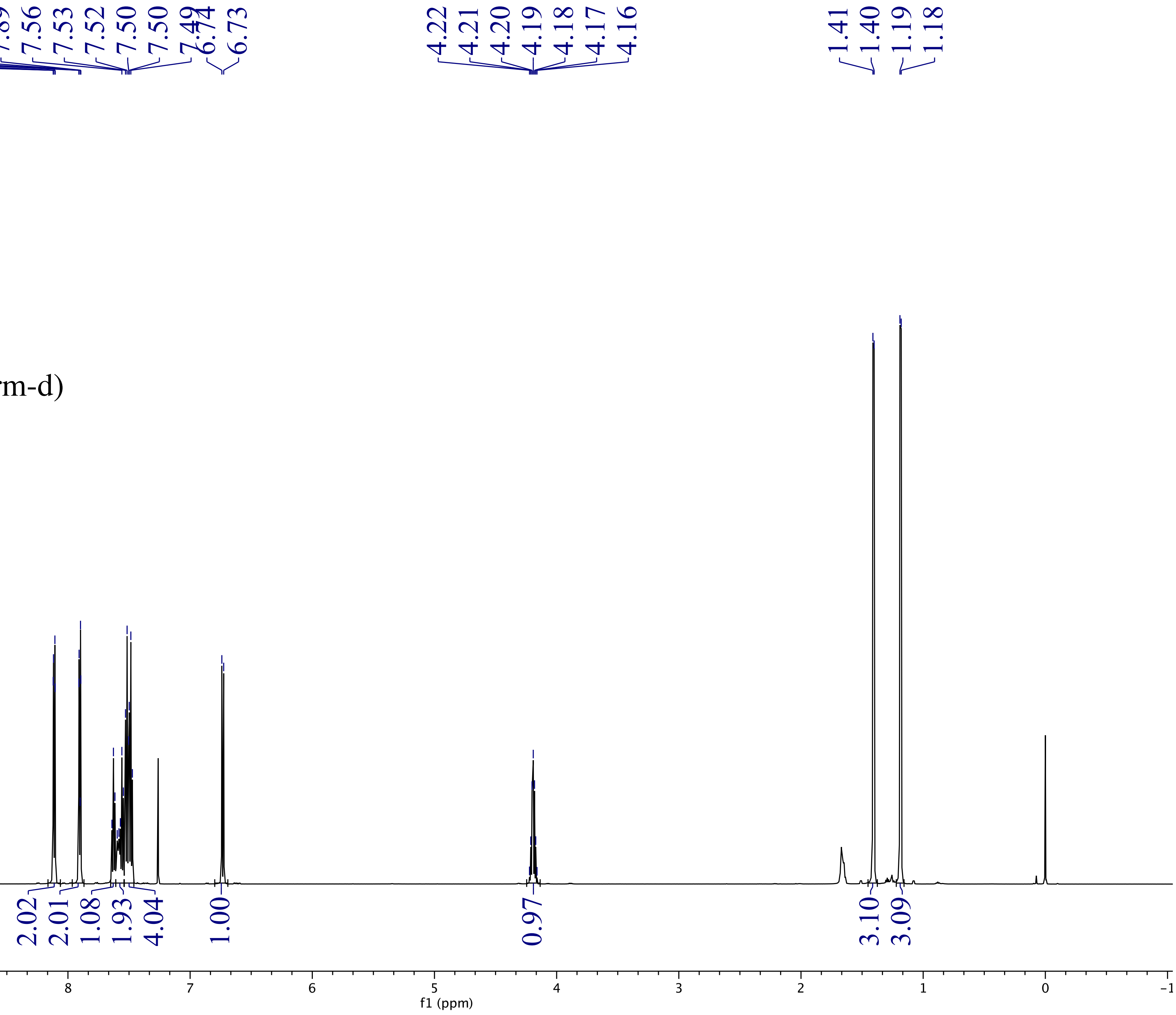

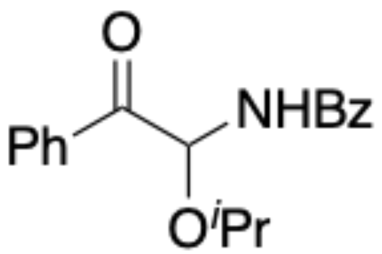

3h for 13C{1H} NMR
(151 MHz, Chloroform-d)

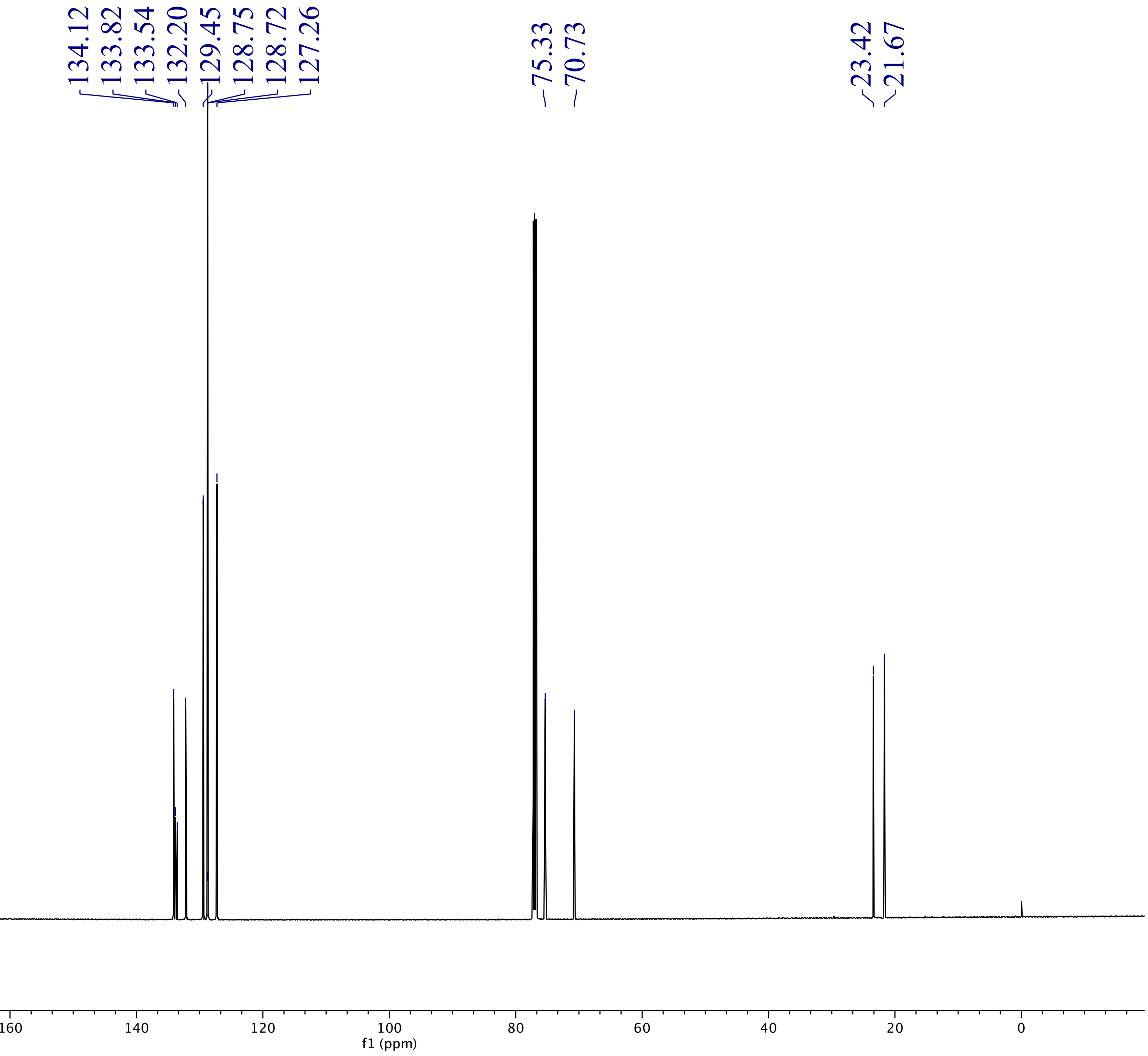

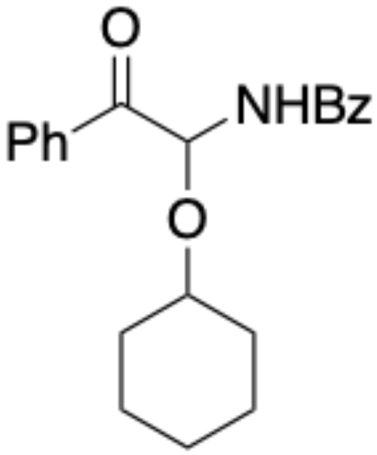

3i for 1H NMR
(600 MHz, Chloroform-d)

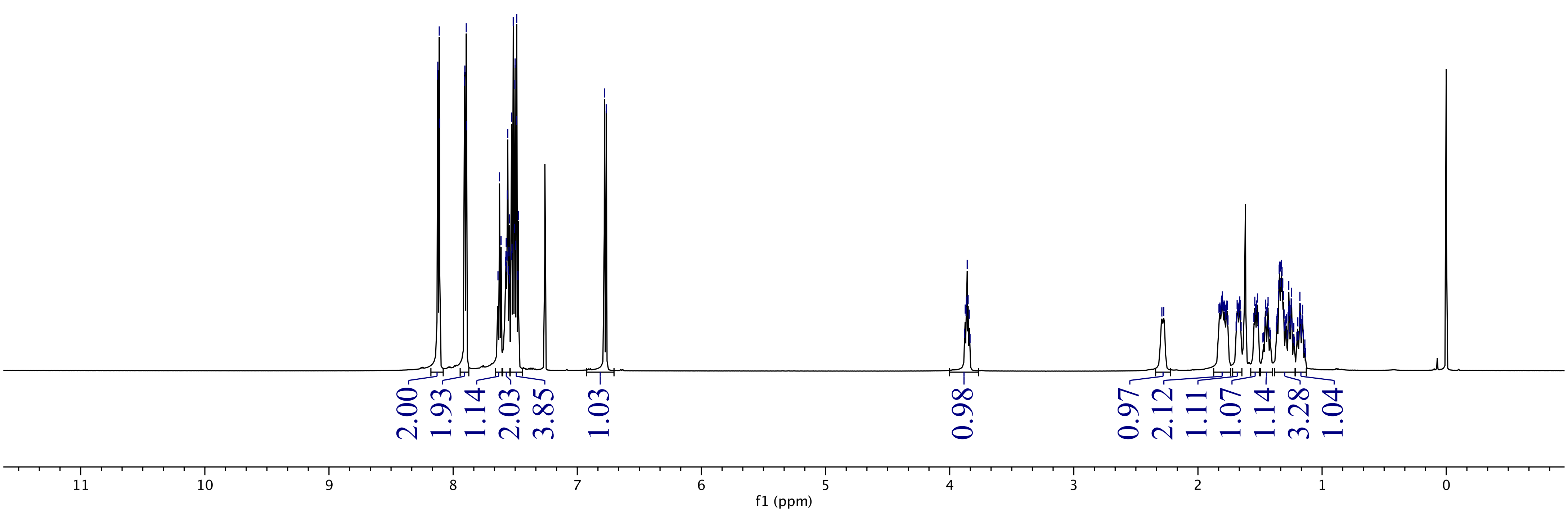

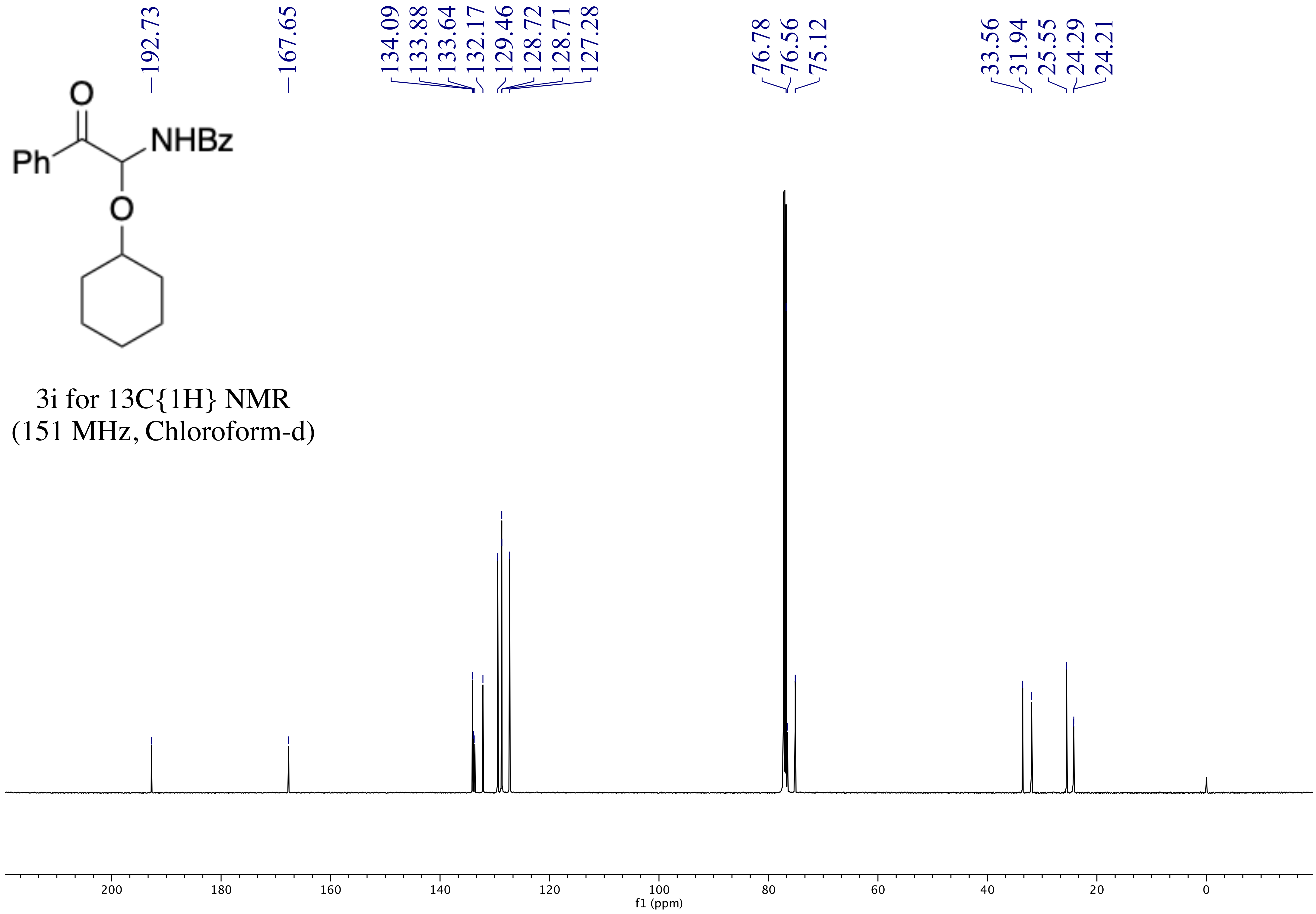

192.73
167.65
134.09
133.88
133.64
132.17
129.46
128.72
128.71
127.28
76.78
76.56
75.12
33.56
31.94
25.55
24.29
24.21
Ph
O
NHBz
O
3i for 13C{1H} NMR
(151 MHz, Chloroform-d)
200
180
160
140
120
100
80
60
40
20
0
f1 (ppm)

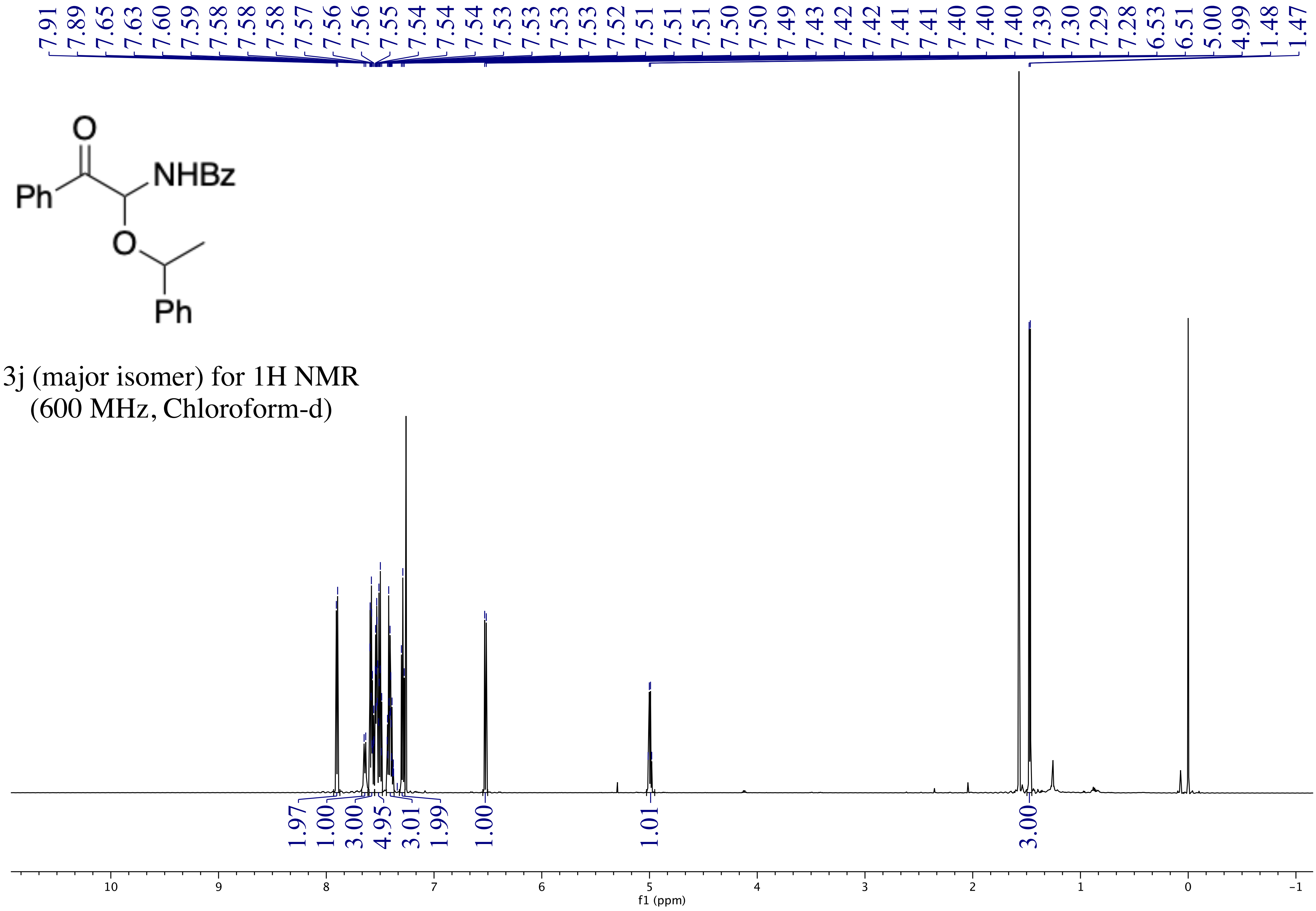

7.91 7.89 7.65 7.63 7.60 7.59 7.58 7.58 7.58 7.57 7.56 7.56 7.55 7.54 7.54 7.54 7.53 7.53 7.53 7.53 7.52 7.51 7.51 7.51 7.50 7.50 7.49 7.43 7.42 7.42 7.41 7.41 7.40 7.40 7.40 7.39 7.30 7.29 7.28 6.53 6.51 5.00 4.99 1.48 1.47
Ph
O
NHBz
O
Ph
3j (major isomer) for 1H NMR
(600 MHz, Chloroform-d)
1.97 1.00 3.00 4.95 3.01 1.99 1.00 1.01 3.00
10 9 8 7 6 5 4 3 2 1 0 -1
f1 (ppm)

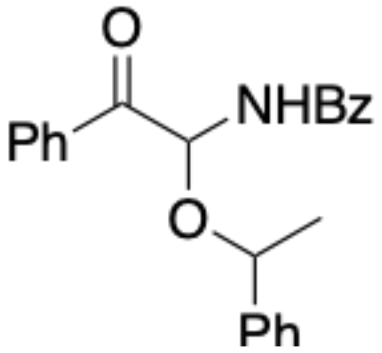

3j (major isomer) for 13C{1H} NMR
(151 MHz, Chloroform-d)

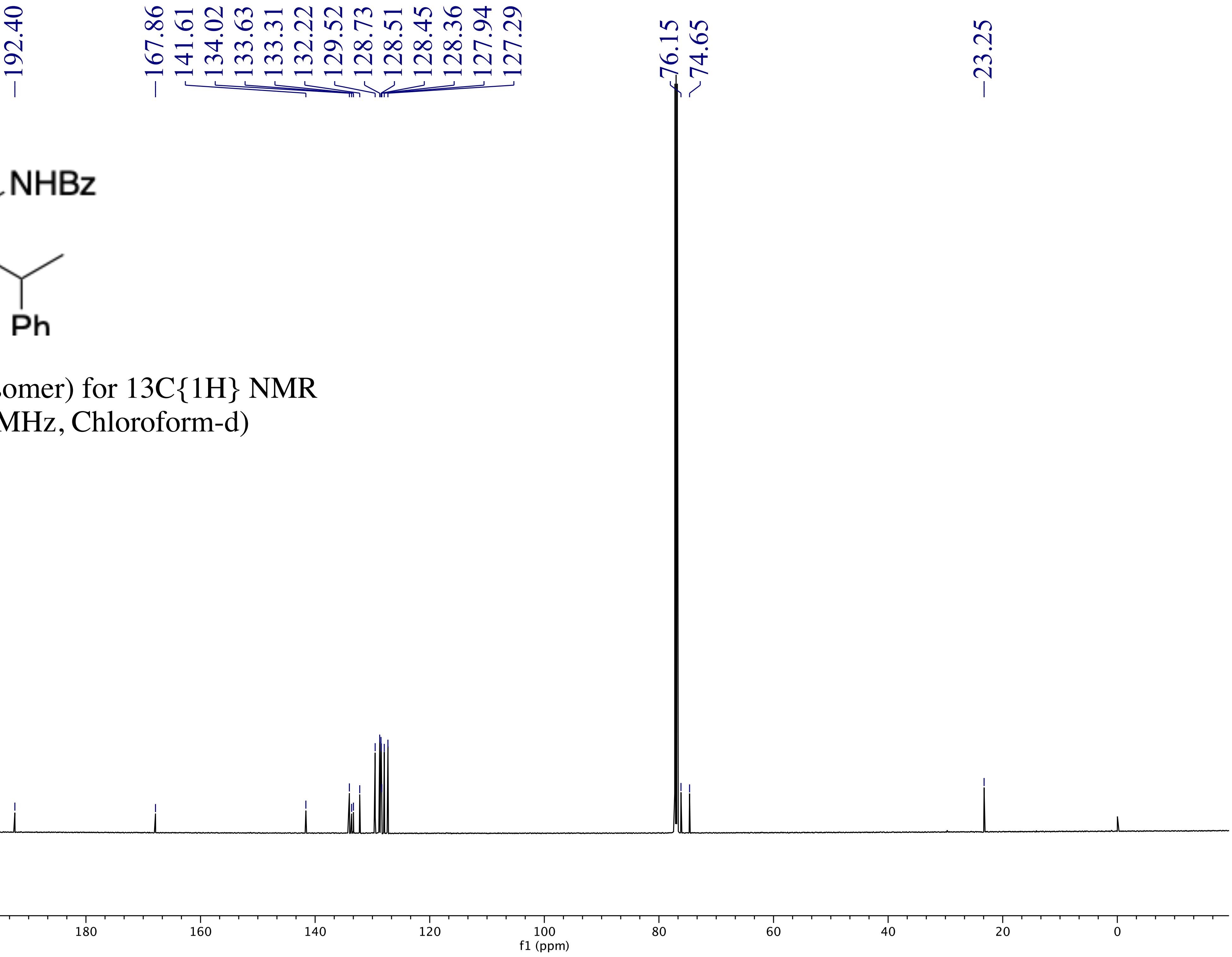

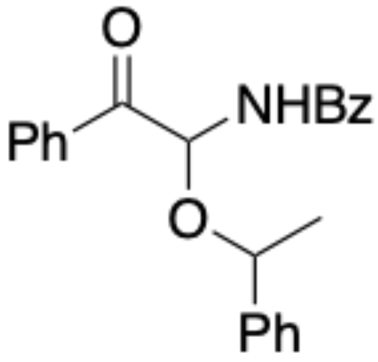

3j (minor isomer) for 1H NMR
(600 MHz, Chloroform-d)

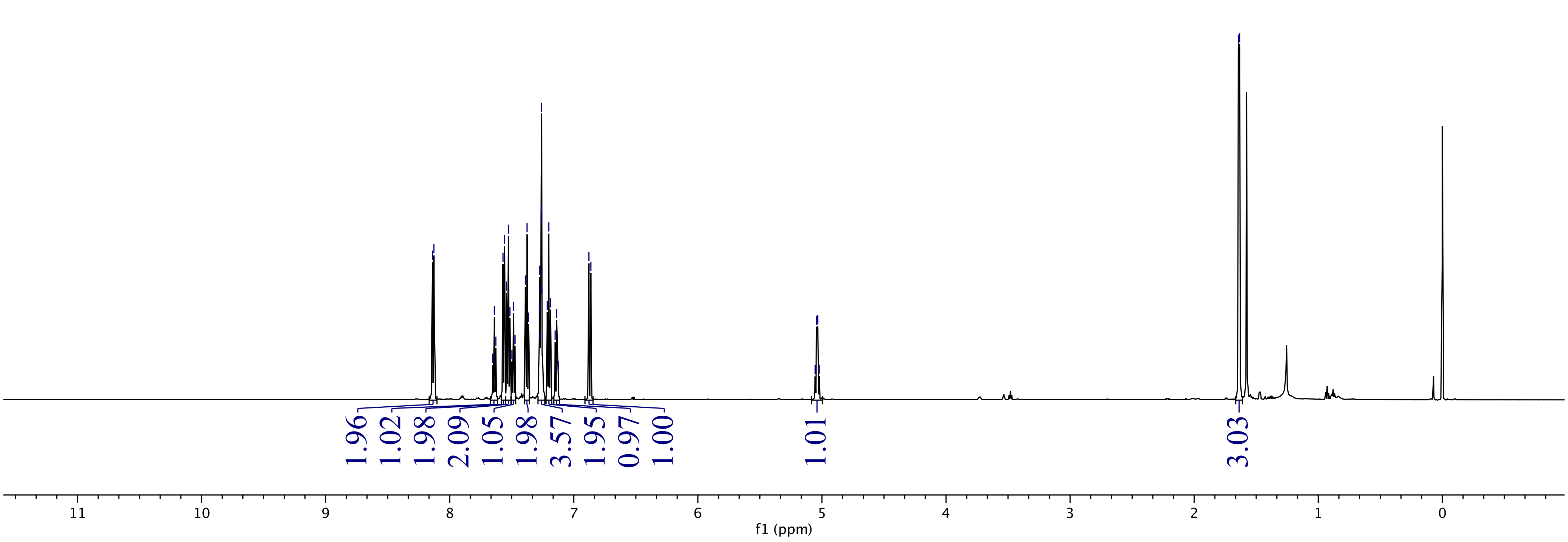

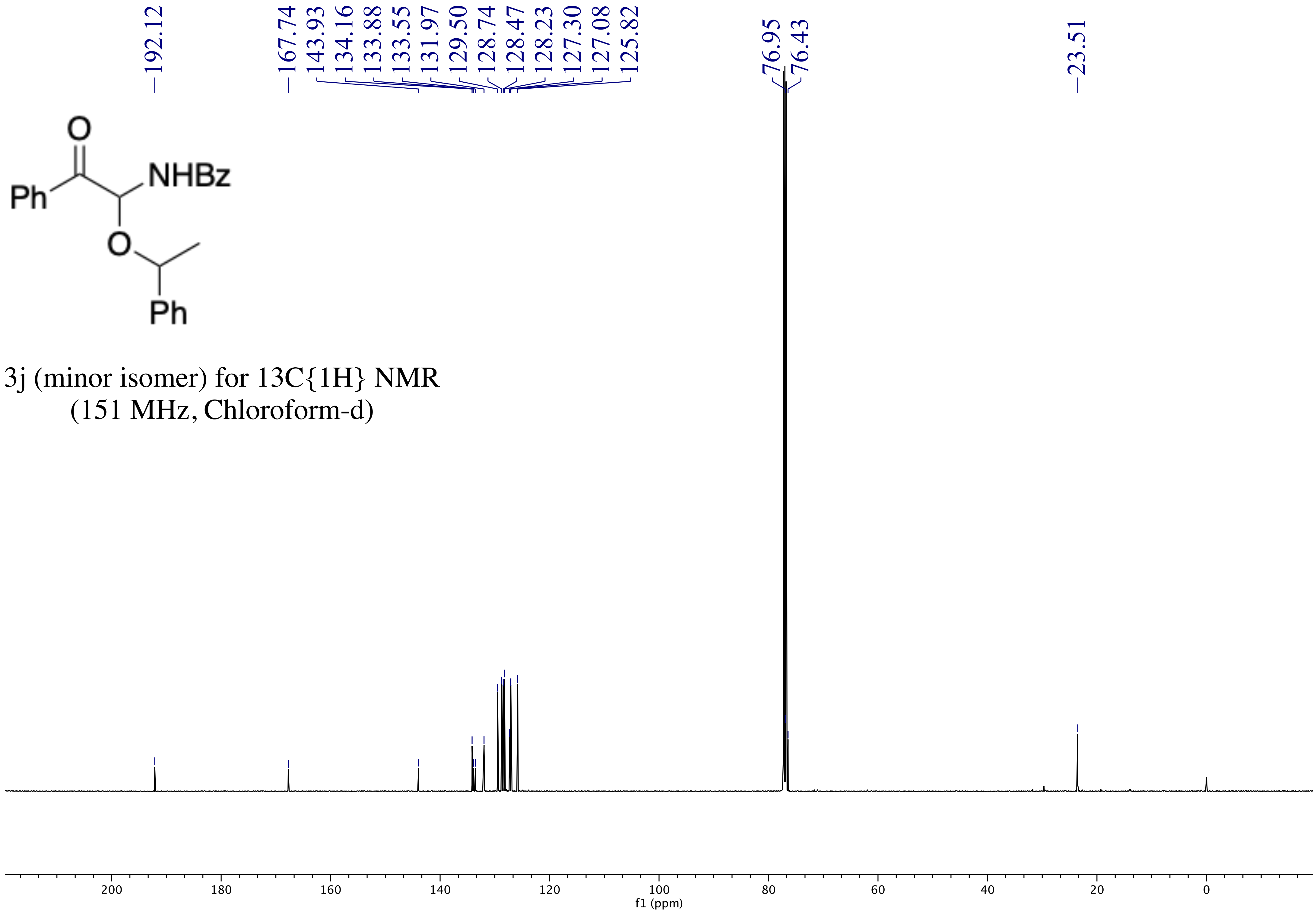
192.12
167.74
143.93
134.16
133.88
133.55
131.97
129.50
128.74
128.47
128.23
127.30
127.08
125.82
76.95
76.43
23.51
O
NHBz
Ph
O
Ph
3j (minor isomer) for 13C{1H} NMR
(151 MHz, Chloroform-d)
200
180
160
140
120
100
80
60
40
20
0
f1 (ppm)

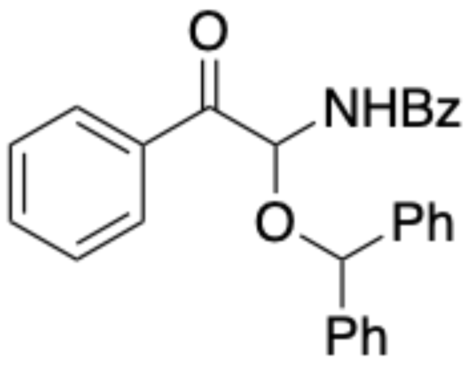

3k for 1H NMR
(400 MHz, Chloroform-d)

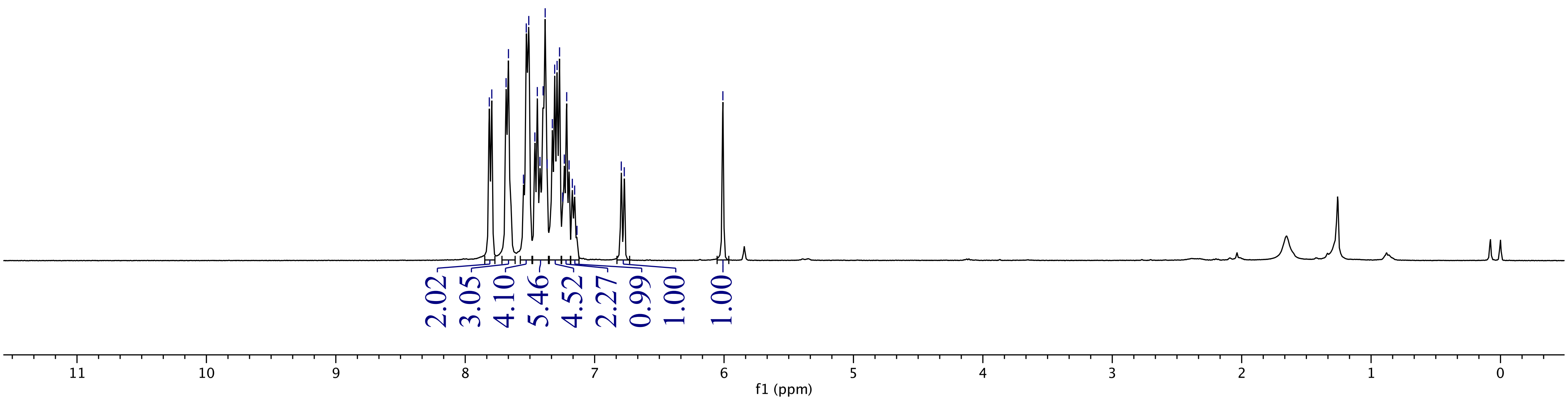

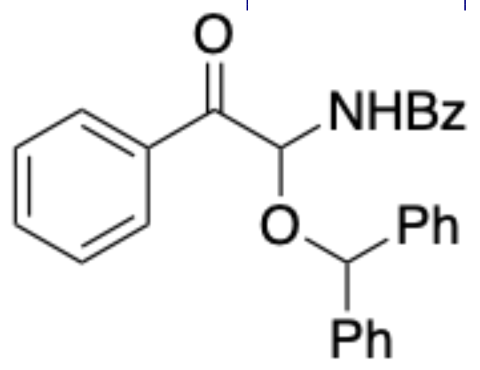

3k for 13C{1H} NMR
(101 MHz, Chloroform-d))

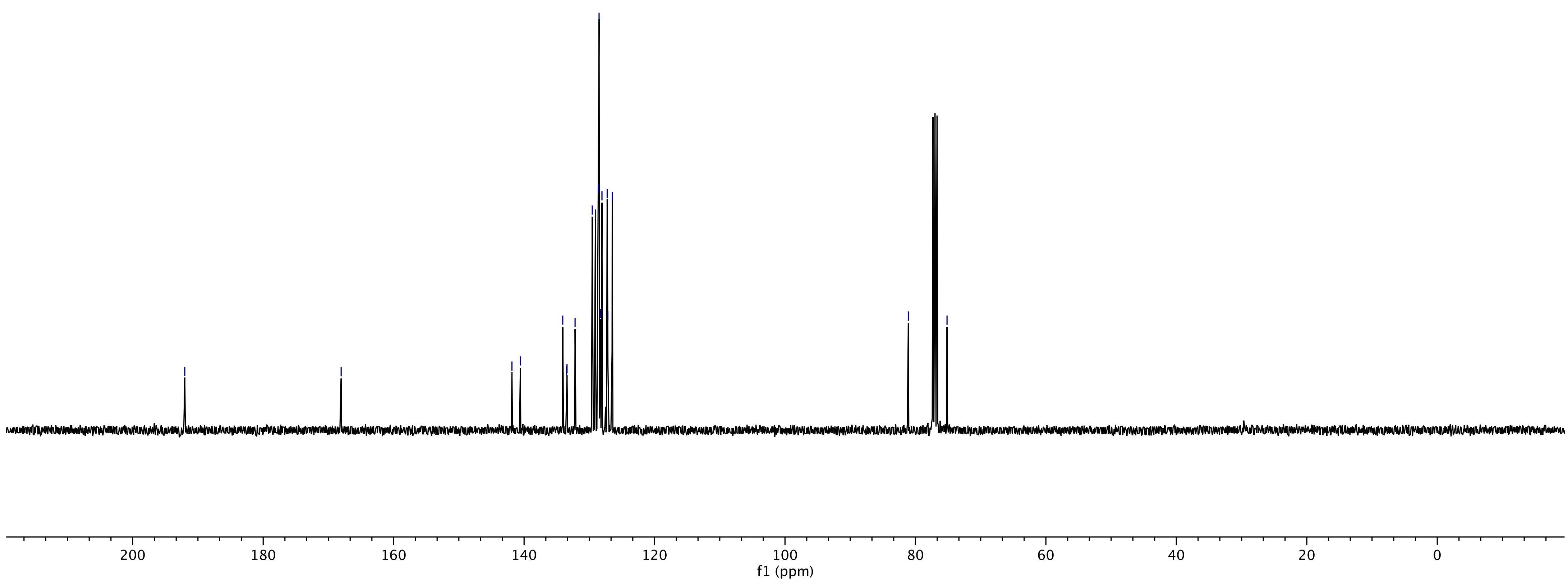

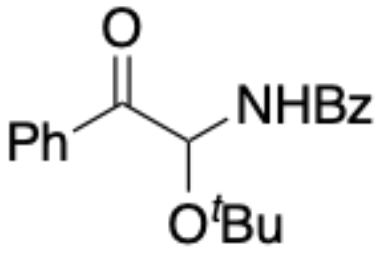

3l for 1H NMR
(600 MHz, Chloroform-d)

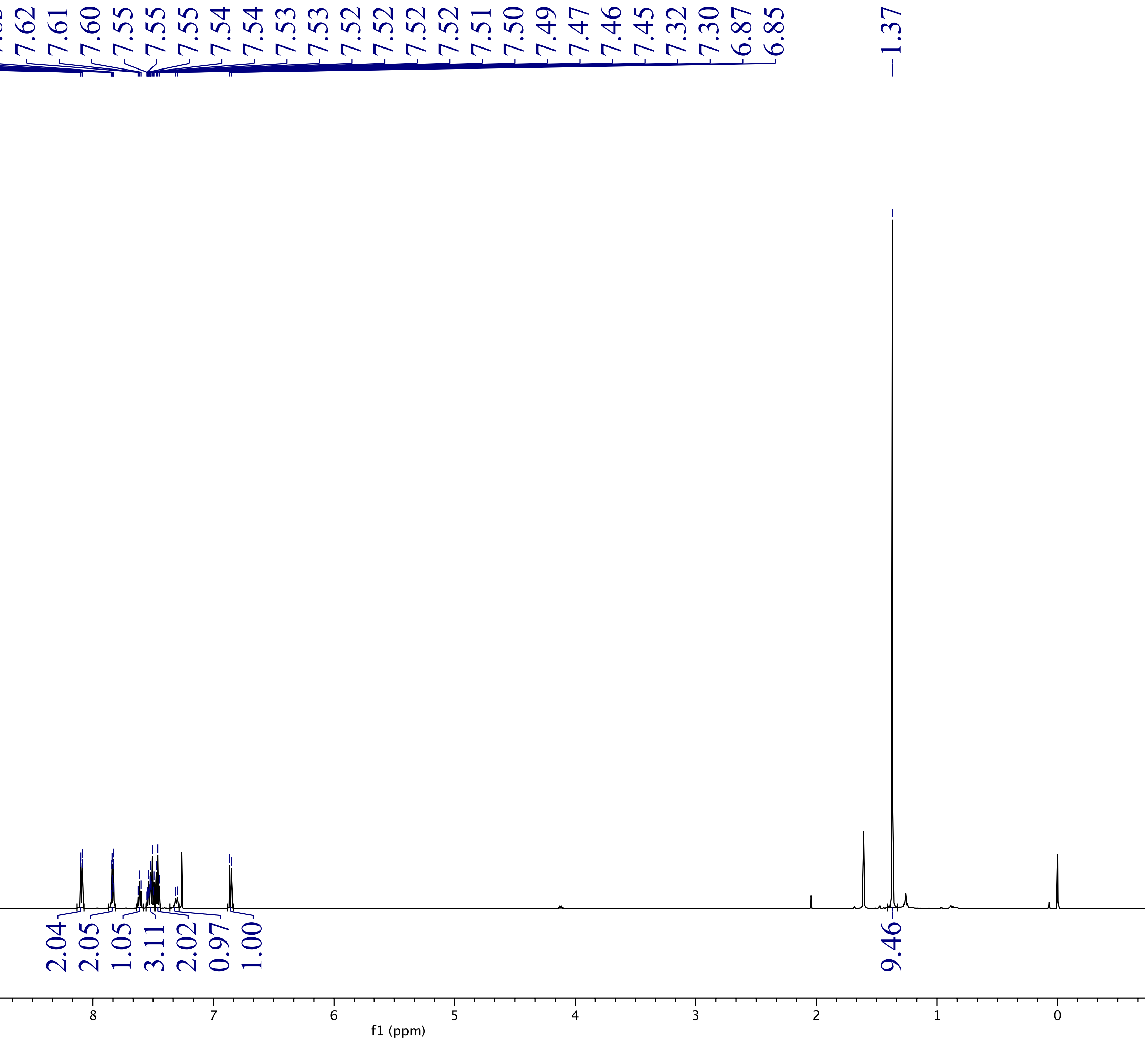

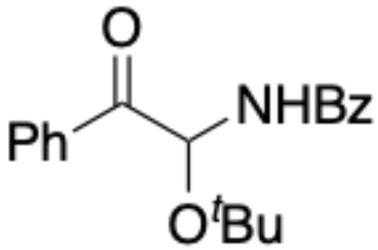

3l for 13C{1H} NMR
(151 MHz, Chloroform-d)

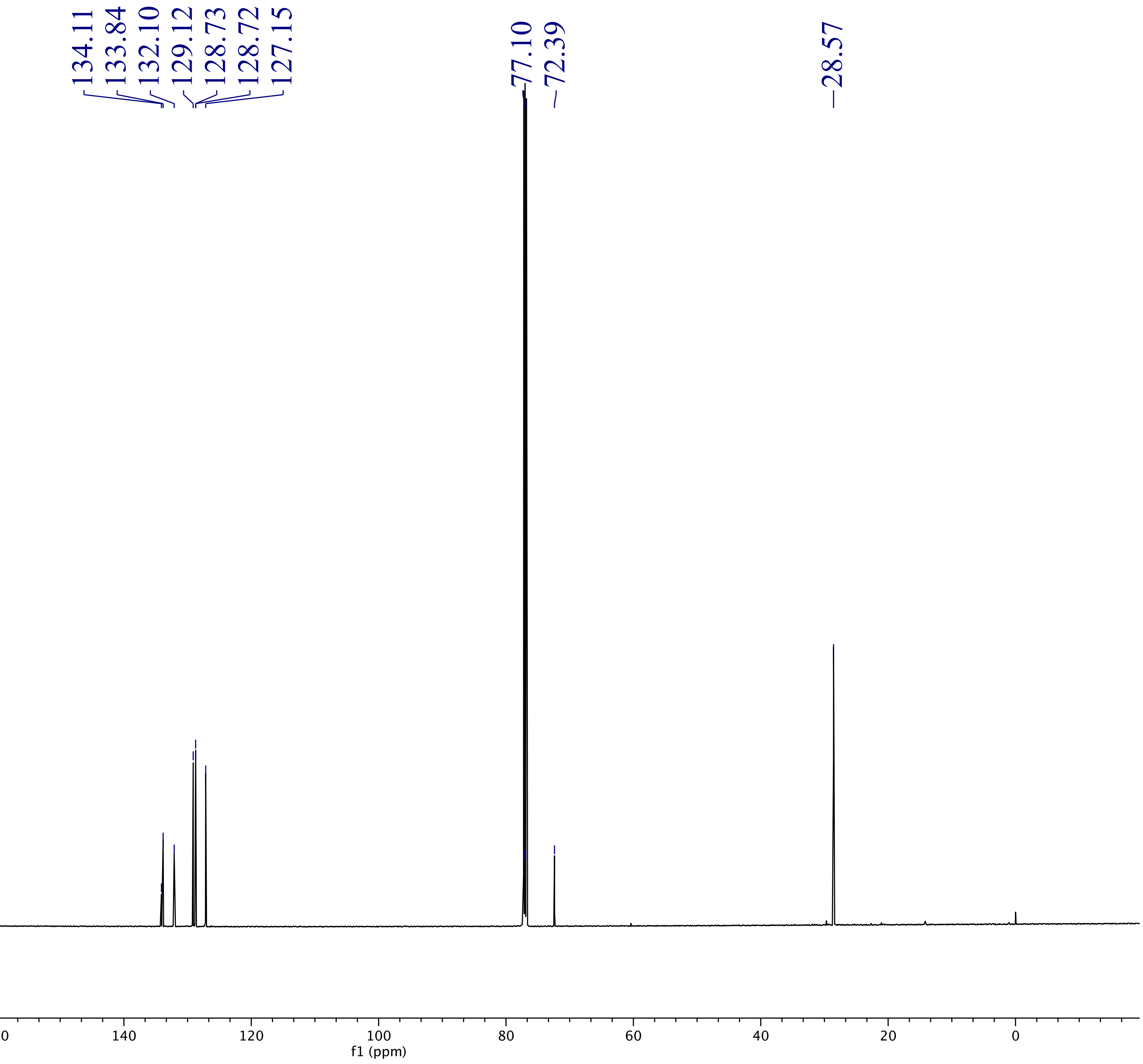

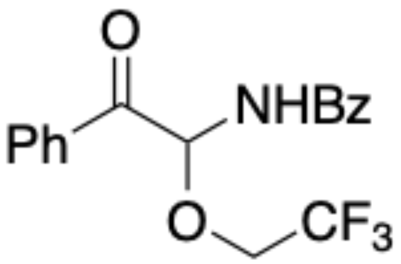

3m for 1H NMR
(600 MHz, Chloroform-d)

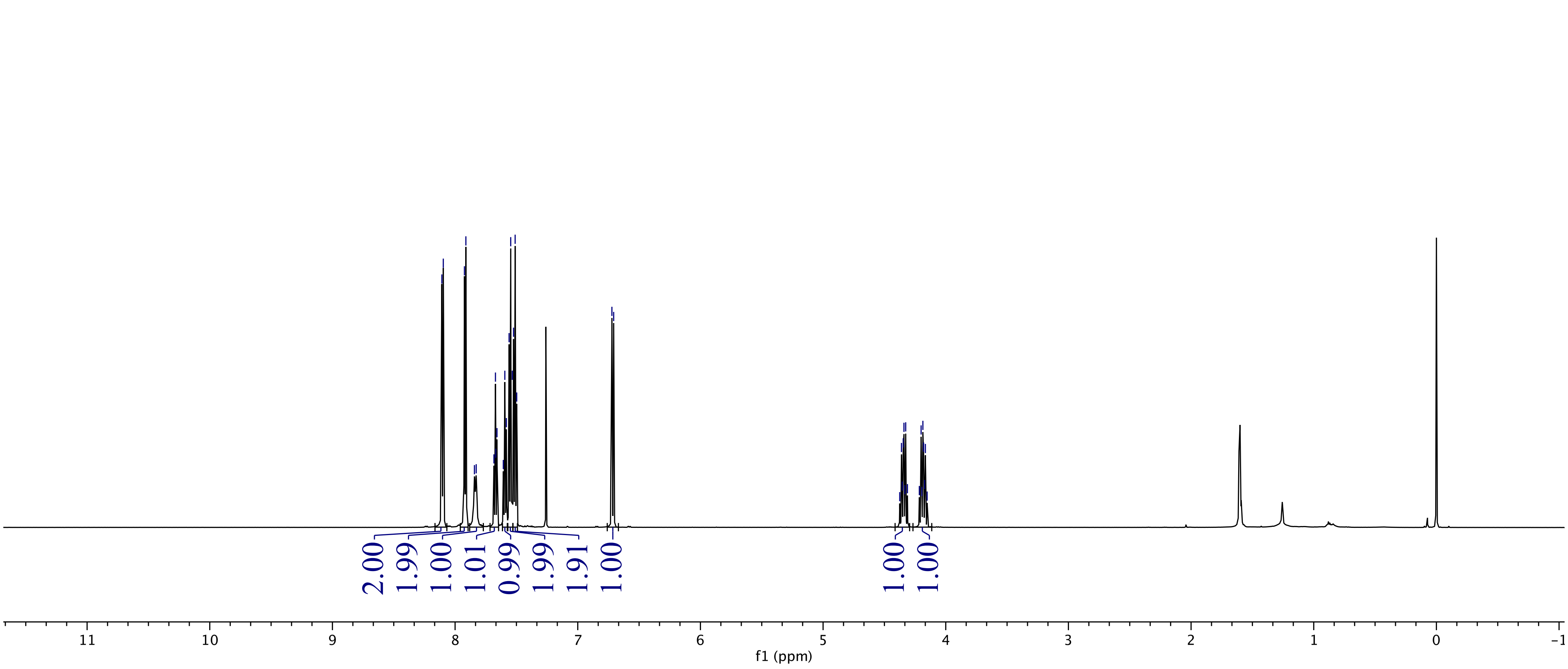

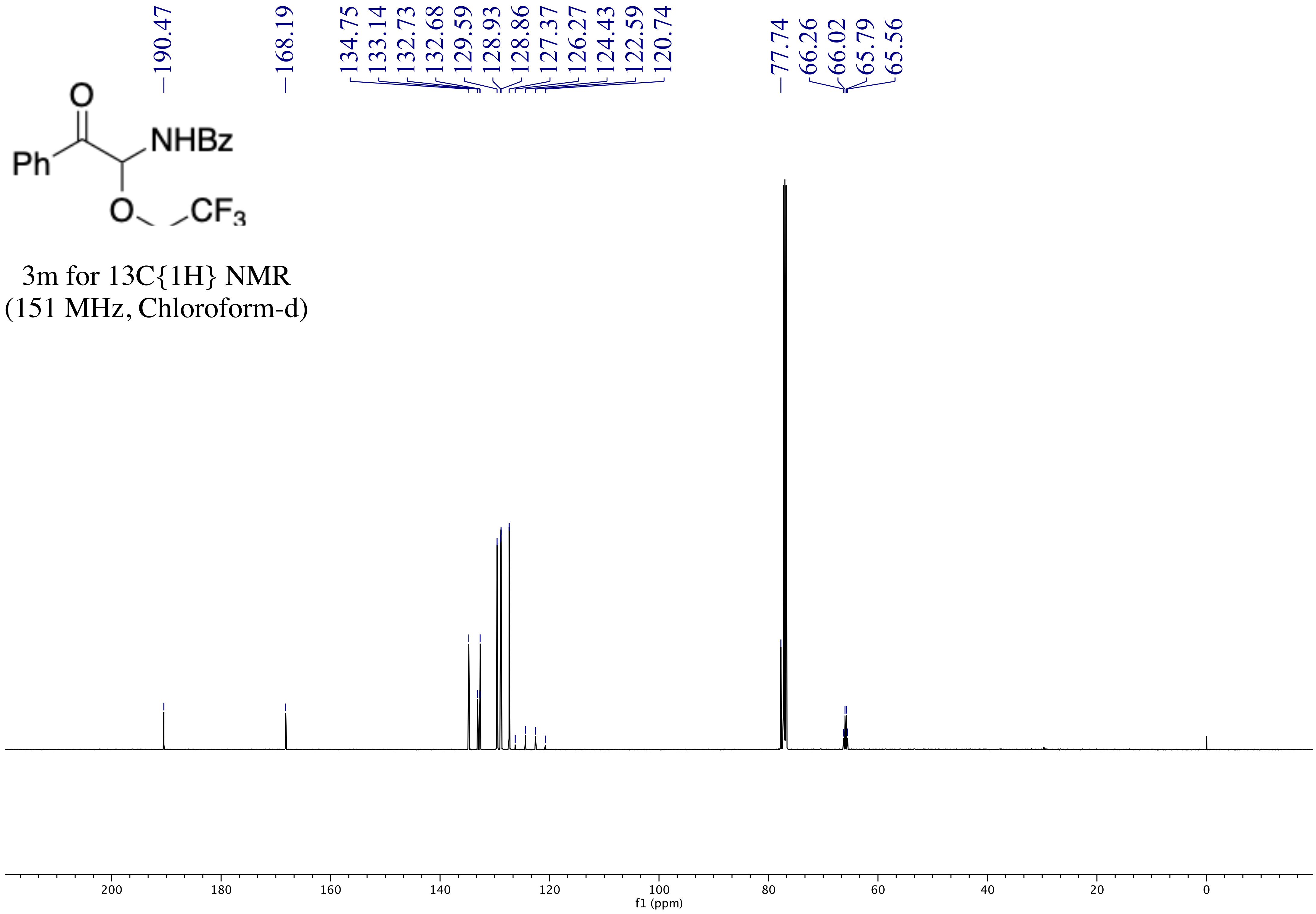

3m for 13C{1H} NMR
(151 MHz, Chloroform-d)

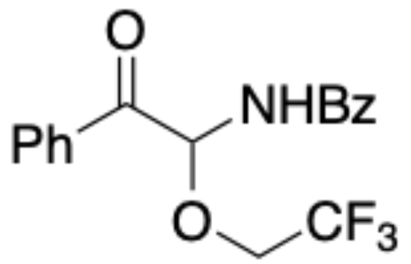

3m for 19F NMR
(376 MHz, Chloroform-d)

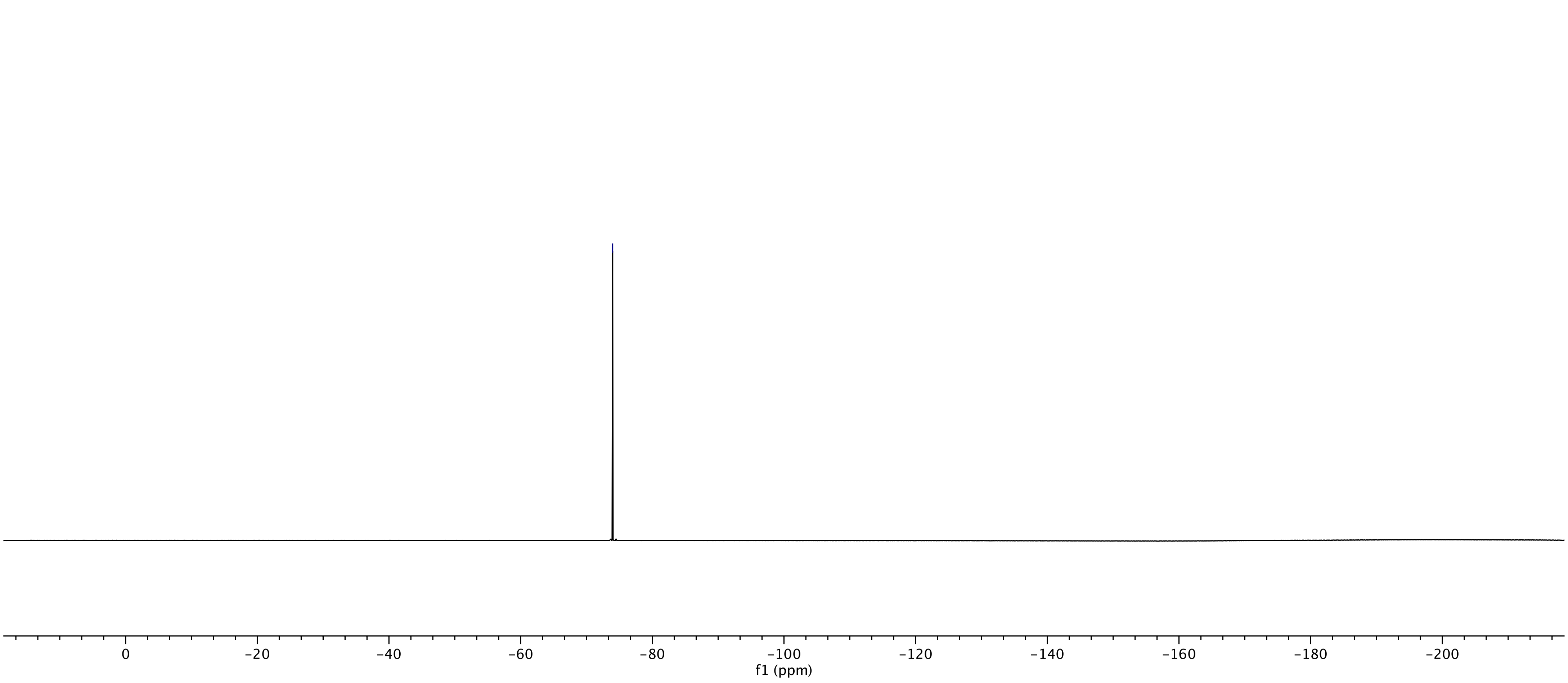

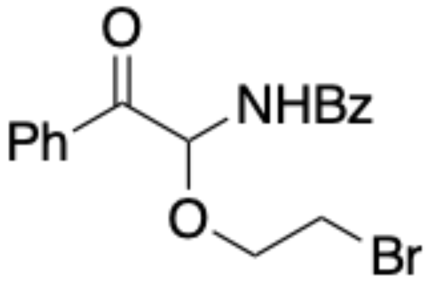

3n for 1H NMR
(600 MHz, Chloroform-d)

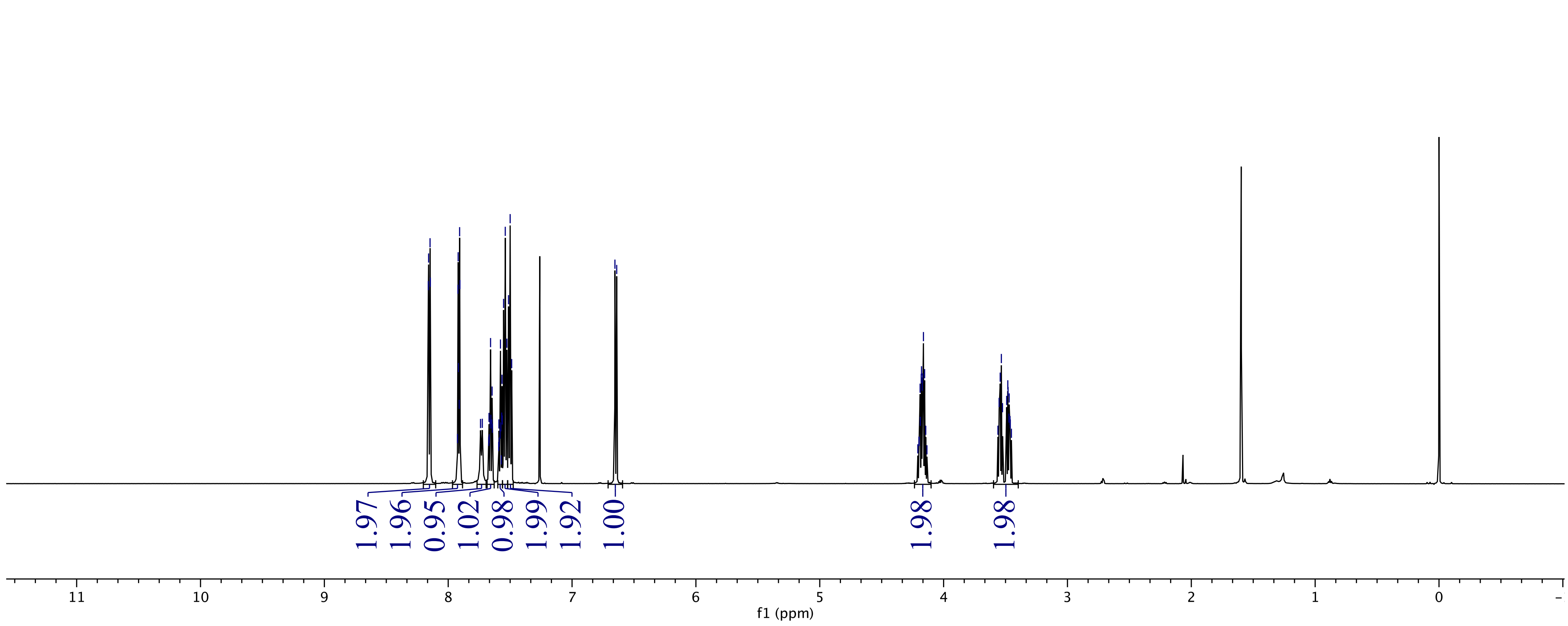

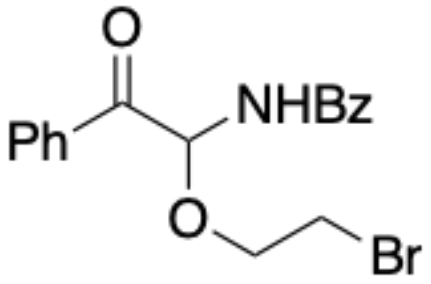

3n for 13C{1H} NMR
(151 MHz, Chloroform-d)

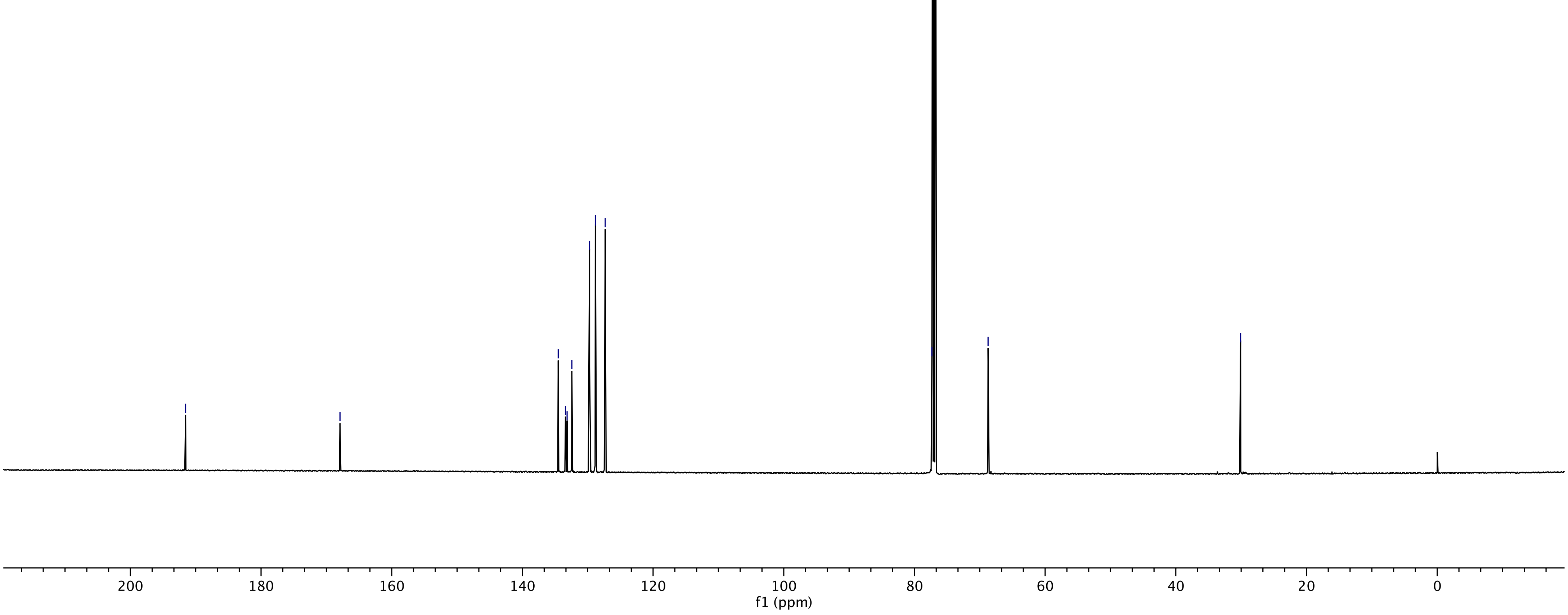

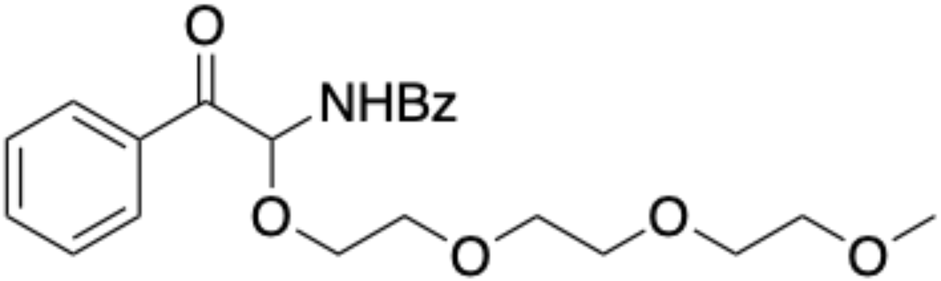

3o for 1H NMR
(400 MHz, Chloroform-d)

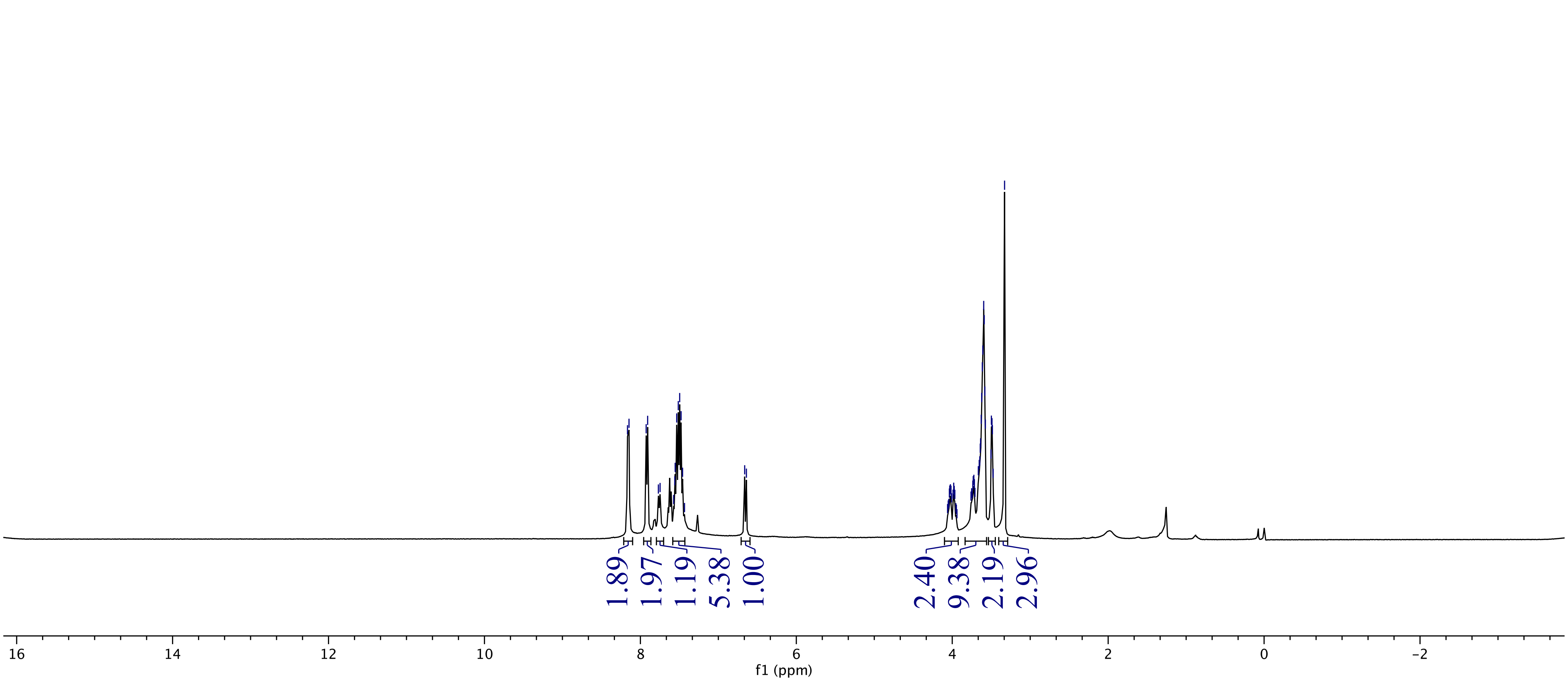

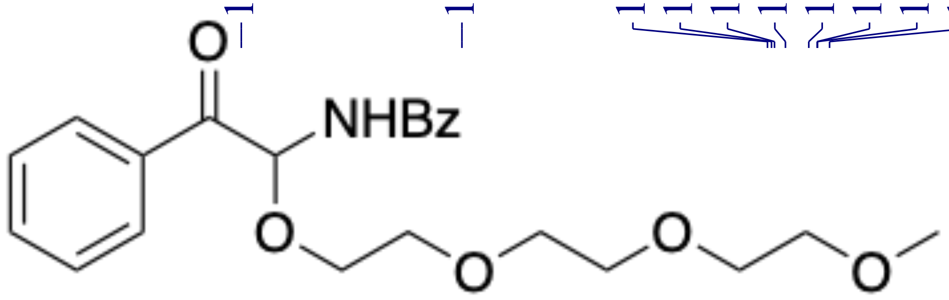

3o for 13C{1H} NMR
(101 MHz, Chloroform-d)

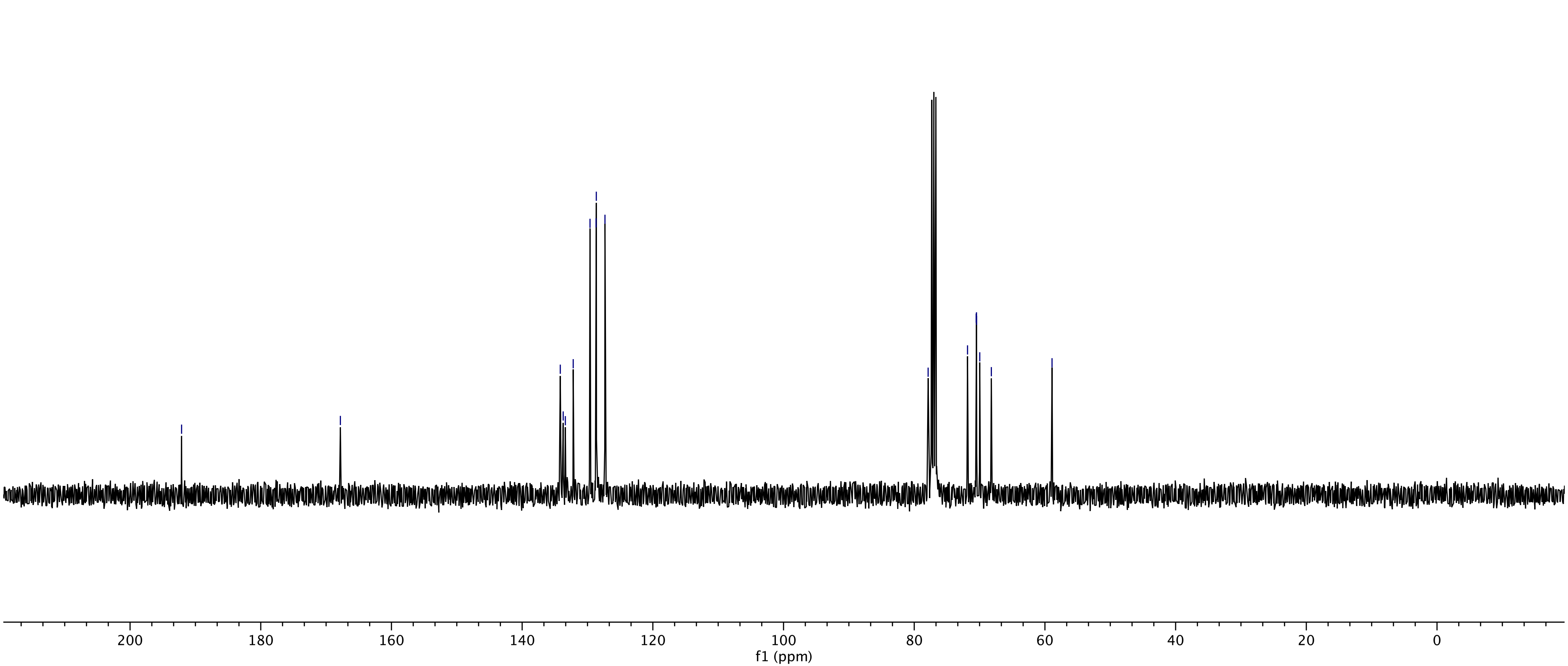

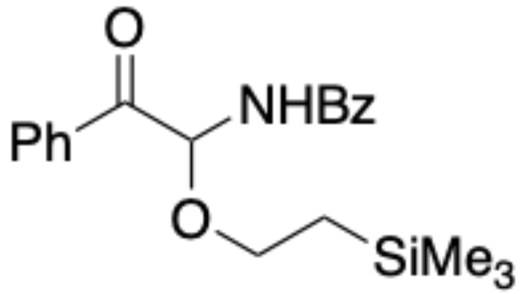

3p for 1H NMR
(600 MHz, Chloroform-d)

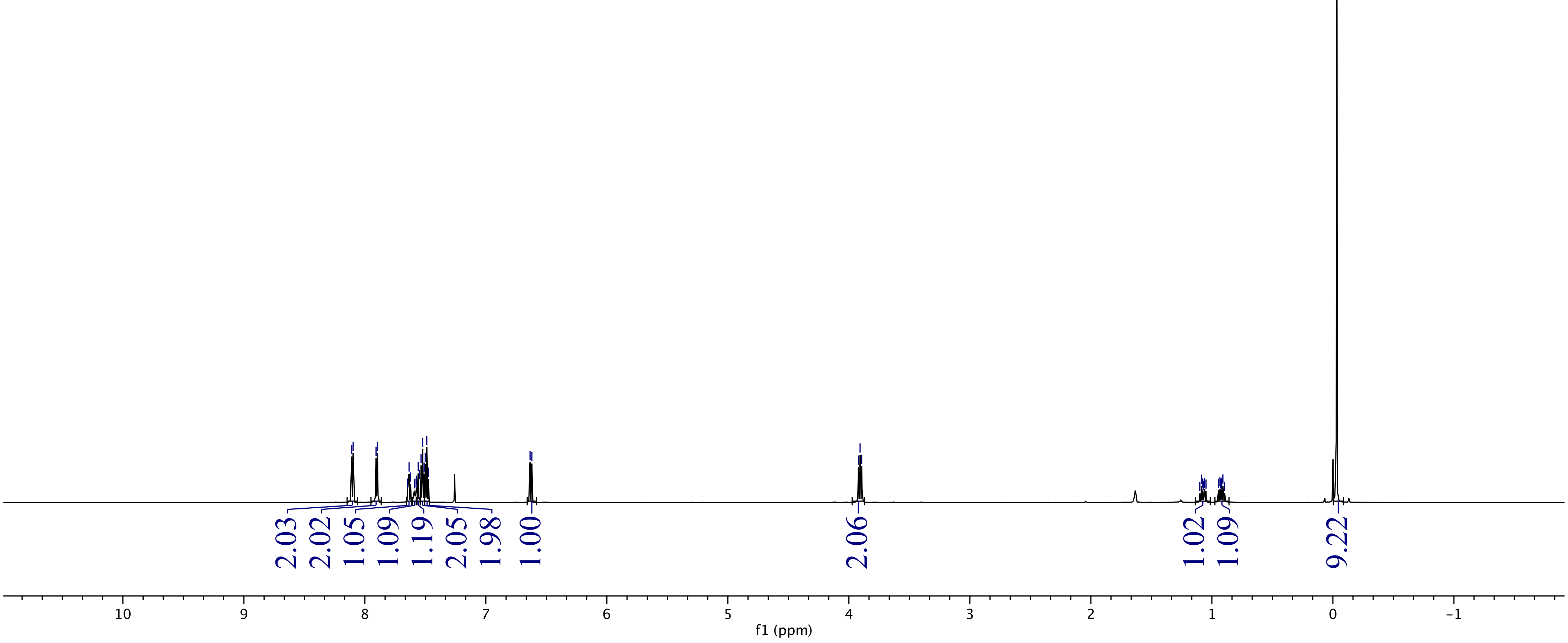

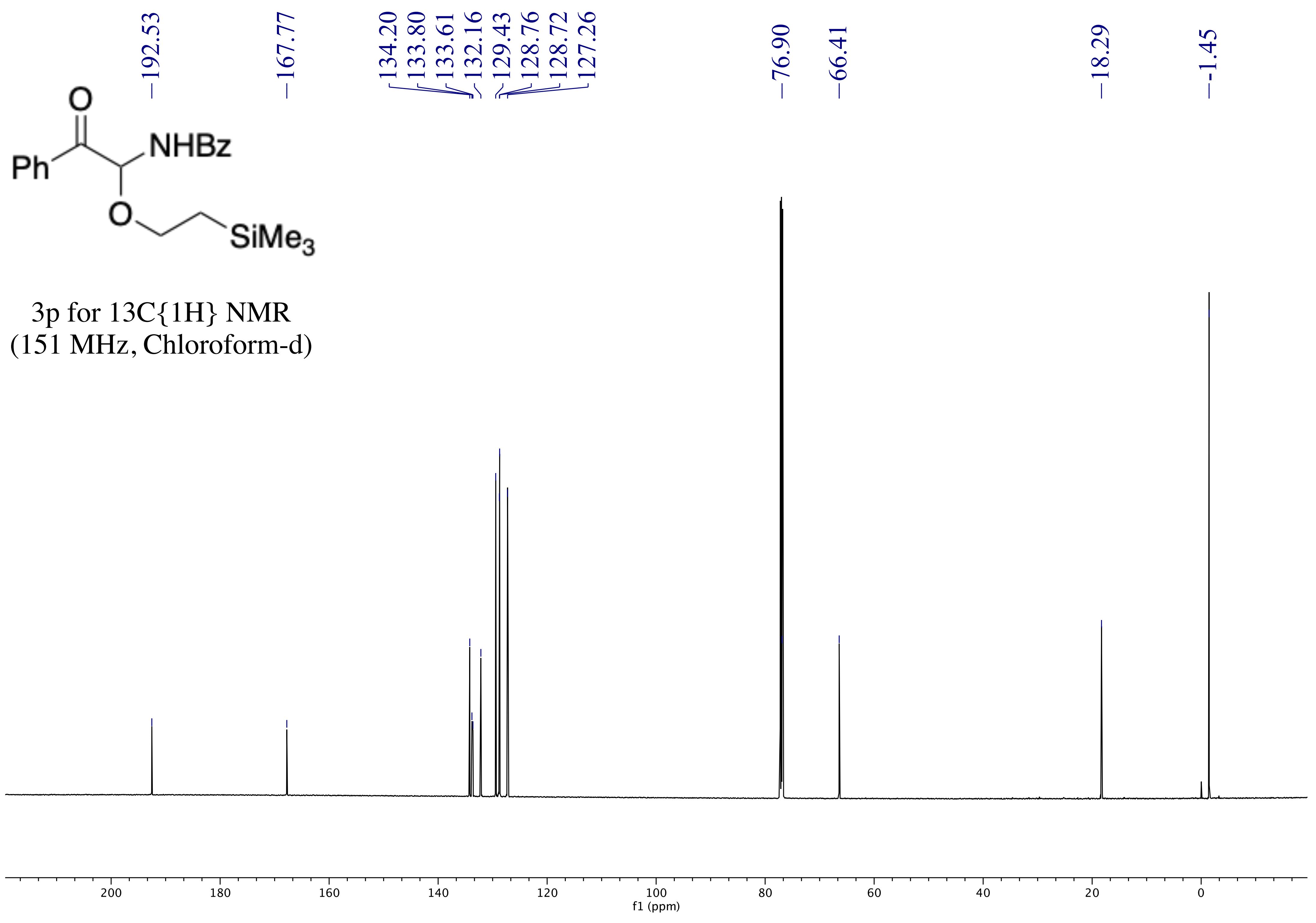

192.53
167.77
134.20
133.80
133.61
132.16
129.43
128.76
128.72
127.26
76.90
66.41
18.29
-1.45
Ph
O
NHBz
O
SiMe3
3p for 13C{1H} NMR
(151 MHz, Chloroform-d)
200
180
160
140
120
100
80
60
40
20
0
f1 (ppm)

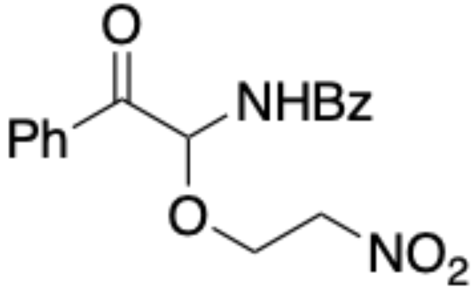

3q for 1H NMR
(600 MHz, Chloroform-d)

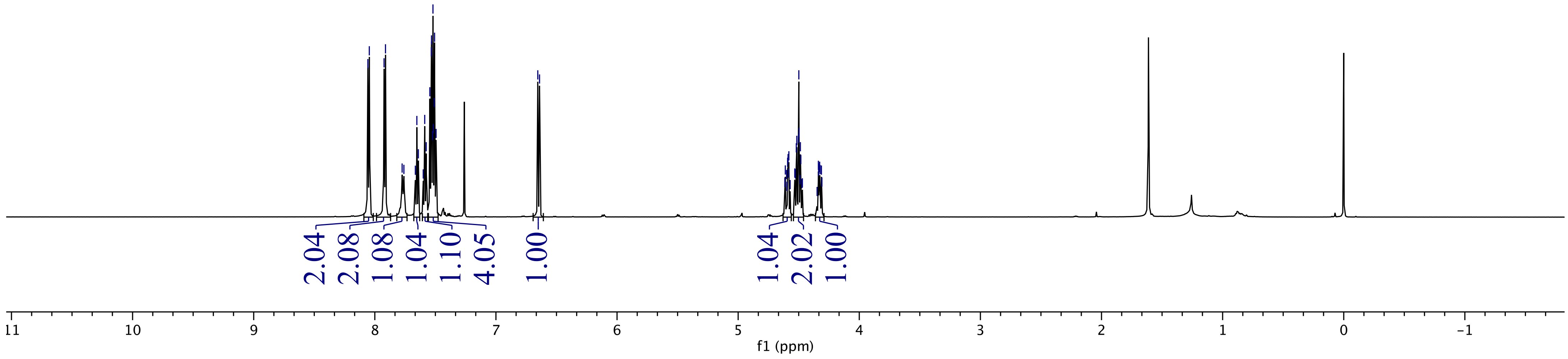

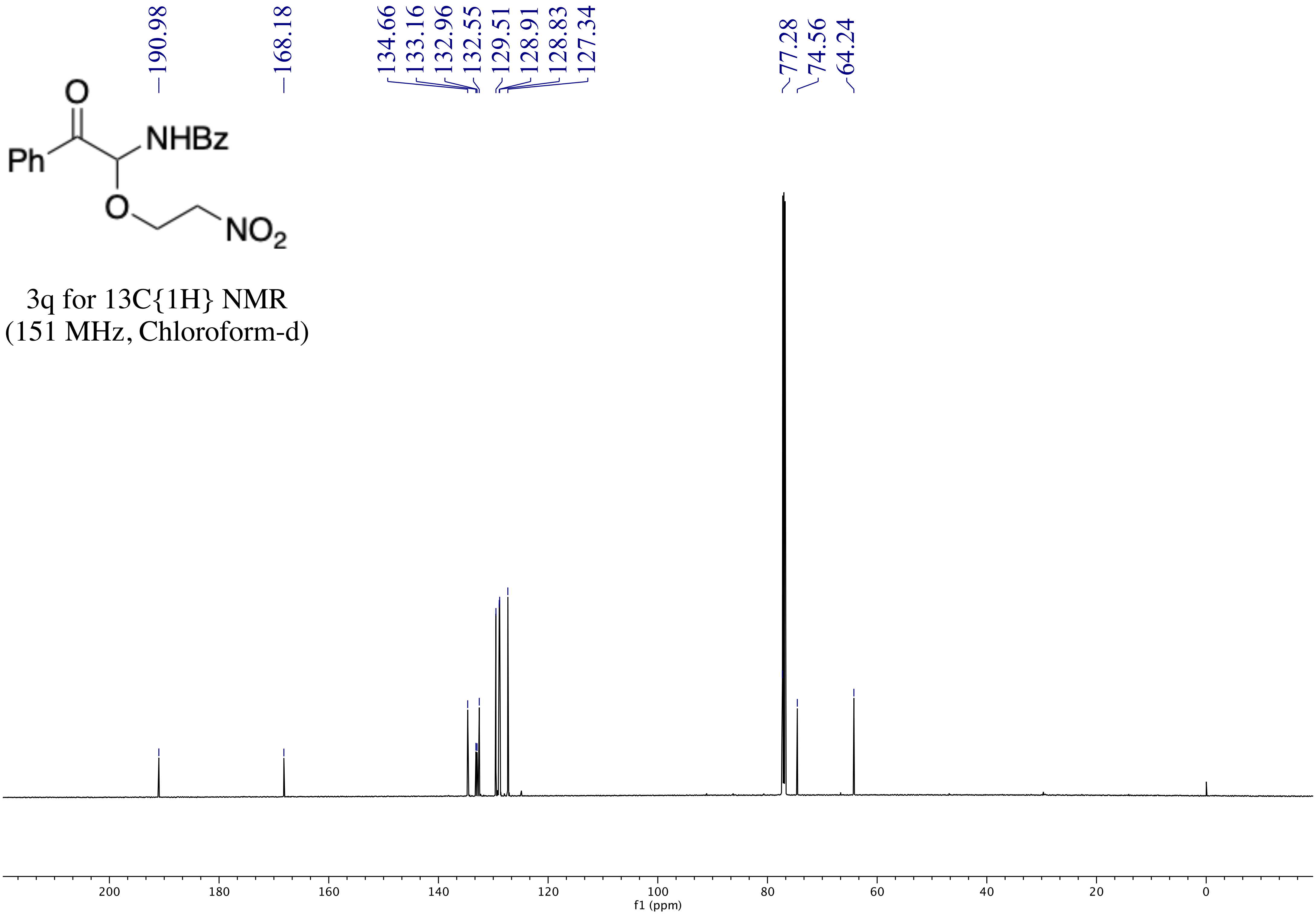

3q for 13C{1H} NMR
(151 MHz, Chloroform-d)

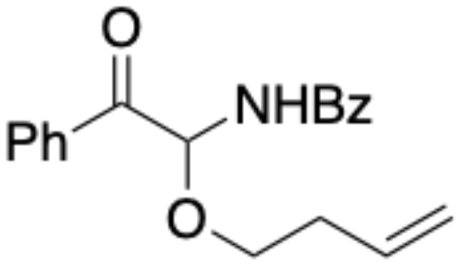

3r for 1H NMR
(600 MHz, Chloroform-d)

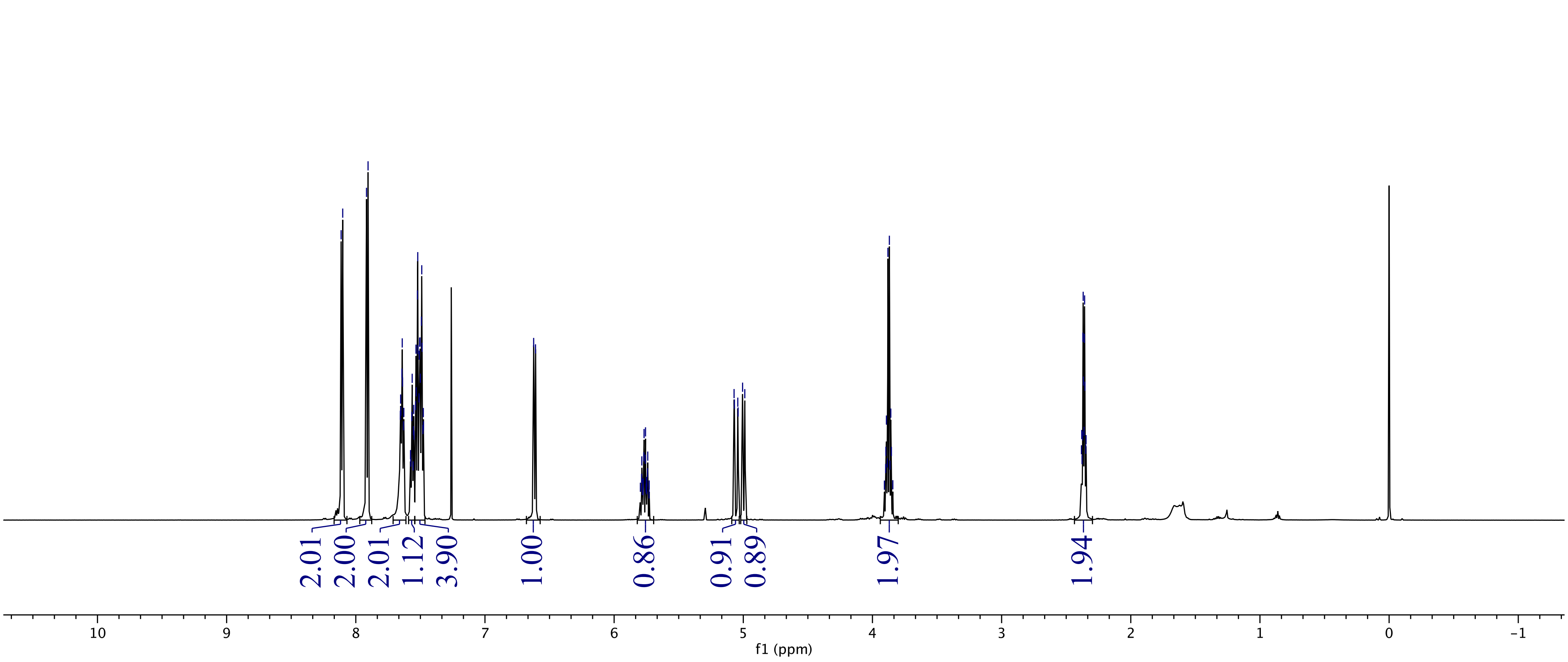

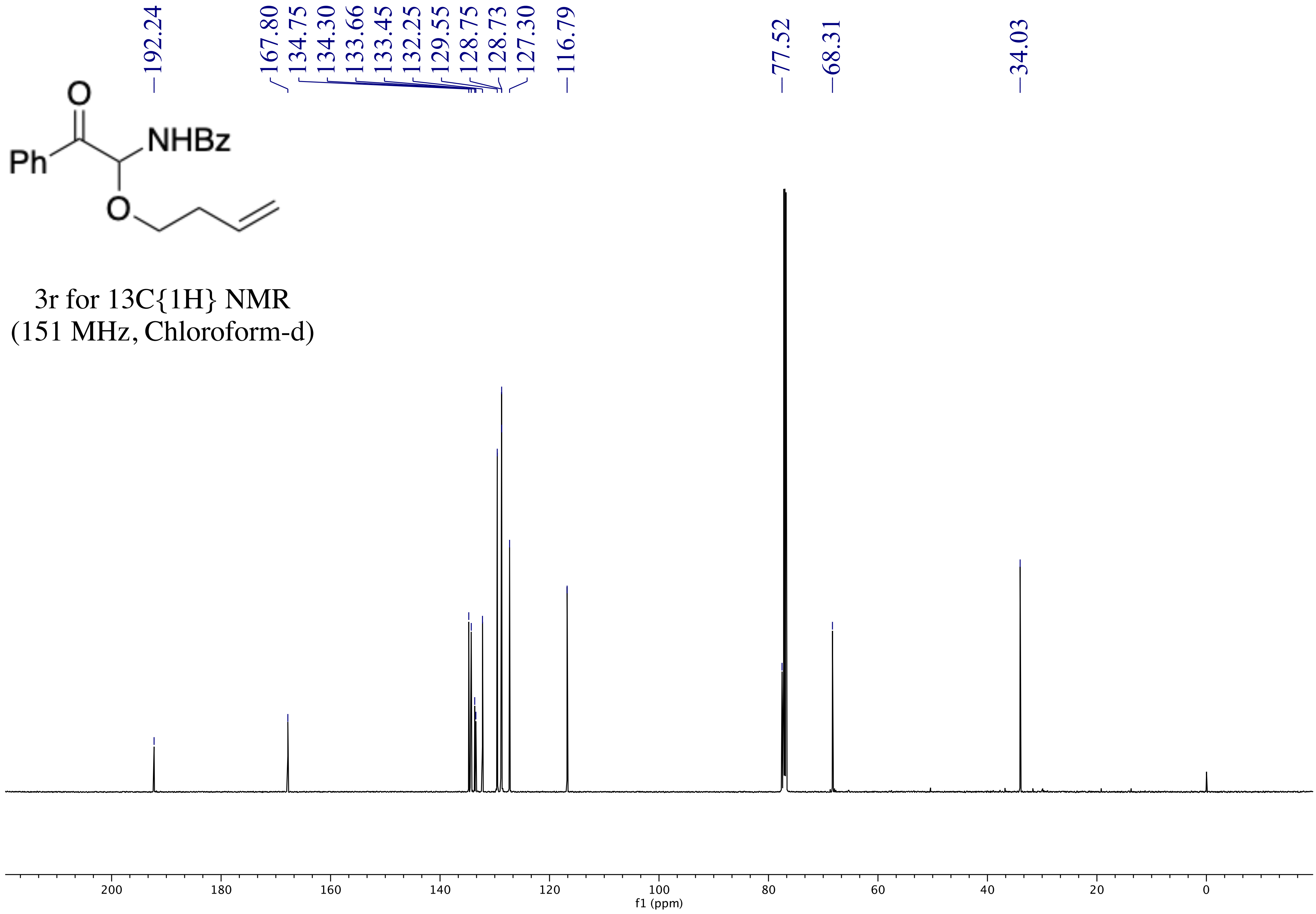
192.24
167.80
134.75
134.30
133.66
133.45
132.25
129.55
128.75
128.73
127.30
116.79
77.52
68.31
34.03
Ph
O
NHBz
O
3r for 13C{1H} NMR
(151 MHz, Chloroform-d)
200
180
160
140
120
100
80
60
40
20
0
f1 (ppm)

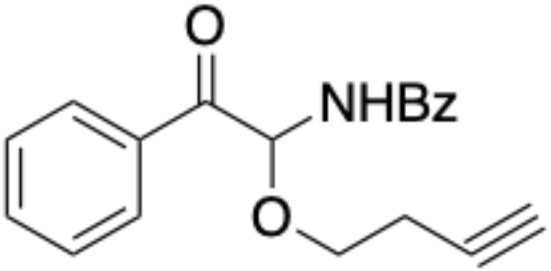

3s for 1H NMR
(400 MHz, Chloroform-d)

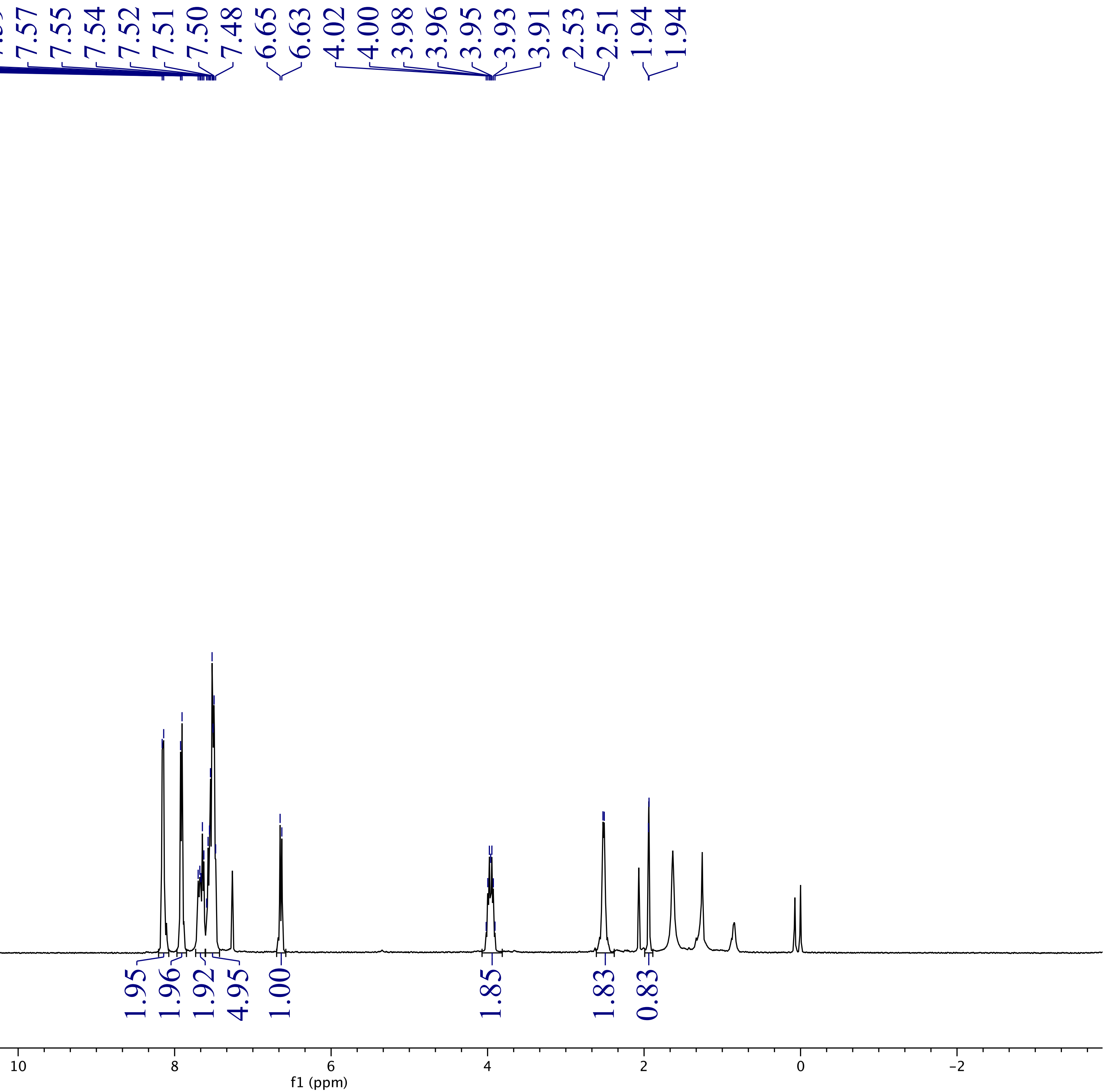

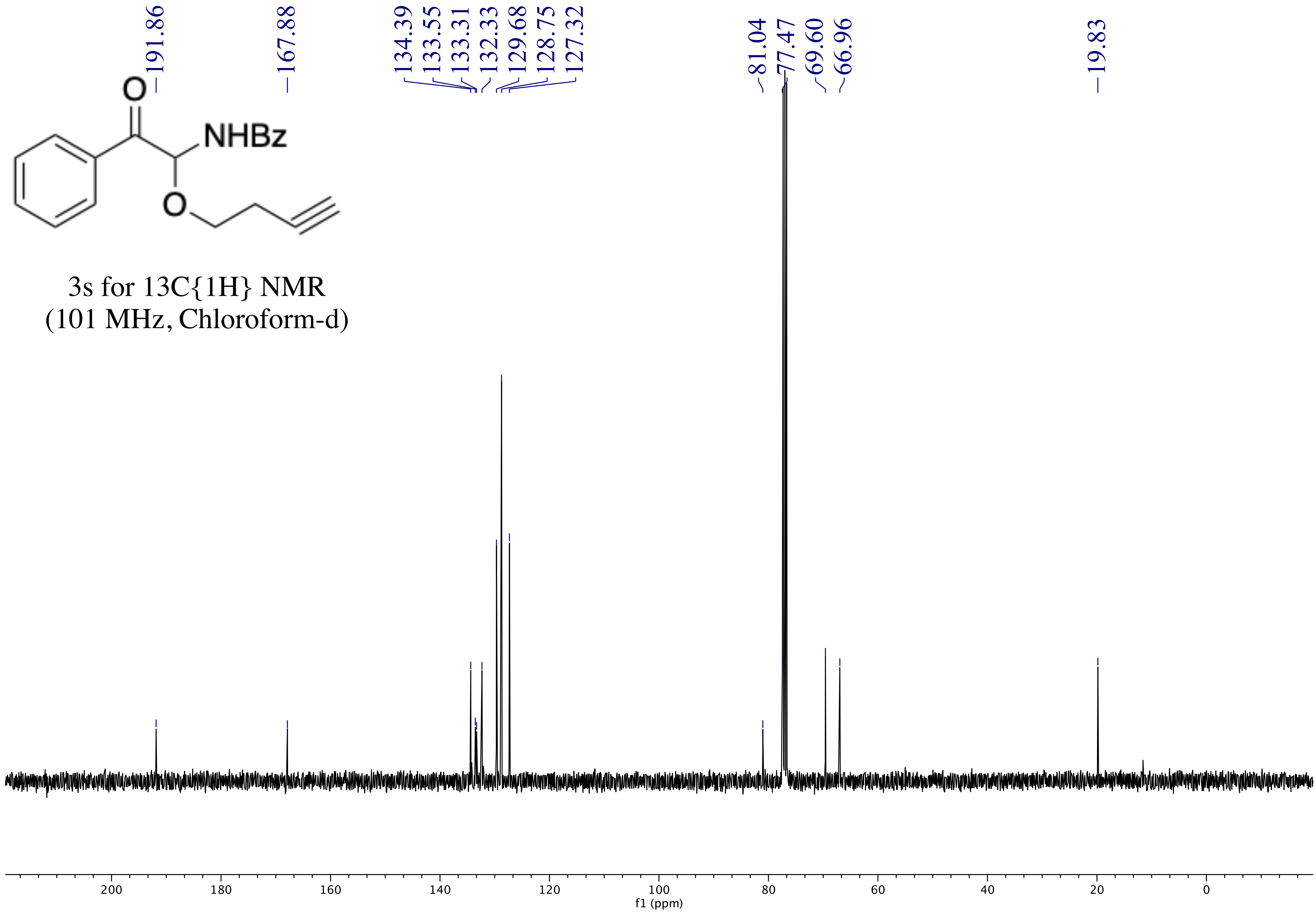

NHBz
3s for 13C{1H} NMR
(101 MHz, Chloroform-d)
191.86
167.88
134.39
133.55
133.31
132.33
129.68
128.75
127.32
81.04
77.47
69.60
66.96
19.83
200
180
160
140
120
100
80
60
40
20
0
f1 (ppm)

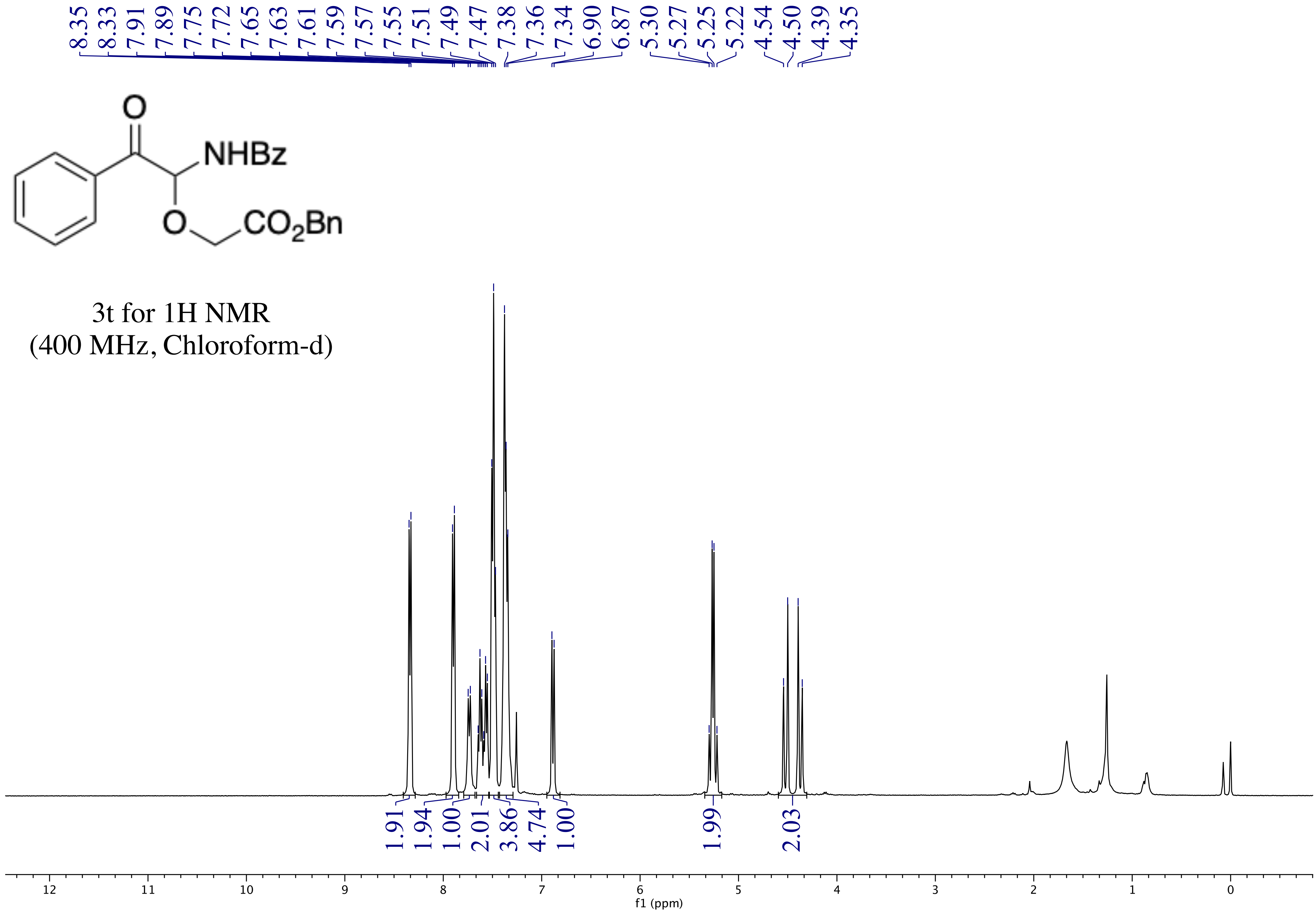
8.35 8.33 7.91 7.89 7.75 7.72 7.65 7.63 7.61 7.59 7.57 7.55 7.51 7.49 7.47 7.38 7.36 7.34 6.90 6.87 5.30 5.27 5.25 5.22 4.54 4.50 4.39 4.35
NHBz
CO2Bn
3t for 1H NMR
(400 MHz, Chloroform-d)
1.91 1.94 1.00 2.01 3.86 4.74 1.00 1.99 2.03
12 11 10 9 8 7 6 5 4 3 2 1 0
f1 (ppm)

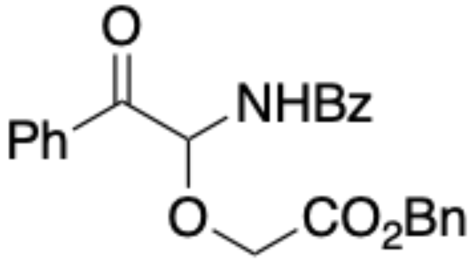

3t for 13C{1H} NMR
(151 MHz, Chloroform-d)

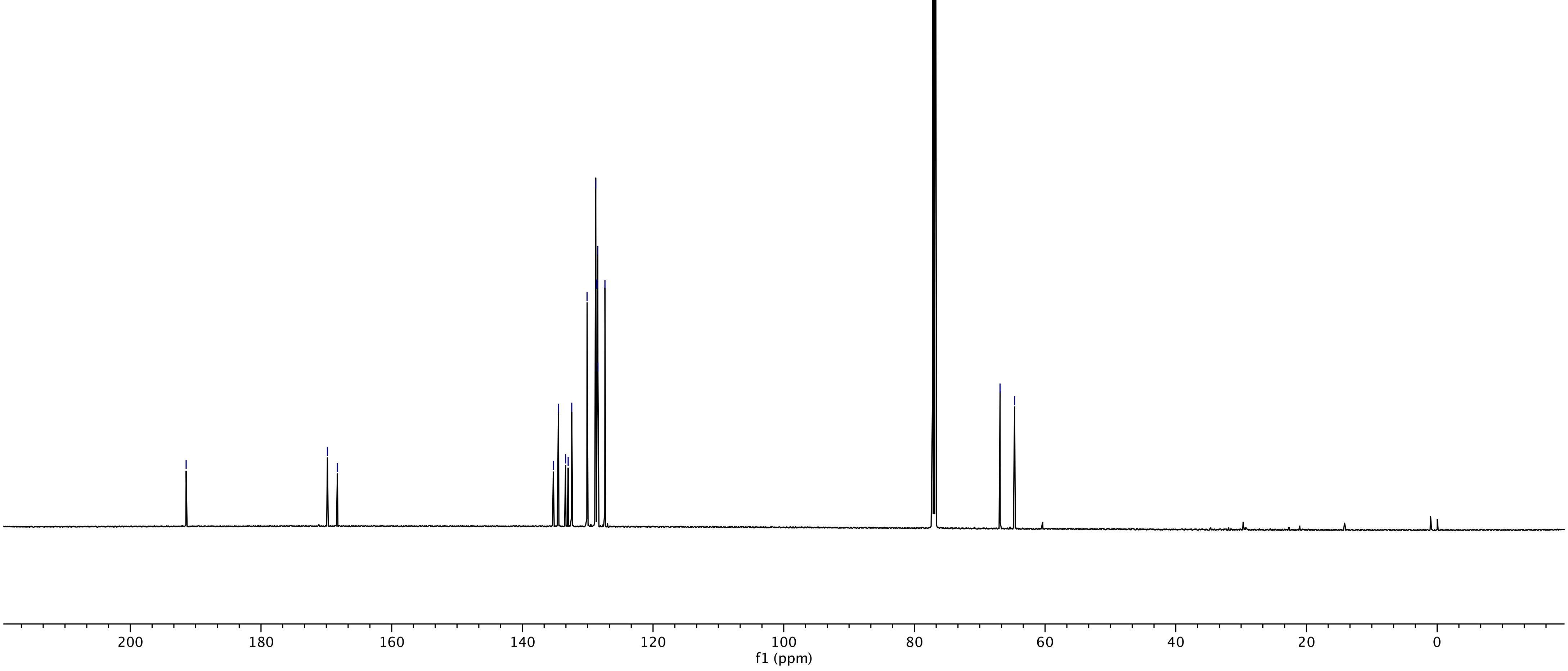

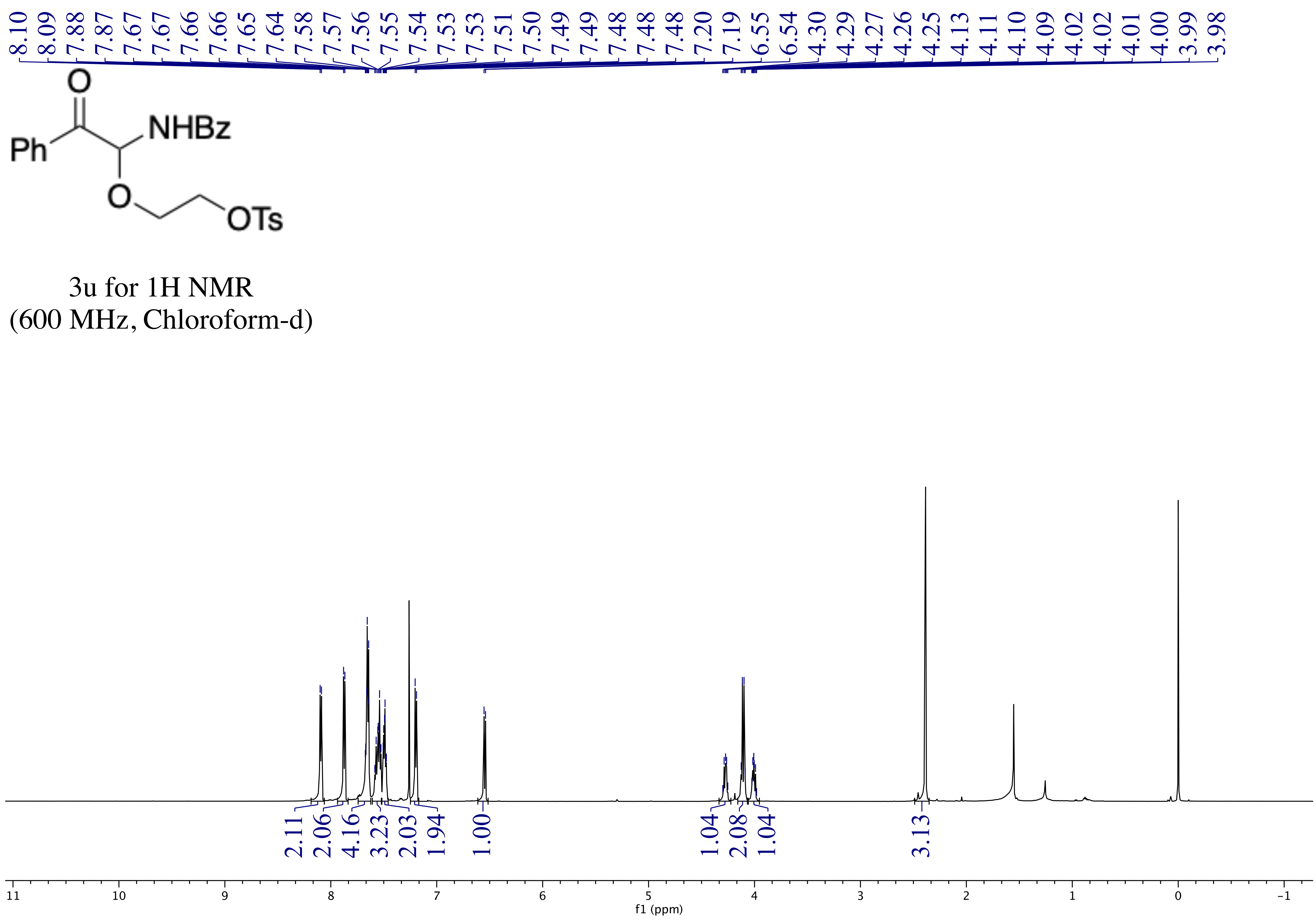
8.10 8.09 7.88 7.87 7.67 7.67 7.66 7.66 7.65 7.64 7.58 7.57 7.56 7.55 7.54 7.53 7.53 7.51 7.50 7.49 7.49 7.48 7.48 7.48 7.20 7.19 6.55 6.54 4.30 4.29 4.27 4.26 4.25 4.13 4.11 4.10 4.09 4.02 4.02 4.01 4.00 3.99 3.98
Ph
O
NHBz
O
OTs
3u for 1H NMR
(600 MHz, Chloroform-d)
2.11 2.06 4.16 3.23 2.03 1.94 1.00 1.04 2.08 1.04 3.13
11 10 9 8 7 6 5 4 3 2 1 0 -1
f1 (ppm)

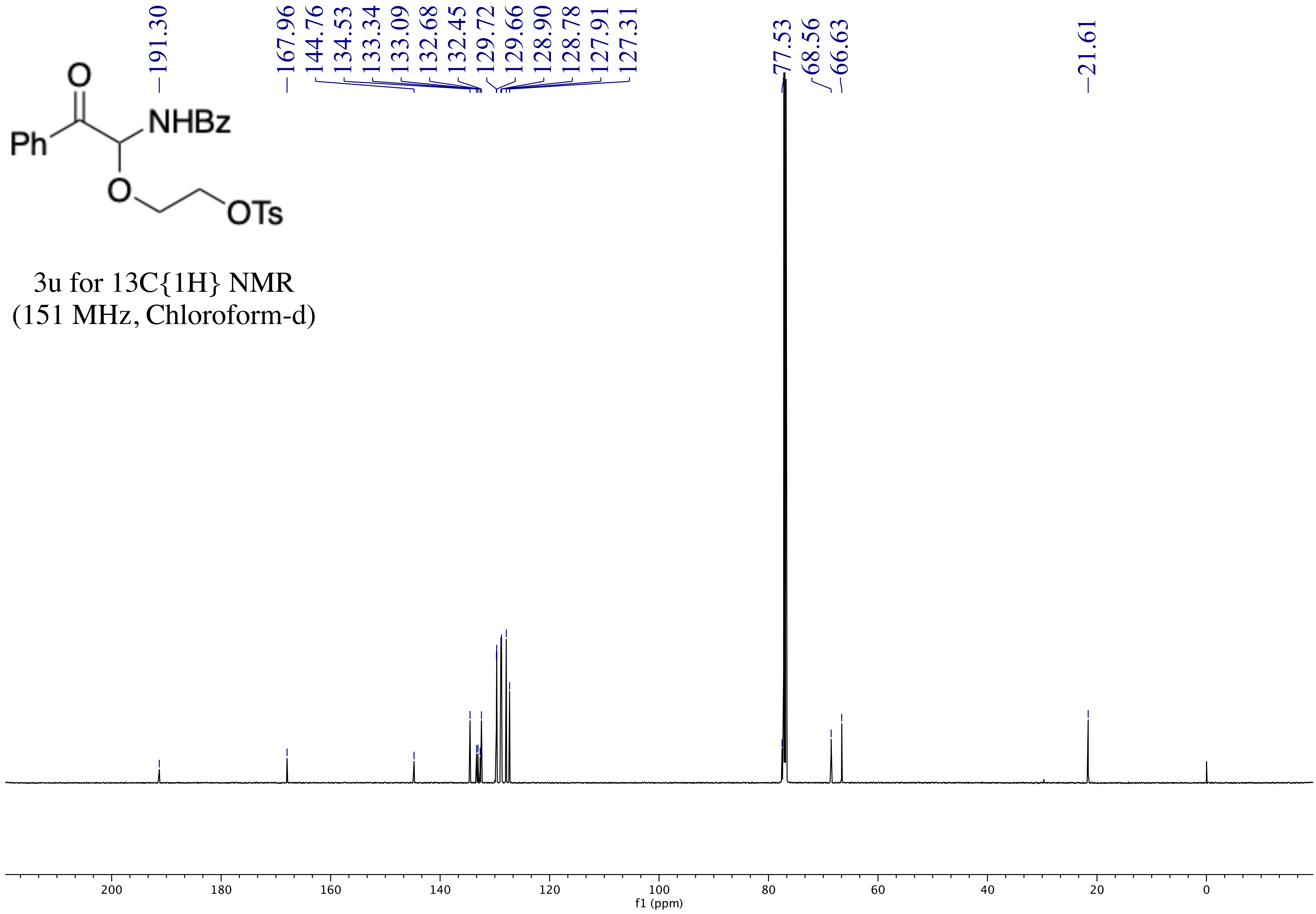

Ph
O
NHBz
O
OTs
3u for 13C{1H} NMR
(151 MHz, Chloroform-d)
191.30
167.96
144.76
134.53
133.34
133.09
132.68
132.45
129.72
129.66
128.90
128.78
127.91
127.31
77.53
68.56
66.63
21.61
200
180
160
140
120
100
80
60
40
20
0
f1 (ppm)

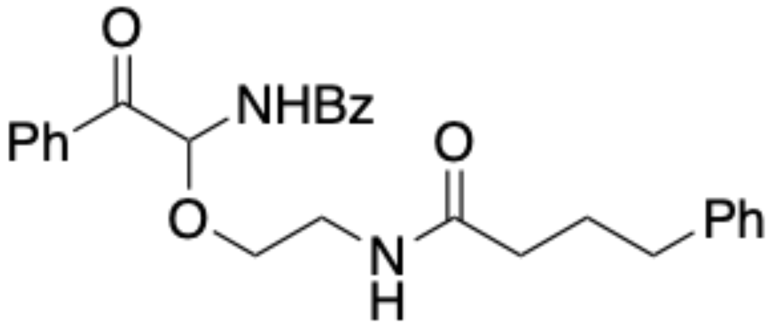

3v for 1H NMR
(600 MHz, Chloroform-d)

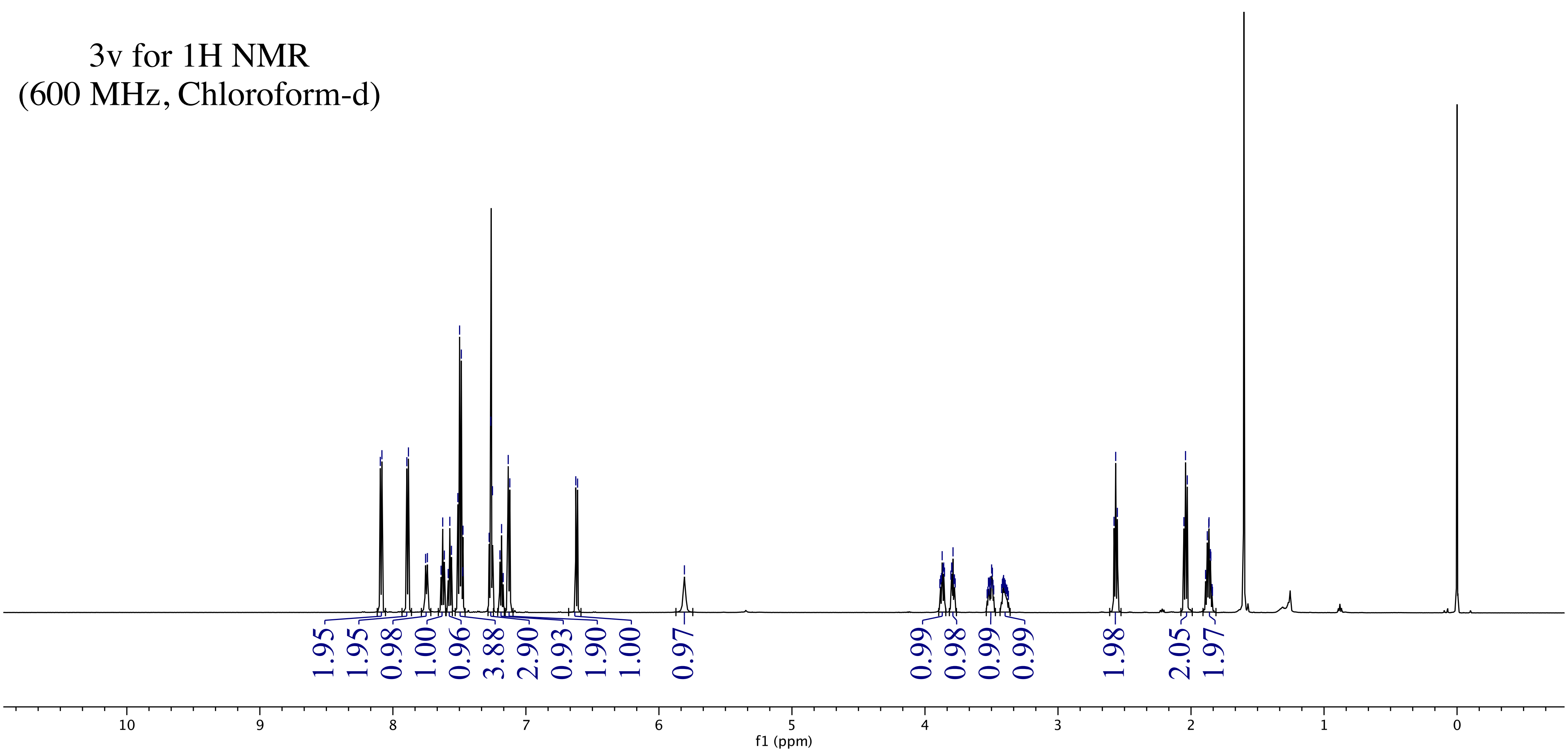

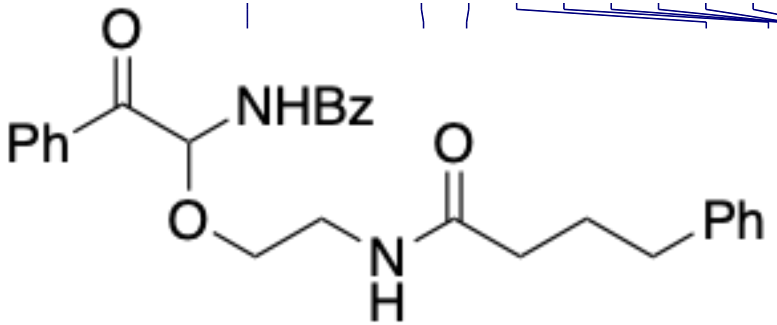

3v for 13C{1H} NMR
(151 MHz, Chloroform-d)

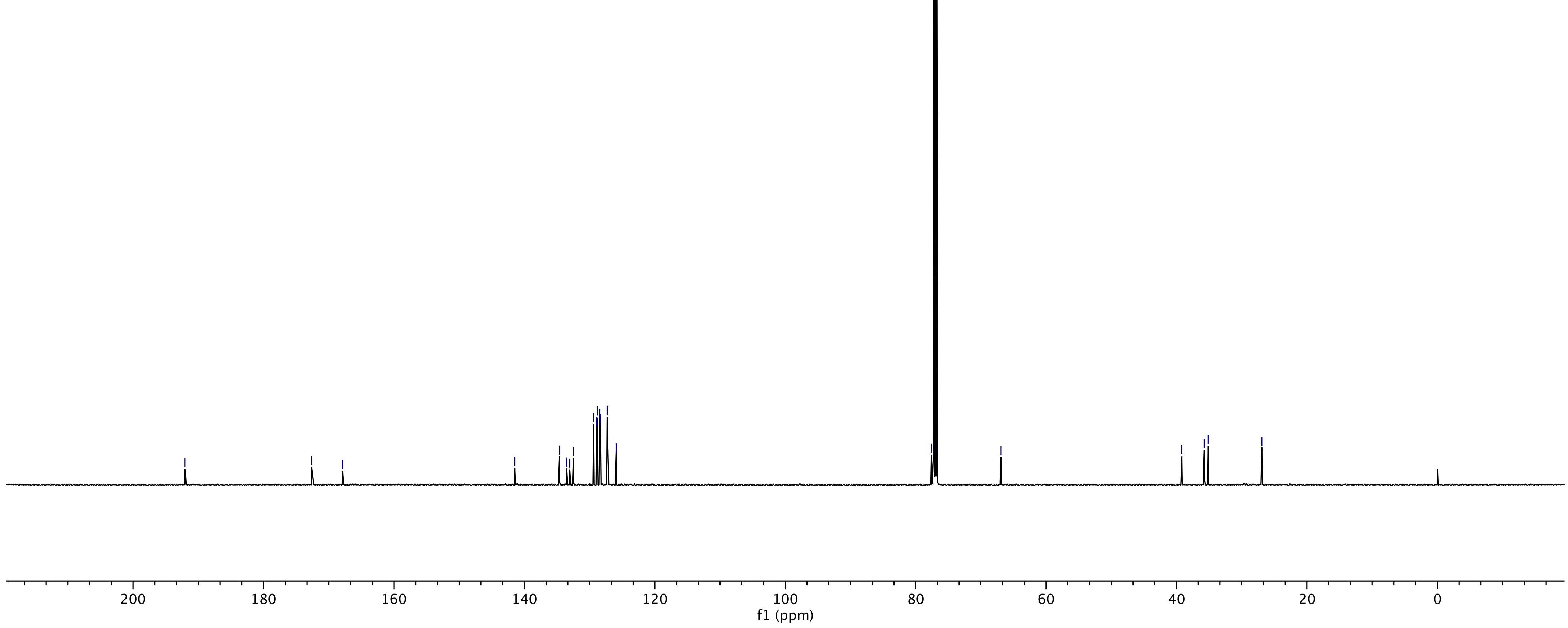

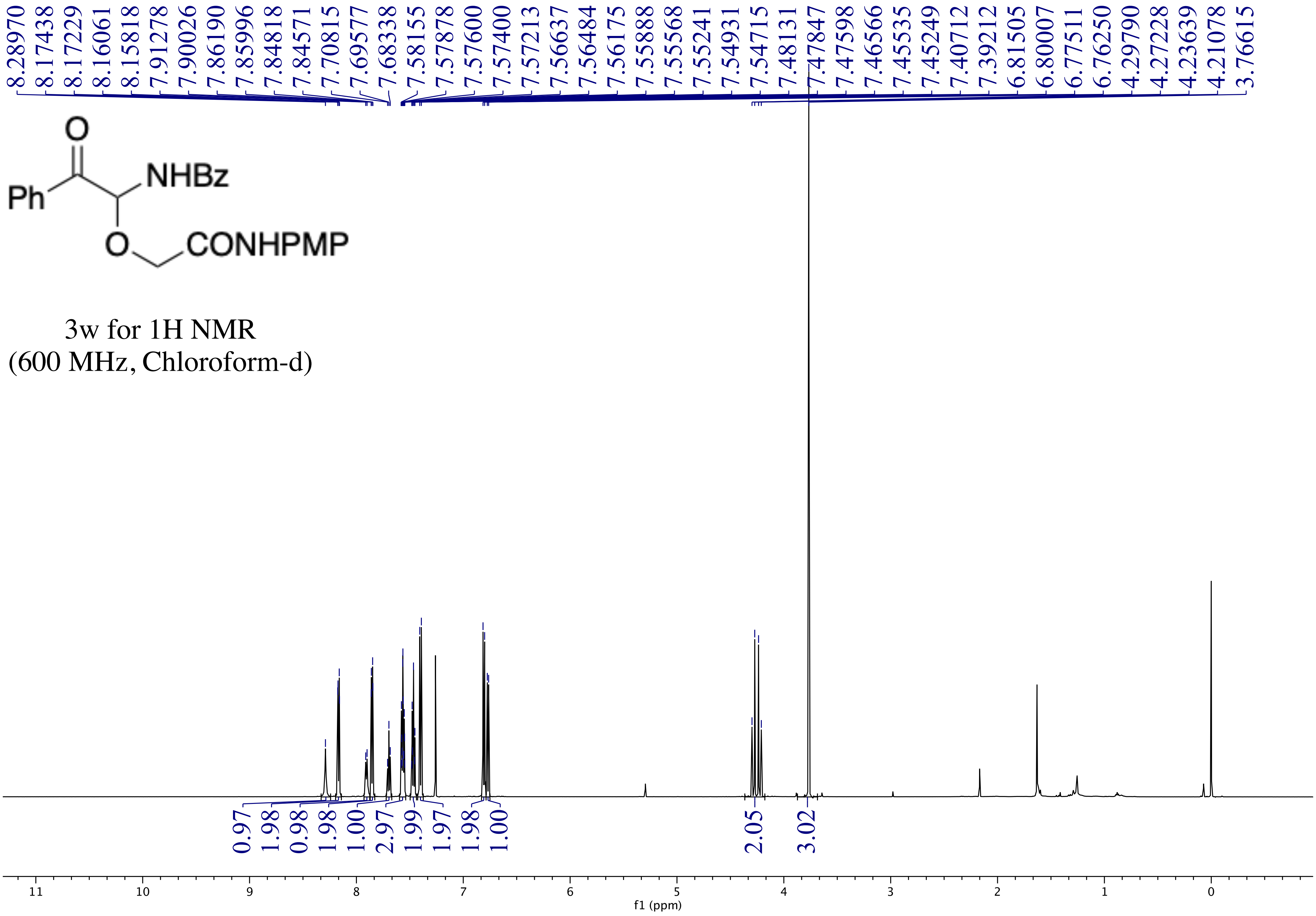

3w for 1H NMR
(600 MHz, Chloroform-d)

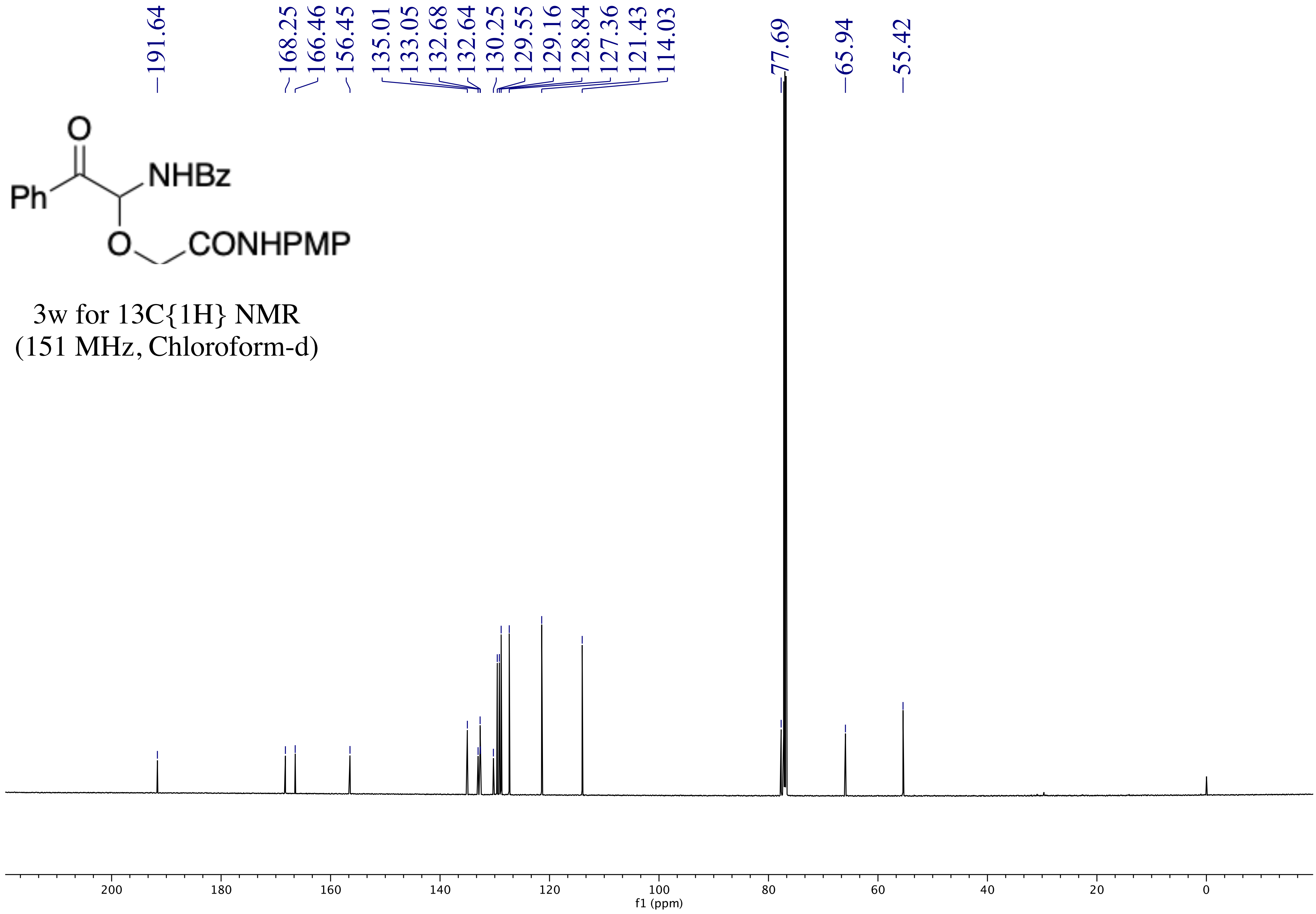
3w for 13C{1H} NMR
(151 MHz, Chloroform-d)
NHBz
Ph
O
CONHPMP
191.64
168.25
166.46
156.45
135.01
133.05
132.68
132.64
130.25
129.55
129.16
128.84
127.36
121.43
114.03
77.69
65.94
55.42
200
180
160
140
120
100
80
60
40
20
0
f1 (ppm)

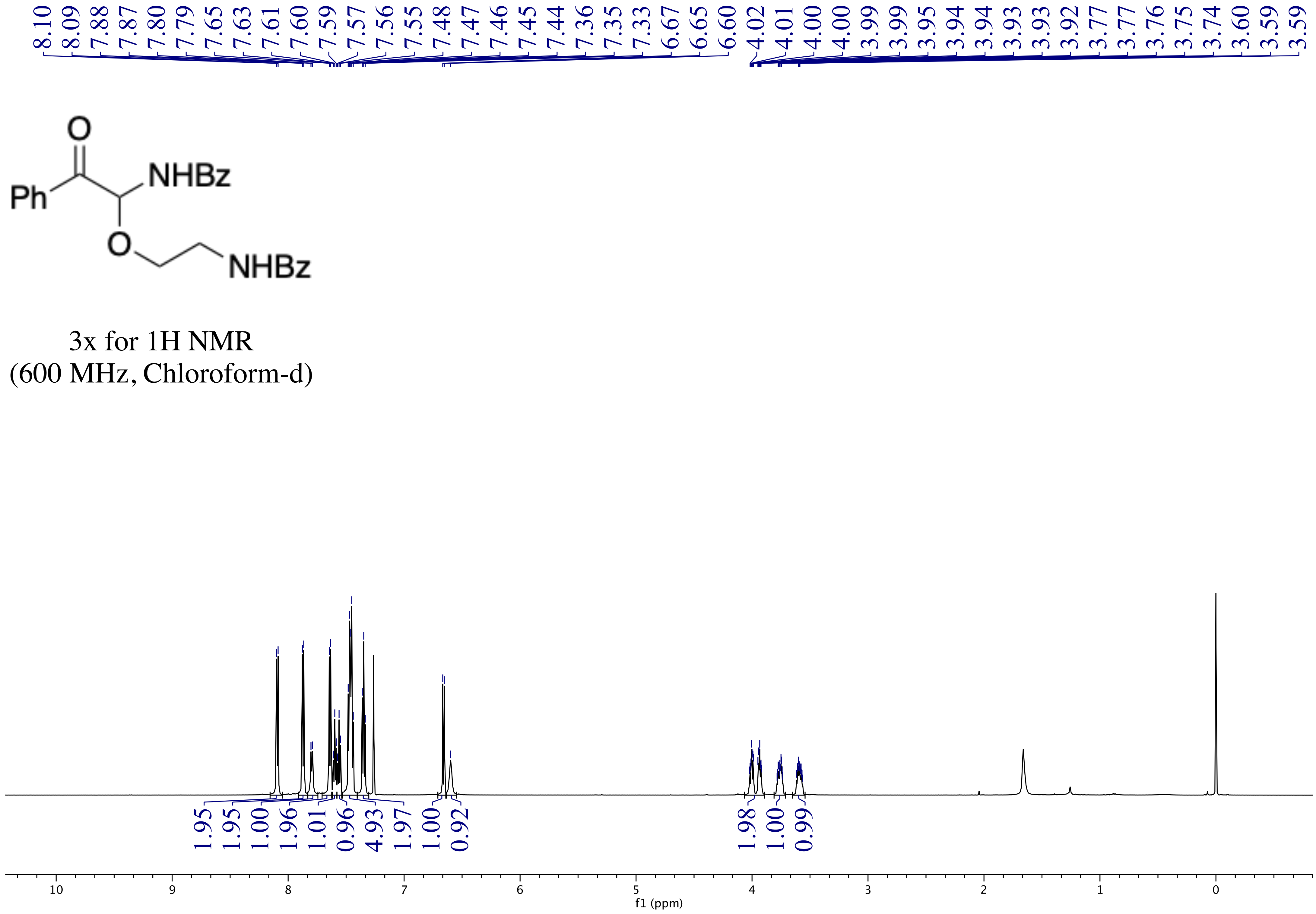
8.10 8.09 7.88 7.87 7.80 7.79 7.65 7.63 7.61 7.60 7.59 7.57 7.56 7.55 7.48 7.47 7.46 7.45 7.44 7.36 7.35 7.33 6.67 6.65 6.60 4.02 4.01 4.00 4.00 3.99 3.99 3.95 3.94 3.94 3.93 3.93 3.92 3.77 3.77 3.76 3.75 3.74 3.60 3.59 3.59
Ph
O
NHBz
O
NHBz
3x for 1H NMR
(600 MHz, Chloroform-d)
1.95 1.95 1.00 1.96 1.01 0.96 4.93 1.97 1.00 0.92
1.98 1.00 0.99
10 9 8 7 6 5 4 3 2 1 0
f1 (ppm)

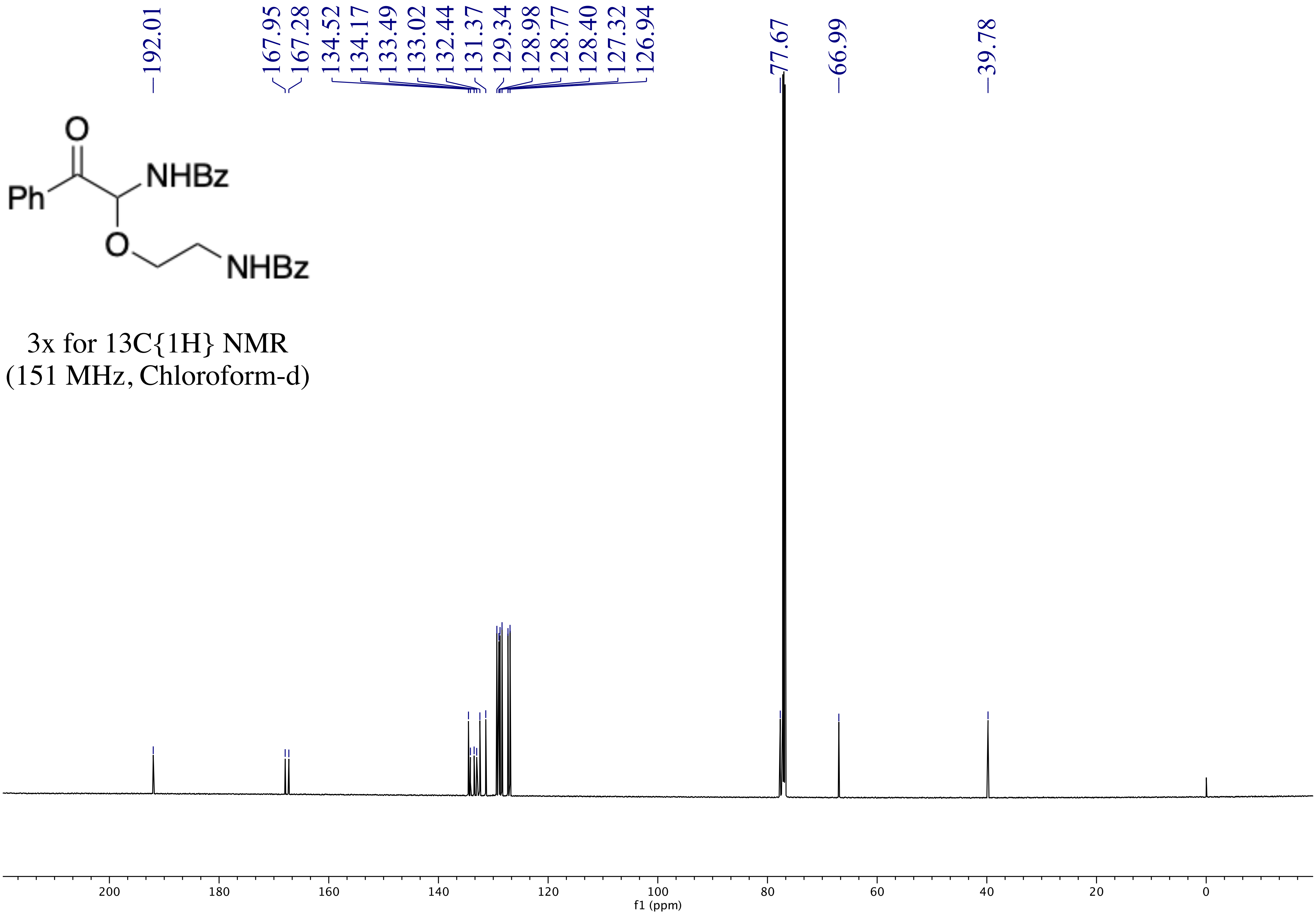

3x for 13C{1H} NMR
(151 MHz, Chloroform-d)

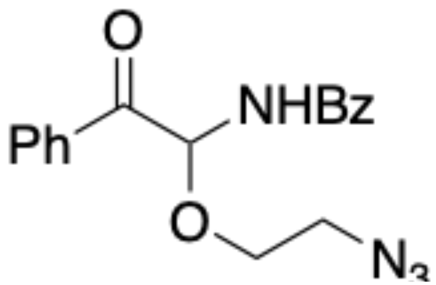

3y for 1H NMR
(600 MHz, Chloroform-d)

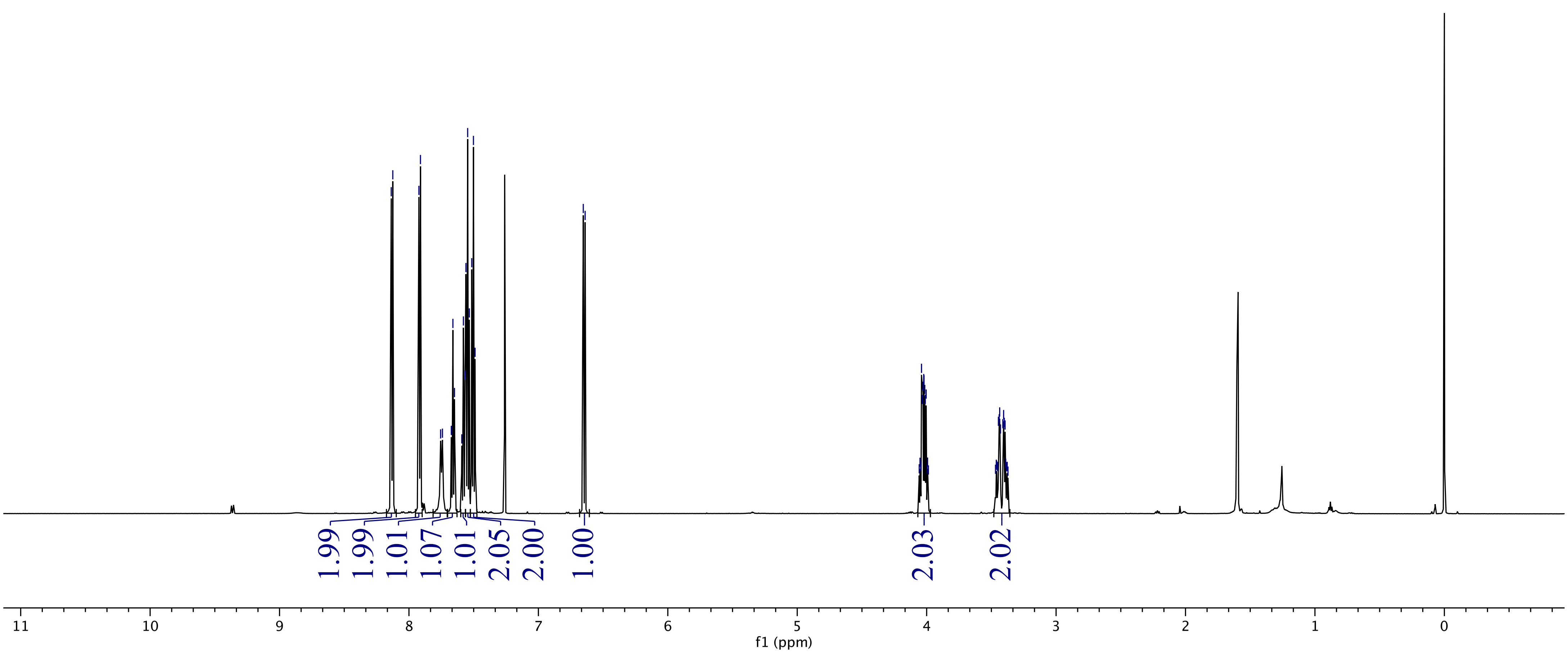

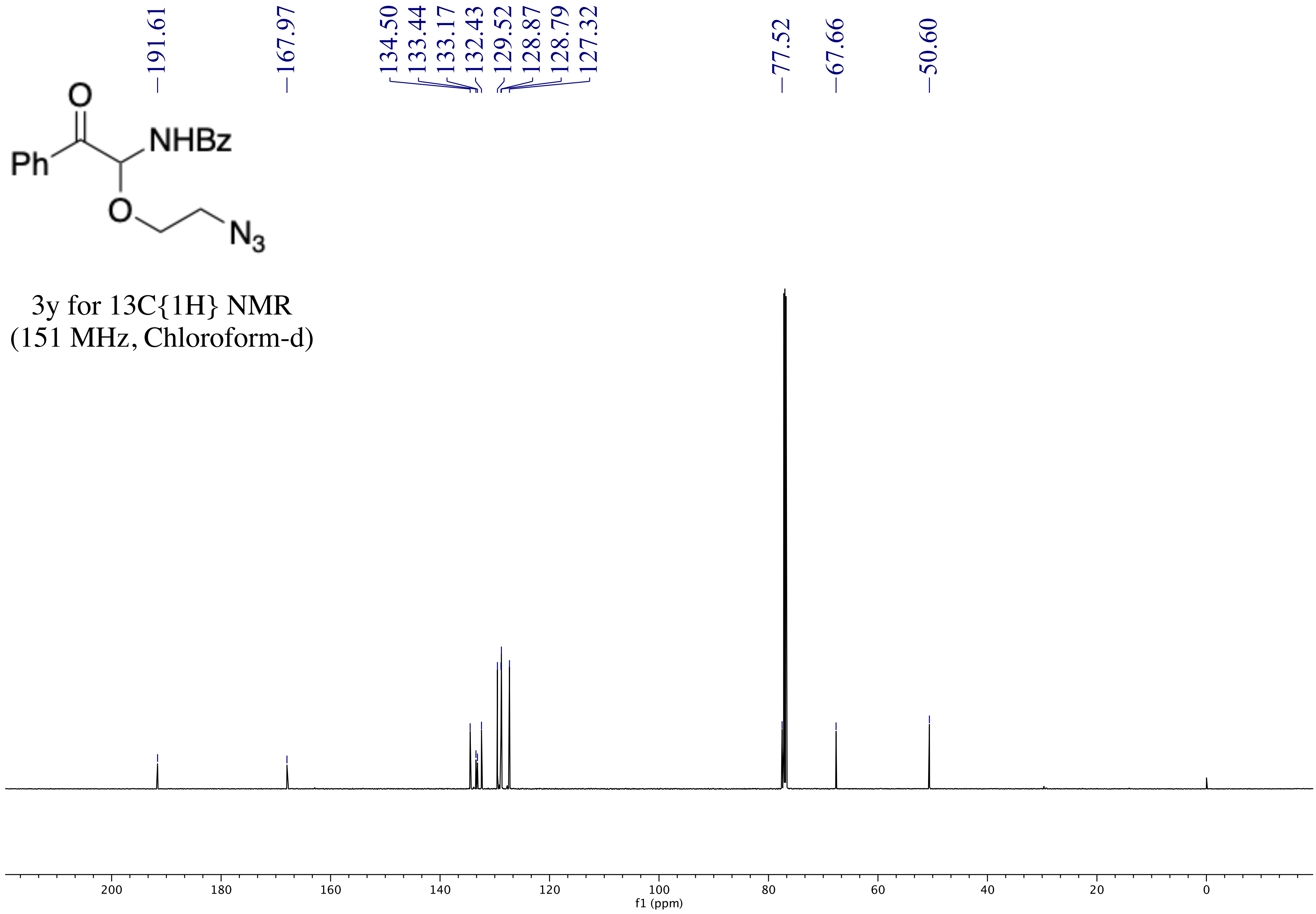

3y for 13C{1H} NMR
(151 MHz, Chloroform-d)

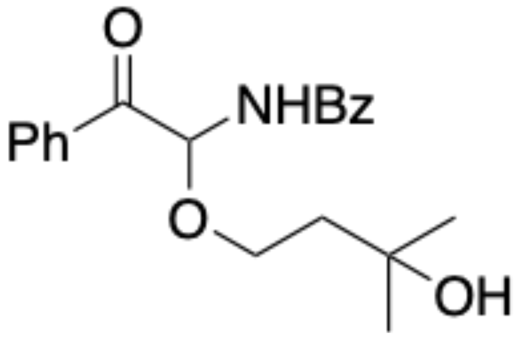

3z for 1H NMR
(600 MHz, Chloroform-d)

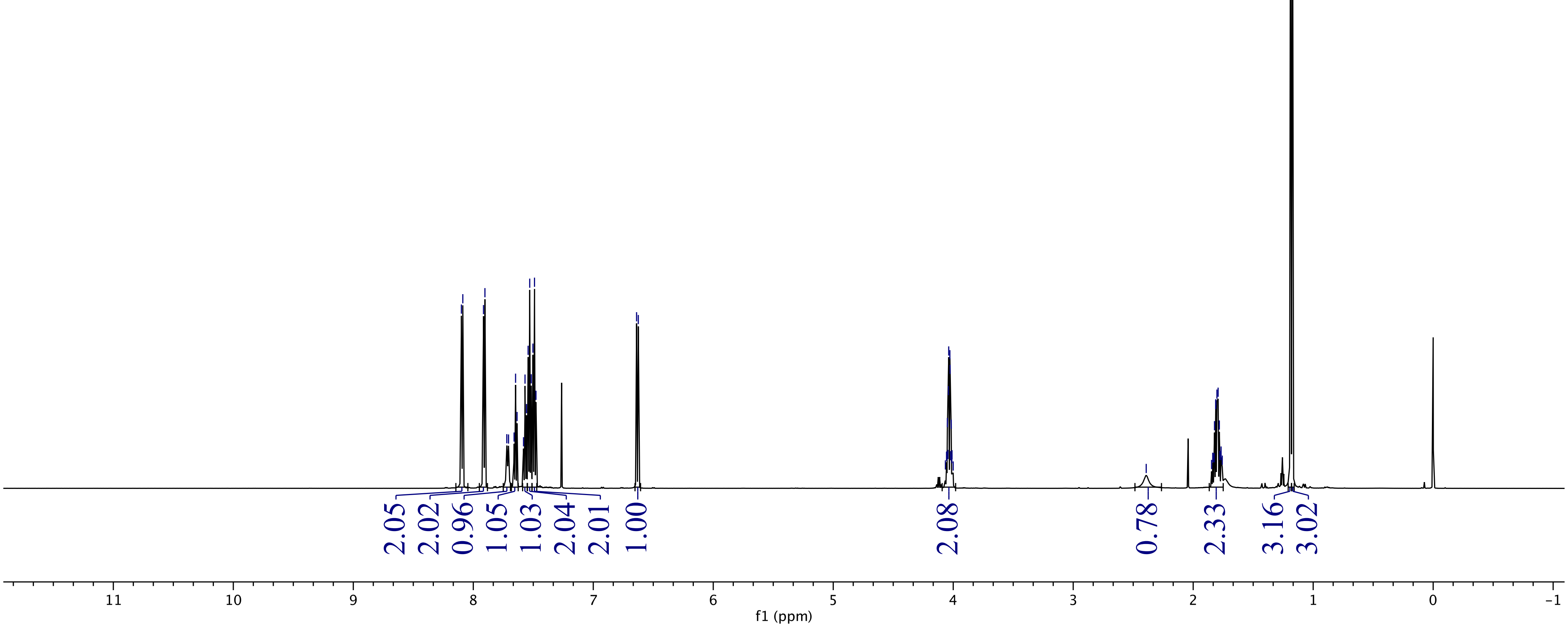

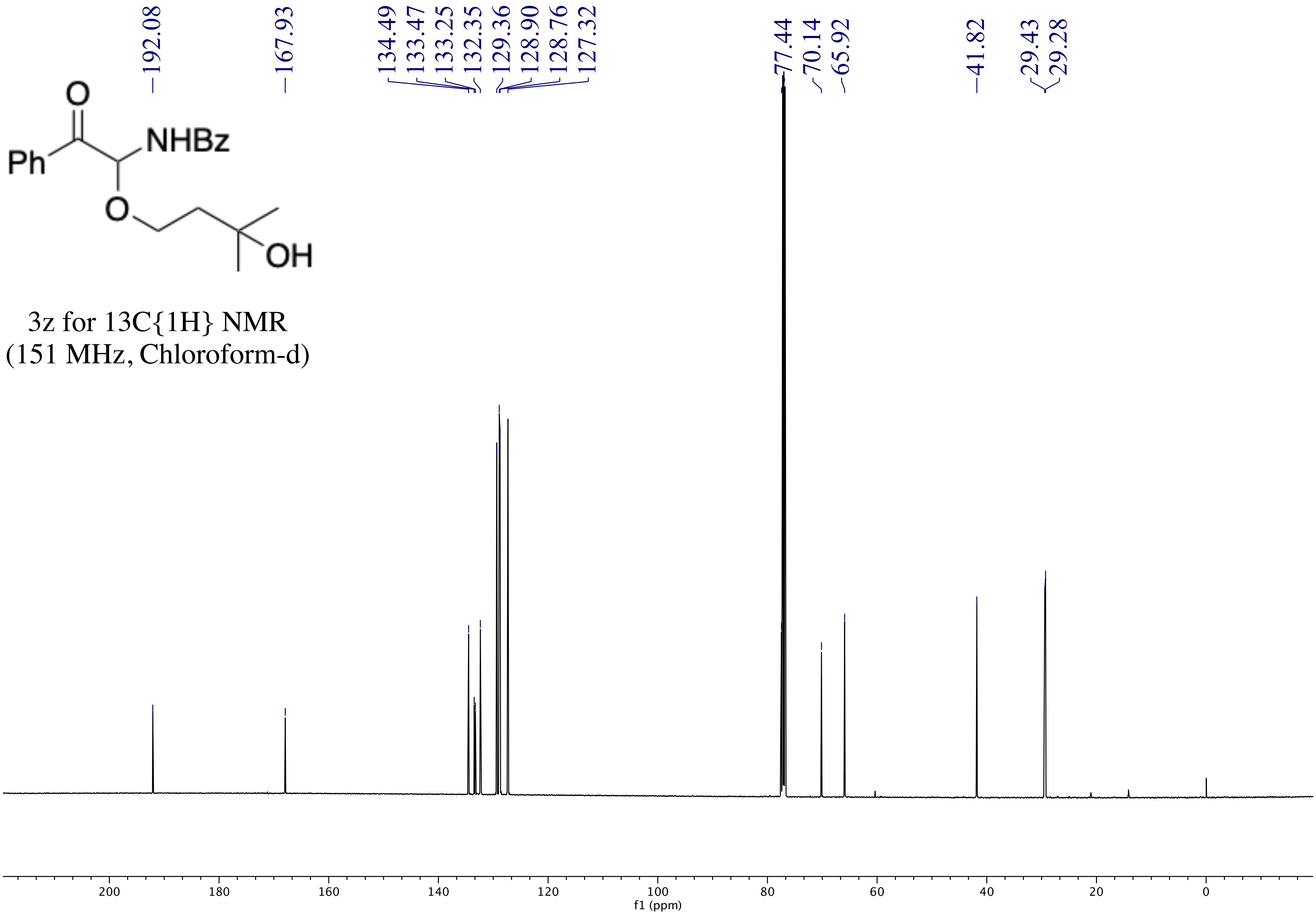

3z for 13C{1H} NMR
(151 MHz, Chloroform-d)

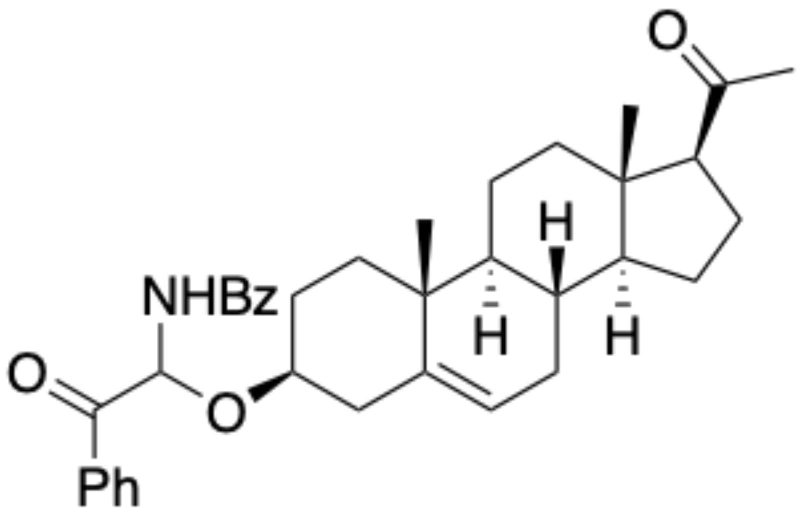

3aa for 1H NMR
(600 MHz, Chloroform-d)

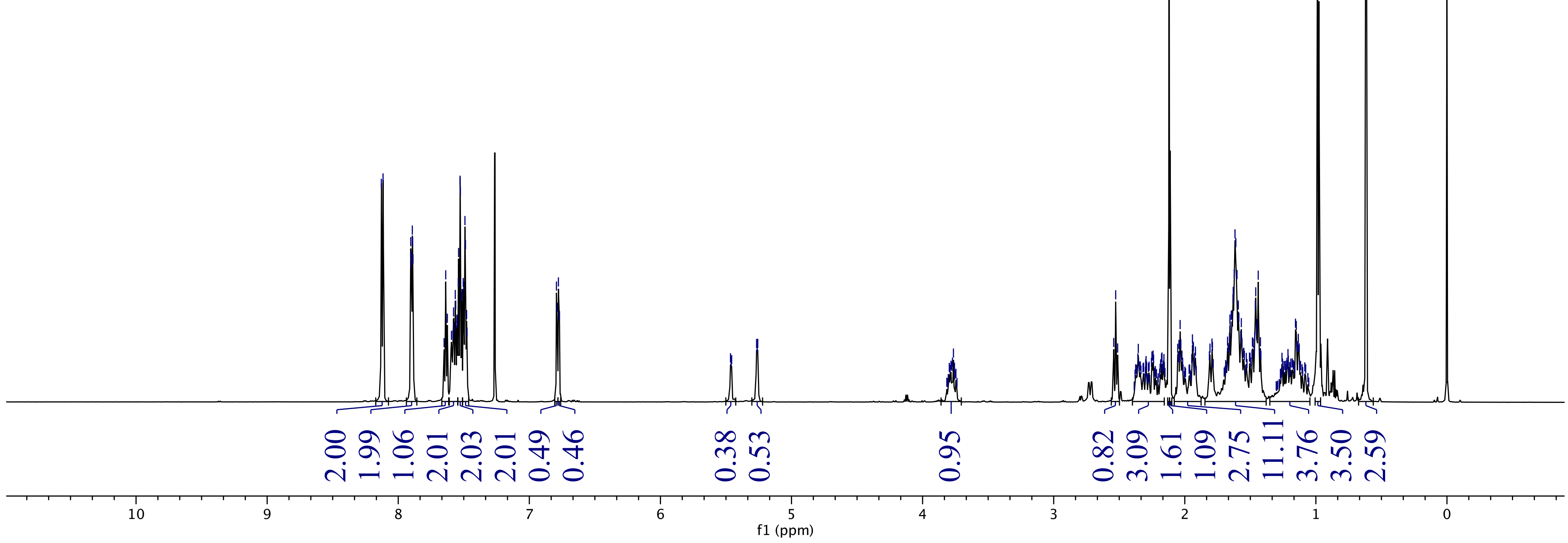

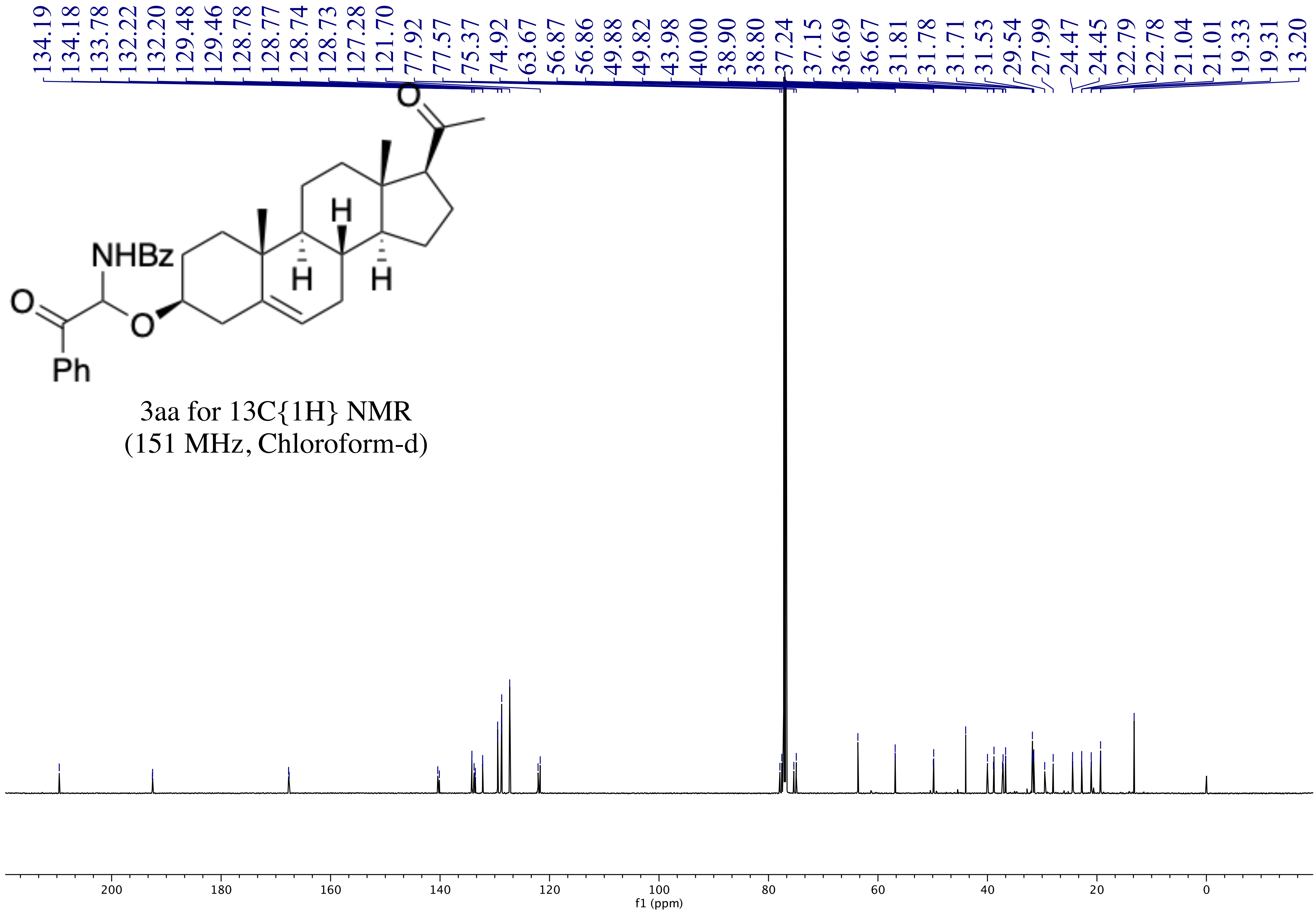

3aa for 13C{1H} NMR
(151 MHz, Chloroform-d)

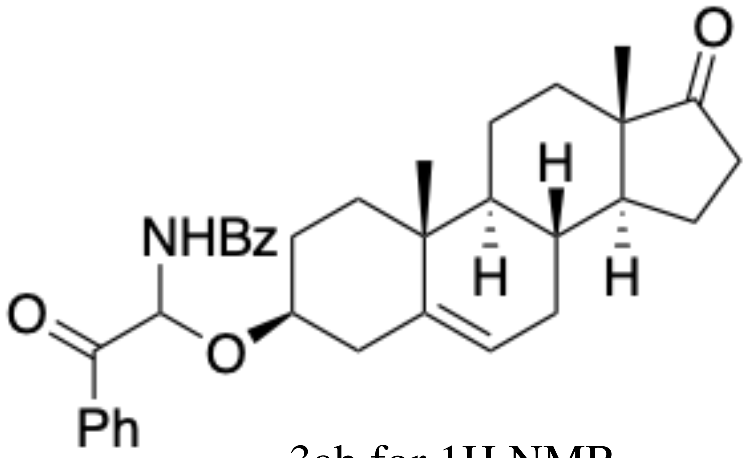

3ab for 1H NMR
(600 MHz, Chloroform-d)

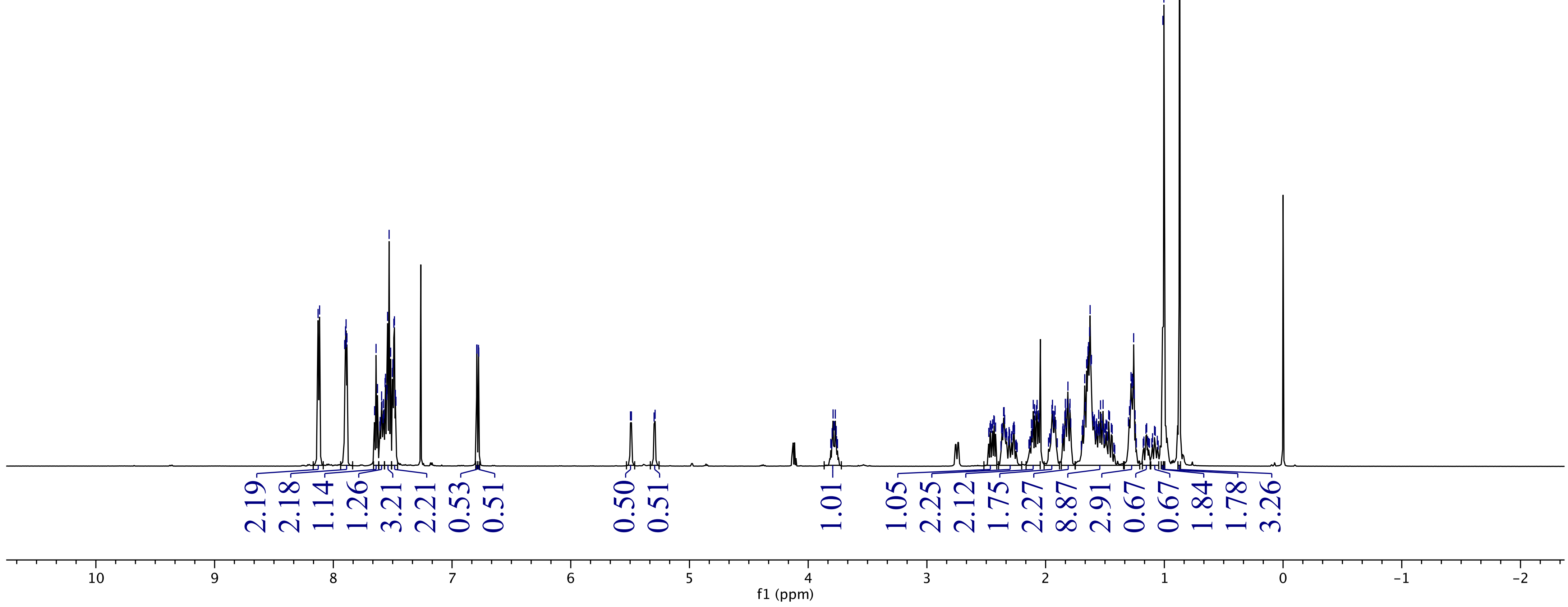

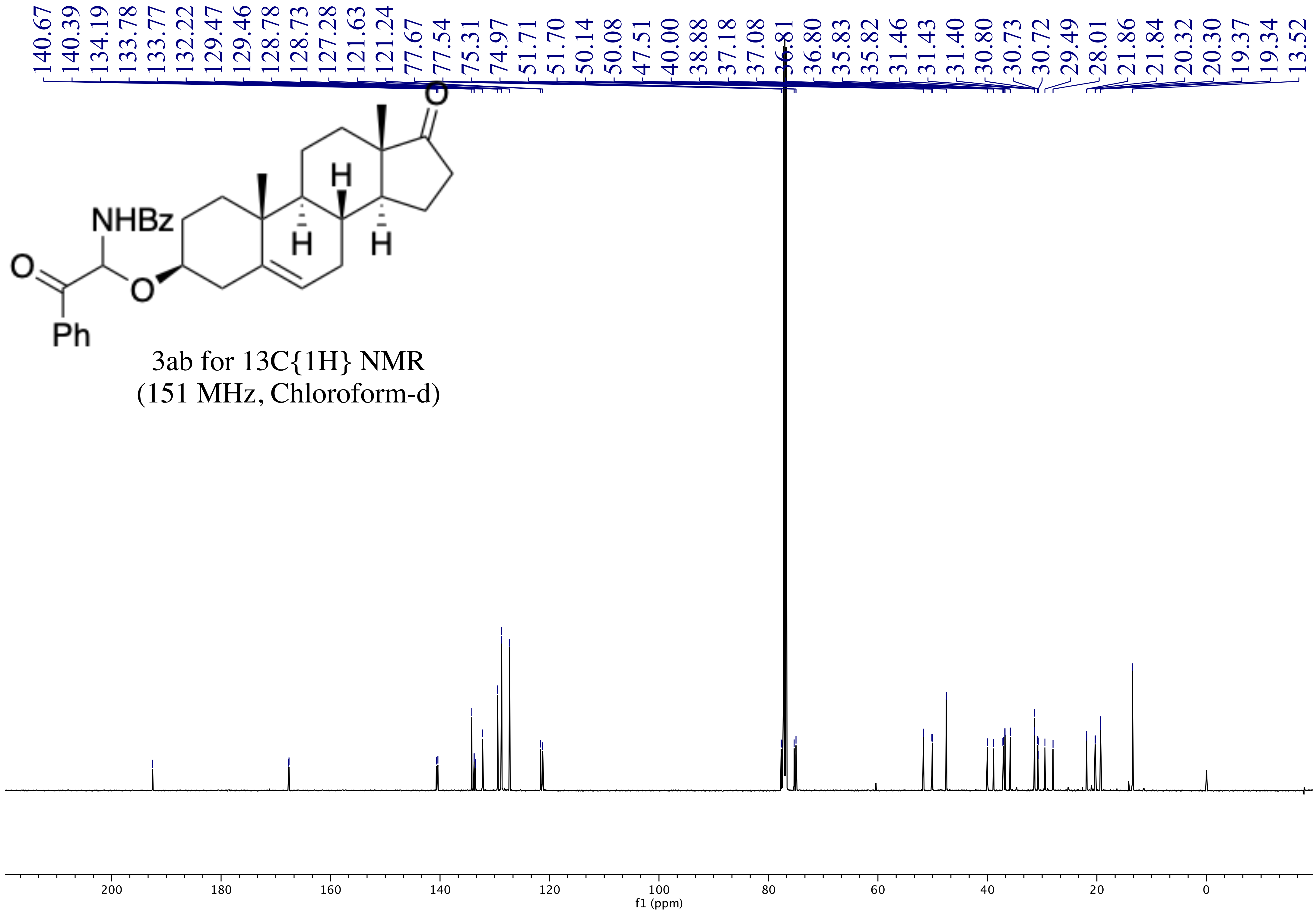

3ab for 13C{1H} NMR
(151 MHz, Chloroform-d)

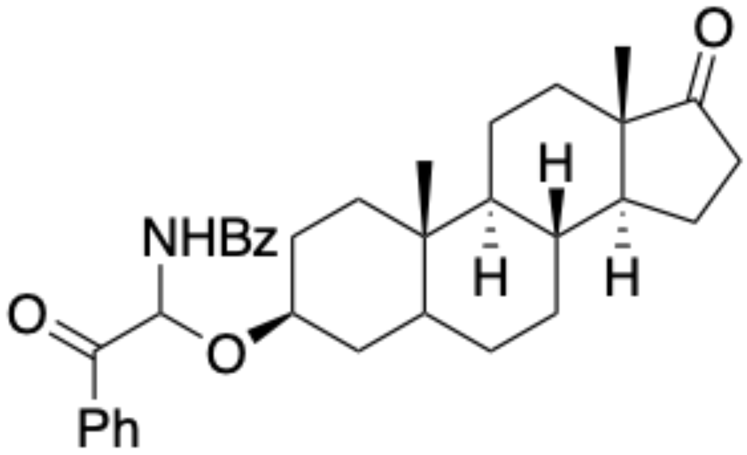

3ac for 1H NMR
(600 MHz, Chloroform-d)

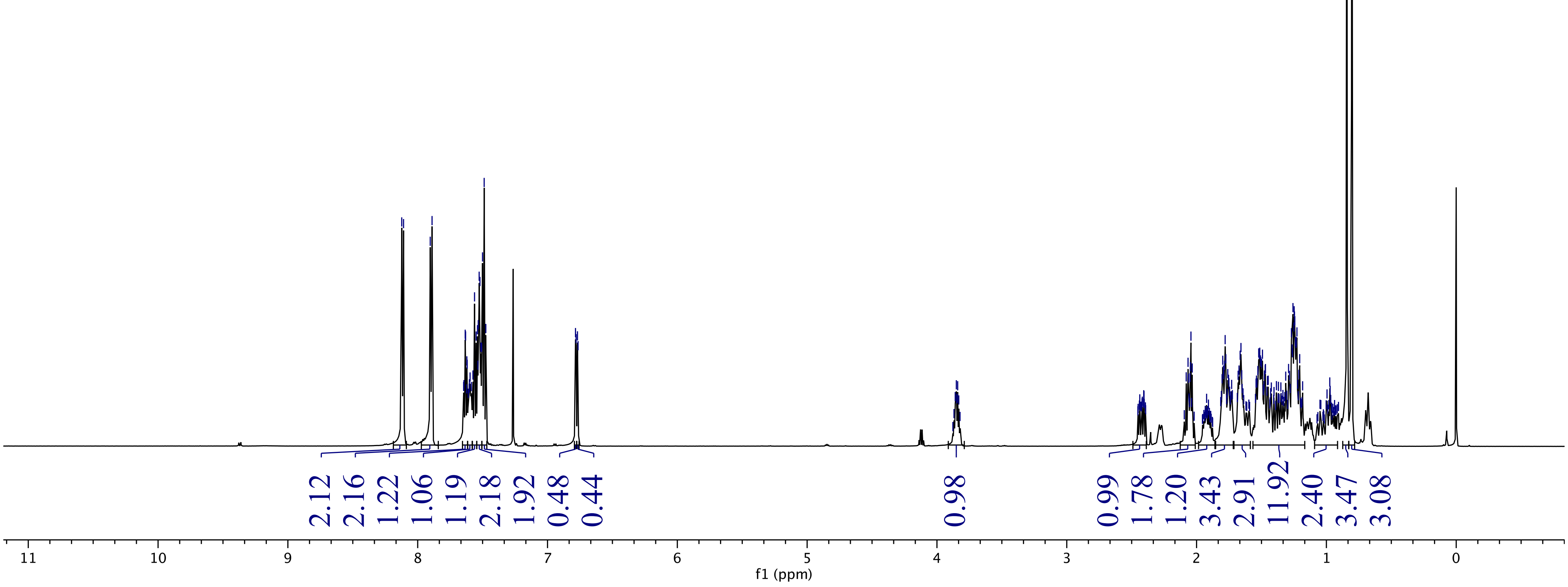

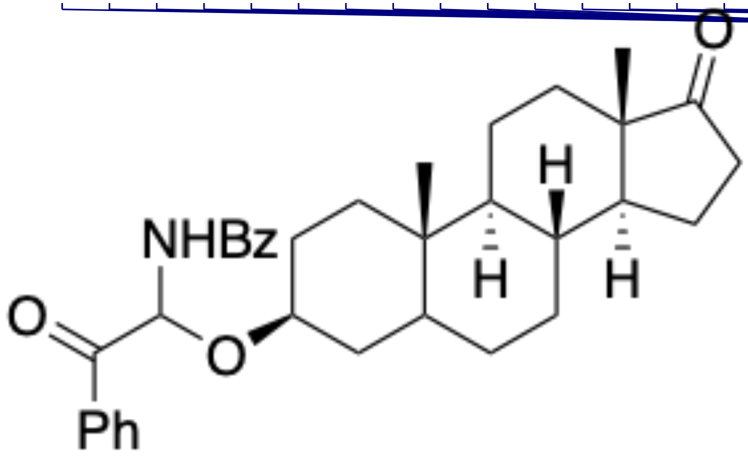

3ac for 13C{1H} NMR
(151 MHz, Chloroform-d)

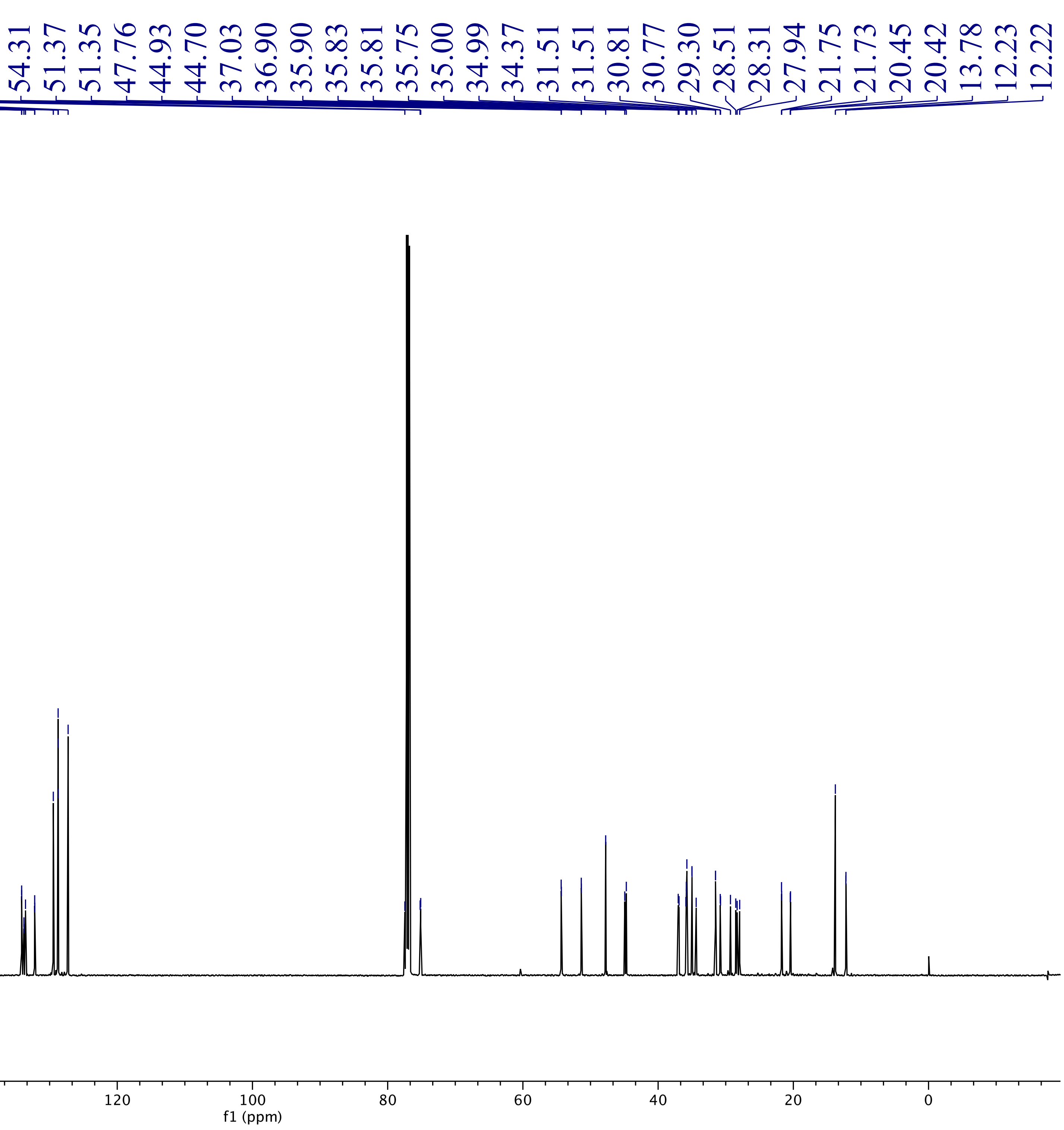

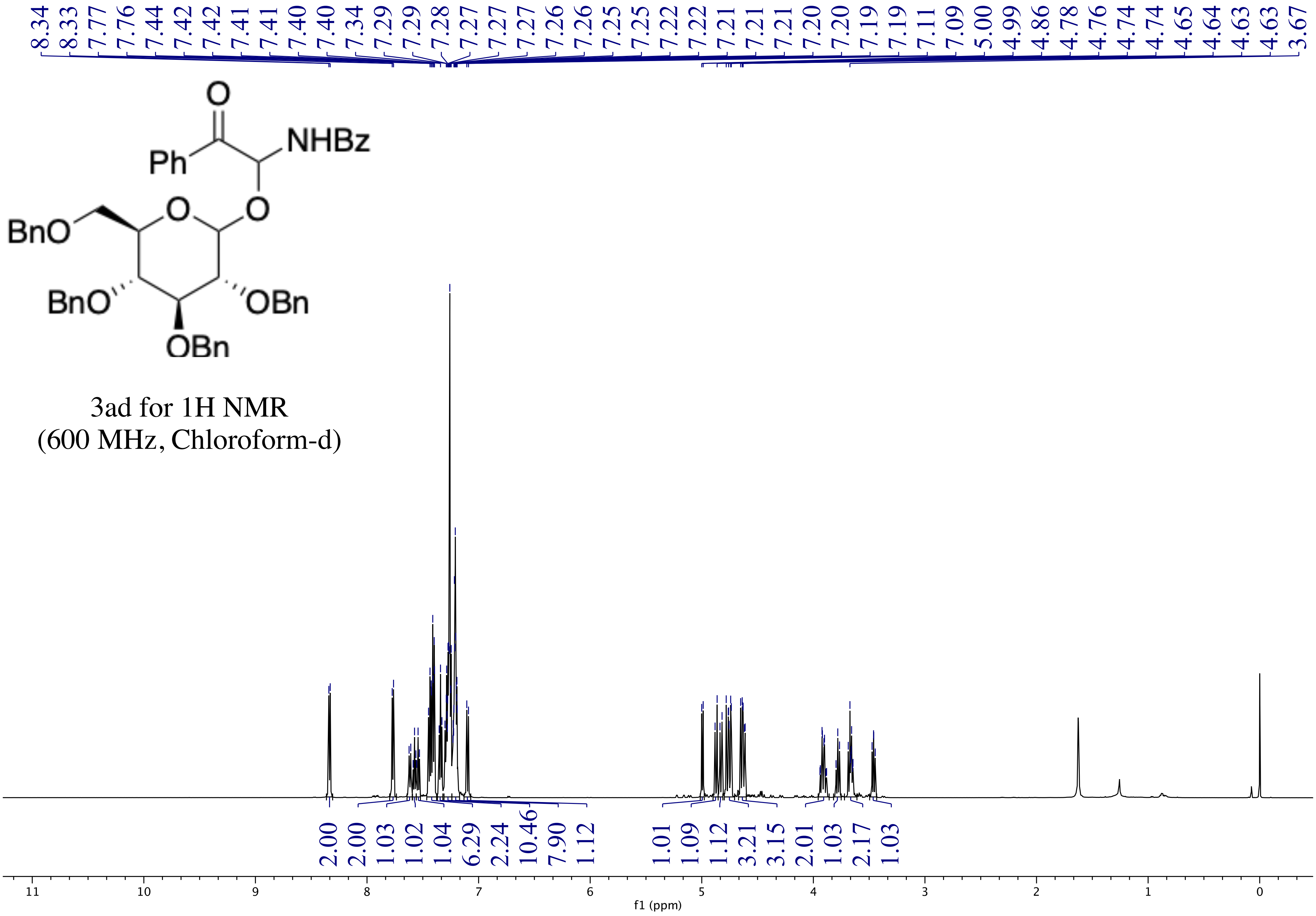

3ad for 1H NMR
(600 MHz, Chloroform-d)

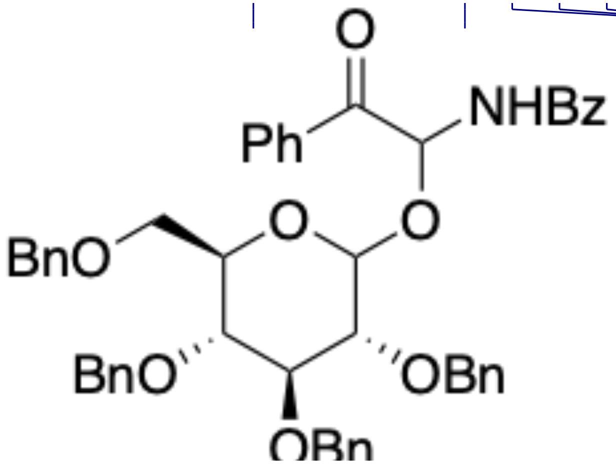

3ad for 13C{1H} NMR
(151 MHz, Chloroform-d)

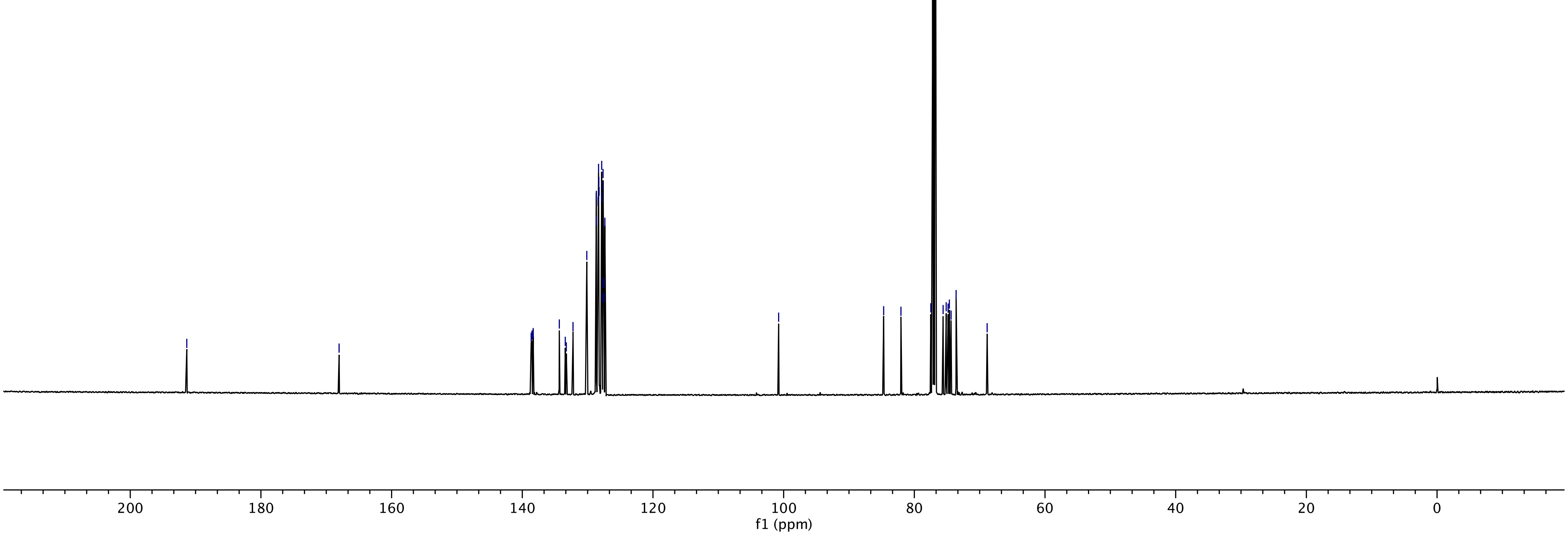

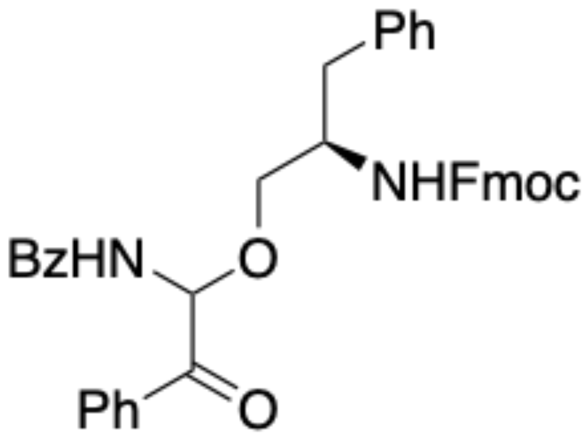

3ae for 1H NMR
(600 MHz, Chloroform-d)

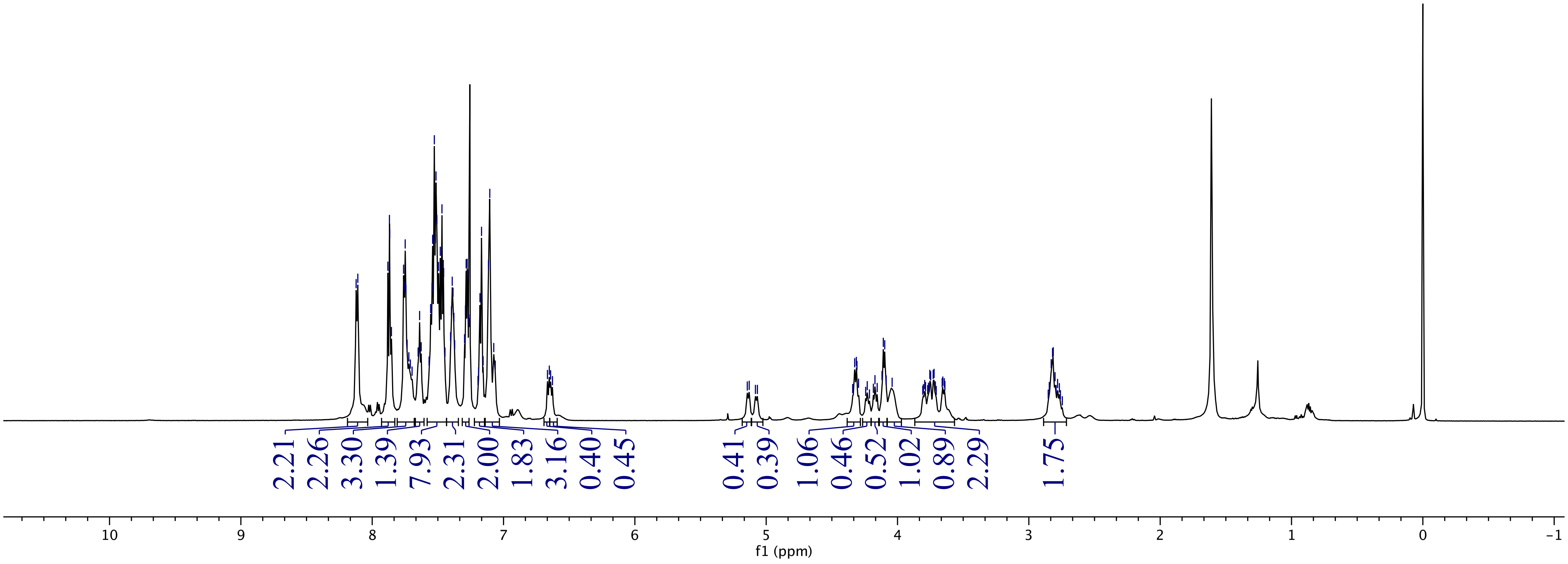

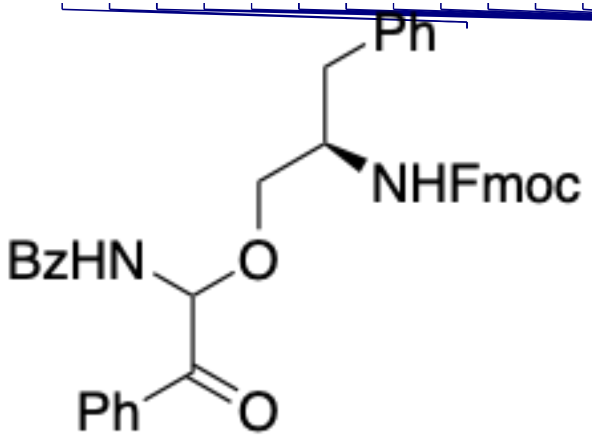

3ae for 13C{1H} NMR
(151 MHz, Chloroform-d)

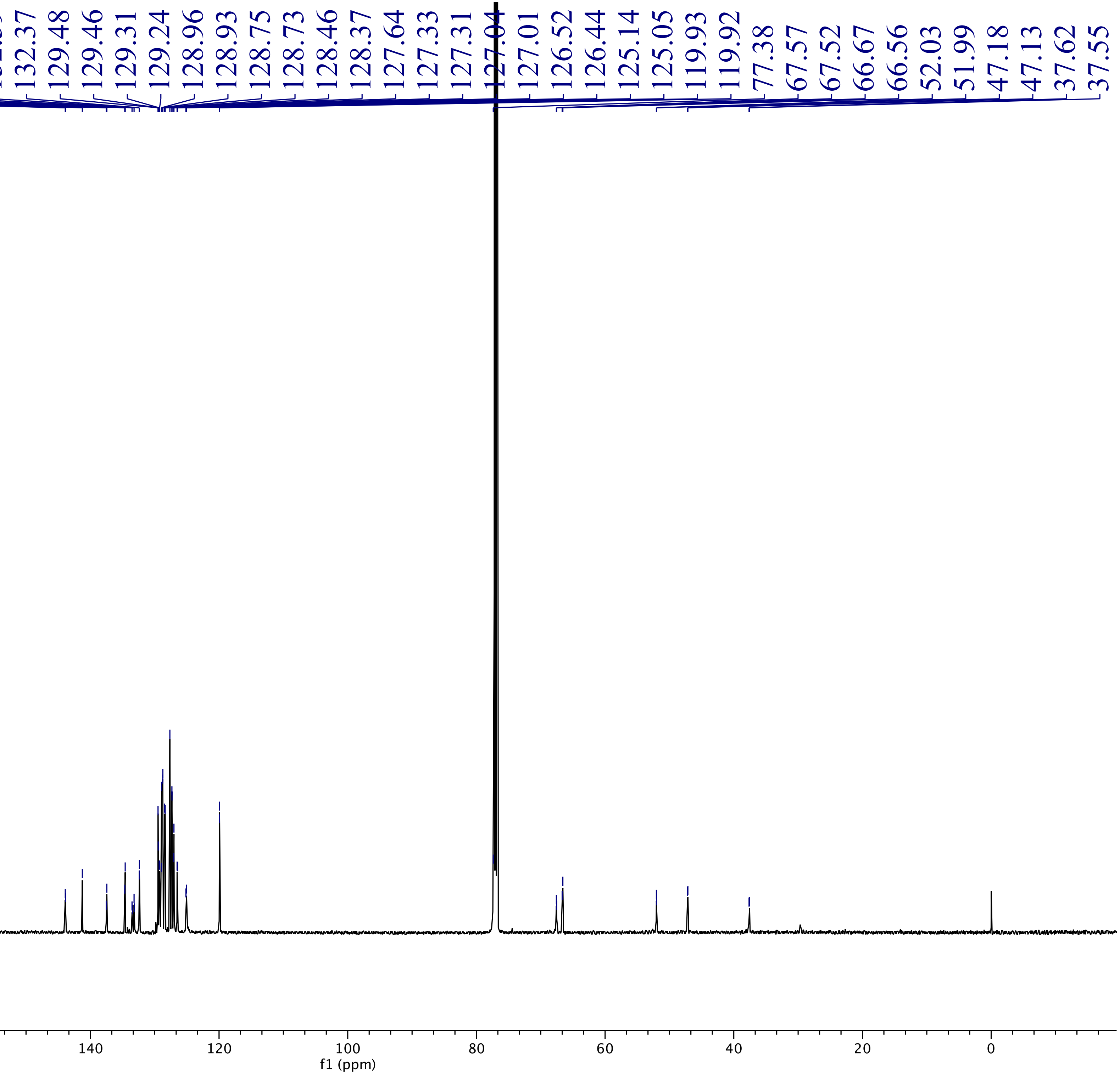

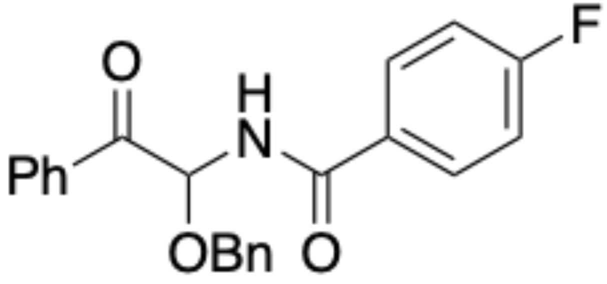

3af for 1H NMR
(600 MHz, Chloroform-d)

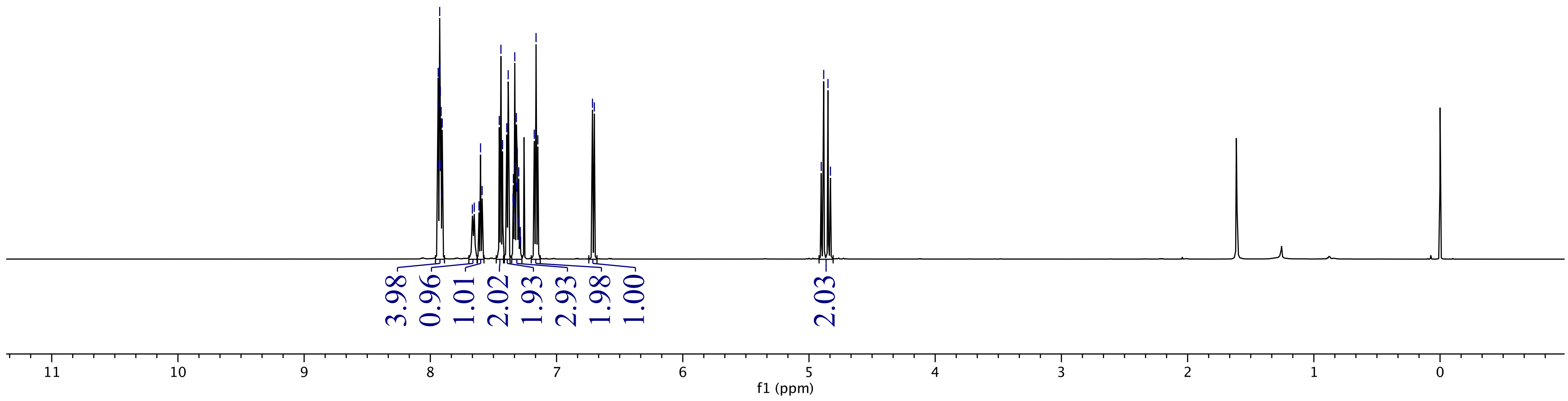

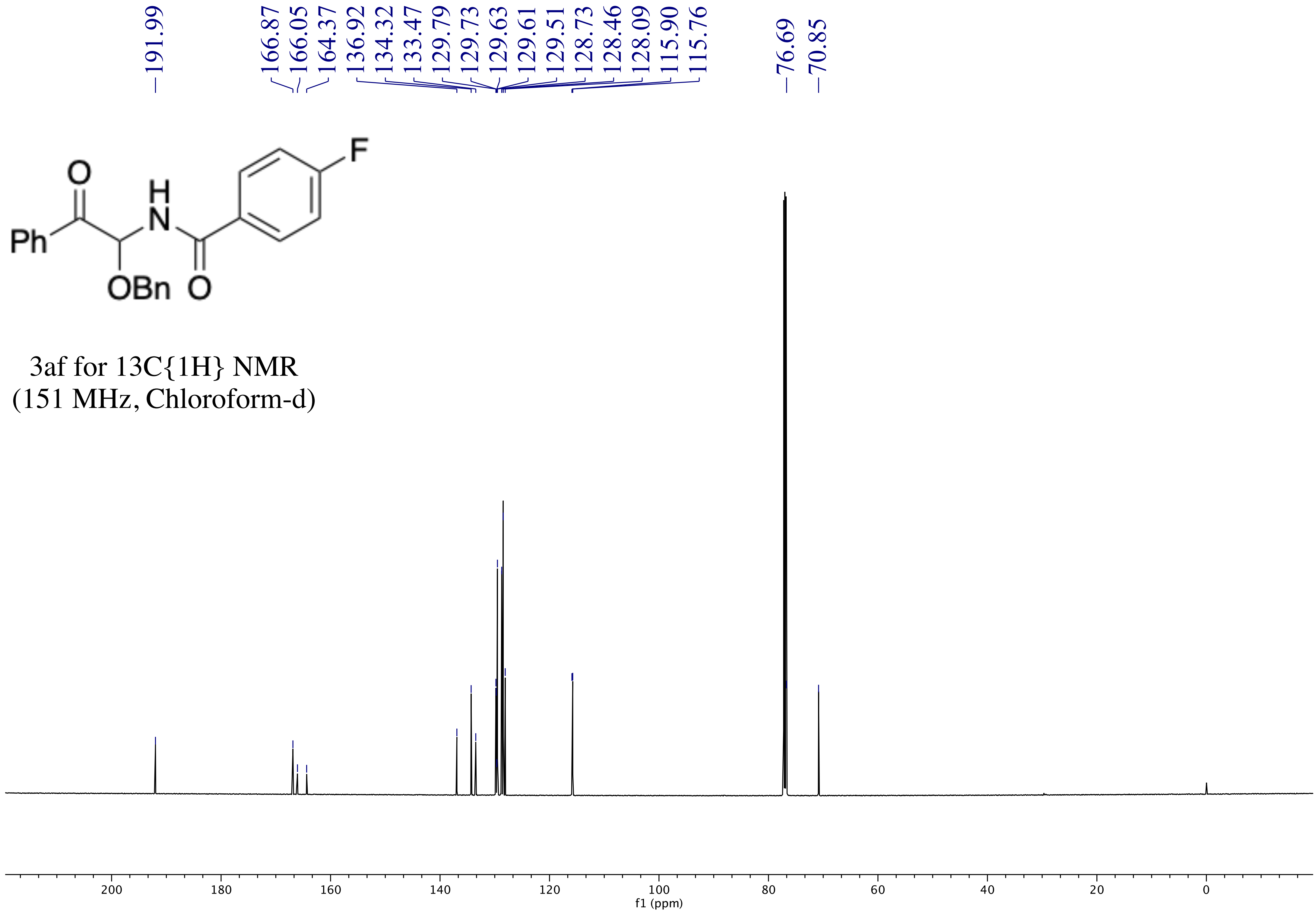

191.99
166.87
166.05
164.37
136.92
134.32
133.47
129.79
129.73
129.63
129.61
129.51
128.73
128.46
128.09
115.90
115.76
76.69
70.85
Ph
O
H
N
OBn
O
F
3af for 13C{1H} NMR
(151 MHz, Chloroform-d)
200
180
160
140
120
100
80
60
40
20
0
f1 (ppm)

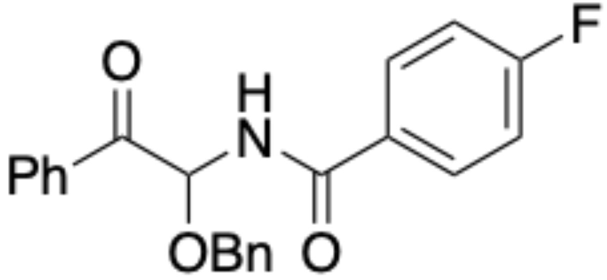

3af for 19F NMR
(376 MHz, Chloroform-d)

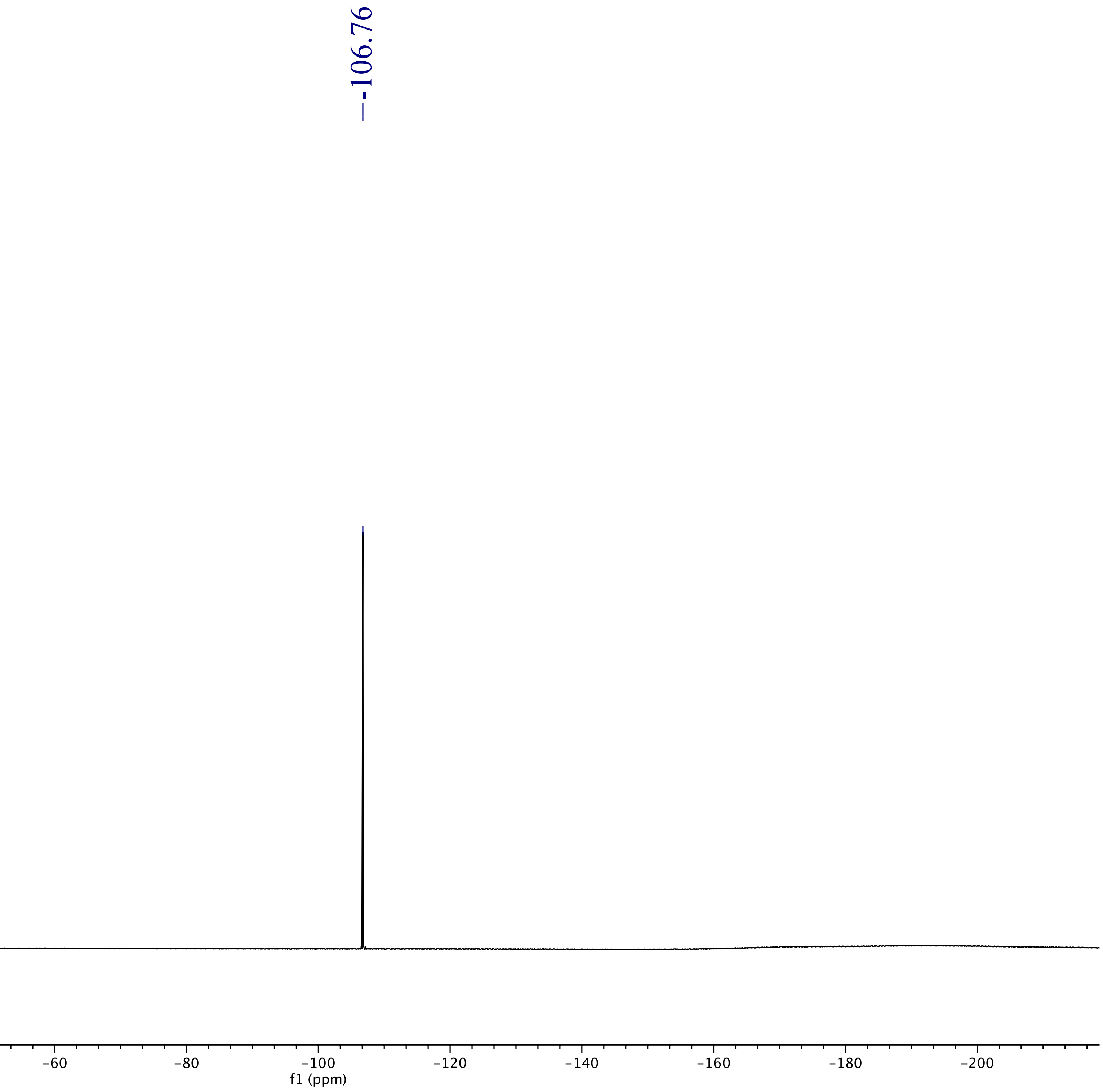

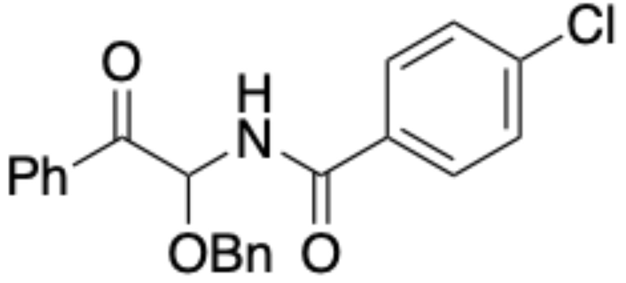

3ag for 1H NMR
(600 MHz, Chloroform-d)

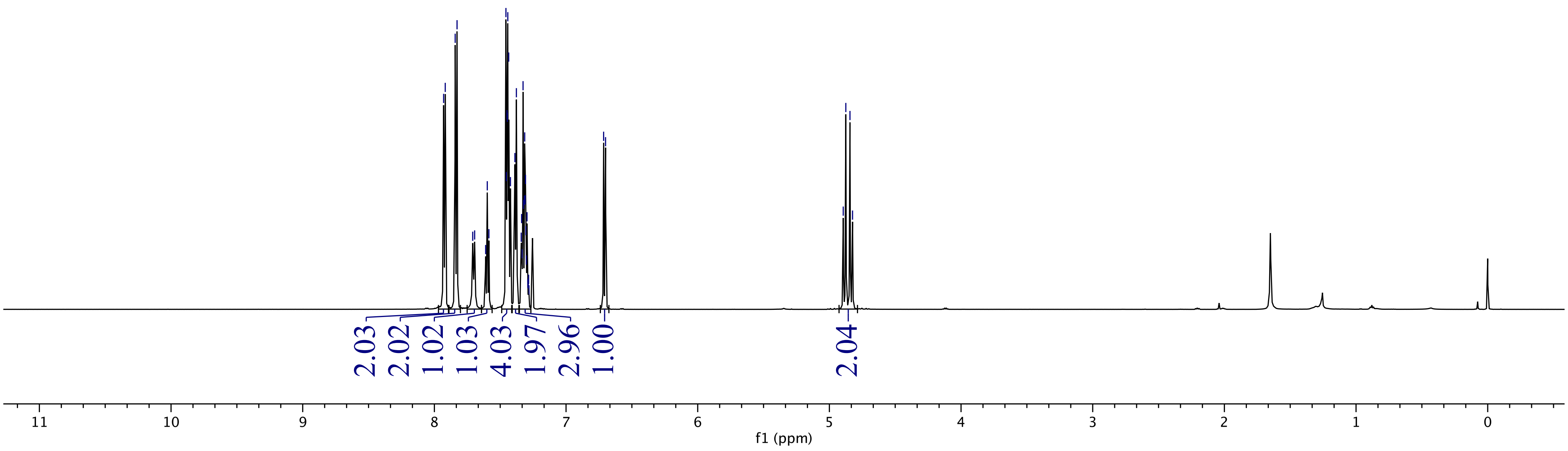

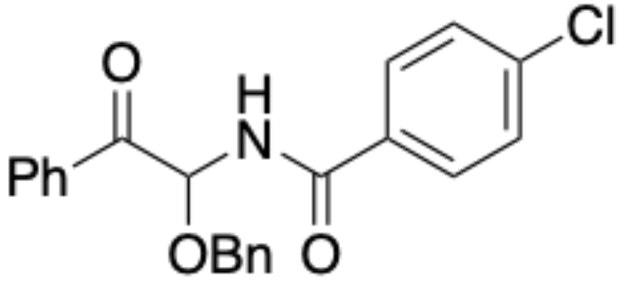

3ag for 13C{1H} NMR
(151 MHz, Chloroform-d)

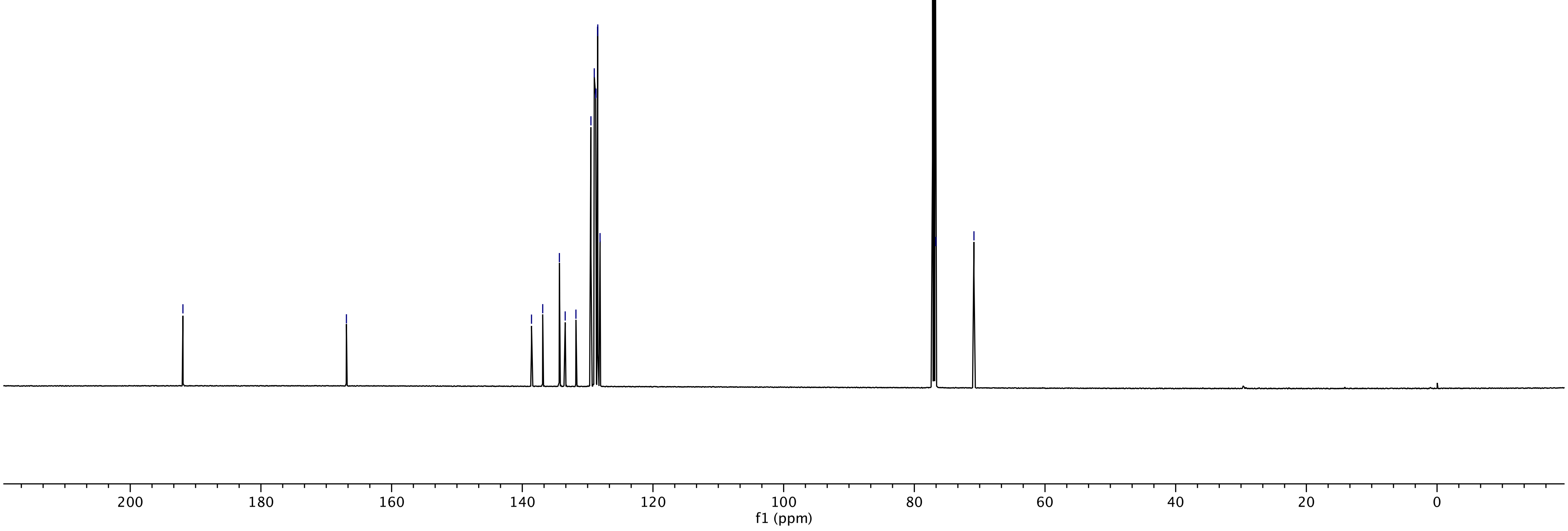

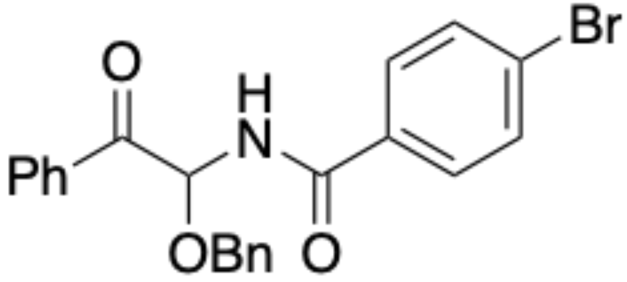

3ah for 1H NMR
(600 MHz, Chloroform-d)

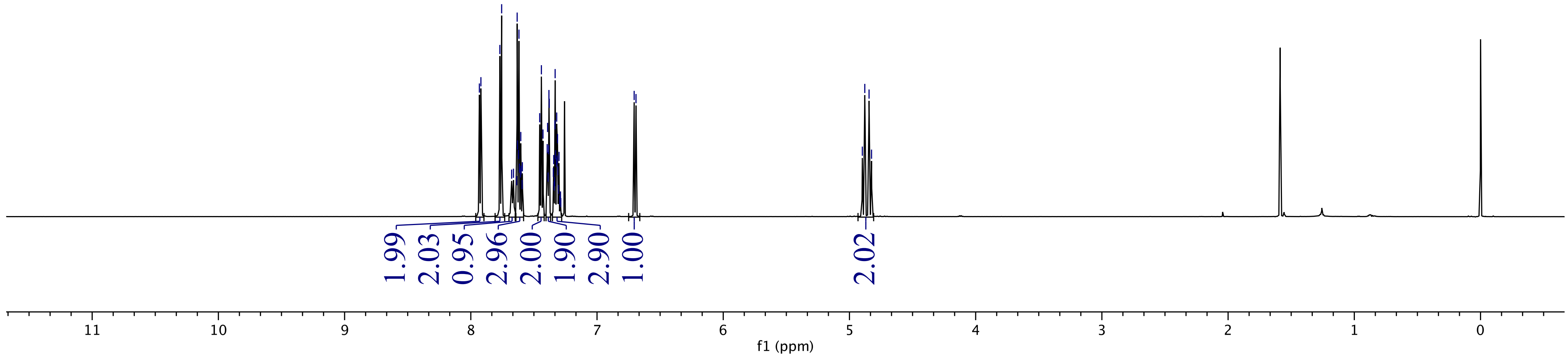

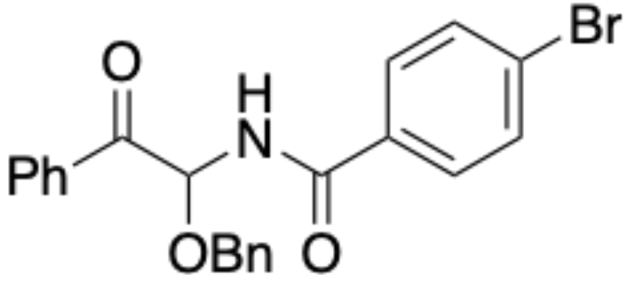

3ah for 13C{1H} NMR
(151 MHz, Chloroform-d)

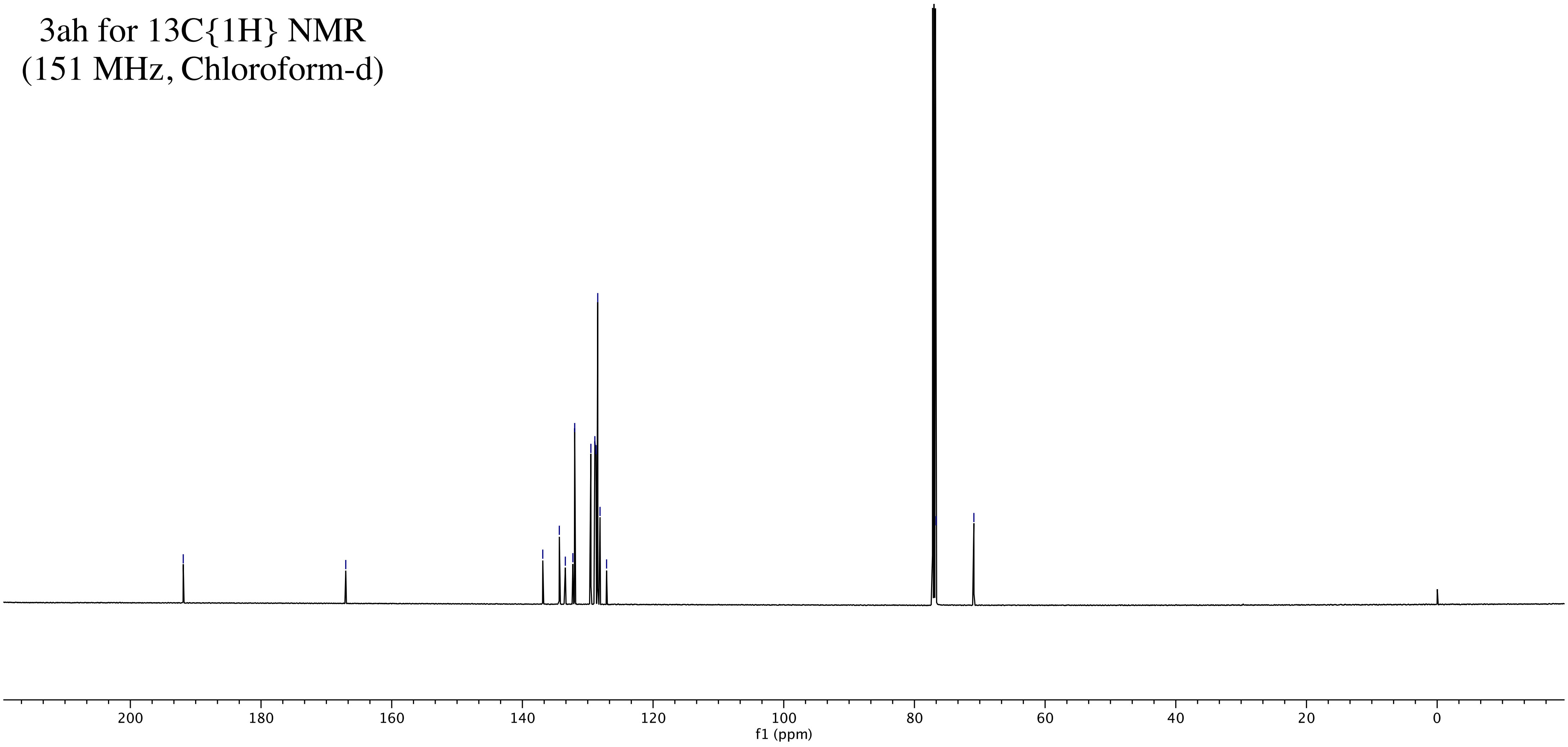

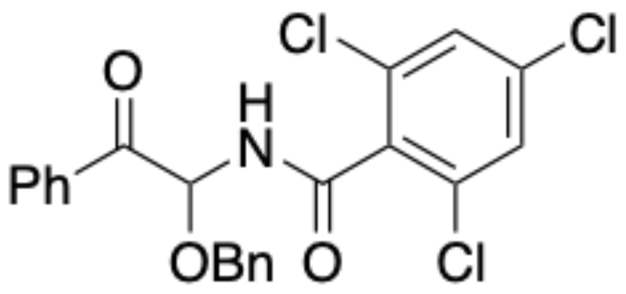

3ai for 1H NMR
(600 MHz, Chloroform-d)

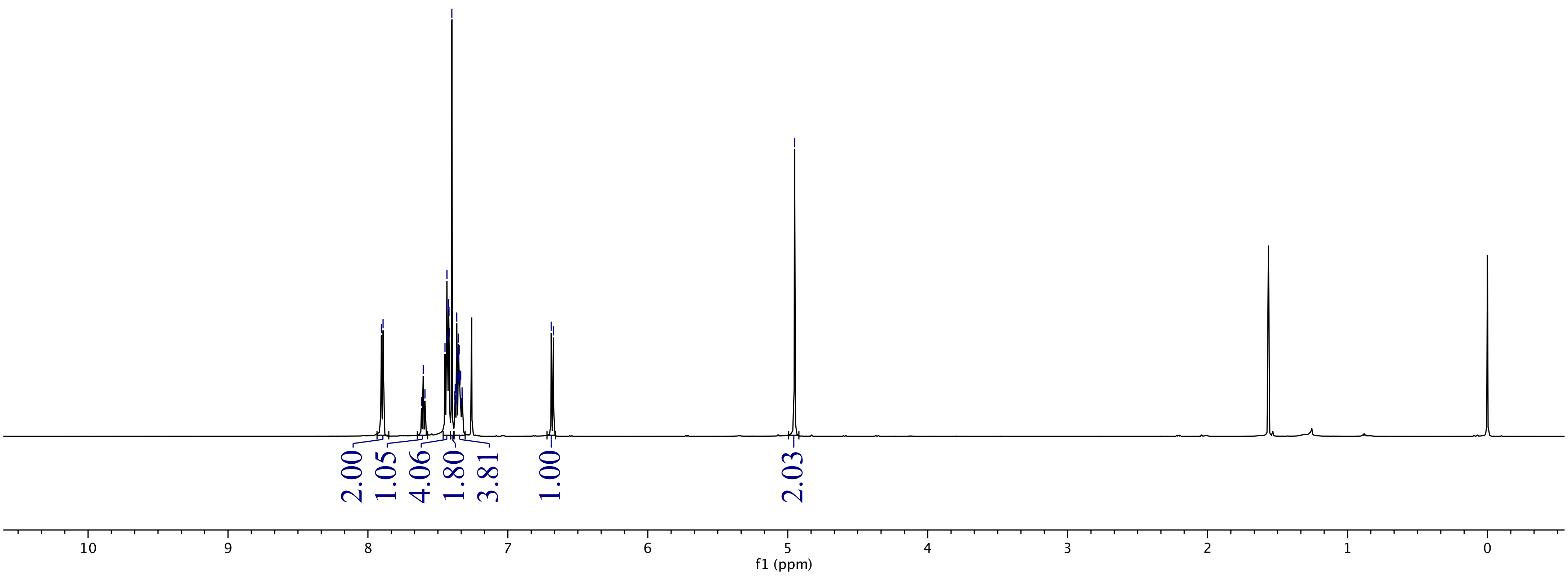

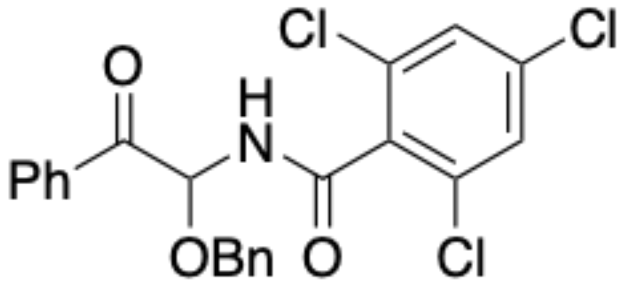

3ai for 13C{1H} NMR
(151 MHz, Chloroform-d)

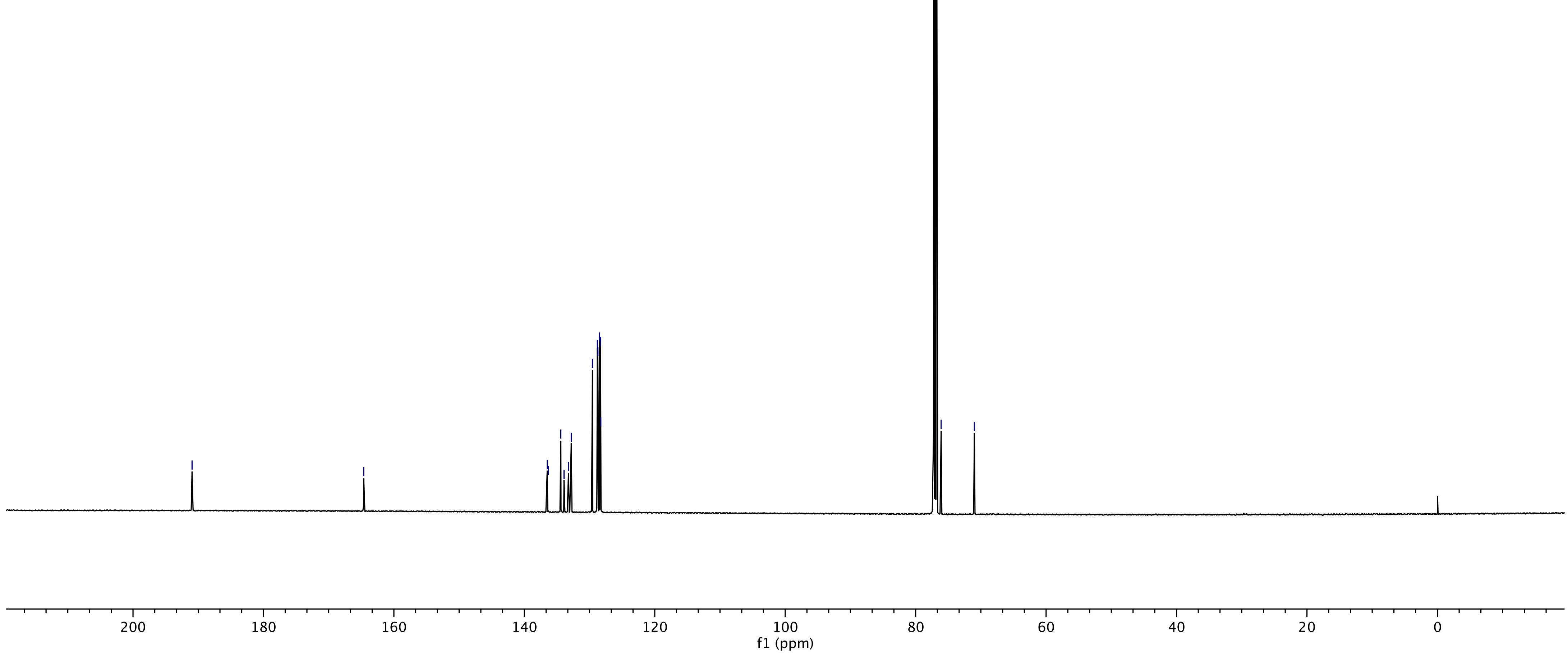

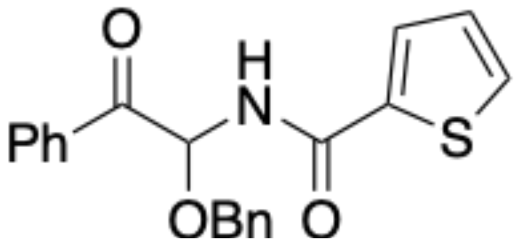

3aj for 1H NMR
(600 MHz, Chloroform-d)

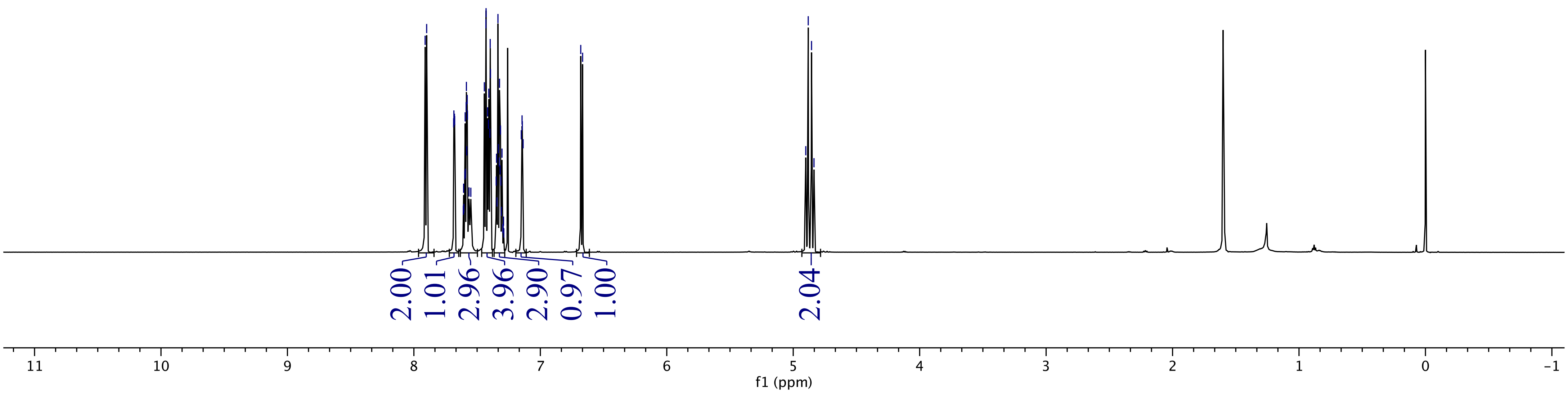

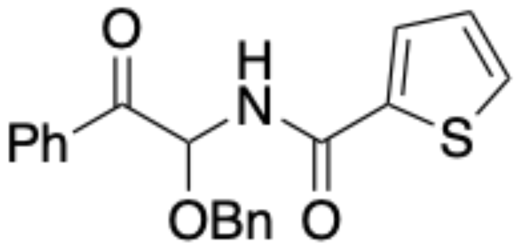

3aj for 13C{1H} NMR
(151 MHz, Chloroform-d)

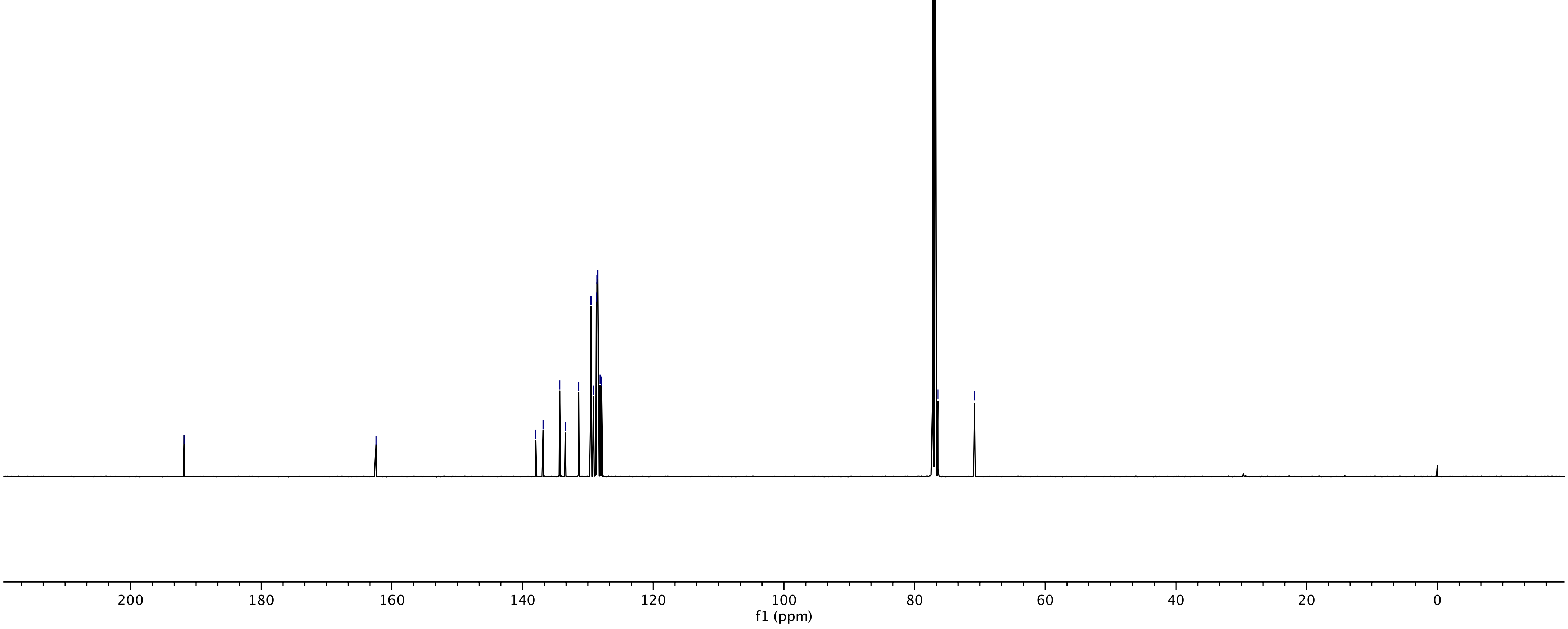

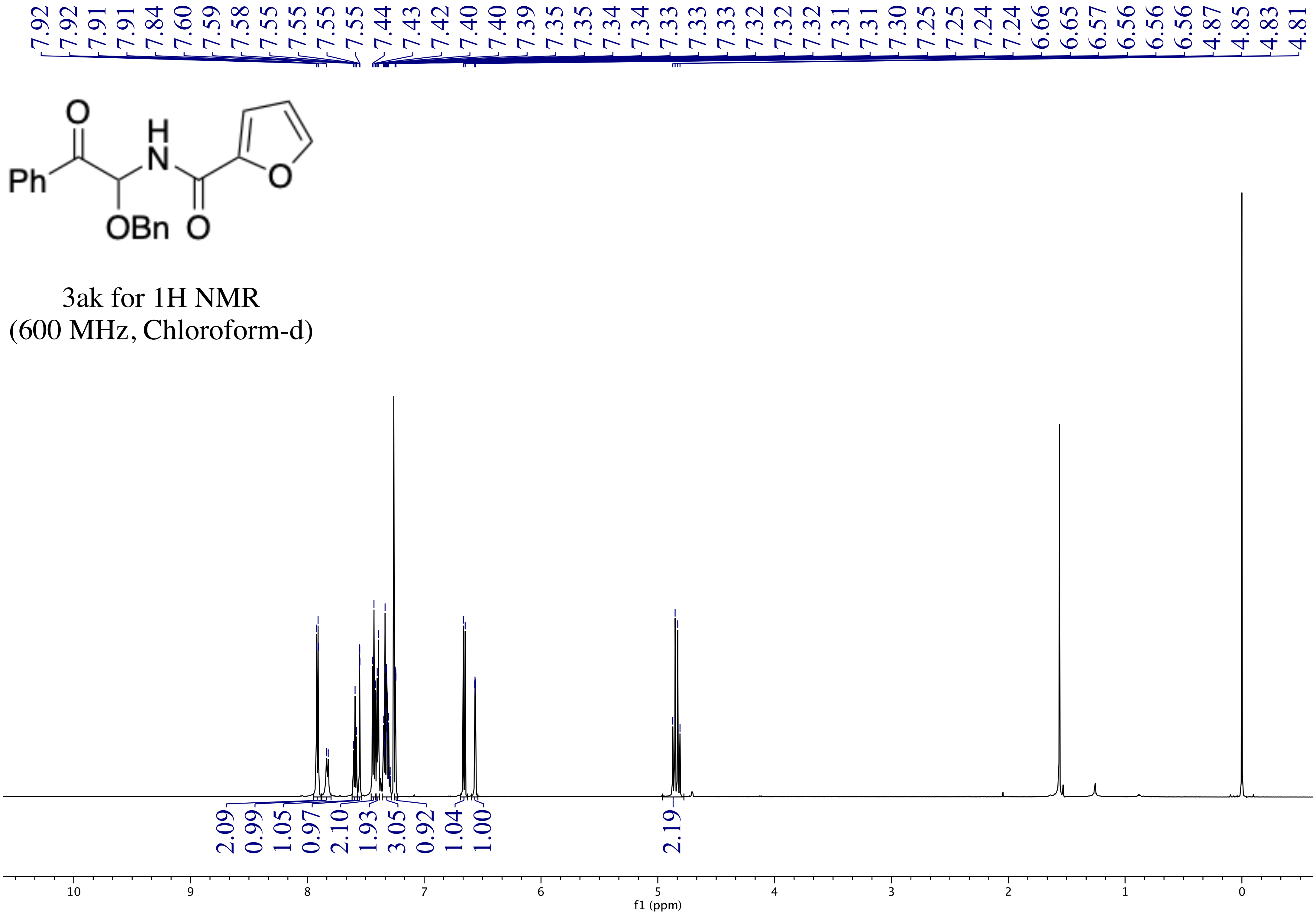

3ak for 1H NMR
(600 MHz, Chloroform-d)

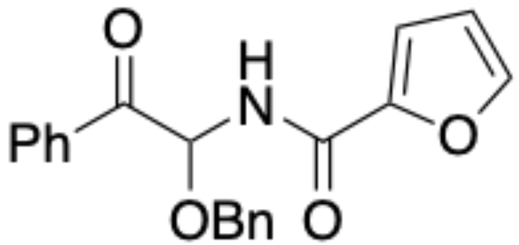

3ak for 13C{1H} NMR
(151 MHz, Chloroform-d)

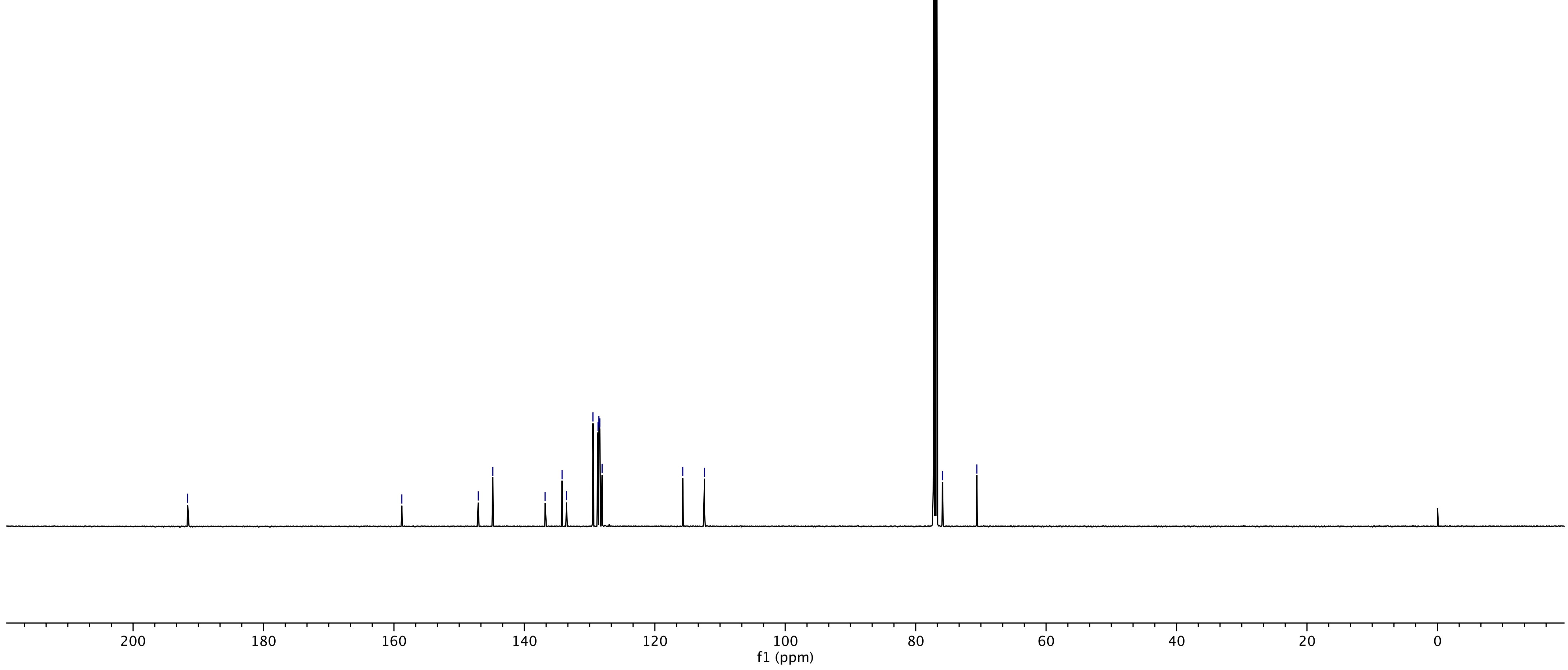

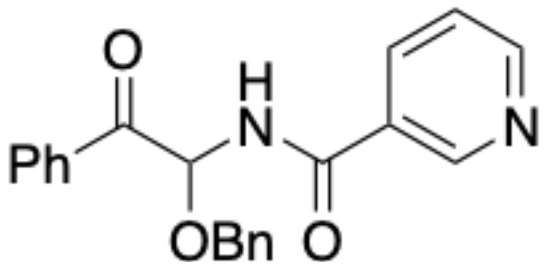

3al for 1H NMR
(600 MHz, Chloroform-d)

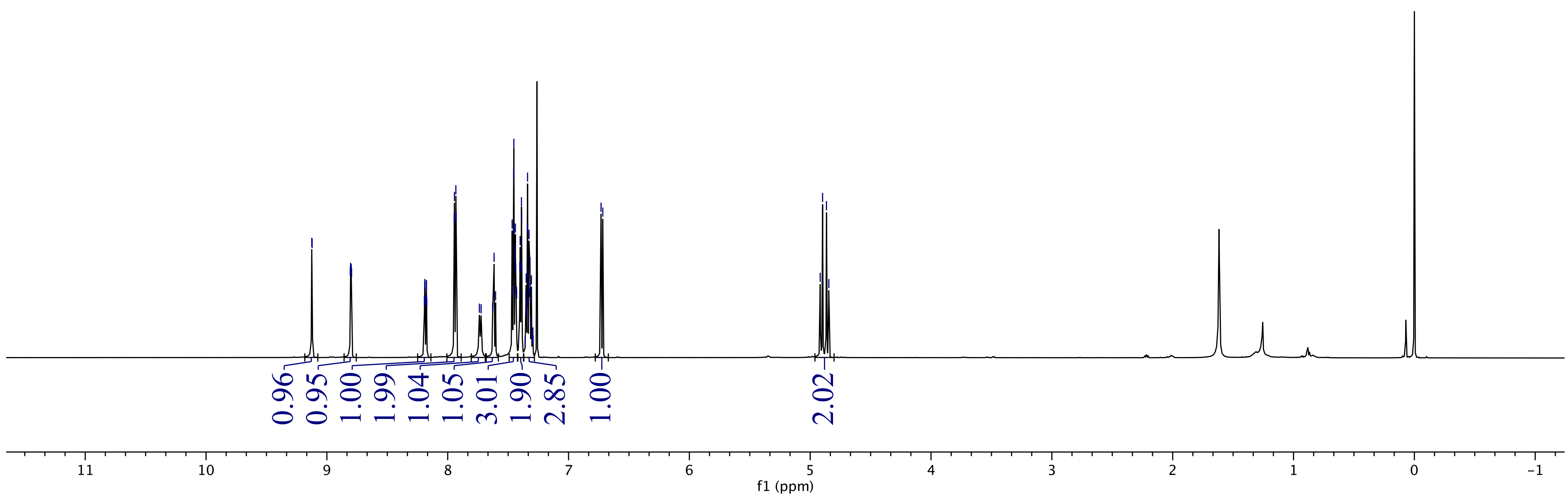

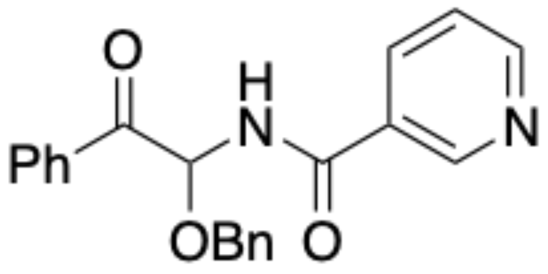

3al for 13C{1H} NMR
(151 MHz, Chloroform-d)

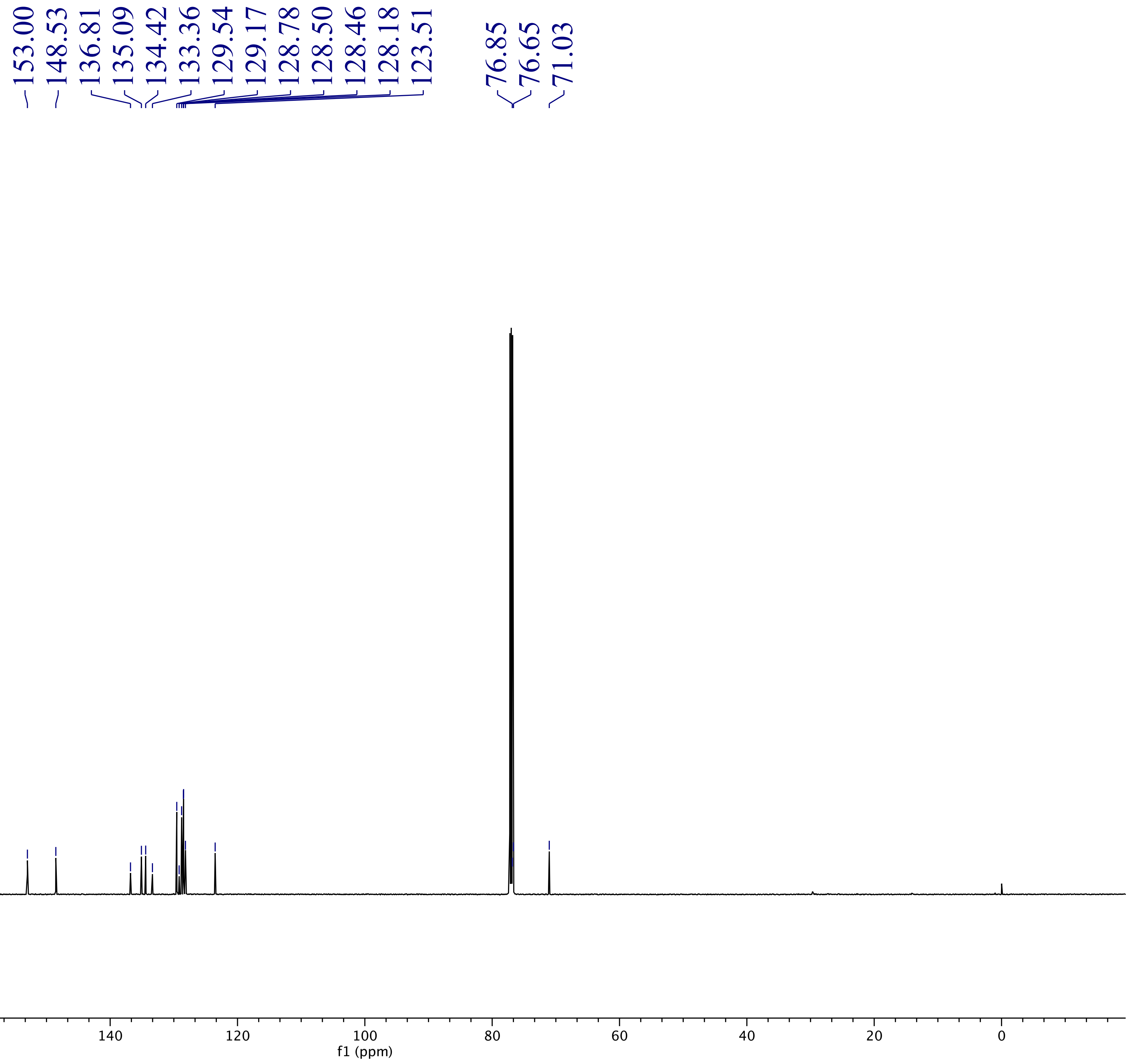

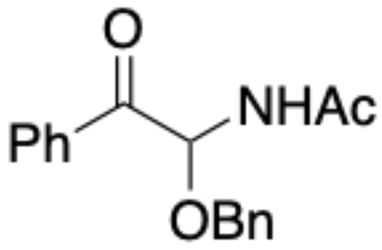

3am for 1H NMR
(600 MHz, Chloroform-d)

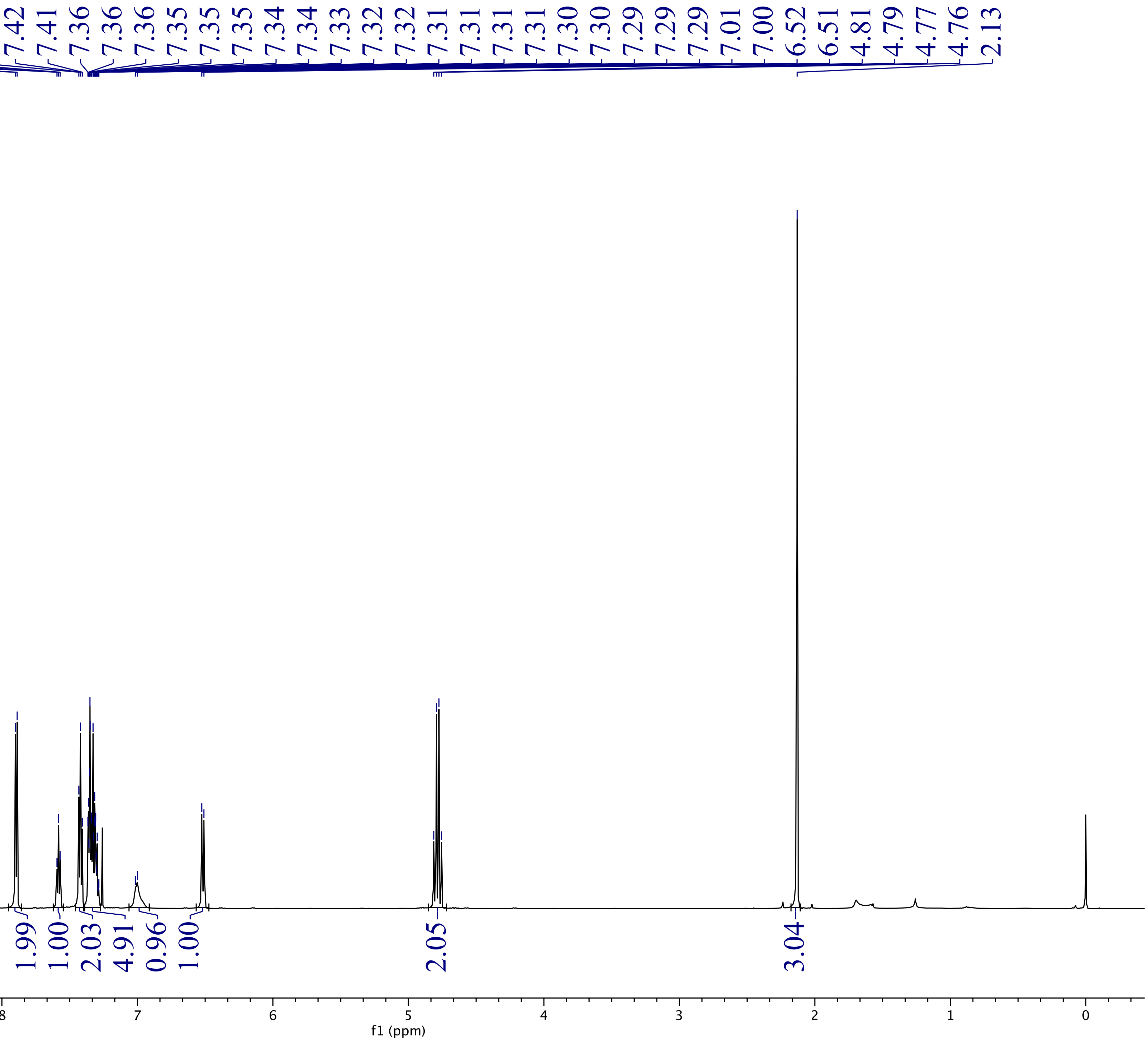

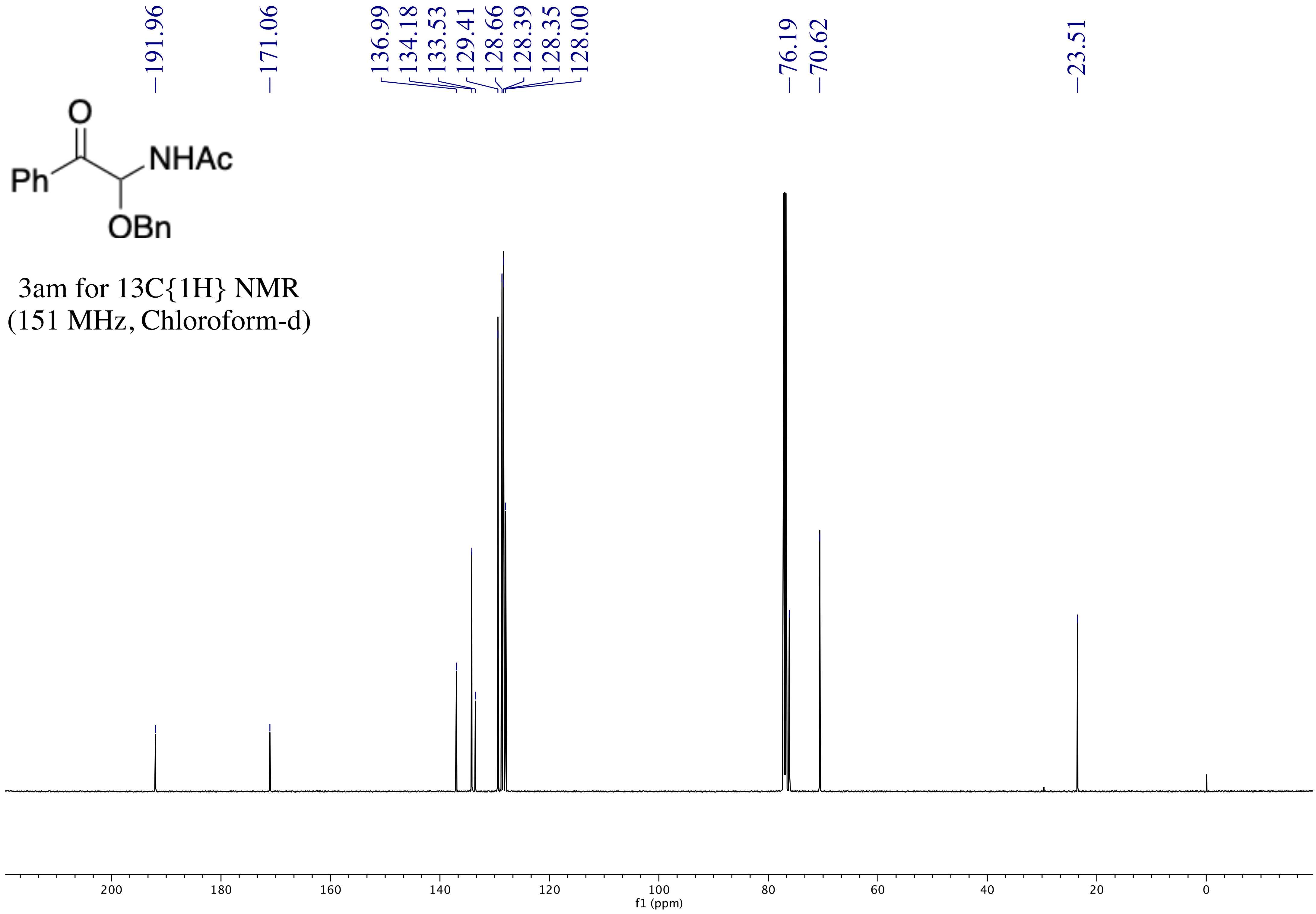
191.96
171.06
136.99
134.18
133.53
129.41
128.66
128.39
128.35
128.00
76.19
70.62
23.51
Ph
O
NHAc
OBn
3am for 13C{1H} NMR
(151 MHz, Chloroform-d)
200
180
160
140
120
100
80
60
40
20
0
f1 (ppm)

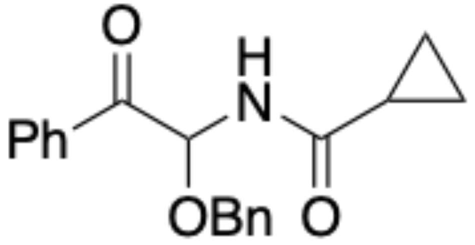

3an for 1H NMR
(600 MHz, Chloroform-d)

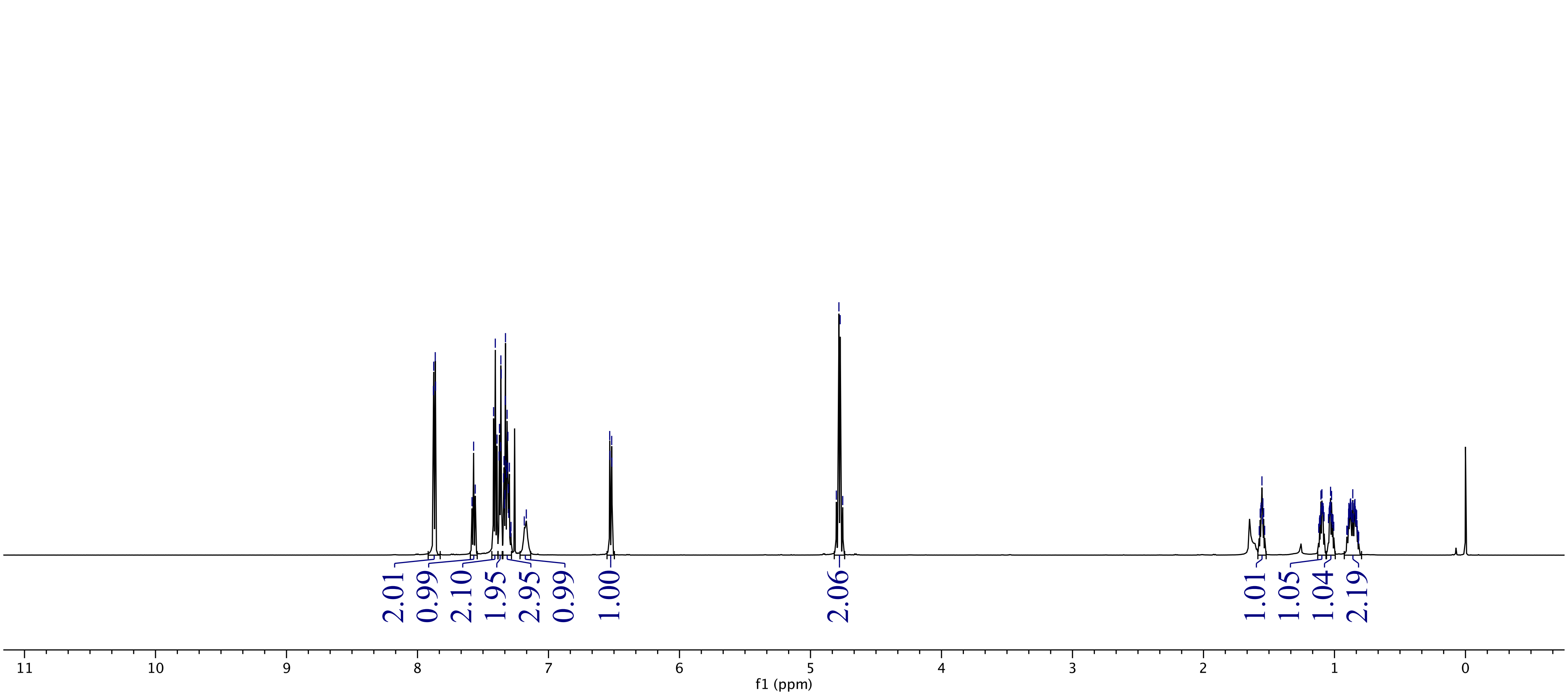

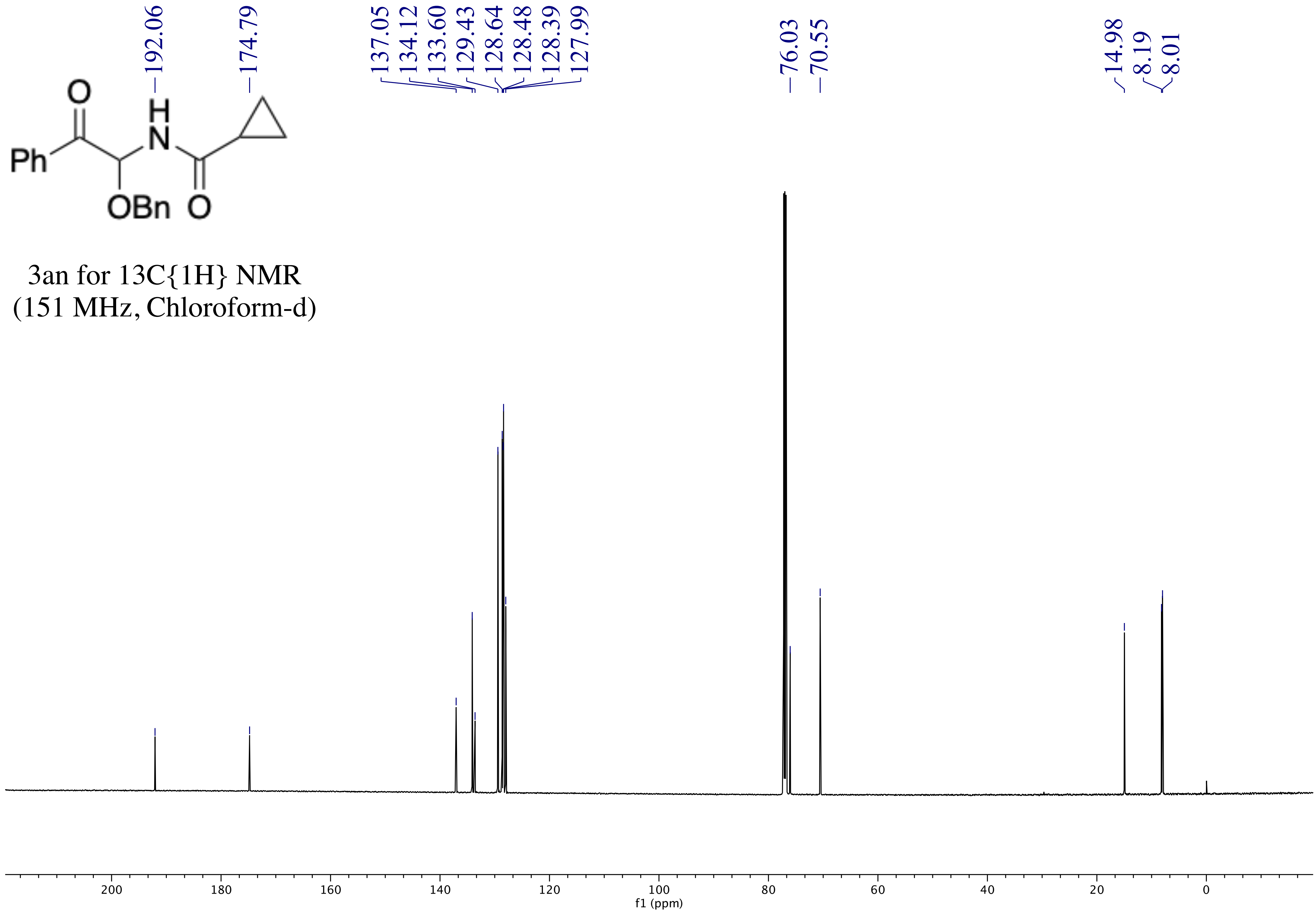

3an for 13C{1H} NMR
(151 MHz, Chloroform-d)
Ph
O
H
N
OBn
O
192.06
174.79
137.05
134.12
133.60
129.43
128.64
128.48
128.39
127.99
76.03
70.55
14.98
8.19
8.01
200
180
160
140
120
100
80
60
40
20
0
f1 (ppm)

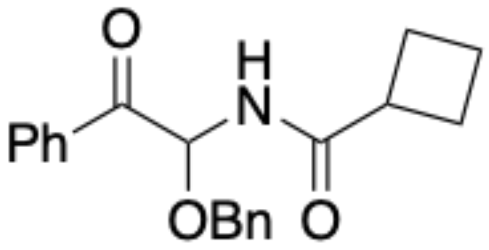

3ao for 1H NMR
(600 MHz, Chloroform-d)

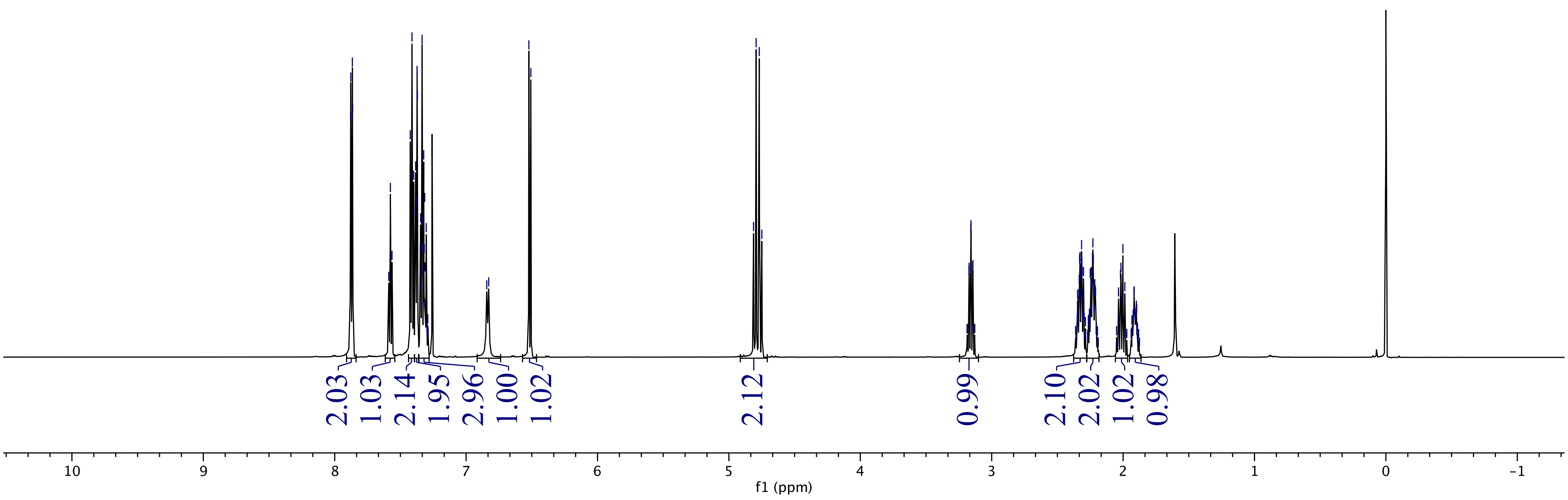

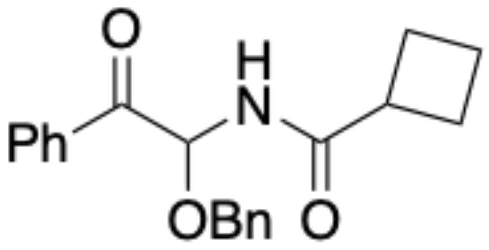

3ao for 13C{1H} NMR
(151 MHz, Chloroform-d)

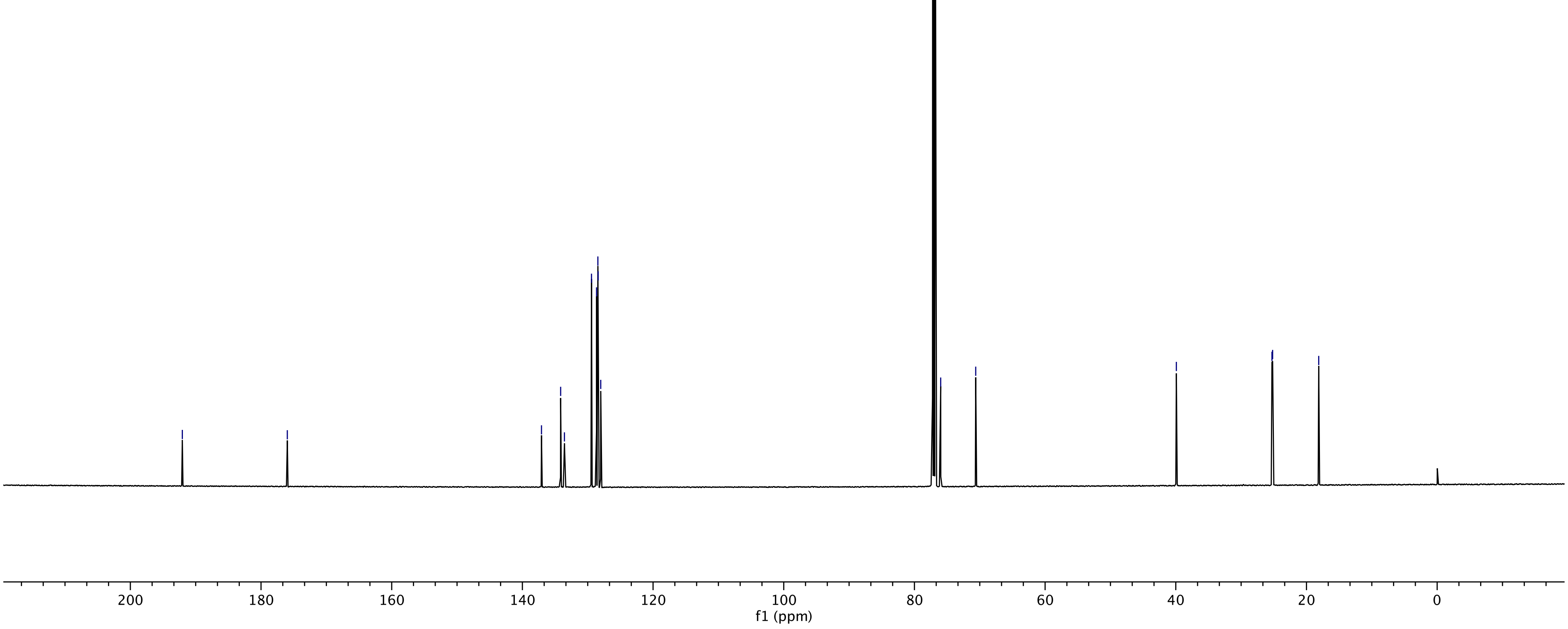

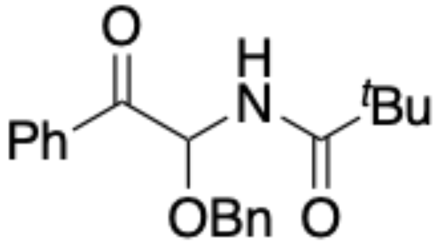

3ap for 1H NMR
(600 MHz, Chloroform-d)

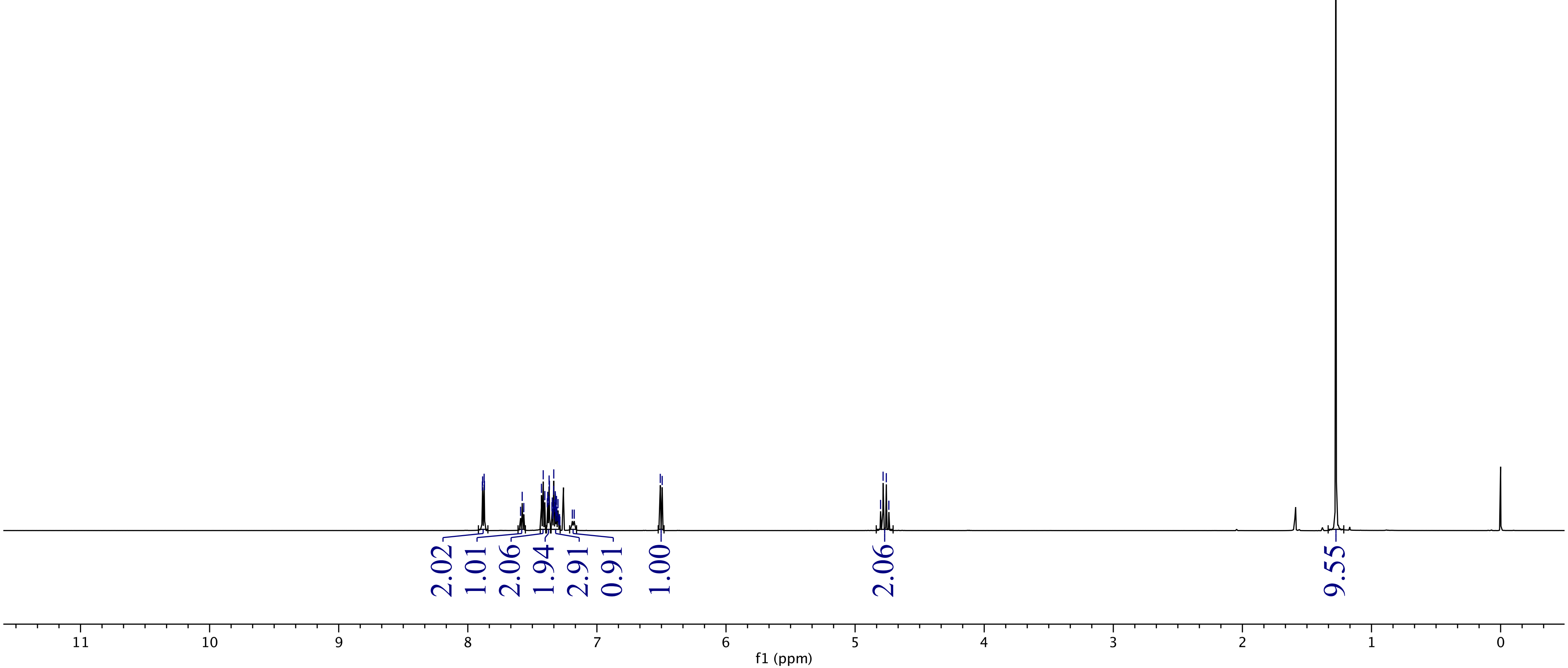

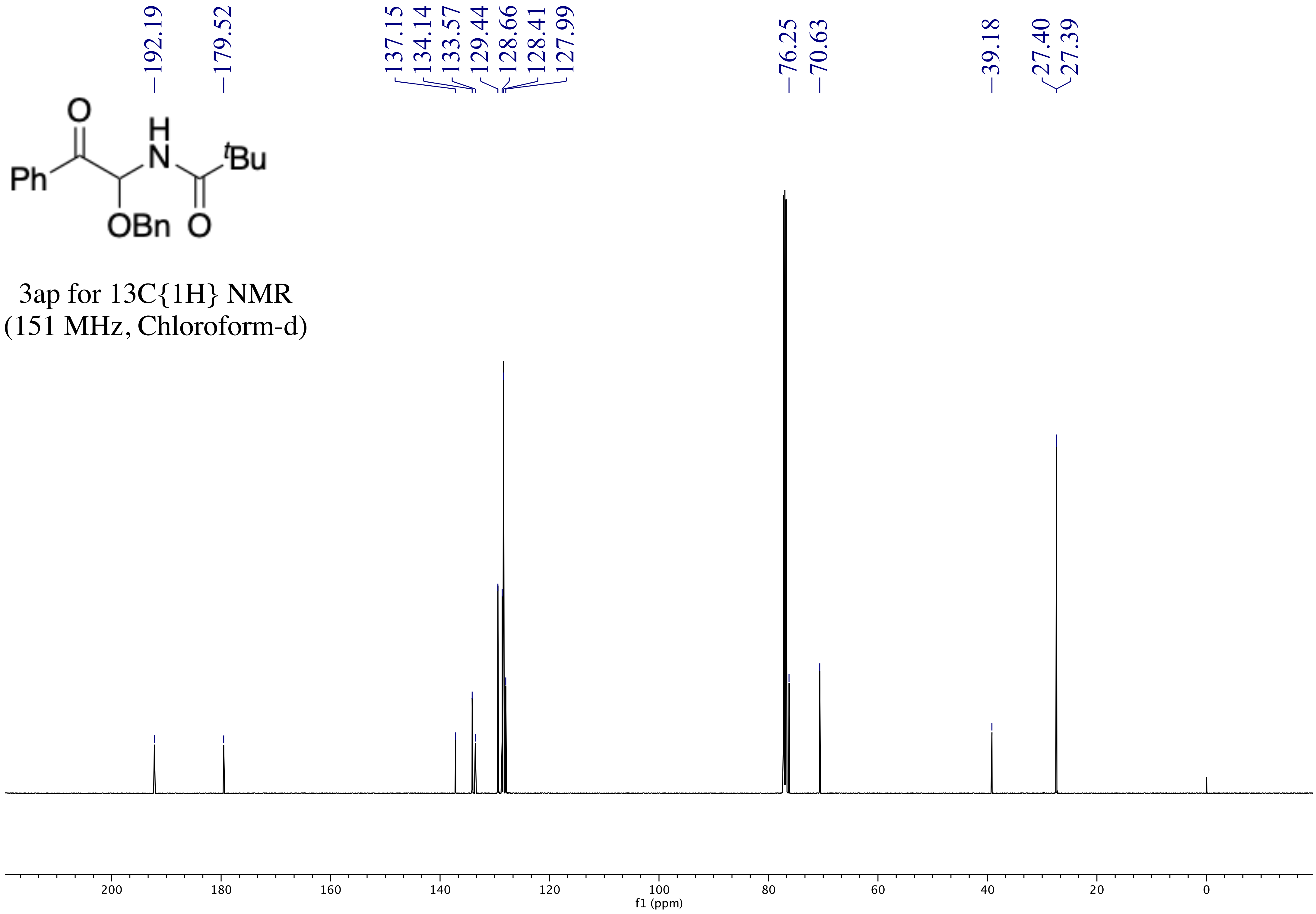
192.19
179.52
137.15
134.14
133.57
129.44
128.66
128.41
127.99
76.25
70.63
39.18
27.40
27.39
O
H
N
Ph
tBu
OBn
O
3ap for 13C{1H} NMR
(151 MHz, Chloroform-d)
200
180
160
140
120
100
80
60
40
20
0
f1 (ppm)

3aq for 1H NMR
(600 MHz, Chloroform-d)

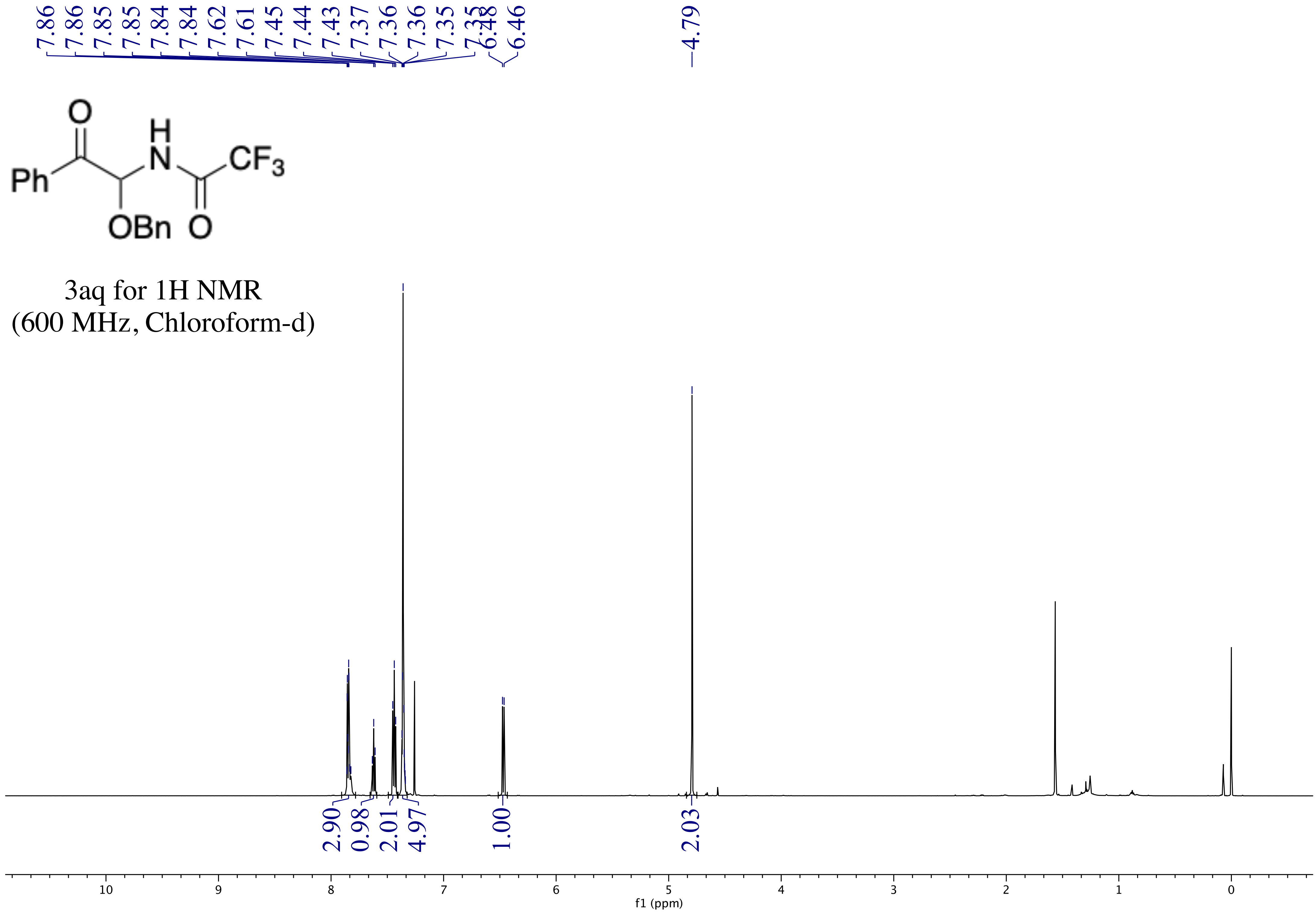

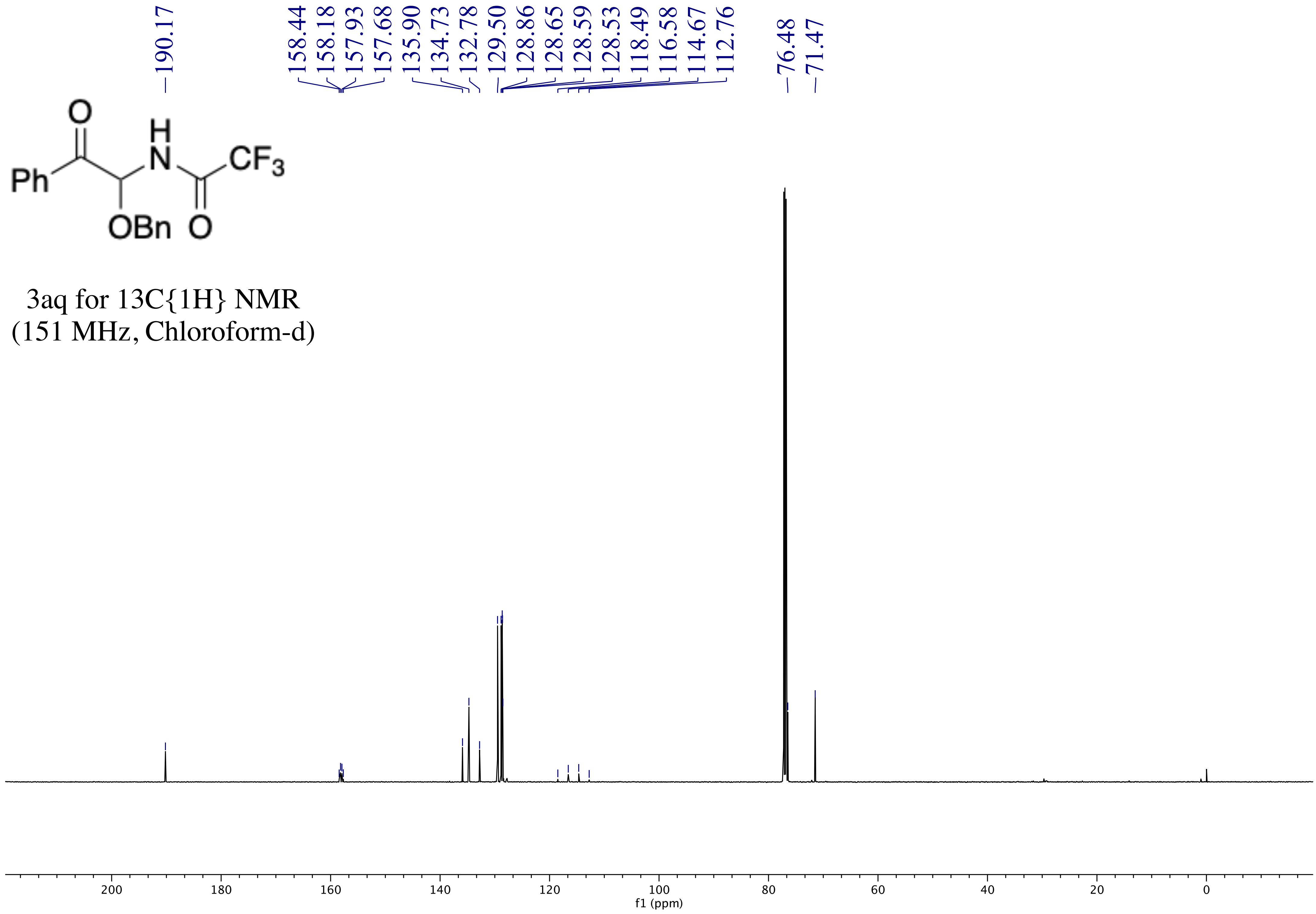

3aq for 13C{1H} NMR
(151 MHz, Chloroform-d)

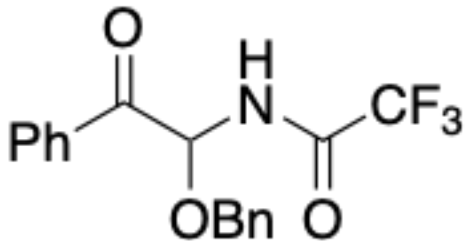

3aq for 19F NMR
(376 MHz, Chloroform-d)

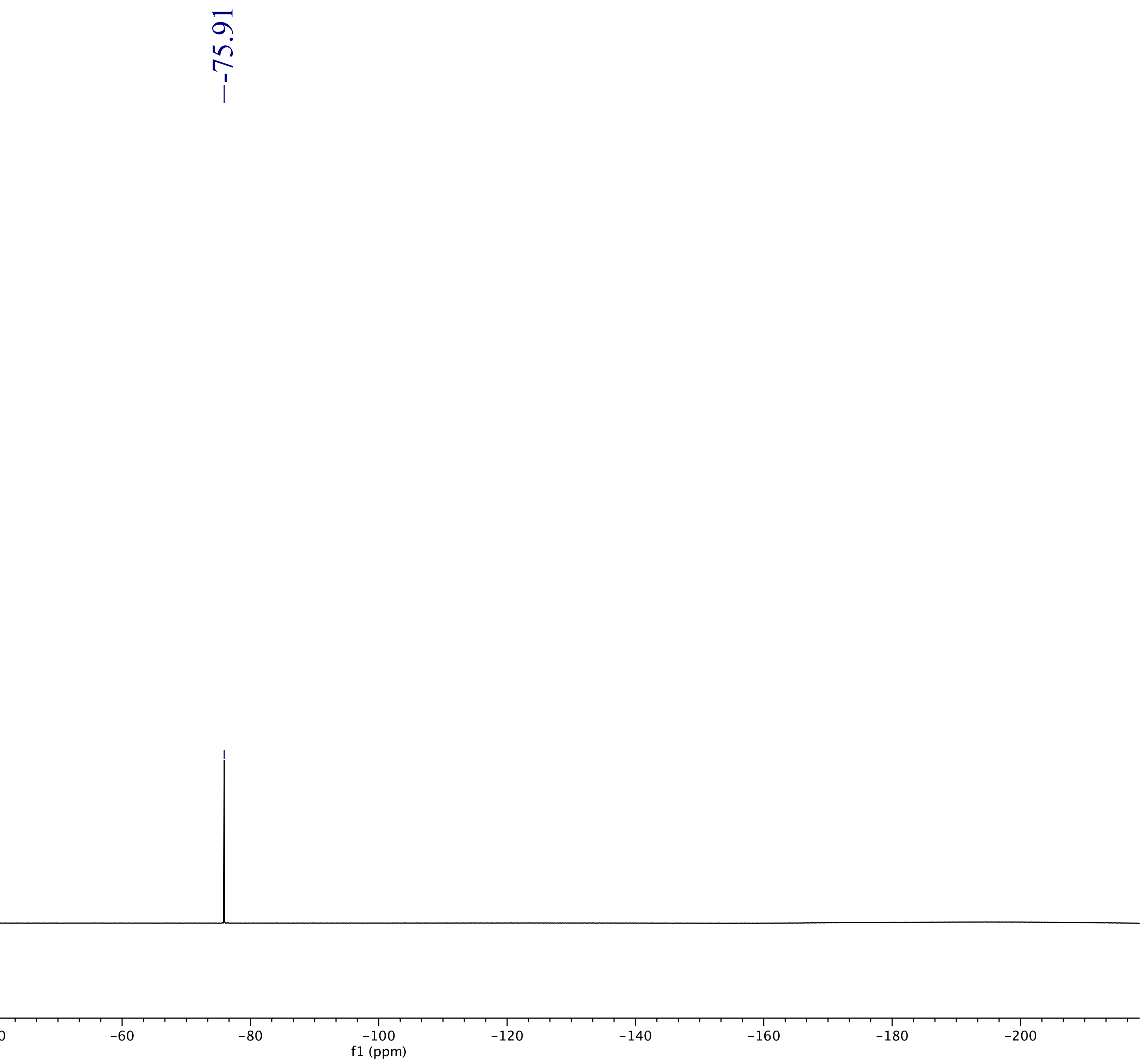

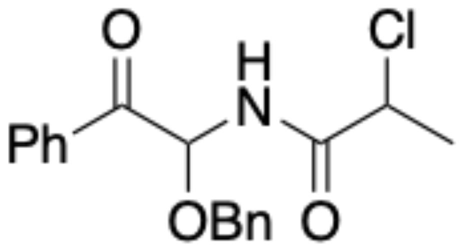

3ar for 1H NMR
(600 MHz, Chloroform-d)

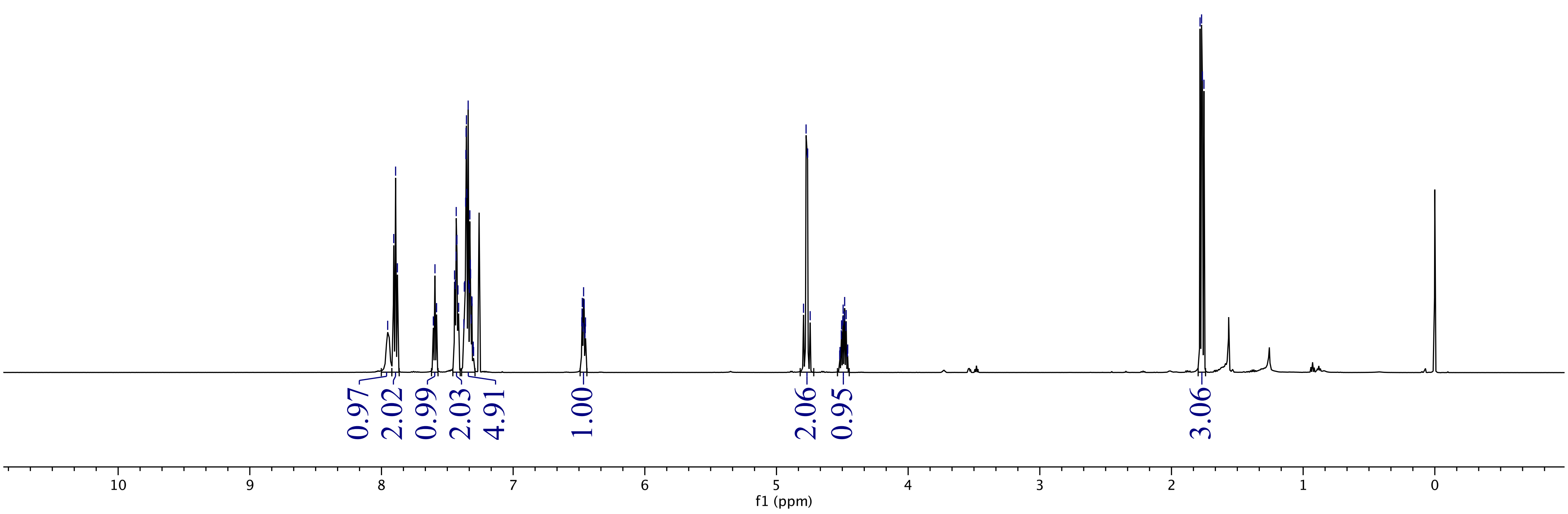

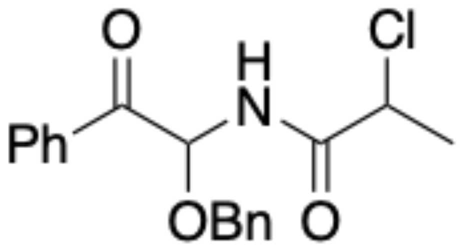

3ar for 13C{1H} NMR
(151 MHz, Chloroform-d)

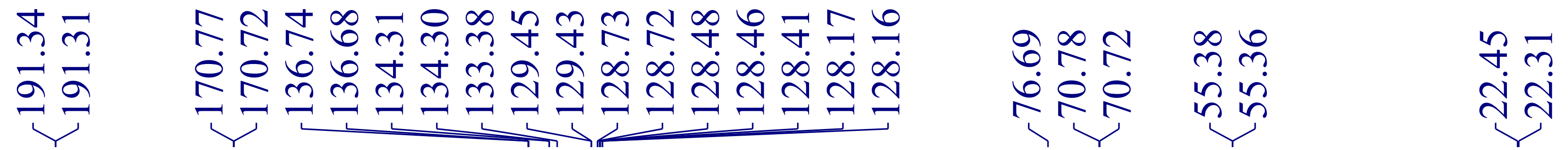

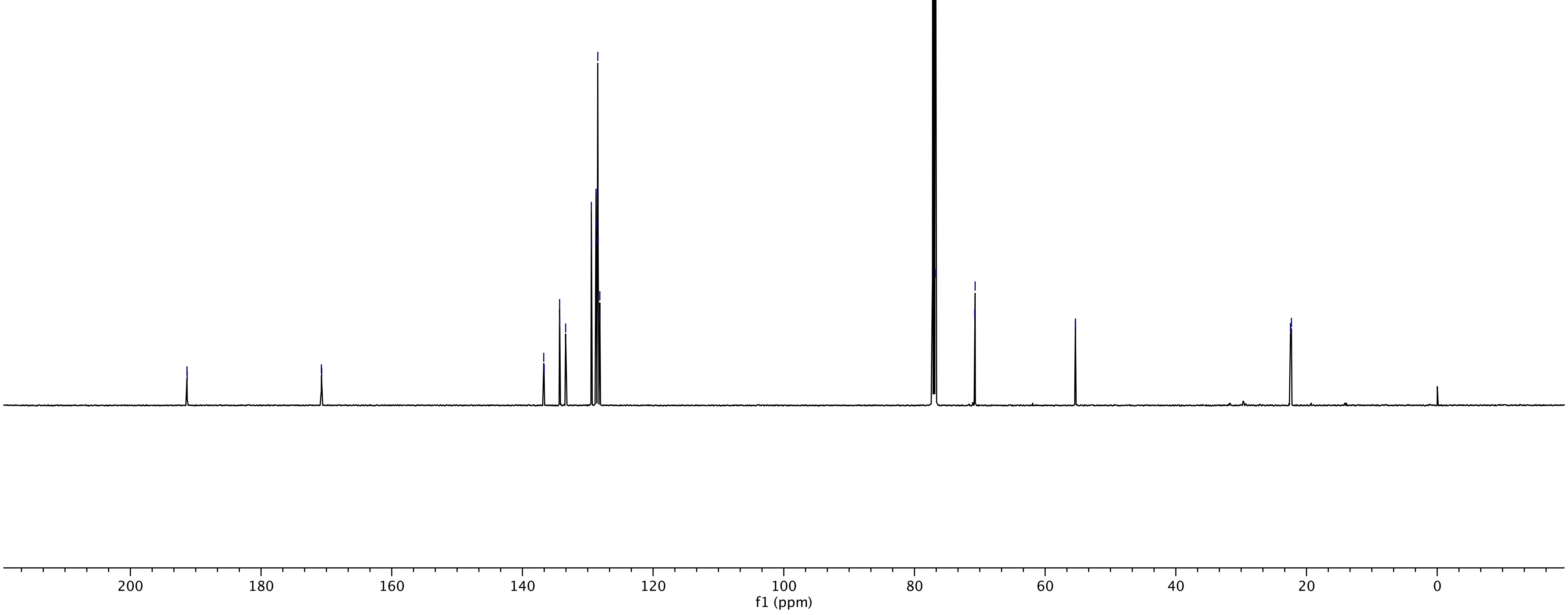

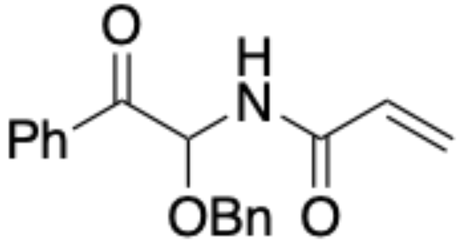

3as for 1H NMR
(600 MHz, Chloroform-d)

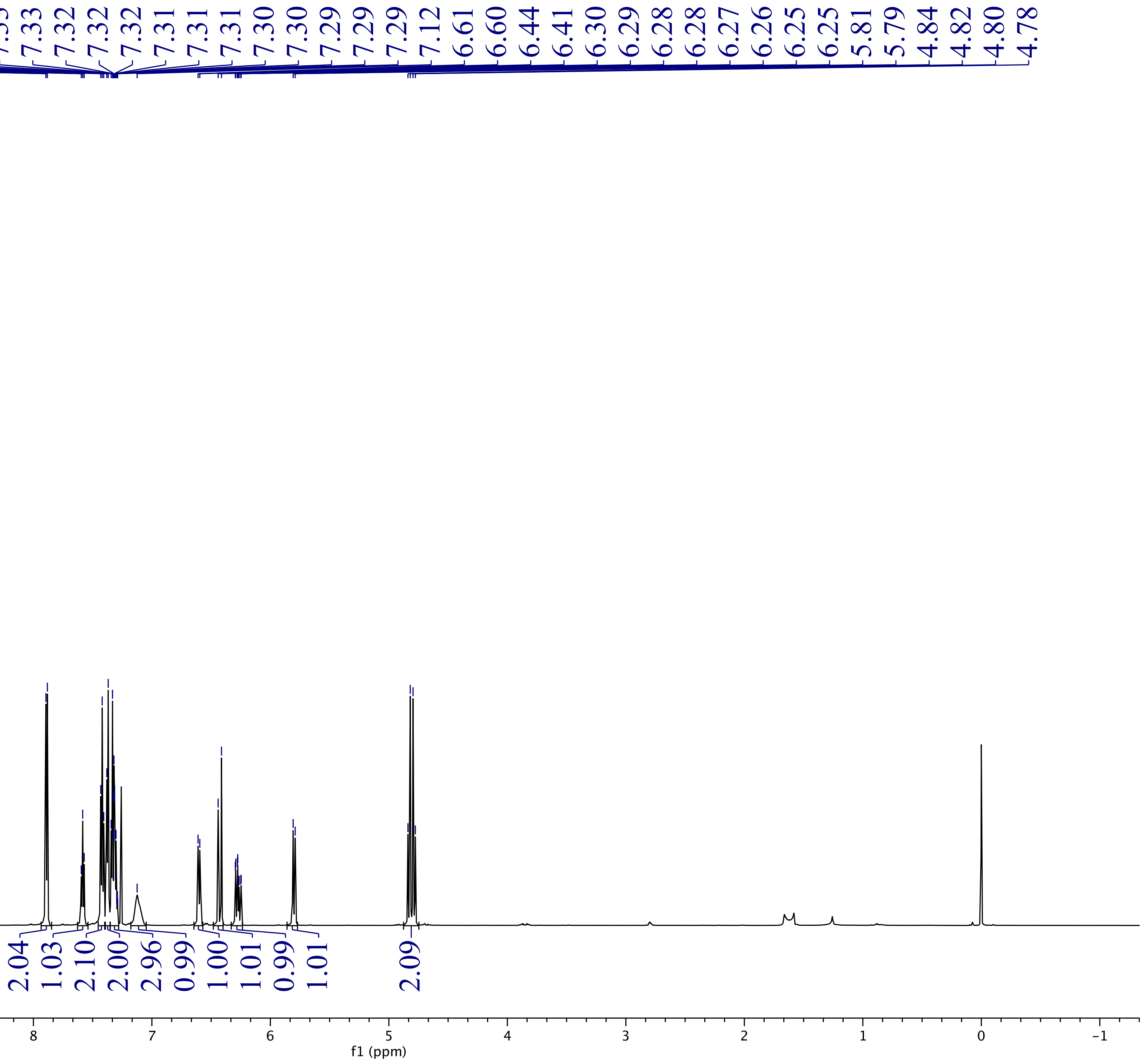

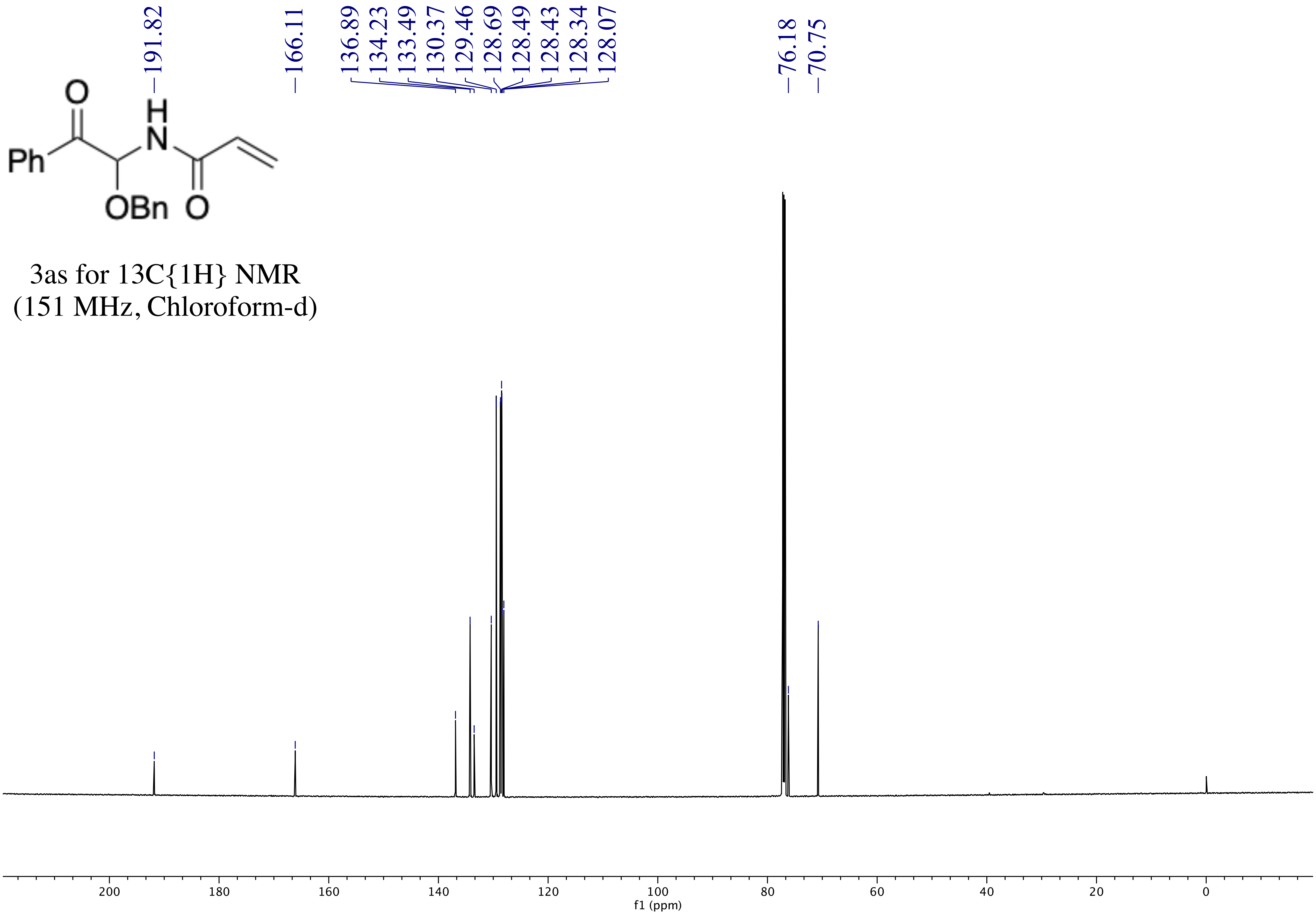

3as for 13C{1H} NMR
(151 MHz, Chloroform-d)

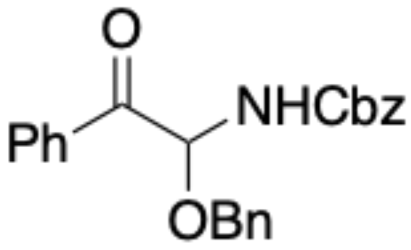

3at for 1H NMR
(600 MHz, Chloroform-d)

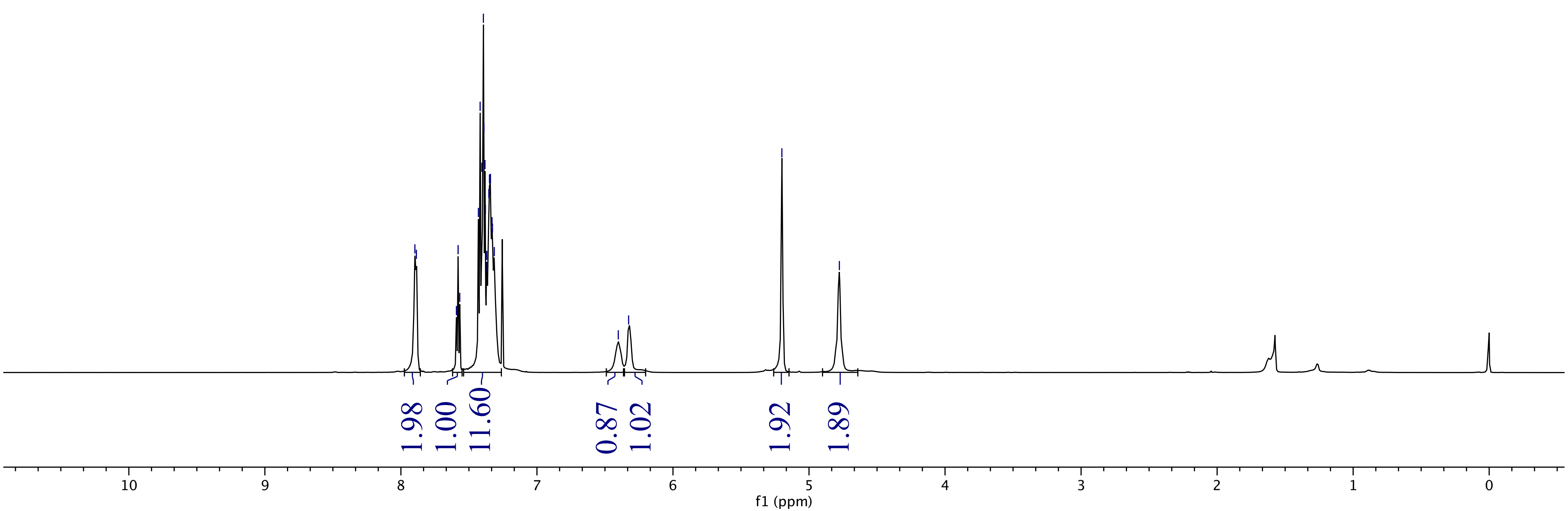

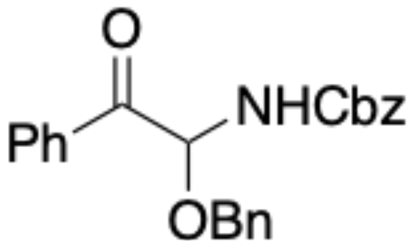

3at for 13C{1H} NMR
(151 MHz, Chloroform-d)

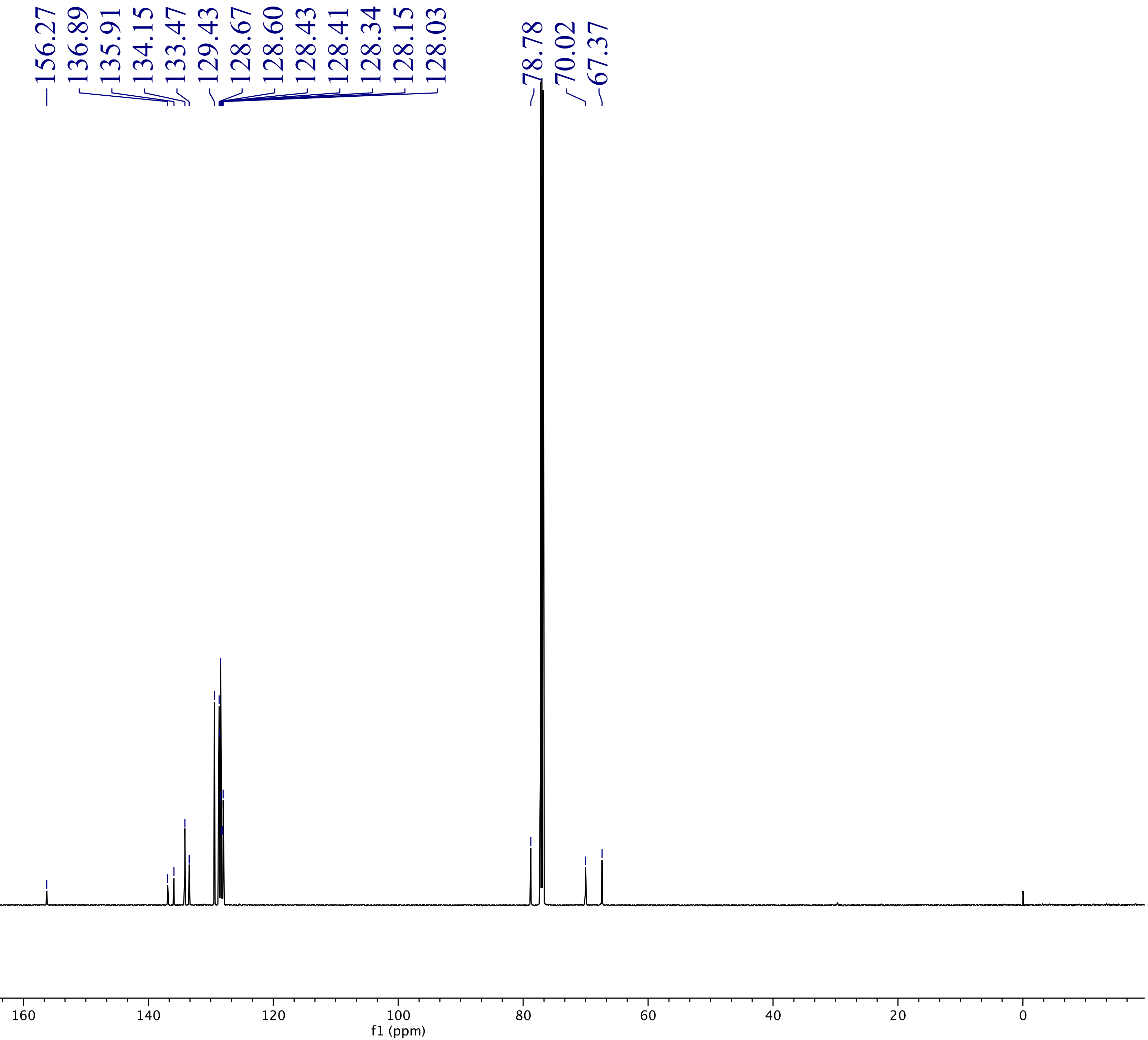

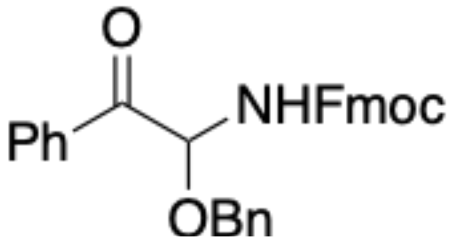

3au for 1H NMR
(600 MHz, Chloroform-d)

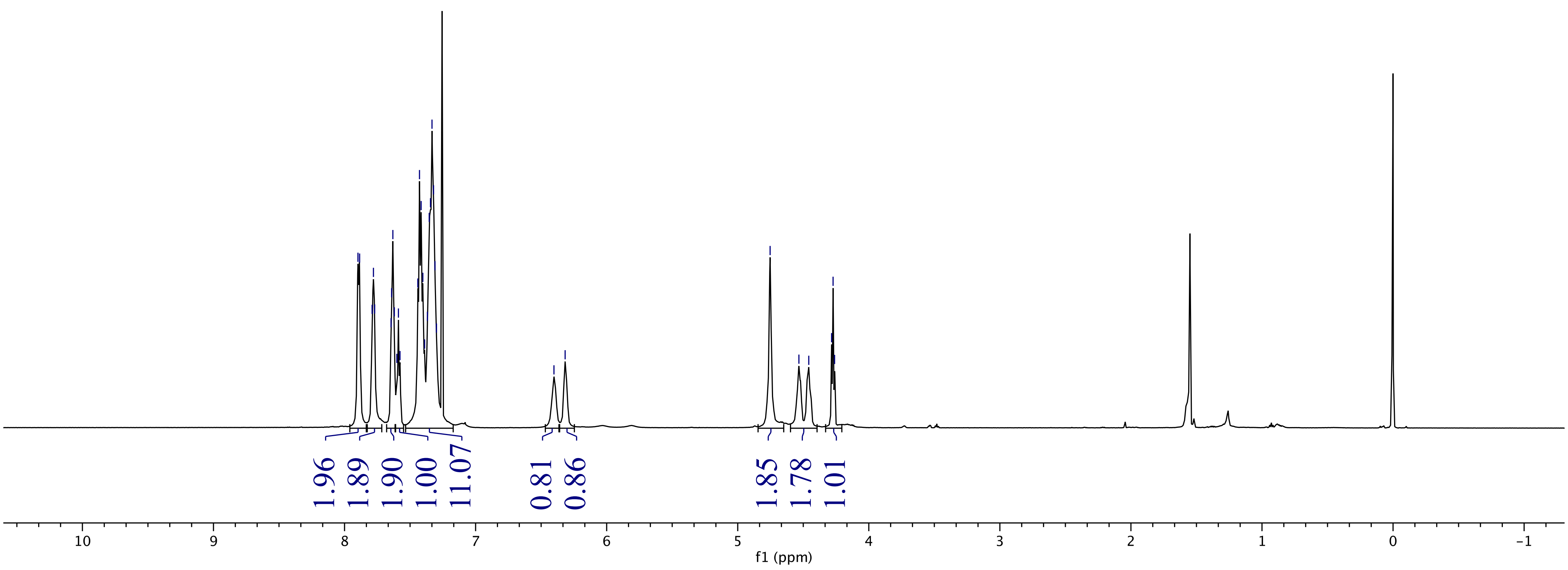

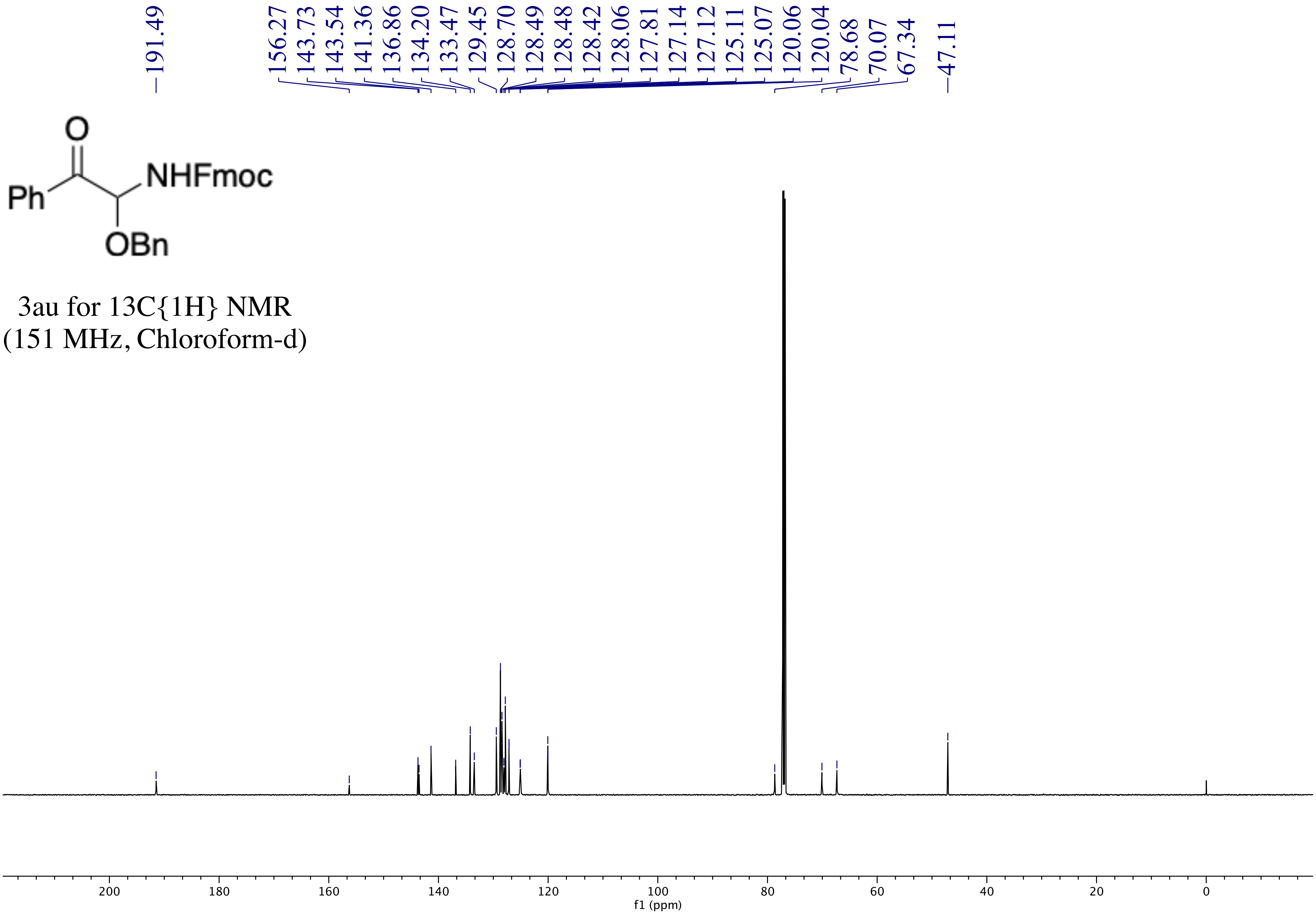

3au for 13C{1H} NMR
(151 MHz, Chloroform-d)

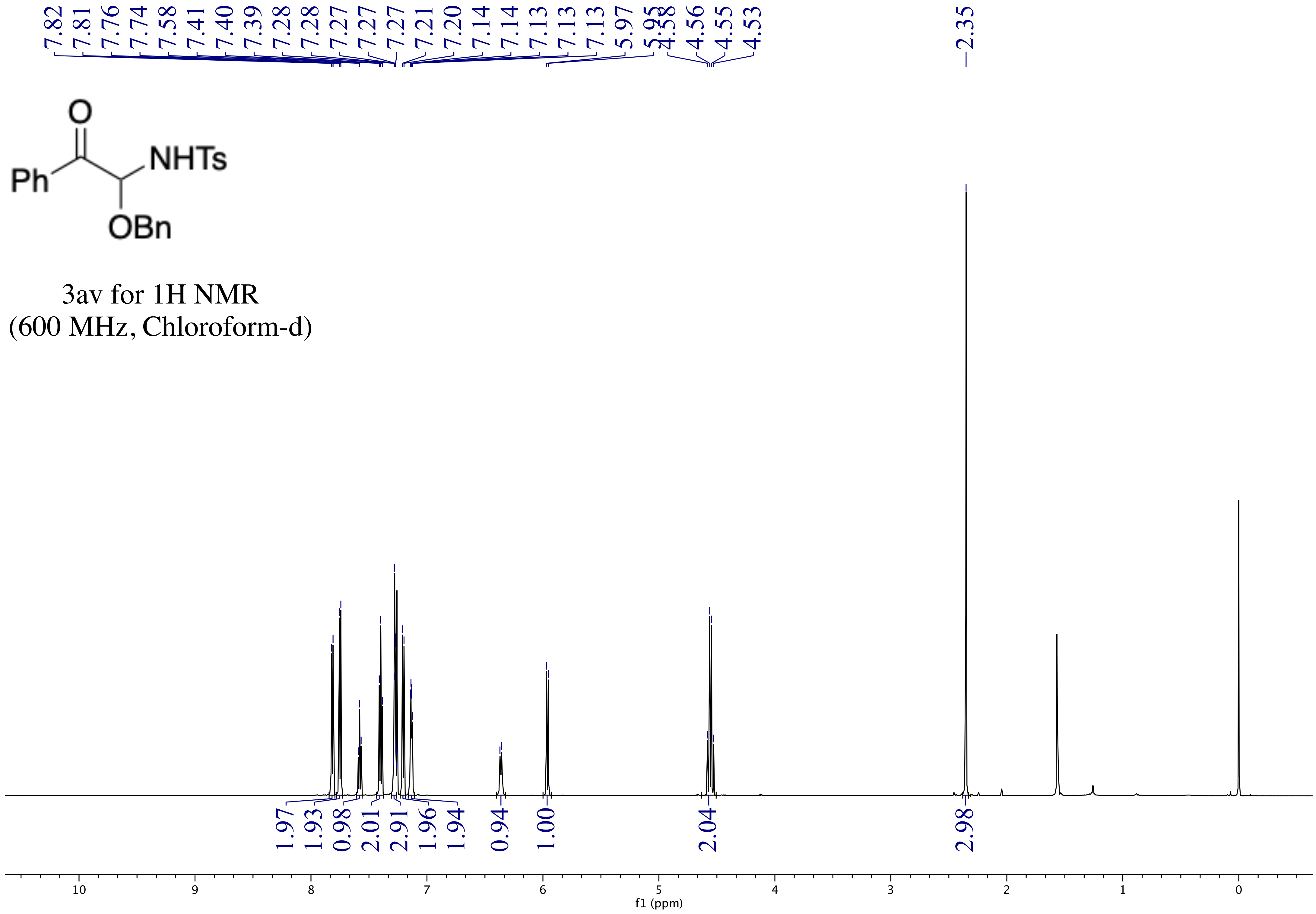
7.82
7.81
7.76
7.74
7.58
7.41
7.40
7.39
7.28
7.28
7.27
7.27
7.27
7.21
7.20
7.14
7.14
7.13
7.13
7.13
5.97
5.95
4.58
4.56
4.55
4.53
2.35
O
NHTs
Ph
OBn
3av for 1H NMR
(600 MHz, Chloroform-d)
1.97
1.93
0.98
2.01
2.91
1.96
1.94
0.94
1.00
2.04
2.98
10
9
8
7
6
5
4
3
2
1
0
f1 (ppm)

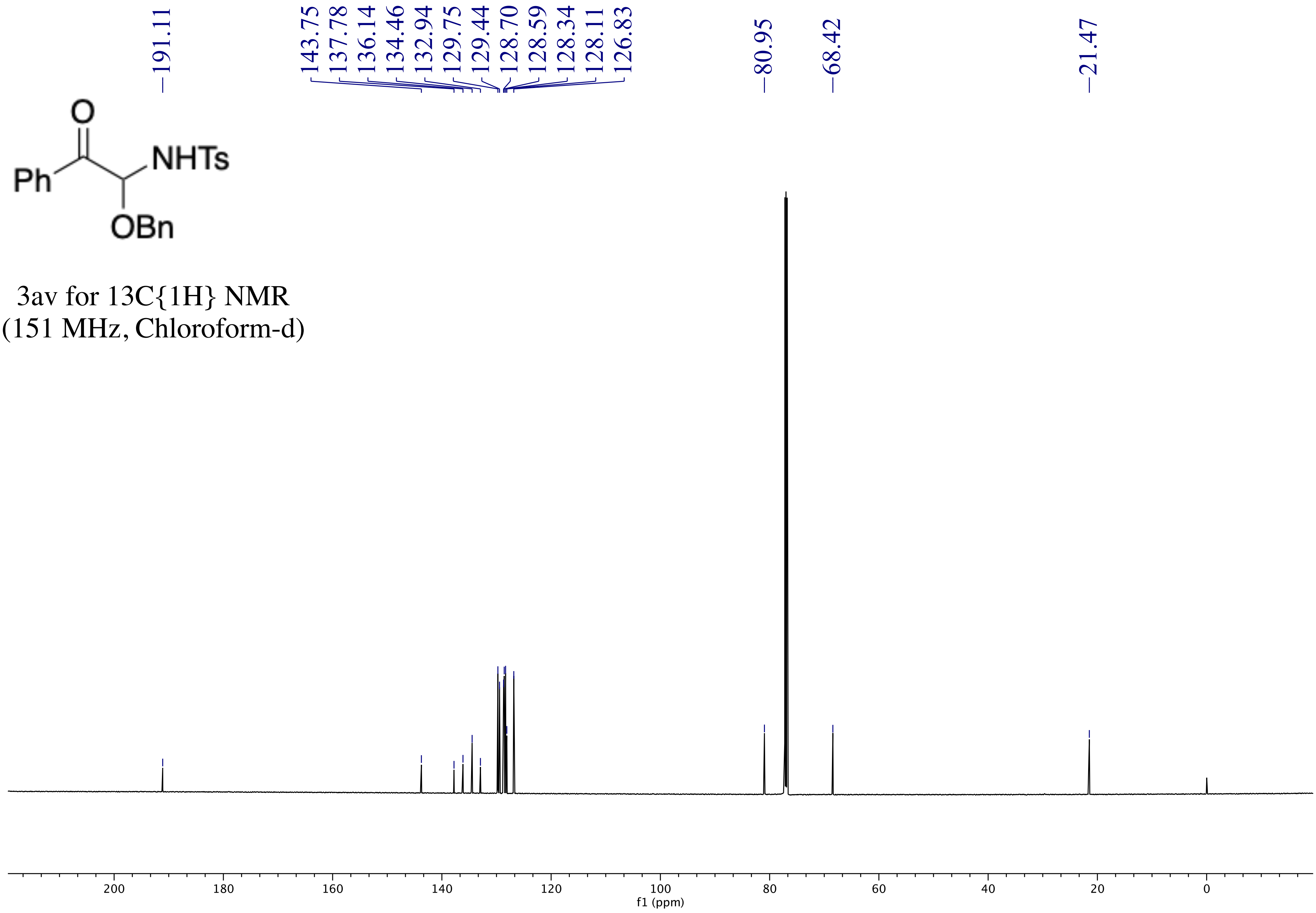

3av for 13C{1H} NMR
(151 MHz, Chloroform-d)

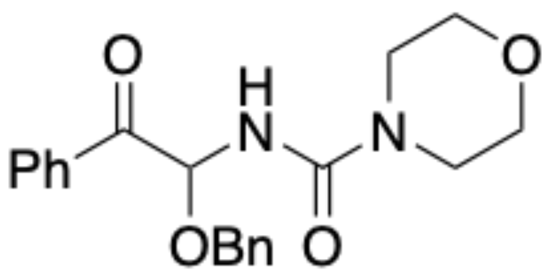

3aw for 1H NMR
(600 MHz, Chloroform-d)

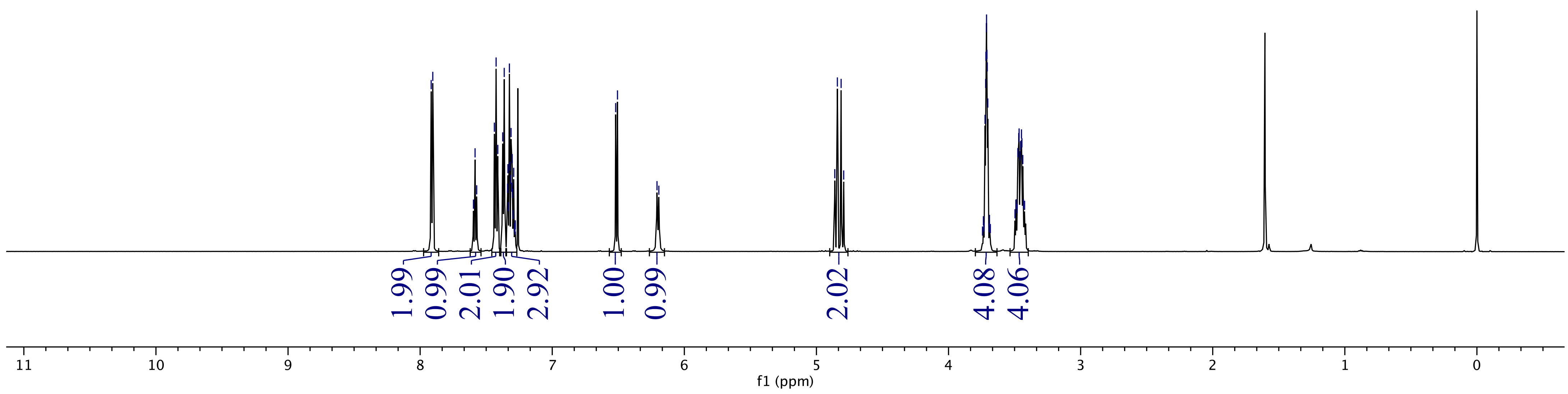

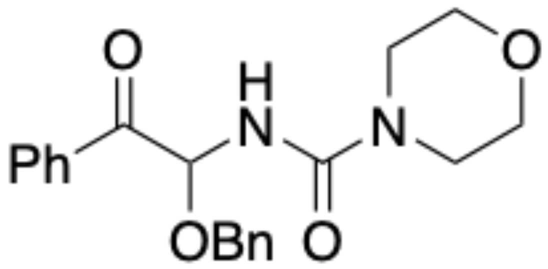

3aw for 13C{1H} NMR
(151 MHz, Chloroform-d)

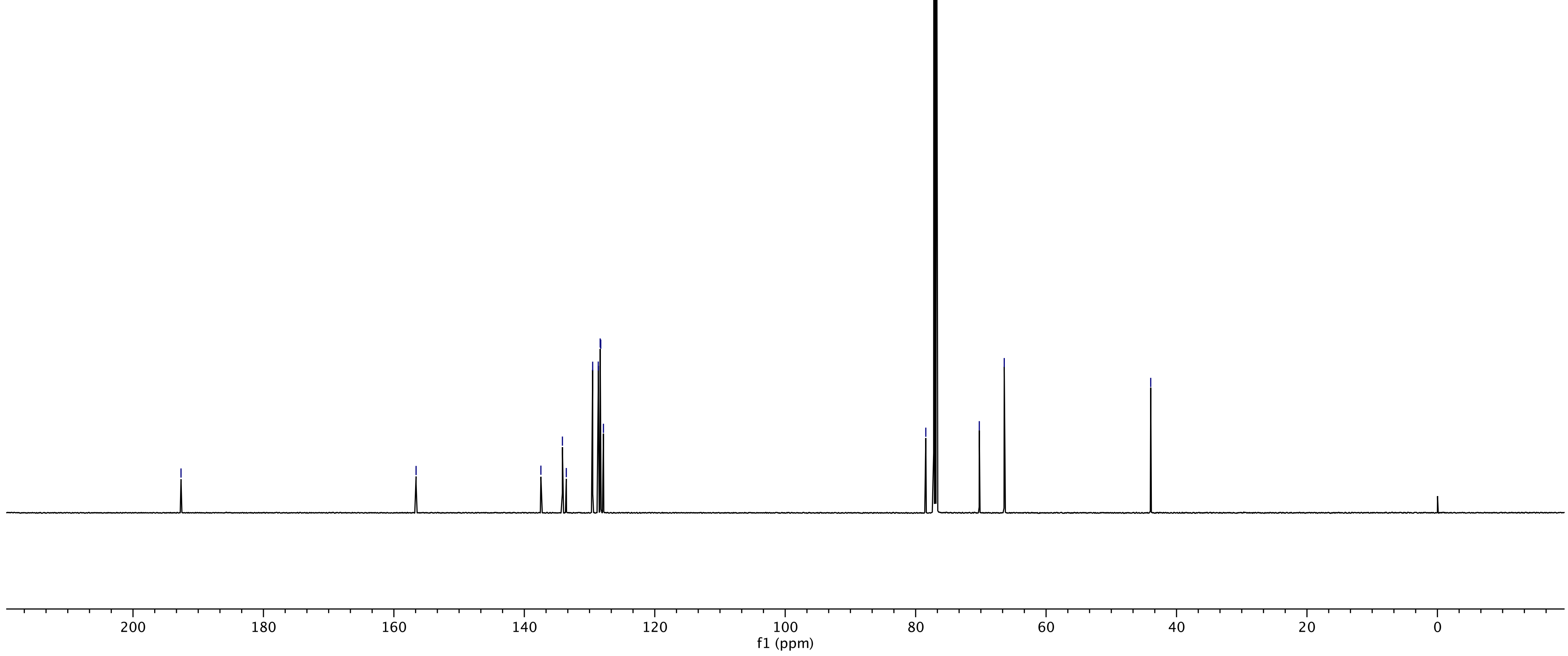

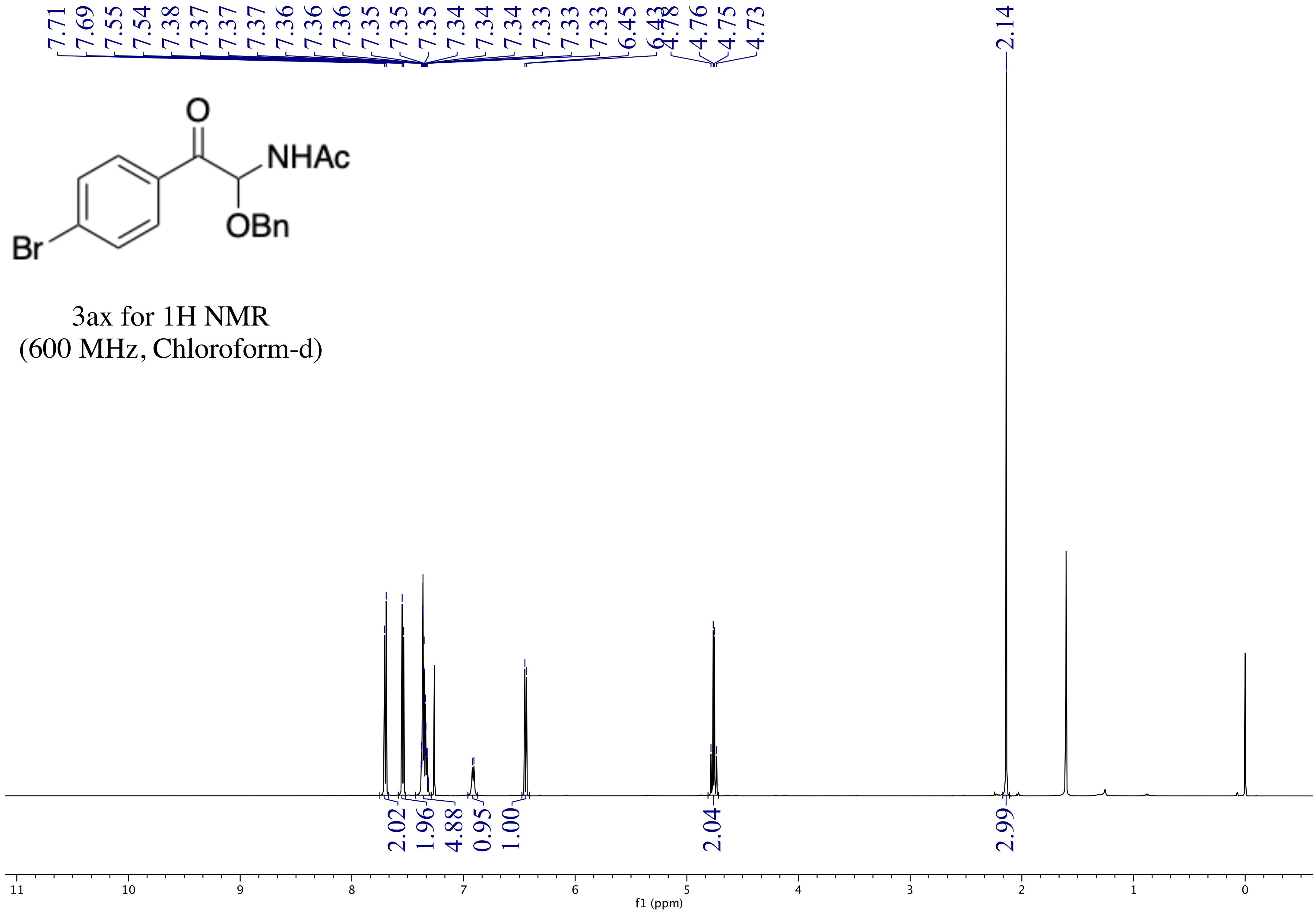
7.71
7.69
7.55
7.54
7.38
7.37
7.37
7.37
7.36
7.36
7.36
7.35
7.35
7.35
7.34
7.34
7.34
7.33
7.33
7.33
6.45
6.43
4.78
4.76
4.75
4.73
2.14
O
NHAc
OBn
Br
3ax for 1H NMR
(600 MHz, Chloroform-d)
2.02
1.96
4.88
0.95
1.00
2.04
2.99
11
10
9
8
7
6
5
4
3
2
1
0
f1 (ppm)

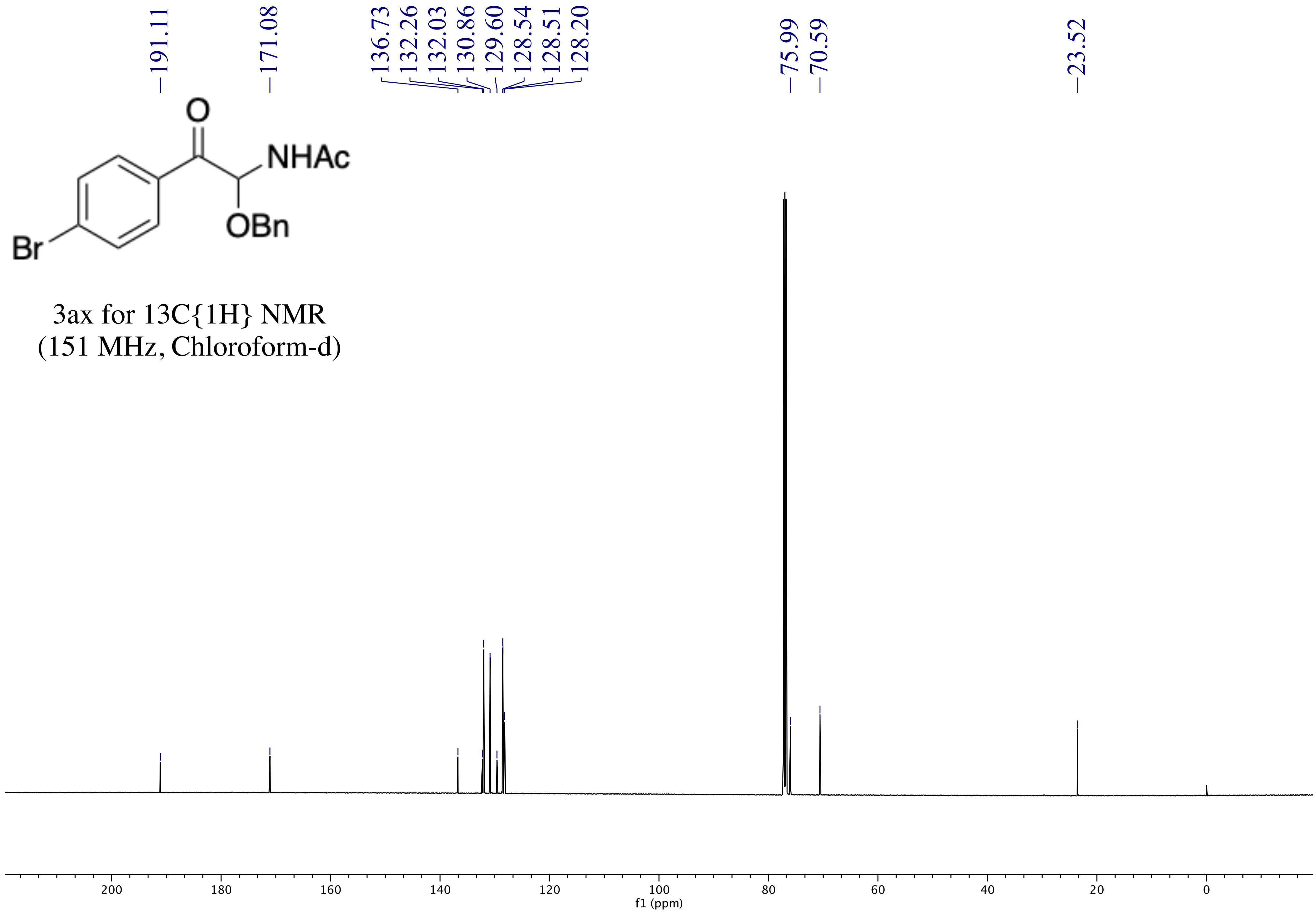

191.11
171.08
136.73
132.26
132.03
130.86
129.60
128.54
128.51
128.20
75.99
70.59
23.52
O
NHAc
OBn
Br
3ax for 13C{1H} NMR
(151 MHz, Chloroform-d)
200
180
160
140
120
100
80
60
40
20
0
f1 (ppm)

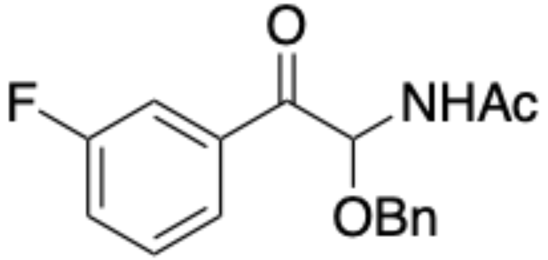

3ay for 1H NMR
(600 MHz, Chloroform-d)

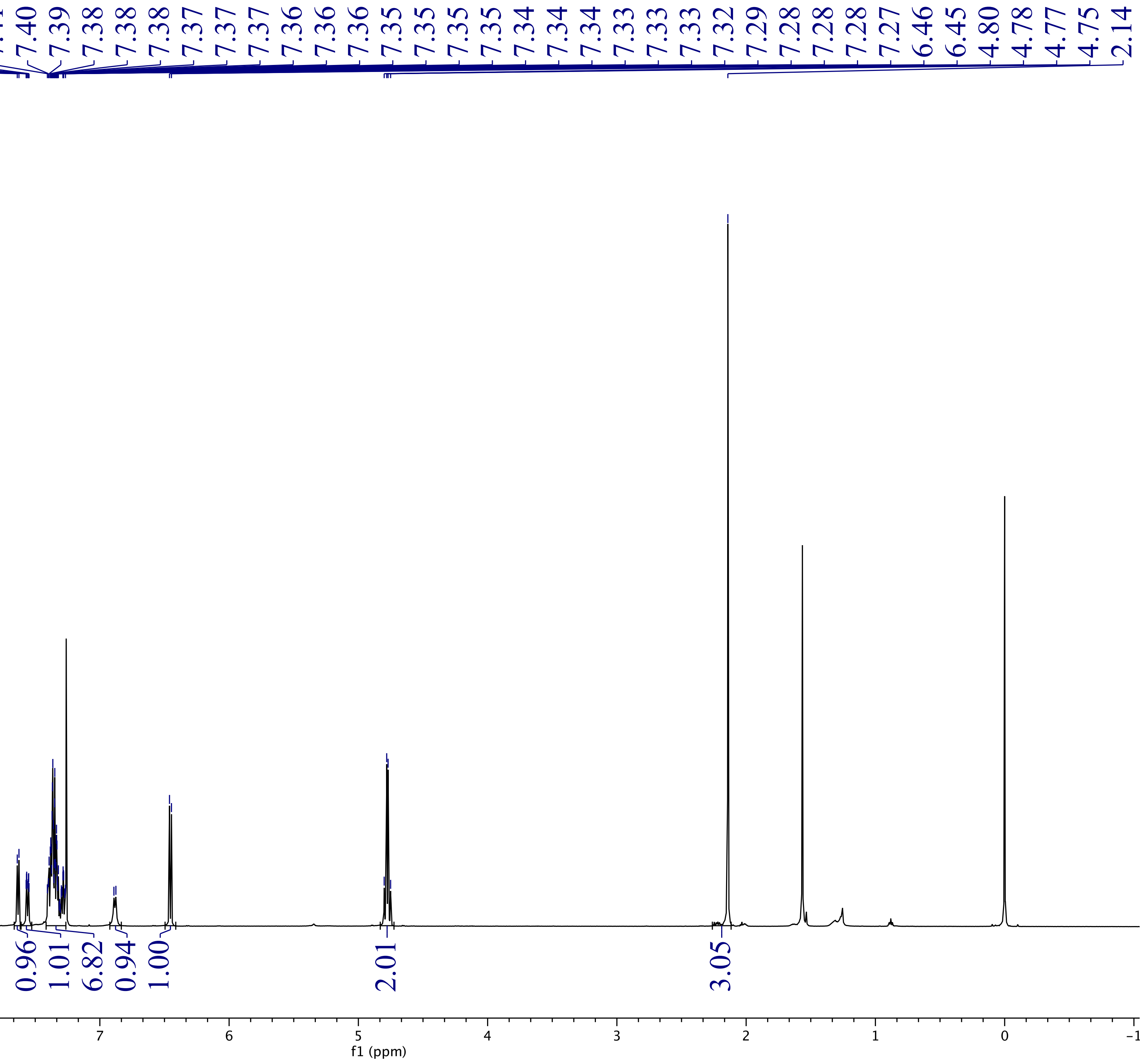

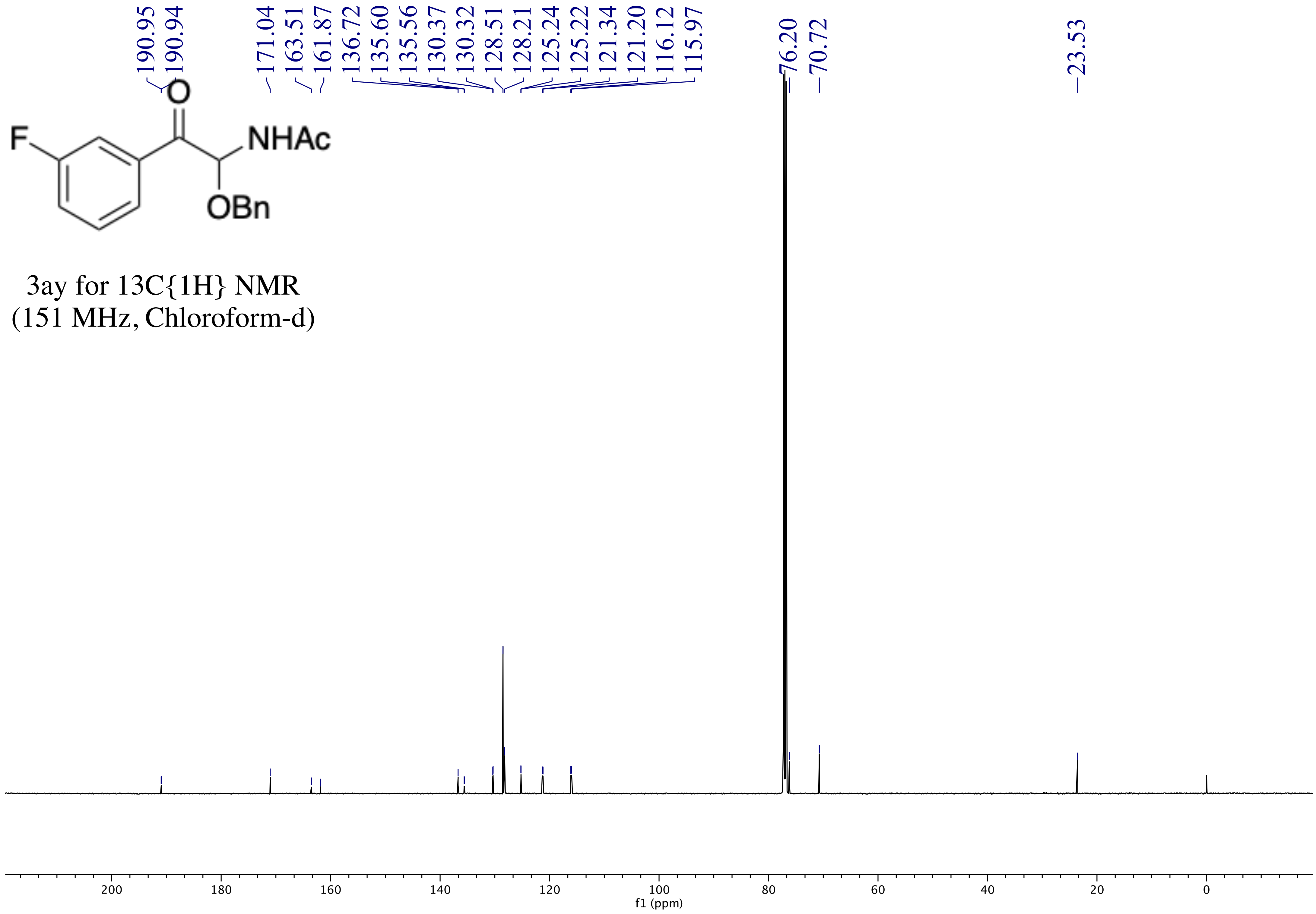
190.95
190.94
171.04
163.51
161.87
136.72
135.60
135.56
130.37
130.32
128.51
128.21
125.24
125.22
121.34
121.20
116.12
115.97
76.20
70.72
23.53
F
O
NHAc
OBn
3ay for 13C{1H} NMR
(151 MHz, Chloroform-d)
200
180
160
140
120
100
80
60
40
20
0
f1 (ppm)

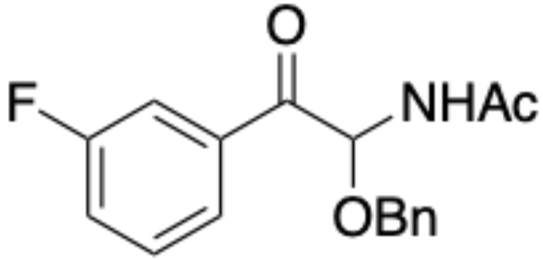

3ay for 19F NMR
(376 MHz, Chloroform-d)

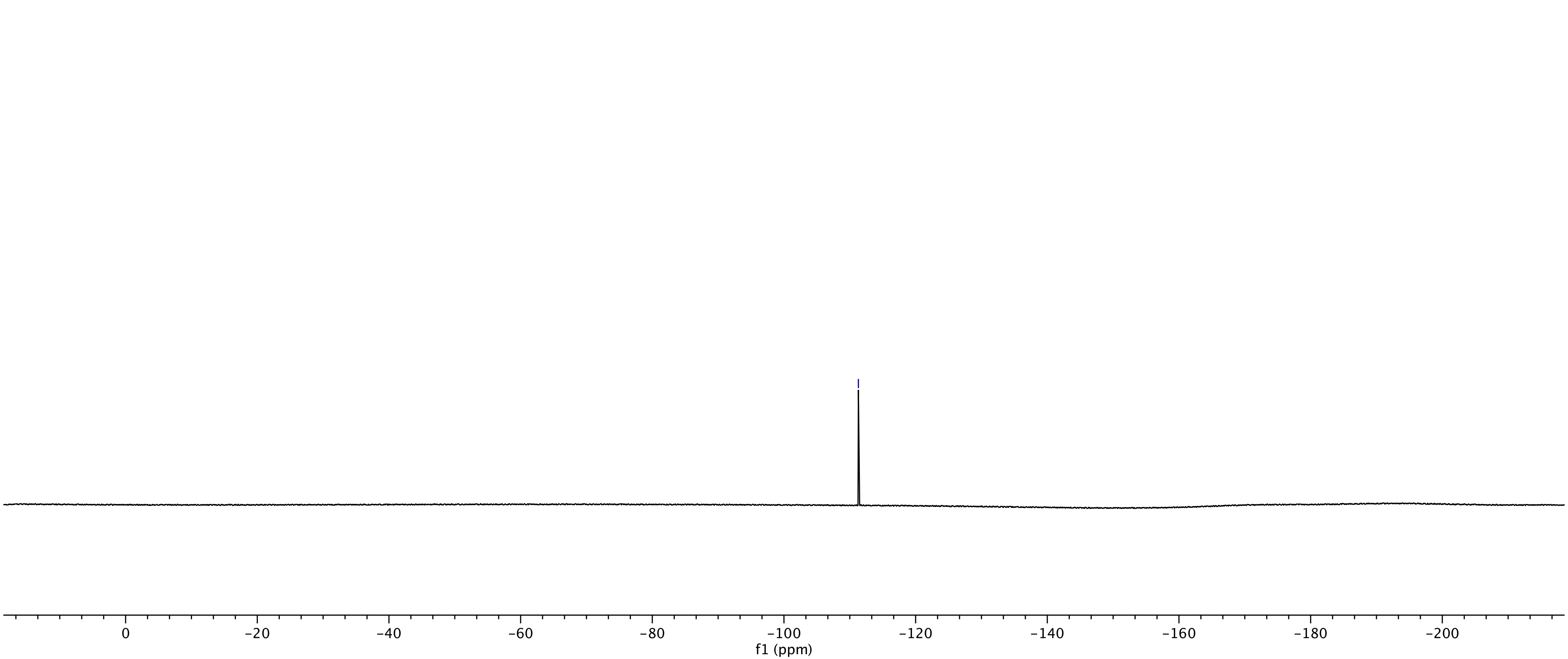

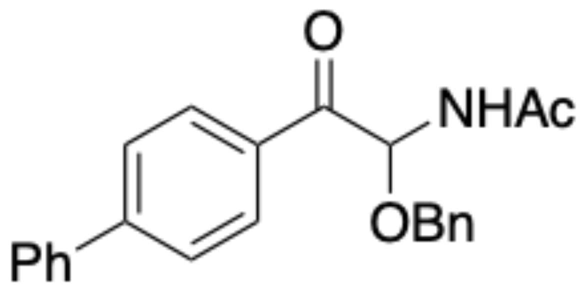

3az for 1H NMR
(600 MHz, Chloroform-d)

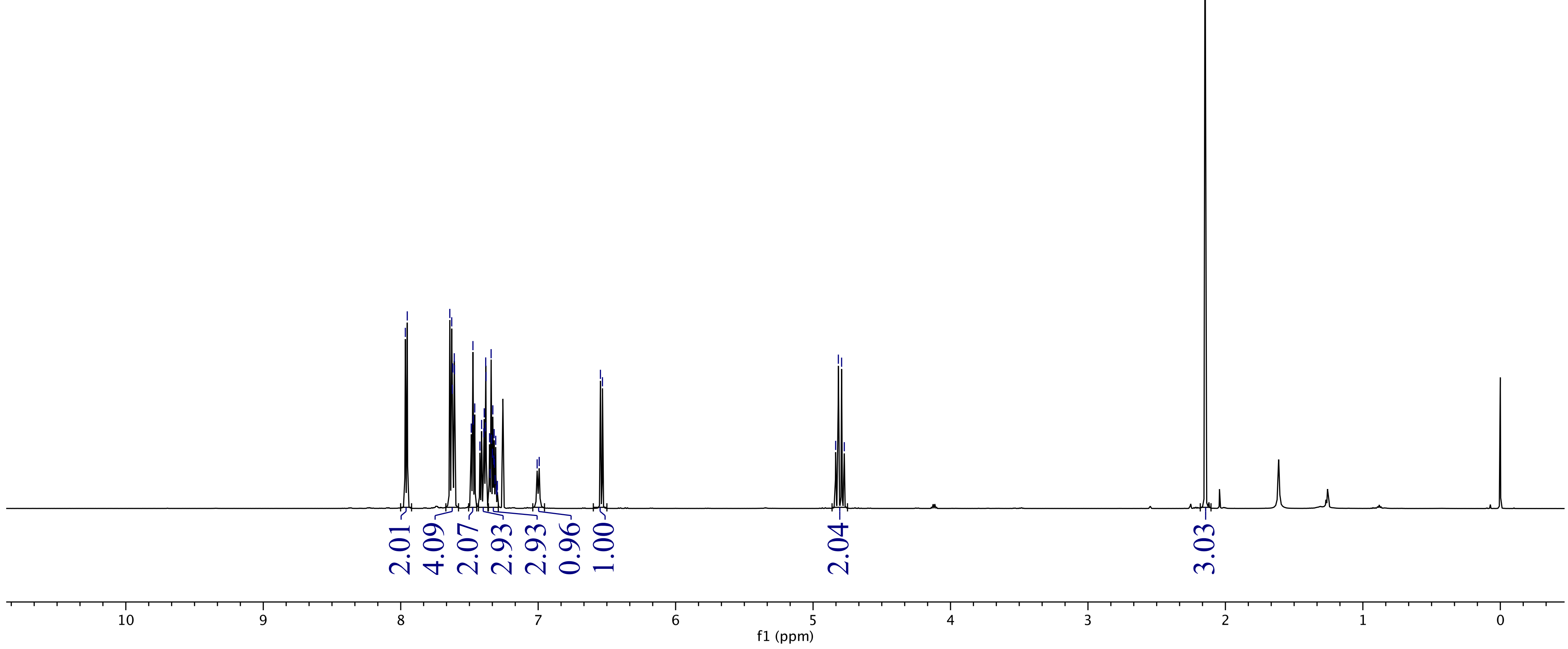

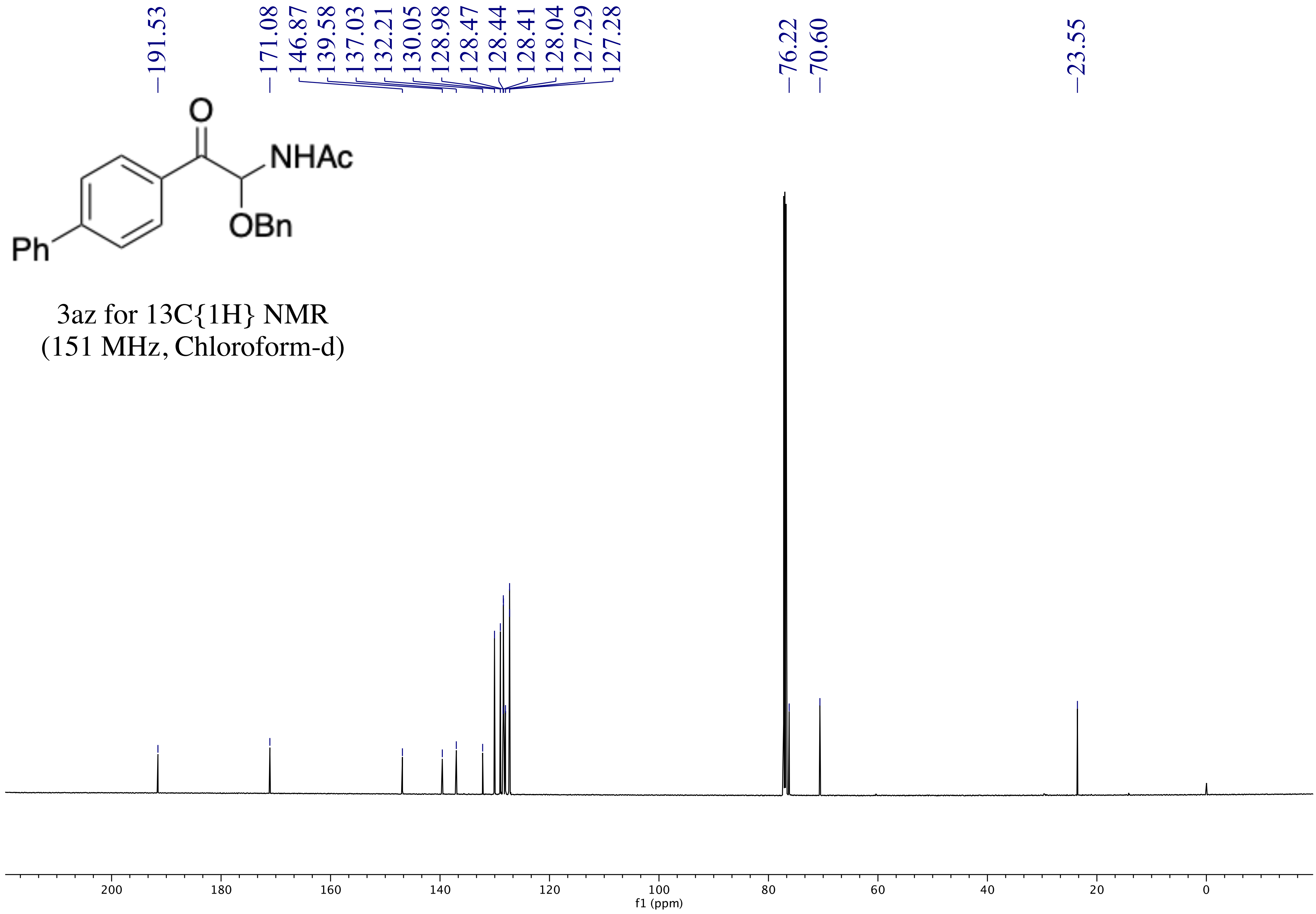

191.53
171.08
146.87
139.58
137.03
132.21
130.05
128.98
128.47
128.44
128.41
128.04
127.29
127.28
76.22
70.60
23.55
NHAc
OBn
Ph
O
3az for 13C{1H} NMR
(151 MHz, Chloroform-d)
200
180
160
140
120
100
80
60
40
20
0
f1 (ppm)

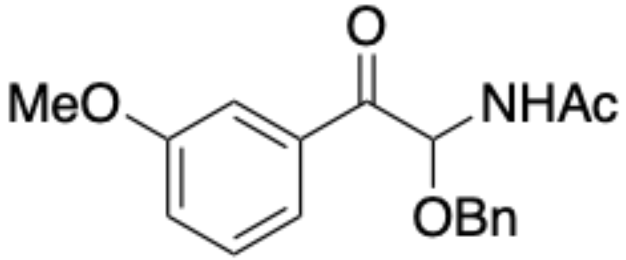

3ba for 1H NMR
(600 MHz, Chloroform-d)

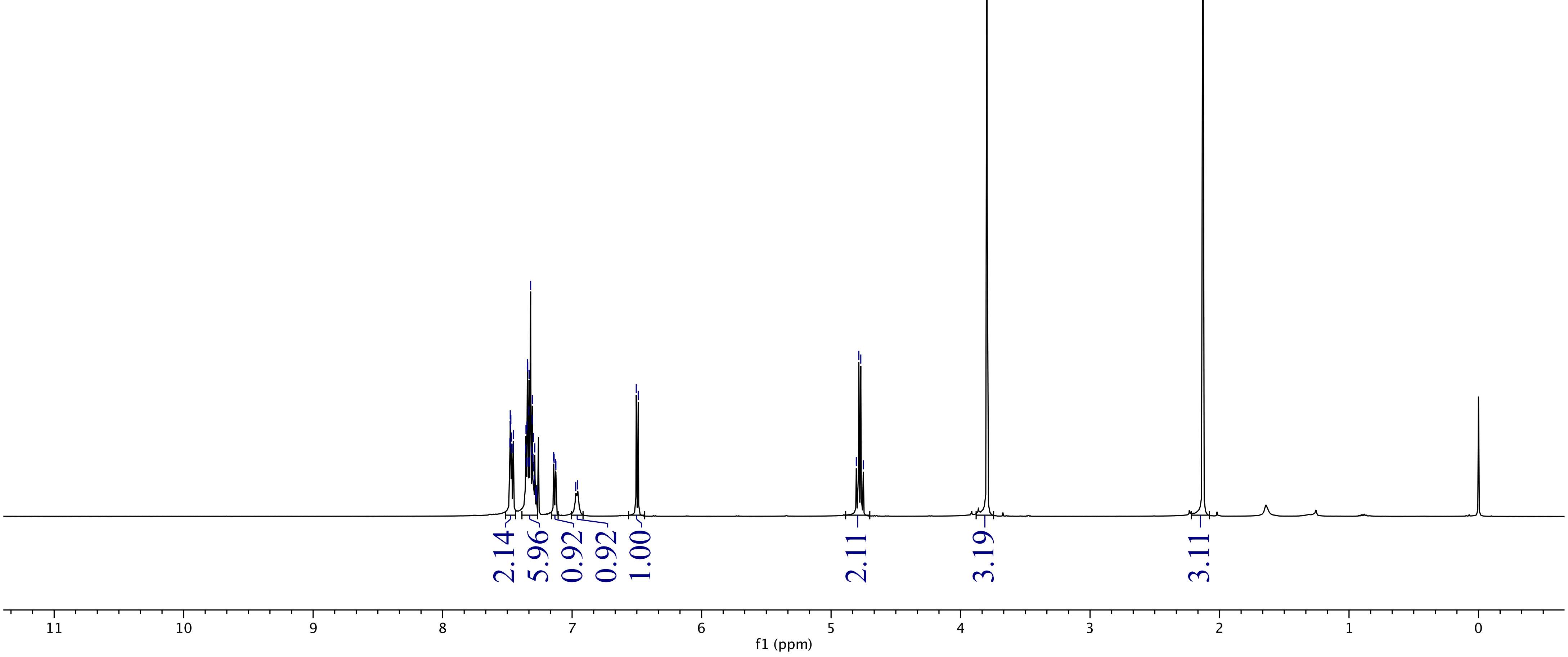

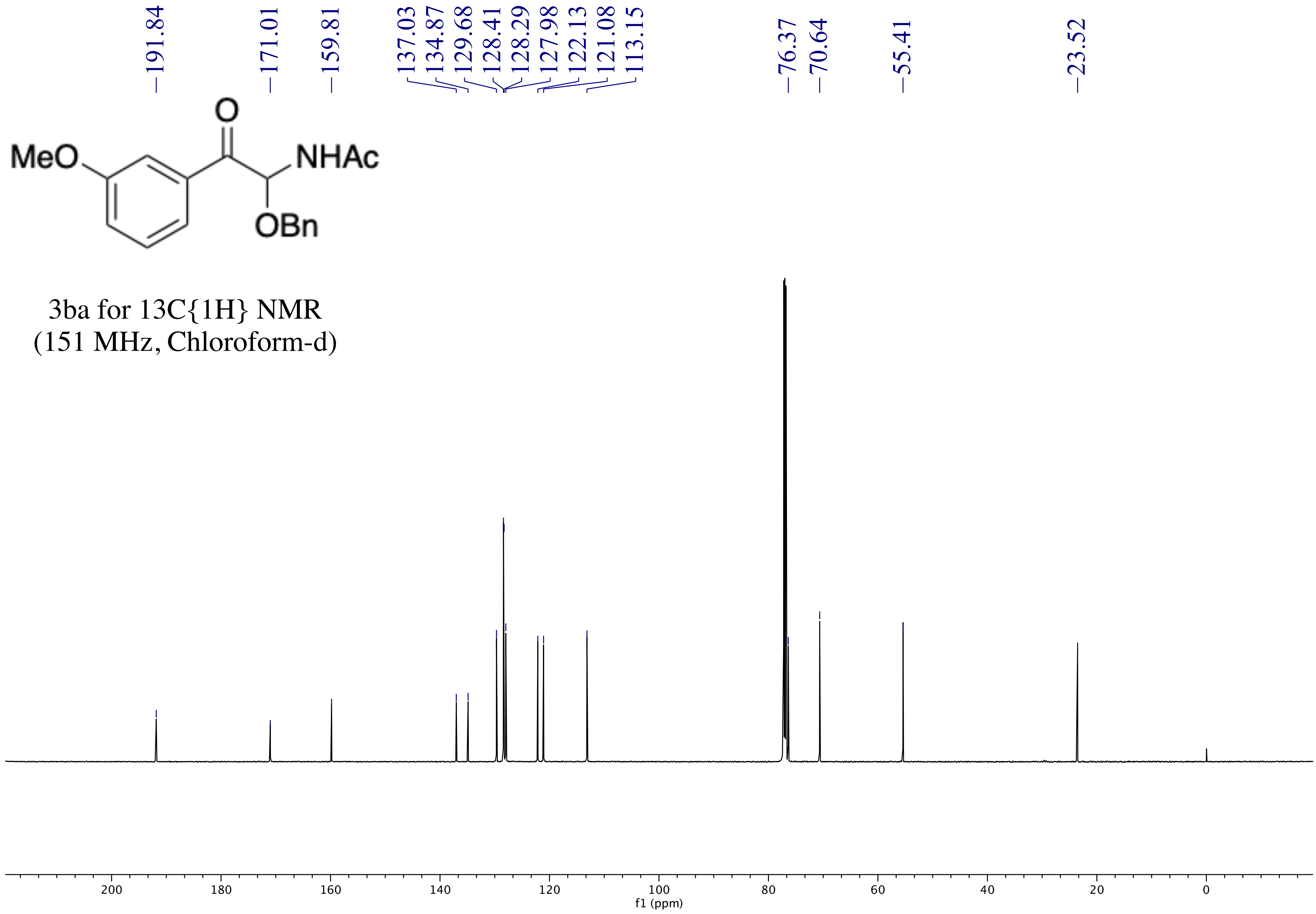

191.84
171.01
159.81
137.03
134.87
129.68
128.41
128.29
127.98
122.13
121.08
113.15
76.37
70.64
55.41
23.52
MeO
O
NHAc
OBn
3ba for 13C{1H} NMR
(151 MHz, Chloroform-d)
200
180
160
140
120
100
80
60
40
20
0
f1 (ppm)

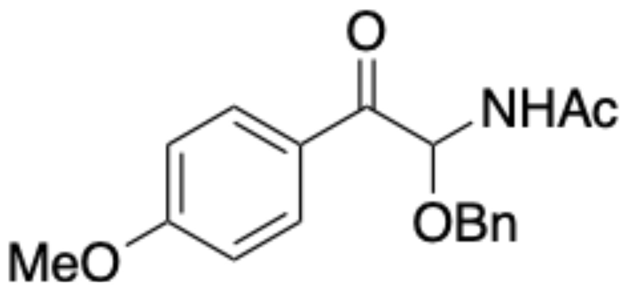

3bb for 1H NMR
(600 MHz, Chloroform-d)

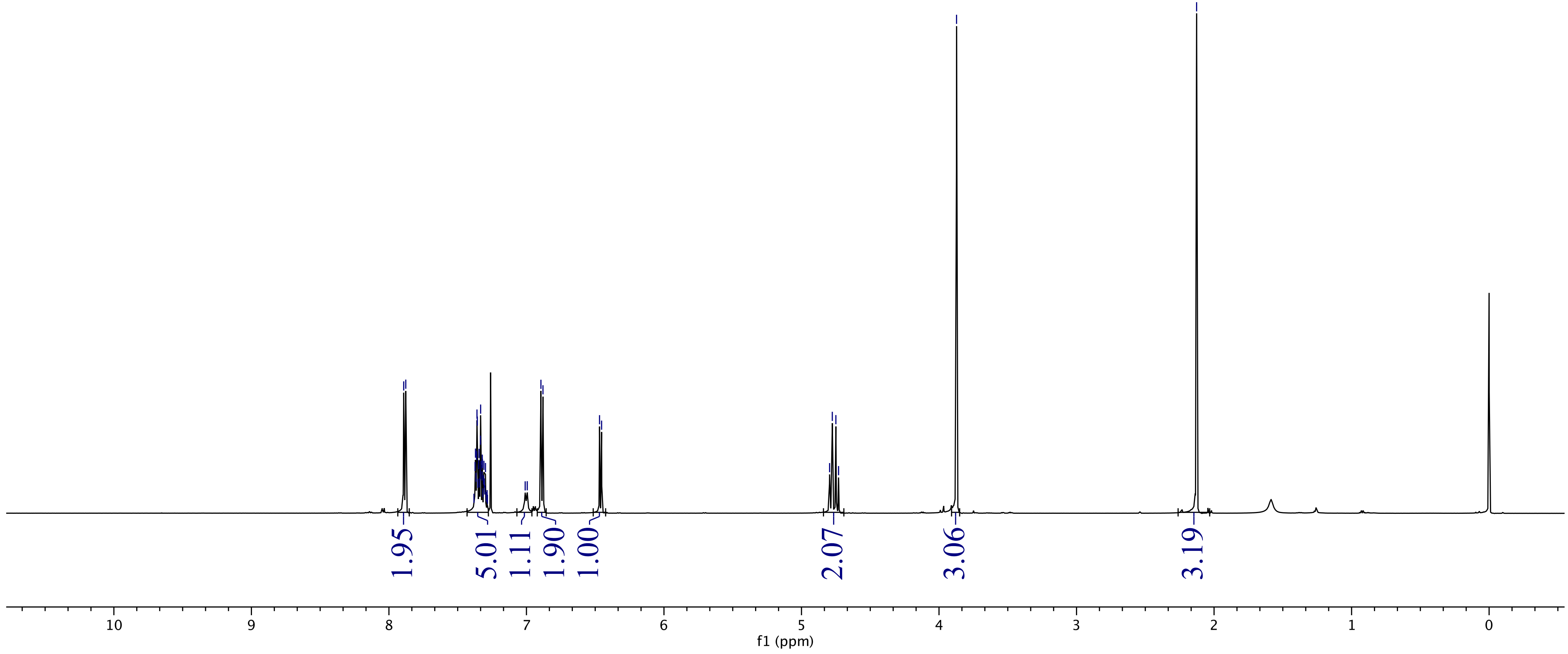

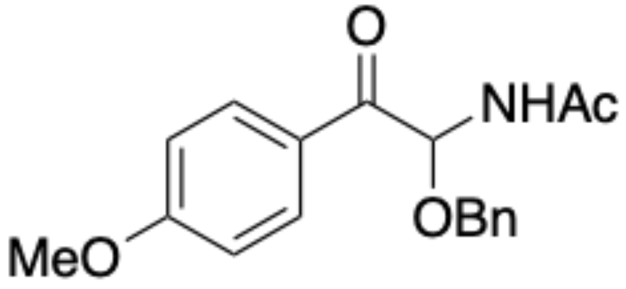

3bb for 13C{1H} NMR
(151 MHz, Chloroform-d)

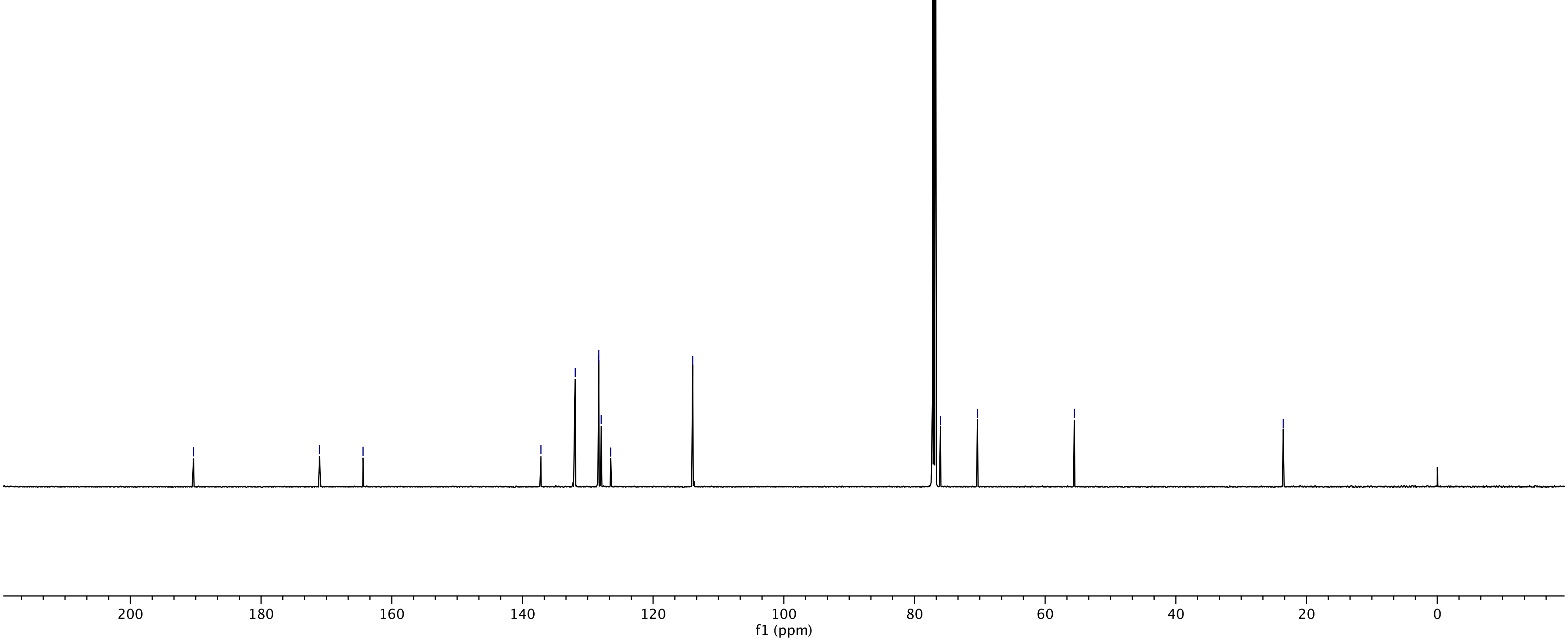

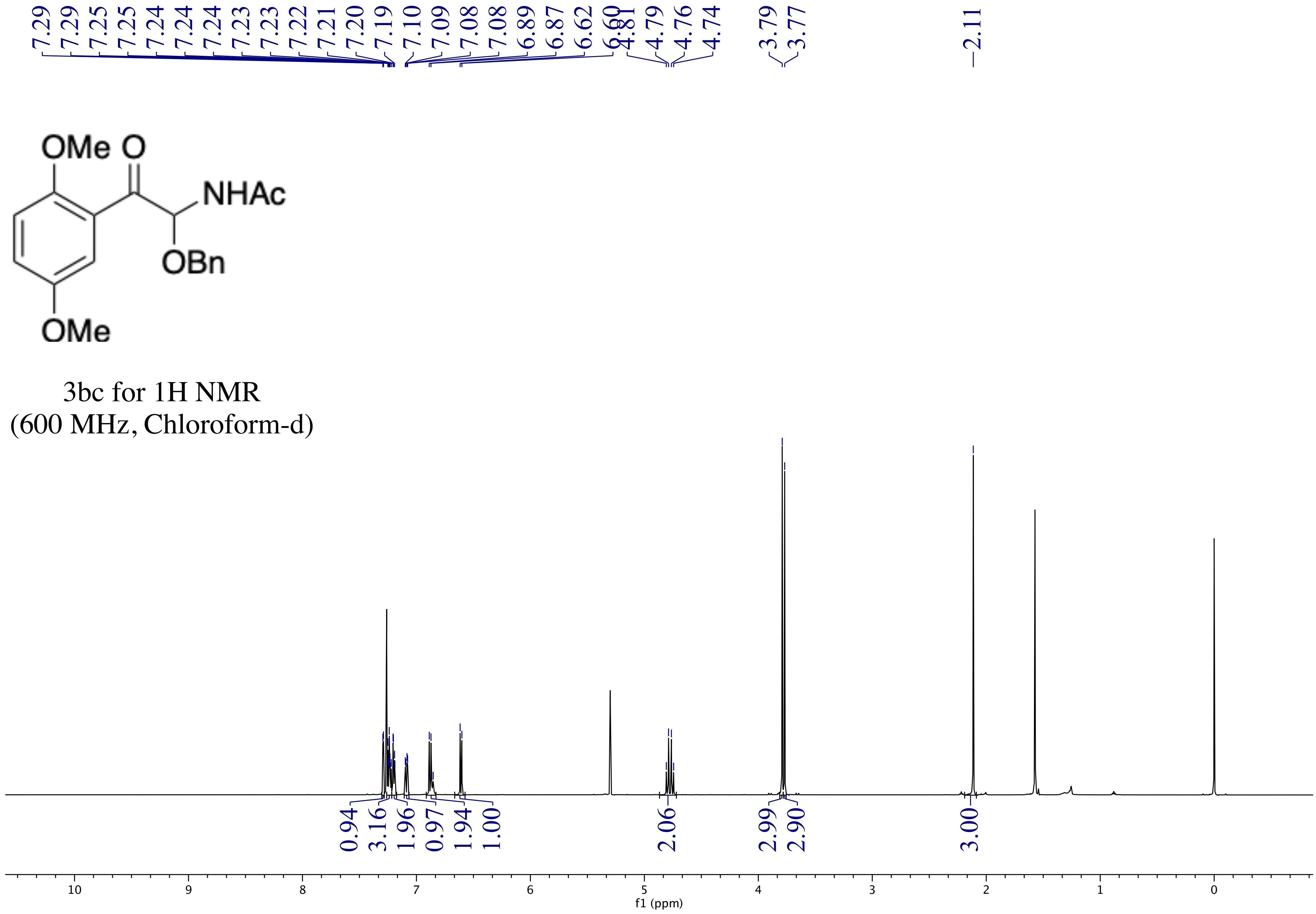

3bc for 1H NMR
(600 MHz, Chloroform-d)

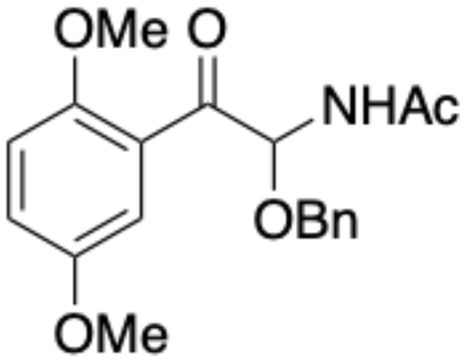

3bc for 13C{1H} NMR
(151 MHz, Chloroform-d)

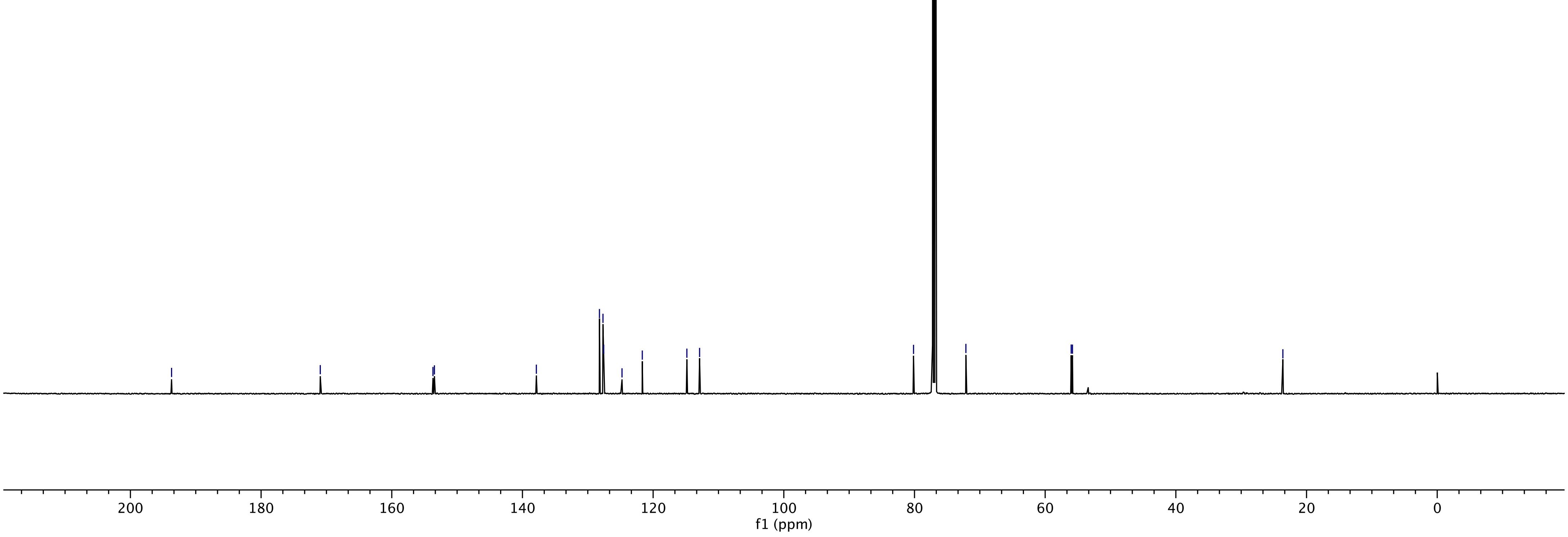

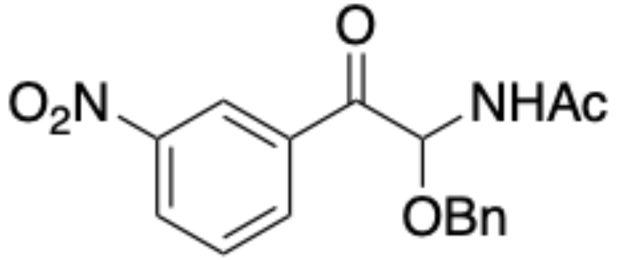

3bd for 1H NMR
(600 MHz, Chloroform-d)

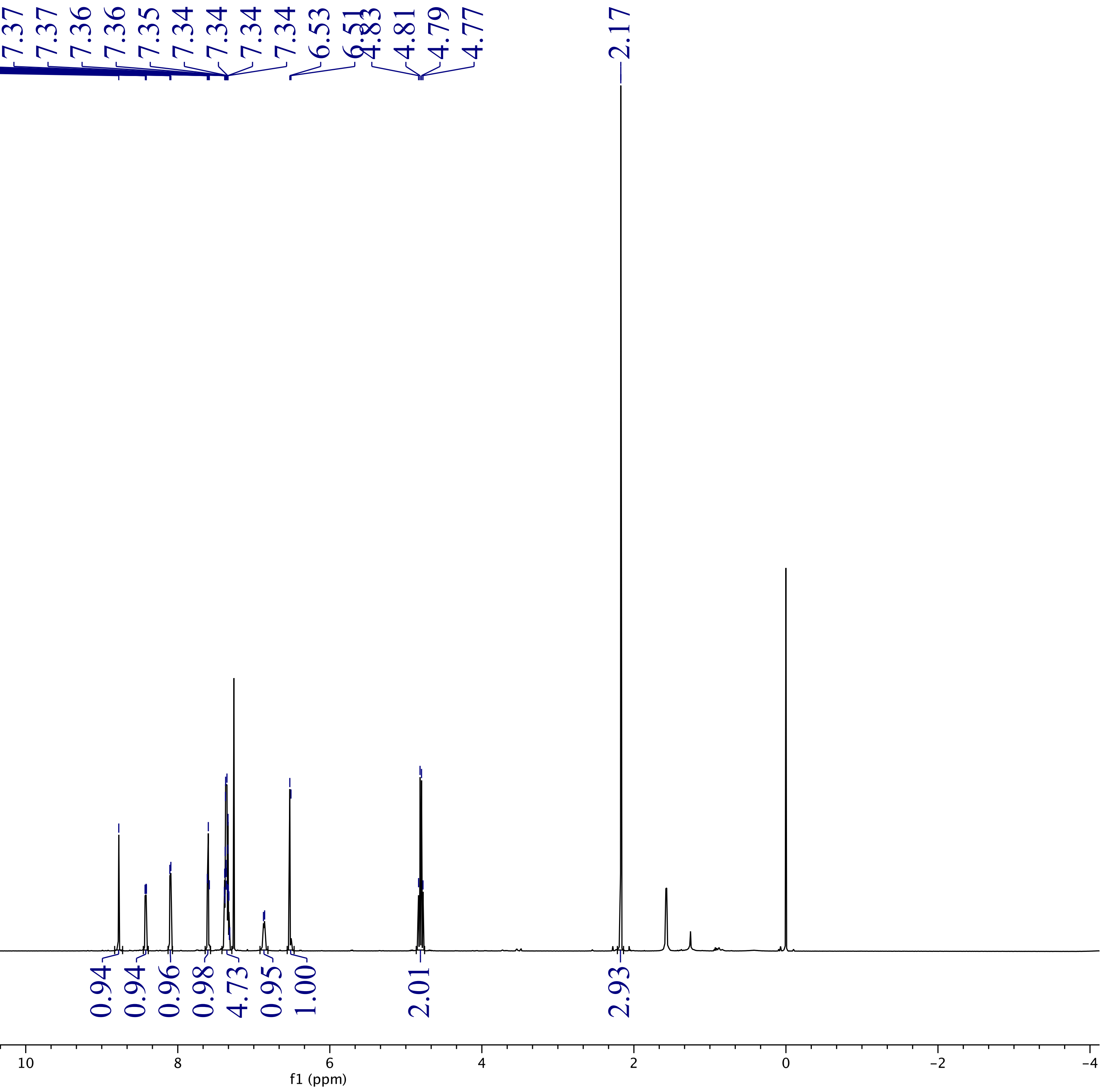

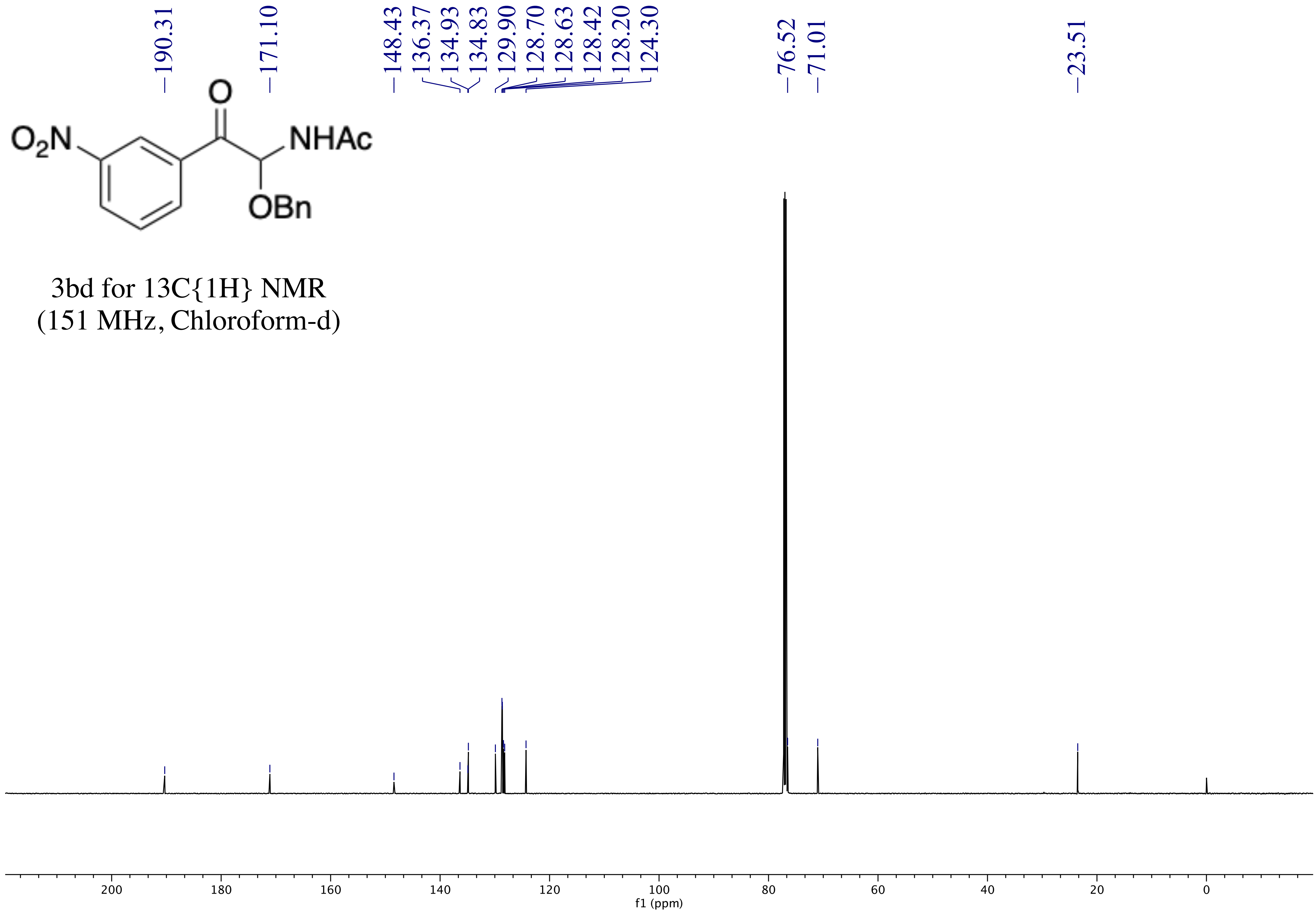

3bd for 13C{1H} NMR
(151 MHz, Chloroform-d)

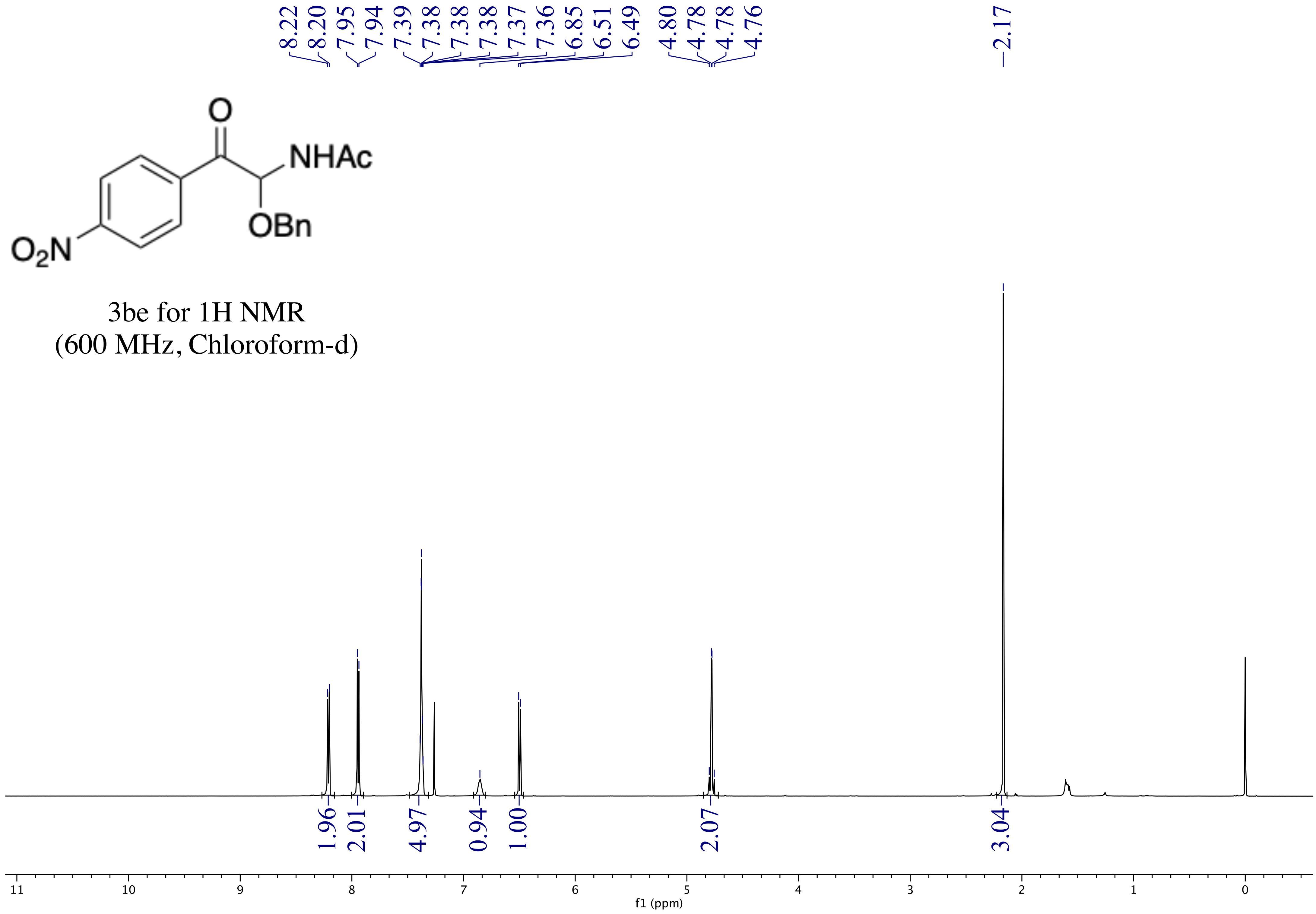

3be for 1H NMR
(600 MHz, Chloroform-d)

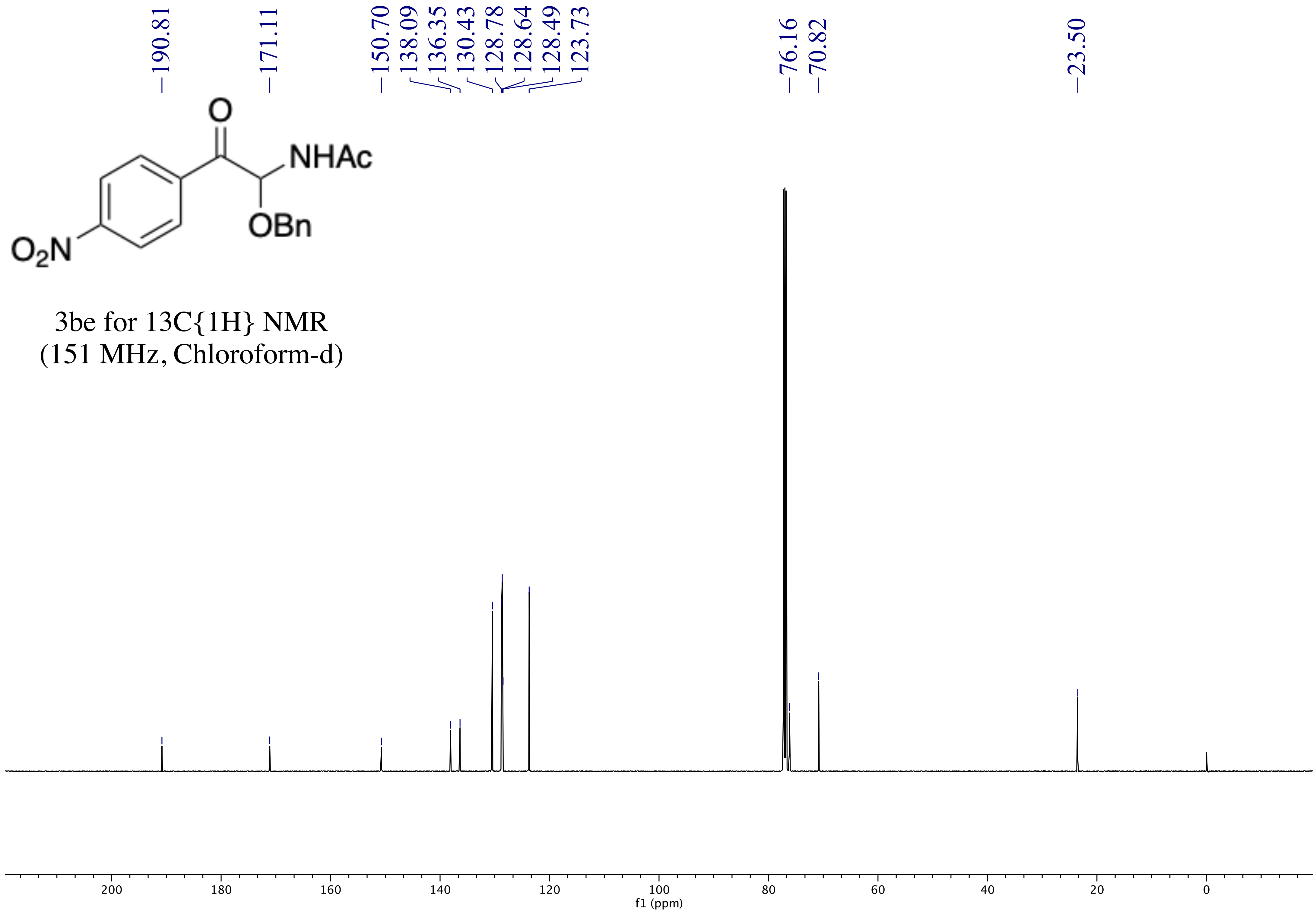

3be for 13C{1H} NMR
(151 MHz, Chloroform-d)
190.81
171.11
150.70
138.09
136.35
130.43
128.78
128.64
128.49
123.73
76.16
70.82
23.50
O
NHAc
OBn
O2N
200
180
160
140
120
100
80
60
40
20
0
f1 (ppm)

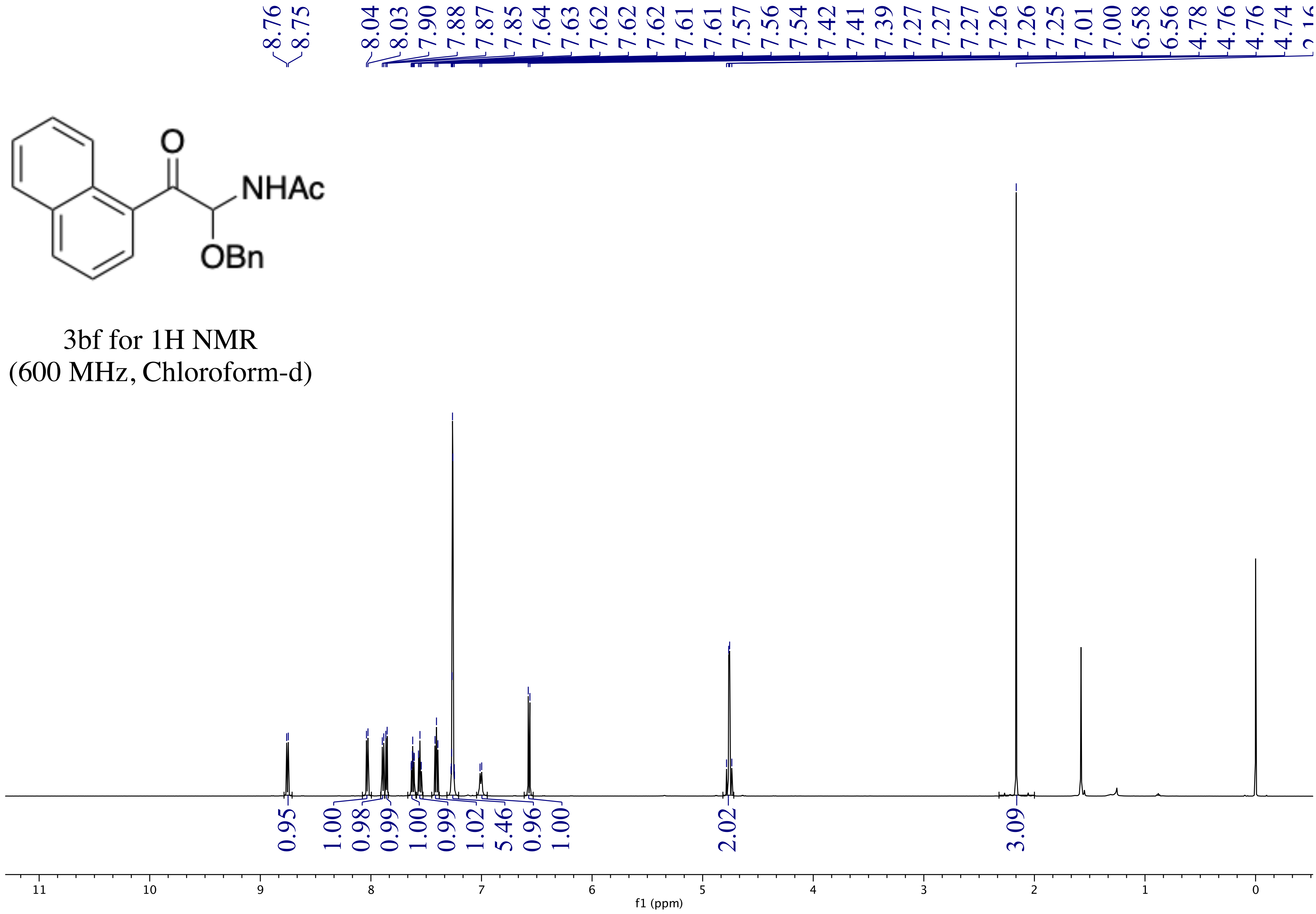

3bf for 1H NMR
(600 MHz, Chloroform-d)

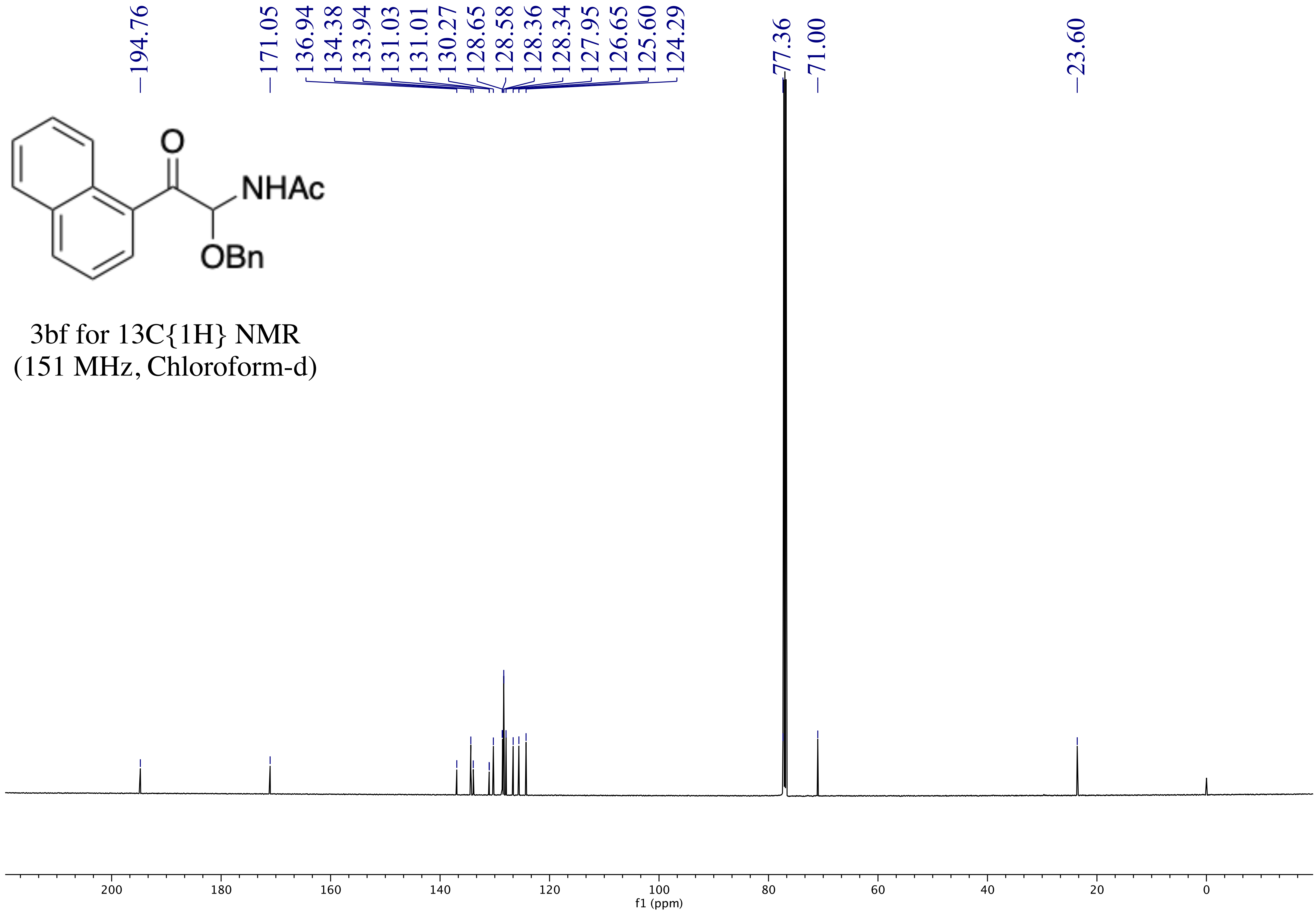
O
NHAc
OBn
3bf for 13C{1H} NMR
(151 MHz, Chloroform-d)
194.76
171.05
136.94
134.38
133.94
131.03
131.01
130.27
128.65
128.58
128.36
128.34
127.95
126.65
125.60
124.29
77.36
71.00
23.60
200
180
160
140
120
100
80
60
40
20
0
f1 (ppm)

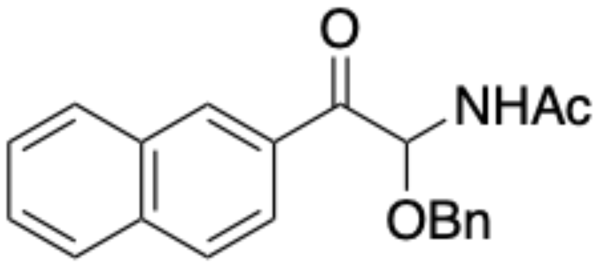

3bg for 1H NMR
(600 MHz, Chloroform-d)

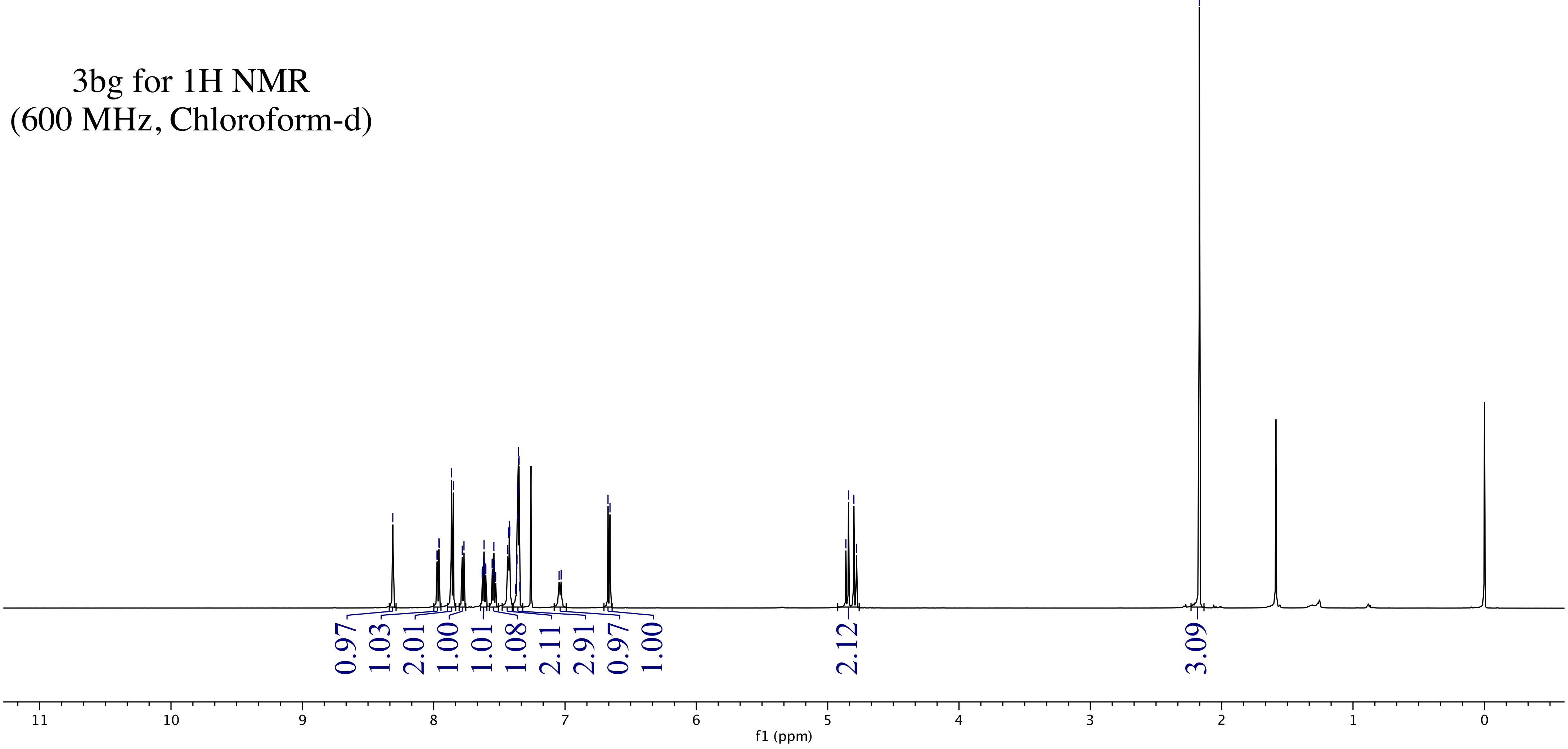

3bg for 13C{1H} NMR
(151 MHz, Chloroform-d)

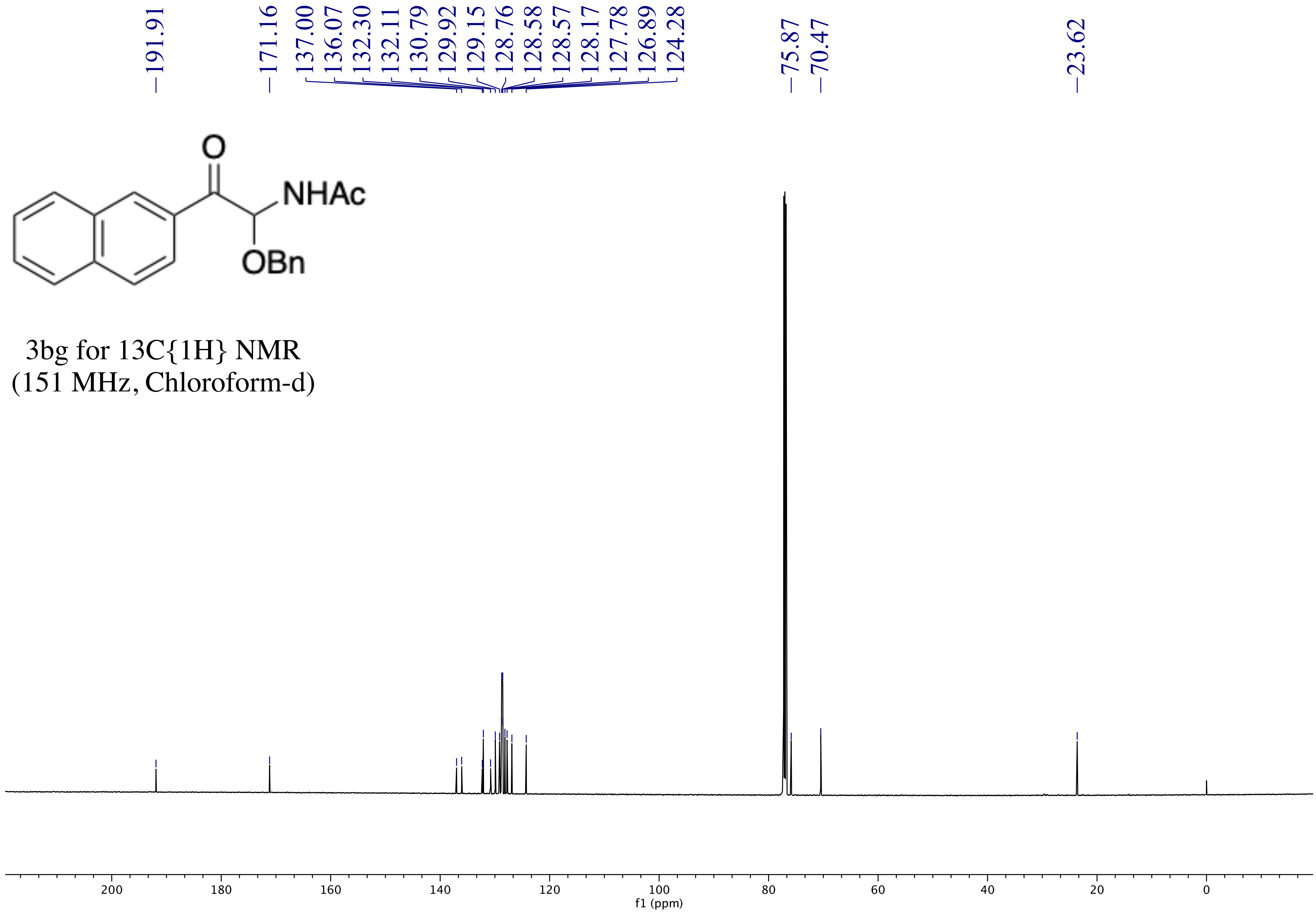

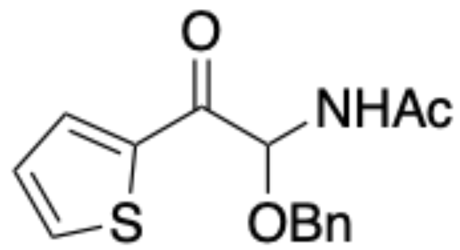

3bh for 1H NMR
(600 MHz, Chloroform-d)

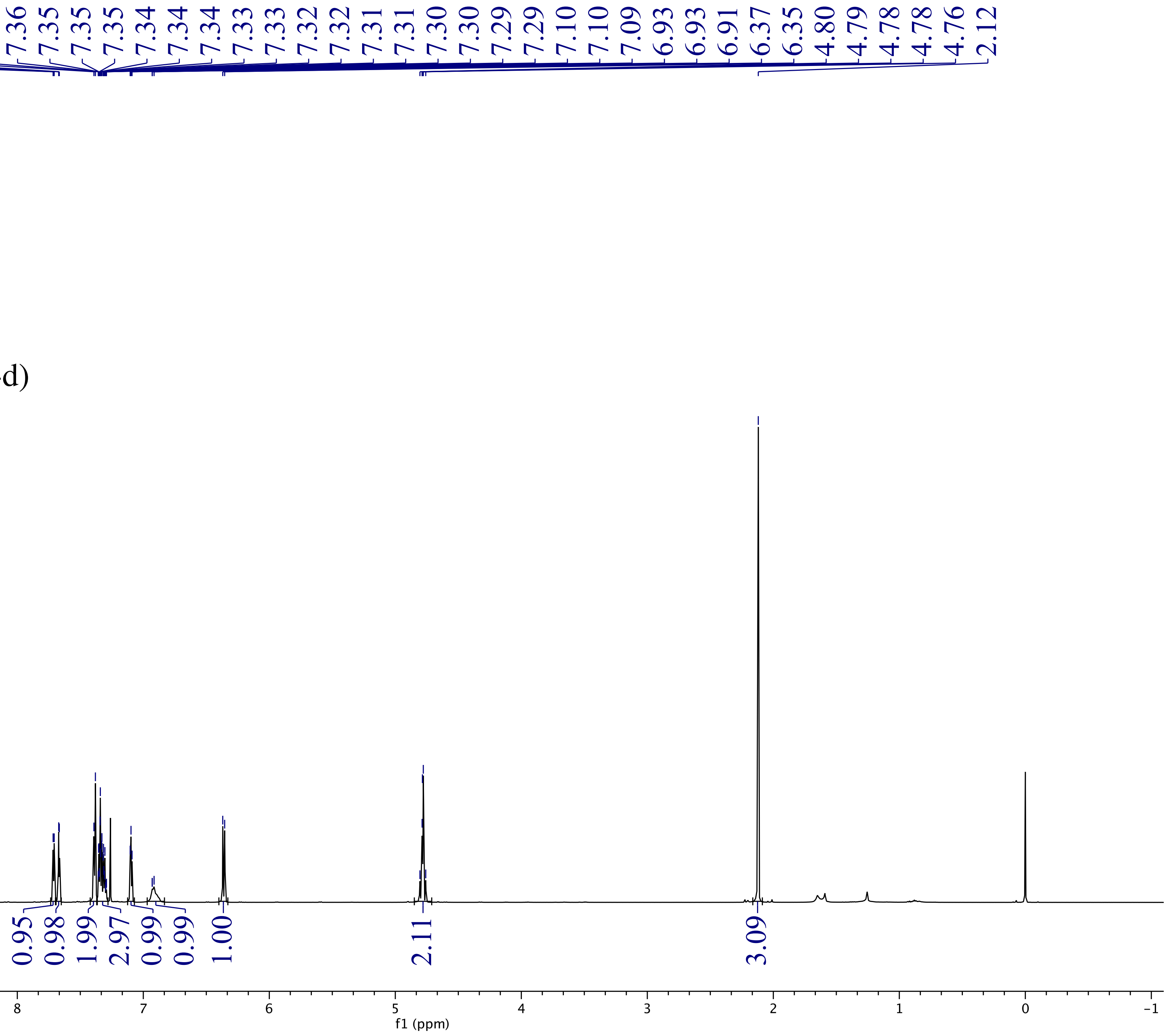

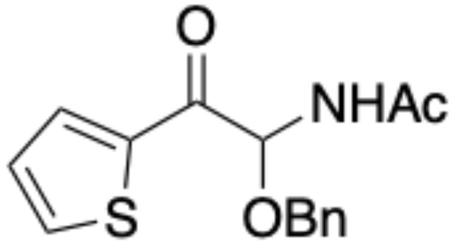

3bh for 13C{1H} NMR
(151 MHz, Chloroform-d)

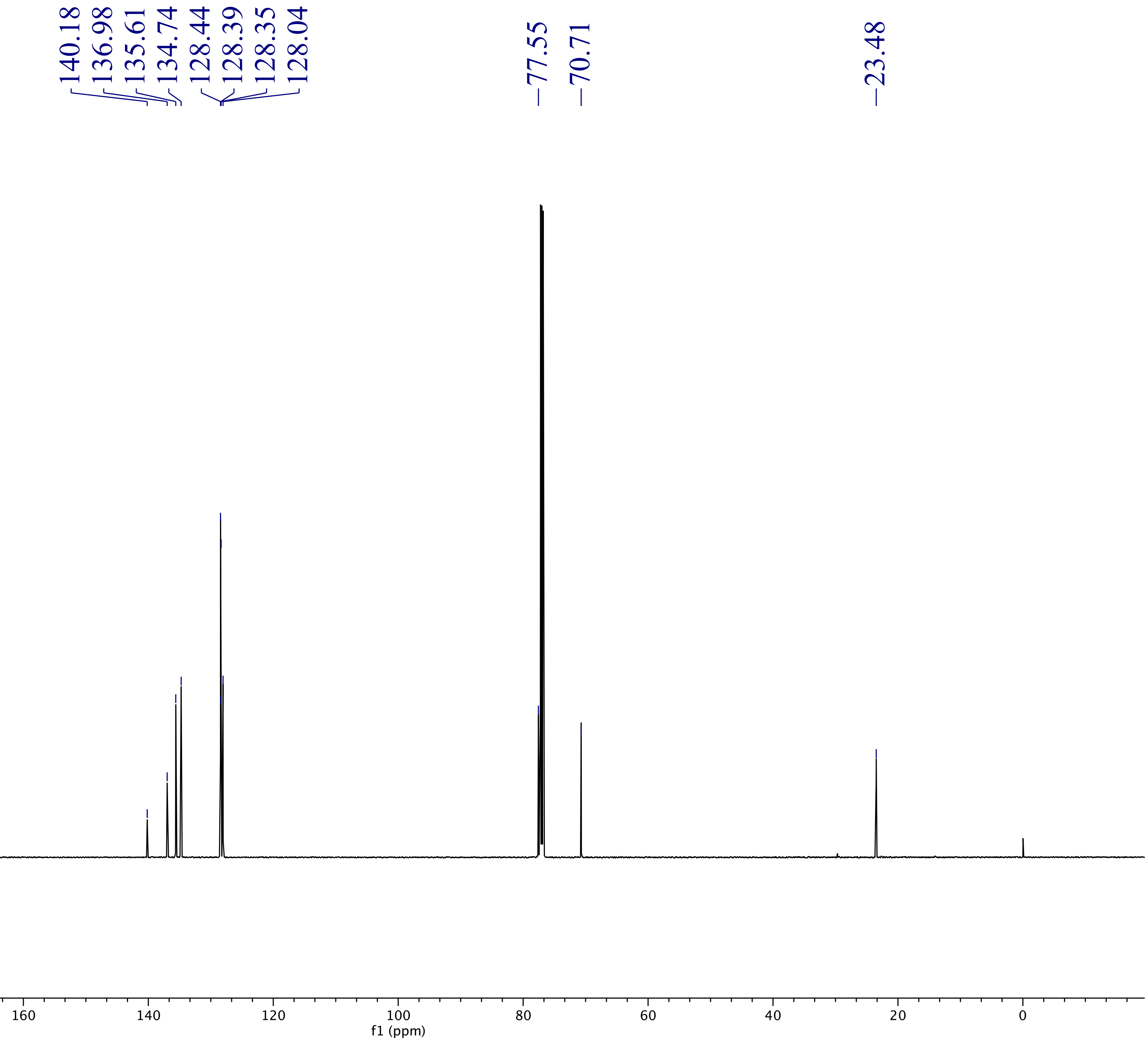

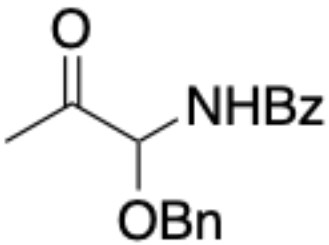

3bi for 1H NMR
(600 MHz, Chloroform-d)

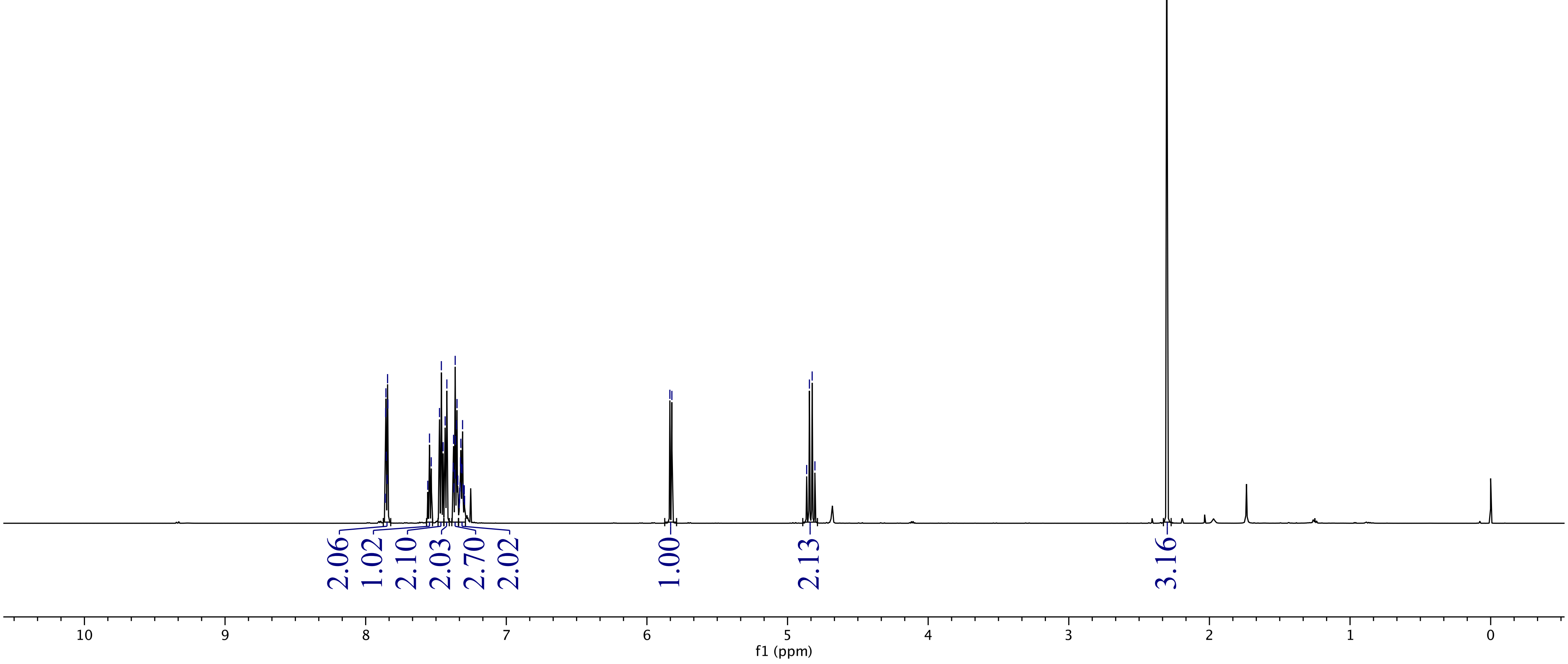

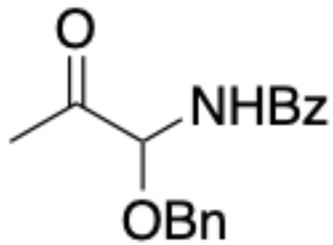

3bi for 13C{1H} NMR
(151 MHz, Chloroform-d)

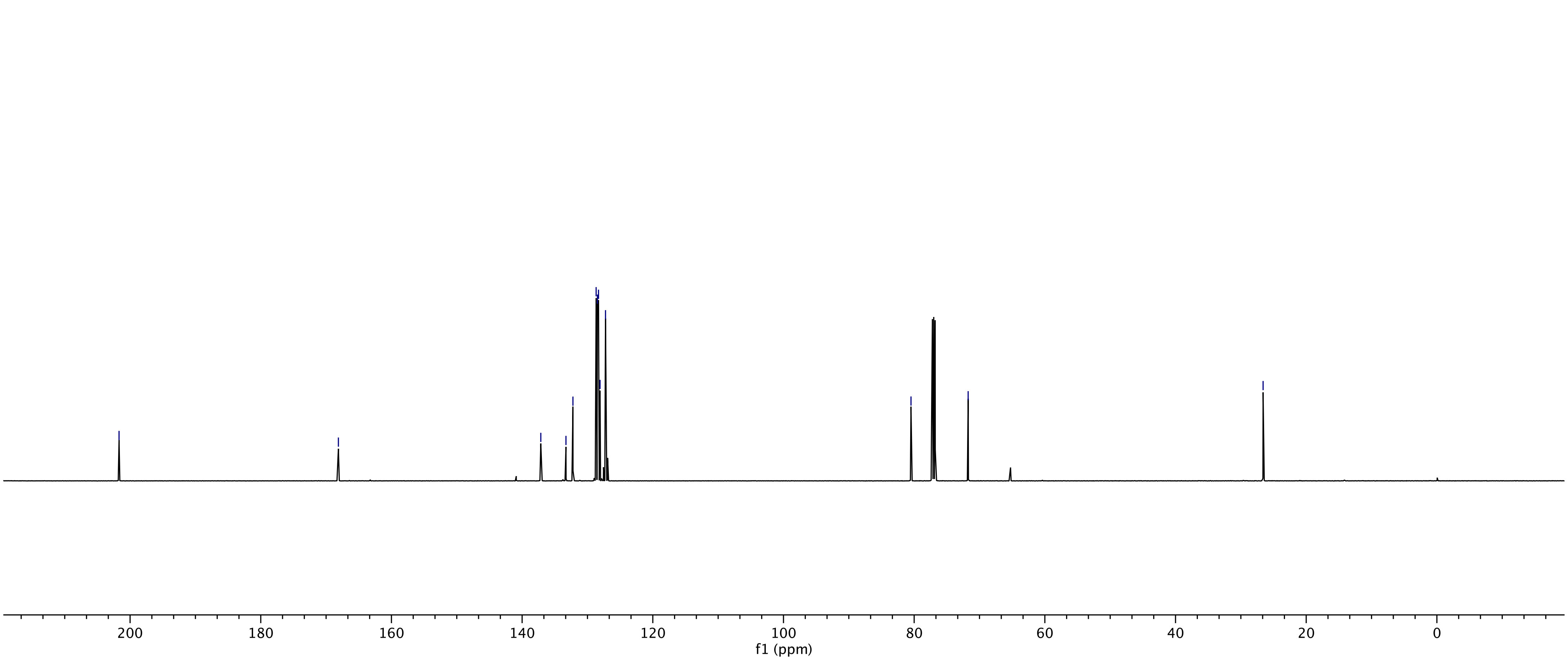

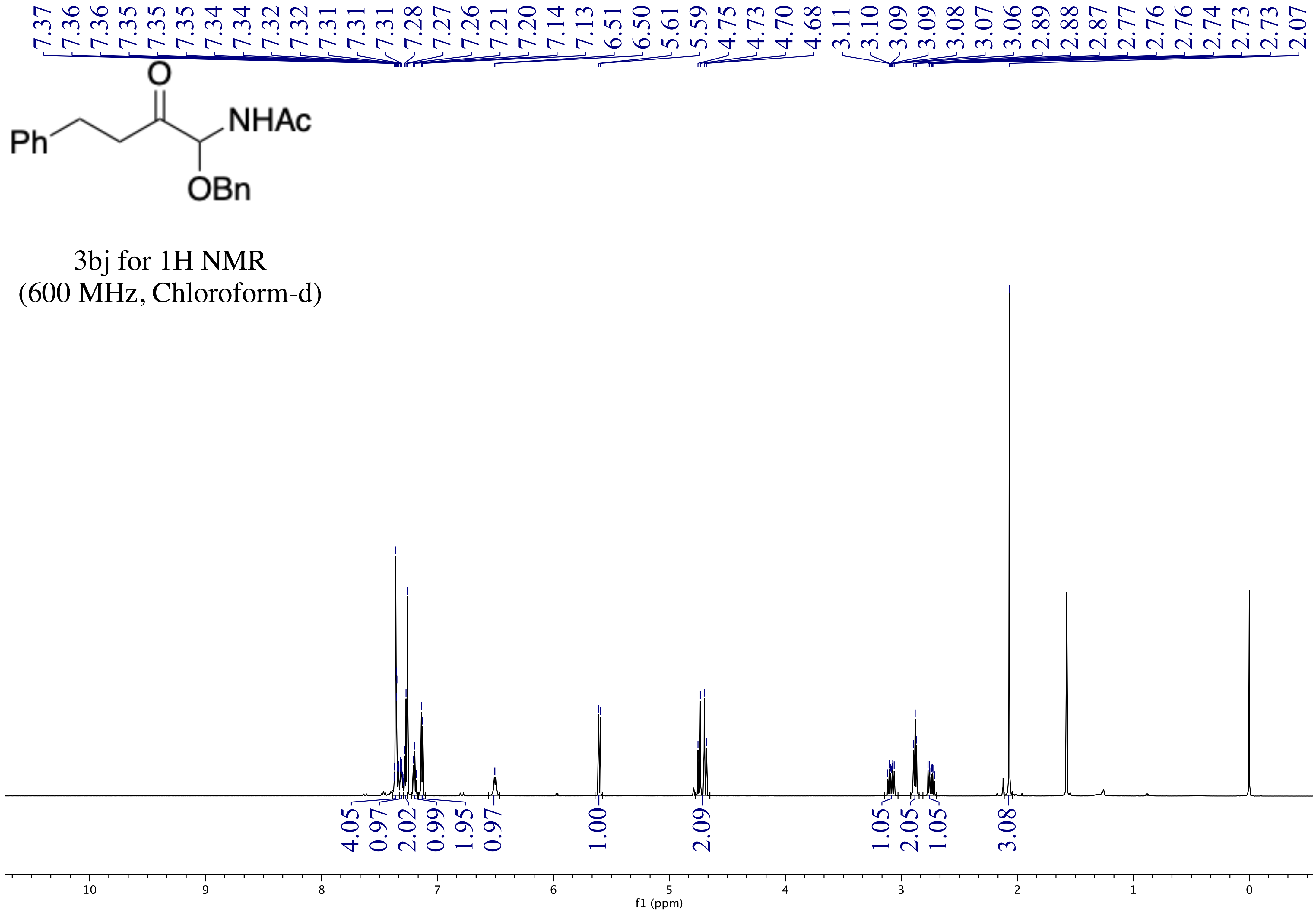

3bj for 1H NMR
(600 MHz, Chloroform-d)

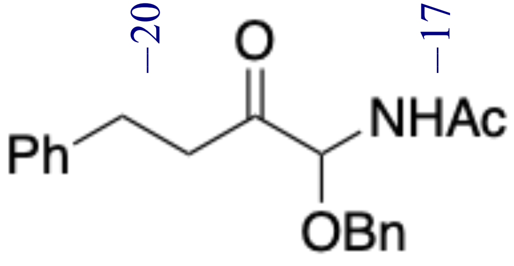

3bj for 13C{1H} NMR
(151 MHz, Chloroform-d)

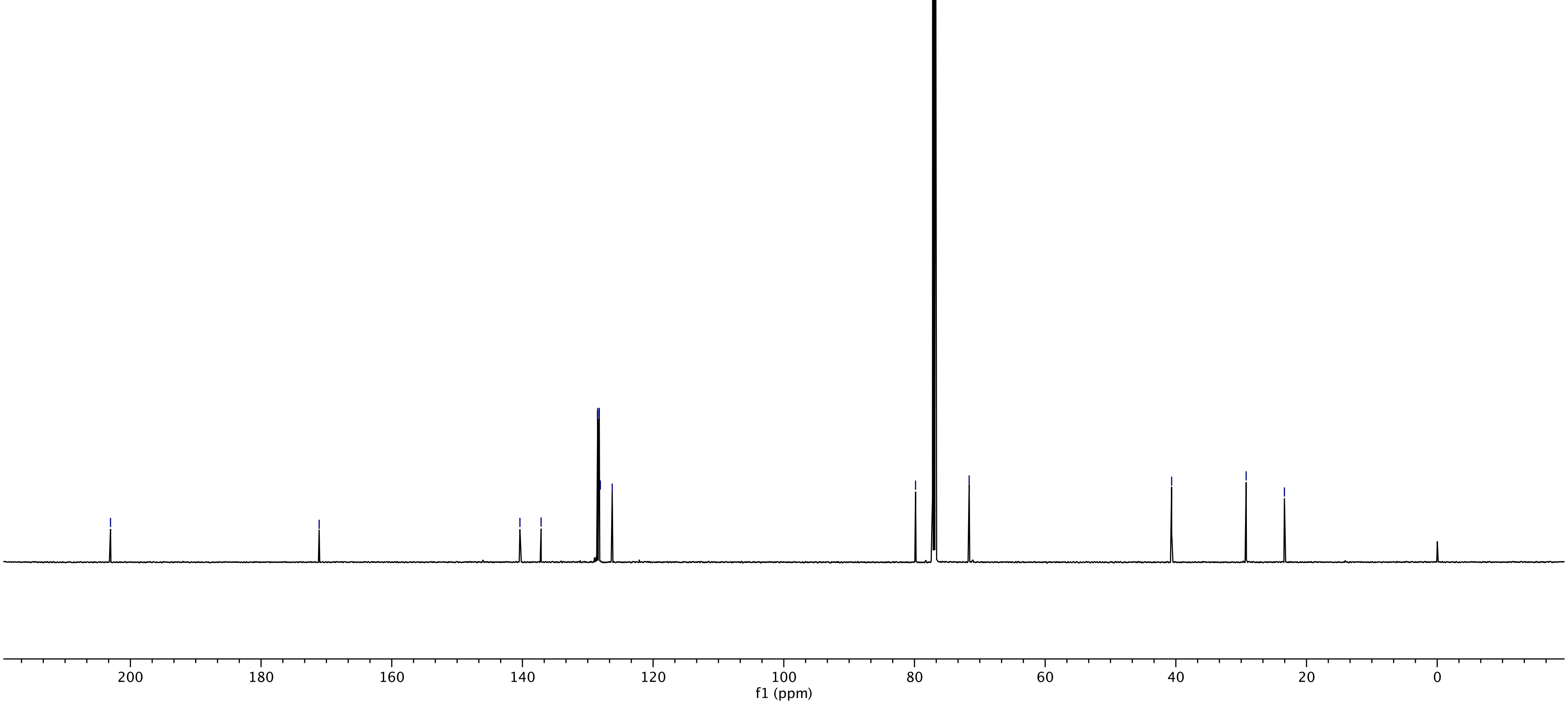

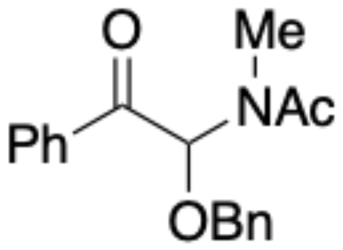

3bk for 1H NMR
(600 MHz, Chloroform-d)

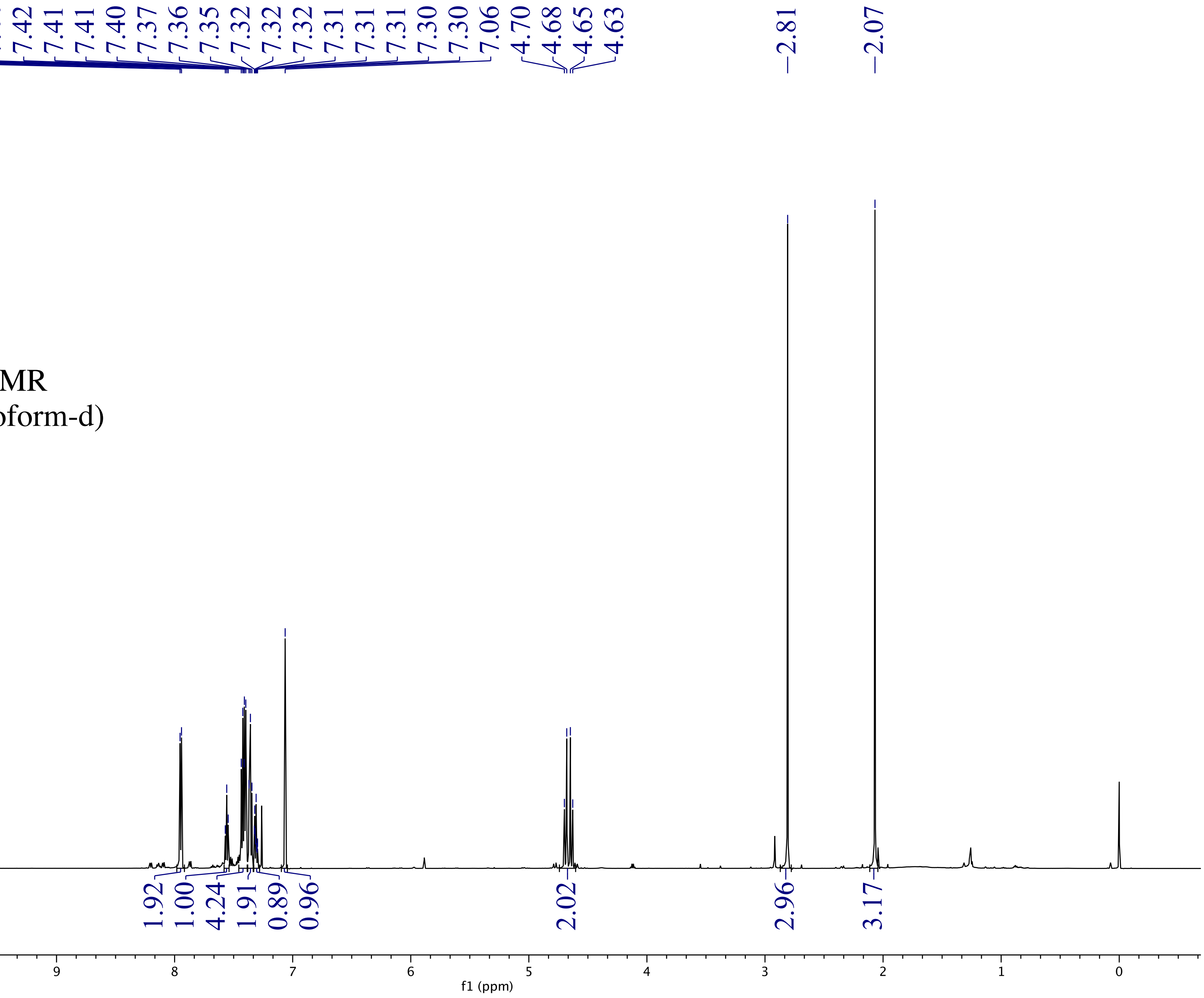

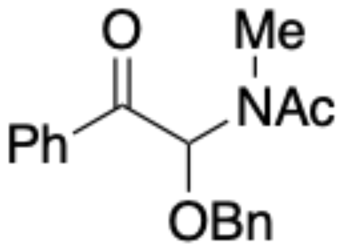

3bk for 13C{1H} NMR
(151 MHz, Chloroform-d)

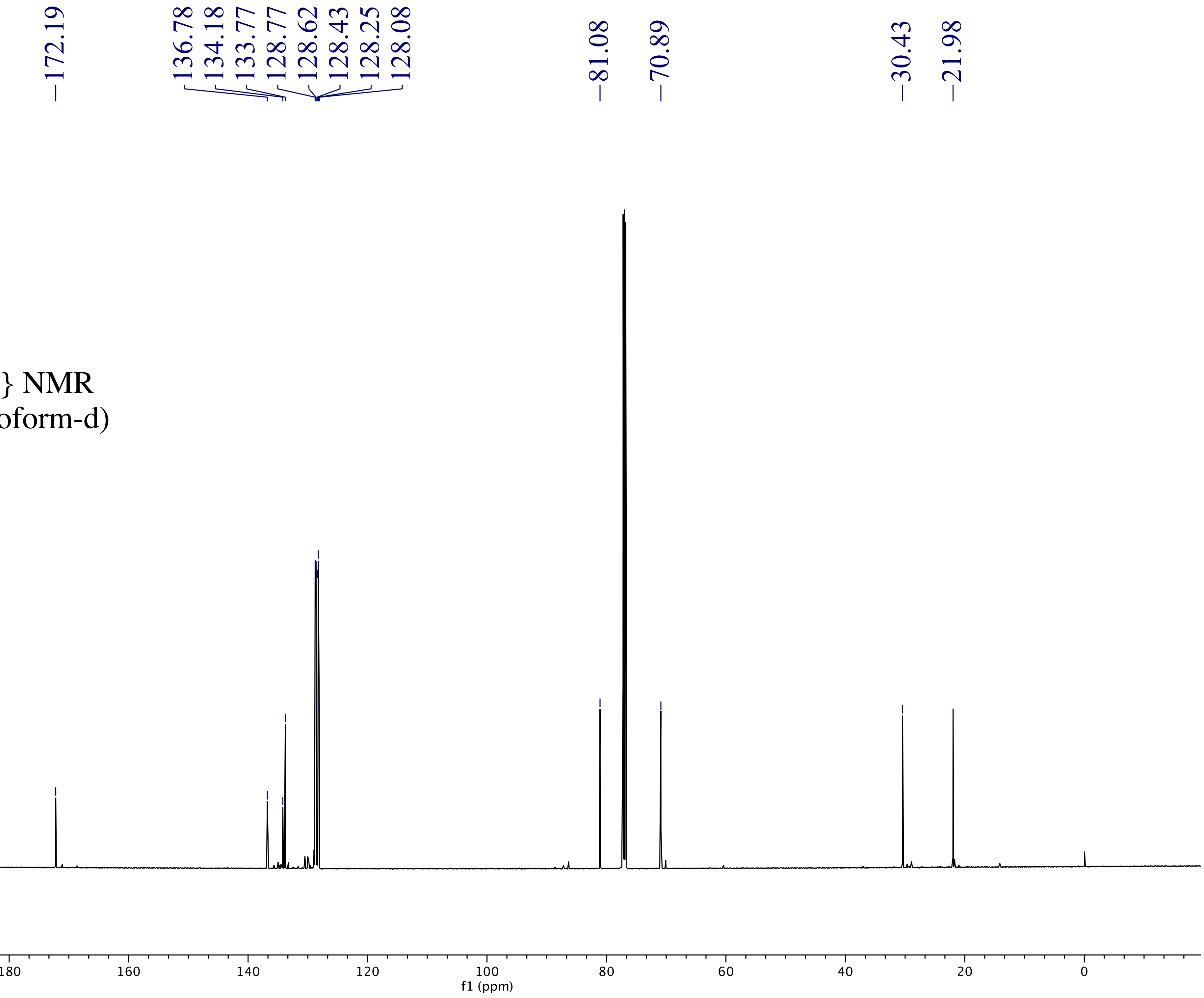

N-(1-oxo-1-(p-tolyl)propan-2-yl)benzamide for 1H NMR
(600 MHz, Chloroform-d)

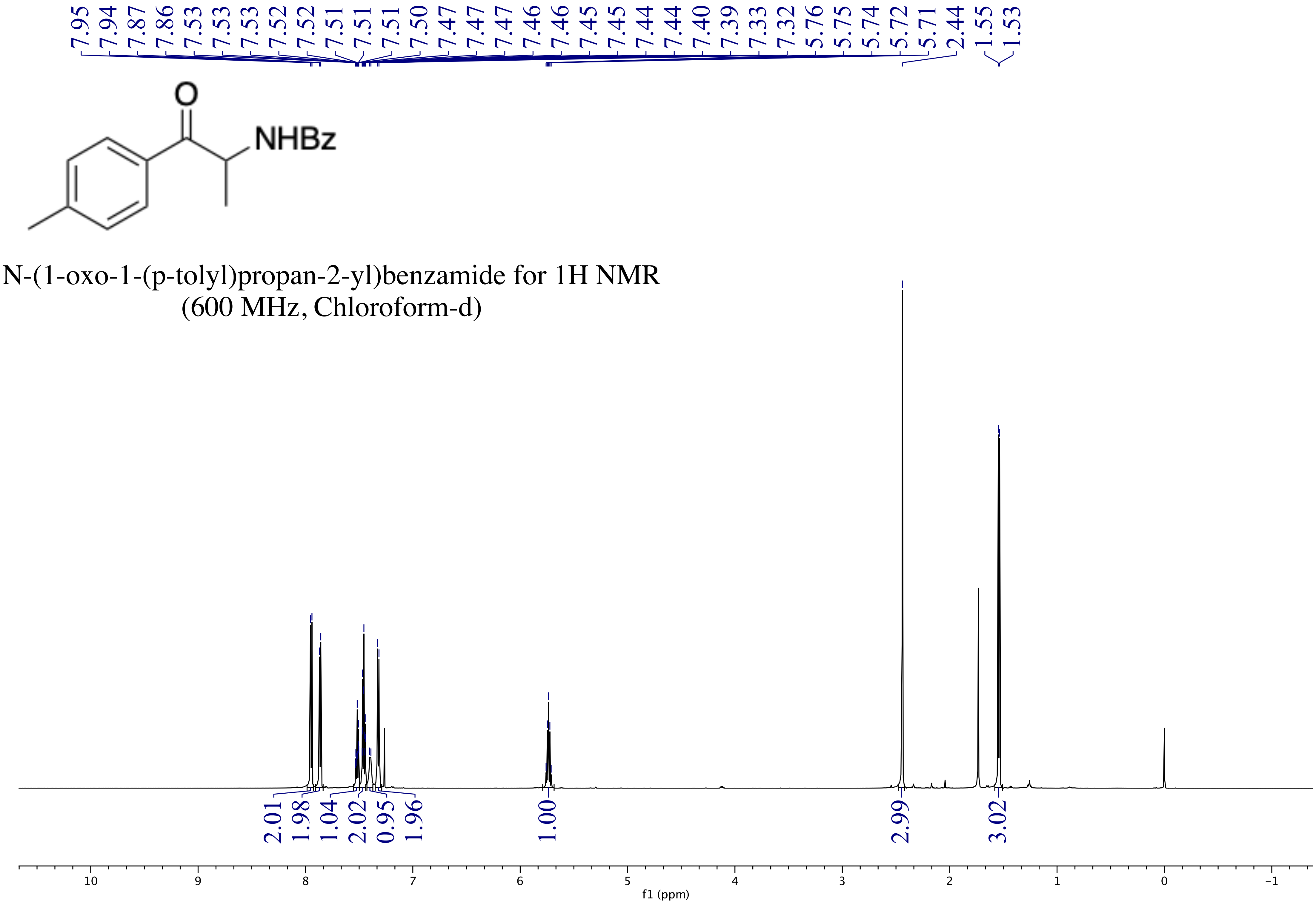

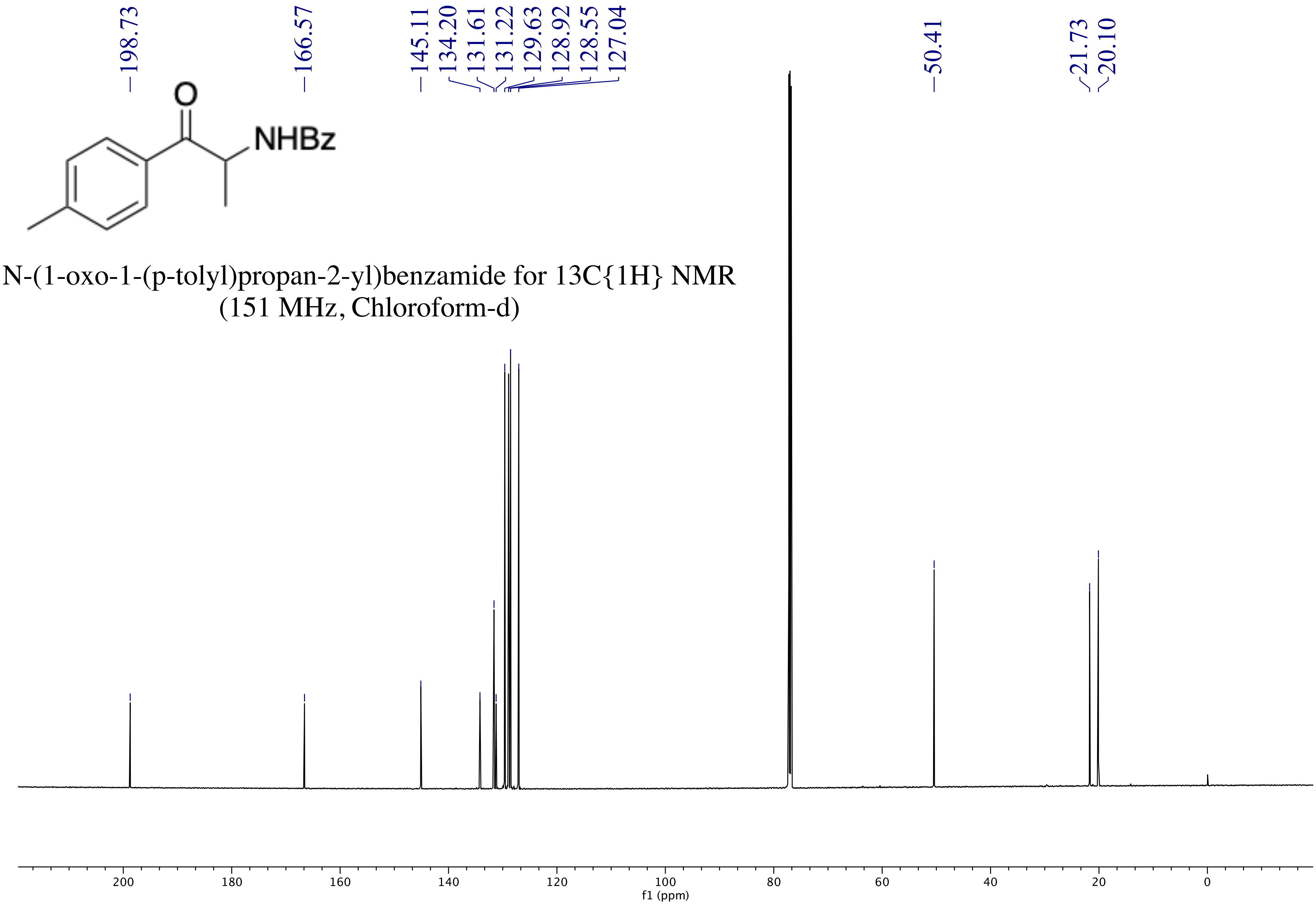

N-(1-oxo-1-(p-tolyl)propan-2-yl)benzamide for 13C{1H} NMR
(151 MHz, Chloroform-d)

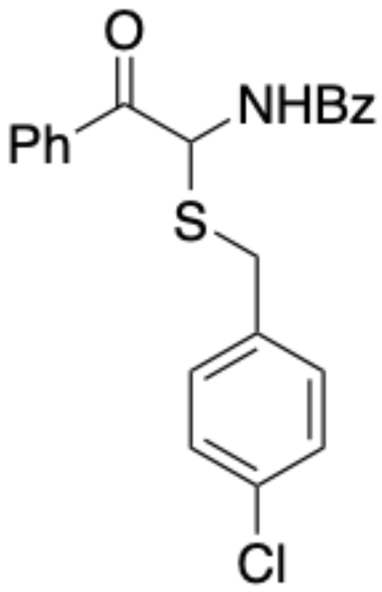

4a for 1H NMR
(600 MHz, Chloroform-d)

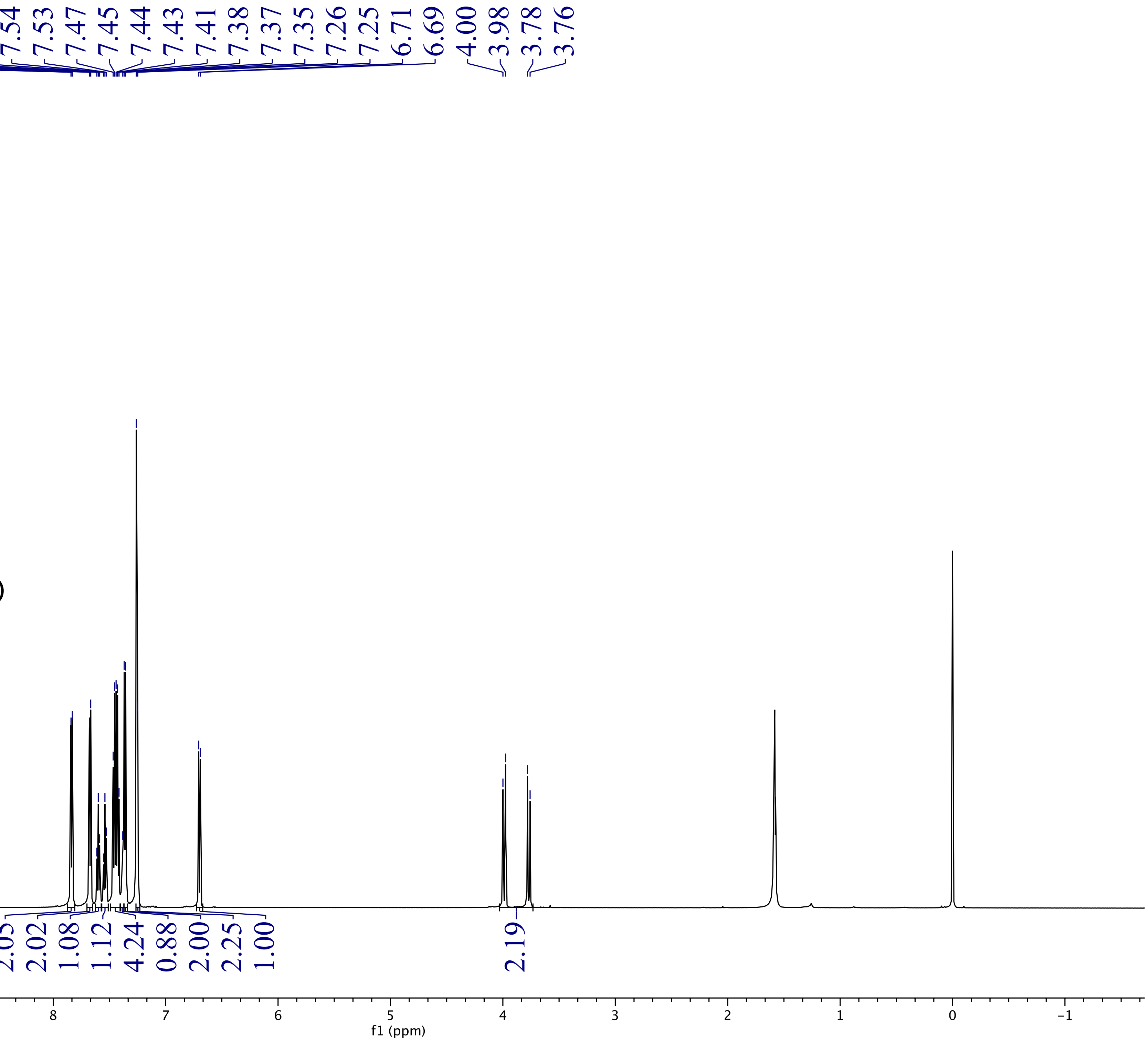

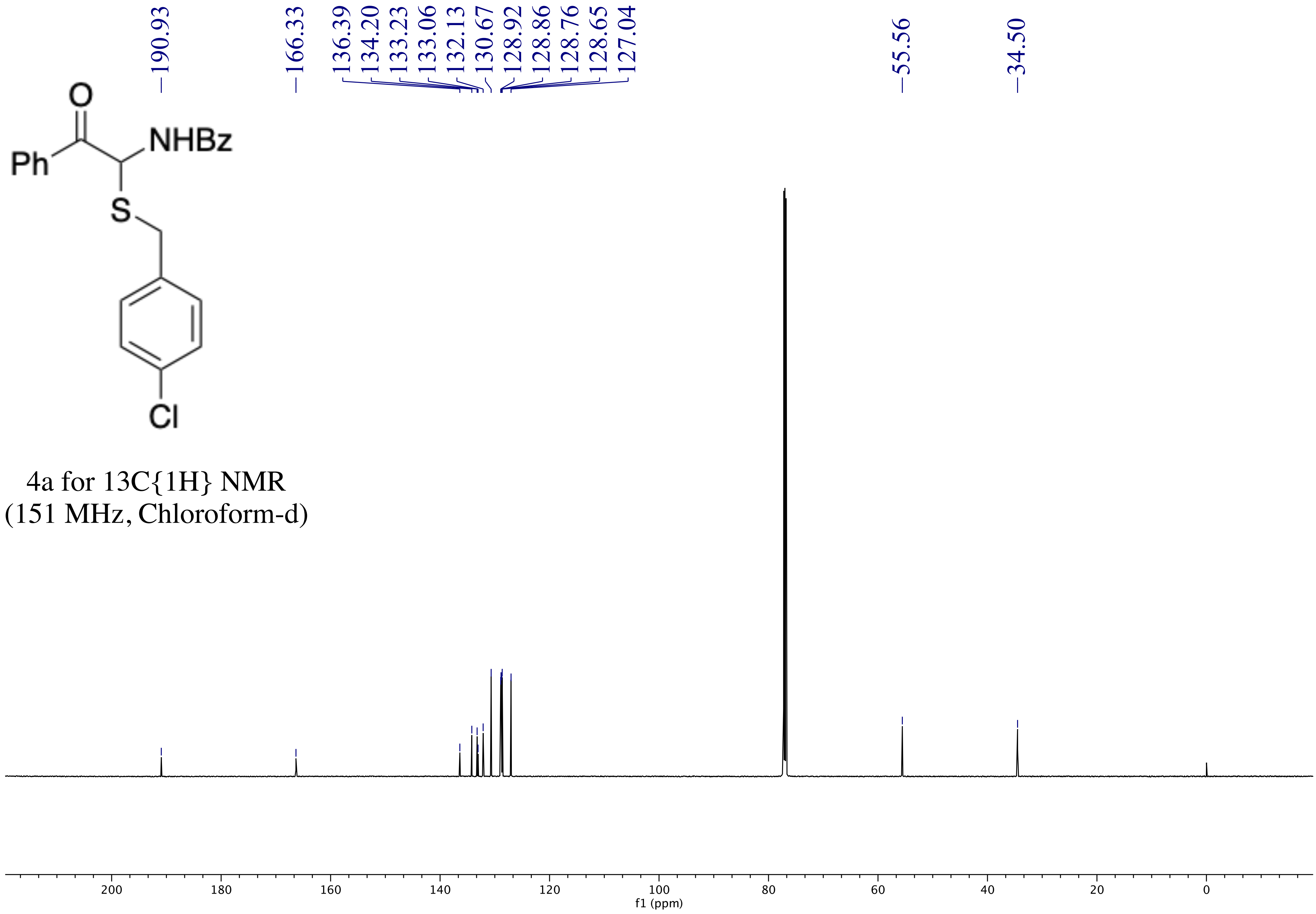

190.93
166.33
136.39
134.20
133.23
133.06
132.13
130.67
128.92
128.86
128.76
128.65
127.04
55.56
34.50
O
Ph
NHBz
S
Cl
4a for 13C{1H} NMR
(151 MHz, Chloroform-d)
200
180
160
140
120
100
80
60
40
20
0
f1 (ppm)

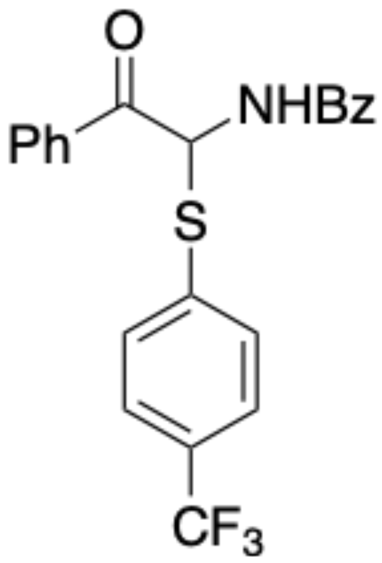

4b for 1H NMR
(600 MHz, Chloroform-d)

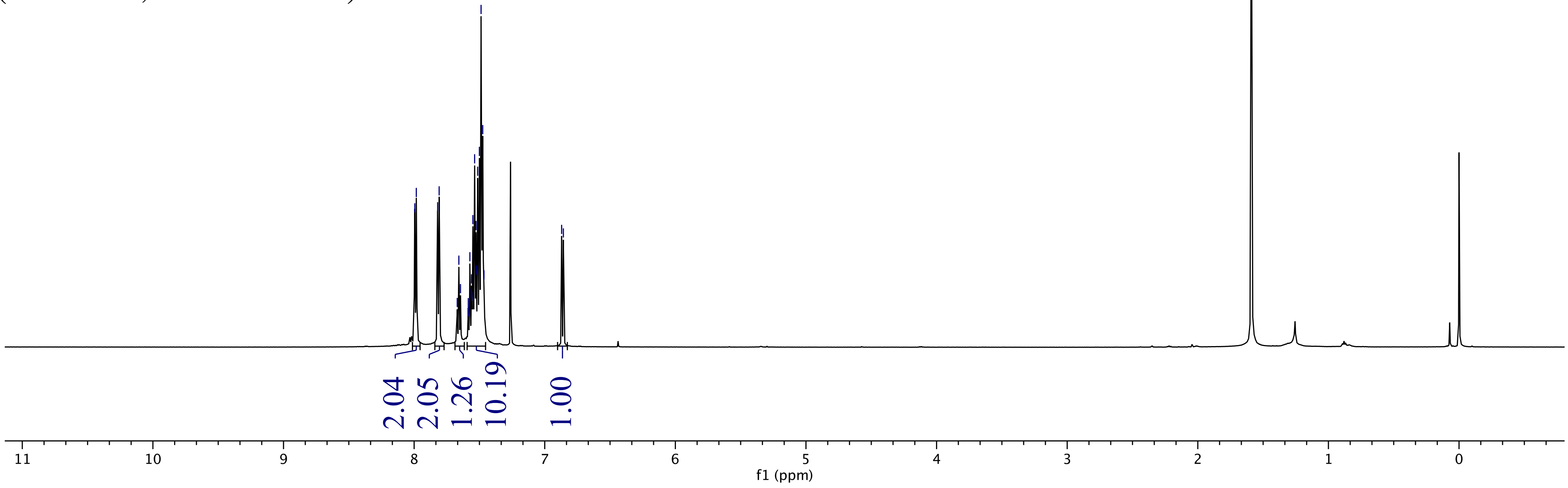

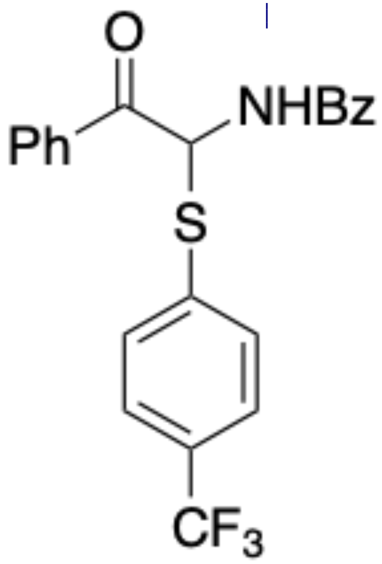

4b for 13C{1H} NMR
(151 MHz, Chloroform-d)

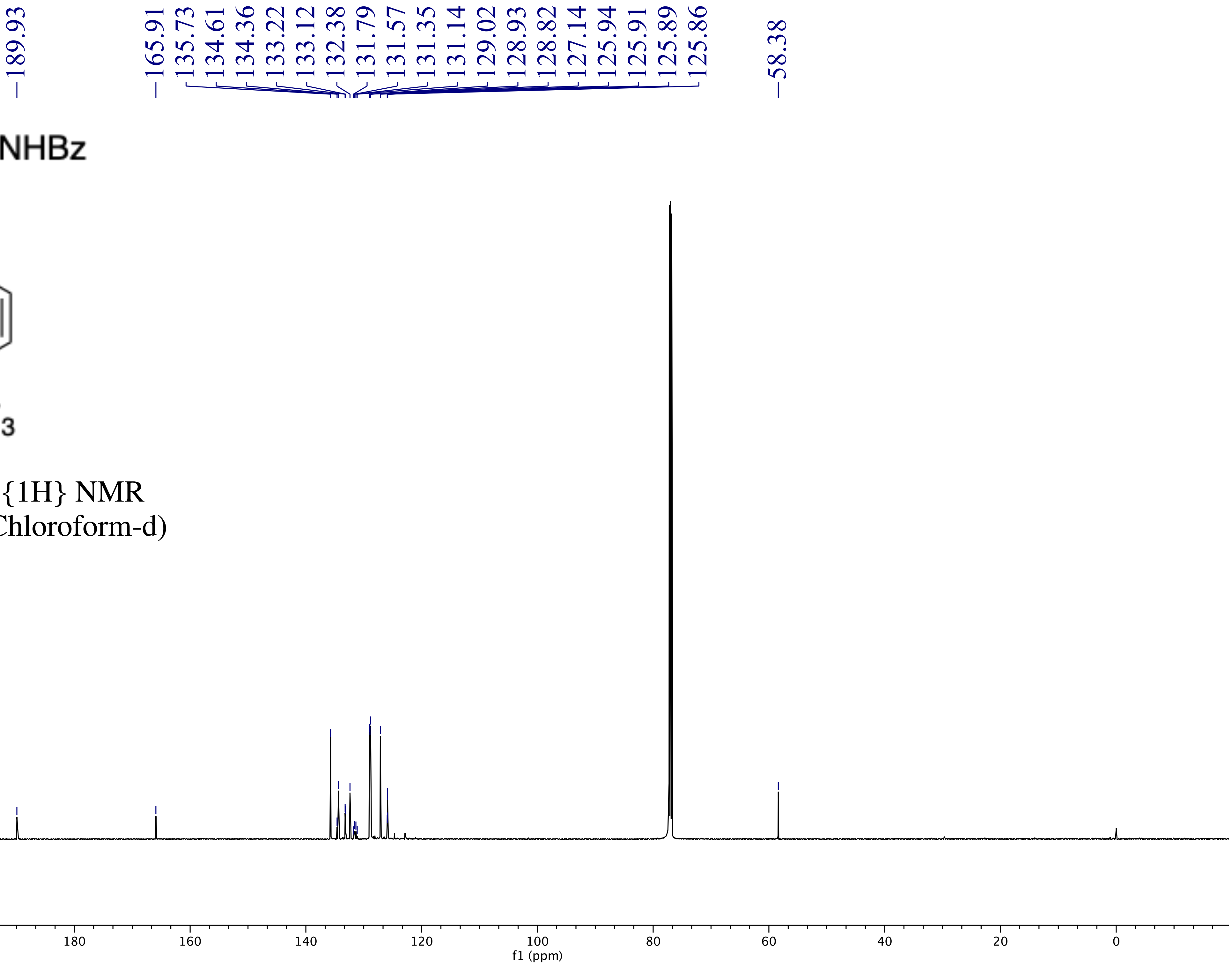

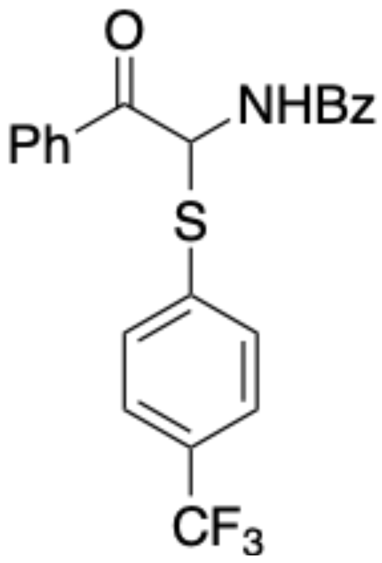

4b for 19F NMR
(376 MHz, Chloroform-d)

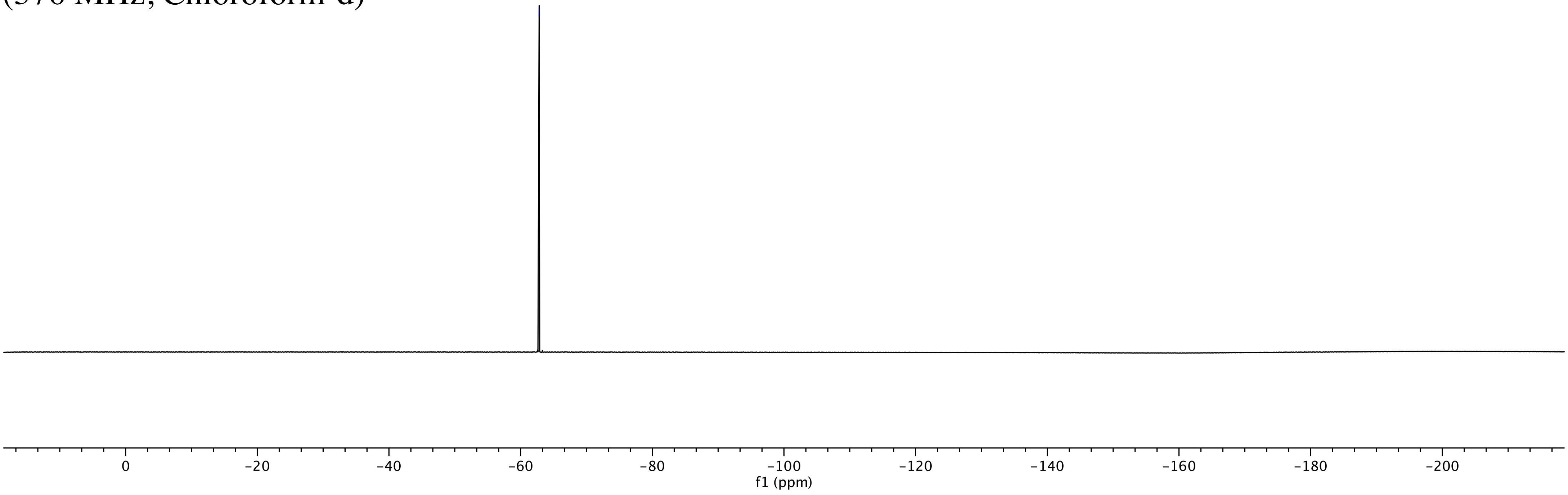

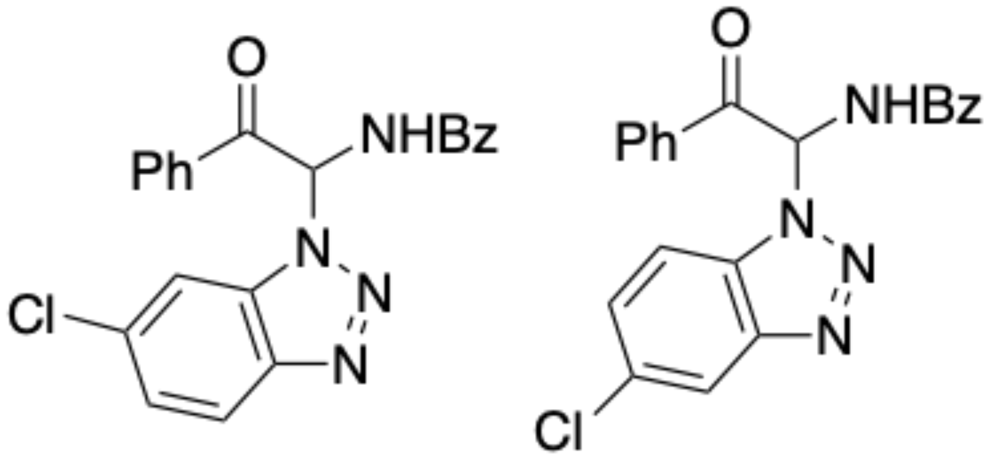

4c for 1H NMR
(600 MHz, Chloroform-d)

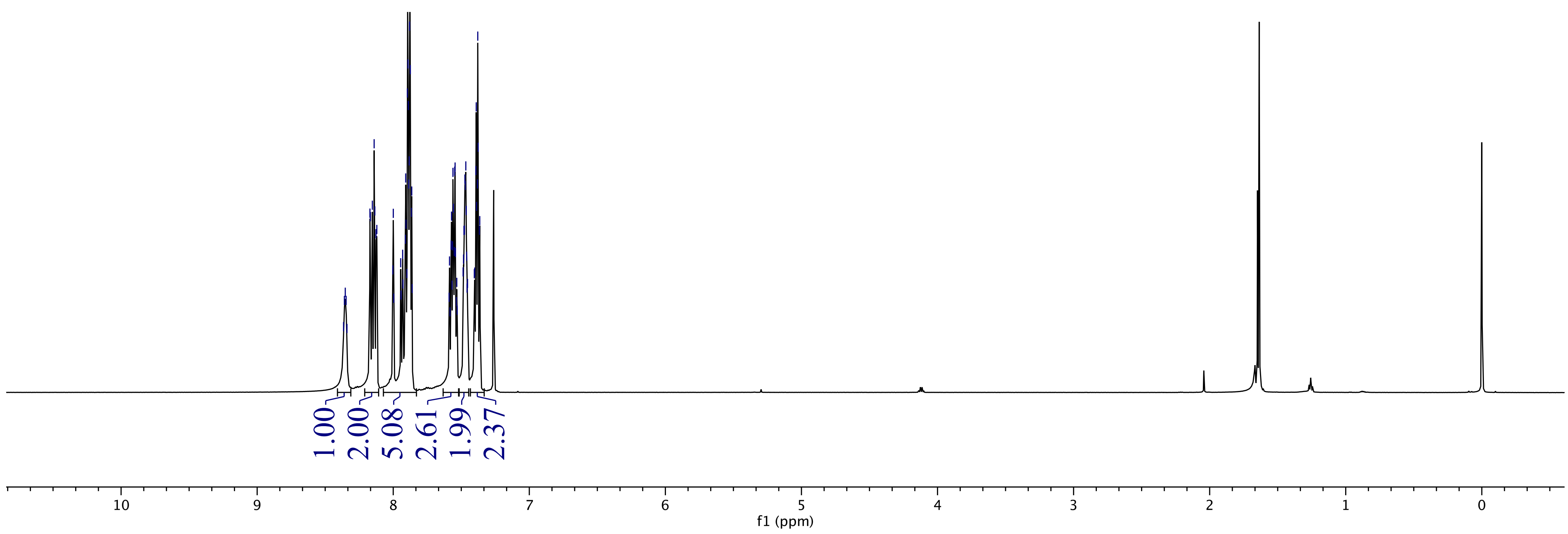

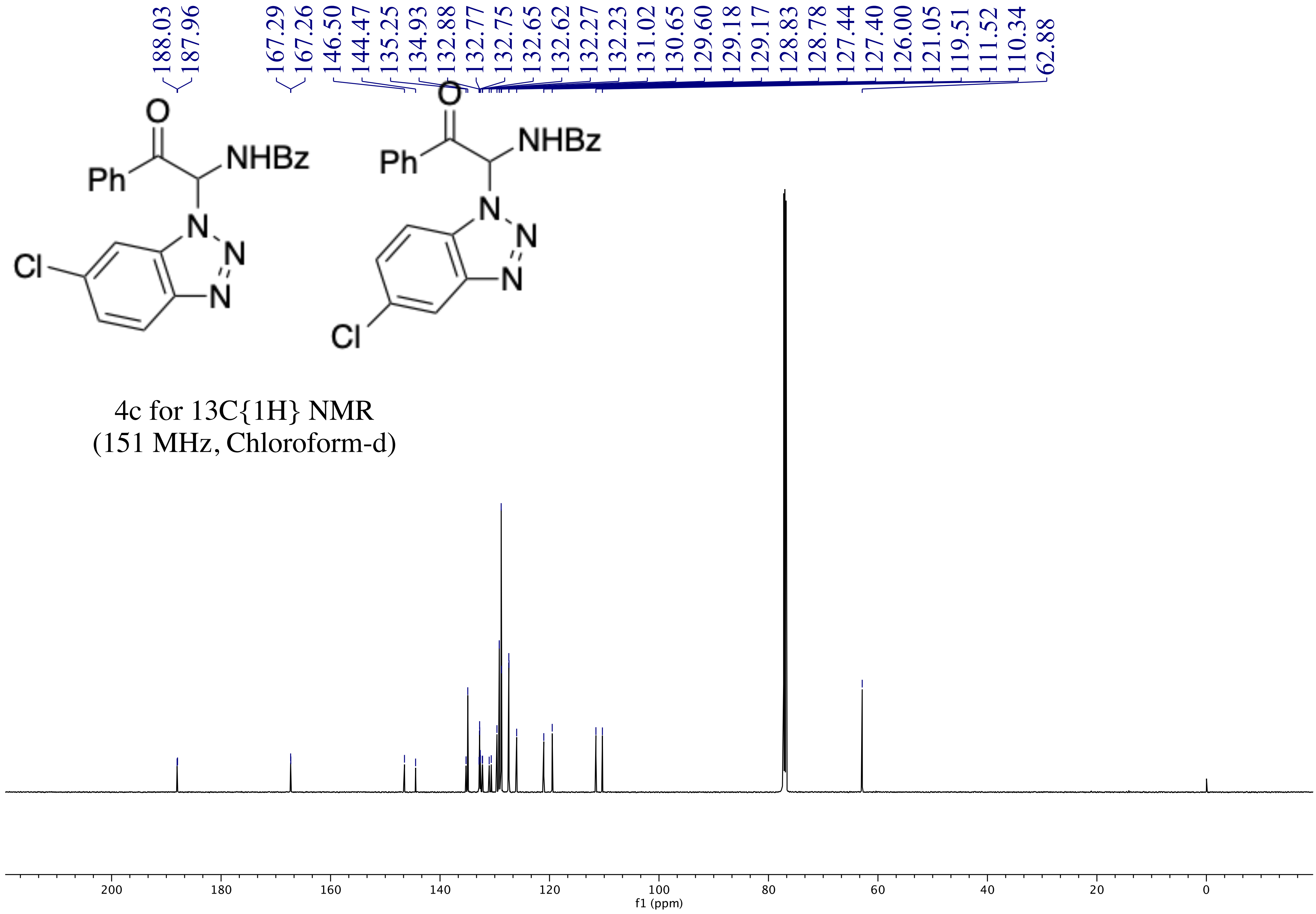
188.03
187.96
167.29
167.26
146.50
144.47
135.25
134.93
132.88
132.77
132.75
132.65
132.62
132.27
132.23
131.02
130.65
129.60
129.18
129.17
128.83
128.78
127.44
127.40
126.00
121.05
119.51
111.52
110.34
62.88
Ph
NHBz
Cl
N
4c for 13C{1H} NMR
(151 MHz, Chloroform-d)
200
180
160
140
120
100
80
60
40
20
0
f1 (ppm)

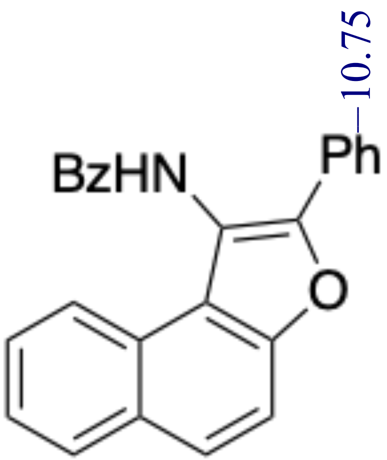

4d for 1H NMR
(600 MHz, DMSO-d)

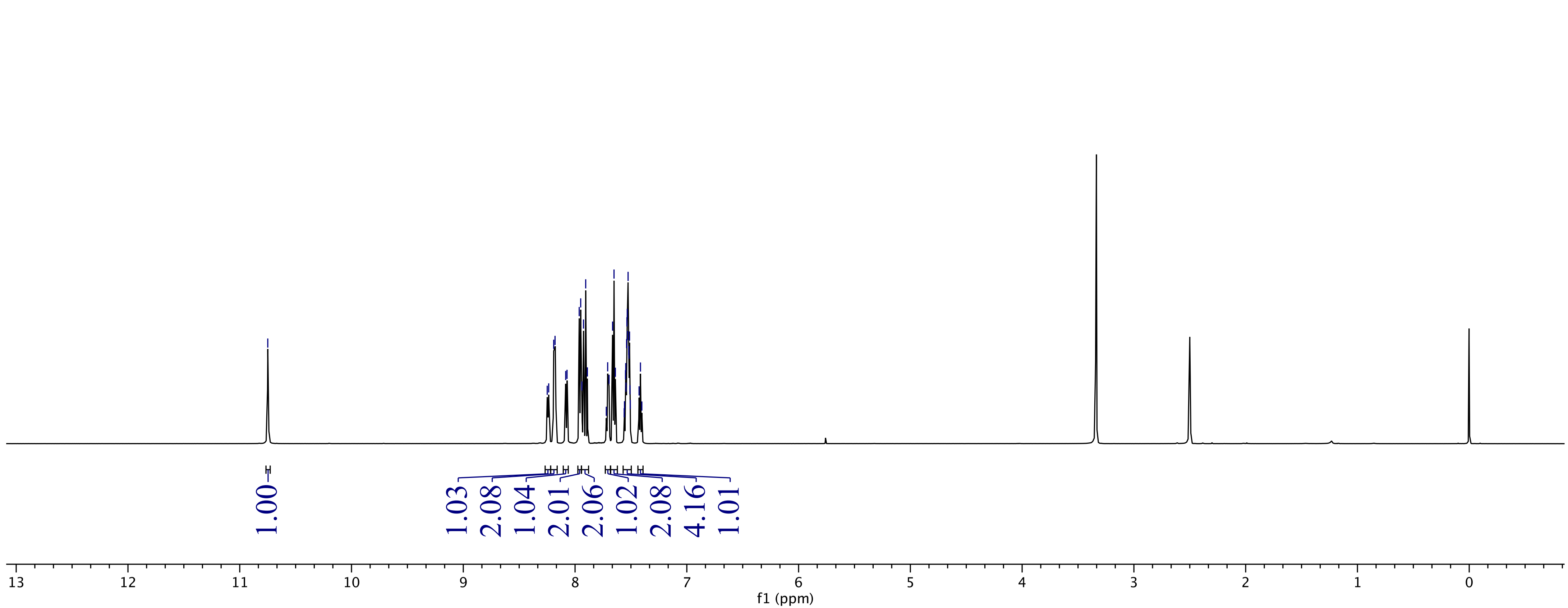

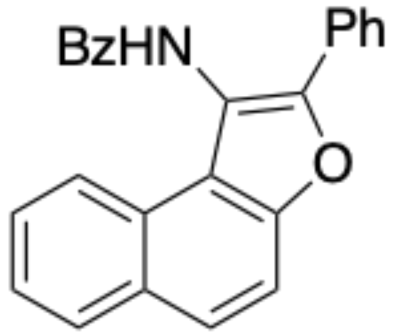

4d for 13C{1H} NMR
(151 MHz, DMSO-d)

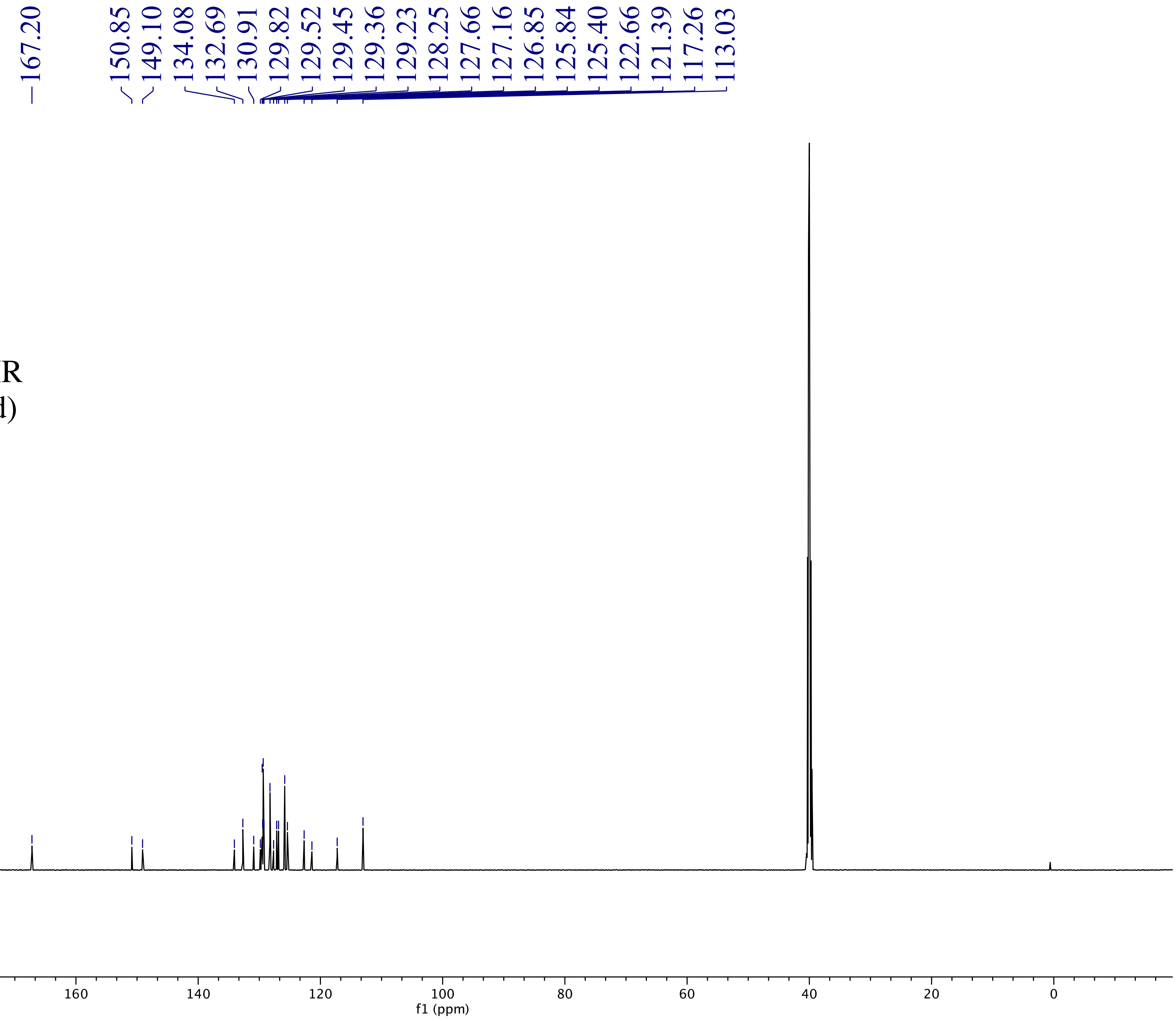

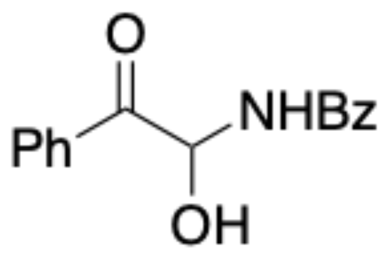

5 for 1H NMR
(600 MHz, DMSO-d)

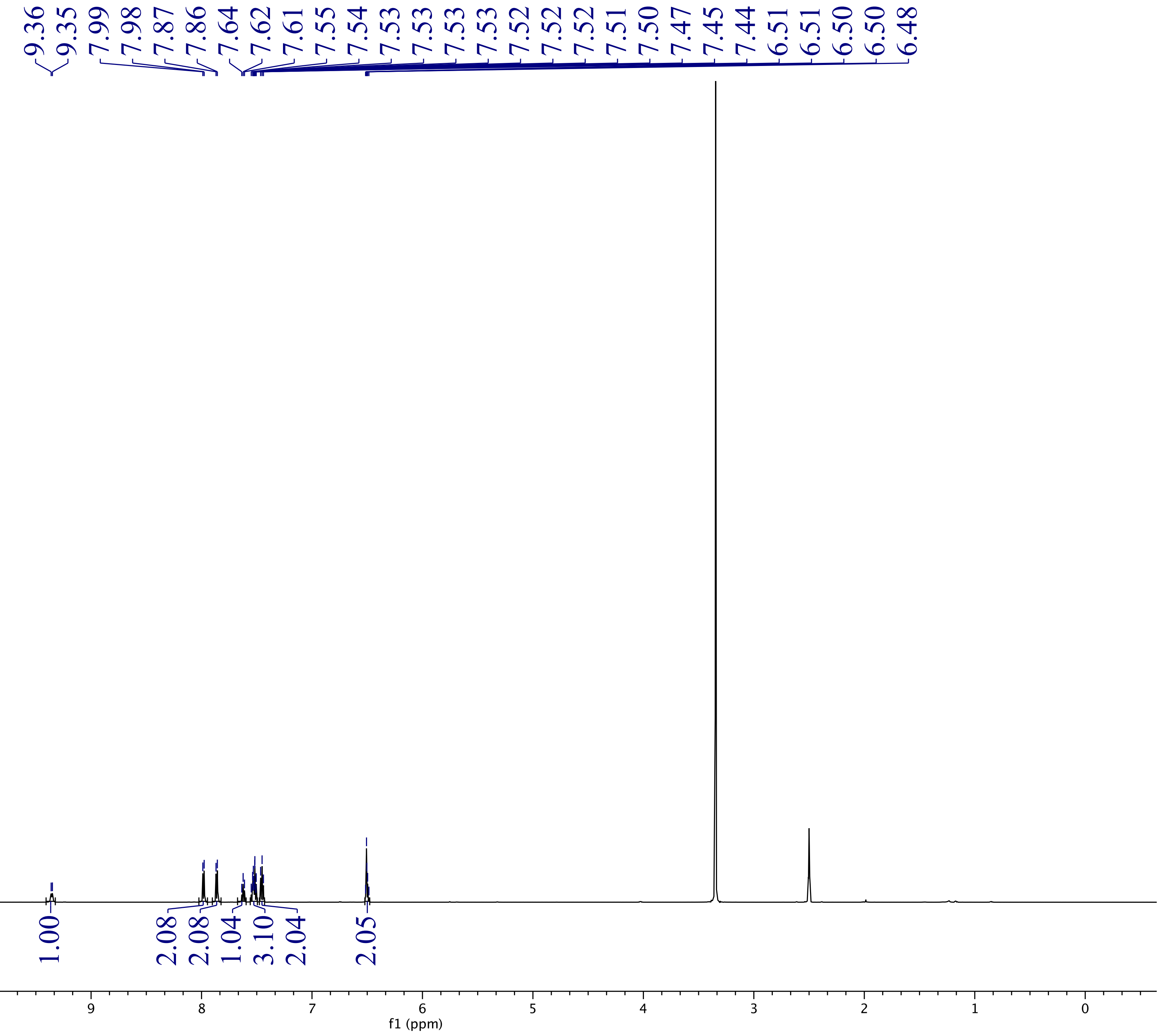

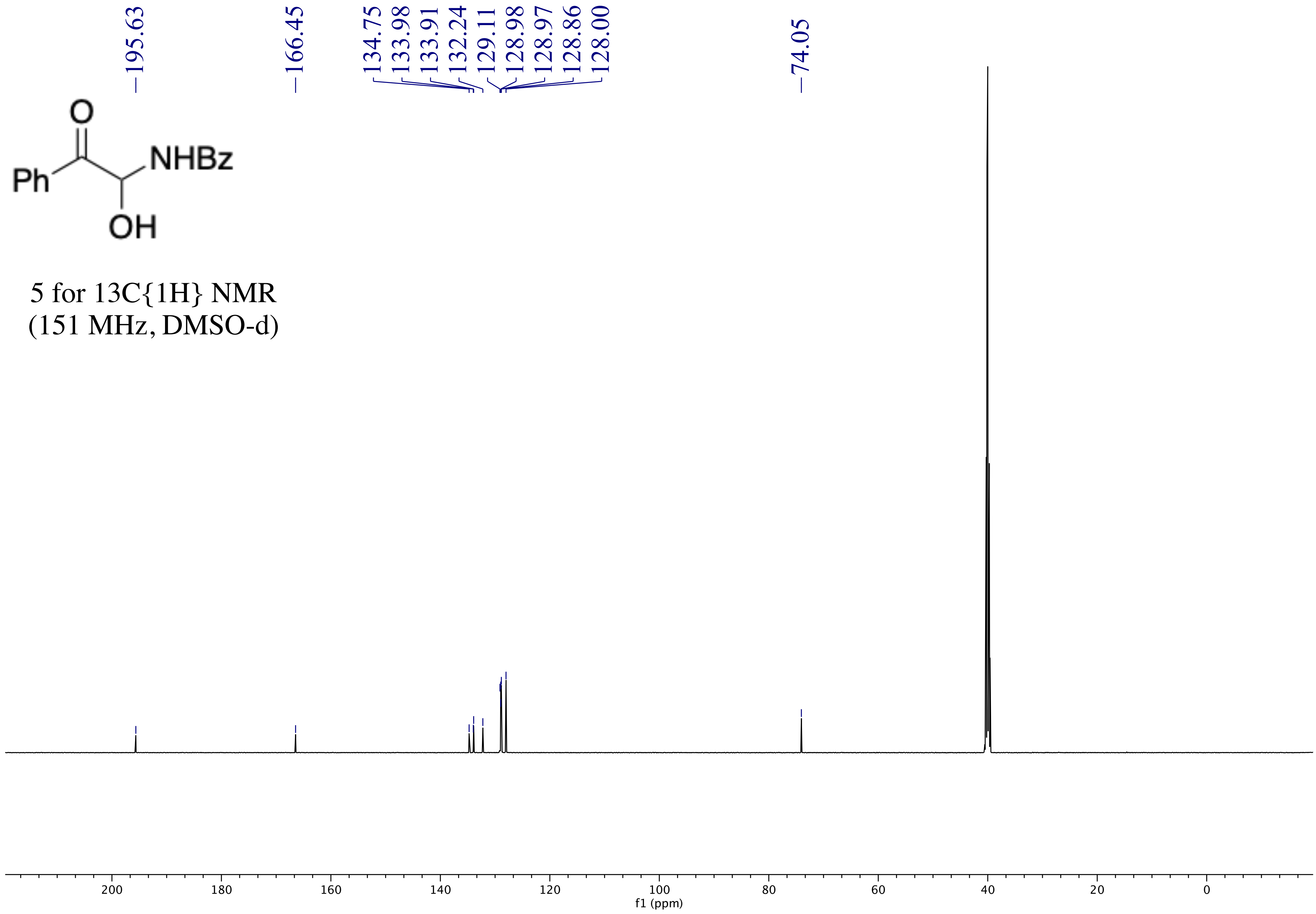

5 for 13C{1H} NMR
(151 MHz, DMSO-d)

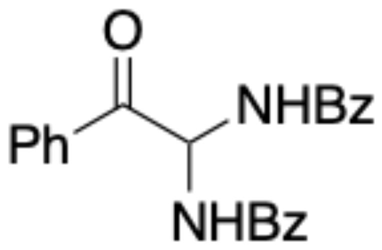

6 for 1H NMR
(600 MHz, DMSO-d)

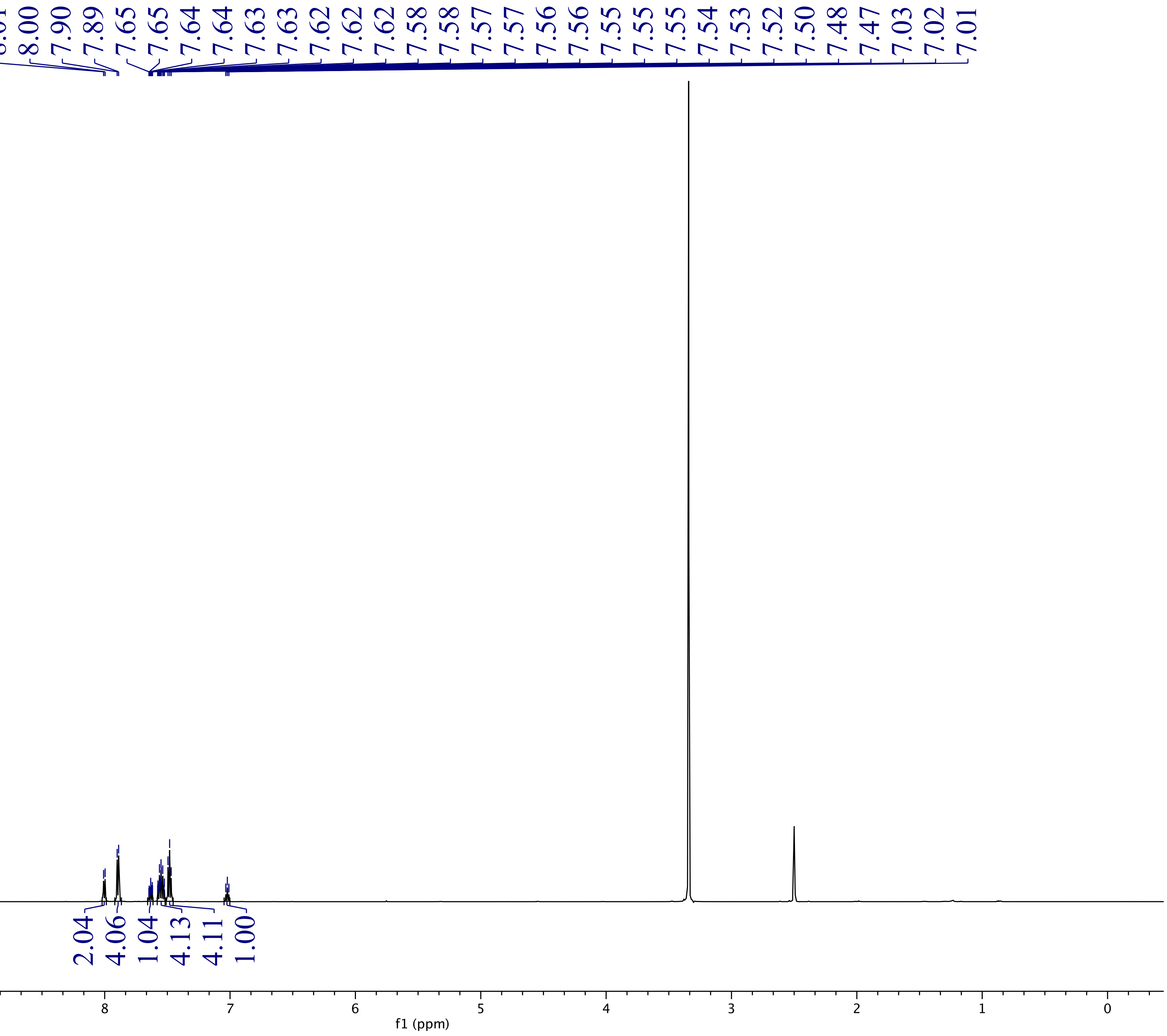

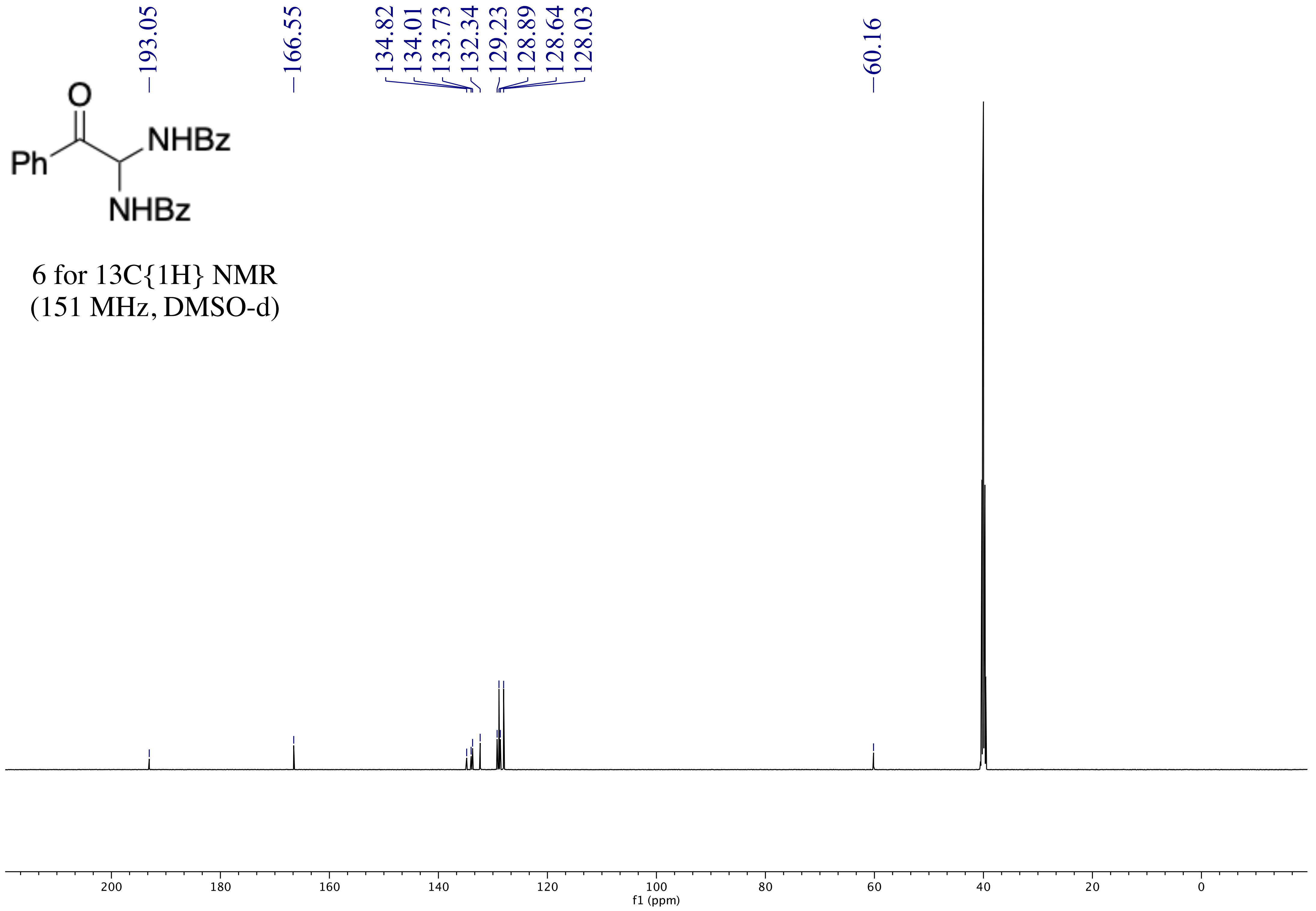
193.05
166.55
134.82
134.01
133.73
132.34
129.23
128.89
128.64
128.03
60.16
Ph
O
NHBz
NHBz
6 for 13C{1H} NMR
(151 MHz, DMSO-d)
200
180
160
140
120
100
80
60
40
20
0
f1 (ppm)

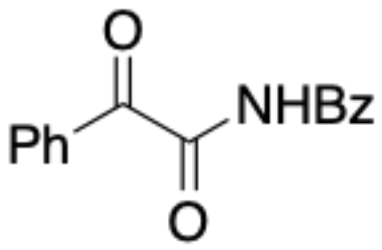

7 for 1H NMR
(600 MHz, Chloroform-d)

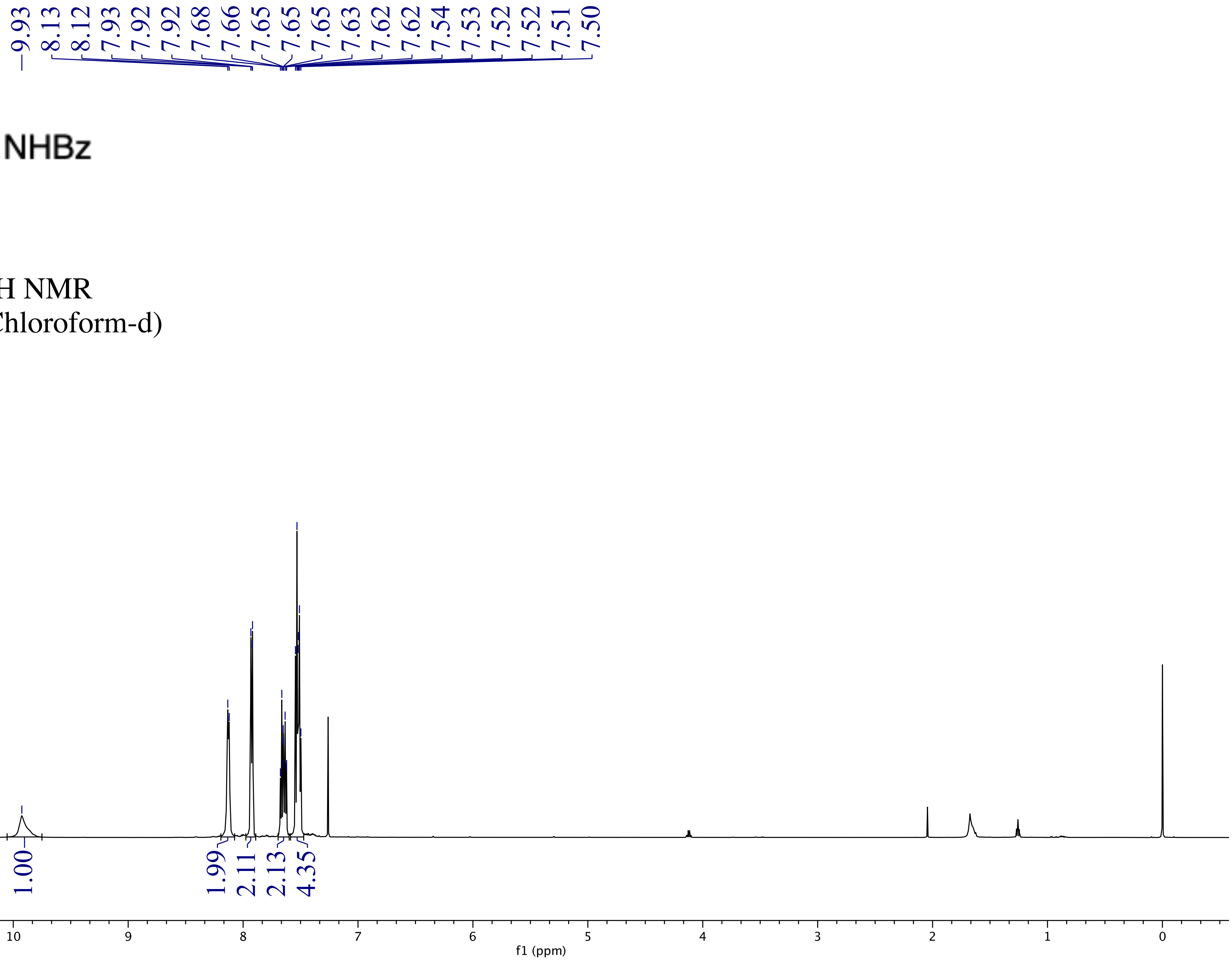

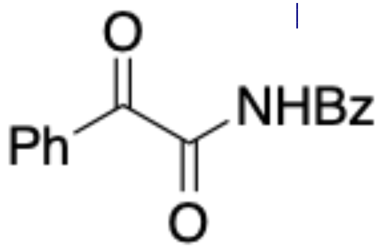

7 for 13C{1H} NMR
(151 MHz, Chloroform-d)

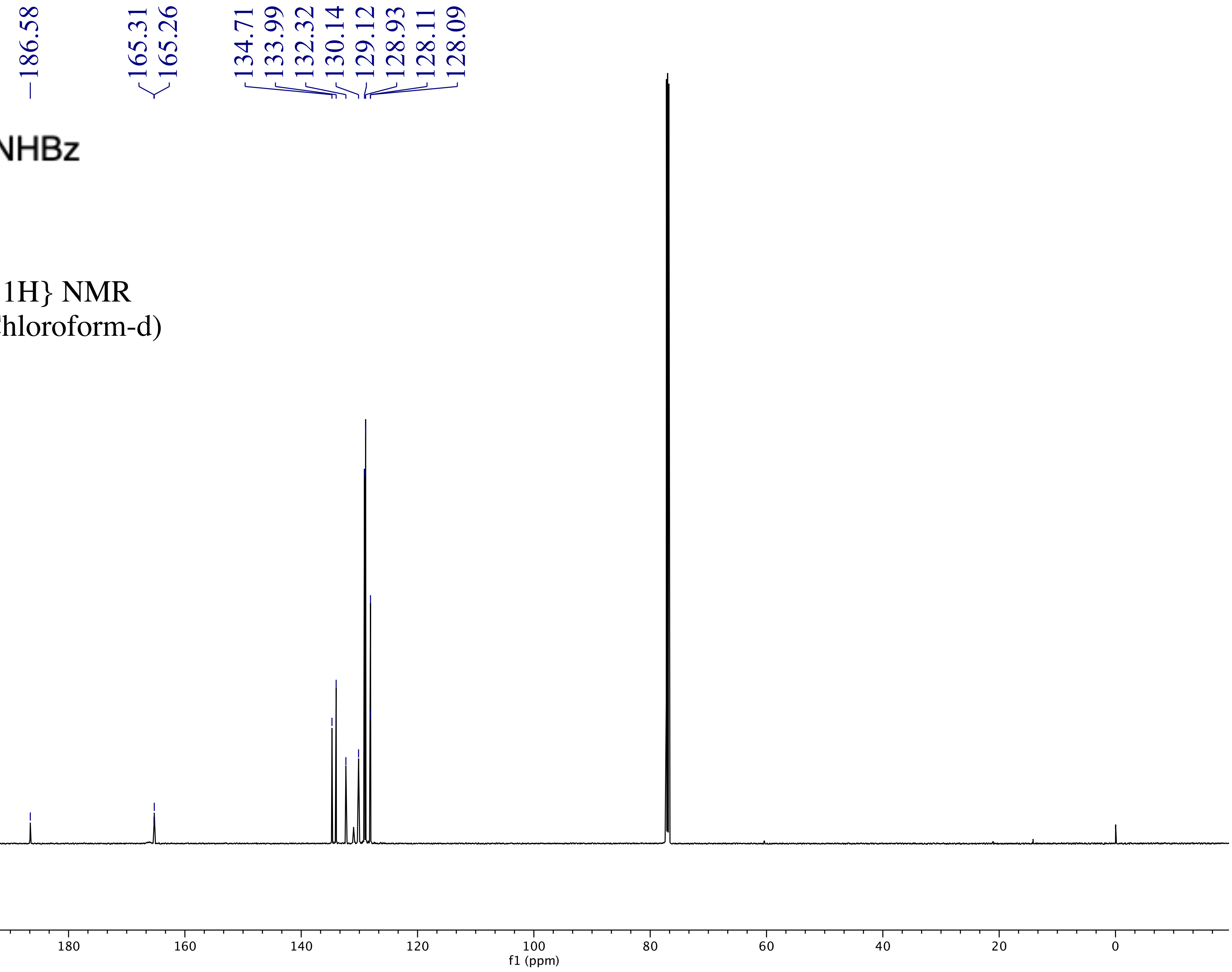

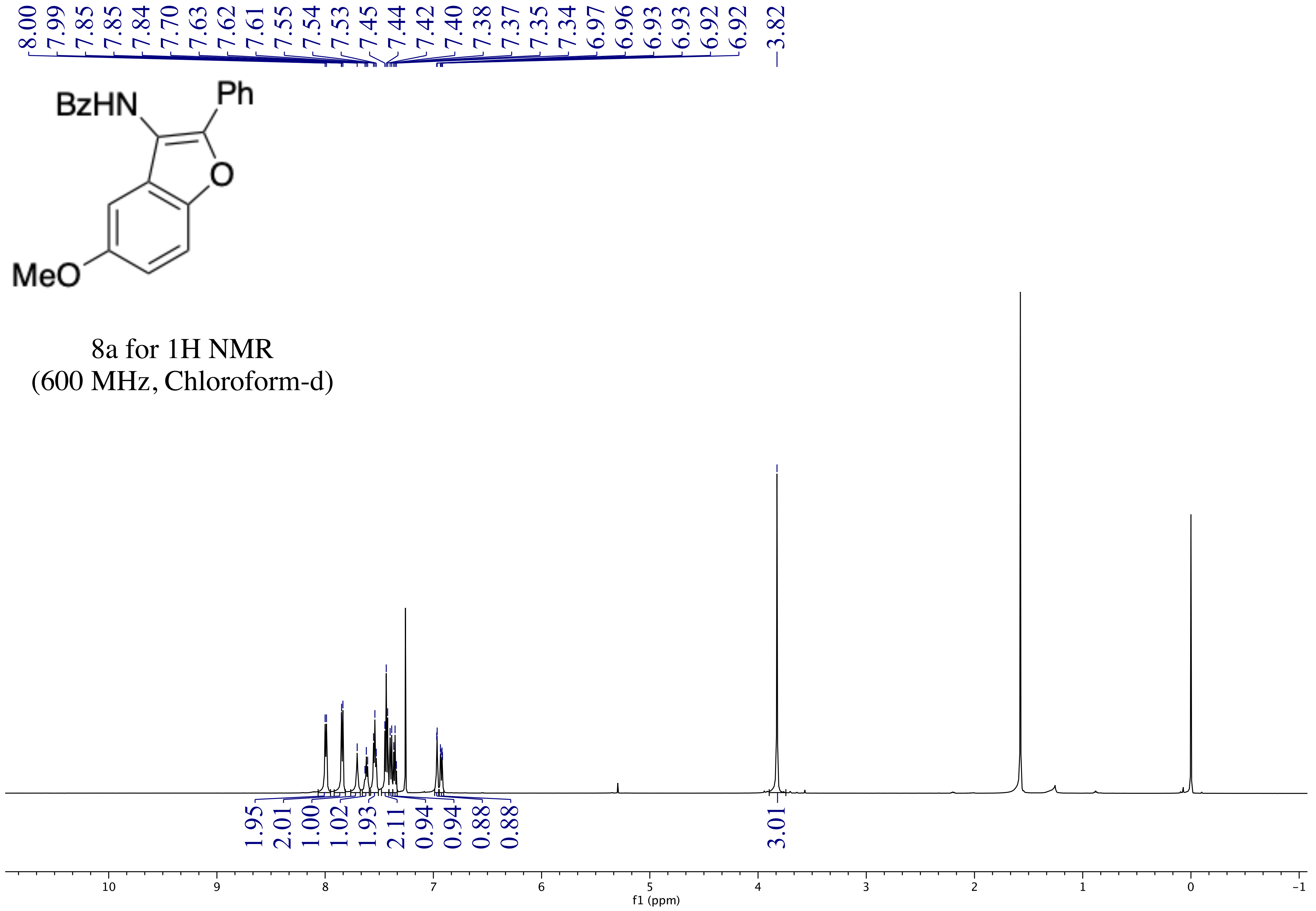

8a for 1H NMR
(600 MHz, Chloroform-d)

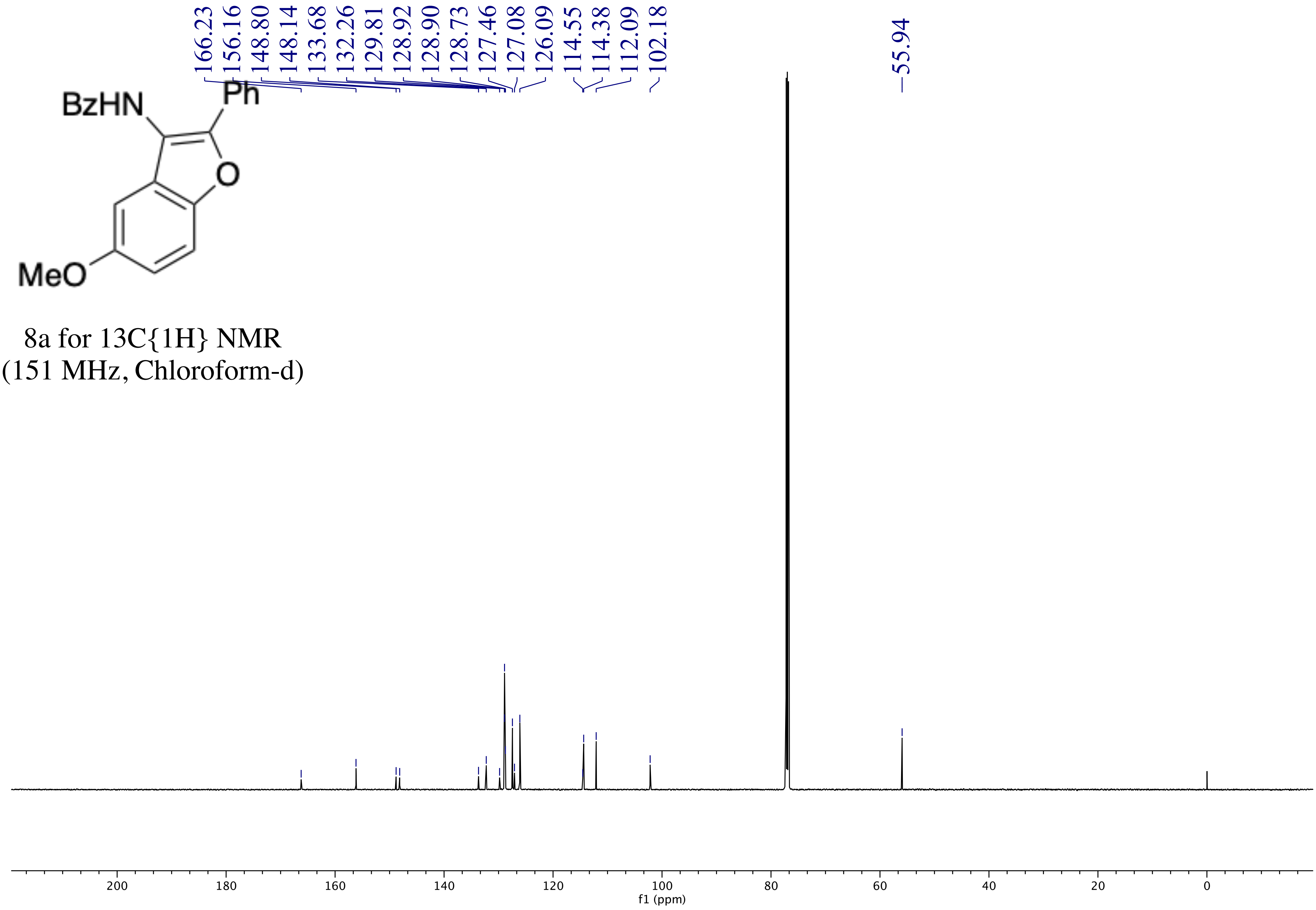
BzHN
Ph
O
MeO
8a for 13C{1H} NMR
(151 MHz, Chloroform-d)
166.23
156.16
148.80
148.14
133.68
132.26
129.81
128.92
128.90
128.73
127.46
127.08
126.09
114.55
114.38
112.09
102.18
55.94
200
180
160
140
120
100
80
60
40
20
0
f1 (ppm)

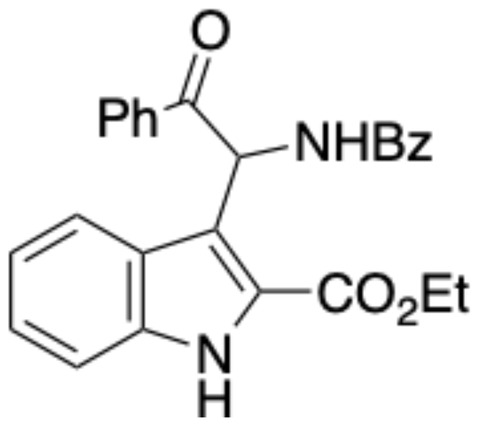

8b for 1H NMR
(600 MHz, Chloroform-d)

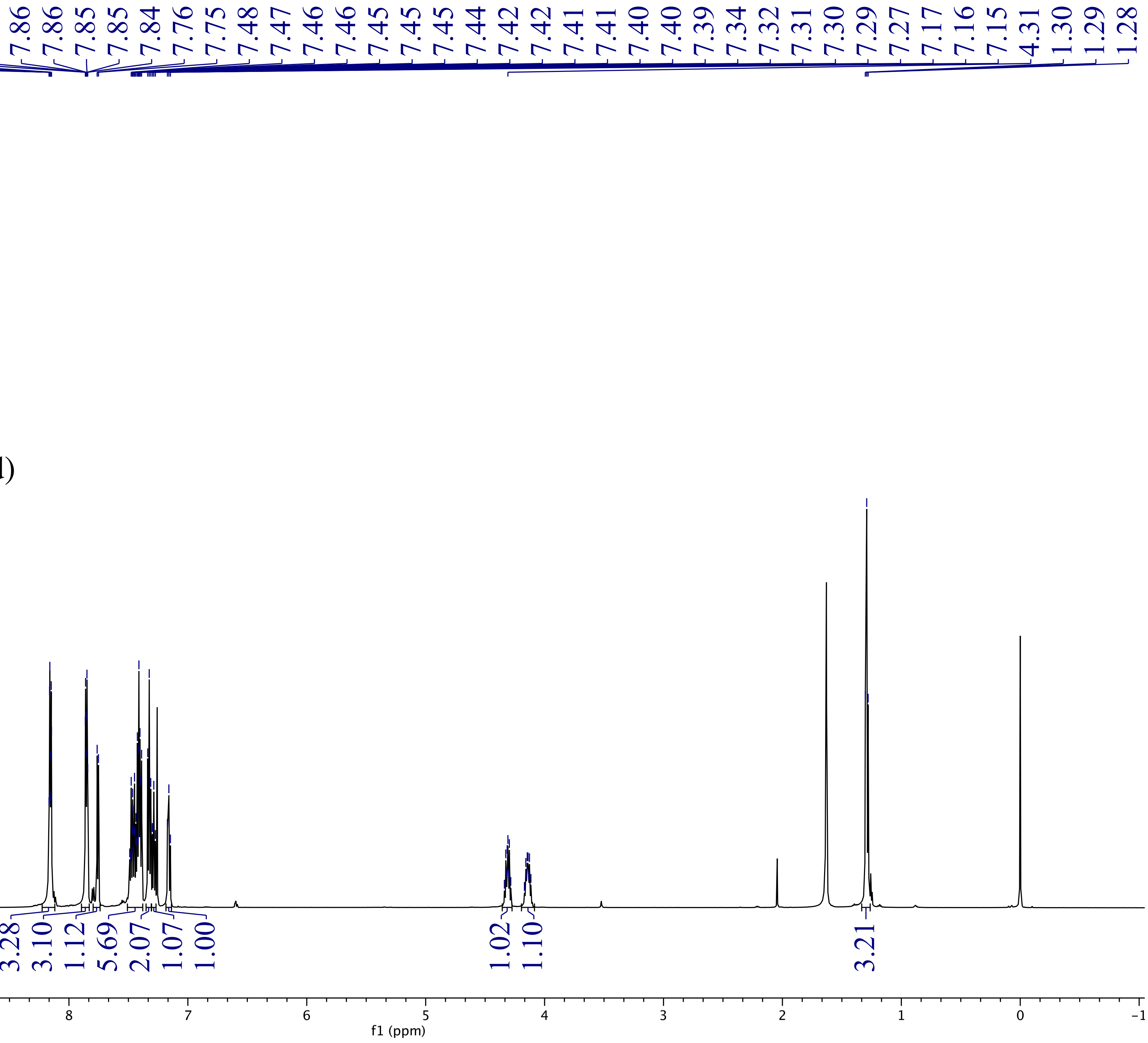

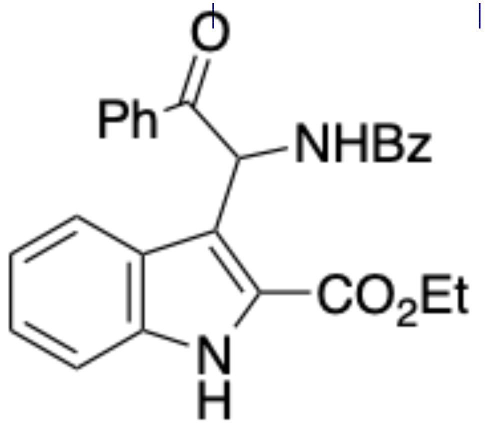

8b for 13C{1H} NMR
(151 MHz, Chloroform-d)

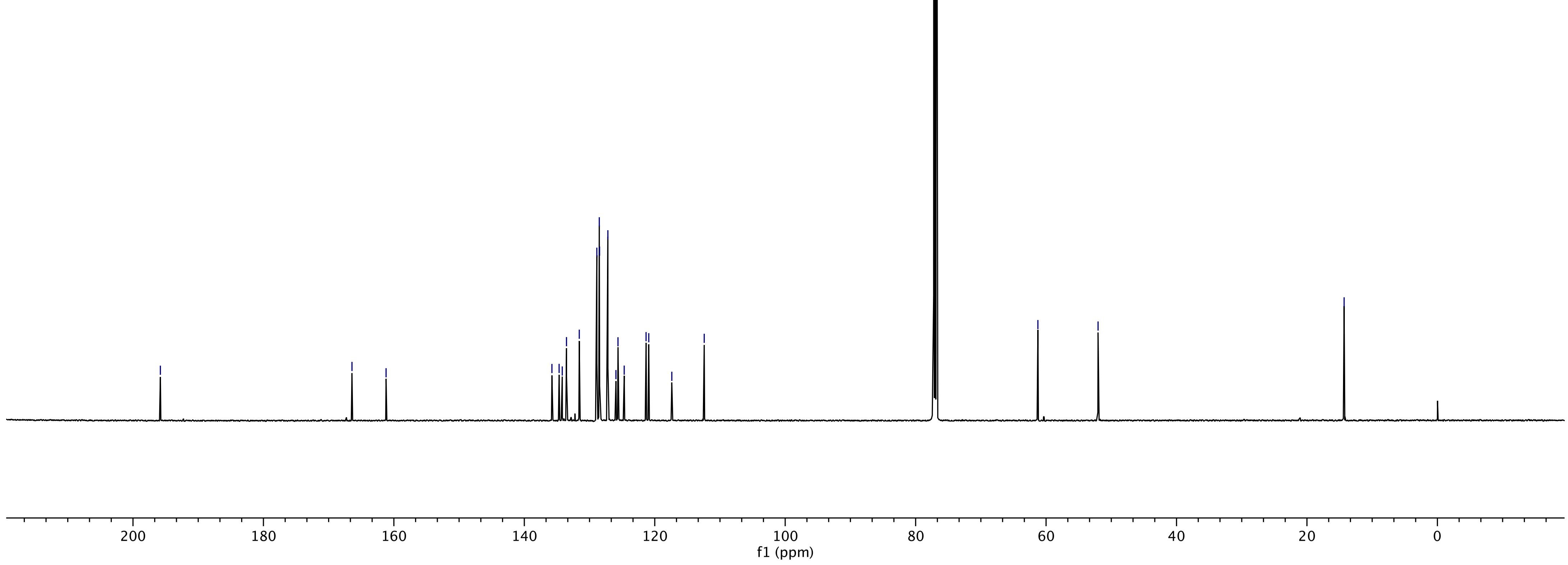

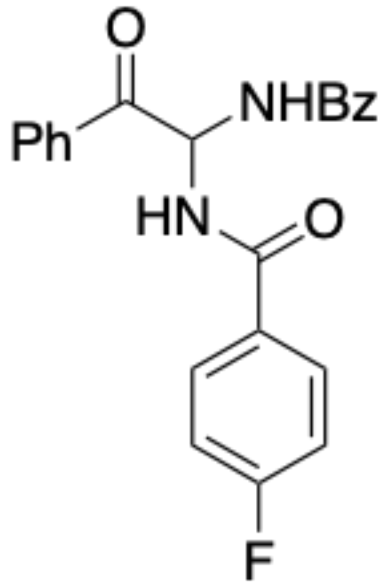

8c for 1H NMR
(600 MHz, DMSO-d)

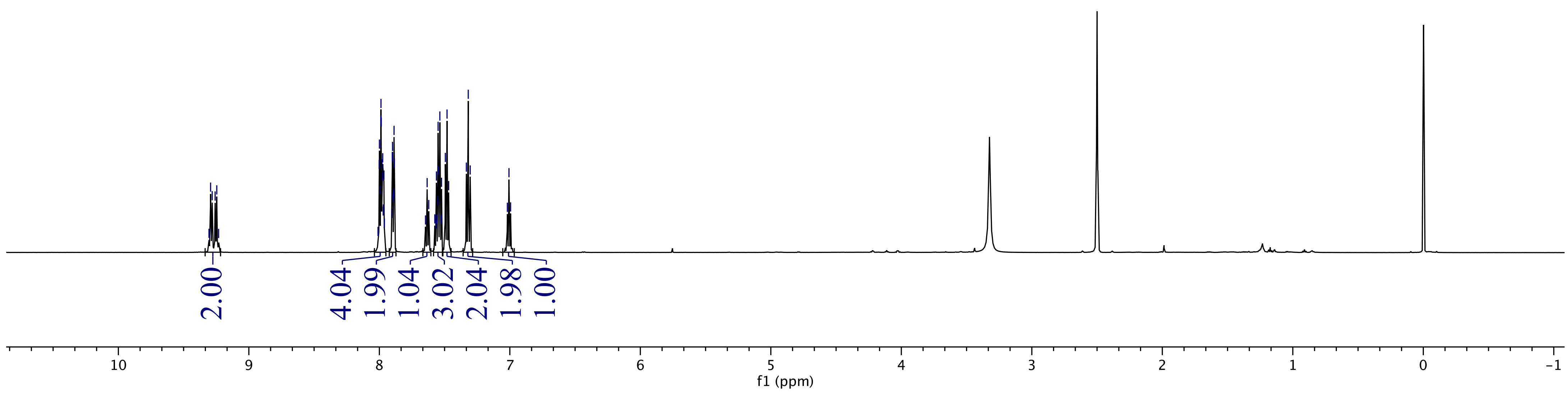

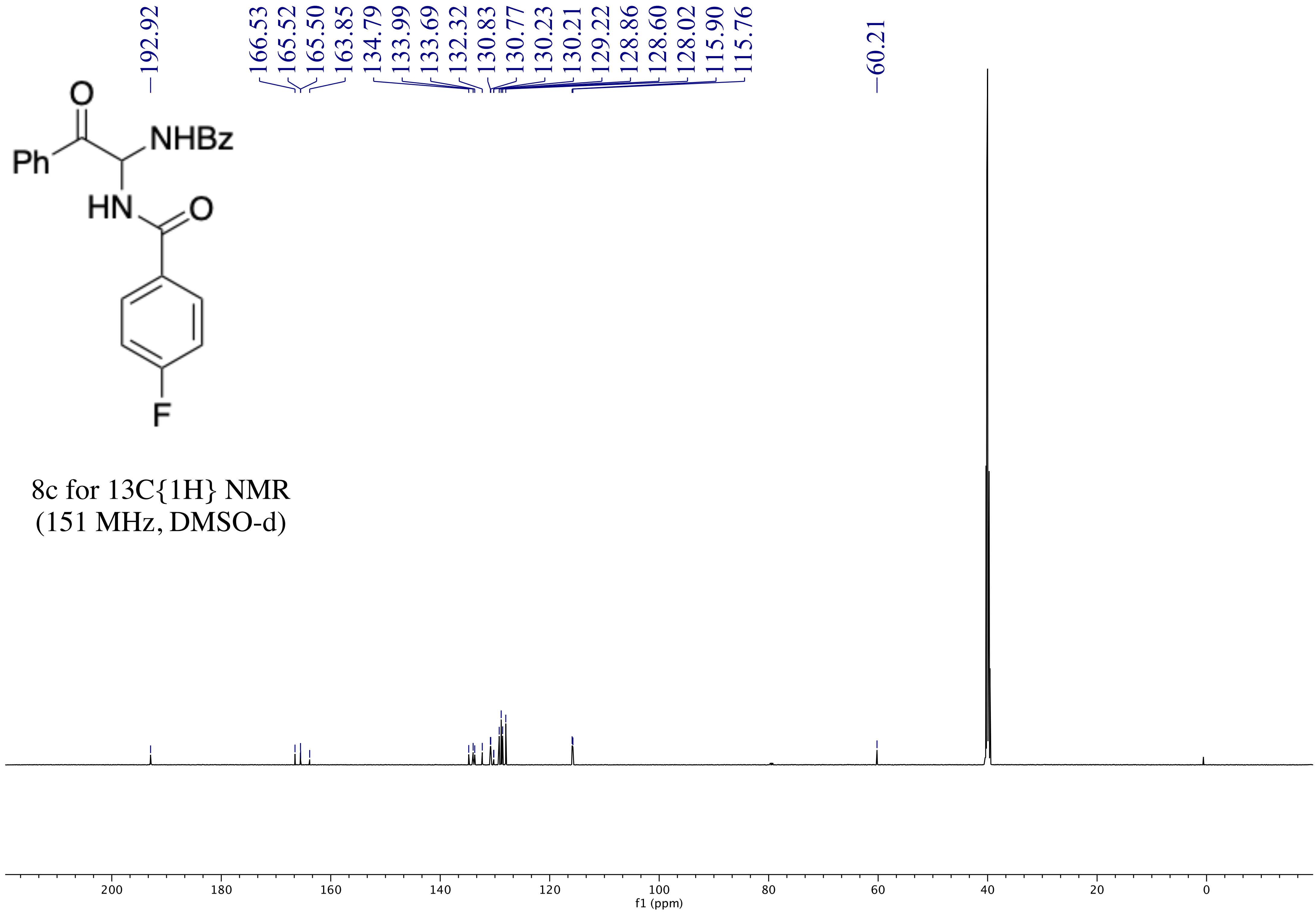

8c for 13C{1H} NMR
(151 MHz, DMSO-d)

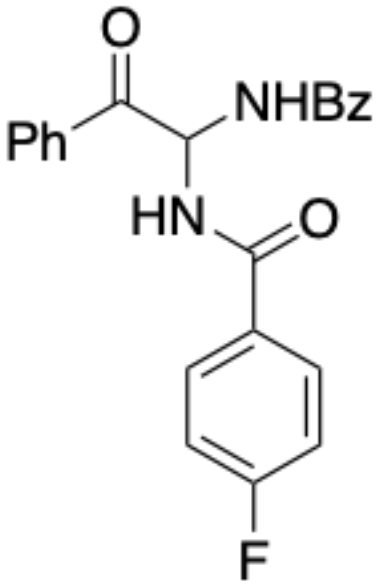

8c for ^{19}F NMR
(376 MHz, DMSO-d)

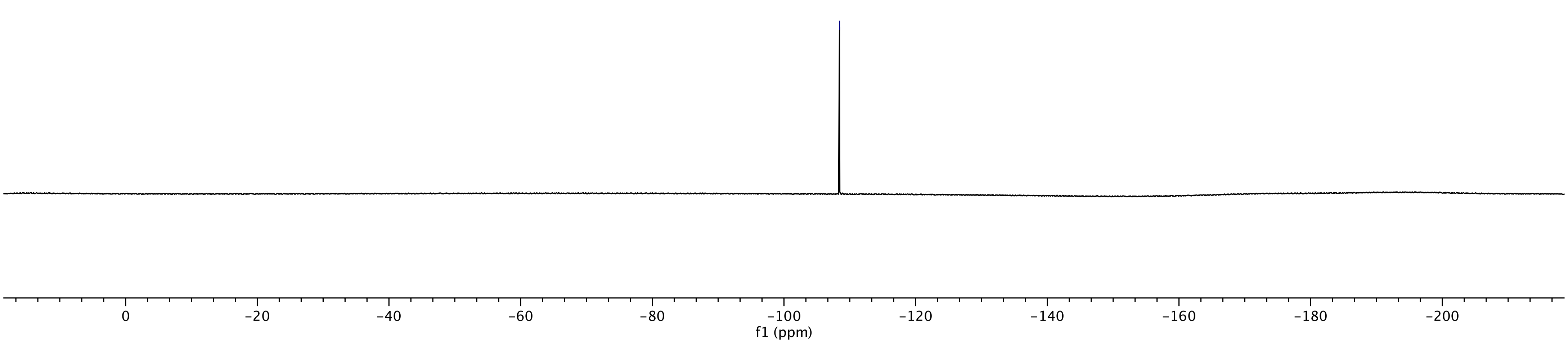

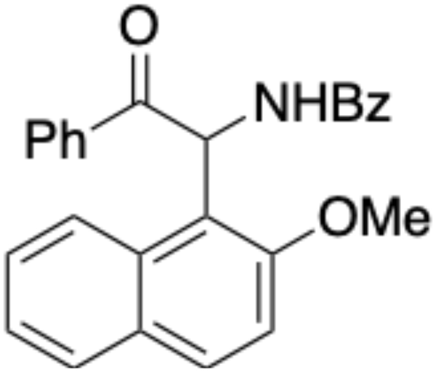

8d for 1H NMR
(400 MHz, Chloroform-d)

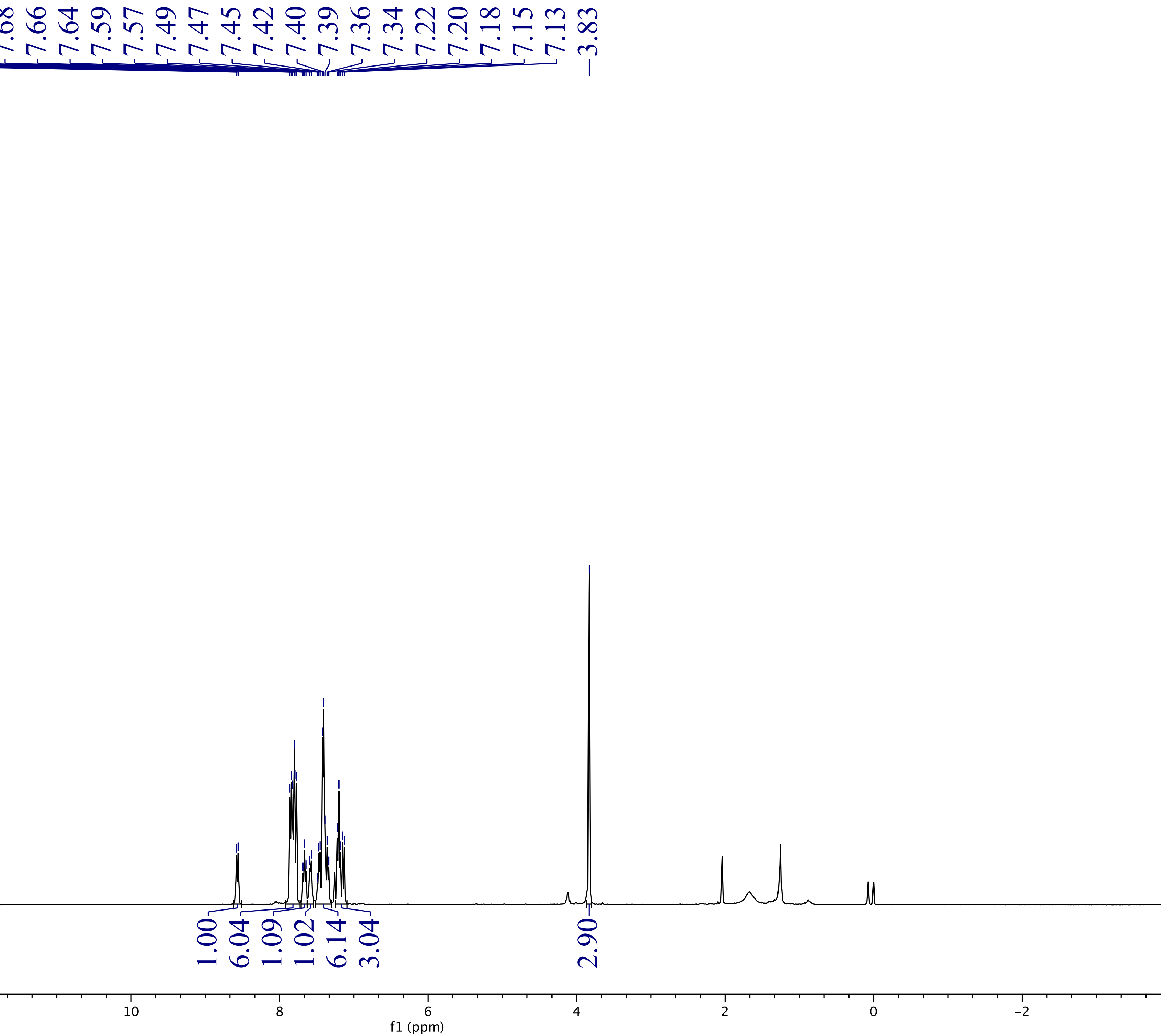

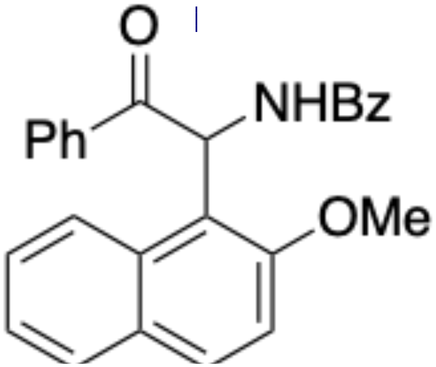

8d for 13C{1H} NMR
(101 MHz, Chloroform-d)

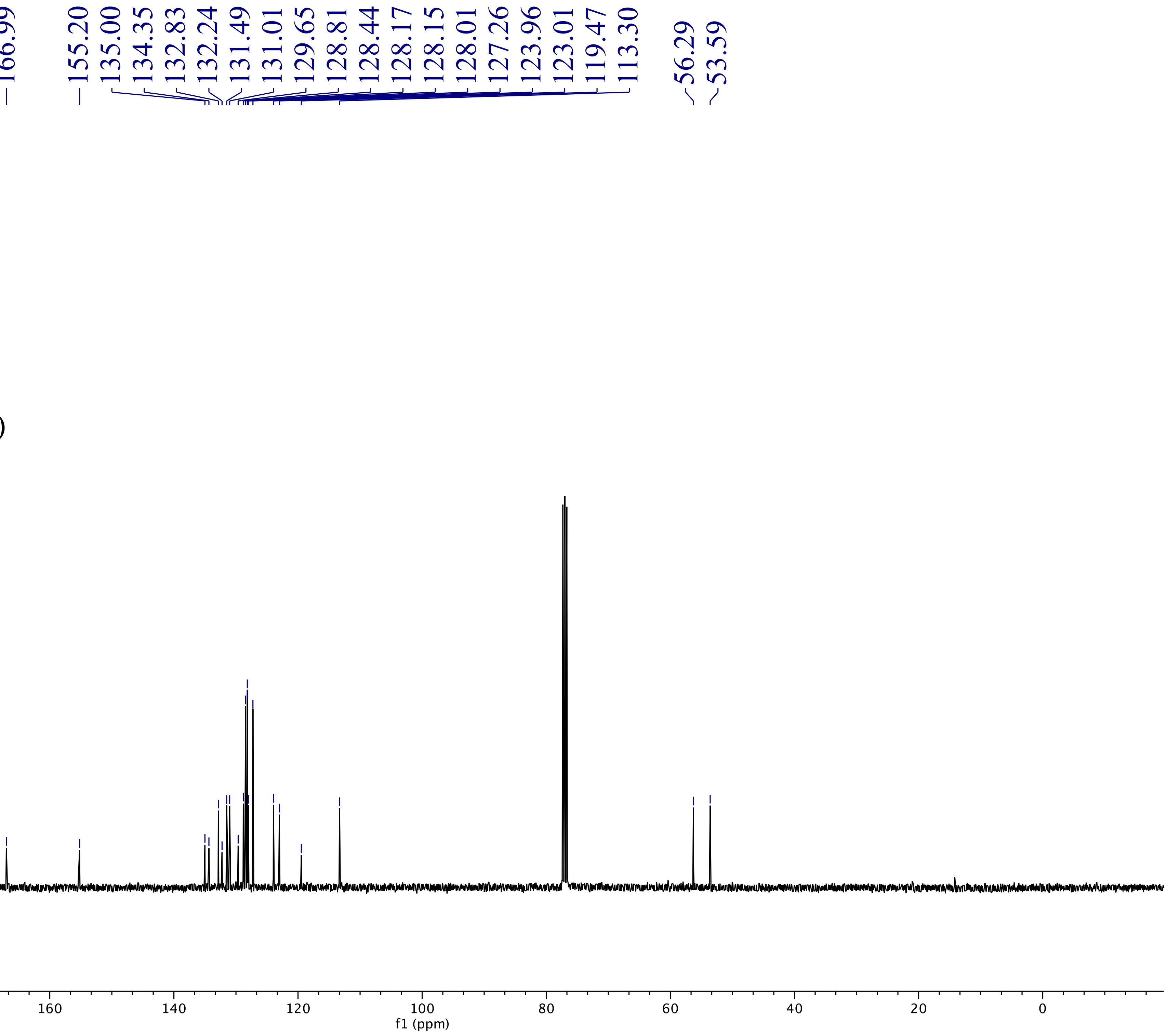

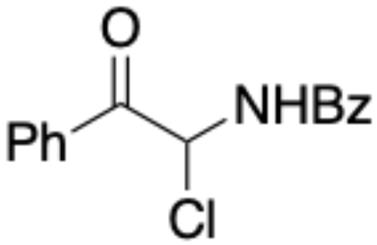

8e for 1H NMR
(600 MHz, Chloroform-d)

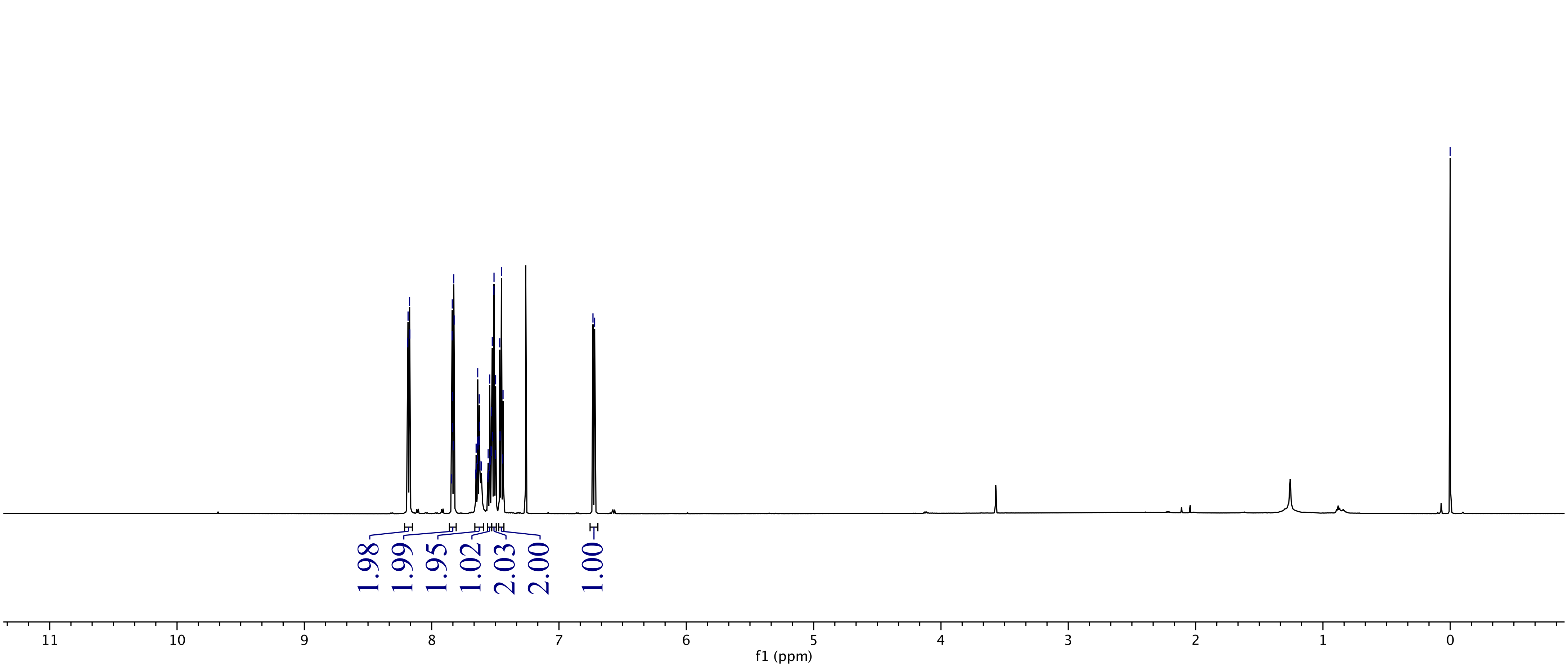

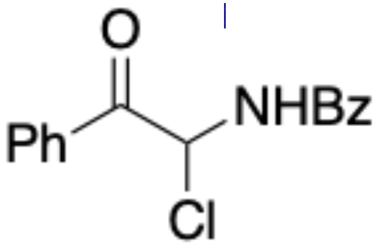

8e for 13C{1H} NMR
(151 MHz, Chloroform-d)

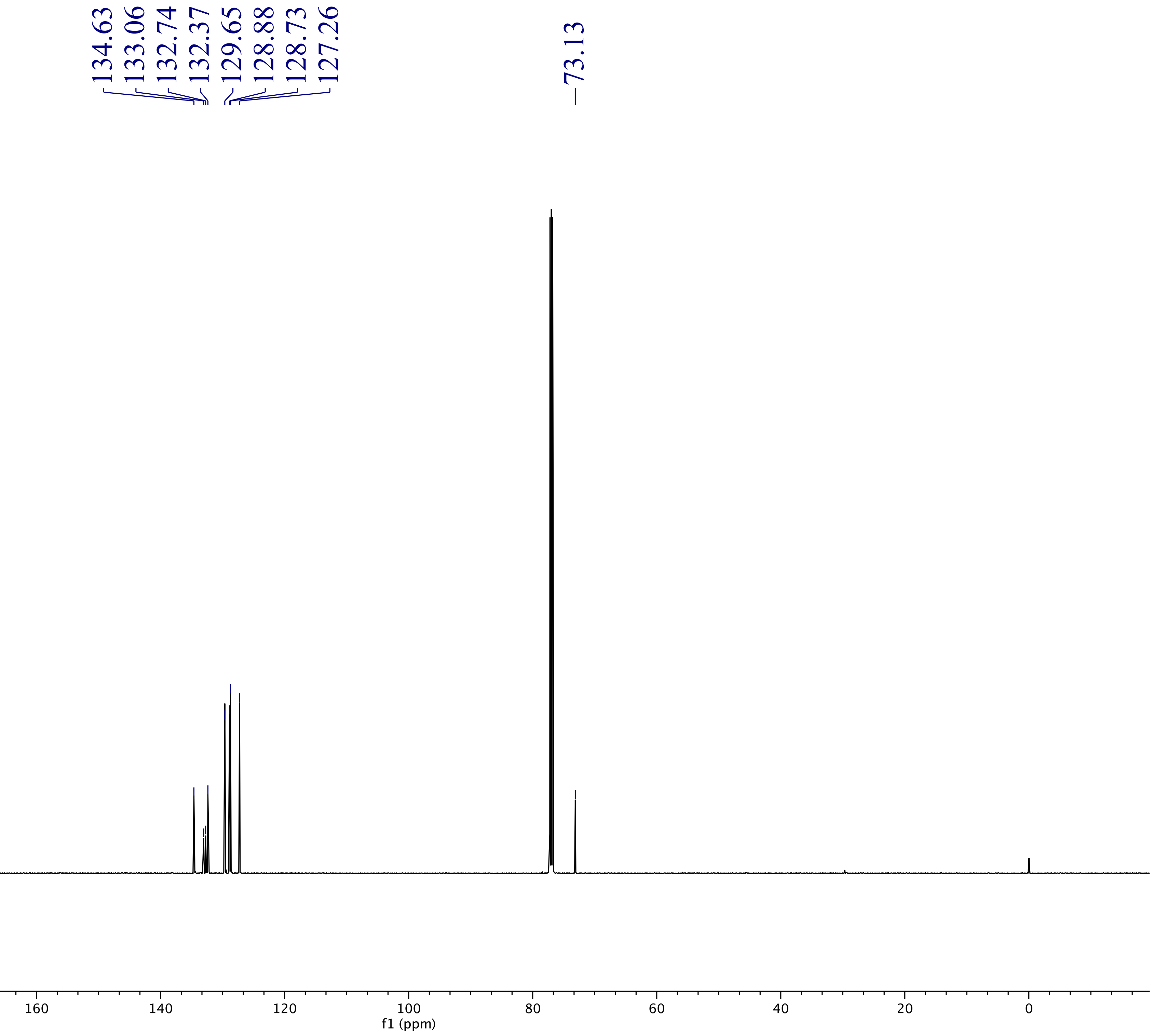

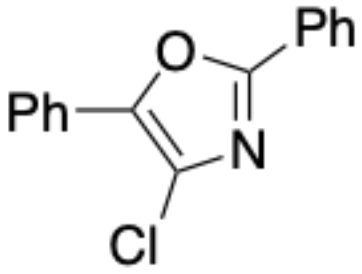

8f for 1H NMR
(600 MHz, Chloroform-d)

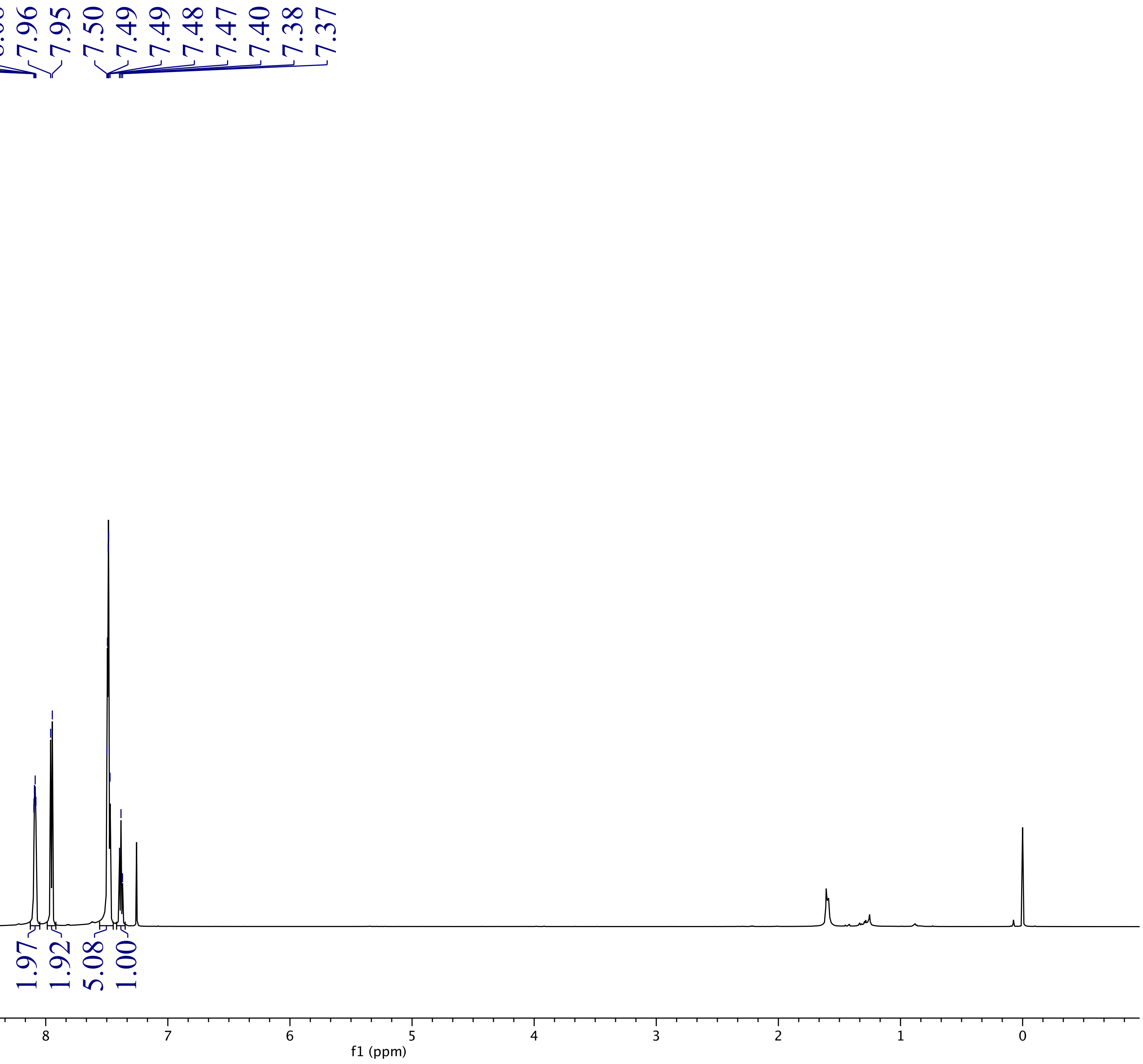

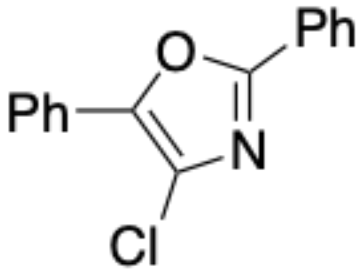

8f for 13C{1H} NMR
(151 MHz, Chloroform-d)

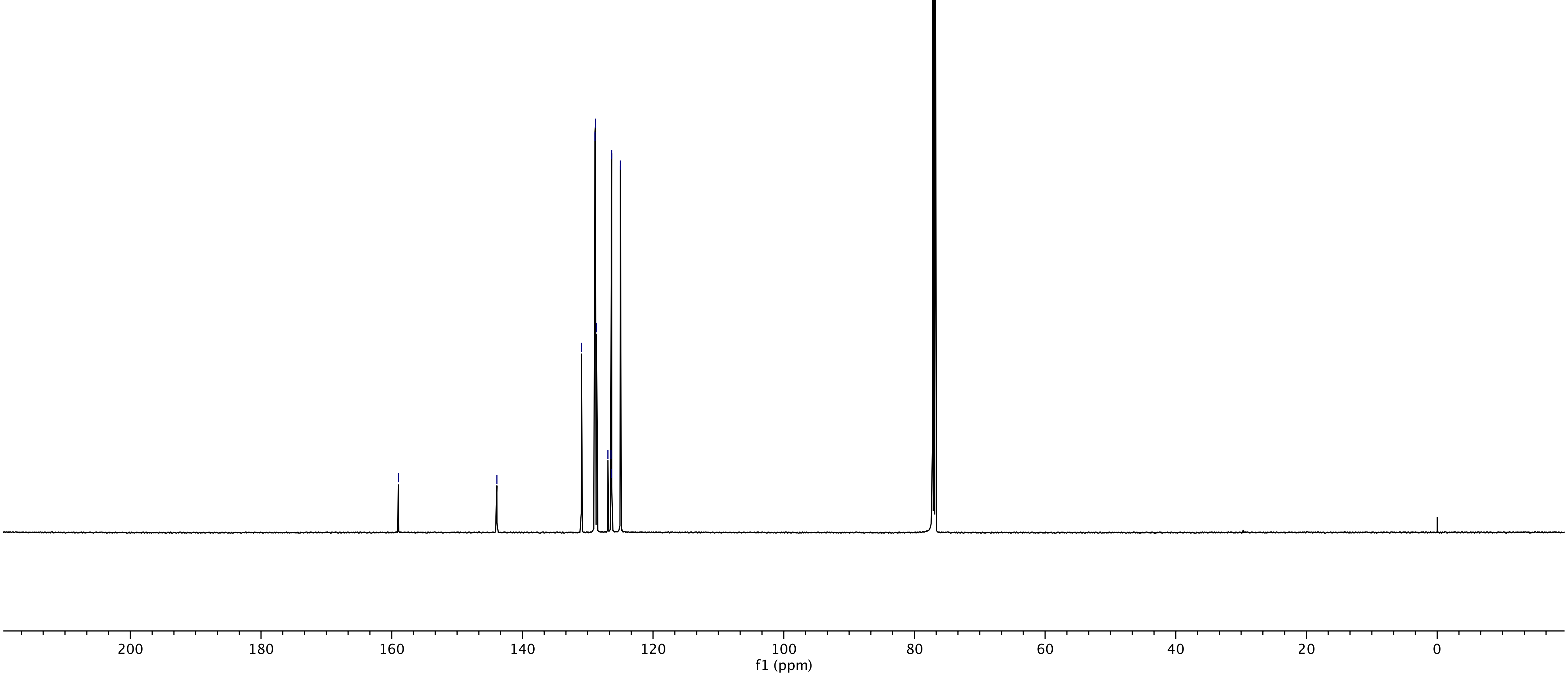

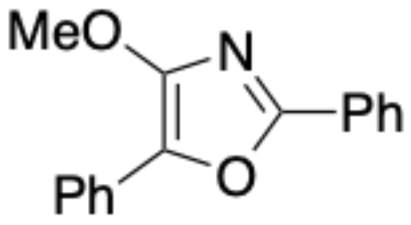

8g for 1H NMR
(600 MHz, Chloroform-d)

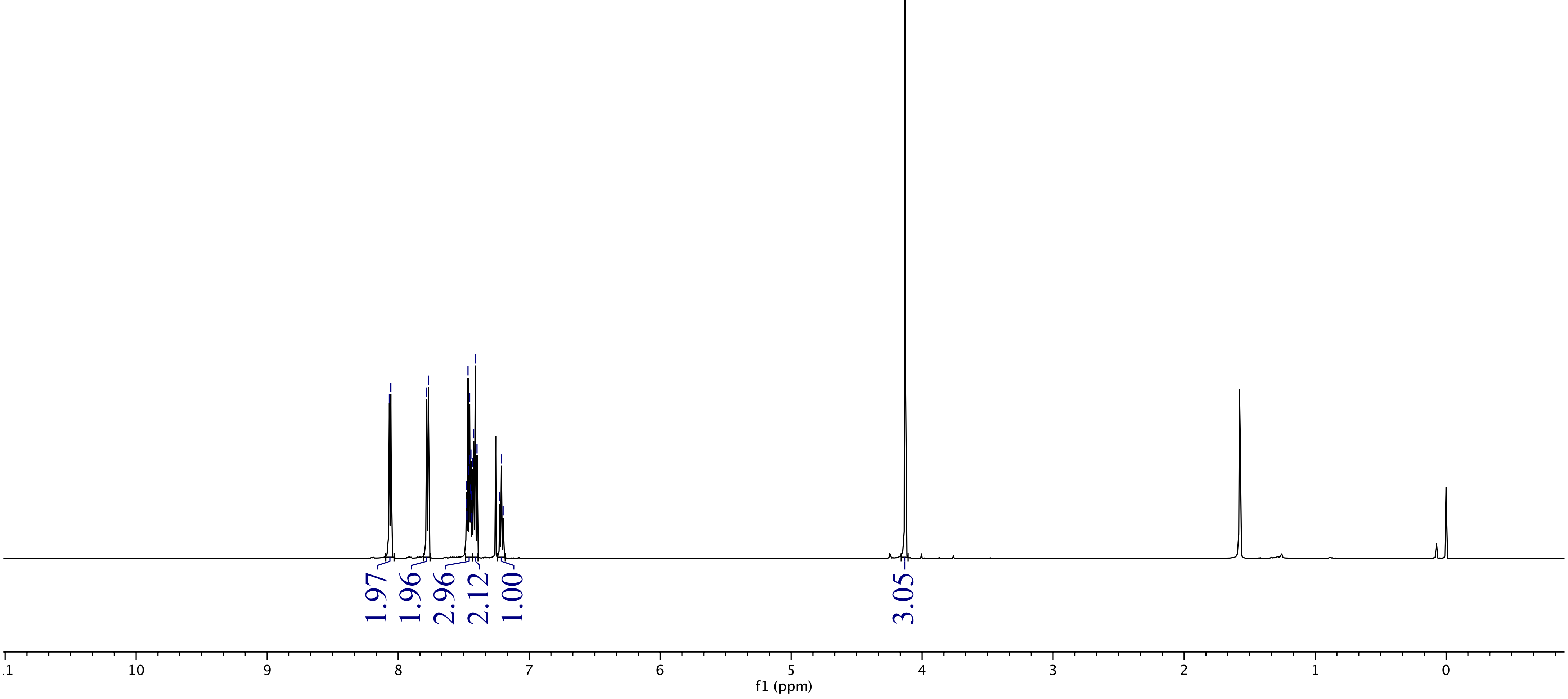

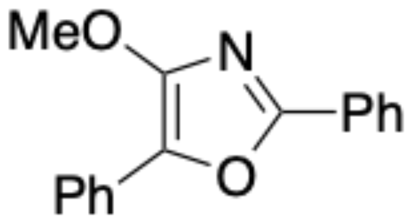

8g for 13C{1H} NMR
(151 MHz, Chloroform-d)

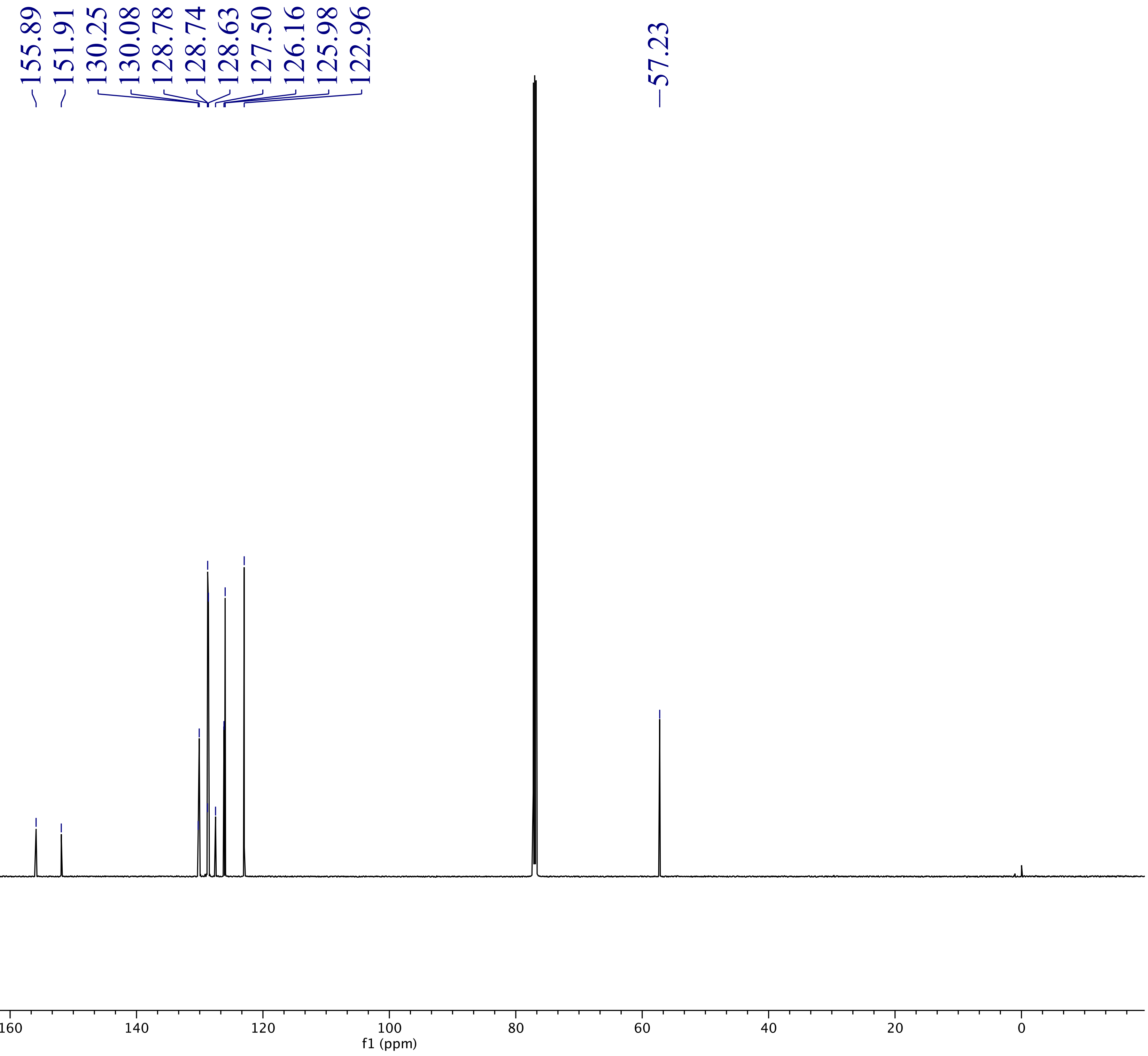

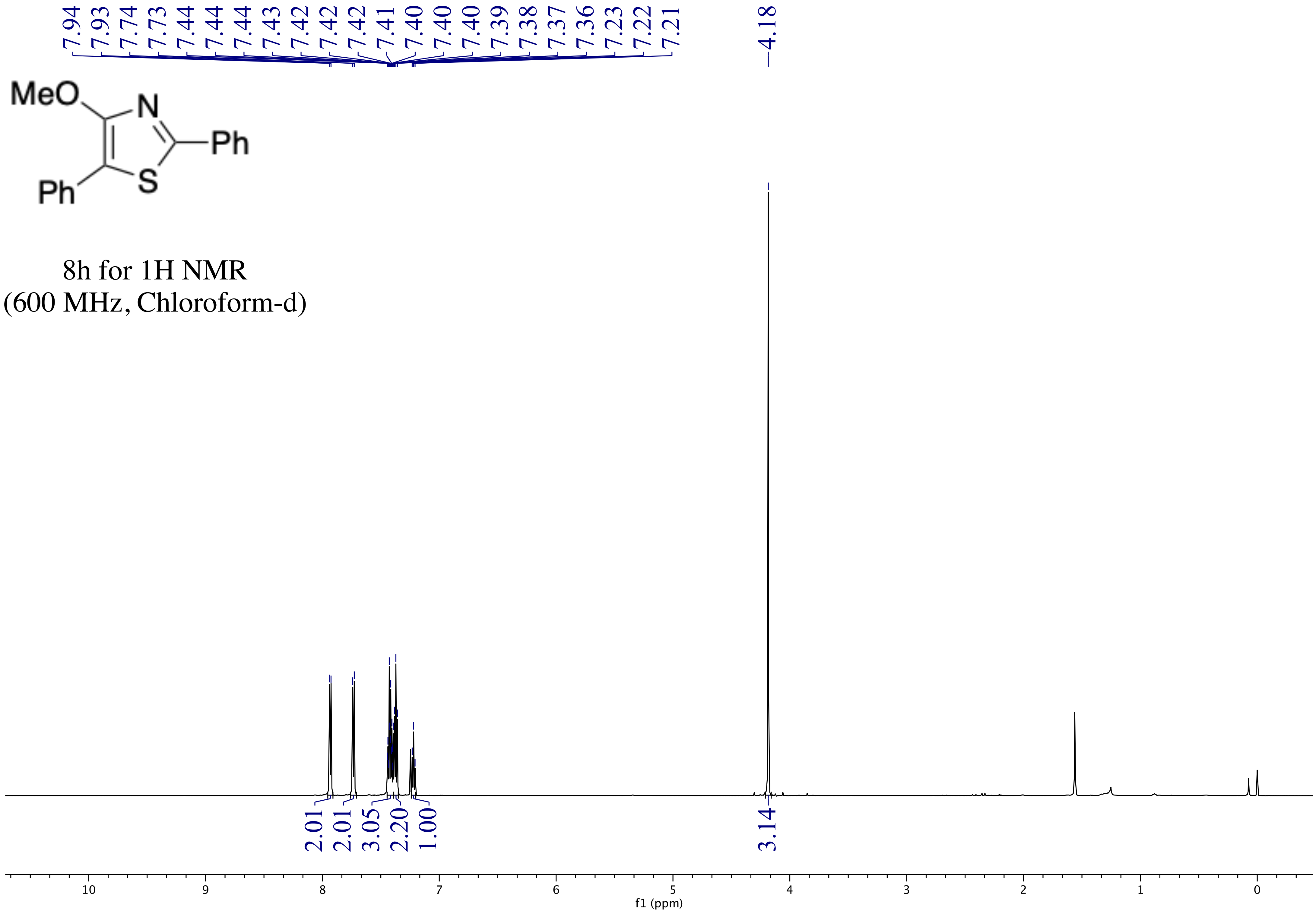

8h for 1H NMR
(600 MHz, Chloroform-d)

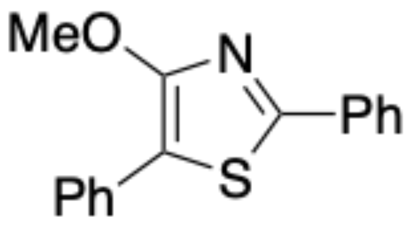

8h for 13C{1H} NMR
(151 MHz, Chloroform-d)

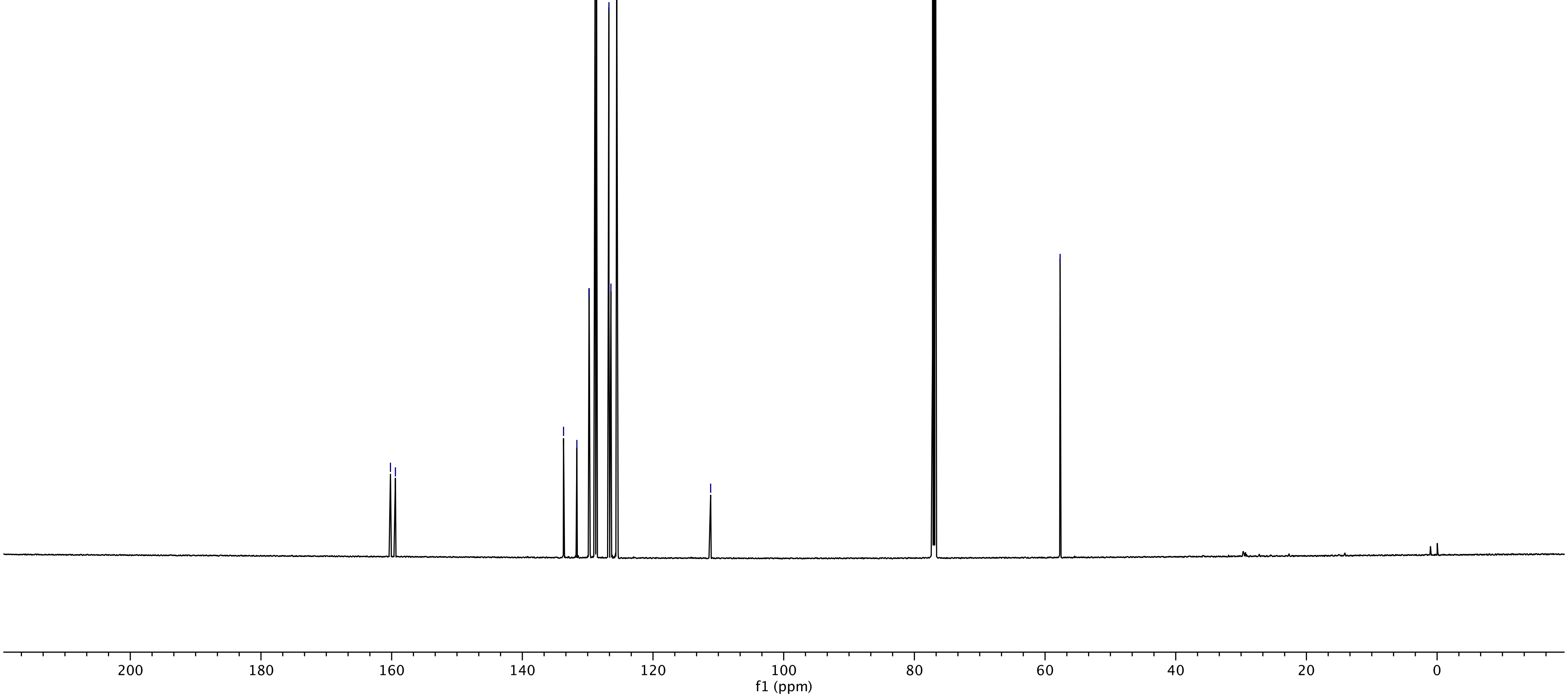

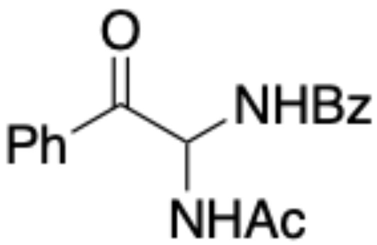

8i for 1H NMR
(600 MHz, DMSO-d)

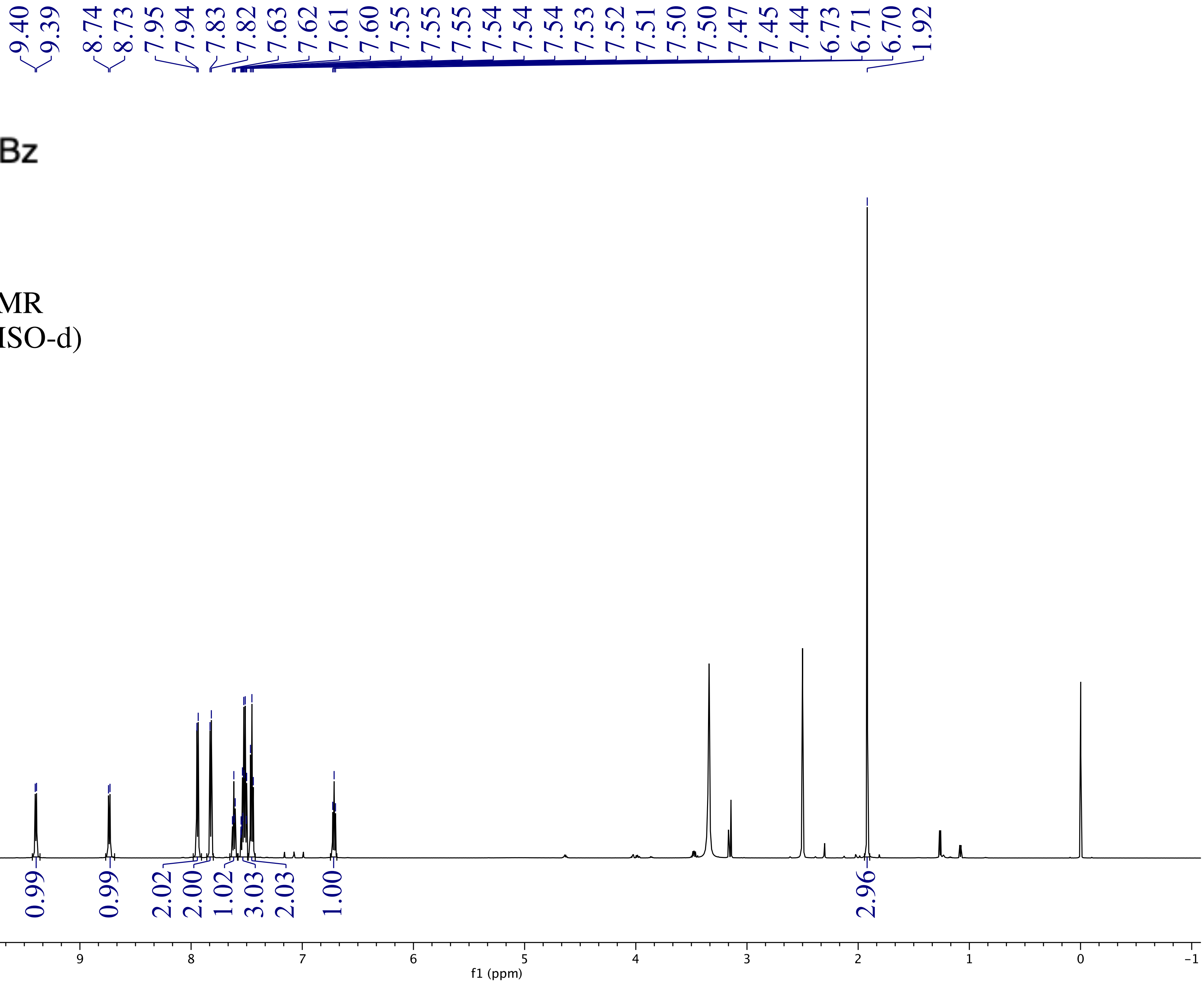

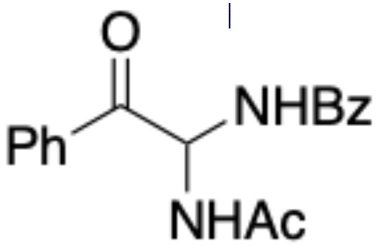

8i for 13C{1H} NMR
(151 MHz, DMSO-d)

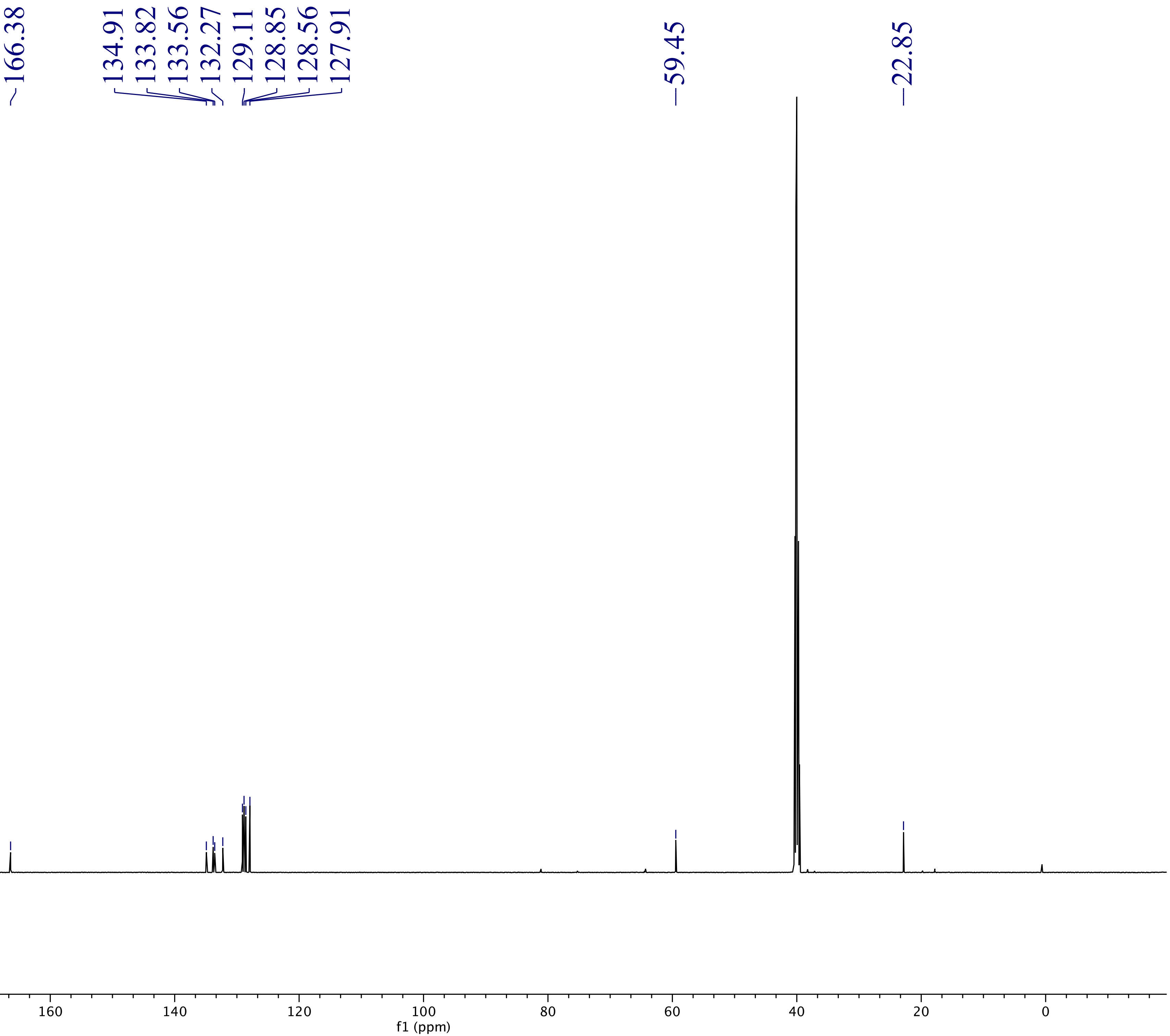

Supplement: Supplementary file 1 — jo3c01469_si_001.pdf [file jo3c01469_si_001.pdf]
